# Aryl Azocyclopropeniums: Minimalist, Visible-Light Photoswitches

**DOI:** 10.1021/jacs.4c01786

**Published:** 2024-03-28

**Authors:** Moritz Fink, Jannik Stäuble, Maïté Weisgerber, Erick M. Carreira

**Affiliations:** Department of Chemistry and Applied Biosciences, Laboratory of Organic Chemistry, ETH Zürich, 8093 Zürich, Switzerland

## Abstract

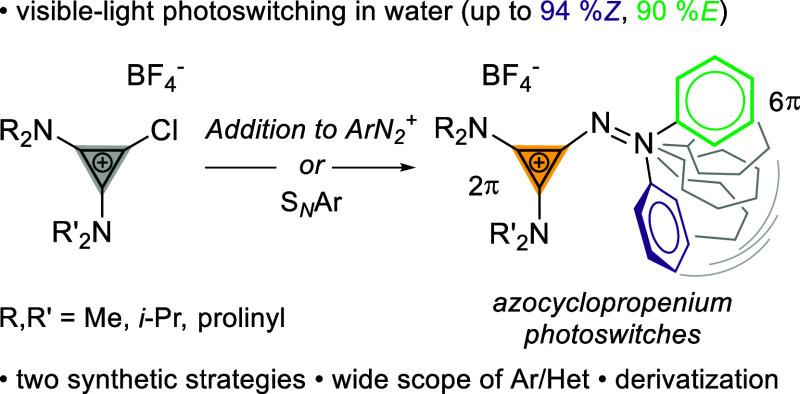

We report convenient syntheses of aryl azocyclopropeniums and a study of their photochemical properties. Incorporation of the smallest arene leads to pronounced redshift of the π–π* absorbance band, compared to azobenzenes. Photoisomerization under purple or green light irradiation affords *Z*- or *E*-isomers in ratios up to 94% *Z* or 90% *E*, and the switches proved stable over multiple irradiation cycles. Thermal half-lives of metastable *Z*-isomers range from minutes to hours in acetonitrile and water. These properties together with the concise, versatile syntheses render aryl azocyclopropeniums exciting additions to the tool kit of readily available molecular photoswitches for wide ranging applications.

Azobenzenes have been used as dyes for almost 200 years.^1^ Only recently light-controlled isomerization^2^ has sparked the development of photopharmacology^3−5^ and photoresponsive materials.^6,7^ These studies are leading to a deeper understanding of the physical chemistry of azoarene photoisomerization. Variation of substituents on phenyl rings or their replacement with heteroarenes enables tuning photoswitching properties, such as *E*/*Z*-ratios,^8^ thermal stability of the metastable isomer,^9,10^ and absorbance wavelength.^11−13^ Numerous azo photoswitches have been designed, synthesized, and studied that encompass a large variety of arenes, most commonly incorporating 6 π- and less prevalently 10 π-electron systems. Interestingly, the smallest 2 π-electron system, the cyclopropenium cation,^14^ has not been studied in azoarenes. Herein, we report two versatile synthetic approaches to azo photoswitches that incorporate cyclopropenium cations (Scheme 1). Their photophysical properties are investigated, and fully reversible, high-yielding photoisomerization under visible-light irradiation is presented.

**Scheme 1:**
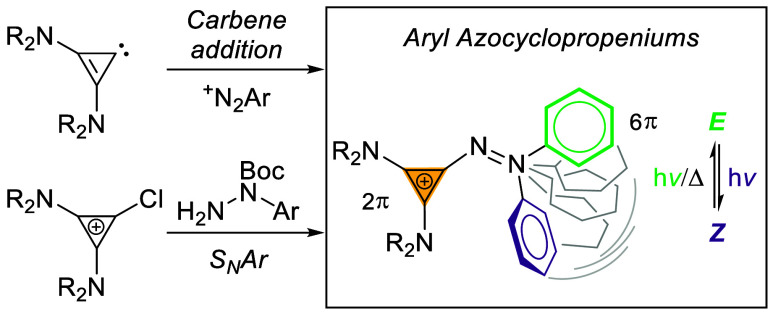
Aryl Azocyclopropenium Photoswitches

Photopharmacology has fueled the search for azoarenes that undergo high-yielding bidirectional switching, display red-shifted absorbance, or have *Z*-isomers with long thermal half-lives (Figure 1). Azoarenes featuring five-membered heteroarenes have enjoyed considerable attention. Herges and Fuchter showed that aryl azoimidazoles and azopyrazoles undergo near-quantitative isomerization and display remarkable bistability.^8,9,15^ Most aryl azoheteroarenes require UV irradiation for photoswitching,^8,9,16−18^ which can limit applications in biology and material sciences.^19,20^ Bistable tetra-*ortho*-substituted azobenzenes, as introduced by Woolley^13,21^ and Hecht,^10^ allow for visible-light photoswitching at longer irradiation times via excitation of the weakly absorbing n−π* transitions. Switches featuring 10 π-arenes have also been studied. As an example, König reported aryl azoindoles with tunable thermal half-lives.^22^ Interestingly, azoarenes featuring 2 π systems remain elusive.

**Figure 1:**
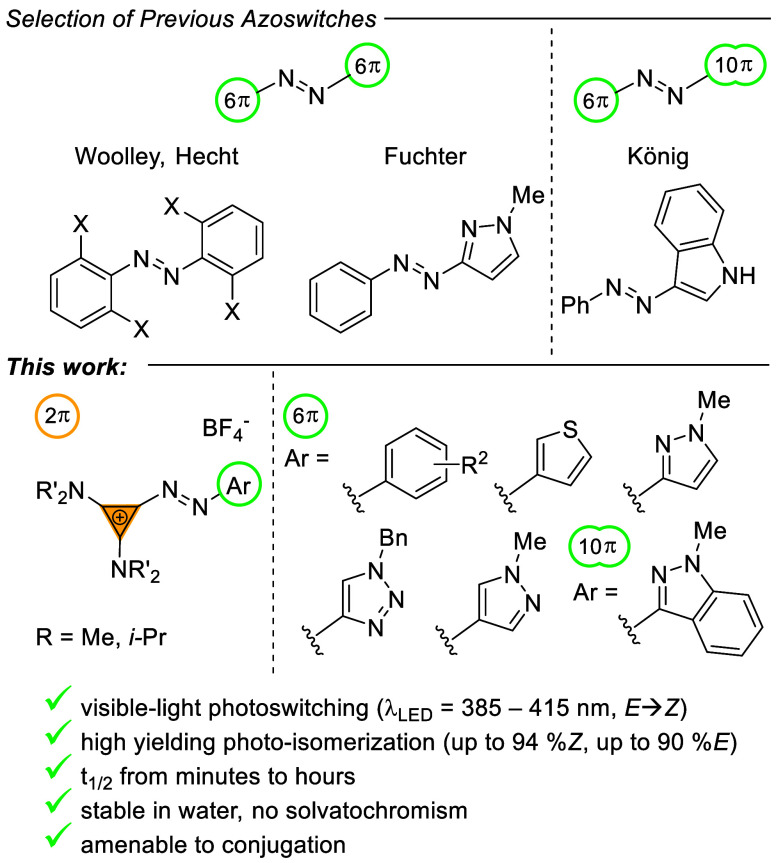
Development of azoarene photoswitches.

Triphenylcyclopropenium tetrafluoroborate was described by Breslow in 1957 and constitutes the first isolable arene with 2 π-electrons.^23^ However, Breslow noted the decomposition of the cation over prolonged storage in methanol. Because strong π-donors elicit stabilizing effects, applications of amine-substituted cyclopropeniums have subsequently dominated.^24,25^ In recent years, the use of cyclopropeniums in materials science and catalysis has been disclosed.^25,26^ For example, Sanford described the application of cyclopropenium-based anolytes in redox flow batteries.^27,28^ Tris(dialkylamino)cyclopropenium salts were employed by Lambert in electrophotocatalytic C–H bond oxidation reactions.^29^

Recently, we have been interested in applying azoarenes to gain spatiotemporal control of biological processes and identifying novel azoarenes and their synthesis.^17,30−32^ To expand the scope of visible-light photoswitches, we envisioned that the electron-accepting character of cyclopropenium would lead to electronic push–pull systems displaying a redshift of the strongly absorbing π–π* bands.^33^ Additionally, we sought to study the stability and photochromism of these 2 π-electron azoarenes that have not been previously examined.

For a synthesis of aryl azocyclopropeniums, we chose chloro bis(dialkylamino)cyclopropenium salts as suitable precursors, which are readily prepared from tetrachlorocyclopropene and dialkylamines.^34^ Feringa has reported access to azoarenes from organolithium reagents and aryl diazonium salts.^35^ Chloro bis(diisopropylamino)cyclopropenium salt **1a** has previously been shown to react with *n*-BuLi, affording carbene **2** (Scheme 2).^36−38^ It was unclear whether **2** would be sufficiently nucleophilic to add onto ArN_2_^+^. Employing literature conditions afforded deeply red phenyl azocyclopropenium tetrafluoroborate **3a** in 20% yield.^17,35^ We surmised that the poor outcome resulted from low nucleophilicity of **2**, compared to organolithiums, compounded by the low solubility of ArN_2_^+^ salt in tetrahydrofuran (THF). Accordingly, chlorocyclopropenium **1a** was treated with *n*-BuLi in THF at −78 °C, and the resulting solution was added to PhN_2_BF_4_ in MeCN, affording **3a** in 60% yield. Under these conditions, **3b**–**d** were prepared (Scheme 2).

**Scheme 2:**
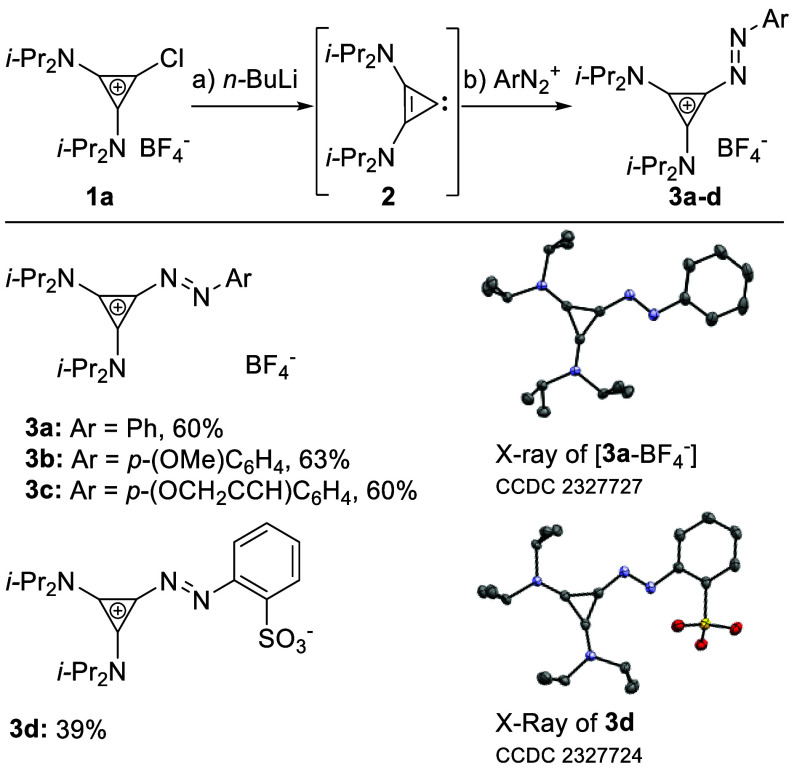
Carbene Addition to Diazonium Salts Reaction conditions: (a) **1a** (1.0 equiv), *n*-BuLi (1.0 equiv), THF, −78 °C; (b) ArN_2_BF_4_ (1.0 equiv), MeCN, −45 °C.

The isolated aryl azocyclopropeniums **3a**–**d** proved stable toward moisture and air, aqueous workup, and column chromatography. Analysis of the X-ray structure of cationic **3a** (Scheme 2) revealed a N=N bond distance of 1.27 Å, matching that of azobenzenes (1.26–1.27 Å).^39^ The C–C bonds within the cyclopropenium are of comparable length (1.37–1.39 Å).^40^

Next, when bisdimethylamino-substituted chlorocyclopropenium **1b** was treated with *n*-BuLi and PhN_2_BF_4_, only decomposition was observed. We concluded that accessing sterically less shielded aryl azocyclopropeniums would necessitate different approaches.

Chlorocyclopropenium cations undergo S_N_Ar with pyridine to form dicationic adducts.^41,42^ We envisioned harnessing this reactivity to activate chlorocyclopropeniums for coupling with Boc-hydrazides **4a**–**r** (Scheme 3).^43−45^

**Scheme 3:**
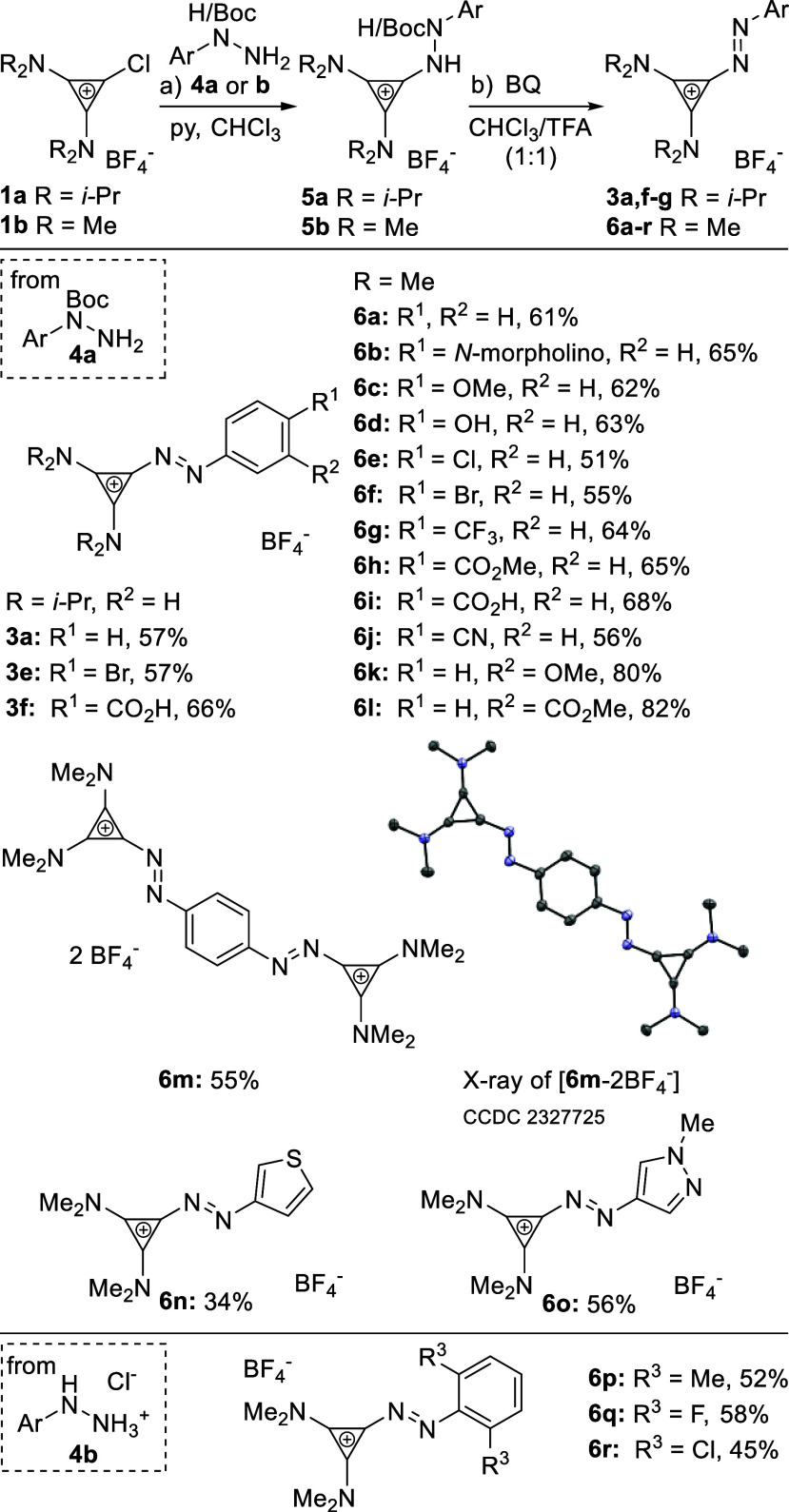
Synthesis of Aryl Azocyclopropenium Salts through S_N_Ar Reaction conditions: (a) **1a** or **1b** (1.0–1.3 equiv), **4a** or **4b** (1.0 equiv), py (1.2 equiv), CHCl_3_ or DCE, 50–80 °C; (b) BQ (2.0 equiv), CHCl_3_ or DCE/TFA (1:1), rt.

Treatment of **1b** with **4a** (Ar = Ph) in the presence of pyridine gave **5b**. Without purification, subjecting **5b** to deprotection (TFA) and *in situ* oxidation with benzoquinone (BQ) furnished **6a** (63%). **3a** could be prepared from chlorodiisopropylaminocyclopropenium **1a** under modified conditions (Scheme 3) in 60% yield, highlighting the method’s generality. The S_N_Ar approach tolerates substrates bearing nucleophilic groups, such as phenol (**6d**) as well as carboxylic acid (**6i**), allowing the synthesis of a variety of different aryl- and heteroaryl-substituted aryl azocyclopropeniums. Hydrazine hydrochlorides were also competent substrates, leading to the formation of **6p-r** bearing two *ortho*-substituents. The stability of **6o** in D_2_O was monitored by ^1^H NMR spectroscopy, and it retained ∼95% purity after seven months at ambient temperature (see SI).

With a variety of aryl azocyclopropeniums, we set out to investigate their photophysical properties (Figure 2, Table 1). The compounds synthesized (**3a**–**f**, **6a**–**r**) displayed λ_max_ = 380–510 nm (Figure 2, SI). Comparing the dominant π–π* absorption of **3a** in DMSO (λ_max_ = 401 nm, see SI) to azobenzene (λ_max_ = 323 nm)^46^ and phenyl azopyrazolium MeOSO_3_^–^ (λ_max_ = 320 nm)^47^ reveals that phenyl azocyclopropenium **3a** is redshifted by ∼80 nm. *para*-Substitution of the phenyl with electron-donating substituents, as in **3b** or **6b**–**d**, augmented the bathochromic shift for λ_max_ (see SI). No hypsochromic shift was observed for compounds incorporating a phenyl ring substituted with electron-withdrawing groups (**6j**).

**Figure 2:**
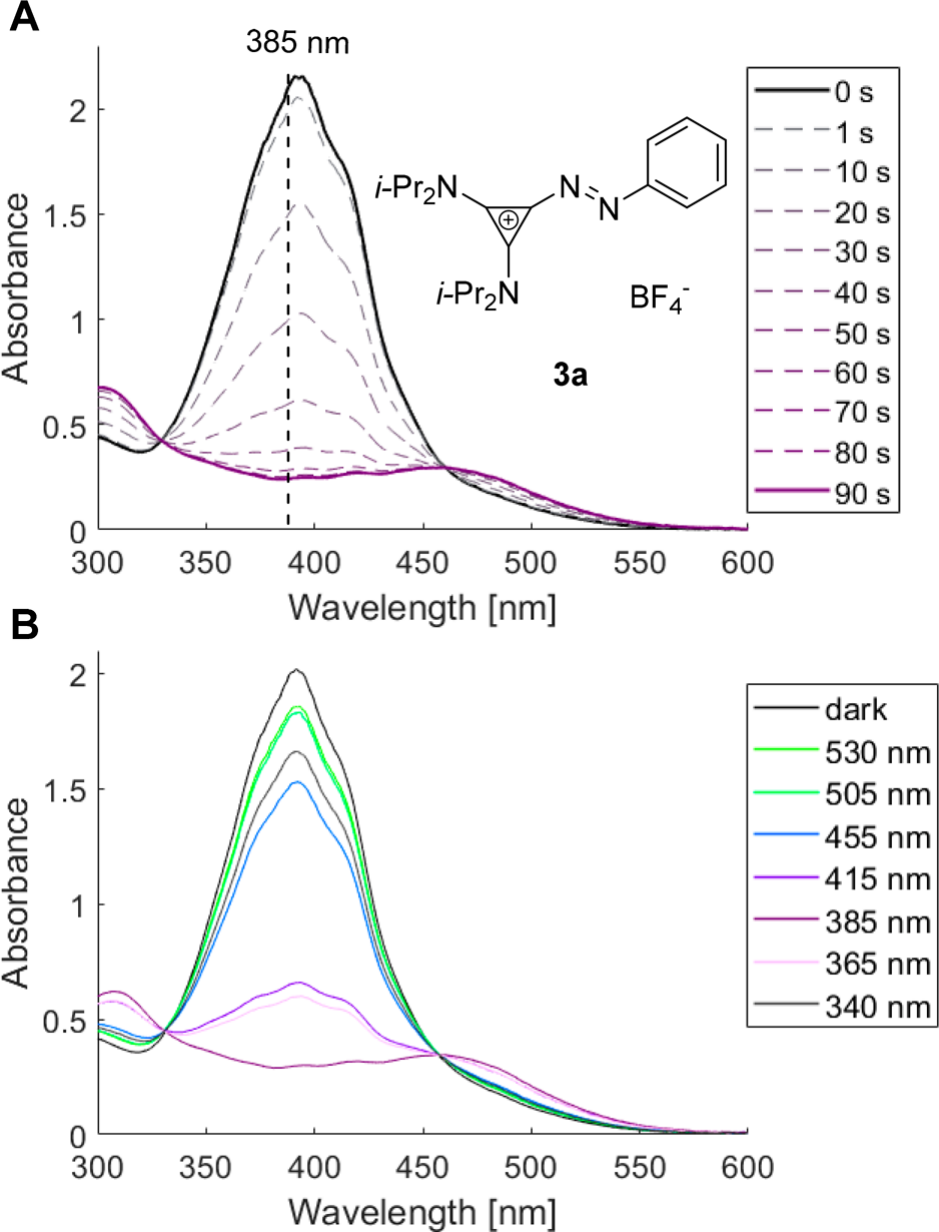
Photochromism of **3a**. (A) Time-dependent UV/vis absorbance of **3a** (100 μM, MeCN) under irradiation at 385 nm; (B) UV/vis absorption spectra of **3a** (100 μM, water) before (dark) and after irradiation at 340–530 nm for at least 20 min.

**Table 1:** Photophysical Properties of Selected Aryl Azocyclopropenium Salts

	λ_max_ (nm) MeCN | H_2_O	*t*_1/2_^a^ (min) MeCN | H_2_O	PSS_385 nm_^c^ % *Z* MeCN | H_2_O	PSS_505 nm_^c^ % *E* MeCN | H_2_O
**3a**	393 | 392	4 | 13	90^d^ | 90^d^	88^d^ | 90^d^^,^^f^
**6a**	386 | 386	73 | 452	92 | 91	90 | 85
**6c**	412 | 410	16 | 93^b^	81^e^ | 80^e^	82^f^ | 81^f^
**6e**	392 | 392	5 | 361	88^d^ | 90	82^d^ | 84
**6l**	387 | 385	17 | 309	83 | 86	87 | 86
**6n**	387 | 388	170 | 340	94 | 92	87 | 87
**6o**	393 | 394	337 | 223^b^	90^d^ | 90	90^d^^,^^f^ | 81

a Determined at 25 °C.

b Determined in water at 37 °C.

c Determined by HPLC analysis.

d Estimated based on absorbance spectra; for details see SI.

e PSS_415 nm_.

f PSS_530 nm_.

The photochromism of prototypical aryl azocyclopropenium **3a** was investigated by irradiating a solution in MeCN (λ_LED_ = 385 nm, 100 μM) close to λ_max_. Within 90 s, the photostationary state (PSS) was reached (Figure 2). Performing this procedure at different wavelengths (340–530 nm), we observed reversible changes in absorption intensity and band position, indicative of *E*/*Z*-isomerization. Photophysical properties of a collection of (hetero)aryl azocylopropeniums can be found in Table 1 (for additional examples and photophysical characterization, see SI).

Generally, irradiation at 385 nm in MeCN afforded PSSs displaying the highest *Z*-isomer content (% *Z*, Table 1). Most efficient switching was observed for thiophenyl azocyclopropenium **6n**, affording 94% *Z*, as determined by HPLC analysis. Electron-rich arene **6c** (*p*-OMe), displaying red-shifted absorbance (λ_max_ = 412 nm), gives the highest % *Z* under violet light irradiation (81%, 415 nm). *Z*-**6c** and *Z*-**6n** are switched to 80–90% *E*, using green light irradiation (505–530 nm). Importantly, no solvatochromism was observed for any of the structures studied (Δλ_max_ < 10 nm, water vs MeCN), and PSS-*E*/*Z*-ratios remained largely unaffected. We did not detect photochromism for highly red-shifted **6b** and **6m** (λ_max_ > 500 nm).^48,49^

Thermal half-lives of the photochemically generated *Z*-isomers, measured in MeCN or water, range from minutes to hours (Table 1 and SI). The values were solvent-dependent, and in all cases, longer thermal half-lives were observed in water. The largest solvent effect was measured for *Z*-**6e** (MeCN: 5 min vs water: 361 min). Electron-rich azocyclopropenium **6c** displayed a reduced thermal half-life compared to phenyl-substituted **6a** (MeCN: 16 min vs 73 min; H_2_O: 93 min vs 452 min). The same trend was observed when comparing NMe_2_- with N(*i*-Pr)_2_-substituted aryl azocyclopropeniums, as can be seen for **3a** and **6a** (MeCN: 4 min vs 73 min; H_2_O: 13 min vs 452 min).

Dreuw and Wachtveitl have reported that *Z-*2-thiophenylazobenzene adopts a perpendicular disposition between phenyl and thiophene.^50^ Such T-shaped arrangement features a *S*-lone pair−π interaction that impacts the photoisomerization time scale. We synthesized pyrazol-4-yl-, indazol-3-yl-, and triazol-4-yl-azocyclopropeniums **6s**–**u** to examine potential interactions between the N-lone pair and the cyclopropenium cation in the *Z*-isomer (Scheme 4).

**Scheme 4:**
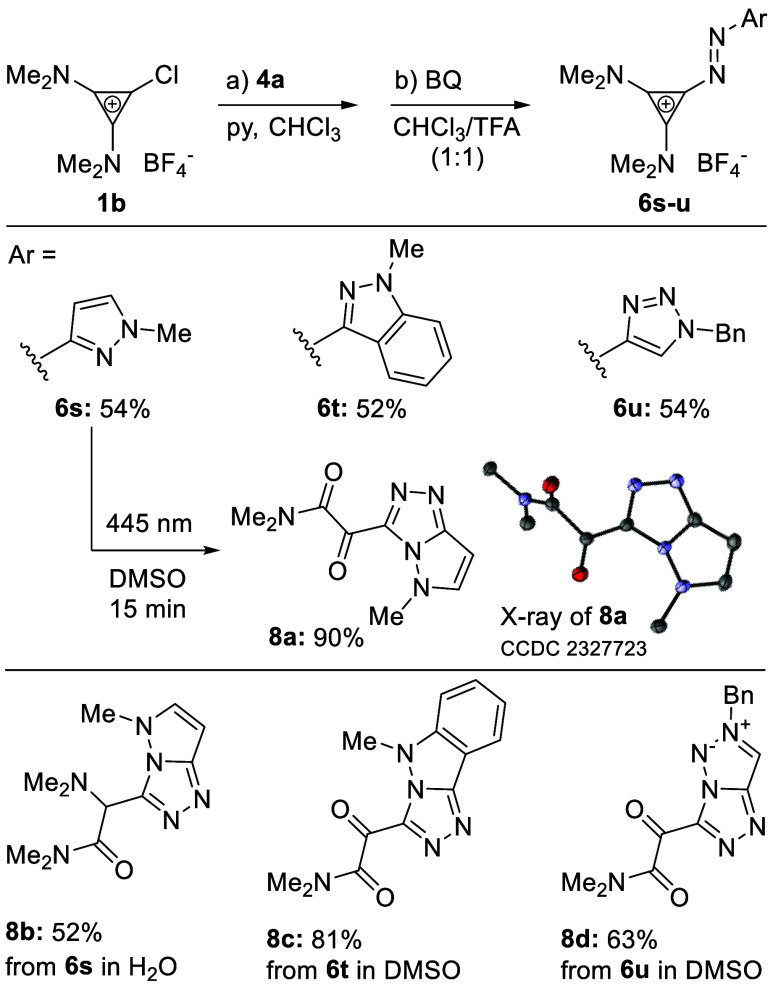
Photochemically Induced Reactivity Reaction conditions: (a) **1b** (1.3 equiv), **4a** (1.0 equiv), pyridine (1.2 equiv), CHCl_3_, 50 °C; (b) BQ (2.0 equiv), CHCl_3_/TFA (1:1), rt.

Irradiation of a sample of **6s** with green light (505 nm) in DMSO caused the disappearance of the yellow color (see SI). When the reaction was performed on a preparative scale, we isolated α-keto amide **8a**, as determined by NMR spectroscopy and X-ray analysis (Scheme 4). We hypothesize that formation of **8a** involves *E*/*Z*-isomerization, skeletal rearrangement, and oxidation by DMSO solvent (for the detailed mechanism see SI). When the reaction was performed in water, α-amino amide **8b** was isolated. Similar reactivity was observed for indazole- and triazole-substituted cyclopropenium azo arenes **6t** and **6u**, resulting in the formation of heteropentalenes **8c** and **8d**, respectively. To showcase that this reactivity is highly dependent on the presence of nitrogen substitution as found in **6s**, photostability of **3a**, **6a**, **6c**, and **6o** was investigated and validated (see SI).^51^

We extended our study on the stability of aryl azocyclopropeniums in PBS buffer and bacterial growth medium, wherein **3a** and **6c** displayed good stability. Half-lives in the presence of 1–10 mM glutathione ranged from minutes to >2 h. Photophysical properties of **6o** (100 mM, PBS buffer) were not affected by other ions in the buffer (see SI).^52^

Application of aryl azocyclopropeniumss in probes requires handles for further manipulation. We demonstrate that alkyne **3c** and carboxylic acid **3f** undergo Cu-catalyzed click reaction^53^ and T3P-mediated amide bond formation,^54^ respectively (Scheme 5). Thus, aryl azocyclopropeniums can be synthetically manipulated by employing routinely applied transformations in chemical biology.

**Scheme 5:**
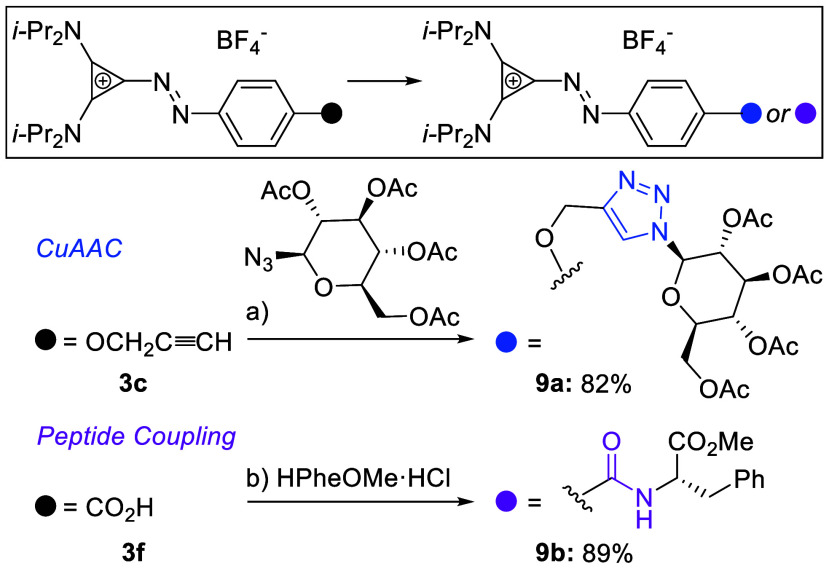
Aryl Azocyclopropenium Derivatization Reaction conditions: (a) **3c** (1.0 equiv), azide (1.0 equiv), sodium ascorbate (4.0 equiv), CuSO_4_ (10 mol %), MeCN/water, rt; (b) **3f** (1.0 equiv), H-l-PheOMe·HCl (1.5 equiv), T3P (2.0 equiv), NEt_3_ (5.1 equiv), MeCN, rt.

We turned our attention to the introduction of a handle for derivatization of the cyclopropenium. Ideally, this would require an unsymmetrically substituted chloro bisaminocyclopropenium bearing a selectively addressable functional group, which has not been reported to date. With the protocol we developed, multigram quantities of **1b** are synthesized, which we envisioned as a suitable precursor to access unsymmetrically substituted chloro bisaminocyclopropeniums.

Accordingly, **1b** was treated with L-HProO*t*-Bu, and the condensation product was observed, as determined by NMR analysis. Treatment of the unpurified material with KOH (2 M in water, 70 °C) led to **10** in 70% yield. Chlorocyclopropenium **1c** was accessible in 87% yield from **10**. Subsequent condensation of **1c** with Boc-hydrazide followed by deprotection and oxidation afforded **11a** and **11b** (Scheme 6). Analysis of the photophysical characteristics of **11a** revealed λ_max_ and PSS-*E*/*Z*-ratios comparable to those of **6c** (see SI). Incorporation of the *N*-prolinyl substituent allows for an increase in structural complexity without affecting the beneficial photophysical properties of the underlying switch.

**Scheme 6:**
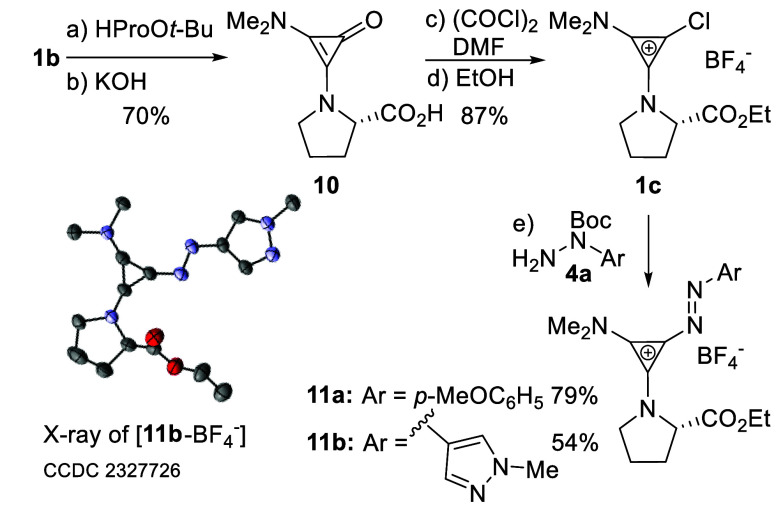
Chlorocyclopropenium Derivatization Reaction conditions: (a) **1b** (1.0 equiv), H-l-ProO*t*-Bu (1.1 equiv), NaHCO_3_ (2.0 equiv), rt; (b) KOH (2 M), H_2_O, 70 °C; (c) (COCl)_2_ (3.0 equiv), DMF (cat.), 0 °C to rt; (d) EtOH, rt; (e) **1c** (1.2 equiv), **4a** (1.0 equiv), py (1.2 equiv), CHCl_3_, 50 °C; then BQ (2.0 equiv), CHCl_3_/TFA (1:1), rt.

We have presented two protocols to access a variety of (hetero)aryl azocyclopropeniums. Analysis of their photochemical properties revealed high-yielding photoisomerism under visible-light irradiation with thermal half-lives in the range of minutes to hours. Additional interesting aspects of these new switches include the absence of solvatochromism and longer thermal half-lives in water than in MeCN. Owing to their beneficial photophysical properties, unique geometry, and readily available functionalization protocols, aryl azocyclopropeniums are set to serve as a useful expansion to the scope of water-soluble photoswitches.

## Supporting Information Available

Details on the syntheses and analyses of presented compounds, NMR spectra, crystallographic data, and photophysical measurements, including Figures S1–S177, Schemes S1 and S2, as well as Tables T1–T5 (PDF)
