# Supplementary material for: Aryl Azocyclopropeniums: Minimalist, Visible-Light Photoswitches
Source: J Am Chem Soc. 2024 Mar 28;146(14):9519–25. doi: 10.1021/jacs.4c01786 (PMC11010232; doi:10.1021/jacs.4c01786)
Supplement: Supplementary file 1 — ja4c01786_si_001.pdf [file ja4c01786_si_001.pdf]

Supporting Information

Aryl Azocyclopropeniums: Minimalist, Visible-Light Photoswitches

Moritz Fink, Jannik Stäuble, Maïté Weisgerber, and Erick M. Carreira*

Laboratorium für Organische Chemie Eidgenössische Technische Hochschule Zürich 8093 Zürich, Switzerland

Email: erickm.carreira@org.chem.ethz.ch

Table of Contents

Materials and Methods 2
Analytical Data 4
Preparation of Chlorocyclopropenium Salts 5
Preparation of Aryl Azocyclopropenium Salts from Diazonium Salts 8
Preparation of *N*-Boc-*N*-Arylhydrazides 13
Preparation of Aryl Azocyclopropenium Salts from *N*-Boc-*N*-Arylhydrazides 27
Rearrangement of Heteroaryl Azocyclopropenium Salts 58
Derivatization of Aryl Azocyclopropenium Salts 63
Single Crystal X-Ray Data 73
Determination of Photo-physical Data 86
  Overview about Photophysical Data 88
  UV/vis Absorption Spectra 90
  Thermal Relaxation Plots 111
Stability Experiments 126
  Dynamic Scanning Calorimetry (DSC) 126
  Thermal Stability of 6o in D_2O 129
  Stability in Different Biologically Relevant Media 130
  Photostability Experiments 135
NMR Spectra 138
HPLC Traces 231
References 273

Materials and Methods

General Information

Unless otherwise noted, all reactions were carried out under ambient atmosphere. Reactions, requiring oxygen- and water-free conditions, were performed under nitrogen atmosphere in glassware dried with a heat gun (650°C) under high vacuum (<1 mbar). Syringes for transferring anhydrous solvents or reagents were purged trice with nitrogen prior to use. All commercially available reagents were obtained from suppliers (ABCR GmbH, Apollo Scientific Ltd., Sigma Aldrich, Fluorochem Ltd) and used without further purification unless otherwise noted. Anhydrous solvents, stored over molecular sieves, were purchased from Acros Organics B.V.B.A. and used as received. Benzenediazonium tetrafluoroborate,[1] 4-methoxybenzenediazonium tetrafluoroborate,[2,3] 4-(prop-2-yn-1-yloxy)benzenediazonium tetrafluoroborate,[3] 2-diazoniumbenzenesulfonate,[4] 4-iodo-1-methyl-1*H*-pyrazole,[5] (2,6-dimethylphenyl)hydrazine hydrochloride,[6] 3-iodo-1-methyl-1*H*-indazole,[7] and tetraacetyl-β-glucopyranosyl azide[8] were synthesized according to previously reported procedures.

Safety Statement

No unexpected or unusually high safety hazards were encountered during this work. Diazonium tetrafluoroborate salts as described in this report, although reported to be stable, are potentially energetic.[9] While no hazardous incidences occurred in our laboratory during this work, care should be taken at any point by using the appropriate personal protective equipment, safety glasses, lab coat and, possibly, blast shields. The diazonium salts should not be prepared above gram scale. It is noteworthy that aryl azocyclopropeniums **6m**, **6o**, **6s**, and **6u** as well as heteropentalenes **8a**, **8b**, **8c**, and **8d** prepared in this manuscript display a C/N ratio < 3, which might be taken as an indicator for potential energetic behavior.

To address this concern, the thermal decomposition of **3a** and **6o** was investigated by DSC and benchmarked against modern industrial standards.[10–12] Although the results do not predict explosive or shock sensitive properties, the compounds should not be heated above 200 °C as exothermic decomposition was detected.

When working with LEDs, direct eye contact with the light source should be avoided to prevent eye damage.

LED Equipment. Irradiation was performed with a fiber-coupled LED set-up, purchased from ThorLabs Inc., including the following LEDs: 340 nm LED (M340F3), 365 nm LED (M365FP1), 385 nm LED (M385FP1), 415 nm LED (M415F3), 455 nm LED (M455F3), 505 nm LED (M505F3), 530 nm LED (M530F2). The light beam was directed with a glass fiber (400 UMT custom MUC) and the LEDs were powered by a LEDD1B-Driver, operated at 1.2 A amperage

and full power, or a DC4100 Four-Channel LED Driver, coupled to a DC4100-HUB LED Connector Hub and operated at 0.7 A amperage for the 340 nm LED or 1 A amperage for all other LEDs.

Photoreactor. The blue-light photoreactor was custom made in coordination with the mechanical workshop in the Department of Chemistry and Applied Biosciences at ETH Zürich. The reactor features ten 35 W blue LEDs, equally spaced in a circle design, powered by a 10.3 A power supply, emitting light with a maximum intensity at 446 nm. The LEDs were water-cooled and further cooled by built-in fans to maintain a maximal working temperature of 40°C.[13]

Chromatography

Analytical thin layer chromatography (TLC) was performed on Merck TLC Silica gel 60 F254 aluminum plates and visualized with 254 nm light and potassium permanganate or ceric ammonium molybdate staining solutions followed by heating. Organic solutions were concentrated by rotary evaporation at 40 °C. Purification of reaction products was carried out by flash column chromatography using Brunschwig silica 32-63, 60Å under 0.2 bar overpressure. Automated chromatography was performed on a Büchi Pure C-810 Flash machine using FlashPure silica cartridges.

$KMnO_4$ solution: $KMnO_4$ (3.0 g), 5 drops of conc. H_2SO_4 in water (300 mL).

Ceric ammonium molybdate solution: $Ce(SO_4)_2$ (5 g), $(NH_4)_6Mo_7O_{24}$*$4H_2O$ (25 g), conc. H_2SO_4 (50 mL) in H_2O (450 mL).

Buffer recipe PBS: NaCl (1.6 g), KCl (0.04 g), Na_2HPO_4*$2H_2O$ (0.361 g), KH_2PO_4 (0.048 g), 200 mL purified water, pH adjusted to 7.40.

Analytical Data

NMR. ^{1}H NMR, ^{13}C NMR, and ^{19}F NMR spectra were recorded at ambient temperature on Bruker Neo 500 MHz and 400 MHz spectrometers as well as Bruker Ascend 400 MHz and Oxford 400 MHz spectrometers. Chemical shifts are reported in ppm with the solvent resonance as the reference unless noted otherwise ($CDCl_3$, ^{1}H: δ = 7.26 ppm, ^{13}C: δ = 77.2 ppm; CD_3CN, ^{1}H: δ = 1.94 ppm, ^{13}C: δ = 1.3, 118.3 ppm). Peaks are reported as (s = singlet, d = doublet, dd = doublet of doublets, ddd = doublet od doublets of doublets, t = triplet, dt = doublet of triplets, tt = triplet of triplets, q = quartet, sept. = septet, m = multiplet or unresolved, br = broad signal, coupling constant(s) in Hz, integration).

Infrared Spectra (IR) were recorded neat on a Perkin-Elmer Spectrum Two FT-IR spectrometer. The main peaks are reported as absorption maxima (cm^{-1}).

Mass Spectrometry. High resolution mass spectrometric data were obtained at the mass spectrometry service operated by the Laboratory of Organic Chemistry at the ETHZ on a Bruker Daltonics maXis ESI-QTOF machine and are reported as (m/z).

X-Ray Crystallographic Analysis. Measurement and analysis of X-Ray crystallographic data was performed by Dr. Michael Wörle, Dr. Nils Trapp, and Michael Solar (all Small Molecule Crystallography Center, ETH Zurich). Details are listed below.

UV VIS Spectroscopy. UV-Vis absorption spectra were recorded on a Mettler Toledo UV5Bio spectrometer in the indicated solvents at 100 μM concentration, unless stated otherwise. For details see below. Measurements at elevated temperature or requiring constant stirring were performed using a CuveT thermostat with integrated stir plate.

Analytical HPLC. Separation of *E* and *Z* isomers of aryl azocyclopropenium salts was performed on a Waters e2965 separation module equipped with a 2998 PDA detector. To this end, 10 μL of the solution were injected onto a Reproshper 100 C12 column (5 um, 125x4.6 mm), eluting with MeCN/water containing 0.1% formic acid.

Preparation of Chlorocyclopropenium Salts

Chloro-bis(diisopropylamino)cyclopropenium tetrafluoroborate (1a)

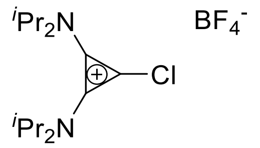

1a

Compound **1a** was synthesized according to modified literature procedures:[14–16]

Over the course of 20 min *N*,*N*-diisopropylamine (21 mL, 150 mmol, 6.1 equiv) was added to a stirred solution of tetrachlorocyclopropene (3.0 mL, 24 mmol, 1.0 equiv) in CH_2Cl_2 (95 mL) at 0°C. After a further 30 min at 0°C, the reaction was warmed to ambient temperature and stirring continued for another 2.5 h. $NaBF_4$ (4.0 g, 36 mmol, 1.5 equiv) was added and the suspension stirred for 14 h. The resulting mixture was washed with half-sat. NH_4Cl-sln. (2x140 mL) and water (70 mL), dried over Na_2SO_4, and concentrated under reduced pressure. The product was precipitated from CH_2Cl_2 by slow addition of Et_2O. Decantation and washing with Et_2O (2x20 mL) afforded **1a** (7.8 g, 22 mmol, 89%) as colorless solid.

^{1}H-NMR (500 MHz, CD_3CN): δ / ppm = 3.99 (sept., J = 7.0 Hz, 2H), 3.94 (sept., J = 7.0 Hz, 2H), 1.33 (d, J = 7.0 Hz, 12H), 1.32 (d, J = 7.0 Hz, 12H).

^{13}C-NMR (126 MHz, CD_3CN): δ / ppm = 133.5, 93.9, 58.8, 49.0, 22.7, 20.6.

^{19}F-NMR (471 MHz, CD_3CN): δ / ppm = -152.

IR (Diamond-ATR, neat): $\tilde{\nu}$ / cm^{-1} = 2983, 2939, 1927, 1581, 1453, 1424, 1394, 1375, 1350, 1340, 1279, 1208, 1182, 1154, 1140, 1094, 1044, 1024, 935, 893, 748, 658.

HRMS (ESI) calcd. for $C_{15}H_{28}ClN_2$ [M-BF$_4$]$^+$: 271.1936, found 271.1931.

Chloro-bis(dimethylamino)cyclopropenium tetrafluoroborate (1b)

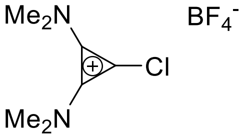

1b

Compound **1b** was synthesized according to modified literature procedures:[17–22]

A flame-dried 250 mL round-bottom flask equipped with a magnetic stir bar was set under N_2 atmosphere, charged with tetrachlorocyclopropene (8.7 g, 6.0 mL, 49 mmol, 1.0 equiv) in dry CH_2Cl_2 (100 mL, 0.49 M), and cooled to 0 °C. *N*,*N*-Dimethyltrimethylsilylamine (19 g, 26 mL, 160 mmol, 3.3 equiv) was added over the course of 10 min. After 1 h, the cooling was removed, and stirring continued for 2 h. The reaction mixture was concentrated under reduced pressure, and KOH solution (65 mL, 15%, [w/w]) added. The resulting mixture was stirred at 70 °C for 3h. After cooling to ambient temperature, the pH was adjusted to 7 by addition of conc. HCl (18 mL, 37%, [w/w]) and sat. $NaHCO_3$-sln. NaCl (37 g) was added, and the mixture stirred for 10 min. The resulting suspension was filtered, and the aq. phase extracted with CH_2Cl_2 (8x100 mL). The combined org. phases were dried over Na_2SO_4 and concentrated under reduced pressure. The remaining yellow solid was quickly dried under high vac., set under N_2 atmosphere, and dissolved in dry CH_2Cl_2 (50 mL). The solution was cooled to 0 °C and oxalylchloride (7.4 g, 5.0 mL, 58 mmol, 1.2 equiv) added dropwise over the course of 3 min. The cooling was removed, the reaction stirred at ambient temperature for 15 min and immediately concentrated under reduced pressure. The resulting solids were dissolved in dry MeCN (50 mL) and treated with $NaBF_4$ (6.4 g, 58 mmol, 1.2 equiv) at ambient temperature for 17 h. The suspension was filtered over a short plug of silica and the plug washed with CH_2Cl_2/MeOH (9:1). The solution was concentrated, and the crude product adsorbed onto silica for flash column chromatography (SiO_2, CH_2Cl_2:MeOH = 95:5, R_f = 0.25). The obtained solids were dissolved in CH_2Cl_2 (25 mL) and the product precipitated by addition of Et_2O (100 mL). The resulting suspension was stored at 5 °C for 16 h. Decantation and washing with Et_2O (2x5 mL) afforded **1b** (8.0 g, 32 mmol, 66%) as colorless solid.

^{1}H-NMR (400 MHz, $CDCl_3$): δ / ppm = 3.29 (s, 1H), 3.26 (s, 1H).

^{13}C-NMR (101 MHz, $CDCl_3$): δ / ppm = 134.8, 91.9, 42.4, 41.7.

^{19}F-NMR (377 MHz, $CDCl_3$): δ / ppm = -154.

IR (Diamond-ATR, neat): $\tilde{\nu}$ / cm^{-1} = 2950, 1954, 1800, 1634, 1570, 1452, 1418, 1410, 1391, 1278, 1238, 1213, 1096, 1030, 797, 773, 728.

HRMS (ESI) calcd. for $C_7H_{12}ClN_2$ $[M-BF_4]^+$: 159.0684, found 159.0684.

Literature references:

Synthesis of tris-*N*,*N*-dimethylamino cyclopropenium chloride.[17,18]

Bis(dimethylamino)cyclopropenone.[19,20]

Chloro-bis(*N*,*N*-dimethylamino)-cyclopropenium tetrafluoroborate (**1b**).[21,22]

Preparation of Aryl Azocyclopropenium Salts from Diazonium Salts

General Procedure 1.1: Preparation of *N*,*N*-Bis(diisopropylamino)cyclopropenylidene (**2**)[23]

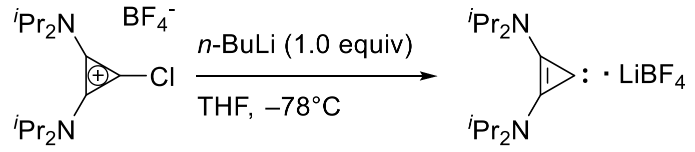

1a **2** (·$LiBF_4$)

A flame-dried 10 mL Schlenk tube equipped with a magnetic stir bar was set under N_2 atmosphere and charged with chloro-bis(diisopropylamino)cyclopropenium tetrafluoroborate (**1a**) (360 mg, 1.0 mmol, 1.0 equiv) and dry THF (2.7 mL, final concentration: 0.3 M). The suspension was cooled to –78°C and *n*-BuLi (0.65 mL, 1.6 M in hexanes, 1.0 mmol, 1.0 equiv) added dropwise. After 30 min, the cooling was removed, the solution stirred at ambient temperature for 10 min and used directly in the next step.

General Procedure 1.2: Preparation of 1,2-Bis(diisopropylamino)-3-aryldiazenyl cyclopropenium salts (**3**)

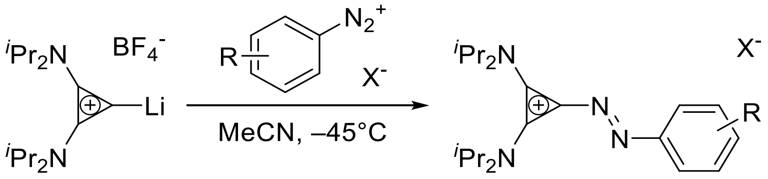

2 **3**

A flame-dried 5 mL flask was charged with diazonium salt (0.30 mmol, 1.0 equiv) and flushed with N_2 for 10 min. The flask was cooled to –45°C on a bath of dry ice and MeCN. Dry MeCN (2 mL) was added at -45°C, followed by a stock solution of lithium diisopropylamino cyclopropylidene (1.0 mL, 0.3 M in THF:hexanes = 4:1, 0.30 mmol, 1.0 equiv). The reaction was allowed to reach –30°C over the course of 1 h. The cooling was removed, and the reaction stirred at ambient temperature for another 30 min. The reaction was quenched by addition of acetic acid (0.020 mL, 0.35 mmol, 1.2 equiv) and concentrated under reduced pressure. The residue was diluted with $CHCl_3$/*i*-PrOH (5 mL, 3:1, [v/v]), washed with sat. $NaHCO_3$-sln. (2 mL) and $NaBF_4$-sln. (2 mL, 10%, [w/w]), dried over Na_2SO_4, and concentrated under reduced pressure. Flash column chromatography (SiO_2, EtOAc, then CH_2Cl_2:MeOH = 1:0 to 95:5) and precipitation from $CHCl_3$/Et_2O afforded aryl azocyclopropenium salts in spectroscopical purity.

(*E*)-1,2-Bis(*N,N*-diisopropylamino)-3-phenyldiazenylcyclopropenium tetrafluoroborate (3a)

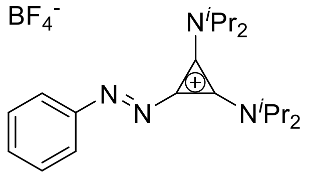

3a

3a was prepared according to **GP1.2** from benzenediazonium tetrafluoroborate (58 mg, 0.30 mmol, 1.0 equiv) and *N*,*N*-bis(diisopropylamino)cyclopropenylidene (**2**) (1.0 mL, 0.3 M in THF:hexanes = 4:1, 0.30 mmol, 1.0 equiv, **GP1.1**) in dry MeCN (2 mL, 0.1 M final concentration). Purification by flash column chromatography (SiO_2, EtOAc, then CH_2Cl_2:MeOH = 1:0 to 95:5, R_f = 0.20 in CH_2Cl_2:MeOH = 98:2) and precipitation from $CHCl_3$/Et_2O afforded **3a** (77 mg, 0.18 mmol, 60%) as red solid.

^{1}H-NMR (500 MHz, CD_3CN): δ / ppm = 7.99 – 7.95 (m, 2H), 7.70 – 7.66 (m, 1H), 7.65 – 7.61 (m, 2H), 4.16 (sept., *J* = 7.0 Hz, 2H), 4.07 (sept., *J* = 7.0 Hz, 2H), 1.47 (d, *J* = 7.0 Hz, 12H), 1.41 (d, *J* = 7.0 Hz, 12H).

^{13}C-NMR (126 MHz, CD_3CN): δ / ppm = 154.1, 135.4, 130.9, 129.1, 124.3, 110.9, 59.3, 50.1, 21.5, 20.6.

^{19}F-NMR (471 MHz, CD_3CN): δ / ppm = -152.

IR (Diamond-ATR, neat): $\tilde{\nu}$ / cm^{-1} = 2984, 2940, 2880, 1871, 1582, 1461, 1400, 1376, 1349, 1310, 1284, 1209, 1184, 1162, 1139, 1091, 1047, 1033, 999, 954, 929, 899, 886, 831, 773, 751, 685, 637, 607.

HRMS (ESI) calcd. for $C_{21}H_{33}N_4$ $[M-BF_4]^+$: 341.2700, found 341.2698.

(E)-1,2-Bis(N,N-diisopropylamino)-3-((3-methoxyphenyl)diazenyl) cyclopropenium tetrafluoroborate (3b)

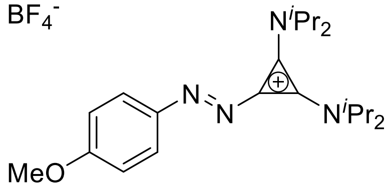

3b

3b was prepared according to **GP1.2** using 4-methoxybenzenediazonium tetrafluoroborate (67 mg, 0.30 mmol, 1.0 equiv) and *N*,*N*-bis(diisopropylamino)cyclopropenylidene (**2**) (1.0 mL, 0.3 M in THF:hexanes = 4:1, 0.30 mmol, 1.0 equiv, **GP1.1**) in dry MeCN (2 mL, 0.1 M final concentration). Purification by flash column chromatography (SiO_2, EtOAc, then CH_2Cl_2:MeOH = 1:0 to 95:5, R_f = 0.20 in CH_2Cl_2:MeOH = 98:2) and precipitation from $CHCl_3/Et_2O$ afforded **3b** (87 mg, 0.19 mmol, 63%) as red solid.

^{1}H-NMR (500 MHz, CD_3CN): δ / ppm = 7.99 – 7.94 (m, 2H), 7.15 – 7.11 (m, 2H), 4.14 (sept., J = 6.5 Hz, 2H), 4.04 (sept., J = 7.0 Hz, 2H), 3.93 (s, 3H), 1.46 (d, J = 7.0 Hz, 12H), 1.40 (d, J = 6.5 Hz, 12H).

^{13}C-NMR (126 MHz, CD_3CN): δ / ppm = 166.3, 148.7, 128.5, 127.0, 116.2, 111.6, 58.9, 56.9, 49.9, 21.6, 20.7.

^{19}F-NMR (471 MHz, CD_3CN): δ / ppm = -152.

IR (Diamond-ATR, neat): $\tilde{\nu}$ / cm^{-1} = 2982, 2940, 2846, 1876, 1595, 1575, 1500, 1462, 1396, 1375, 1351, 1316, 1298, 1259, 1209, 1183, 1138, 1091, 1048, 1035, 1020, 957, 900, 888, 840, 808, 776, 742, 688, 638, 622, 606.

HRMS (ESI) calcd. for $C_{22}H_{35}N_4O$ [M-BF_4]$^+$: 371.2805, found 371.2805.

(E)-1,2-Bis(N,N-diisopropylamino)-3-((4-(prop-2-yn-1-yloxy)phenyl) diazenyl)cyclopropenium tetrafluoroborate (3c)

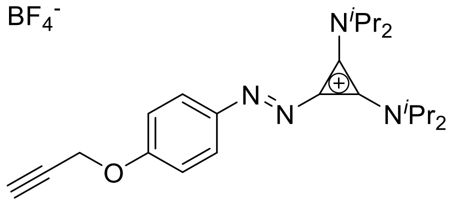

3c

3c was prepared according to **GP1.2** using 4-(prop-2-yn-1-yloxy)benzenediazonium tetrafluoroborate (250 mg, 1.0 mmol, 1.0 equiv) and *N,N*-bis(diisopropylamino) cyclopropenylidene (**2**) (3.3 mL, 0.3 M in THF:hexanes = 4:1, 1.0 mmol, 1.0 equiv, **GP1.1**) in dry MeCN (6.6 mL, 0.1 M final concentration). Purification by flash column chromatography (SiO_2, EtOAc, then CH_2Cl_2:MeOH = 1:0 to 95:5, R_f = 0.30 in CH_2Cl_2:MeOH = 97:3) and precipitation from $CHCl_3/Et_2O$ afforded **3c** (290 mg, 0.60 mmol, 60%) as red solid.

^{1}H-NMR (400 MHz, CD_3CN): δ / ppm = 8.03 – 7.95 (m, 2H), 7.22 – 7.16 (m, 2H), 4.89 (d, J = 2.5 Hz, 2H), 4.15 (sept., J = 7.0 Hz, 2H), 4.05 (sept., J = 7.0 Hz, 2H), 2.90f (t, J = 2.5 Hz, 1H), 1.46 (d, J = 7.0 Hz, 12H), 1.40 (d, J = 7.0 Hz, 12H).

^{13}C-NMR (101 MHz, CD_3CN): δ / ppm = 163.8, 149.1, 128.6, 126.8, 117.0, 111.4, 78.7, 77.7, 59.0, 57.3, 49.9, 21.6, 20.7.

^{19}F-NMR (377 MHz, CD_3CN): δ / ppm = -152.

IR (Diamond-ATR, neat): $\tilde{\nu}$ / cm^{-1} = 3257, 2983, 2939, 2879, 2123, 1874, 1593, 1576, 1498, 1459, 1396, 1376, 1348, 1313, 1301, 1250, 1226, 1208, 1184, 1138, 1049, 1034, 1016, 957, 930, 899, 887, 871, 839, 764, 739, 725, 689, 641, 623, 606.

HRMS (ESI) calcd. for $C_{24}H_{35}N_4O$ $[M-BF_4]^+$: 395.2805, found 395.2803.

(E)-1,2-Bis(N,N-diisopropylamino)-3-((2-sulfophenyl)diazenyl) cyclopropenium (3d)

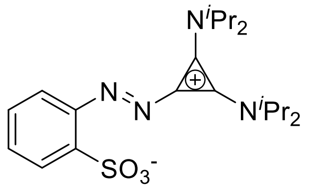

3d

3d was prepared according to **GP1.2** using 2-diazoniumbenzenesulfonate (55 mg, 0.30 mmol, 1.0 equiv) and *N*,*N*-bis(diisopropylamino)cyclopropenylidene (**2**) (1.0 mL, 0.3 M in THF:hexanes = 4:1, 0.30 mmol, 1.0 equiv, **GP1.1**) in dry MeCN (2 mL, 0.1 M final concentration). Purification by flash column chromatography (SiO_2, EtOAc, then CH_2Cl_2:MeOH = 1:0 to 95:5, R_f = 0.25 in CH_2Cl_2:MeOH = 95:5) and precipitation from $CHCl_3$/Et_2O afforded **3d** (49 mg, 0.12 mmol, 39%) as red solid.

^{1}H-NMR (400 MHz, CD_3CN): δ / ppm = 8.13 – 8.05 (m, 1H), 7.61 – 7.53 (m, 1H), 7.50 – 7.43 (m, 2H), 4.40 (sept., J = 7.0 Hz, 2H), 4.19 (sept., J = 7.0 Hz, 2H), 1.47 (d, J = 7.0 Hz, 12H), 1.40 (d, J = 7.0 Hz, 12H).

^{13}C-NMR (101 MHz, CD_3CN): δ / ppm = 151.0, 148.3, 134.0, 130.6, 129.8, 128.7, 115.3, 111.8, 55.8, 53.5, 21.2, 21.2.

IR (Diamond-ATR, neat): $\tilde{\nu}$ / cm^{-1} = 2981, 2937, 2878, 1869, 1636, 1580, 1457, 1376, 1351, 1228, 1204, 1160, 1138, 1085, 1072, 1035, 1020, 949, 898, 888, 833, 771, 738, 714, 686, 648, 636, 612.

HRMS (ESI) calcd. for $C_{21}H_{33}N_4O_3S$ $[M+H]^+$: 421.2268, found 421.2268.

Preparation of *N*-Boc-*N*-Arylhydrazides

General Procedure 2.1: Preparation of *N*-Boc-*N*-Arylhydrazides (**4**)

This procedure is based on a report by Buchwald and co-workers.[24]

A flame-dried flask equipped with a magnetic stir bar was charged with aryl iodide, Cs_2CO_3, *tert*-butyl carbazate, and 1,10-phenanthroline. The atmosphere was exchanged for N_2 and dry DMF (1M) and CuI were added in the N_2 counter current. The reaction was capped with a stopper and heated at 80 °C. After consumption of starting material as indicated by TLC analysis, the crude reaction mixture was diluted with EtOAc and filtered over a short plug of silica gel eluting with EtOAc or CH_2Cl_2/MeOH. The solvent was removed under reduced pressure and the crude product purified by flash column chromatography.

The following compounds were prepared according to Buchwald's report and physical data were in good accordance with those reported.[24]

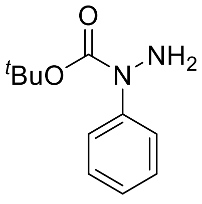

SI-4a

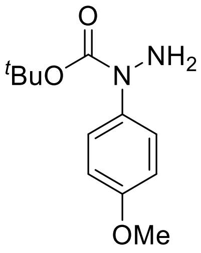

SI-4c

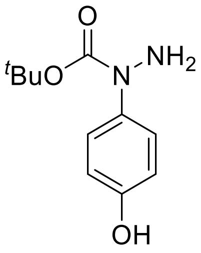

SI-4d

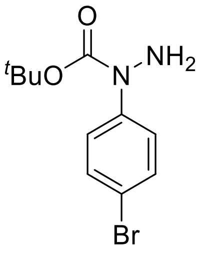

SI-4f

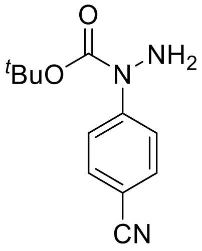

SI-4j

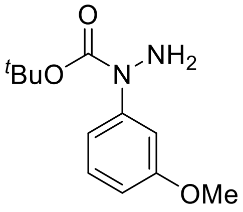

SI-4k

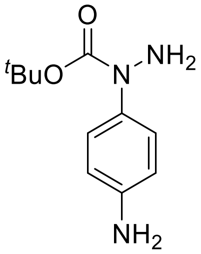

SI-4v

N-Boc-*N*-(4-morpholinophenyl)hydrazide (SI-4b)

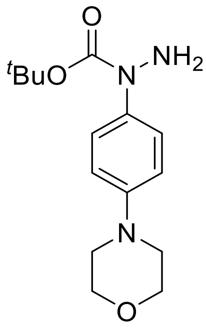

SI-4b

SI-4b was prepared according to **GP 2.1** using 4-(4-iodophenyl)morpholine (0.72 g, 2.5 mmol, 1.0 equiv), Cs_2CO_3 (1.1 g, 3.4 mmol, 1.4 equiv), *tert*-butyl carbazate (0.40 g, 3.0 mmol, 1.2 equiv), 1,10-phenanthroline (45 mg, 0.25 mmol, 0.10 equiv), and CuI (4.8 mg, 0.03 mmol, 1.0 Mol%) in dry DMF (2.5 mL, 1 M). The reaction was heated at 80 °C for 21 h. The crude product was filtered over silica, eluting with EtOAc, and purified by flash column chromatography (SiO_2, hexane:EtOAc = 1:0 to 1:1, R_f = 0.20 in hexane:EtOAc = 6:4) to obtain **SI-4b** (0.67 g, 2.28 mmol, 92%) as pale-yellow solid.

^{1}H-NMR (400 MHz, $CDCl_3$): δ / ppm = 7.33 – 7.27 (m, 2H), 6.89 – 6.81 (m, 2H), 4.42 (s, 2H), 3.90 – 3.81 (m, 4H), 3.16 – 3.07 (m, 4H), 1.48 (s, 9H).

^{13}C-NMR (101 MHz, $CDCl_3$): δ / ppm = 155.6, 148.7, 136.0, 124.9, 115.6, 81.5, 67.0, 49.8, 28.5.

IR (Diamond-ATR, neat): $\tilde{\nu}$ / cm^{-1} = 3336, 3220, 2973, 2930, 2856, 2822, 1692, 1634, 1609, 1514, 1476, 1451, 1427, 1391, 1367, 1353, 1334, 1303, 1258, 1232, 1171, 1150, 1121, 1055, 1032, 1008, 929, 852, 827, 762, 662, 615, 579, 523.

HRMS (ESI) calcd. for $C_{15}H_{24}N_3O_3$ $[M+H]^+$: 294.1812, found 294.1806.

N-Boc-*N*-(4-chlorophenyl)hydrazide (SI-4e)[25]

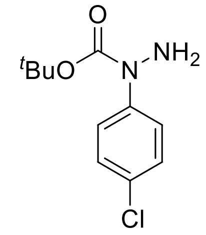

SI-4e

SI-4e was prepared according to **GP 2.1** using 1-chloro-4-iodobenzene (0.60 g, 2.5 mmol, 1.0 equiv), Cs_2CO_3 (1.1 g, 3.4 mmol, 1.3 equiv), *tert*-butyl carbazate (0.40 g, 3.0 mmol, 1.2 equiv), 1,10-phenanthroline (90 mg, 0.50 mmol, 0.20 equiv), and CuI (24 mg, 0.13 mmol, 5.0 Mol%) in dry DMF (2.5 mL, 1 M). The reaction was heated at 80 °C for 21 h. The crude product was filtered over silica, eluting with EtOAc, and purified by flash column chromatography (SiO_2, hexane:EtOAc = 9:1, R_f = 0.25) to obtain **SI-4e** (0.40 g, 2.0 mmol, 80%) as pale-yellow solid.

^{1}H-NMR (400 MHz, $CDCl_3$): δ / ppm = 7.48 – 7.38 (m, 2H), 7.27 – 7.22 (m, 2H), 4.27 (s, br, 2H), 1.50 (s, 9H).

^{13}C-NMR (101 MHz, $CDCl_3$): δ / ppm = 155.0, 141.8, 129.7, 128.2, 124.5, 82.3, 28.4.

IR (Diamond-ATR, neat): $\tilde{\nu}$ / cm^{-1} = 3343, 3225, 2978, 2932, 1891, 1692, 1629, 1593, 1489, 1456, 1411, 1393, 1368, 1327, 1286, 1253, 1149, 1093, 1049, 1029, 1011, 937, 849, 828, 762, 722, 710, 685, 633, 618, 570, 522, 504.

HRMS (ESI) calcd. for $C_{11}H_{15}ClN_2NaO_2$ $[M+Na]^+$: 265.0714, found 265.0709.

N-Boc-*N*-(4-(trifluoromethyl)phenyl)hydrazide (SI-4g)[26]

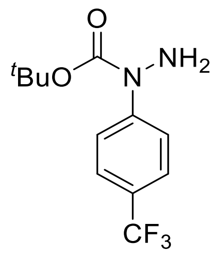

SI-4g

SI-4g was prepared according to **GP 2.1** using 1-iodo-4-(trifluoromethyl)benzene (0.68 g, 2.5 mmol, 1.0 equiv), Cs_2CO_3 (1.14 g, 3.4 mmol, 1.4 equiv), *tert*-butyl carbazate (400 mg, 3.0 mmol, 1.2 equiv), 1,10-phenanthroline (90 mg, 0.50 mmol, 0.20 equiv), and CuI (24 mg, 0.13 mmol, 5.0 Mol%) in dry DMF (2.5 mL, 1 M). The reaction was heated at 80 °C for 15 h. The crude product was filtered over silica, eluting with EtOAc, and purified by flash column chromatography (SiO_2, hexane:EtOAc = 9:1, R_f = 0.30) to obtain **SI-4g** (280 mg, 1.0 mmol, 41%) as colorless solid.

^{1}H-NMR (400 MHz, CDCl$_3$): δ / ppm = 7.72 – 7.64 (m, 2H), 7.57 – 7.51 (m, 2H), 4.42 (s, 2H), 1.53 (s, 9H).

^{13}C-NMR (101 MHz, CDCl$_3$): δ / ppm = 154.7, 146.2 (q, *J* = 1 Hz), 125.9 (q, *J* = 32 Hz), 125.4 (q, *J* = 4 Hz), 124.4 (q, *J* = 272 Hz), 122.4, 82.9, 28.4.

^{19}F-NMR (377 MHz, CDCl$_3$): δ / ppm = -62.

IR (Diamond-ATR, neat): $\tilde{\nu}$ / cm^{-1} = 3347, 2981, 2935, 1698, 1615, 1516, 1478, 1458, 1422, 1395, 1370, 1321, 1303, 1285, 1254, 1153, 1109, 1069, 1048, 1029, 1014, 935, 841, 764, 734, 686, 669, 636, 610, 555, 508.

HRMS (ESI) calcd. for $C_8H_8F_3N_2O_2$ [M+H]$^+$: 221.0532, found 221.0531.

N-Boc-*N*-(4-(methoxycarbonyl)phenyl)hydrazide (SI-4h)[27]

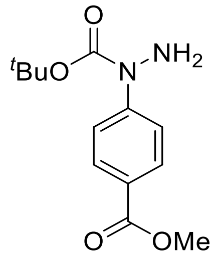

SI-4h

SI-4h was prepared according to **GP 2.1** using methyl 4-iodobenzoate (0.66 g, 2.5 mmol, 1.0 equiv), Cs_2CO_3 (1.1 g, 3.5 mmol, 1.4 equiv), *tert*-butyl carbazate (0.40 g, 3.0 mmol, 1.2 equiv), 1,10-phenanthroline (90 mg, 0.50 mmol, 0.20 equiv), and CuI (24 mg, 0.13 mmol, 5.0 Mol%) in dry DMF (2.5 mL, 1 M). The reaction was heated at 80 °C for 21 h. The product was filtered over silica, eluting with EtOAc, and purified by flash column chromatography (SiO_2, hexane:EtOAc, R_f = 0.25 in hexane:EtOAc = 8:2) to obtain **SI-4h** (0.44 g, 1.1 mmol, 67%) as colorless solid.

^{1}H-NMR (500 MHz, $CDCl_3$): δ / ppm = 7.99 – 7.94 (m, 2H), 7.67 – 7.59 (m, 2H), 4.41 (s, br, 2H), 3.89 (s, 3H), 1.53 (s, 9H).

^{13}C-NMR (126 MHz, $CDCl_3$): δ / ppm = 166.9, 154.7, 147.3, 129.9, 125.5, 121.9, 82.8, 52.1, 28.4.

IR (Diamond-ATR, neat): $\tilde{\nu}$ / cm^{-1} = 3348, 2979, 2952, 1701, 1629, 1604, 1575, 1508, 1477, 1457, 1435, 1421, 1393, 1369, 1328, 1272, 1254, 1163, 1112, 1050, 1030, 1015, 965, 942856, 810, 772, 699, 635, 505.

HRMS (ESI) calcd. for $C_{13}H_{18}N_2NaO_4$ $[M+Na]^+$: 289.1159, found 289.1155.

N-Boc-*N*-(4-(tert-butoxycarbonyl)phenyl)hydrazide (SI-4i)

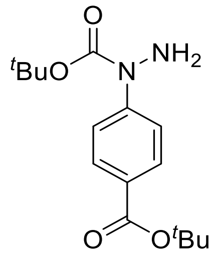

SI-4i

SI-4i was prepared according to **GP 2.1** using tert-butyl 4-iodobenzoate (0.76 g, 2.5 mmol, 1.0 equiv), Cs_2CO_3 (1.1 g, 3.4 mmol, 1.4 equiv), *tert*-butyl carbazate (0.40 g, 3.0 mmol, 1.2 equiv), 1,10-phenanthroline (90 mg, 0.50 mmol, 0.20 equiv), and CuI (24 mg, 0.13 mmol, 5.0 Mol%) in dry DMF (2.5 mL, 1 M). The reaction was heated at 80 °C for 21 h. The product was filtered over silica, eluting with EtOAc, and purified by flash column chromatography (SiO_2, hexane:EtOAc = 1:0 to 7:3, R_f = 0.20 in hexane:EtOAc = 9:1) to obtain **SI-4i** (0.61 g, 2.0 mmol, 79%) as colorless solid.

^{1}H-NMR (500 MHz, $CDCl_3$): δ / ppm = 7.93 – 7.89 (m, 2H), 7.61 – 7.56 (m, 2H), 4.42 (s, 2H), 1.58 (s, 9H), 1.52 (s, 9H).

^{13}C-NMR (126 MHz, $CDCl_3$): δ / ppm = 165.6, 154.8, 146.8, 129.7, 127.5, 121.9, 82.7, 80.9, 28.4, 28.4.

IR (Diamond-ATR, neat): $\tilde{\nu}$ / cm^{-1} = 3348, 3228, 2977, 2933, 1700, 1603, 1506, 1477, 1457, 1420, 1393, 1368, 1329, 1309, 1284, 1253, 1158, 1116, 1050, 1030, 1015, 940, 853, 817, 774, 746, 700, 674, 635, 614, 571, 522.

HRMS (ESI) calcd. for $C_{16}H_{24}N_2NaO_4$ $[M+Na]^+$: 331.1628, found 331.1628.

N-Boc-*N*-(3-methoxycarbonylphenyl)hydrazide (SI-4l)[27]

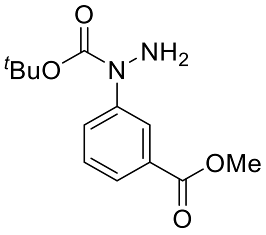

SI-4l

SI-4l was prepared according to **GP 2.1** using methyl 3-iodobenzoate (0.66 g, 2.5 mmol, 1.0 equiv), Cs_2CO_3 (1.1 g, 3.5 mmol, 1.4 equiv), *tert*-butyl carbazate (0.40 g, 3.0 mmol, 1.2 equiv), 1,10-phenanthroline (90 mg, 0.50 mmol, 0.20 equiv), and CuI (24 mg, 0.13 mmol, 5.0 Mol%) in dry DMF (2.5 mL, 1 M). The reaction was heated at 80 °C for 21 h. The product was filtered over silica, eluting with EtOAc, and purified by flash column chromatography (SiO_2, hexane:EtOAc = 1:0 to 81:19, R_f = 0.25 in hexane:EtOAc = 8:2) to obtain **SI-4l** (0.29 g, 1.1 mmol, 44%) as colorless solid.

^{1}H-NMR (500 MHz, $CDCl_3$): δ / ppm = 8.18 (t, *J* = 2.0 Hz, 1H), 7.77 (ddd, *J* = 7.5, 2.0, 1.0 Hz, 1H), 7.73 – 7.67 (m, 1H), 7.37 (td, *J* = 8.0, 7.5, 0.5 Hz, 1H), 4.45 (s, br, 2H), 3.91 (s, 3H), 1.52 (s, 9H).

^{13}C-NMR (126 MHz, $CDCl_3$): δ / ppm = 167.0, 155.1, 143.5, 130.4, 128.3, 127.6, 125.6, 124.3, 82.5, 52.3, 28.4.

IR (Diamond-ATR, neat): $\tilde{\nu}$ / cm^{-1} = 3346, 2978, 2953, 1697, 1626, 1605, 1586, 1487, 1448, 1393, 1368, 1318, 1283, 1247, 1149, 1111, 1082, 1058, 1034, 982, 946, 906, 857, 810, 755, 711, 688, 654, 599, 503.

HRMS (ESI) calcd. for $C_{13}H_{18}N_2NaO_4$ $[M+Na]^+$: 289.1159, found 289.1150.

di-*tert*-Butyl 1,1'-(1,4-phenylene)bis(hydrazine-1-carboxylate) (SI-4m)[28]

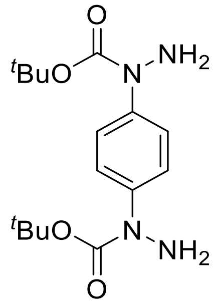

SI-4m

SI-4m was prepared according to **GP 2.1** using 1,4-diiodobenzene (0.41 g, 1.2 mmol, 1.0 equiv), Cs_2CO_3 (1.1 g, 3.4 mmol, 2.7 equiv), *tert*-butyl carbazate (0.40 g, 3.0 mmol, 2.4 equiv), 1,10-phenanthroline (90 mg, 0.50 mmol, 0.40 equiv), and CuI (24 mg, 0.13 mmol, 10 Mol%) in dry DMF (2.5 mL, 0.5 M). The reaction was heated at 80 °C for 21 h. The product was filtered over silica, eluting with EtOAc, and purified by flash column chromatography (SiO_2, hexane:EtOAc = 1:0 to 55:45, R_f = 0.20 in hexane:EtOAc = 6:4) to obtain **SI-4m** (0.34 g, 1.0 mmol, 81%) as pale-yellow solid.

^{1}H-NMR (500 MHz, $CDCl_3$): δ / ppm = 7.38 (s, 4H), 4.42 (s, 4H), 1.49 (s, 18H).

^{13}C-NMR (126 MHz, $CDCl_3$): δ / ppm = 155.3, 139.7, 123.2, 81.9, 28.5.

IR (Diamond-ATR, neat): $\tilde{\nu}$ / cm^{-1} = 3339, 3223, 2977, 2932, 1684, 1629, 1506, 1477, 1456, 1428, 1392, 1367, 1327, 1289, 1253, 1167, 1150, 1056, 1031, 1014, 942, 839, 763, 733, 676, 639, 616, 594, 574, 504.

HRMS (ESI) calcd. for $C_{16}H_{26}N_4NaO_4$ $[M+Na]^+$: 361.1846, found 361.1845.

N-Boc-*N*-(thiophen-3-yl)hydrazide (SI-4n)[27]

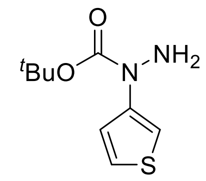

SI-4n

SI-4n was prepared according to **GP 2.1** using 3-iodothiophene (0.42 g, 2.0 mmol, 1.0 equiv), Cs_2CO_3 (0.91 g, 2.8 mmol, 1.4 equiv), *tert*-butyl carbazate (0.32 g, 2.4 mmol, 1.2 equiv), 1,10-phenanthroline (72 mg, 0.40 mmol, 0.20 equiv), and CuI (19 mg, 0.1 mmol, 5.0 Mol%) in dry DMF (2 mL, 1 M). The reaction was heated at 80 °C for 14 h. The product was filtered over silica, eluting with EtOAc, and purified by flash column chromatography (SiO_2, hexane:EtOAc = 1:0 to 5:1, R_f = 0.20 in hexane:EtOAc = 95:5) to obtain **SI-4n** (292 mg, 1.0 mmol, 68%) as yellow solid.

^{1}H-NMR (500 MHz, $CDCl_3$): δ / ppm = 7.38. – 7.30 (m, 1H), 7.19 – 7.11 (m, 2H), 4.43 (s, 2H), 1.55 (s, 9H).

^{13}C-NMR (126 MHz, $CDCl_3$): δ / ppm = 154.3, 141.9, 123.7, 123.0, 109.8, 82.3, 28.4.

IR (Diamond-ATR, neat): $\tilde{\nu}$ / cm^{-1} = 3345, 3228, 3131, 2977, 2932, 1686, 1625, 1536, 1476, 1454, 1417, 1394, 1367, 1320, 1253, 1217, 1205, 1163, 1146, 1080, 1051, 1033, 957, 900, 866, 849, 835, 769, 701, 673, 624.

HRMS (ESI) calcd. for $C_9H_{14}N_2NaO_2S$ $[M+Na]^+$: 237.0668, found 237.0666.

N-Boc-*N*-(1-methyl-1*H*-pyrazol-4-yl)hydrazide (SI-4o)

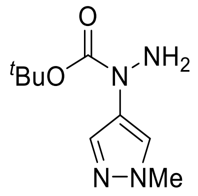

SI-4o

SI-4o was prepared according to **GP 2.1** using 4-iodo-1-methyl-1*H*-pyrazole (0.42 g, 2.0 mmol, 1.0 equiv), Cs_2CO_3 (0.91 g, 2.8 mmol, 1.4 equiv), *tert*-butyl carbazate (0.32 g, 2.4 mmol, 1.2 equiv), 1,10-phenanthroline (72 mg, 0.40 mmol, 0.20 equiv), and CuI (19 mg, 0.1 mmol, 5.0 Mol%) in dry DMF (2 mL, 1 M). The reaction was heated at 80 °C for 5.5 h. The product was filtered over silica, eluting with EtOAc, and purified by flash column chromatography (SiO_2, hexane:EtOAc = 1:1 to 1:4, R_f = 0.20 in hexane:EtOAc = 1:1) to obtain **SI-4o** (210 mg, 1.0 mmol, 49%) as orange solid.

^{1}H-NMR (500 MHz, $CDCl_3$): δ / ppm = 7.72 – 7.16 (m, 2H), 7.34 (s, br, 1H), 4.34 (s, 2H), 3.80 (s, 3H), 1.52 (s, 9H).

^{13}C-NMR (126 MHz, $CDCl_3$): δ / ppm = 154.4, 130.8, 128.2, 120.7, 82.3, 39.2, 28.4.

IR (Diamond-ATR, neat): $\tilde{\nu}$ / cm^{-1} = 3339, 2977, 2935, 1689, 1634, 1574, 1476, 1449, 1423, 1393, 1367, 1337, 1316, 1256, 1149, 1076, 984, 945, 835, 797, 759, 698, 676, 652, 621.

HRMS (ESI) calcd. for $C_9H_{16}N_4NaO_2$ $[M+Na]^+$: 235.1165, found 235.1167.

N-Boc-*N*-(1-methyl-1*H*-pyrazol-3-yl)hydrazide (SI-4s)

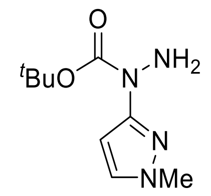

SI-4s

SI-4s was prepared according to **GP 2.1** using 3-iodo-1-methyl-1*H*-pyrazole (0.21 g, 1.0 mmol, 1.0 equiv), Cs_2CO_3 (0.46 g, 1.4 mmol, 1.4 equiv), *tert*-butyl carbazate (0.16 g, 1.2 mmol, 1.2 equiv), 1,10-phenanthroline (18 mg, 0.10 mmol, 0.10 equiv), and CuI (9.6 mg, 0.050 mmol, 5.0 Mol%) in dry DMF (1 mL, 1 M). The reaction was heated at 80 °C for 6 h. The product was filtered over silica, eluting with CH_2Cl_2:MeOH (4:1, [v/v], 50 mL), and purified by flash column chromatography (SiO_2, CH_2Cl_2:MeOH = 1:0 to 97:3, R_f = 0.30 in CH_2Cl_2:MeOH = 97:3) to obtain **SI-4s** (124 mg, 0.58 mmol, 58%) as colorless solid.

^{1}H-NMR (500 MHz, $CDCl_3$): δ / ppm = 7.21 (d, 1H), 6.25 (s, br, 1H), 3.83 (s, 3H), 3.48 (s, br, 2H), 1.53 (s, 9H).

^{13}C-NMR (126 MHz, $CDCl_3$): δ / ppm = 154.2, 151.0, 130.7, 98.6, 82.2, 39.1, 28.5.

IR (Diamond-ATR, neat): $\tilde{\nu}$ / cm^{-1} = 3335, 3217, 3121, 2978, 2933, 1699, 1630, 1533, 1481, 1456, 1433, 1392, 1368, 1322, 1253, 1159, 1110, 1075, 1032, 1000, 956, 856, 799, 759, 692, 675, 621.

HRMS (ESI) calcd. for $C_9H_{16}N_4NaO_2$ $[M+Na]^+$: 235.1165, found 235.1171.

N-Boc-*N*-(1-methyl-1*H*-indazol-3-yl)hydrazide (SI-4t)

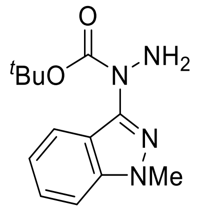

SI-4t

SI-4t was prepared according to **GP 2.1** using 3-iodo-1-methyl-1*H*-indazole (0.26 g, 1.0 mmol, 1.0 equiv), Cs_2CO_3 (0.46 mg, 1.4 mmol, 1.4 equiv), *tert*-butyl carbazate (0.16 g, 1.2 mmol, 1.2 equiv), 1,10-phenanthroline (36 mg, 0.20 mmol, 0.20 equiv), and CuI (9.5 mg, 0.05 mmol, 5.0 Mol%) in dry DMF (1 mL, 1 M). The reaction was heated at 80 °C for 21 h. The product was filtered over silica, eluting with EtOAc, and purified by flash column chromatography (SiO_2, hexane:EtOAc = 1:0 to 1:1, R_f = 0.20 in hexane:EtOAc = 1:1) to obtain **SI-4t** (67 mg, 0.26 mmol, 25%) as colorless oil.

^{1}H-NMR (500 MHz, $CDCl_3$): δ / ppm = 7.62 (dt, *J* = 8.0, 1.0 Hz, 1H), 7.38 (ddd, *J* = 8.5, 6.5, 1.0 Hz, 1H), 7.33 (dt, *J* = 8.5, 1.0 Hz, 1H), 7.14 (ddd, *J* = 8.0, 6.5, 1.0 Hz, 1H), 4.67 (s, 2H), 4.03 (s, 3H), 1.44 (s, 9H).

^{13}C-NMR (126 MHz, $CDCl_3$): δ / ppm = 155.6, 143.4, 141.0, 126.7, 121.0, 120.5, 118.0, 109.2, 82.2, 77.4, 76.9, 35.7, 28.3.

IR (Diamond-ATR, neat): $\tilde{\nu}$ / cm^{-1} = 3337, 3219, 3059, 2978, 2933, 1704, 1620, 1578, 1501, 1477, 1450, 1429, 1393, 1368, 1332, 1252, 1213, 1164, 1144, 1096, 1043, 1004, 925, 860, 813, 770, 745, 683, 608.

HRMS (ESI) calcd. for $C_{13}H_{18}N_4NaO_2$ $[M+Na]^+$: 285.1322, found 285.1316.

N-Boc-*N*-(1-benzyl-1*H*-1,2,3-triazole-4-yl)hydrazide (SI-4u-3)

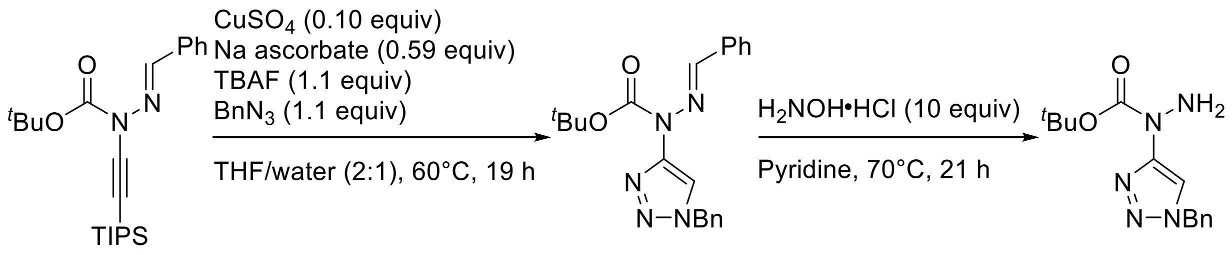

SI-4u-1 **SI-4u-2** **SI-4u-3**

tert-Butyl-2-benzylidene-1-((triisopropylsilyl)ethynyl)hydrazine-1-carboxylate (**SI-4u-1**) was prepared according to a known literature procedure.[29] **SI-4u-2** and **SI-4u-3** were prepared according to modified procedures originating from the same reference.

tert-Butyl 1-(1-benzyl-1*H*-1,2,3-triazol-4-yl)-2-benzylidenehydrazine-1-carboxylate (**SI-4u-2**):

A 25 mL flask equipped with a magnetic stir bar was charged with $CuSO_4$ (20 mg, 0.12 mmol, 0.10 equiv) and sodium ascorbate (0.15 g, 0.74 mmol, 0.59 equiv) in water (1.5 mL) under N_2 atmosphere. TBAF (1.4 mL, 1 M in THF, 1.4 mmol, 1.1 equiv) was added, followed by a solution of **SI-4u-1** (0.50 g, 1.2 mmol, 1.0 equiv) and (azidomethyl)benzene (0.18 g, 0.17 mL, 1.4 mmol, 1.1 equiv) in THF (3.0 mL). The resulting mixture was heated at 60 °C for 19 h. After cooling to ambient temperature, the reaction was diluted with CH_2Cl_2 (20 mL) and water (20 mL), the phases were separated, and the aq. layer extracted with CH_2Cl_2 (2x10 mL). The combined org. layers were dried over Na_2SO_4 and concentrated under reduced pressure. Purification by flash column chromatography (SiO_2, hexane:EtOAc = 1:0 to 4:1) afforded **SI-4u-2** as colorless solid (0.36 g, 0.96 mmol, 78%).

^{1}H-NMR (500 MHz, $CDCl_3$): δ / ppm = 7.64 – 7.59 (m, 2H), 7.52 (s, 1H), 7.50 (s, 1H), 7.40 – 7.34 (m, 3H), 7.33 – 7.29 (m, 3H), 7.28 – 7.25 (m, 2H), 5.59 (s, 2H), 1.45 (s, 9H).

^{13}C-NMR (126 MHz, $CDCl_3$): δ / ppm = 152.1, 144.7, 141.4, 134.3, 134.1, 130.0, 129.3, 129.0, 128.6, 127.9, 127.6, 121.7, 82.7, 76.9, 54.9, 28.1.

IR (Diamond-ATR, neat): $\tilde{\nu}$ / cm^{-1} = 3119, 3068, 3031, 2980, 2929, 1731, 1605, 1545, 1497, 1454, 1439, 1382, 1373, 1366, 1350, 1319, 1302, 1289, 1255, 1229, 1203, 1155, 1143, 1124, 1081, 1037, 986, 941, 913, 874, 856, 842, 813, 770, 761.

HRMS (ESI) calcd. for $C_{21}H_{23}N_5NaO_2$ $[M+Na]^+$: 400.1744, found 400.1737.

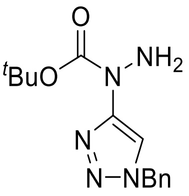

SI-4u-3

N-Boc-*N*-(1-benzyl-1*H*-1,2,3-triazole-4-yl)hydrazide **SI-4u-3**:

A 10 mL flask equipped with a magnetic stir bar was charged with **SI-4u-2** (300 mg, 0.79 mmol, 1.0 equiv) and hydroxylamine hydrochloride (550 mg, 7.9 mmol, 10 equiv). The atmosphere was exchanged for N_2, dry pyridine (1.6 mL) was added, and the reaction heated at 70 °C for 23 h. After cooling to ambient temperature, the crude was concentrated and partitioned between EtOAc (20 mL) and sat. $NaHCO_3$-sln. (20 mL). The phases were separated, and the aq. phase extracted with EtOAc (20 mL). The combined organic layers were dried over Na_2SO_4 and concentrated under reduced pressure. The crude product was purified by flash column chromatography (SiO_2, hexane:EtOAc = 1:1 to 3:7, R_f = 0.30 in hexane:EtOAc = 3:7) afforded **SI-4u-3** as colorless solid (150 mg, 0.53 mmol, 67%).

^{1}H-NMR (500 MHz, $CDCl_3$): δ / ppm = 7.55 (s, br, 1H), 7.39 – 7.31 (m, 3H), 7.31 – 7.26 (m, 2H), 5.47 (s, 2H), 4.51 (s, br, 2H), 1.50 (s, 9H).

^{13}C-NMR (126 MHz, $CDCl_3$): δ / ppm = 153.4, 148.2, 134.6, 129.2, 128.9, 128.2, 113.6, 82.7, 54.8, 28.4.

IR (Diamond-ATR, neat): $\tilde{\nu}$ / cm^{-1} = 3344, 3238, 3169, 2981, 2931, 1685, 1644, 1586, 1558, 1497, 1476, 1453, 1442, 1391, 1367, 1358, 1346, 1326, 1308, 1252, 1223, 1211, 1167, 1152, 1136, 1065, 1051, 1029, 977, 917, 853, 824, 781, 765, 734, 712, 698, 669, 621.

HRMS (ESI) calcd. for $C_{14}H_{20}N_5O_2$ $[M+H]^+$: 290.1612, found 290.1607.

Preparation of Aryl Azocyclopropenium Salts from *N*-Boc-*N*-Arylhydrazides

Reaction Optimization

Condensation: A 4 mL glass vial equipped with a magnetic stir bar was charged with chloro-bis(dimethylamino)cyclopropenium tetrafluoroborate (**1b**) (40 mg, 0.16 mmol, 1.3 equiv). Stock solutions of *N*-Boc-*N*-phenylhydrazide (130 µL, 1.0 M, 0.13 mmol, 1.0 equiv) and the respective base (0.40 mL, 0.27 M, 1.5 mmol, 1.2 equiv) in $CDCl_3$ were added and the reaction stirred at 50 °C for 30 min. TFA (0.020 mL, 0.25 mmoL, 2.0 equiv) and mesitylene (0.010 mL) were added and the mixture transferred to an NMR tube for analysis by ^{1}H NMR spectroscopy (Figure S1). The content of the condensation product was determined from the relative integral intensity of the multiplet at 7.21 – 7.12 ppm and signals of mesitylene.

Table T1: Reaction Conditions Condensation

Base/Activator	Equiv	NMR Yield (condensation product)
Pyridine	1.2	93%
Triethylamine	1.2	42%
DMAP	1.2	49%
no external base	-	49%

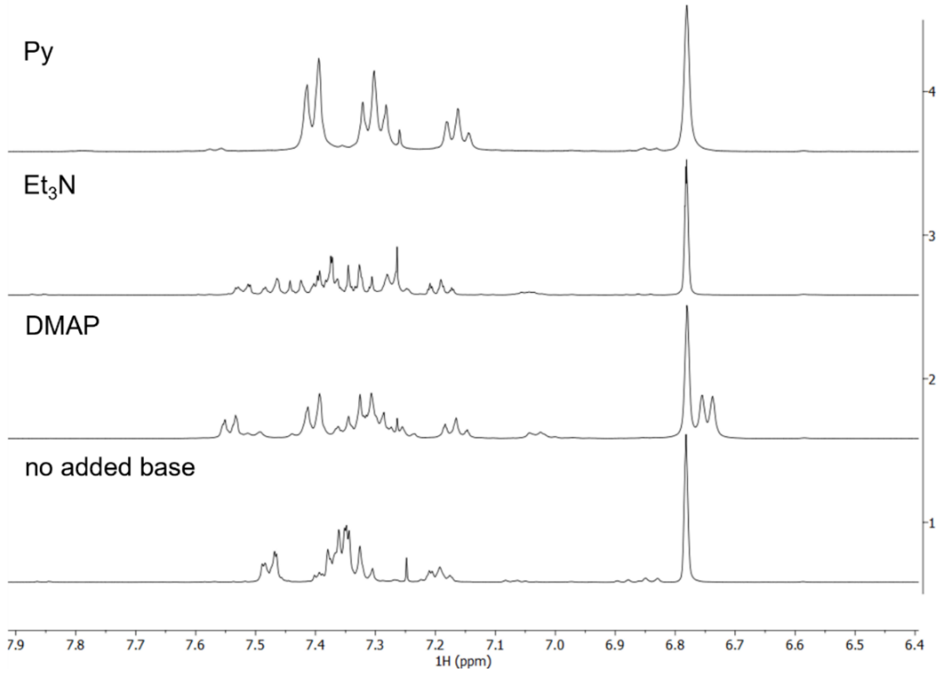

Figure S1: ^{1}H NMR spectra of the unpurified reaction mixtures in $CDCl_3$ with [4] pyridine, [3] NEt_3, or [2] DMAP as basic activator, or [1] no additive.

One-pot Condensation and Oxidation: A 4 mL glass vial equipped with a magnetic stir bar was charged with chloro-bis(dimethylamino)cyclopropenium tetrafluoroborate (**1b**) (40 mg, 0.16 mmol, 1.3 equiv). Stock solutions of *N*-Boc-*N*-phenylhydrazide (130 µL, 1.0 M, 0.13 mmol, 1.0 equiv) and the respective base (0.40 mL, 0.27 M, 1.5 mmol, 1.2 equiv) in $CDCl_3$ were added and the reaction stirred at 50 °C for 30 min. The heating was removed, benzoquinone (27 mg, 0.15 mmol, 2.0 equiv) was added, and the reaction stirred for 1 min at ambient temperature. Then, TFA (0.5 mL) was added at once and stirring continued for 5 min. The reaction was concentrated under reduced pressure, the residue was dissolved in CD_3CN (0.7 mL), and mesitylene (0.010 mL) was added as internal standard for analysis by ^1H NMR spectroscopy (Figure S2). The yield of phenyl azocyclopropenium **6a** was determined from the relative integral intensity of the multiplet at 7.63 – 7.56 ppm and signals of mesitylene.

Table T2: Reaction Conditions One-pot Condensation and Oxidation

Base/Activator	Equiv	NMR Yield (aryl azocyclopropenium)
Pyridine	1.2	84%
Triethylamine	1.2	38%
DMAP	1.2	45%
no external base	-	46%

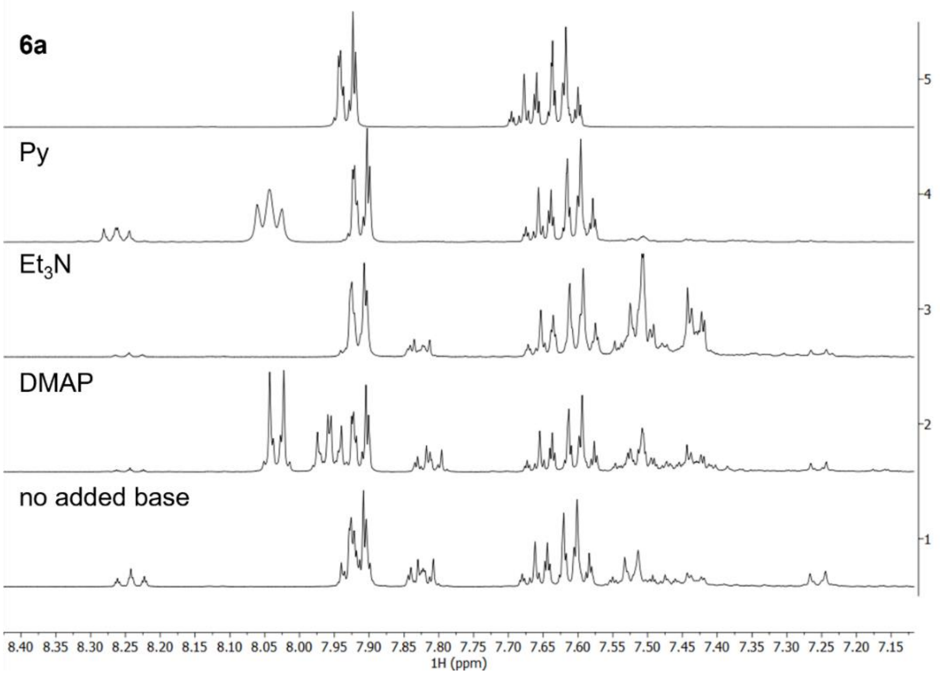

Figure S2: ^1H NMR spectra of [5] purified phenyl azocyclopropenium salt **6a** and unpurified reaction mixtures in CD_3CN with [4] pyridine, [3] NEt_3, or [2] DMAP as basic activator, or [1] no additive.

Chloro-bis(diisopropylamino)cyclopropenium tetrafluoroborate: A 4 mL glass vial equipped with a magnetic stir bar was charged with chloro-bis(diisopropylamino)cyclopropenium tetrafluoroborate (**1a**) (45 mg, 0.13 mmol, 1.0 equiv). Stock solutions of *N*-Boc-*N*-phenylhydrazide (130 µL, 1.0 M, 0.13 mmol, 1.0 equiv) in the respective solvent and the respective base (1.5 mmol, 1.2 equiv) were added and the reaction stirred at the indicated temperature for 20 h. The reaction was allowed to cool, benzoquinone (27 mg, 0.15 mmol, 2.0 equiv) was added, and the reaction stirred for 1 min at ambient temperature. Then, TFA (130 µL) was added at once and stirring continued for 5 min. The reaction concentrated under reduced pressure. The residue was dissolved in CD_3CN (0.7 mL), and mesitylene (0.010 mL) was added as internal standard for analysis by ^1H NMR spectroscopy. The yield of phenyl azocyclopropenium **3a** was determined from the relative integral intensity of the multiplet at 7.99 – 7.94 ppm and signals of mesitylene.

Table T3: Chloro-bis(diisopropylamino)cyclopropenium tetrafluoroborate

**Base/Activator**	**Equiv**	**solvent**	**T [°C]**	**NMR Yield (aryl azocyclopropenium)**
Pyridine	1.2	$CDCl_3$	60	35%
		1,2-DCE	80	60%
Triethylamine	1.2	1,2-DCE	80	49%

General Procedure 2.2: Synthesis of (*E*)-bis(*N,N*-diisopropylamino) aryldiazenyl cyclopropenium tetrafluoroborate salts

A 4 mL glass vial equipped with a magnetic stir bar was charged with chloro-bis(diisopropylamino)cyclopropenium tetrafluoroborate **1a** (1.0 equiv) and *N*-Boc-*N*-(hetero)arylhydrazide (1.0 equiv). Dry 1,2-DCE (1 M) was added, and the solution stirred for 1 min. Pyridine (1.2 equiv) was added and the reaction heated at 80 °C until TLC monitoring indicated complete consumption of *N*-Boc-*N*-(hetero)aryl hydrazide. The reaction was allowed to cool, benzoquinone (2.0 equiv) was added, and the reaction stirred for 1 min at ambient temperature. Then, TFA (0.5 mL) was added at once and stirring continued for 5 min. The reaction was concentrated under reduced pressure and the residue dissolved in $CHCl_3$/*i*-PrOH (3:1, [v/v]). The resulting solution was washed with HBF_4-sln. (5%, [w/w]), sat. $NaHCO_3$-sln., and $NaBF_4$-sln. (10%, [w/w]), dried over Na_2SO_4, and concentrated under reduced pressure. The crude was purified by flash column chromatography on silica and precipitation from $CHCl_3$ with Et_2O to afford the arylazocyclopropenium tetrafluoroborate salts in spectroscopical purity.

General Procedure 2.3: Synthesis of (*E*)-bis(*N,N*-dimethylamino) aryldiazenyl cyclopropenium tetrafluoroborate salts

A 4 mL glass vial equipped with a magnetic stir bar was charged with chloro-bis(dimethylamino)cyclopropenium tetrafluoroborate **1b** (1.3 equiv) and *N*-Boc-*N*-(hetero)aryl-hydrazide (1.0 equiv). Dry $CHCl_3$ (0.25 M) was added, and the solution stirred for 1 min. Pyridine (1.2 equiv) was added and the reaction stirred at 50 °C until TLC monitoring indicated complete consumption of the *N*-Boc-*N*-(hetero)aryl hydrazide. The reaction was allowed to cool, benzoquinone (2.0 equiv) was added, and the reaction stirred for 1 min at ambient temperature. Then, TFA (0.5 mL) was added at once and stirring continued for 5 to 10 min. The reaction was concentrated under reduced pressure and the residue dissolved in $CHCl_3$/*i*-PrOH (3:1, [v/v]). The resulting solution was washed with HBF_4-sln. (5%, [w/w]), dried over Na_2SO_4, and concentrated under reduced pressure. The crude was dissolved in MeCN and precipitated with Et_2O (ratio ca. 1:10). Further purification was performed either by repeated precipitation or flash column chromatography on silica to afford the arylazocyclopropenium tetrafluoroborate salts in spectroscopical purity.

(E)-1,2-Bis(N,N-diisopropylamino)-3-phenyldiazenylcyclopropenium tetrafluoroborate (3a)

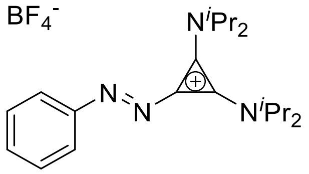

3a

3a was prepared according to **GP2.2** using 1-tert-butoxycarbonyl-1-phenylhydrazine (**SI-4a**) (52 mg, 0.25 mmol, 1.0 equiv), chloro-bis(diisopropylamino)cyclopropenium tetrafluoroborate (90 mg, 0.25 mmol, 1.0 equiv), and pyridine (24 uL, 0.3 mmol, 1.2 equiv) in dry DCE (0.3 mL, 1 M). The reaction was heated at 80 °C for 21 h. Benzoquinone (54 mg, 0.5 mmol, 2.0 equiv) was added and the reaction worked up as described in **GP2.2**. Purification by flash column chromatography (SiO_2, EtOAc, then CH_2Cl_2:MeOH = 1:0 to 95:5, R_f = 0.20 in CH_2Cl_2:MeOH = 98:2) and precipitation from $CHCl_3$/Et_2O afforded (61 mg, 0.14 mmol, 57%) as red solid.

The physical data of the obtained material were in accordance with the sample prepared according to **GP1.2**. NMR spectra of the isolated material are given for comparison.

(E)-1,2-Bis(N,N-diisopropylamino)-3-((4-bromophenyl)diazenyl) cyclopropenium tetrafluoroborate (3e)

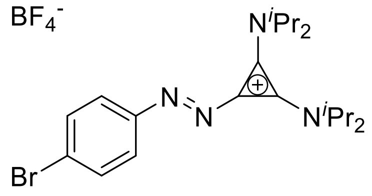

3e

3e was prepared according to **GP2.2** using tert-butyl 1-(4-bromophenyl)hydrazine-1-carboxylate (359 mg, 1.0 mmol, 1.0 equiv), chloro-bis(diisopropylamino)cyclopropenium tetrafluoroborate (287 mg, 1.0 mmol, 1.0 equiv), and pyridine (96 uL, 1.2 mmol, 1.2 equiv) in dry $CHCl_3$ (1 mL, 1 M). The reaction was heated at 80 °C for 21 h. Benzoquinone (216 mg, 2.0 mmol, 2.0 equiv) was added and the reaction worked up as described in **GP2.2**. Purification by flash column chromatography (SiO_2, EtOAc, then CH_2Cl_2:MeOH = 1:0 to 95:5, R_f = 0.35 in CH_2Cl_2:MeOH = 95:5) and precipitation from $CHCl_3$ (3 mL) and Et_2O (15 mL) afforded **3e** (289 mg, 0.57 mmol, 57%) as red solid.

^{1}H-NMR (500 MHz, CD_3CN): δ / ppm = 7.89 – 7.84 (m, 2H), 7.81 – 7.78 (m, 2H), 4.16 (sept., J = 6.5 Hz, 2H), 4.07 (sept., J = 7.0 Hz, 2H), 1.46 (d, J = 7.0 Hz, 12H), 1.41 (d, J = 6.5 Hz, 12H).

^{13}C-NMR (126 MHz, CD_3CN): δ / ppm = 152.9, 134.1, 129.6, 129.3, 125.8, 110.8, 59.4, 50.2, 21.5, 20.6.

^{19}F-NMR (471 MHz, CD_3CN): δ / ppm = -152.

IR (Diamond-ATR, neat): $\tilde{\nu}$ / cm^{-1} = 2983, 2940, 1871, 1581, 1459, 1415, 1394, 1376, 1349, 1311, 1301, 1283, 1207, 1183, 1156, 1139, 1090, 1047, 1033, 1003, 954, 898, 887, 871, 834, 764, 747, 709, 656, 623, 606, 573, 558, 539, 520, 492.

HRMS (ESI) calcd. for $C_{21}H_{32}BrN_4$ $[M-BF_4]^+$: 419.1805, found 419.1795.

(E)-1,2-Bis(N,N-diisopropylamino)-3-((4-carboxyphenyl)diazenyl) cyclopropenium tetrafluoroborate (3f)

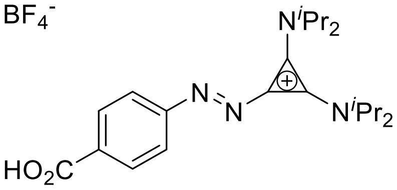

3f

3f was prepared according to **GP2.2** using tert-butyl 1-(4-(tert-butoxycarbonyl)phenyl)hydrazine-1-carboxylate (**SI-4-i**) (150 mg, 0.50 mmol, 1.0 equiv), chloro-bis(diisopropylamino)cyclopropenium tetrafluoroborate (**1a**) (180 mg, 5.0 mmol, 1.0 equiv), and pyridine (48 uL, 0.6 mmol, 1.2 equiv) in dry DCE (0.5 M, 1 mL). The reaction was heated at 80°C for 21 h. Benzoquinone (110 mg, 1.0 mmol, 2.0 equiv) was added and the reaction worked up as described in **GP2.3**. Precipitation from MeCN/$CHCl_3$ (1:4) using excess Et_2O afforded **3f** (156 mg, 0.33 mmol, 66%) as red solid.

^{1}H-NMR (500 MHz, CD_3CN): δ / ppm = 9.78 (s, br, 1H), 8.23 – 8.16 (m, 2H), 8.05 – 7.99 (m, 2H), 4.17 (sept., J = 7.0 Hz, 2H), 4.09 (sept., J = 6.5 Hz, 2H), 1.48 (d, J = 7.0 Hz, 12H), 1.42 (d, J = 6.5 Hz, 12H).

^{13}C-NMR (126 MHz, CD_3CN): δ / ppm = 166.7, 156.3, 135.3, 132.1, 129.5, 124.2, 110.7, 59.5, 50.3, 21.5, 20.6.

^{19}F-NMR (471 MHz, CD_3CN): δ / ppm = -152.

IR (Diamond-ATR, neat): $\tilde{\nu}$ / cm^{-1} = 3238, 2986, 2941, 2882, 1870, 1722, 1586, 1493, 1459, 1420, 1378, 1378, 1351, 1310, 1280, 1227, 1207, 1183, 1160, 1139, 1054, 1035, 1018, 955, 899, 868, 778, 739, 693, 653, 606, 559, 520, 497.

HRMS (ESI) calcd. for $C_{22}H_{33}N_4O_2$ $[M-BF_4]^+$: 385.2598, found 385.2593.

(*E*)-Bis(*N,N*-dimethylamino)phenyldiazenylcyclopropenium tetrafluoroborate (6a)

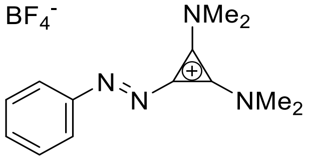

6a

6a was prepared according to **GP2.3** using chloro-bis(dimethylamino)cyclopropenium tetrafluoroborate (**1b**) (40 mg, 0.16 mmol, 1.3 equiv), *N*-Boc-*N*-phenylhydrazide (**SI-4a**) (26 mg, 0.12 mmol, 1.0 equiv), pyridine (12 µL, 0.15 mmol, 1.2 equiv), anhydrous $CHCl_3$ (0.5 mL), benzoquinone (27 mg, 0.25 mmol, 2.0 equiv), TFA (0.5 mL). Purification by flash column chromatography (SiO_2, EtOAc, then CH_2Cl_2:MeOH = 1:0 to 97:3, R_f = 0.25 in CH_2Cl_2:MeOH = 97:3) and precipitation from MeCN (1 mL) and Et_2O (10 mL) afforded **6a** (24 mg, 0.076 mmol, 61%) as orange solid.

^{1}H-NMR (500 MHz, CD_3CN): δ / ppm = 7.97 - 7.92 (m, 2H), 7.70 – 7.66 (m, 1H), 7.65 – 7.60 (m, 2H), 3.48 (s, 6H), 3.32 (s, 6H).

^{13}C-NMR (126z MHz, CD_3CN): δ / ppm = 154.2, 135.5, 131.7, 130.8, 124.5, 110.9, 44.9, 43.2.

^{19}F-NMR (471 MHz, CD_3CN): δ / ppm = -152.

IR (Diamond-ATR, neat): $\tilde{\nu}$ / cm^{-1} = 3096, 2947, 1897, 1630, 1580, 1498, 1452, 1421, 1413, 1309, 1286, 1226, 1201, 1168, 1152, 1094, 1048, 1036, 994, 955, 857, 788, 764, 694, 676, 612, 589, 581, 520.

HRMS (ESI) calcd. for $C_{13}H_{17}N_4$ $[M-BF_4]^+$: 229.1448, found 229.1450.

(E)-Bis(N,N-dimethylamino)-((4-(morpholin-4-yl)phenyl)diazenyl) cyclopropenium tetrafluoroborate (6b)

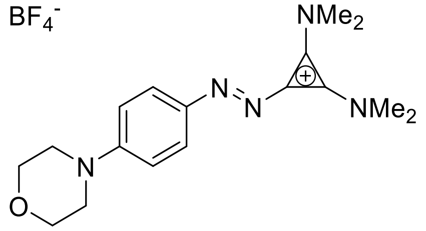

6b

6b was prepared according to **GP2.3** using *N*-Boc-*N*-(4-morpholinophenyl)hydrazide (**SI-4b**) (37 mg, 0.13 mmol, 1.0 equiv), chloro-bis(dimethylamino)cyclopropenium tetrafluoroborate (**1b**) (40 mg, 1.6 mmol, 1.3 equiv), and pyridine (12 µL, 1.5 mmol, 1.2 equiv) in dry $CHCl_3$ (0.5 mL, 1 M). The reaction was heated at 50 °C for 1 h. Benzoquinone (27 mg, 0.25 mmol, 2.0 equiv) was added and the reaction worked up as described in **GP2.3**. Flash column chromatography (SiO_2, EtOAc, then CH_2Cl_2:MeOH = 1:0 to 97:3, R_f = 0.20 in CH_2Cl_2:MeOH = 97:3), and precipitation from MeCN (1 mL) and Et_2O (10 mL) afforded (33 mg, 0.082 mmol, 65%) as dark purple solid.

^{1}H-NMR (500 MHz, CD_3CN): δ / ppm = 7.86 – 7.80 (m, 2H), 7.05 – 6.99 (m, 2H), 3.81 – 3.76 (m, 4H), 3.50 – 3.47 (m, 2H), 3.42 (s, 6H), 3.26 (s, 6H).

^{13}C-NMR (126 MHz, CD_3CN): δ / ppm = 156.6, 146.6, 130.2, 127.8, 114.5, 113.2, 67.0, 47.7, 44.4, 42.8.

^{19}F-NMR (471 MHz, CD_3CN): δ / ppm = -152.

IR (Diamond-ATR, neat): $\tilde{\nu}$ / cm^{-1} = 3625, 2941, 2857, 1913, 1618, 1592, 1546, 1515, 1449, 1408, 1329, 1298, 1269, 1217, 1151, 1108, 1032, 963, 925, 863, 835, 791, 775, 750, 732, 710, 671, 629, 610, 590, 538, 520.

HRMS (ESI) calcd. for $C_{17}H_{24}N_5O$ $[M-BF_4]^+$: 314.1975, found 314.1972.

(E)-Bis(N,N-dimethylamino)-((4-methoxyphenyl)diazenyl) cyclopropenium tetrafluoroborate (6c)

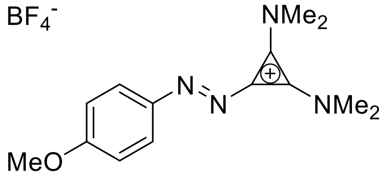

6c

6c was prepared according to **GP2.3** using *N*-Boc-*N*-(4-methoxyphenyl)hydrazide (**SI-4c**) (30 mg, 0.13 mmol, 1.0 equiv), chloro-bis(dimethylamino)cyclopropenium tetrafluoroborate (**1b**) (40 mg, 1.6 mmol, 1.3 equiv), and pyridine (12 µL, 1.5 mmol, 1.2 equiv) in dry $CHCl_3$ (0.5 mL, 1 M). The reaction was heated at 50 °C for 1 h. Benzoquinone (27 mg, 0.25 mmol, 2.0 equiv) was added and the reaction worked up as described in **GP2.3**. Purification by flash column chromatography (SiO_2, EtOAc, then CH_2Cl_2:MeOH = 1:0 to 97:3, R_f = 0.20 in CH_2Cl_2:MeOH = 97:3) and precipitation from MeCN (1 mL) and Et_2O (10 mL) afforded **6c** (27 mg, 0.078 mmol, 62%) as orange solid.

^{1}H-NMR (400 MHz, CD_3CN): δ / ppm = 7.99 - 7.89 (m, 2H), 7.16 - 7.09 (m, 2H), 3.92 (s, 3H), 3.45 (s, 6H), 3.30 (s, 6H).

^{13}C-NMR (101 MHz, CD_3CN): δ / ppm = 166.4, 148.9, 131.1, 127.2, 116.2, 111.6, 56.9, 44.7, 43.0.

^{19}F-NMR (377 MHz, CD_3CN): δ / ppm = -152.

IR (Diamond-ATR, neat): $\tilde{\nu}$ / cm^{-1} = 3616, 3101, 2949, 2008, 1907, 1690, 1629, 1598, 1574, 1499, 1452, 1435, 1425, 1409, 1364, 1329, 1314, 1302, 1252, 1210, 1184, 1148, 1098, 1054, 1040, 1016, 989, 902, 859, 802, 788, 738, 723, 670, 629, 590, 563, 523.

HRMS (ESI) calcd. for $C_{14}H_{19}N_4O$ $[M-BF_4]^+$: 259.1553, found 259.1555.

(E)-Bis(N,N-dimethylamino)-((4-hydroxyphenyl)diazenyl) cyclopropenium tetrafluoroborate (6d)

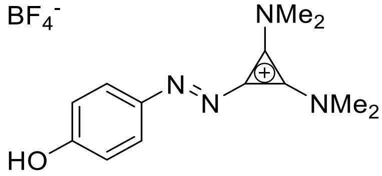

6d

6d was prepared according to **GP2.3** using *N*-Boc-*N*-(4-hydroxyphenyl)hydrazide (**SI-4d**) (28 mg, 0.13 mmol, 1.0 equiv), chloro-bis(dimethylamino)cyclopropenium tetrafluoroborate (**1b**) (40 mg, 1.6 mmol, 1.3 equiv), and pyridine (12 µL, 1.5 mmol, 1.2 equiv) in dry $CHCl_3$ (0.5 mL, 1 M). The reaction was heated at 50 °C for 1 h. Benzoquinone (27 mg, 0.25 mmol, 2.0 equiv) was added and the reaction stirred for 10 min before it was concentrated. The remaining solids were dissolved in $CHCl_3$/*i*-PrOH (4 mL, 3:1, [v/v]) and HBF_4 (2 mL, 5%, [w/w]) was added, resulting in precipitation of an orange solid. The suspension was filtered, and the solid residue washed with HBF_4 (0.5 mL, 5%, [w/w]) and $CHCl_3$/*i*-PrOH (0.5 mL, 3:1, [v/v]), and dissolved in MeCN. The layers of the filtrate were separated, the organic layer dried over Na_2SO_4, and combined with the solution of the previously collected solids in MeCN. Concentration of the resulting mixture and repeated precipitation (2x) of the product from MeCN (1 mL) and Et_2O (10 mL) afforded **6d** (26 mg, 0.078 mmol, 63%) as orange solid.

^{1}H-NMR (400 MHz, CD_3CN): δ / ppm = 8.18 (s, 1H), 7.88 – 7.80 (m, 2H), 7.06 – 6.96 (m, 2H), 3.43 (s, 6H), 3.29 (s, 6H).

^{13}C-NMR (101 MHz, CD_3CN): δ / ppm = 164.6, 148.5, 131.0, 127.6, 117.6, 111.8, 44.6, 43.0.

^{19}F-NMR (377 MHz, CD_3CN): δ / ppm = -152.

IR (Diamond-ATR, neat): $\tilde{\nu}$ / cm^{-1} = 3578, 3518, 3382, 3099, 2948, 1920, 1628, 1603, 1579, 1505, 1453, 1422, 1412, 1364, 1286, 1211, 1146, 1079, 1064, 1004, 962, 859, 789, 765, 730, 672, 635, 591, 550, 521.

HRMS (ESI) calcd. for $C_{13}H_{17}N_4O$ $[M-BF_4]^+$: 245.1397, found 245.1395.

(E)-Bis(N,N-dimethylamino)-((4-chlorophenyl)diazenyl) cyclopropenium tetrafluoroborate (6e)

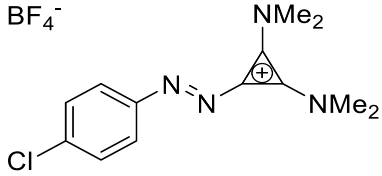

6e

6e was prepared according to **GP2.3** using *N*-Boc-*N*-(4-chlorophenyl)hydrazide (**SI-4e**) (30 mg, 0.12 mmol, 1.0 equiv), chloro-bis(dimethylamino)cyclopropenium tetrafluoroborate (**1b**) (40 mg, 1.6 mmol, 1.3 equiv), and pyridine (12 µL, 1.5 mmol, 1.2 equiv) in dry $CHCl_3$ (0.5 mL, 1 M). The reaction was heated at 50 °C for 1 h. Benzoquinone (27 mg, 0.25 mmol, 2.0 equiv) was added, the reaction stirred for 10 min, and concentrated under reduced pressure. The crude was dissolved in $CHCl_3$/*i*-PrOH (30 mL, 3:1, [v/v]), washed with HBF_4 (15 mL, 5%, [w/w]), and the aqueous layer extracted with $CHCl_3$/*i*-PrOH (3x10 mL, 3:1, [v/v]). The combined organic layers were dried over Na_2SO_4 and concentrated under reduced pressure. Precipitation (2x) from MeCN (1 mL) and Et_2O (10 mL) afforded (22 mg, 0.063 mmol, 51%) as orange solid.

^{1}H-NMR (400 MHz, CD_3CN): δ / ppm = 7.94 – 7.90 (m, 2H), 7.65 – 6.61 (m, 2H), 3.47 (s, 6H), 3.32 (s, 6H).

^{13}C-NMR (101 MHz, CD_3CN): δ / ppm = 152.7, 140.9, 131.8, 131.1, 125.9, 110.7, 44.9, 43.2.

^{19}F-NMR (377 MHz, CD_3CN): δ / ppm = -152.

IR (Diamond-ATR, neat): $\tilde{\nu}$ / cm^{-1} = 3091, 2950, 1897, 1689, 1632, 1582, 1570, 1453, 1437, 1423, 1412, 1286, 1226, 1201, 1086, 1053, 1039, 1006, 955, 858, 788, 715, 699, 627, 586, 521, 505.

HRMS (ESI) calcd. for $C_{13}H_{16}ClN_4$ $[M-BF_4]^+$: 263.1058, found 263.1054.

(*E*)-Bis(*N,N*-dimethylamino)-((4-bromophenyl)diazenyl) cyclopropenium tetrafluoroborate (6f)

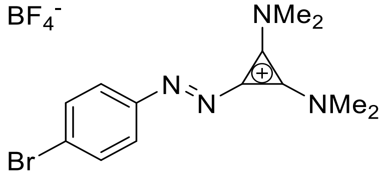

6f

6f was prepared according to **GP2.3** using *N*-Boc-*N*-(4-bromophenyl)hydrazide (**SI-4f**) (36 mg, 0.13 mmol, 1.0 equiv), chloro-bis(dimethylamino)cyclopropenium tetrafluoroborate (**1b**) (40 mg, 1.6 mmol, 1.3 equiv), and pyridine (12 µL, 1.5 mmol, 1.2 equiv) in dry $CHCl_3$ (0.5 mL, 1 M). The reaction was heated at 50 °C for 1 h. Benzoquinone (27 mg, 0.25 mmol, 2.0 equiv) was added, the reaction stirred for 10 min, and concentrated under reduced pressure. The crude was dissolved in $CHCl_3$/*i*-PrOH (30 mL, 3:1, [v/v]), washed with HBF_4 (15 mL, 5%, [w/w]), and the aqueous layer extracted with $CHCl_3$/*i*-PrOH (5x10 mL, 3:1, [v/v]). The combined organic layers were dried over Na_2SO_4 and concentrated under reduced pressure. Precipitation (2x) from MeCN (1 mL) and Et_2O (10 mL) afforded **6f** (27 mg, 0.068 mmol, 55%) as orange solid.

^{1}H-NMR (500 MHz, CD_3CN): δ / ppm = 7.86 – 7.81 (m, 2H), 7.81 – 7.77 (m, 2H), 3.47 (s, 6H), 3.32 (s, 6H).

^{13}C-NMR (126 MHz, CD_3CN): δ / ppm = 153.0, 134.1, 131.8, 129.6, 125.9, 110.7, 44.9, 43.3.

^{19}F-NMR (471 MHz, CD_3CN): δ / ppm = -152.

IR (Diamond-ATR, neat): $\tilde{\nu}$ / cm^{-1} = 3087, 2947, 1903, 1689, 1635, 1580, 1567, 1452, 1436, 1423, 1411, 1305, 1284, 1226, 1201, 1096, 1054, 1039, 1004, 956, 856, 801, 788, 766, 711, 689, 625, 585, 521, 506.

HRMS (ESI) calcd. for $C_{13}H_{16}BrN_4$ $[M-BF_4]^+$: 307.0553, found 307.0554.

(*E*)-Bis(*N*,*N*-dimethylamino)-((4-(trifluoromethyl)phenyl)diazenyl) cyclopropenium tetrafluoroborate (6g)

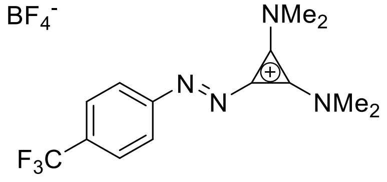

6g

6g was prepared according to **GP2.3** using *N*-Boc-*N*-(4-trifluoromethylphenyl)hydrazide (**4g**) (35 mg, 0.13 mmol, 1.0 equiv) chloro-bis(dimethylamino)cyclopropenium tetrafluoroborate (**1b**) (41 mg, 1.7 mmol, 1.3 equiv), and pyridine (12 µL, 1.5 mmol, 1.2 equiv) in dry $CHCl_3$ (0.5 mL, 1 M). The reaction was heated at 50 °C for 2 h. Benzoquinone (27 mg, 0.25 mmol, 2.0 equiv) was added and the reaction worked up as described in **GP2.3**. Purification by flash column chromatography (SiO_2, CH_2Cl_2:MeOH = 1:0 to 95:5, R_f = 0.25 in CH_2Cl_2:MeOH = 97:3) and precipitation from MeCN (1 mL) and Et_2O (10 mL) afforded **6g** (31 mg, 0.081 mmol, 64%) as orange solid.

^{1}H-NMR (400 MHz, CD_3CN): δ / ppm = 8.10 – 8.03 (m, 2H), 7.96 - 7.88 (m, 2H), 3.50 (s, 6H), 3.35 (s, 6H).

^{13}C-NMR (101 MHz, CD_3CN): δ / ppm = 155.9 (q, *J* = 2 Hz), 134.8 (q, *J* = 33 Hz), 132.2, 127.9 (q, *J* = 4 Hz), 124.9 (q, *J* = 272 Hz), 124.8, 110.4, 45.1, 43.4.

^{19}F-NMR (377 MHz, CD_3CN): δ / ppm = -63, -152.

IR (Diamond-ATR, neat): $\tilde{\nu}$ / cm^{-1} = 3104, 2950, 1905, 1638, 1609, 1455, 1421, 1382, 1321, 1228, 1204, 1171, 1125, 1107, 1051, 1038, 959, 862, 833, 789, 747, 685, 609, 521.

HRMS (ESI) calcd. for $C_{14}H_{16}F_3N_4$ $[M-BF_4]^+$: 297.1322, found 297.1317.

(*E*)-Bis(*N*,*N*-dimethylamino)-((4-methyl carbonoylphenyl) diazenyl)cyclopropenium tetrafluoroborate (6h)

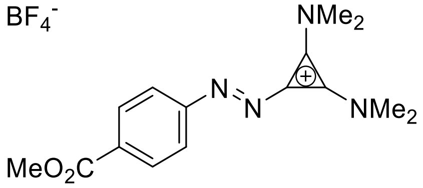

6h

6h was prepared according to **GP2.3** using *N*-Boc-*N*-(3-(methoxycarbonyl)phenyl)hydrazide (**SI-4h**) (33 mg, 0.12 mmol, 1.0 equiv), chloro-bis(dimethylamino)cyclopropenium tetrafluoroborate (**1b**) (40 mg, 1.6 mmol, 1.3 equiv), and pyridine (12 µL, 1.5 mmol, 1.2 equiv) in dry $CHCl_3$ (0.5 mL, 1 M). The reaction was heated at 50 °C for 2 h. Benzoquinone (27 mg, 0.25 mmol, 2.0 equiv) was added and the reaction worked up as described in **GP2.3**. Purification by flash column chromatography (SiO_2, EtOAc, then CH_2Cl_2:MeOH = 1:0 to 95:5, R_f = 0.25 in CH_2Cl_2:MeOH = 95:5) and precipitation from MeCN (1 mL) and Et_2O (10 mL) afforded **6h** (30 mg, 0.080 mmol, 65%) as orange solid.

^{1}H-NMR (500 MHz, CD_3CN): δ / ppm = 8.22 – 8.18 (m, 2H), 8.02 – 7.98 (m, 2H), 3.92 (s, 3H), 3.49 (s, 6H), 3.34 (s, 6H).

^{13}C-NMR (126 MHz, CD_3CN): δ / ppm = 166.8, 156.3, 135.5, 132.1, 131.7, 124.3, 110.7, 53.2, 45.0, 43.3.

^{19}F-NMR (471 MHz, CD_3CN): δ / ppm = -152.

IR (Diamond-ATR, neat): $\tilde{\nu}$ / cm^{-1} = 2953, 1902, 1718, 1637, 1434, 1373, 1281, 1198, 1049, 955, 875, 863, 822, 782, 764, 698, 587, 521.

HRMS (ESI) calcd. for $C_{15}H_{19}N_4O_2$ [M-BF_4]$^+$: 287.1503, found 287.1503.

(E)-Bis(N,N-dimethylamino)-((4-carboxyphenyl)diazenyl) cyclopropenium tetrafluoroborate (6i)

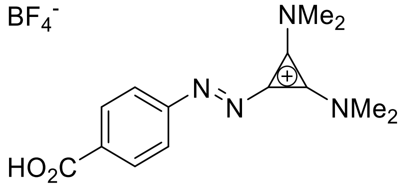

6i

6i was prepared according to **GP2.3** using *N*-Boc-*N*-(4-(tert-butoxycarbonyl)phenyl)hydrazide (**SI-4i**) (39 mg, 0.13 mmol, 1.0 equiv), chloro-bis(dimethylamino)cyclopropenium tetrafluoroborate (**1b**) (41 mg, 1.7 mmol, 1.3 equiv), and pyridine (12 µL, 1.5 mmol, 1.2 equiv) in dry $CHCl_3$ (0.5 mL, 1 M). The reaction was heated at 50 °C for 2 h. Benzoquinone (27 mg, 0.25 mmol, 2.0 equiv) was added and the reaction worked up as described in **GP2.3**. Precipitation from MeCN (3 mL) and Et_2O (15 mL) afforded **6i** (31 mg, 0.086 mmol, 68%) as orange solid.

^{1}H-NMR (500 MHz, CD_3CN): δ / ppm = 8.22 – 8.18 (m, 2H), 8.01 – 7.97 (m, 2H), 3.49 (s, 6H), 3.34 (s, 6H).

^{13}C-NMR (126 MHz, CD_3CN): δ / ppm = 166.8, 156.4, 135.3, 132.1, 132.0, 124.3, 110.7, 45.0, 43.3.

^{19}F-NMR (471 MHz, CD_3CN): δ / ppm = -152.

IR (Diamond-ATR, neat): $\tilde{\nu}$ / cm^{-1} = 3565, 3509, 3252, 2944, 2808, 2635, 2524, 1898, 1710, 1631, 1489, 1451, 1431, 1413, 1377, 1314, 1300, 1267, 1227, 1197, 1048, 1008, 959, 868, 844, 821, 789, 777, 765, 750, 737, 691, 613.

HRMS (ESI) calcd. for $C_{14}H_{17}N_4O_2$ $[M-BF_4]^+$: 273.1346, found 273.1341.

(*E*)-Bis(*N,N*-dimethylamino)-((4-cyanophenyl)diazenyl) cyclopropenium tetrafluoroborate (6j)

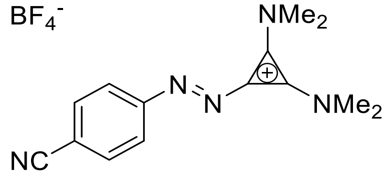

6j

6j was prepared according to **GP2.3** using *N*-Boc-*N*-(4-cyanophenyl)hydrazide (**SI-4j**) (29 mg, 0.13 mmol, 1.0 equiv), chloro-bis(dimethylamino)cyclopropenium tetrafluoroborate (**1b**) (40 mg, 1.6 mmol, 1.3 equiv), and pyridine (12 µL, 1.5 mmol, 1.2 equiv) in dry $CHCl_3$ (0.5 mL, 1 M). The reaction was heated at 50 °C for 4 h. Benzoquinone (27 mg, 0.25 mmol, 2.0 equiv) was added and the reaction worked up as described in **GP2.3**. Purification by flash column chromatography (SiO_2, EtOAc, then CH_2Cl_2:MeOH = 1:0 to 95:5, R_f = 0.25 in CH_2Cl_2:MeOH = 95:5) and precipitation from MeCN (1 mL) and Et_2O (10 mL) afforded **6j** (24 mg, 0.070 mmol, 56%) as orange solid.

^{1}H-NMR (500 MHz, CD_3CN): δ / ppm = 8.05 – 8.01 (m, 2H), 7.97 – 7.93 (m, 2H), 3.49 (s, 6H), 3.34 (m, 2H).

^{13}C-NMR (126 MHz, CD_3CN): δ / ppm = 155.7, 134.9, 132.2, 124.7, 119.0, 117.2, 110.4, 45.1, 43.4.

^{19}F-NMR (471 MHz, CD_3CN): δ / ppm = -152.

IR (Diamond-ATR, neat): $\tilde{\nu}$ / cm^{-1} = 3612, 3094, 2953, 2225, 1895, 1637, 1452, 1435, 1412, 1372, 1291, 1270, 1228, 1202, 1051, 957, 862, 784, 719, 654, 587, 547, 522.

HRMS (ESI) calcd. for $C_{14}H_{16}N_5$ $[M-BF_4]^+$: 254.1400, found 254.1396.

(E)-Bis(N,N-dimethylamino)-((3-methoxyphenyl)diazenyl) cyclopropenium tetrafluoroborate (6k)

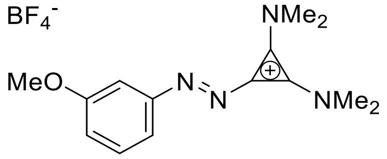

6k

6k was prepared according to **GP2.3** using *N*-Boc-*N*-(3-methoxyphenyl)hydrazide (30 mg, 0.13 mmol, 1.0 equiv), chloro-bis(dimethylamino)cyclopropenium tetrafluoroborate (**1b**) (40 mg, 1.6 mmol, 1.3 equiv), and pyridine (12 µL, 1.5 mmol, 1.2 equiv) in dry $CHCl_3$ (0.5 mL, 1 M). The reaction was heated at 50 °C for 1 h. Benzoquinone (27 mg, 0.25 mmol, 2.0 equiv) was added and the reaction worked up as described in **GP2.3**. Purification by flash column chromatography (SiO_2, EtOAc, then CH_2Cl_2:MeOH = 1:0 to 97:3, R_f = 0.35, in CH_2Cl_2:MeOH = 97:3) afforded **6k** (35 mg, 0.10 mmol, 80%) as orange solid.

^{1}H-NMR (500 MHz, CD_3CN): δ / ppm = 7.59 – 7.56 (m, 1H), 7.55 – 7.50 (m, 1H), 7.46 – 7.42 (m, 1H), 7.26 – 7.22 (m, 1H), 3.87 (s, 3H), 3.48 (s, 6H), 3.32 (s, 6H).

^{13}C-NMR (126 MHz, CD_3CN): δ / ppm = 161.7, 155.4, 131.8, 131.6, 121.9, 119.1, 110.8, 107.0, 56.4, 44.9, 43.2.

^{19}F-NMR (471 MHz, CD_3CN): δ / ppm = -152.

IR (Diamond-ATR, neat): $\tilde{\nu}$ / cm^{-1} = 3635, 2946, 1903, 1633, 1595, 1483, 1449, 1418, 1333, 1283, 1250, 1227, 1046, 1030, 992, 913, 873, 789, 765, 742, 731, 686, 584, 559, 520.

HRMS (ESI) calcd. for $C_{14}H_{19}N_4O$ [M-BF_4]$^+$: 259.1553, found 259.1552.

(E)-Bis(N,N-dimethylamino)-((3-methyl carbonoylphenyl) diazenyl)cyclopropenium tetrafluoroborate (6l)

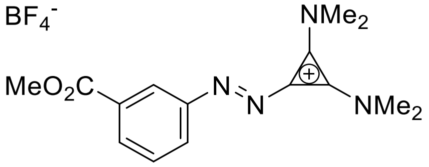

6l

6l was prepared according to **GP2.3** using *N*-Boc-*N*-(3-(methoxycarbonyl)phenyl)hydrazide (**SI-4l**) (33 mg, 0.12 mmol, 1.0 equiv), chloro-bis(dimethylamino)cyclopropenium tetrafluoroborate (**1b**) (40 mg, 1.6 mmol, 1.3 equiv), and pyridine (12 µL, 1.5 mmol, 1.2 equiv) in dry $CHCl_3$ (0.5 mL, 1 M). The reaction was heated at 50 °C for 1 h. Benzoquinone (27 mg, 0.25 mmol, 2.0 equiv) was added and the reaction worked up as described in **GP2.3**. Purification by flash column chromatography (SiO_2, EtOAc, then CH_2Cl_2:MeOH = 1:0 to 95:5, R_f = 0.30 in CH_2Cl_2:MeOH = 95:5) afforded **6l** (38 mg, 0.10 mmol, 82%) as orange solid.

^{1}H-NMR (500 MHz, CD_3CN): δ / ppm = 8.48 (ddd, J = 2.0, 1.5, 0.5 Hz, 1H), 8.25 (ddd, J = 7.5, 1.5, 1.0, 1H), 8.15z (ddd, J = 8.0, 2.0, 1.0, 1H), 7.74 (ddd, J = 8.0, 7.5, 0.5 Hz, 1H), 3.93 (s, 3H), 3.50 (s, 6H), 3.34 (s, 6H).

^{13}C-NMR (126 MHz, CD_3CN): δ / ppm = 166.7, 154.1, 135.2, 133.1, 132.0, 131.3, 128.4, 124.8, 110.6, 53.2, 45.0, 43.3.

^{19}F-NMR (471 MHz, CD_3CN): δ / ppm = -152.

IR (Diamond-ATR, neat): $\tilde{\nu}$ / cm^{-1} = 2951, 1905, 1721, 1635, 1434, 1419, 1299, 1277, 1228, 1198, 1158, 1049, 1037, 997, 959, 909, 822, 789, 761, 704, 684, 587, 521.

HRMS (ESI) calcd. for $C_{15}H_{19}N_4O_2$ $[M-BF_4]^+$: 287.1503, found 287.1502.

Bis(*N*,*N*-dimethylamino)-((*E*,*E*)-1,4-phenylenebis(diazenyl)) cyclopropenium tetrafluoroborate (6m)

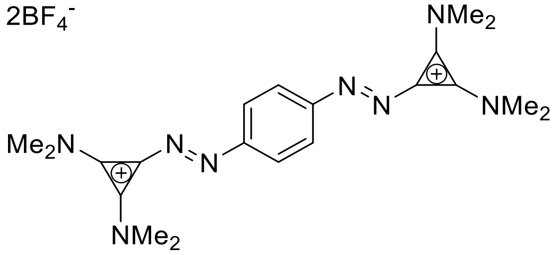

6m

6n was prepared according to **GP2.3** using di-tert-butyl 1,1'-(1,4-phenylene)bis(hydrazine-1-carboxylate) (**SI-4m**) (42 mg, 0.12 mmol, 1.0 equiv), chloro-bis(dimethylamino)cyclopropenium tetrafluoroborate (**1b**) (80 mg, 0.32 mmol, 2.6 equiv), and pyridine (24 µL, 0.30 mmol, 2.4 equiv) in dry $CHCl_3$ (0.5 mL, 1 M). The reaction was heated at 50 °C for 2 h. Benzoquinone (54 mg, 0.50 mmol, 4.0 equiv) was added, followed by TFA (0.5 mL). After stirring for 10 min at ambient temperature, the reaction was concentrated under reduced pressure. The crude was dissolved in MeCN (3 mL) and Et_2O (6 mL) added slowly. The obtained precipitate was collected by filtration and dissolved in MeCN (3 mL). $NaBF_4$ (145 mg) was added and the suspension stirred at ambient temperature for 6 h. The mixture was diluted with $CHCl_3$ (3 mL) and filtered over a short plug of celite. The plug was washed with MeCN/$CHCl_3$ (1:1, 1 mL) and the combined filtrates concentrated under reduced pressure. Precipitation from MeCN (3 mL) and Et_2O (10 mL) afforded **6n** (38 mg, 0.069 mmol, 55%) as purple solid.

^{1}H-NMR (500 MHz, CD_3CN): δ / ppm = 8.12 (s, 4H), 3.51 (s, 12H), 3.35 (s, 12H).

^{13}C-NMR (126 MHz, CD_3CN): δ / ppm = 156.6, 132.1, 125.9, 110.9, 45.1, 43.4.

^{19}F-NMR (471 MHz, CD_3CN): δ / ppm = -152.

IR (Diamond-ATR, neat): $\tilde{\nu}$ / cm^{-1} = 2945, 1897, 1722, 1634, 1451, 1426, 1202, 1049, 957, 870, 849, 788, 741, 601, 521.

HRMS (ESI) calcd. for $C_{20}H_{28}N_8$ $[M-2BF_4]^{2+}$: 190.1213, found 190.1215.

(*E*)-Bis(*N,N*-dimethylamino)-((thiophene-3-yl)diazenyl) cyclopropenium tetrafluoroborate (6n)

and

(*E*)-Bis(*N,N*-dimethylamino)-((2-chloro-thiophene-3-yl)diazenyl) cyclopropenium tetrafluoroborate (6n')

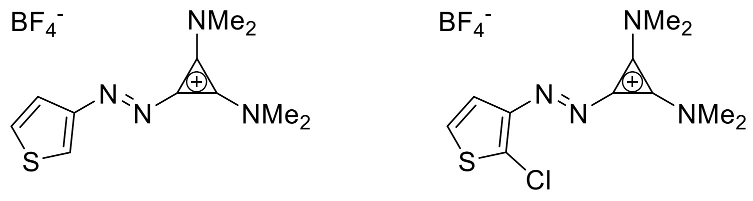

6n **6n'**

6n was prepared according to **GP2.3** using *N*-Boc-*N*-(thiophen-3-yl)hydrazide (**SI-4n**) (27 mg, 0.13 mmol, 1.0 equiv), chloro-bis(dimethylamino)cyclopropenium tetrafluoroborate (**1b**) (40 mg, 1.6 mmol, 1.3 equiv), and pyridine (12 uL, 1.5 mmol, 1.2 equiv) in dry $CHCl_3$ (0.5 mL, 1 M). The reaction was heated at 50 °C for 1 h. Benzoquinone (27 mg, 0.25 mmol, 2.0 equiv) was added and the reaction worked up as described in **GP2.3**. Purification by flash column chromatography (SiO_2, EtOAc, then CH_2Cl_2:MeOH = 1:0 to 95:5, R_f(**6n**/**6n'**) = 0.25 in CH_2Cl_2:MeOH = 95:5) afforded a mixture of **6n** and **6n'** (22 mg of a 2:1 mixture as determined by ^{1}H NMR; **6n**: 14 mg, 0.043 mmol, 34%; **6n'**: 8.0 mg, 0.022 mmol, 18%) as yellow solid. Analytically pure samples of **6n** were obtained by iterated fractionated precipitation from MeCN/Et_2O.

6n:

^{1}H-NMR (400 MHz, CD_3CN): δ / ppm = 8.47 (dd, *J* = 3.0, 1.5 Hz, 1H), 7.57 (dd, *J* = 5.5, 3.0 Hz, 1H), 7.48 (dd, *J* = 5.5, 1.5 Hz, 1H), 3.44 (s, 6H), 3.30 (s, 6H).

^{13}C-NMR (101 MHz, CD_3CN): δ / ppm = 159.0, 136.1, 131.6, 129.8, 117.7, 111.4, 44.7, 43.1.

^{19}F-NMR (377 MHz, CD_3CN): δ / ppm = -152.

IR (Diamond-ATR, neat): $\tilde{\nu}$ / cm^{-1} = 3109, 2960, 1907, 1630, 1494, 1452, 1425, 1414, 1352, 1261, 1222, 1097, 1049, 1038, 961, 881, 827, 793, 739, 713, 652, 550, 521, 499, 473.

HRMS (ESI) calcd. for $C_{11}H_{15}N_4S$ $[M-BF_4]^+$: 235.1012, found 235.1016.

6n':

^{1}H-NMR (500 MHz, CD_3CN): δ / ppm = 7.44 (d, *J* = 6.1 Hz, 1H), 7.29 (d, *J* = 6.1 Hz, 1H), 3.47 (s, 6H), 3.31 (s, 6Hz).

^{13}C-NMR (126 MHz, CD_3CN): δ / ppm = 153.1, 142.6, 131.7, 127.1, 116.6, 111.5, 44.8, 43.2.

^{19}F-NMR (471 MHz, CD_3CN): δ / ppm = -152.

HRMS (ESI) calcd. for $C_{11}H_{14}ClN_4S$ $[M(^{35}Cl)-BF_4]^+$: 269.0622, found 269.0615; calcd. for $C_{11}H_{14}ClN_4S$ $[M(^{37}Cl)-BF_4]^+$: 271.0593, found 271.0589.

(*E*)-Bis(*N*,*N*-dimethylamino)-((1-methyl-1*H*-pyrazol-4-yl)diazenyl) cyclopropenium tetrafluoroborate (6o)

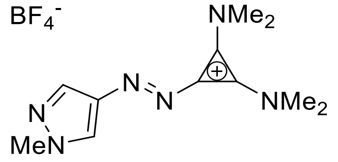

6o

6o was prepared according to **GP2.3** using *N*-Boc-*N*-(1-methyl-1*H*-pyrazol-4-yl)hydrazide (**SI-4o**) (27 mg, 0.13 mmol, 1.0 equiv), chloro-bis(dimethylamino)cyclopropenium tetrafluoroborate (**1b**) (41 mg, 1.7 mmol, 1.3 equiv), and pyridine (12 µL, 1.5 mmol, 1.2 equiv) in dry $CHCl_3$ (0.5 mL, 1 M). The reaction was heated at 50 °C for 1 h. Benzoquinone (27 mg, 0.25 mmol, 2.0 equiv) was added and the reaction worked up as described in **GP2.3**. Precipitation from Et_2O/MeCN (10:1) afforded (23 mg, 0.072 mmol, 56%) as yellow solid.

^{1}H-NMR (500 MHz, CD_3CN): δ / ppm = 8.27 (s, 1H), 7.96 (s, 1H), 3.93 (s, 3H), 3.40 (s, 6H), 3.27 (s, 6H).

^{13}C-NMR (126 MHz, CD_3CN): δ / ppm = 144.2, 133.8, 131.9, 130.9, 112.1, 44.5, 42.9, 40.4.

^{19}F-NMR (471 MHz, CD_3CN): δ / ppm = -152.

IR (Diamond-ATR, neat): $\tilde{\nu}$ / cm^{-1} = 3123, 2951, 1907, 1628, 1524, 1506, 1493, 1452, 1436, 1409, 1384, 1348, 1264, 1217, 1190, 1167, 1047, 1034, 1011, 964, 896, 862, 827, 788, 766, 747, 731, 667, 640, 630.

HRMS (ESI) calcd. for $C_{11}H_{17}N_6$ $[M-BF_4]^+$: 233.1509, found 233.1510

(*E*)-Bis(*N*,*N*-dimethylamino)-((2,6-dimethylphenyl)diazenyl) cyclopropenium tetrafluoroborate (6p)

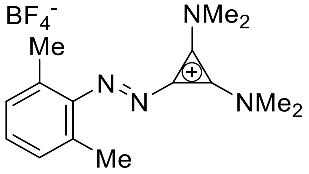

6p

6p was prepared according to **GP2.3** using (2,6-methylphenyl)hydrazine hydrochloride (23 mg, 0.13 mmol, 1.0 equiv), chloro-bis(dimethylamino)cyclopropenium tetrafluoroborate (**1b**) (41 mg, 0.17 mmol, 1.3 equiv), and pyridine (22 µL, 0.27 mmol, 2.1 equiv) in dry $CHCl_3$ (0.5 mL). The reaction was heated at 50 °C for 3 h. TFA (0.5 mL) was added, followed by Benzoquinone (27 mg, 0.25 mmol, 2.0 equiv) and the reaction worked up as described in **GP2.3**. Purification by flash column chromatography (EtOAc, then CH_2Cl_2:MeOH = 1:0 to 95:5, R_f = 0.30 in CH_2Cl_2:MeOH = 95:5) afforded **6p** (23 mg, 0.067 mmol, 52%) as orange solid.

^{1}H-NMR (500 MHz, CD_3CN): δ / ppm = 7.37 – 7.33 (m, 1H), 7.27 – 7.22 (m, 2H), 3.46 (s, 6H), 3.32 (s, 6H), 2.48 (s, 6H).

^{13}C-NMR (126 MHz, CD_3CN): δ / ppm = 151.3, 136.0, 133.7, 131.4, 131.2, 111.0, 44.7, 43.1, 20.9.

^{19}F-NMR (471 MHz, CD_3CN): δ / ppm = -152.

IR (Diamond-ATR, neat): $\tilde{\nu}$ / cm^{-1} = 3627, 2952, 1900, 1630, 1587, 1476, 1437, 1424, 1416, 1376, 1287, 1228, 1190, 1099, 1056, 1036, 964, 905, 857, 810, 790, 700, 640.

HRMS (ESI) calcd. for $C_{15}H_{21}N_4$ $[M-BF_4]^+$: 257.1761, found 257.1764.

(*E*)-1,2-Bis(*N,N*-dimethylamino)-3-((2,6-difluorophenyl)diazenyl) cyclopropenium tetrafluoroborate (6q)

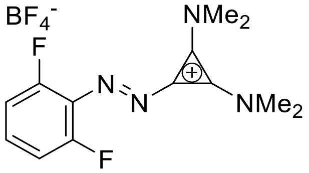

6q

6q was prepared according to **GP2.3** using (2,6-difluorophenyl)hydrazine hydrochloride (23 mg, 0.13 mmol, 1.0 equiv), chloro-bis(dimethylamino)cyclopropenium tetrafluoroborate (**1b**) (41 mg, 0.17 mmol, 1.3 equiv), and pyridine (22 µL, 0.27 mmol, 2.1 equiv) in dry $CHCl_3$ (0.5 mL, 1 M). The reaction was heated at 50 °C for 1 h. TFA (0.5 mL) was added, followed by benzoquinone (27 mg, 0.25 mmol, 2.0 equiv) and the reaction worked up as described in **GP2.3**. Purification by flash column chromatography (EtOAc, then CH_2Cl_2:MeOH = 1:0 to 95:5, Rf = 0.25 in CH_2Cl_2:MeOH = 95:5) afforded **6q** (26 mg, 0.074 mmol, 58%) as orange solid.

^{1}H-NMR (400 MHz, CD_3CN): δ / ppm = 7.63 (tt, *J* = 8.5, 8.5, 6.0, 6.0 Hz, 1H), 7.68 – 7.59 (m, 1H), 3.46 (s, 6H), 3.33 (s, 6H).

^{13}C-NMR (101 MHz, CD_3CN): δ / ppm = 157.2 (dd, *J* = 265, 4 Hz), 136.6 (t, *J* = 11 Hz), 132.1 (t, *J* = 9 Hz), 131.9, 114.5 – 114.1 (m), 111.2, 45.0, 43.3.

^{19}F-NMR (377 MHz, CD_3CN): δ / ppm = -118 (dd, *J* = 10, 6 Hz), -152.

IR (Diamond-ATR, neat): $\tilde{\nu}$ / cm^{-1} = 3627, 3062, 1903, 1643, 1565, 1547, 1432, 1425, 1371, 1280, 1227, 1207, 1188, 1051, 1035, 956, 859, 826, 793, 758, 736, 682.

HRMS (ESI) calcd. for $C_{13}H_{15}F_2N_4$ $[M-BF_4]^+$: 265.1259, found 265.1262.

(*E*)-Bis(*N,N*-dimethylamino)-((2,6-dichlorophenyl)diazenyl) cyclopropenium tetrafluoroborate (6r)

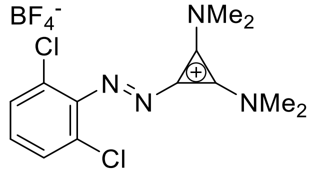

6r

6r was prepared according to **GP2.3** using (2,6-dichlorophenyl)hydrazine hydrochloride (27 mg, 0.13 mmol, 1.0 equiv) chloro-bis(dimethylamino)cyclopropenium tetrafluoroborate (**1b**) (41 mg, 0.17 mmol, 1.3 equiv), and pyridine (22 μL, 0.27 mmol, 2.2 equiv) in dry $CHCl_3$ (0.5 mL, 1.0 M). The reaction was heated at 50 °C for 10 h. TFA (0.5 mL) was added, followed by benzoquinone (27 mg, 0.25 mmol, 2.0 equiv) and the reaction worked up as described in **GP2.3**. Purification by flash column chromatography (SiO_2, EtOAc, then CH_2Cl_2:MeOH = 1:0 to 95:5, R_f = 0.25 in CH_2Cl_2:MeOH = 95:5) and precipitation from Et_2O/MeCN (6:1) afforded **6r** (22 mg, 0.057 mmol, 45%) as orange solid.

^{1}H-NMR (500 MHz, CD_3CN): δ / ppm = 7.58 (d, *J* = 8.5 Hz, 1H), 7.58 (d, *J* = 7.5 Hz, 1H), 7.44 (dd, *J* = 8.5, 7.5 Hz, 1H), 3.48 (s, 6H), 3.35 (s, 6H).

^{13}C-NMR (126 MHz, CD_3CN): δ / ppm = 147.4, 133.2, 132.4, 131.3, 129.7, 109.9, 45.1, 43.4.

^{19}F-NMR (471 MHz, CD_3CN): δ / ppm = -152.

IR (Diamond-ATR, neat): $\tilde{\nu}$ / cm^{-1} = 3627, 3062, 1903, 1643, 1565, 1547, 1432, 1425, 1371, 1280, 1227, 1207, 1188, 1051, 1035, 956, 859, 826, 793, 758, 736, 682.

HRMS (ESI) calcd. for $C_{13}H_{15}Cl_2N_4$ $[M-BF_4]^+$: 297.0668, found 297.0670.

(E)-Bis(N,N-dimethylamino)-((1-methyl-1H-pyrazol-3-yl)diazenyl) cyclopropenium tetrafluoroborate (6s)

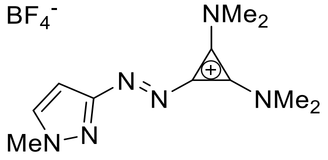

6s

6s was prepared according to **GP2.3** using *N*-Boc-*N*-(1-methyl-1*H*-pyrazol-3-yl)hydrazide (**SI-4s**) (27 mg, 0.13 mmol, 1.0 equiv), chloro-bis(dimethylamino)cyclopropenium tetrafluoroborate (**1b**) (41 mg, 1.7 mmol, 1.3 equiv), and pyridine (12 µL, 1.5 mmol, 1.2 equiv) in dry $CHCl_3$ (0.5 mL, 1 M). The reaction was heated at 50 °C for 1 h. Benzoquinone (27 mg, 0.25 mmol, 2.0 equiv) was added and the reaction worked up as described in **GP2.3**. Precipitation from Et_2O/MeCN (10:1) afforded **6s** (22 mg, 0.069 mmol, 54%) as yellow solid.

^{1}H-NMR (500 MHz, CD_3CN): δ / ppm = 7.62 (d, J = 2.5 Hz, 1H), 6.61 (d, J = 2.5 Hz, 1H), 4.00 (s, 3H), 3.44 (s, 6H), 3.30 (s, 6H).

^{13}C-NMR (126 MHz, CD_3CN): δ / ppm = 165.4, 134.8, 131.6, 111.3, 97.0, 44.7, 43.1, 40.7.

^{19}F-NMR (471 MHz, CD_3CN): δ / ppm = -152.

IR (Diamond-ATR, neat): $\tilde{\nu}$ / cm^{-1} = 3129, 2951, 1907, 1640, 1446, 1435, 1424, 1410, 1385, 1349, 1299, 1268, 1212, 1047, 1035, 1006, 955, 828, 787, 728, 703, 626.

HRMS (ESI) calcd. for $C_{11}H_{17}N_6$ $[M-BF_4]^+$: 233.1509, found 233.1507.

(E)-Bis(N,N-dimethylamino)-((1-methyl-1H-indazol-3-yl)diazenyl) cyclopropenium tetrafluoroborate (6t)

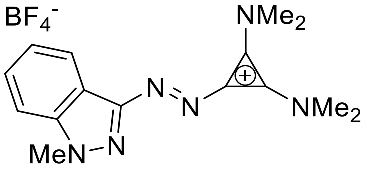

6t

6t was prepared according to **GP2.3** using *N*-Boc-*N*-(1-methyl-1*H*-pyrazol-3-yl)hydrazide (**SI-4t**) (33 mg, 0.13 mmol, 1.0 equiv), chloro-bis(dimethylamino)cyclopropenium tetrafluoroborate (**1b**) (40 mg, 1.6 mmol, 1.3 equiv), and pyridine (12 µL, 1.5 mmol, 1.2 equiv) in dry $CHCl_3$ (0.5 mL, 1 M). The reaction was heated at 50 °C for 1 h. Benzoquinone (27 mg, 0.25 mmol, 2.0 equiv) was added and the reaction worked up as described in **GP2.3**. Precipitation from Et_2O/MeCN (10:1) afforded of **6t** (24 mg, 0.065 mmol, 52%) as yellow solid.

^{1}H-NMR (500 MHz, CD_3CN): δ / ppm = 8.27 (dt, *J* = 8.0, 1.0, 1.0 Hz, 1H), 7.69 (dt, *J* = 8.5, 1.0, 1.0 Hz, 1H), 7.60 (ddd, *J* = 8.5, 7.0, 1.0 Hz, 1H), 7.47 (ddd, *J* = 8.0, 7.0, 1.0 Hz, 1H), 4.25 (s, 3H), 3.51 (s, 6H), 3.32 (s, 6H).

^{13}C-NMR (101 MHz, CD_3CN): δ / ppm = 156.9, 143.1, 131.2, 129.4, 127.3, 124.1, 115.3, 112.2, 111.6, 44.8, 43.1, 38.0.

^{19}F-NMR (471 MHz, CD_3CN): δ / ppm = -152.

IR (Diamond-ATR, neat): $\tilde{\nu}$ / cm^{-1} = 3629, 2945, 1909, 1628, 1463, 1416, 1366, 1332, 1300, 1247, 1222, 1049, 953, 839, 790, 772, 724, 672, 568, 521.

HRMS (ESI) calcd. for $C_{15}H_{19}N_6$ $[M-BF_4]^+$: 283.1666, found 283.1665.

(E)-Bis(N,N-dimethylamino)-((1-benzyl-1H-1,2,3-triazole-4-yl)diazenyl)cyclopropenium tetrafluoroborate (6u)

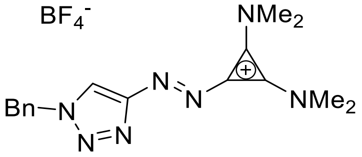

6u

6u was prepared according to **GP2.3** using *N*-Boc-*N*-(1-benzyl-1*H*-1,2,3-triazol-4-yl)hydrazide (**SI-4u-3**) (36 mg, 0.13 mmol, 1.0 equiv), chloro-bis(dimethylamino)cyclopropenium tetrafluoroborate (**1b**) (40 mg, 1.6 mmol, 1.3 equiv), and pyridine (12 µL, 1.5 mmol, 1.2 equiv) in dry $CHCl_3$ (0.5 mL, 1 M). The reaction was heated at 50 °C for 1 h. Benzoquinone (27 mg, 0.25 mmol, 2.0 equiv) and TFA (0.5 mL) were added and the reaction worked up as described in **GP2.3**. Flash column chromatography (EtOAc, then CH_2Cl_2:MeOH = 1:0 to 95:5, R_f = 0.25 in CH_2Cl_2:MeOH = 95:5) afforded **6u** (27 mg, 0.068 mmol, 54%) as orange oil.

^{1}H-NMR (400 MHz, CD_3CN): δ / ppm = 8.37 (s, 1H), 7.44 – 7.36 (m, 5H), 5.64 (s, 2H), 3.43 (s, 6H), 3.31 (s, 6H).

^{13}C-NMR (101 MHz, CD_3CN): δ / ppm = 161.8, 135.8, 131.9, 130.1, 129.8, 129.4, 120.1, 110.9, 55.4, 44.9, 43.2.

^{19}F-NMR (377 MHz, CD_3CN): δ / ppm = -152.

IR (Diamond-ATR, neat): $\tilde{\nu}$ / cm^{-1} = 3140, 2945, 1909, 1638, 1532, 1498, 1474, 1455, 1418, 1361, 1285, 1226, 1157, 1049, 1035, 837, 790, 766, 742, 720, 707, 665, 569, 520, 508, 474, 458.

HRMS (ESI) calcd. for $C_{16}H_{20}N_7$ $[M-BF_4]^+$: 310.1775, found 310.1772.

(*E*)-Bis(*N,N*-dimethylamino)-(4-((2,3-bis(dimethylamino) cycloprop-2-en-1-ylidene)ammonio) phenyldiazenyl)cyclopropenium tetrafluoroborate (SI-6v)

and

(*E*)-*N*-(2,3-bis(dimethylamino)cycloprop-2-en-1-ylidene)-4-((4-hydroxyphenyl)diazenyl)benzenaminium tetrafluoroborate (SI-6v')

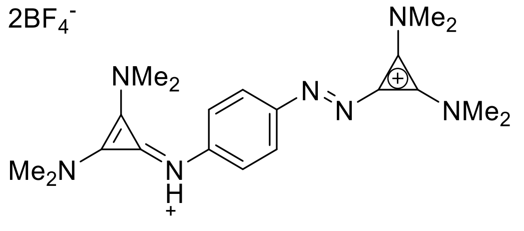

SI-6v

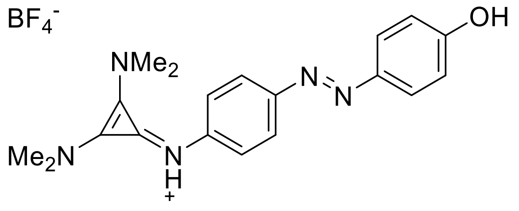

SI-6v'

SI-6v and **SI-6v'** were obtained following **GP2.3** and using chloro-bis(dimethylamino)cyclopropenium tetrafluoroborate (**1b**) (40 mg, 0.16 mmol, 1.3 equiv), *N*-Boc-*N*-(4-aminophenyl)hydrazide (28 mg, 0.13 mmol, 1.0 equiv), pyridine (12 µL, 0.15 mmol, 1.2 equiv), anhydrous $CHCl_3$ (0.5 mL), benzoquinone (27 mg, 0.25 mmol, 2.0 equiv), TFA (0.5 mL). Fractionated precipitation from MeCN and Et_2O afforded **SI-6v** (9.0 mg, 0.017 mmol, 13%) as red solid. **SI-6v'** (9.0 mg, 0.021 mmol, 17%) was obtained by purification of the remaining material by flash column chromatography (SiO_2, EtOAc, then CH_2Cl_2:MeOH = 1:0 to 95:5) as orange solid.

SI-6v:

^{1}H-NMR (400 MHz, CD_3CN): δ / ppm = 8.72 (s, br, 1H), 8.01 – 7.94 (m, 2H), 7.31 – 7.25 (m, 2H), 3.46 (s, 6H), 3.31 (s, 6H), 3.16 (s, 12H).

^{13}C-NMR (101 MHz, CD_3CN): δ / ppm = 150.5, 146.3, 131.4, 126.9, 122.6, 119.3, 111.4, 107.1, 44.8, 43.1, 42.6.

^{19}F-NMR (500 MHz, CD_3CN): δ / ppm = -152.

IR (Diamond-ATR, neat): $\tilde{\nu}$ / cm^{-1} = 3621, 3564, 3296, 2946, 1969, 1910, 1632, 1590, 1534, 1498, 1472, 1452, 1421, 1359, 1310, 1280, 1256, 1230, 1214, 1151, 1051, 962, 874, 855, 791, 775, 764, 738, 726, 675, 621, 591, 547, 521, 479.

HRMS (ESI) calcd. for $C_{20}H_{29}N_7$ $[M-2BF_4]^{2+}$:183.6237, found 183.6238.

SI-6v':

^{1}H-NMR (500 MHz, CD_3CN): δ / ppm = 8.41 (s, br, 1H), 7.91 – 7.87 (m, 2H), 7.83 – 7.79 (m, 2H), 7.58 (s, br, 1H), 7.28 – 7.23 (m, 2H), 6.99 – 6.95 (m, 2H), 3.12 (s, 12H).

^{13}C-NMR (126 MHz, CD_3CN): δ / ppm = 161.1, 150.2, 147.3, 142.3, 125.7, 125.0, 121.3, 120.0, 116.8, 109.0, 42.6.

^{19}F-NMR (471 MHz, CD_3CN): δ / ppm = -152.

IR (Diamond-ATR, neat): $\tilde{\nu}$ / cm^{-1} = 3418, 3322, 2932, 2260, 1981, 1586, 1533, 1490, 1425, 1408, 1349, 1305, 1277, 1252, 1215, 1149, 1055, 960, 848, 836, 796, 785, 739.

HRMS (ESI) calcd. for $C_{19}H_{22}N_5O$ $[M-BF_4]^+$: 336.1819, found 336.1813.

Rearrangement of Heteroaryl Azocyclopropenium Salts

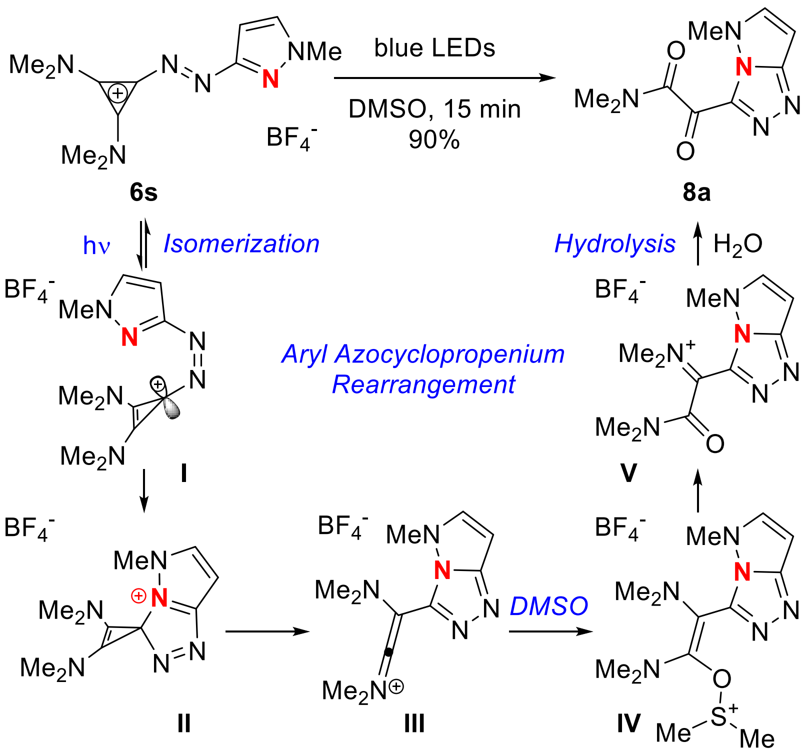

Scheme S1: Proposed mechanism for the formation of **8a** from **6s** in DMSO.

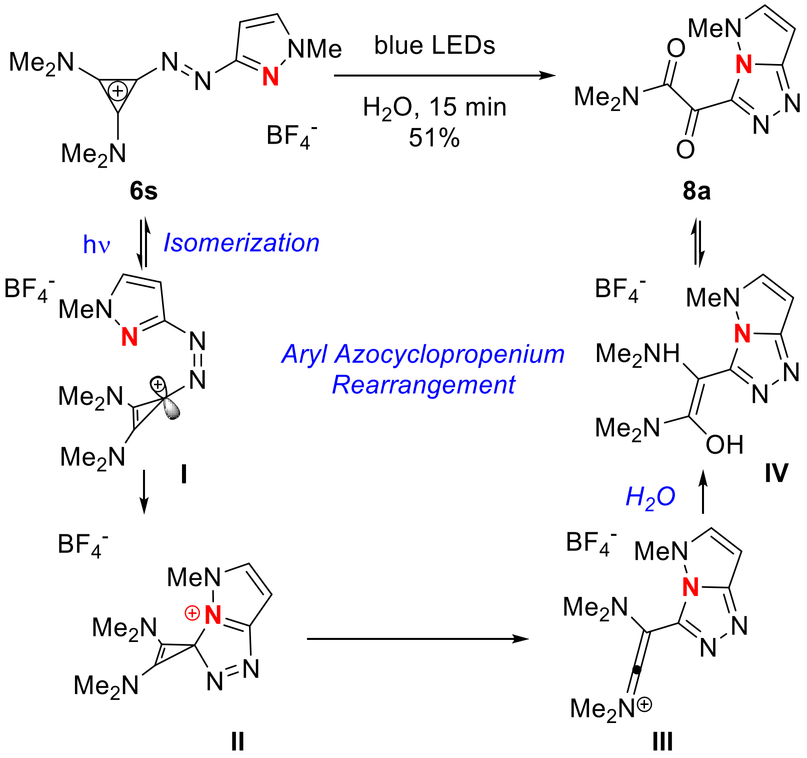

Scheme S2: Proposed mechanism for the formation of **8b** from **6s** in water.

N,N-Dimethyl-2-(5-methyl-5*H*-pyrazolo[5,1-c][1,2,4]triazol-3-yl)-2-oxoacetamide (8a)

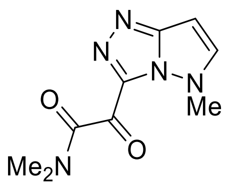

8a

8a was prepared by irradiating a solution of (*E*)-bis(*N*,*N*-dimethylamino)-((1-methyl-1*H*-pyrazol-3-yl)diazenyl)cyclopropenium tetrafluoroborate (**6s**) (32 mg, 0.10 mmol, 1.0 equiv) in DMSO (0.01 M, 10 mL) for 15 min in a blue LED photo reactor. The reaction mixture was lyophilized, sat. $NaHCO_3$-sln. (1 mL) and brine (1 mL) were added, and the resulting mixture extracted with $CHCl_3$/*i*-PrOH (4x4 mL, 3:1, [v/v]). The combined organic layers were dried over Na_2SO_4 and concentrated under reduced pressure. Purification by flash column chromatography (SiO_2, CH_2Cl_2:MeOH = 1:0 to 95:5, R_f = 0.30 in CH_2Cl_2:MeOH = 95:5) afforded **8a** (20 mg, 0.090 mmol, 90%) as colorless solid.

^{1}H-NMR (400 MHz, $CDCl_3$): δ / ppm = 7.47 (d, *J* = 3.5 Hz, 1H), 6.37 (d, *J* = 3.5 Hz, 1H), 4.23 (s, 3H), 3.11 (s, 3H), 3.01 (s, 3H).

^{13}C-NMR (101 MHz, $CDCl_3$): δ / ppm = 178.6, 165.7, 159.0, 140.3, 139.9, 90.7, 39.9, 37.4, 34.3.

IR (Diamond-ATR, neat): $\tilde{\nu}$ / cm^{-1} = 3463, 3115, 3024, 2932, 2241, 1643, 1547, 1520, 1474, 1427, 1414, 1362, 1315, 1246, 1197, 1145, 1101, 14061, 1010, 983, 919, 878, 833, 751, 727, 656, 645, 628.-

HRMS (ESI) calcd. for $C_9H_{12}N_5O_2$ $[M+H]^+$: 222.0986, found 222.0998.

2-(Dimethylamino)-*N,N*-dimethyl-2-(5-methyl-5*H*-pyrazolo[5,1-c][1,2,4]triazol-3-yl)acetamide (8b)

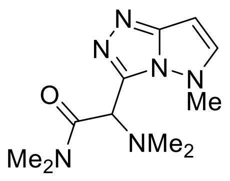

8b

8b was prepared by irradiating a solution of (*E*)-bis(*N*,*N*-dimethylamino)-((1-methyl-1*H*-pyrazol-3-yl)diazenyl)cyclopropenium tetrafluoroborate (**6s**) (32 mg, 0.10 mmol, 1.0 equiv) in water (0.01 M, 10 mL) for 15 min in a blue LED photo reactor. The reaction mixture was lyophilized, sat. $NaHCO_3$-sln. (1 mL) and brine (1 mL) were added, and the resulting mixture extracted with $CHCl_3$/*i*-PrOH (4x4 mL, 3:1, [v/v]). The combined organic layers were dried over Na_2SO_4 and concentrated under reduced pressure. Purification by flash column chromatography using (CH_2Cl_2:MeOH = 95:5, R_f = 0.30 in CH_2Cl_2:MeOH = 95:5, column equilibrated with CH_2Cl_2:NEt_3:MeOH = 93:2:5) and preparative TLC using (CH_2Cl_2:MeOH = 95:5, plate pretreated with hexane:NEt_3 = 98:2) afforded **8b** (13 mg, 0.052 mmol, 52%) as colorless oil.

^{1}H-NMR (400 MHz, $CDCl_3$): δ / ppm = 7.20 (d, *J* = 3.5 Hz, 1H), 6.18 (d, *J* = 3.5 Hz, 1H), 4.84 (s, 1H), 4.12 (s, 3H), 3.28 (s, 3H), 2.93 (s, 3H), 2.28 (s, 6H).

^{13}C-NMR (101 MHz, $CDCl_3$): δ / ppm = 167.5, 158.7, 140.8, 139.3, 90.7, 64.4, 43.7, 39.5, 38.2, 36.6.

IR (Diamond-ATR, neat): $\tilde{\nu}$ / cm^{-1} = 3414, 3111, 2929, 2783, 1650, 1558, 1524, 1488, 1397, 1250, 1192, 1135, 1102, 1058, 1033, 954, 891, 868, 847, 746, 717, 638.

HRMS (ESI) calcd. for $C_{11}H_{19}N_6O$ $[M+H]^+$: 251.1615, found 251.1621.

N,*N*-Dimethyl-2-(5-methyl-5H-[1,2,4]triazolo[4,3-b]indazol-3-yl)-2-oxoacetamide (8c)

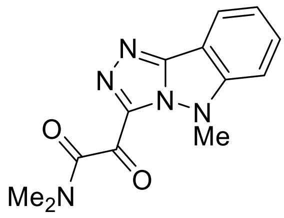

8c

8c was prepared by irradiating a solution of (*E*)-bis(*N*,*N*-dimethylamino)-((1-methyl-1*H*-indazol-3-yl)diazenyl)cyclopropenium tetrafluoroborate (**6t**) (37 mg, 0.10 mmol, 1.0 equiv) in DMSO (0.01 M, 10 mL) for 10 min in a blue LED photo reactor. Water (2 mL) was added, and the reaction mixture was lyophilized. Sat. $NaHCO_3$-sln. (1 mL) and brine (1 mL) were added, and the resulting mixture extracted with $CHCl_3$/*i*-PrOH (4x4 mL, 3:1, [v/v]). The combined organic layers were dried over Na_2SO_4 and concentrated under reduced pressure. Purification by flash column chromatography (CH_2Cl_2:MeOH = 1:0 to 98:2, R_f = 0.30 in CH_2Cl_2:MeOH = 98:2) afforded **8c** (22 mg, 0.081 mmol, 81%) as colorless solid.

^{1}H-NMR (400 MHz, $CDCl_3$): δ / ppm = 8.22 – 8.14 (m, 1H), 7.72 – 7.62 (m, 1H), 7.48 – 7.37 (m, 2H), 4.05 (s, 3H), 3.17 (s, 3H), 3.04 (s, 3H).

^{13}C-NMR (101 MHz, $CDCl_3$): δ / ppm = 179.7, 165.4, 154.5, 151.3, 141.6, 131.1, 124.3, 122.4, 111.8, 111.2, 39.6, 37.4, 34.4.

IR (Diamond-ATR, neat): $\tilde{\nu}$ / cm^{-1} = 2934, 1654, 1629, 1541, 1488, 1426, 1407, 1337, 1262, 1194, 1167, 1155, 1135, 1093, 1061, 1017, 995, 970, 919, 874, 751, 709, 700, 669, 641, 600, 548, 516, 498, 483.

HRMS (ESI) calcd. for $C_{13}H_{14}N_5O_2$ $[M+H]^+$: 272.1142, found 272.1144.

N,N-Dimethyl-2-(5-methyl-5*H*-pyrazolo[5,1-c][1,2,4]triazol-3-yl)-2-oxoacetamide (8d)

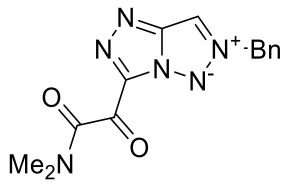

8d

8d was prepared by irradiating a solution of (*E*)-bis(*N*,*N*-dimethylamino)-((1-benzyl-1*H*-1,2,3-triazole-4-yl)diazenyl)cyclopropenium tetrafluoroborate (**6u**) (36 mg, 0.091 mmol, 1.0 equiv) in DMSO (0.01 M, 9 mL) for 15 min in a blue LED photo reactor. The reaction mixture was lyophilized, sat. $NaHCO_3$-sln. (1 mL) and brine (1 mL) were added, and the resulting mixture extracted with $CHCl_3$/*i*-PrOH (4x4 mL, 3:1, [v/v]). The combined organic layers were dried over Na_2SO_4 and concentrated under reduced pressure. Purification by flash column chromatography (CH_2Cl_2:MeOH = 98:2 to 95:5, R_f = 0.30 in CH_2Cl_2:MeOH = 95:5) afforded **8d** (17 mg, 0.057 mmol, 63%) as colorless oil.

^{1}H-NMR (500 MHz, $CDCl_3$): δ / ppm = 7.85 (s, 1H), 7.47 – 7.38 (m, 5H), 5.73 (s, 2H), 3.15 (s, 3H), 3.06 (s, 3H).

^{13}C-NMR (126 MHz, $CDCl_3$): δ / ppm = 178.1, 165.3, 153.9, 137.9, 131.9, 130.2, 129.8, 128.9, 108.4, 58.3, 37.4, 34.5.

IR (Diamond-ATR, neat): $\tilde{\nu}$ / cm^{-1} = 3154, 3115, 2927, 2855, 1648, 1498, 1473, 1456, 1428, 1405, 1335, 1275, 1258, 1205, 1159, 1102, 1062, 1016, 987, 913, 881, 818, 761, 733, 704, 673, 665, 646, 622, 606.

HRMS (ESI) calcd. for $C_{14}H_{15}N_6O_2$ $[M+H]^+$: 299.1251, found 299.1250.

Derivatization of Aryl Azocyclopropenium Salts

Cu-Catalyzed-Azide-Alkyne-Cycloaddition Product 9a – 1-(2,3-bis(diisopropylamino)cycloprop-2-en-1-ylidene)-2-(4-((1-((2*R*,3*R*,4*S*,5*R*,6*R*)-3,4,5-triacetoxy-6-(acetoxymethyl)tetrahydro-2H-pyran-2-yl)-1*H*-1,2,3-triazol-4-yl)methoxy)phenyl)diazen-1-ium tetrafluoroborate

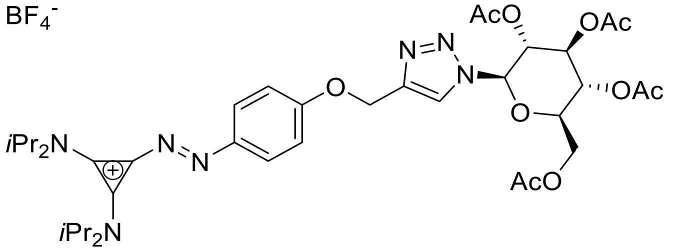

9a

A 2 mL vial equipped with a magnetic stir bar was charged with **3f** (24 mg, 0.050 mmol, 1.0 equiv), tetraacetyl-β-glucopyranosyl azide (21 mg, 0.056 mmol, 1.1 equiv), and $CuCO_4$ (0.80 mg, 5.0 µmol, 0.010 equiv). MeCN (100 µL) and a stock solution of sodium ascorbate in water (1 M, 50 µL, 0.050 mmol, 1.0 equiv) added whilst stirring at ambient temperature. Further sodium ascorbate solution was added every 30 min over a period of 2 h. After stirring for another 1 h, the reaction was diluted with $NaBF_4$-sln. (10 %, [w/w], 1 mL) and $CHCl_3$/*i*-PrOH (5 mL, 3:1, [v/v]). The phases were separated, the organic layer dried over Na_2SO_4, and concentrated under reduced pressure. Purification by flash column chromatography (SiO_2, CH_2Cl_2:MeOH = 1:0 to 96:4, R_f = 0.30 in CH_2Cl_2:MeOH = 96:4) afforded **9a** (35 mg, 0.041 mmol, 82%) as red oil.

^{1}H-NMR (500 MHz, CD_3CN): δ / ppm = 8.13 (s, 1H), 7.99 – 7.95 (m, 2H), 7.24 – 7.19 (m, 2H), 6.04 (d, *J* = 9.5 Hz, 1H), 5.62 – 5.58 (m, 1H), 5.52 – 5.46 (m, 1H), 5.34 (s, 2H), 5.27 – 5.22 (m, 1H), 4.22 – 4.10 (m, 5H), 4.04 (sept., *J* = 7.0 Hz, 1H), 2.02 (s, 3H), 2.00 (s, 3H), 1.97 (s, 3H), 1.76 (s, 3H), 1.46 (d, *J* = 7.0 Hz, 12H), 1.40 (d, *J* = 7.0 Hz, 12H).

^{13}C-NMR (126 MHz, CD_3CN): δ / ppm = 171.2, 170.8, 170.6, 169.8, 164.7, 149.0, 144.4, 128.5, 126.9, 124.2, 117.0, 111.5, 86.0, 75.5, 73.3, 71.1, 68.6, 62.9, 62.5, 59.0, 49.9, 21.6, 20.9, 20.9, 20.8, 20.7, 20.4.

^{19}F-NMR (471 MHz, CD_3CN): δ / ppm = 152.

IR (Diamond-ATR, neat): $\tilde{\nu}$ / cm^{-1} = 3531, 2982, 2940, 2260, 1875, 1751, 1576, 1497, 1461, 1375, 1350, 1300, 1209, 1138, 1033, 925, 887, 871, 843, 762, 740, 687, 638, 606, 558, 544, 520, 507, 490.

HRMS (ESI) calcd. for $[M\text{-}BF_4]^+$: 768.3927, found 768.3904.

[a]$_D^{25}$ = –35.4 (c = 0.1, $CHCl_3$).

Peptide Coupling Product 9b – *N*-(2-(diisopropylamino)-3-((*E*)-(4-(((*S*)-1-methoxy-1-oxo-3-phenylpropan-2-yl)carbamoyl)phenyl)diazenyl) cyclopropylidene)-*N*-isopropylpropan-2-aminium tetrafluoroborate

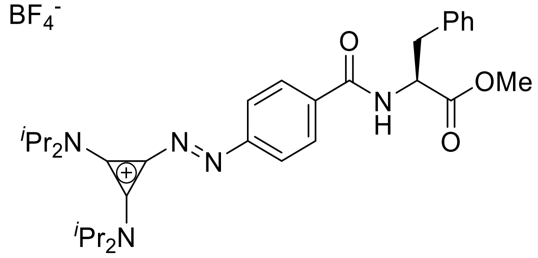

9b

A 4 mL vial equipped with a magnetic stir bar was charged with **3f** (20 mg, 0.042 mmol, 1.0 equiv) and *L*-phenyl alanine hydrochloride (14 mg, 0.065 mmol, 1.5 equiv) in MeCN (200 µL). Triethylamine (30 µL, 0.22 mmol, 5.1 equiv) and T3P (50µL, 50% in EtOAc, 0.085 mmol, 2.0 equiv) were added subsequently and the reaction stirred at ambient temperature. After 1.5 h, the reaction mixture was diluted with $CHCl_3$/*i*-PrOH (4 mL, 3:1, [v/v]), washed with aq. HBF_4-sln. (4 mL, 5%, [w/w]), dried over Na_2SO_4 and concentrated under reduced pressure. Purification by flash column chromatography (SiO_2, CH_2Cl_2:MeOH = 1:0 to 95:5, R_f = 0.35 in CH_2Cl_2:MeOH = 96:4) afforded **9b** (24 mg, 0.038 mmol, 89 %) as red oil.

^{1}H-NMR (500 MHz, CD_3CN): δ / ppm = 8.01 – 7.97 (m, 2H), 7.94 – 7.90 (m, 2H), 7.43 (d, *J* = 8.0 Hz, 1H), 7.33 – 7.26 (m, 4H), 7.26 – 7.21 (m, 1H), 4.87 (ddd, *J* = 9.0, 8.0, 5.5 Hz, 1H), 4.18 (sept., *J* = 6.5 Hz, 2H), 4.09 (sept., *J* = 7.0 Hz, 2H), 3.29 (dd, *J* = 14.0, 5.0 Hz, 1H), 3.13 (dd, *J* = 14.0, 9.0 Hz, 1H), 1.47 (d, *J* = 7.0 Hz, 12H), 1.42 (d, *J* = 6.5 Hz, 12H).

^{13}C-NMR (126 MHz, CD_3CN): δ / ppm = 172.8, 166.7, 155.5, 139.3, 138.2, 130.3, 129.7, 129.5, 129.4, 127.8, 124.3, 110.8, 59.5, 55.4, 52.9, 50.3, 37.9, 21.5, 20.6.

^{19}F-NMR (471 MHz, CD_3CN): δ / ppm = 152.

IR (Diamond-ATR, neat): $\tilde{\nu}$ / cm^{-1} = 3373, 2984, 2939, 2880, 1870, 1744, 1699, 1662, 1585, 1534, 1491, 1456, 1393, 1376, 1349, 1323, 1300, 1207, 1182, 1161, 1139, 1052, 1034, 1018, 955, 899, 861, 828, 773, 763, 747, 702, 657, 608, 559, 520, 495.

HRMS (ESI) calcd. for $C_{32}H_{44}N_5O_3$ $[M-BF_4]^+$: 546.3439, found 546.3434.

$[a]_D^{25}$ = +78.8 (c = 0.1, $CHCl_3$).

(2-(Dimethylamino)-3-oxocycloprop-1-en-1-yl)proline (10)

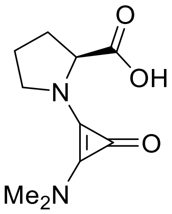

10

A 10 mL round bottom flask equipped with a magnetic stir bar was charged with chloro-bis(dimethylamino)cyclopropenium tetrafluoroborate (**1b**) (490 mg, 2.0 mmol, 1.0 equiv) and $NaHCO_3$ (340 mg, 4.0 mmol, 2.0 equiv). Water (0.4 mL) was added, followed by MeCN (2 mL). L-Proline *tert*-butyl ester (380 mg, 2.1 mmol, 1.1 equiv) was dissolved in MeCN (2 mL) and added dropwise to the reaction mixture. After stirring at ambient temperature for 1 h, the resulting suspension was filtered over celite and concentrated under reduced pressure. The obtained oil was treated with KOH (5 mL, 2 M) at 70°C for 3 h. After cooling to ambient temperature, the reaction was transferred with water (5 mL) and washed with CH_2Cl_2 (2x5 mL). The aqueous layer was cooled to 0°C and carefully acidified by addition of conc. HCl solution (1 mL, 37%). The aqueous layer was extracted with $CHCl_3$/*i*-PrOH (4x10 mL, 3:1, [v/v]). The organic layers were dried over Na_2SO_4 and concentrated under reduced pressure. Purification by flash column chromatography (SiO_2, CH_2Cl_2/MeOH = 98:2 to 93:7, R_f = 0.3 in CH_2Cl_2/MeOH = 93:7) afforded **10** (290 mg, 1.4 mmol, 70%) as colorless oil.

^{1}H-NMR (400 MHz, CD_3CN): δ / ppm = 4.34 (dd, *J* = 8.5, 4.0 Hz), 3.58 – 3.51 (m, 1H), 3.51 – 3.43 (m, 1H), 2.97 (s, 6H), 2.31 – 2.20 (m, 1H), 2.12 – 2.04 (m, 1H), 1.97 – 1.85 (m, 2H).

^{13}C-NMR (101 MHz, CD_3CN): δ / ppm = 175.1, 131.5, 121.4, 118.7, 63.0, 52.0, 41.2, 31.3, 24.9.

IR (Diamond-ATR, neat): $\tilde{\nu}$ / cm^{-1} = 2937, 2879, 2807, 2500, 1908, 1721, 1646, 1536, 1499, 1456, 1406, 1350, 1223, 1175, 1104, 1085, 1060, 1031, 975, 911, 868, 831, 785, 740, 702, 623, 583, 509, 485.

HRMS (ESI) calcd. for $C_{10}H_{15}N_2O_3$ $[M+H]^+$: 211.1077, found 211.1075.

[a]$_D^{27}$ = –96.6 (c = 0.5, MeOH).

1-chloro-2-(dimethylamino)-3-((*S*)-2-(ethoxycarbonyl)pyrrolidin-1-yl)cyclopropenium tetrafluoroborate (1c)

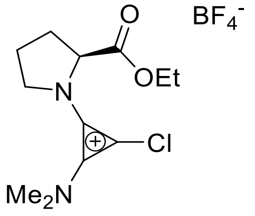

1c

A 10 mL flask equipped with a magnetic stir bar was charged with (2-(dimethylamino)-3-oxocycloprop-1-en-1-yl)proline (**10**) (230 mg, 1.1 mmol, 1.0 equiv) in dry CH_2Cl_2 (4.4 mL) and cooled to 0 °C. Oxalyl chloride (280 µL, 420 mg, 3.3 mmol, 3.0 equiv) was added dropwise while stirring. After the gas evolution had ceased, DMF (1 drop from a glass Pasteur pipet) was added, and the cooling removed after 10 min. After stirring for another 1.5 h, the reaction was concentrated under reduced pressure. The flask was cooled to 0 °C, EtOH (2 mL) added, and the solution stirred for 3 h. The reaction mixture was concentrated under reduced pressure and the resulting oil dissolved in $CHCl_3$/^{i}PrOH (6 mL, 3:1, [v/v]). The solution was washed with $NaBF_4$-sln. (3 mL, 10%, [w/w]) and the aq. phase extracted with $CHCl_3$/^{i}PrOH (2x4 mL, 3:1, [v/v]). The combined org. phases were dried over Na_2SO_4 and concentrated under reduced pressure. Purification by flash column chromatography (SiO_2, CH_2Cl_2:MeOH = 1:0 to 95:5, R_f = 0.20 in CH_2Cl_2:MeOH = 98:2) afforded **1c** (330 mg, 0.96 mmol, 87%) as colorless oil.

*Note: The ^{1}H NMR spectrum of **1c** showed a mixture of two rotamers (2:1 in CD_3CN). Signals corresponding to the major rotamer.*

^{1}H-NMR (500 MHz, CD_3CN): δ / ppm = 4.64 – 4.59 (m, 1H), 4.25 – 4.18 (m, 2H), 3.88 – 3.79 (m, 1H), 3.78 – 3.72 (m, 1H), 3.21 (s, 3H), 3.17 (s, 3H), 2.41 – 2.22 (m, 2H), 2.11 – 1.95 (m, 2H), 1.27 (t, *J* = 7.0 Hz, 3H).

^{13}C-NMR (126 MHz, CD_3CN): δ / ppm = 170.8, 135.5, 133.0, 94.1, 63.7, 63.2, 53.7, 42.5, 42.0, 30.6, 25.2, 14.4.

Note: Signals corresponding to the minor rotamer.

^{1}H-NMR (500 MHz, CD_3CN): δ / ppm = 4.64 – 4.59 (m, 1H), 4.25 – 4.18 (m, 2H), 3.88 – 3.79 (m, 1H), 3.78 – 3.72 (m, 1H), 3.21 (s, 3H), 3.03 (s, 3H), 2.41 – 2.22 (m, 2H), 2.11 – 1.95 (m, 2H), 1.25 (t, *J* = 7.0 Hz, 3H).

^{13}C-NMR (126 MHz, CD_3CN): δ / ppm = 171.1, 135.4, 133.0, 94.2, 64.2, 63.5, 52.8, 42.3, 42.2, 30.5, 25.0, 14.3.

^{19}F-NMR (471 MHz, CD_3CN): δ / ppm = -152.

IR (Diamond-ATR, neat): $\tilde{\nu}$ / cm^{-1} = 2985, 2949, 1949, 1738, 1616, 1509, 1435, 1389, 1347, 1320, 1284, 1240, 1219, 1187, 1156, 1050, 1035, 919, 857, 796, 767, 733, 646, 521, 498, 483.

HRMS (ESI) calcd. for $C_{12}H_{18}ClN_2O_2$ $[M-BF_4]^+$: 257.1051, found 257.1048.

[a]$_D^{27}$ = –119.4 (c = 0.1, $CHCl_3$).

E)-1-*N*,*N*-dimethylamino-2-((*S*)-2-(ethoxycarbonyl)pyrrolidin-1-yl)-3-((4-methoxyphenyl)diazenyl) cyclopropenium tetrafluoroborate (11a)

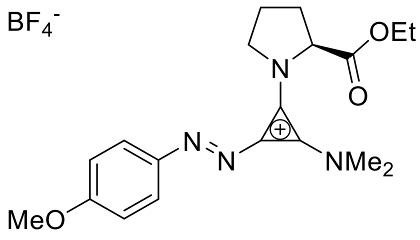

11a

11a was prepared according to **GP2.3** using tert-butyl *N*-Boc-*N*-(4-methoxyphenyl)hydrazide (**SI-4c**) (30 mg, 0.13 mmol, 1.0 equiv), chlorocyclopropenium tetrafluoroborate **1c** (52 mg, 1.5 mmol, 1.2 equiv), and pyridine (12 µL, 1.5 mmol, 1.2 equiv) in dry $CHCl_3$ (0.5 mL, 0.25 M). The reaction was heated at 50 °C for 1 h. Benzoquinone (27 mg, 0.25 mmol, 2.0 equiv) was added and the reaction worked up as described in **GP2.2**. Purification by flash column chromatography (SiO_2, EtOAc, then CH_2Cl_2:MeOH = 1:0 to 95:5, Rf = 0.30 in CH_2Cl_2:MeOH = 97:3) afforded **11a** (44 mg, 0.099 mmol, 79 %) as red oil.

*Note: The ^1H NMR spectrum of **11a** showed a mixture of two rotamers (2:1 in CD_3CN). Signals corresponding to the major rotamer.*

^{1}H-NMR (500 MHz, CD_3CN): δ / ppm = 7.90 – 7.86 (m, 2H), 7.18 – 7.13 (m, 2H), 4.81 – 4.76 (ddd, *J* = 8.5, 5.0, 0.5 Hz, 1H), 4.19 – 4.07 (m, 2H), 3.97 – 3.90 (m, 1H), 3.92 (s, 3H), 3.90 – 3.84 (m, 1H), 3.45 (s, 3H), 3.30 (s, 3H), 2.51 – 2.40 (m, 1H), 2.29 – 2.21 (m, 1H), 2.16 – 2.01 (m, 2H), 1.19 (t, *J* = 7.0 Hz, 3H).

^{13}C-NMR (126 MHz, CD_3CN): δ / ppm = 171.0, 166.7, 148.8, 131.1, 127.9, 127.4, 116.3, 112.9, 65.8, 63.0, 57.0, 53.7, 44.1, 42.9, 30.9, 25.4, 14.4.

Note: Signals corresponding to the minor rotamer.

^{1}H-NMR (500 MHz, CD_3CN): δ / ppm = 7.98 – 7.94 (m, 2H), 7.19 – 7.16 (m, 2H), 4.73 (m, 1H), 4.25 (q, *J* = 7.0 Hz, 2H), 4.06 – 4.00 (m, 1H), 3.99 – 3.91 (m, 1H), 3.93 (s, 3H), 3.45 (s, 3H), 3.15 (s, 3H), 2.51 – 2.40 (m, 1H), 2.40 – 2.33 (m, 1H), 2.16 – 2.01 (m, 2H), 1.27 (t, *J* = 7.0 Hz, 3H).

^{13}C-NMR (126 MHz, CD_3CN): δ / ppm = 171.2, 166.7, 148.9, 131.0, 128.1, 127.4, 116.3, 112.8, 64.4, 63.5, 57.0, 55.0, 44.7, 42.5, 30.8, 25.0, 14.4.

^{19}F-NMR (471 MHz, CD_3CN): δ / ppm = -152.

IR (Diamond-ATR, neat): $\tilde{\nu}$ / cm^{-1} = 2983, 2946, 2847, 1914, 1738, 1611, 1595, 1575, 1502, 1457, 1424, 1357, 1315, 1300, 1257, 1220, 1185, 1144, 1048, 1033, 1013, 964, 917, 846, 798, 764, 742, 729, 706, 634, 612, 589, 520, 499.

HRMS (ESI) calcd. for $C_{19}H_{25}N_4O_3$ $[M\text{-}BF_4]^+$: 357.1921, found 357.1913.

[a]$_D^{27}$ = –8.2 (c = 0.1, $CHCl_3$).

(*E*)-1-*N,N*-dimethylamino)-2-((*S*)-2-(ethoxycarbonyl)pyrrolidin-1-yl)-3-((1-methyl-1*H*-pyrazol-4-yl)diazenyl)cyclopropenium tetrafluoroborate (11b)

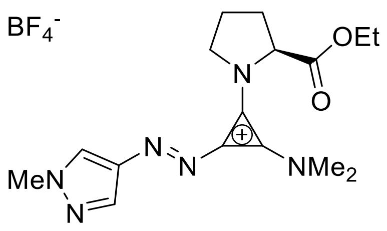

11b

10b was prepared according to **GP2.3** using tert-butyl 1-(1-methyl-1*H*-pyrazol-4-yl)hydrazine-1-carboxylate (**SI-4o**) (27 mg, 0.13 mmol, 1.0 equiv), chlorocyclopropenium tetrafluoroborate **1c** (52 mg, 1.5 mmol, 1.2 equiv), and pyridine (12 µL, 1.5 mmol, 1.2 equiv) in dry $CHCl_3$ (0.5 mL, 0.25 M). The reaction was heated at 50 °C for 1 h. Benzoquinone (27 mg, 0.25 mmol, 2.0 equiv) was added and the reaction worked up as described in **GP2.2**. Purification by flash column chromatography (SiO_2, EtOAc, then CH_2Cl_2:MeOH = 1:0 to 95:5, R_f = 0.20 in CH_2Cl_2:MeOH = 97:3) afforded **11b** (28 mg, 0.067 mmol, 54%) as orange solid.

*Note: The ^{1}H NMR spectrum of **11b** showed a mixture of two rotamers (2:1 in CD_3CN). Signals corresponding to the major rotamer.*

^{1}H-NMR (400 MHz, CD_3CN): δ / ppm = 8.22 (s, 1H), 7.92 (s, 1H), 4.74 – 4.67 (m, 1H), 4.22 – 4.09 (m, 2H), 3.93 (s, 3H), 3.97 – 3.80 (m, 2H), 3.40 (s, 3H), 3.27 (s, 3H), 2.50 – 2.30 (m, 2H), 2.14 – 1.99 (m, 2H), 1.20 (t, *J* = 7.0 Hz, 3H).

^{13}C-NMR (101 MHz, CD_3CN): δ / ppm = 171.0, 144.1, 133.9, 132.0, 130.8, 127.7, 113.3, 65.6, 63.0, 53.7, 44.0, 42.7, 40.4, 30.9, 25.4, 14.5.

Note: Signals corresponding to the minor rotamer.

^{1}H-NMR (400 MHz, CD_3CN): δ / ppm = 8.30 (s, 1H), 7.98 (s, 1H), 4.74 – 4.67 (m, 1H), 4.24 (q, *J* = 7.0 Hz, 2H), 4.02 – 3.91 (m, 2H), 3.94 (s, 3H), 3.40 (s, 3H), 3.12 (s, 3H), 2.28 – 2.19 (m, 2H), 2.14 – 1.99 (m, 2H), 1.27 (t, *J* = 7.0 Hz, 3H).

^{13}C-NMR (101 MHz, CD_3CN): δ / ppm = 171.2, 144.2, 133.9, 132.1, 130.9, 127.8, 113.3, 64.3, 63.5, 54.8, 44.6, 42.4, 40.4, 30.8, 25.0, 14.3.

^{19}F-NMR (377 MHz, CD_3CN): δ / ppm = -152.

IR (Diamond-ATR, neat): $\tilde{\nu}$ / cm^{-1} = 3128, 2984, 2947, 2815, 1916, 1737, 1610, 1535, 1450, 1426, 1377, 1351, 1272, 1217, 1189, 1158, 1052, 1035, 1011, 918, 872, 831, 764, 747, 736, 718, 666, 626, 580, 542, 520, 499.

HRMS (ESI) calcd. for $C_{16}H_{23}N_6O_2$ $[M-BF_4]^+$: 331.1877, found 331.1867.

[a]$_D^{27}$ = –38.8 (c = 0.1, $CHCl_3$).

Single Crystal X-Ray Data

Single Crystal X-Ray Data

Single crystals of compound **3a**, **3d**, **6n**, **6t**, **8a**, and **10b** were obtained through slow evaporation of $CDCl_3$ (**3d**), MeCN (**6n**, **6t**, **10b**), or EtOH (**8a**).

Single crystalline samples were measured on a Rigaku Oxford Diffraction XtaLAB Synergy-S Dualflex kappa diffractometer equipped with a Dectris Pilatus 300 HPAD detector and using microfocus sealed tube Cu-Kα or Mo-Kα radiation with mirror optics (λ = 1.54178 Å, **3a**, **3d**, **6n**, **6t**) or a Rigaku Oxford Diffraction XtaLAB Synergy-R kappa diffractometer equipped with a Rigaku HyPix Arc150 HPAD detector and using microfocus rotating anode Cu-Kα radiation with mirror optics (λ = 1.54178 Å, **8a**, **10b**).

All measurements were carried out at 100K (unless specifically noted) using an Oxford Cryosystems Cryostream 800 or 1000 sample cryostat. Data collected on the Rigaku instrument were integrated using CrysAlisPro and corrected for absorption effects using a combination of empirical (ABSPACK) and numerical corrections.[30] The structures were solved using SHELXT[31] and refined by full-matrix least-squares analysis (SHELXL)[32,33], using the program package OLEX2.[34] Unless otherwise indicated below, all non-hydrogen atoms were refined anisotropically and hydrogen atoms were constrained to ideal geometries and refined with fixed isotropic displacement parameters (in terms of a riding model).

CCDC 2327727 (**3a**), CCDC 2327724 (**3d**), CCDC 2327725 (**6m**), CCDC 2327728 (**6t**), CCDC 2327723 (**8a**), and CCDC 2327726 (**10b**) and contain the supplementary crystallographic data for this paper including structure factors and refinement instructions. These data can be obtained free of charge from The Cambridge Crystallographic Data Centre, 12 Union Road, Cambridge CB2 1EZ, UK (fax: +44(1223)-336-033; e-mail: deposit@ccdc.cam.ac.uk), or via https://www.ccdc.cam.ac.uk/getstructures.

Crystal Structure Data of 3a

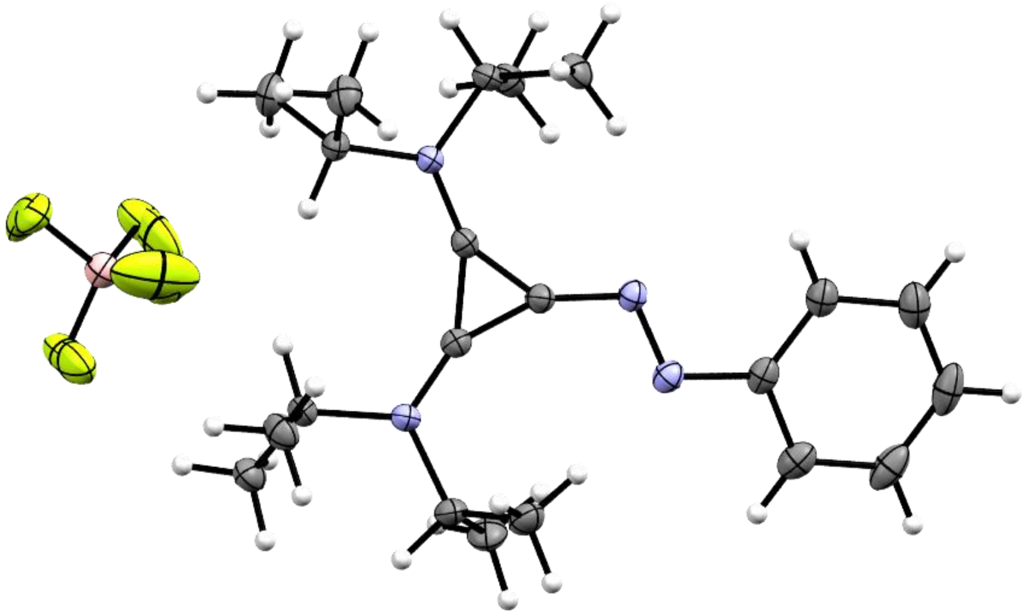

Figure S3: ORTEP diagram of **3a**. Thermal ellipsoids are displayed at 50% probability level. Hydrogen atoms are displayed as spheres with fixed radius (0.15 Å).

CCDC number	2327727
Empirical formula	$C_{21}H_{33}BF_4N_4$
Formula weight	428.32
Temperature [K]	100.0(1)
Crystal system	orthorhombic
Space group (number)	$Pbca$ (61)
a [Å]	15.63120(10)
b [Å]	13.92580(10)
c [Å]	21.14100(10)
α [°]	90
β [°]	90
γ [°]	90

Volume [Å^3]	4601.91(5)
Z	8
ρ_{calc} [gcm^{-3}]	1.236
μ [mm^{-1}]	0.806
F(000)	1824
Crystal size [mm^3]	0.22×0.145×0.071
Crystal colour	clear orange
Crystal shape	block
Radiation	Cu K_α (λ=1.54184 Å)
2Θ range [°]	8.36 to 160.00 (0.78 Å)
Index ranges	-19 ≤ h ≤ 19<br>-16 ≤ k ≤ 17<br>-26 ≤ l ≤ 26
Reflections collected	114388
Independent reflections	5005<br>R_{int} = 0.0510<br>R_{sigma} = 0.0144
Completeness	100.0 %
Data / Restraints / Parameters	5005/226/316
Goodness-of-fit on F^2	1.068
Final R indexes<br>[I≥2σ(I)]	R_1 = 0.0334<br>wR_2 = 0.0832
Final R indexes<br>[all data]	R_1 = 0.0360<br>wR_2 = 0.0849
Largest peak/hole [eÅ^{-3}]	0.22/-0.19

Crystal Structure Data of 3d

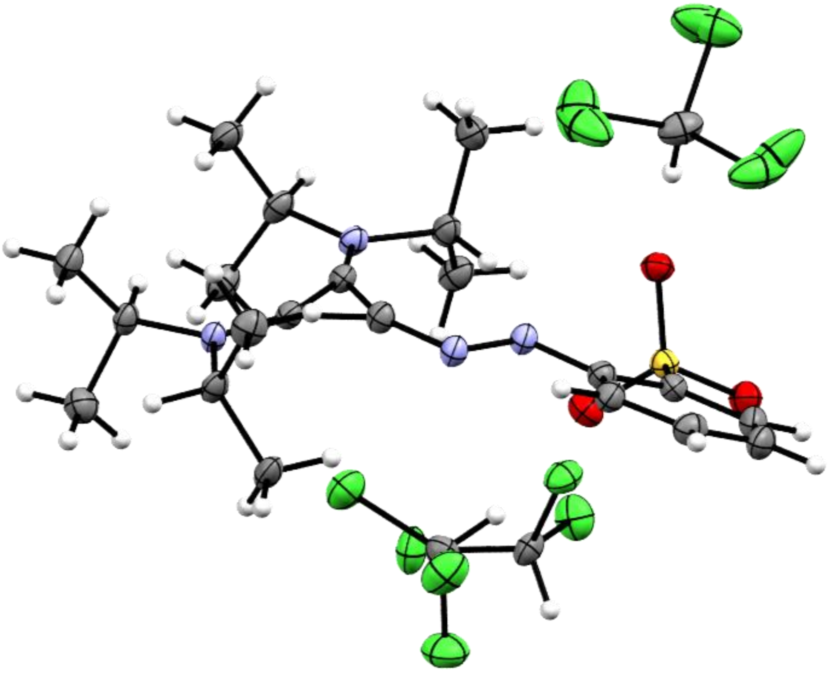

Figure S4: ORTEP diagram of **3d**. Thermal ellipsoids are displayed at 50% probability level. Hydrogen atoms are displayed as spheres with fixed radius (0.15 Å).

CCDC number	2327724
Empirical formula	$C_{24}H_{35}Cl_9N_4O_3S$
Formula weight	778.67
Temperature [K]	100.0(1)
Crystal system	triclinic
Space group (number)	$P\overline{1}$ (2)
a [Å]	9.84550(10)
b [Å]	11.4499(2)
c [Å]	16.7063(3)
α [°]	103.533(2)
β [°]	90.2130(10)
γ [°]	98.325(2)
Volume [Å^3]	1810.33(5)
Z	2
ρ_{calc} [gcm^{-3}]	1.428

μ [mm^{-1}]	7.172
$F(000)$	800
Crystal size [mm^3]	0.206×0.149×0.073
Crystal colour	clear orange
Crystal shape	block
Radiation	Cu K_α (λ=1.54184 Å)
2Θ range [°]	5.45 to 160.00 (0.78 Å)
Index ranges	$-10 \le h \le 11$ $-14 \le k \le 14$ $-21 \le l \le 21$
Reflections collected	40327
Independent reflections	7601 R_{int} = 0.0580 R_{sigma} = 0.0370
Completeness	99.8 %
Data / Restraints / Parameters	7601/114/406
Goodness-of-fit on F^2	1.050
Final R indexes [$I \geq 2\sigma(I)$]	R_1 = 0.0523 wR_2 = 0.1343
Final R indexes [all data]	R_1 = 0.0587 wR_2 = 0.1379
Largest peak/hole [eÅ^{-3}]	0.84/-0.53

Crystal Structure Data of 6m

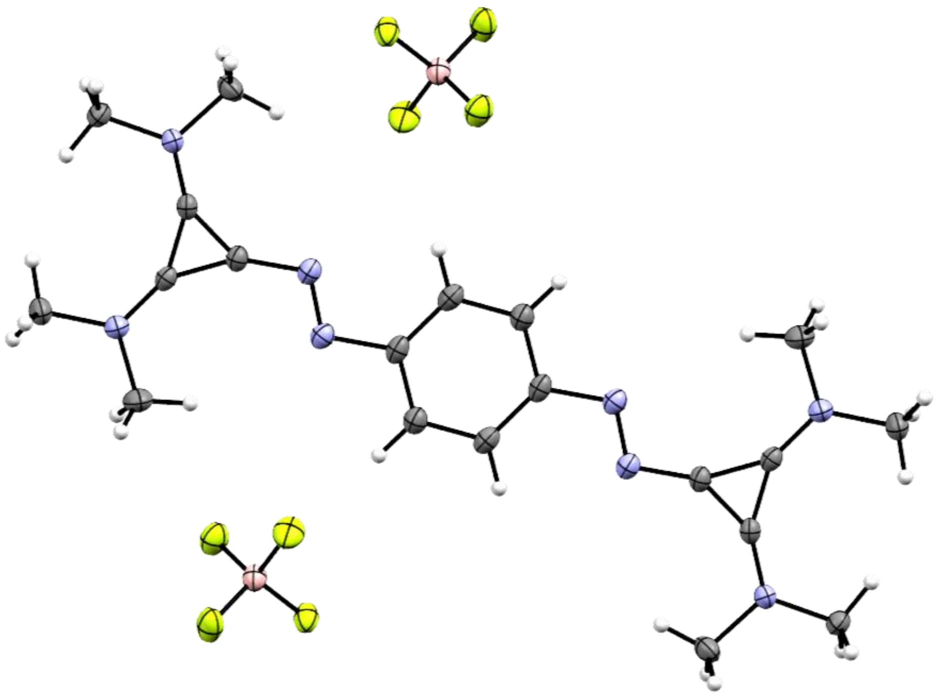

Figure S5: ORTEP diagram of **6m**. Thermal ellipsoids are displayed at 50% probability level. Hydrogen atoms are displayed as spheres with fixed radius (0.15 Å).

CCDC number	2327725
Empirical formula	$C_{20}H_{28}B_2F_8N_8$
Formula weight	554.12
Temperature [K]	100.0(1)
Crystal system	monoclinic
Space group (number)	$P2_1/c$ (14)
a [Å]	6.29000(10)
b [Å]	16.3995(2)
c [Å]	12.8681(2)
α [°]	90
β [°]	101.6230(10)
γ [°]	90
Volume [Å^3]	1300.16(3)
Z	2
ρ_{calc} [gcm^{-3}]	1.415

μ [mm^{-1}]	1.118
F(000)	572
Crystal size [mm^3]	0.28×0.163×0.044
Crystal colour	clear dark red
Crystal shape	plate
Radiation	Cu K_α (λ=1.54184 Å)
2Θ range [°]	8.85 to 159.24<br>(0.78 Å)
Index ranges	-7 ≤ h ≤ 7<br>-20 ≤ k ≤ 20<br>-16 ≤ l ≤ 16
Reflections collected	39830
Independent reflections	2802<br>R_{int} = 0.0471<br>R_{sigma} = 0.0175
Completeness	100.0 %
Data / Restraints / Parameters	2802/0/176
Goodness-of-fit on F^2	1.058
Final R indexes<br>[I≥2σ(I)]	R_1 = 0.0373<br>wR_2 = 0.1006
Final R indexes<br>[all data]	R_1 = 0.0398<br>wR_2 = 0.1028
Largest peak/hole [eÅ^{-3}]	0.32/-0.21

Crystal Structure Data of 6q

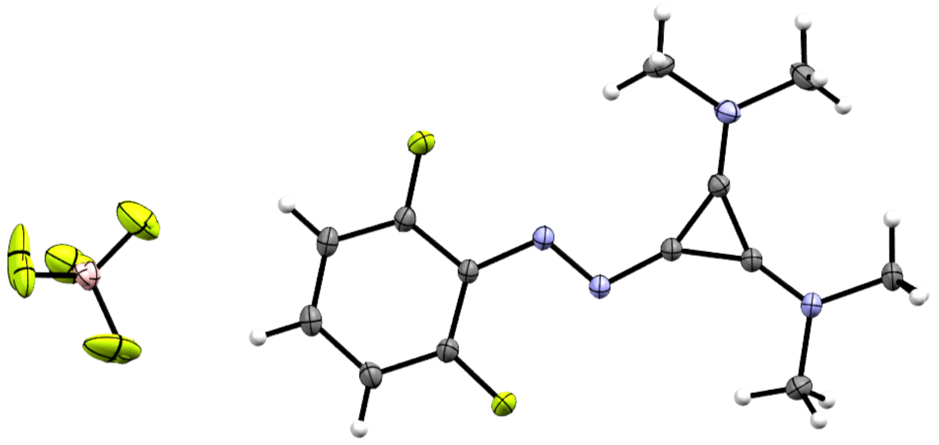

Figure S6: ORTEP diagram of **6q**. Thermal ellipsoids are displayed at 50% probability level. Hydrogen atoms are displayed as spheres with fixed radius (0.15 Å).

CCDC number	2327728
Empirical formula	$C_{13}H_{15}BF_6N_4$
Formula weight	352.10
Temperature [K]	100.0(1)
Crystal system	monoclinic
Space group (number)	$I2/a$ (15)
a [Å]	13.8039(2)
b [Å]	13.06360(10)
c [Å]	17.7630(2)
α [°]	90
β [°]	103.7420(10)
γ [°]	90
Volume [Å^3]	3111.49(6)
Z	8

ρ_{calc} [gcm^{-3}]	1.503
μ [mm^{-1}]	0.142
$F(000)$	1440
Crystal size [mm^3]	0.35×0.275×0.189
Crystal colour	clear orange
Crystal shape	block
Radiation	Mo K_{α} (λ=0.71073 Å)
2Θ range [°]	4.35 to 77.64 (0.57 Å)
Index ranges	$-24 \leq h \leq 24$<br>$-22 \leq k \leq 23$<br>$-31 \leq l \leq 30$
Reflections collected	182851
Independent reflections	8763<br>R_{int} = 0.0326<br>R_{sigma} = 0.0106
Completeness	100.0 %
Data / Restraints / Parameters	8763/280/267
Goodness-of-fit on F^2	1.035
Final R indexes [$I \geq 2\sigma(I)$]	R_1 = 0.0376<br>wR_2 = 0.1134
Final R indexes [all data]	R_1 = 0.0439<br>wR_2 = 0.1171
Largest peak/hole [eÅ^{-3}]	0.56/-0.31

Crystal Structure Data of 8a

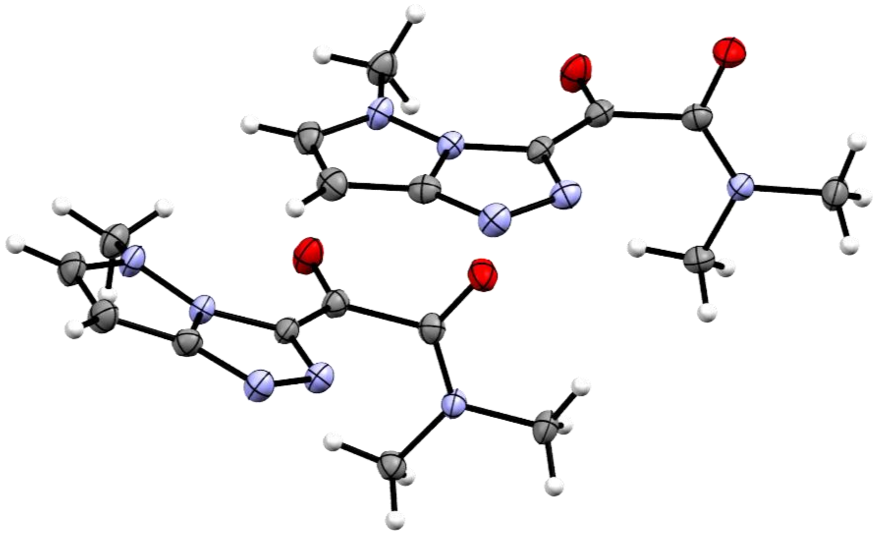

Figure S7: ORTEP diagram of **8a**. Thermal ellipsoids are displayed at 50% probability level. Hydrogen atoms are displayed as spheres with fixed radius (0.15 Å).

CCDC number	2327723
Empirical formula	$C_9H_{11}N_5O_2$
Formula weight	221.23
Temperature [K]	100.0(1)
Crystal system	monoclinic
Space group (number)	$P2_1/c$ (14)
a [Å]	6.29580(10)
b [Å]	17.2571(4)
c [Å]	18.5933(3)
α [°]	90
β [°]	94.105(2)
γ [°]	90
Volume [Å^3]	2014.93(7)
Z	8
ρ_{calc} [gcm^{-3}]	1.459

μ [mm^{-1}]	0.909
F(000)	928
Crystal size [mm^3]	0.162×0.043×0.019
Crystal colour	clear colourless
Crystal shape	needle
Radiation	Cu K_α (λ=1.54184 Å)
2Θ range [°]	7.00 to 149.94 (0.80 Å)
Index ranges	$-7 \le h \le 7$<br>$-20 \le k \le 20$<br>$-23 \le l \le 23$
Reflections collected	7113
Independent reflections	7113<br>R_{int} = 0.00370<br>R_{sigma} = 0.0262
Completeness	99.5 %
Data / Restraints / Parameters	7113/0/296
Goodness-of-fit on F^2	1.036
Final R indexes [I≥2σ(I)]	R_1 = 0.0383<br>wR_2 = 0.0890
Final R indexes [all data]	R_1 = 0.0530<br>wR_2 = 0.0953
Largest peak/hole [eÅ^{-3}]	0.19/-0.22

Crystal Structure Data of 10b

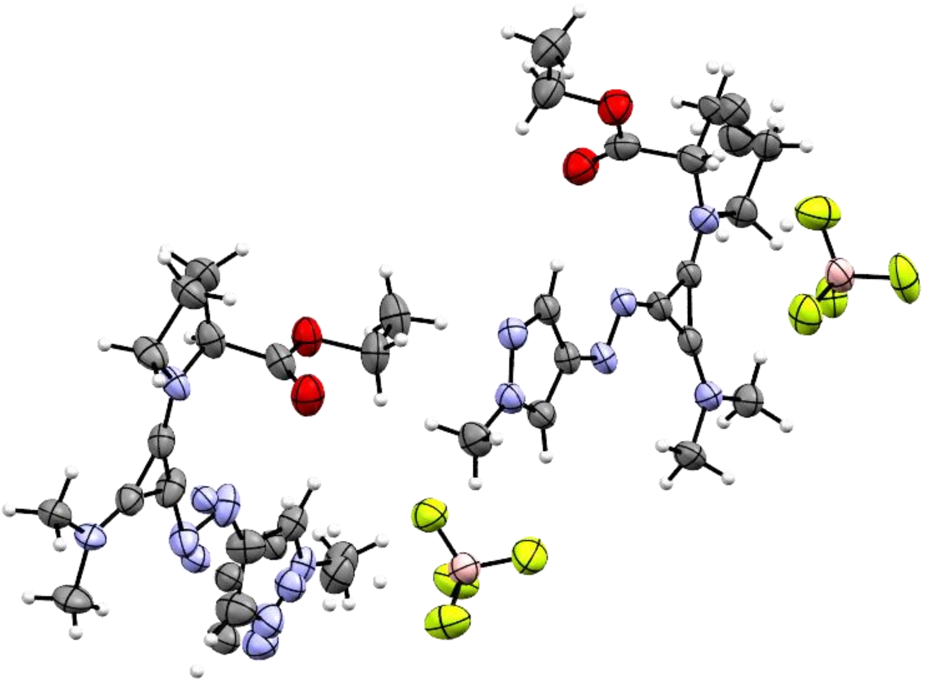

Figure S8: ORTEP diagram of **10b**. Thermal ellipsoids are displayed at 50% probability level. Hydrogen atoms are displayed as spheres with fixed radius (0.15 Å).

CCDC number	2327726
Empirical formula	$C_{16}H_{23}BF_4N_6O_2$
Formula weight	418.21
Temperature [K]	100.0(1)
Crystal system	orthorhombic
Space group (number)	$P2_12_12_1$ (19)
a [Å]	6.09470(10)
b [Å]	18.5713(4)
c [Å]	35.9886(5)
α [°]	90
β [°]	90
γ [°]	90
Volume [Å^3]	4073.42(12)
Z	8
ρ_{calc} [gcm^{-3}]	1.364
μ [mm^{-1}]	1.008

$F(000)$	1744
Crystal size [mm^3]	0.143×0.029×0.029
Crystal colour	clear yellow
Crystal shape	needle
Radiation	Cu K_α (λ=1.54184 Å)
2Θ range [°]	4.91 to 146.43<br>(0.81 Å)
Index ranges	-7 ≤ h ≤ 7<br>-22 ≤ k ≤ 22<br>-38 ≤ l ≤ 43
Reflections collected	40718
Independent reflections	7540<br>R_{int} = 0.0612<br>R_{sigma} = 0.0476
Completeness	99.3 %
Data / Restraints / Parameters	7540/515/618
Goodness-of-fit on F^2	1.059
Final R indexes<br>[$I \geq 2\sigma(I)$]	R_1 = 0.0568<br>wR_2 = 0.1529
Final R indexes<br>[all data]	R_1 = 0.0782<br>wR_2 = 0.1655
Largest peak/hole [eÅ^{-3}]	0.38/-0.21
Flack X parameter	-0.05(8)

Determination of Photo-physical Data

PSS UV/Vis-Spectra. Approximately 2.00 mg of compound were accurately weighted into a 20 mL amber glass vial and dissolved in 10.0 mL of either DMSO (Sigma Aldrich, ReagentPlus® ≥99.5%), MeCN (Sigma Aldrich, for HPLC, gradient grade ≥99.9%), or distilled water, respectively. In case water was used, sonification was applied in some cases to make sure that the compound had completely dissolved. The stock solution was diluted to a concentration of 100 µM in a quartz cuvette (V = 1 mL, d = 1 cm) and the UV/Vis spectrum of the dark adapted state recorded on a Mettler Toledo UV5Bio spectrometer (measurement time 1 s). The cuvette was removed from the spectrometer and the sample irradiated at 340 nm, 365 nm, 385 nm, 415 nm, 455 nm, 505 nm, and 530 nm, respectively, in ascending order. After 30 min (340 nm) or 20 min (all other wavelengths) of irradiation, the UV/Vis spectrum was recorded immediately. Plotting and overlay of all UV/Vis spectra with an in-house written MATLAB R2020b script allowed determination of the absorption maxima and the isosbestic wavelengths from the intersections of the absorption spectra at the photostationary states (PSSs).

PSS Composition. *E*- and *Z*-isomers of aryl azocyclopropenium salt were separated by analytical HPLC to quantify PSS compositions. Therefore, 1 mL of a 100 µM solution of the respective compounds was irradiated in a 2 mL amber glass GC vial at the respective wavelength for 20 min. The vial was properly sealed with a septum cap and submitted to an HPLC machine (Waters e2965 separation module equipped with a 2998 PDA detector). 10 µL of the solution were injected onto a Reproshper 100 C12 column (5 µm, 125x4.6 mm), eluting with MeCN/water (3:7) containing 0.1% formic acid, unless stated otherwise. The delay between the end of irradiation and injection was in the order of 1 min.

Upon successful separation of the isomers, the chromatograms were extracted at both isosbestic wavelengths in water and integrated to determine the *E*/*Z*-ratio. (*Comment:* as the deviation between the isosbestic wavelengths in MeCN and water is commonly below 2 nm for the compounds investigated, the chromatograms were evaluated at the isosbestic wavelengths in water, as it was believed to better represent the eluent mixture.) All PSS contents are reported as average of the relative integrals obtained for both isosbestic points and compiled in Tables **T1** and **T2** (see end of SI for HPLC chromatograms). The absolute deviation of the PSS composition between the values obtained for two different isosbestic wavelengths is in the order of 1%, averaged over all compounds investigated in this study.

Thermal Half-lives. For determination of thermal half-lives, 100 µM solutions of the respective compounds were prepared as described above in a quartz cuvette (V = 1 mL, d = 1 cm). The samples were irradiated at the wavelength that gave the highest *Z*-isomer content in the PSS. Afterwards, the cuvettes were placed on a Mettler Toledo UV5Bio spectrometer, equipped with a CuveT thermostat, and the time course of absorption increase recorded at the absorption maximum of the *E*-isomer. The obtained data points were plotted and fitted with an in-house written MATLAB R2020b script, assuming a first order rate law:

$$A_t = -Ae^{-kt} + C$$

A_t – change in absorbance, k – rate constant, t – time, A – scaling factor, C – scaling constant.

The thermal half-lives were calculated as:

$$t_{1/2} = \frac{ln2}{k}$$

k – rate constant, $t_{1/2}$ – thermal half-life.

Irradiation Time Course. To measure the change in absorbance under UV/Vis irradiation, 100 µM solutions of the respective compounds were prepared as described above in a quartz cuvette (V = 2 mL, d = 1 cm) equipped with a magnetic stir bar. The cuvette was placed on a Mettler Toledo UV5Bio spectrometer with a CuveT thermostat, and the change in absorbance recorded while irradiating the sample at the indicated wavelength with continuous stirring.

Demonstration of Photostability. To investigate photostability, 100 µM solutions of the respective compounds were prepared as described above in a quartz cuvette (V = 2 mL, d = 1 cm) equipped with a magnetic stir bar. The cuvette was placed on a Mettler Toledo UV5Bio spectrometer with a CuveT thermostat, and the time course of absorbance recorded while irradiating at 385 nm or 505 nm with continuous stirring. The time the absorbance required to plateau was defined as time to reach the PSS and chosen as irradiation time in the following experiments. In the following irradiation was performed at 385 nm and 505 nm in alternating fashion. The absence of photobleaching was shown when the system returned to the same PSS after each run.

To test for photostability in the blue light photoreactor (446 nm, 10x 35 W LEDs), a spatula tip of compound was dissolved in deuterated solvent (0.7 mL) and mesitylene (10 µL) was added. The solution was irradiated for 1 h and a ^{1}H NMR spectrum recorded immediately afterwards.

Overview about Photophysical Data

Table T4: Photophysical Data in MeCN

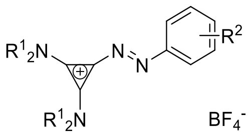

Compound #(R^1, R^2)	$t_{1/2}$[a]	PSS %*Z* (nm)[a]	PSS %*E* (nm)[a]	E ππ* λ_{max} (nm)	$\varepsilon_{\pi\pi*}$ ($M^{-1}cm^{-1}$)	Z ππ* λ_{max} (nm)[c]	Z nπ* λ_{max} (nm)[c]	isosbestic wavelengths (nm)	
**3a**(*i*-Pr, Ph)	4 min	90% (385)[b]	88% (505)[b]	393	22000	301[c]	453[c]	330	461
**3b**(*i*-Pr, *p*-OMe)	n.d.	n.d.	n.d.	414	22000	n.d.	n.d.		
**6a**(Me, Ph)	73 min	92% (385)	90% (505)	386	14000	333	451	335	447
**6b**(Me, morpholinyl)	n.d.	n.d.	n.d.	507	18000	n.d.	n.d.	n.d.	n.d.
**6c**(Me, *p*-OMe)	16 min	81% (415)	82% (530)	412	26000	347	466	360	492
		83% (385)	78% (505)						
**6e**(Me, *p*-Cl)	5 min	88% (385)[b]	82% (505)[b]	392	24000	332[d]	457[d]	342	458
**6f**(Me, *p*-Br)	89 min	85% (385)	86% (505)	393	21000	332	459	344	461
**6j**(Me, *p*-CN)	1 min	n.d.	n.d.	393	21000	n.d.	n.d.	n.d.	n.d.
**6k**(Me, *m*-OMe)	182 min	85% (385)	84% (505)	390	19000	337	451	338	465
**6l**(Me, *m*-CO_2Me)	17 min	83% (385)	87% (505)	387	23000	341	452	335	448
**6n**(Me, thiophenyl)	170 min	94% (385)	87% (505)	387	18000	338	459	341	458
**6o**(Me, 4-pyrazoyl)	336 min	90% (385)[b]	90% (530)[b]	393	19000	n.d.	n.d.	343	475
**6p**(Me, *o*,*o*-diMe)	646 min	92% (385)	66% (505)	384	15000	336	450	339	460
**6q**(Me, *o*,*o*-diF)	69 min	n.d.	n.d.	387	19000	n.d.	n.d.	n.d.	n.d.

[(a)] determined at 25°C.

[(b)] short thermal half-life does not allow the accurate determination of the PSS composition, estimated value based on the criterium of physical meaningfulness (no negative absorbance, maximal absorbance of the E spectrum > maximal absorbance in the dark-adapted state) and comparison to structurally related compounds.

[(c)] value based on the calculated spectrum of the Z-isomer.

Table T5: Photophysical Data in Water

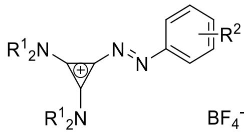

Compound #(R^1, R^2)	$t_{1/2}$[a]	PSS %*Z* (nm)[a]	PSS %*E* (nm)[a]	E ππ* λ_{max} (nm)	$\varepsilon_{\pi\pi^*}$ ($M^{-1}cm^{-1}$)	Z ππ* λ_{max} (nm)[d]	Z nπ* λ_{max} (nm)[d]	isosbestic wavelengths (nm)	
**3a**(*i*-Pr, Ph)	13 min	90% (385)[c]	90% (505)[c]	392	20000	307	460	331	458
**3b**(*i*-Pr, *o*-SO_3^-)	63 min	n.d.	n.d.	403	16000	n.d.	n.d.	337	464
**6a**(Me, Ph)	452 min	91% (385)	85% (505)	386	12000	340	446	337	446
**6b**(Me, morpholinyl)	n.d.	n.d.	n.d.	513	26000	n.d.	n.d.	n.d.	n.d.
**6c**(Me, *p*-OMe)	93 min	80% (415)	81% (530)	410	22000	347	470	360	491
		81% (385)	78% (505)						
**6e**(Me, *p*-Cl)	361 min	90% (385)	84% (505)	392	21000	340	452	342	460
**6f**(Me, *p*-Br)	694 min	89% (385)	85% (505)	392	22000	341	454	345	460
**6h**(Me, *p*-CO_2Me)	80 min	67% (385)	90% (505)	394	22000	347	453	337	456
**6i**(Me, *p*-CO_2H)	158 min	n.d.	n.d.	392	22000	n.d.	n.d.	340	458
**6j**(Me, *p*-CN)	25 min	n.d.	n.d.	393	22000	n.d.	n.d.	n.d.	n.d.
**6k**(Me, *m*-OMe)	781 min	83% (385)	83% (505)	390	17000	342	451	339	464
**6l**(Me, *m*-CO_2Me)	309 min	86% (385)	86% (505)	385	20000	348	447	334	446
**6n**(Me, thiophenyl)	340 min	92% (385)	87% (505)	388	15000	340	454	343	460
**6o**(Me, 4-pyrazoyl)	223 min[b]	90% (385)	81% (505)	394	20000	333	436	341	474
**6p**(Me, *o*,*o*-diMe)	n.d.	80% (385)	73% (505)	380	15000	342	449	340	450
**6q**(Me, *o*,*o*-diF)	318 min	76% (385)	69% (505)	387	17000	350	446	343	450
**11a**(Me/Pro, *p*-OMe	41 min	78% (415)	15% (530)	413	24000	350	466	361	498

[(a)] determined at 25°C. [(b)] determined at 37°C.

[(c)] short thermal half-life does not allow for accurate determination of the PSS composition, estimated value based on the criterium of physical meaningfulness (no negative absorbance, maximal absorbance of the E spectrum > maximal absorbance in the dark-adapted state) and comparison to structurally related compounds.

[(d)] value based on the calculated spectrum of the Z-isomer.

UV/vis Absorption Spectra

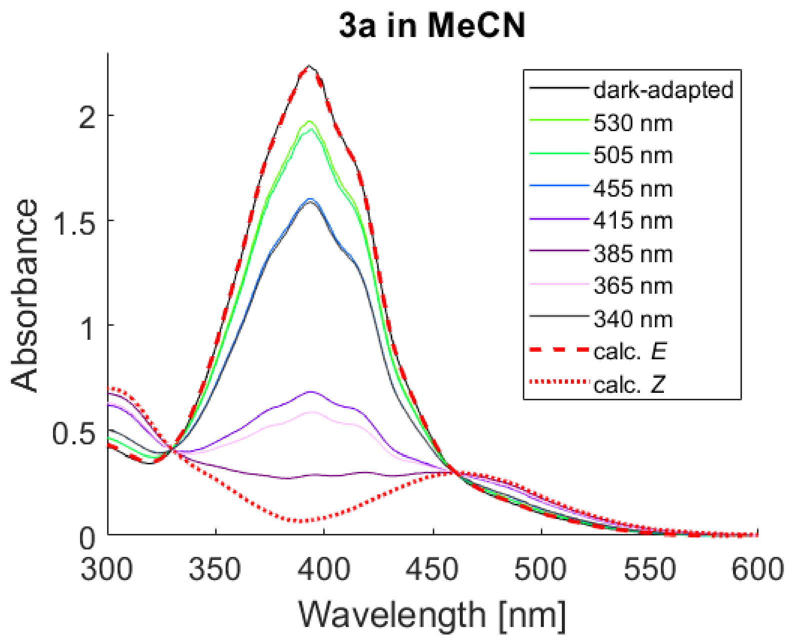

Figure S9: UV/vis absorption spectra of **3a** in MeCN (100 µM) in the dark adapted state and in PSSs after irradiation at the stated wavelengths; *Notes: spectra of the E and Z isomer (red, dashed) estimated based on physical meaningfulness (no negative absorbance, absorbance of the E spectrum = absorbance in the dark-adapted state) and comparison to structurally related* ***6a*** *(NMe_2, Ph); experimental determination not possible due to short thermal half-life; spectra of the PSS after irradiation at 340 and 455 nm overlap.*

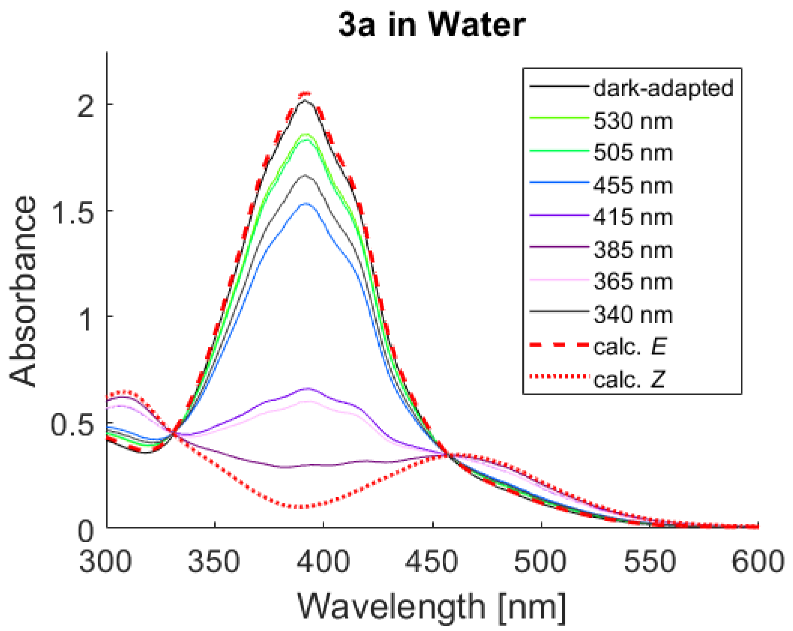

Figure S10: UV/vis absorption spectra of **3a** in water (100 µM) in the dark-adapted state and in PSSs after irradiation at the stated wavelengths; *Notes: spectra of the E and Z isomer (red, dashed) estimated based on physical meaningfulness (no negative absorbance, absorbance of the E spectrum = absorbance in the dark-adapted state) and comparison to structurally related* ***6a*** *(NMe_2, Ph); experimental determination not possible due to short thermal half-life; spectra of the PSS after irradiation at 505 and 530 nm overlap.*

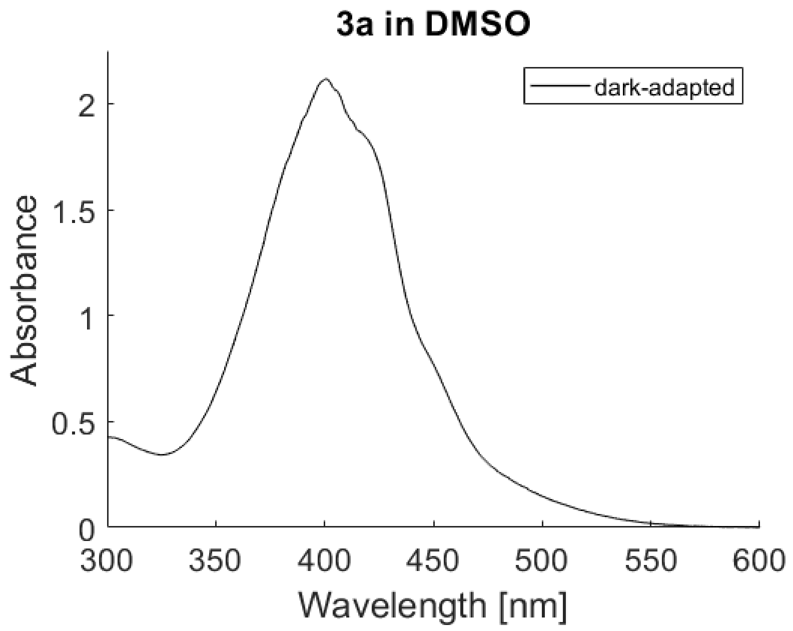

Figure S11: UV/vis absorption spectra of **3a** in DMSO (100 µM), λ_{max} = 401 nm, $\varepsilon_{\pi\pi^*}$ = 21000 ($M^{-1}cm^{-1}$).

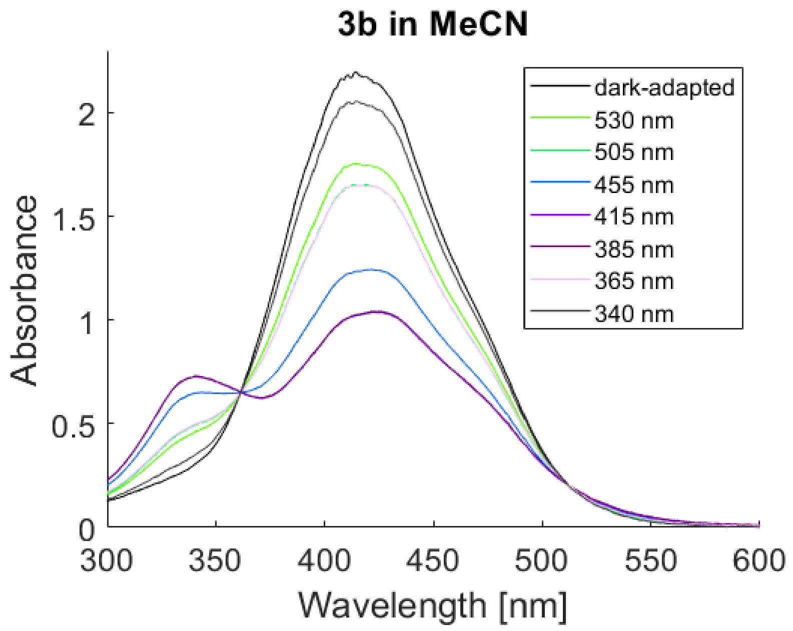

Figure S12: UV/vis absorption spectra of **3b** in MeCN (100 µM) in the dark-adapted state and in PSSs after irradiation at the stated wavelengths; *Note: spectra of the PSSs after irradiation at 365 and 505 nm as well as 385 and 415 nm overlap*.

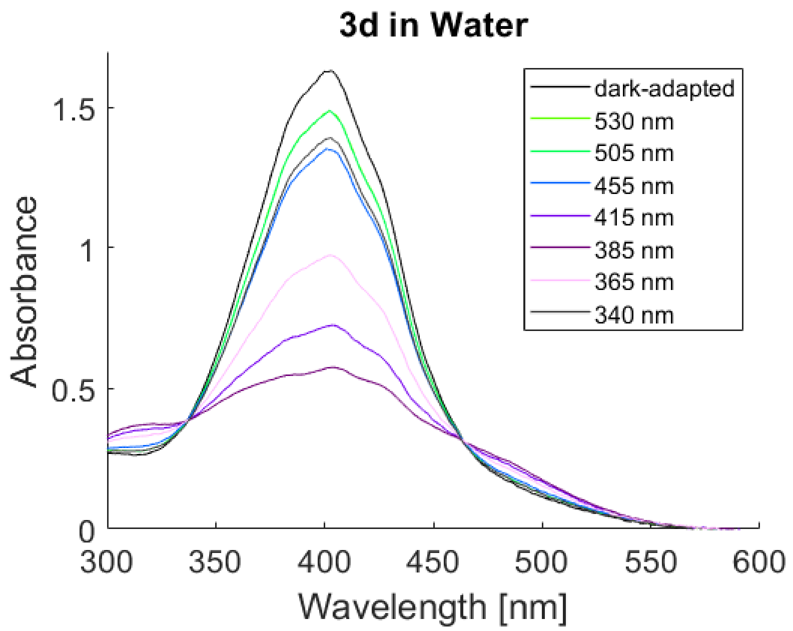

Figure S13: UV/vis absorption spectra of **3d** in water (100 µM) in the dark-adapted state and in PSSs after irradiation at the stated wavelengths; *Note: spectra of the PSS after irradiation at 505 and 530 nm overlap*.

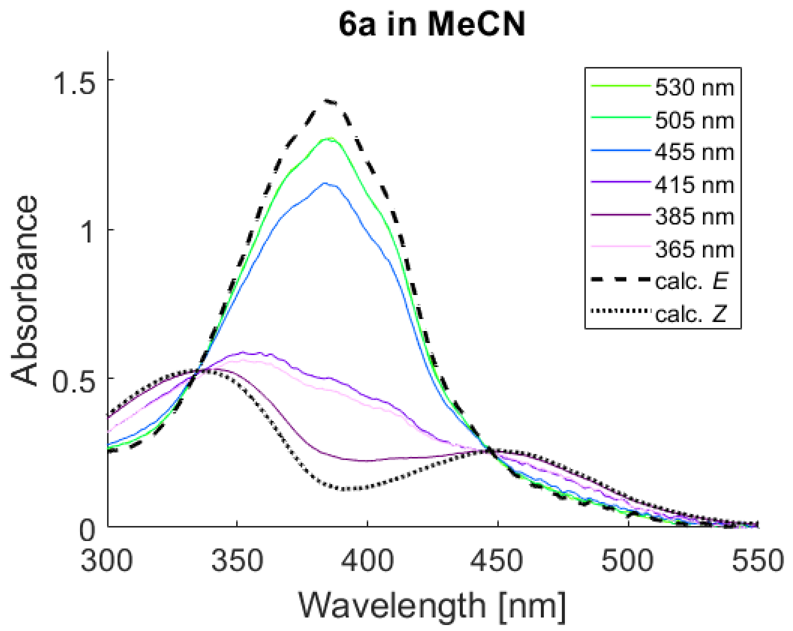

Figure S14: UV/vis absorption spectra of **6a** in MeCN (100 µM) in PSSs after irradiation at the stated wavelengths; calculated spectra are indicated with dashed lines; *Note: spectra of the PSS after irradiation at 505 and 530 nm overlap*.

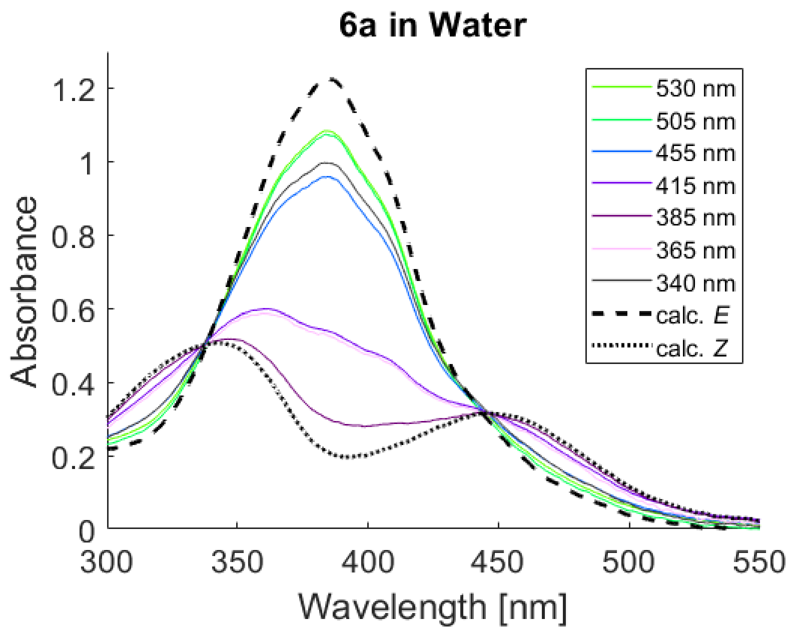

Figure S15: UV/vis absorption spectra of **6a** in water (100 µM) in PSSs after irradiation at the stated wavelengths; calculated spectra are indicated with dashed lines; *Note: spectra of the PSSs after irradiation at 365 and 415 nm as well as 505 and 530 nm overlap*.

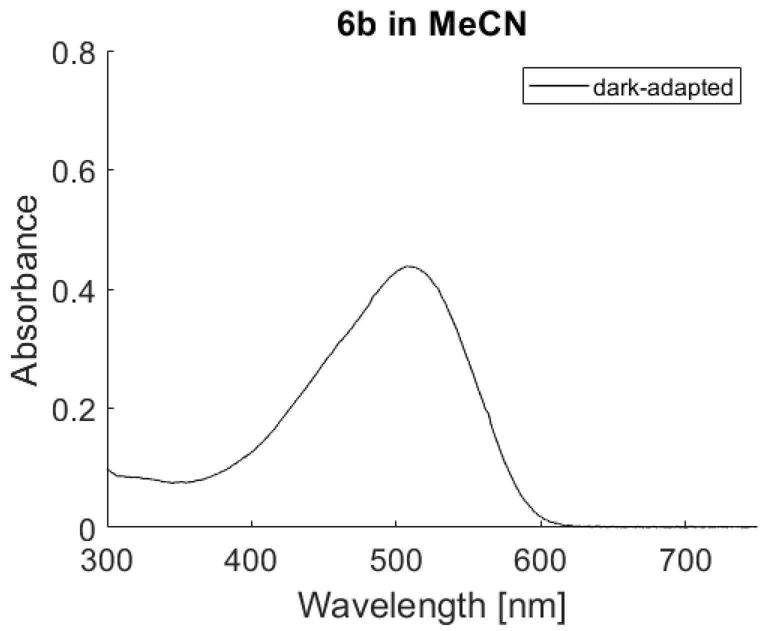

Figure S16: UV/vis absorption spectrum of **6b** in MeCN (25 µM).

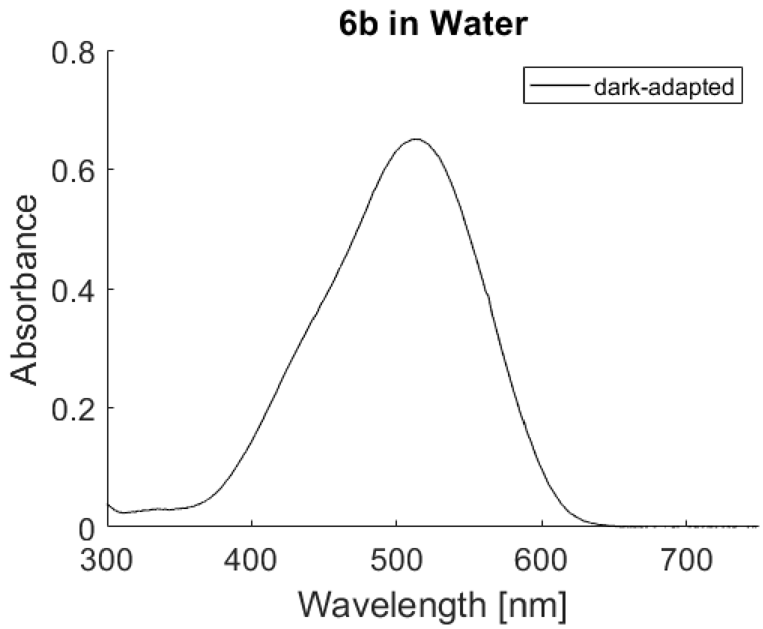

Figure S17: UV/vis absorption spectrum of **6b** in water (25 µM).

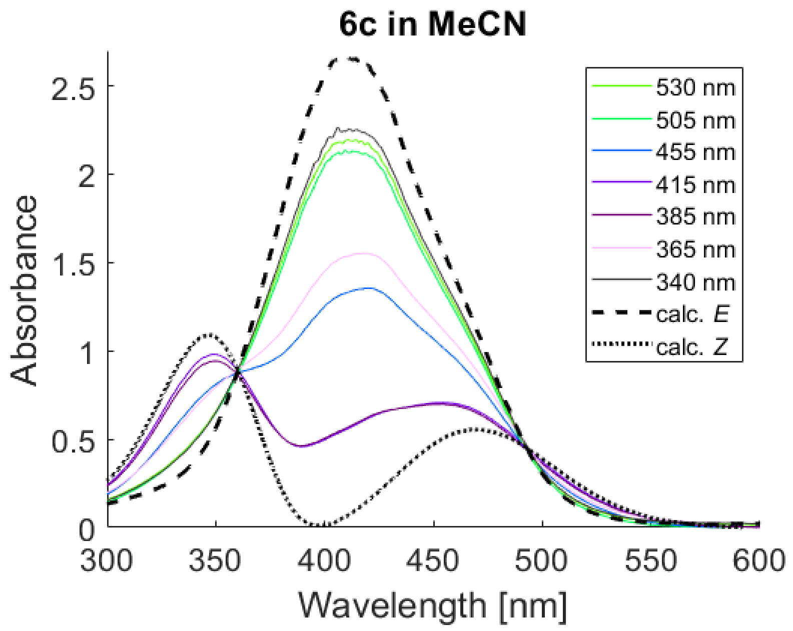

Figure S18: UV/vis absorption spectra of **6c** in MeCN (100 µM) in PSSs after irradiation at the stated wavelengths; calculated spectra are indicated with dashed lines; *Note: spectra of the PSS after irradiation at 385 and 415 nm overlap*.

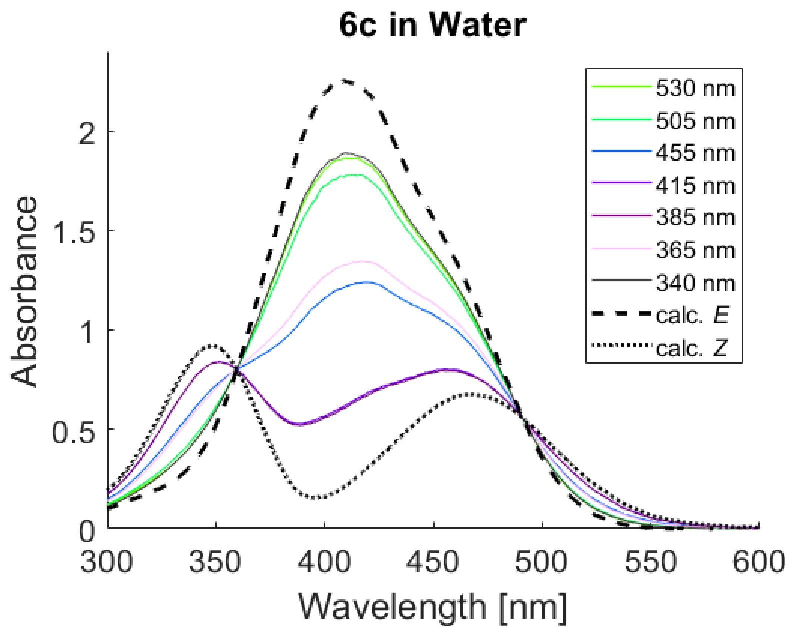

Figure S19: UV/vis absorption spectra of **6c** in water (100 µM) in PSSs after irradiation at the stated wavelengths; calculated spectra are indicated with dashed lines*; Note: spectra of the PSS after irradiation at 385 and 415 nm overlap*.

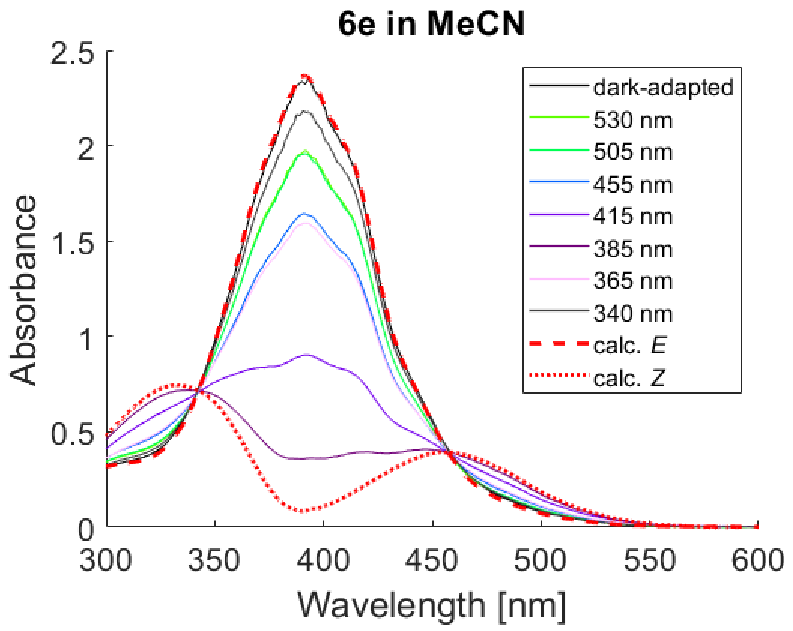

Figure S20: UV/vis absorption spectra of **6e** in MeCN (100 µM) in the dark adapted state and in PSSs after irradiation at the stated wavelengths; *Notes: spectra of the E and Z isomer (red, dashed) estimated based on physical meaningfulness (no negative absorbance, absorbance of the E spectrum = absorbance in the dark-adapted state) and comparison to structurally related* ***6f*** *(NMe_2, p-Br); experimental determination not possible due to short thermal half-life; spectra of the PSS after irradiation at 505 and 530 nm overlap*.

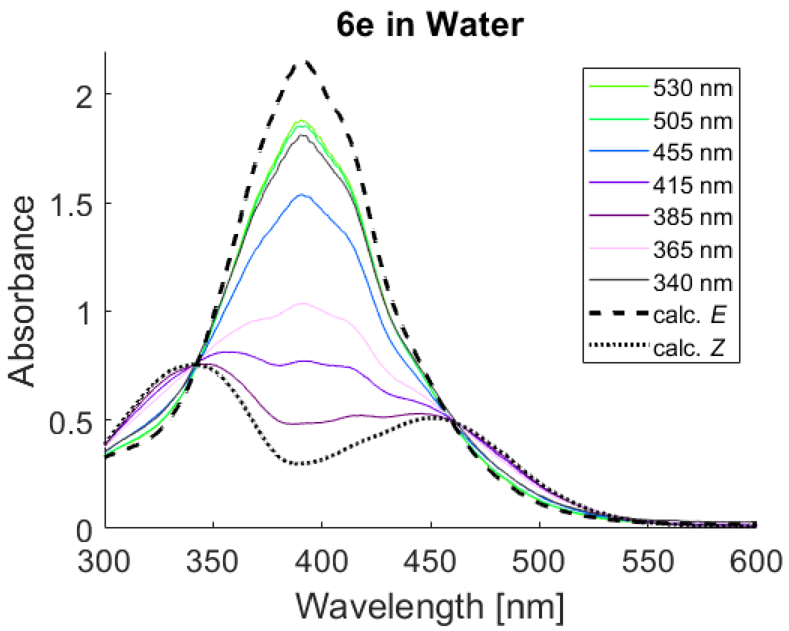

Figure S21: UV/vis absorption spectra of **6e** in water (100 µM) in PSSs after irradiation at the stated wavelengths; calculated spectra are indicated with dashed lines; *Note: spectra of the PSS after irradiation at 505 and 530 nm overlap*.

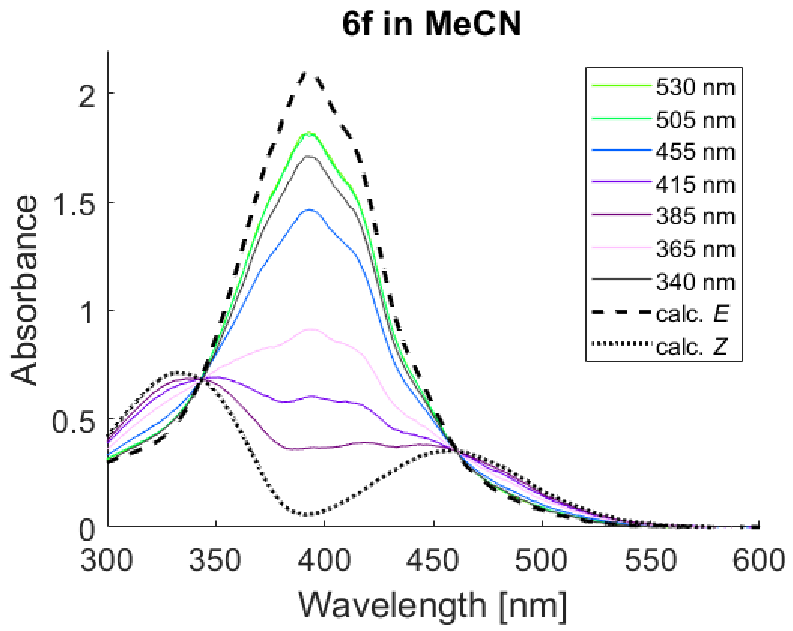

Figure S22: UV/vis absorption spectra of **6f** in MeCN (100 µM) in PSSs after irradiation at the stated wavelengths; calculated spectra are indicated with dashed lines; *Note: spectra of the PSS after irradiation at 505 and 530 nm overlap*.

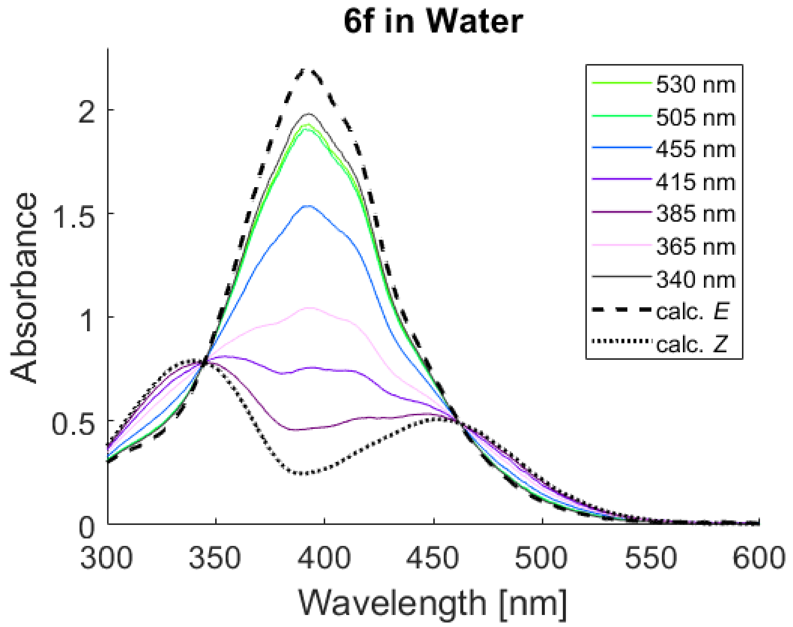

Figure S23: UV/vis absorption spectra of **6f** in water (100 µM) in PSSs after irradiation at the stated wavelengths; calculated spectra are indicated with dashed lines; *Note: spectra of the PSS after irradiation at 505 and 530 nm overlap*.

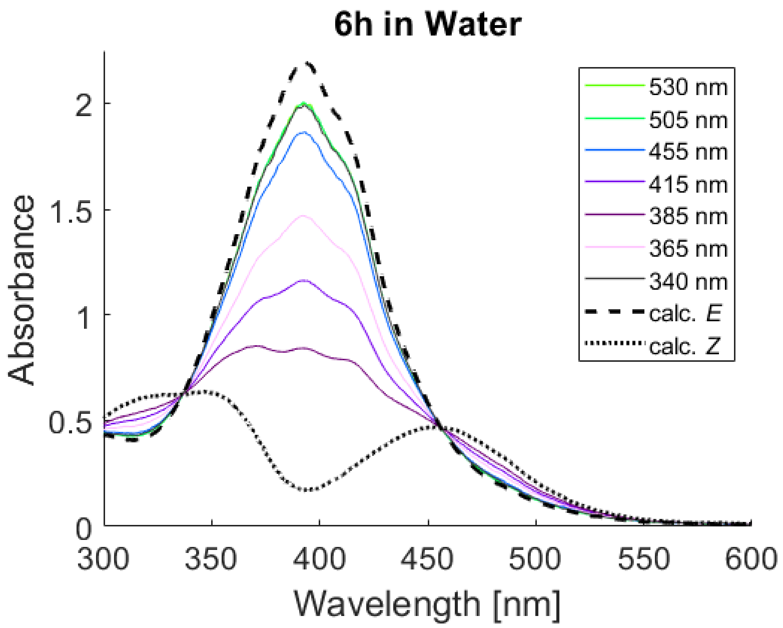

Figure S24: UV/vis absorption spectra of **6h** in water (100 μM) in PSSs after irradiation at the stated wavelengths; calculated spectra are indicated with dashed lines; *Note: spectra of the PSS after irradiation at 340, 505, and 530 nm overlap*.

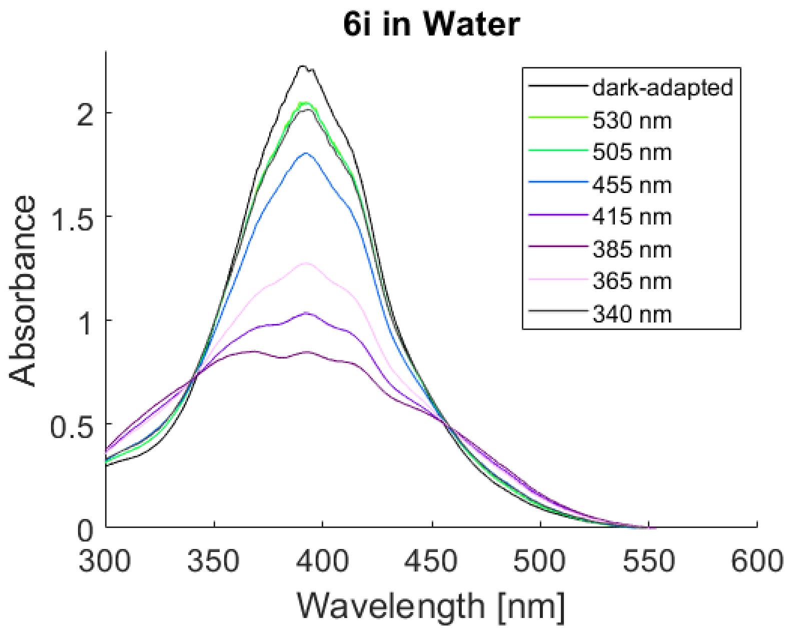

Figure S25: UV/vis absorption spectra of **6i** in water (100 μM) in the dark-adapted state and in PSSs after irradiation at the stated wavelengths; *Note: spectra of the PSS after irradiation at 340, 505, and 530 nm overlap*.

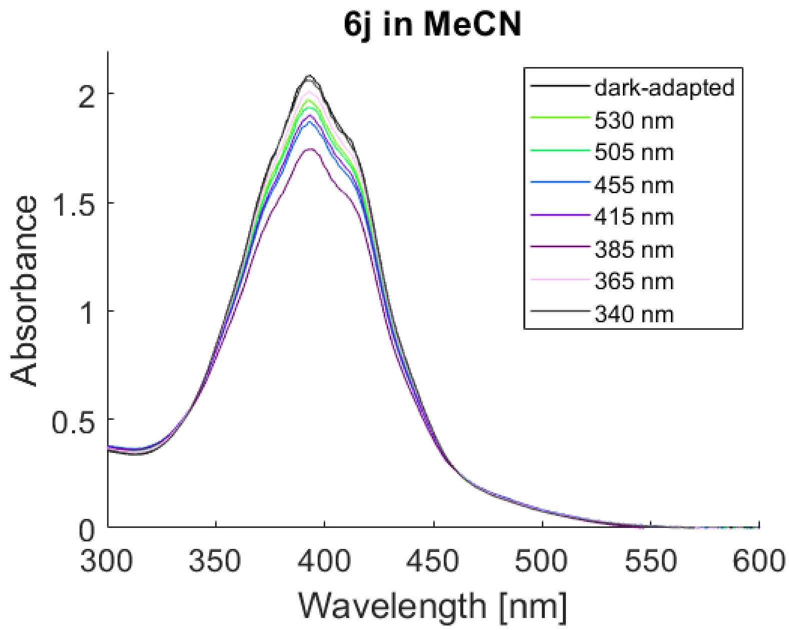

Figure S26: UV/vis absorption spectra of **6j** in MeCN (100 µM) in the dark-adapted state and in PSSs after irradiation at the stated wavelengths; *Note: most spectra overlap*.

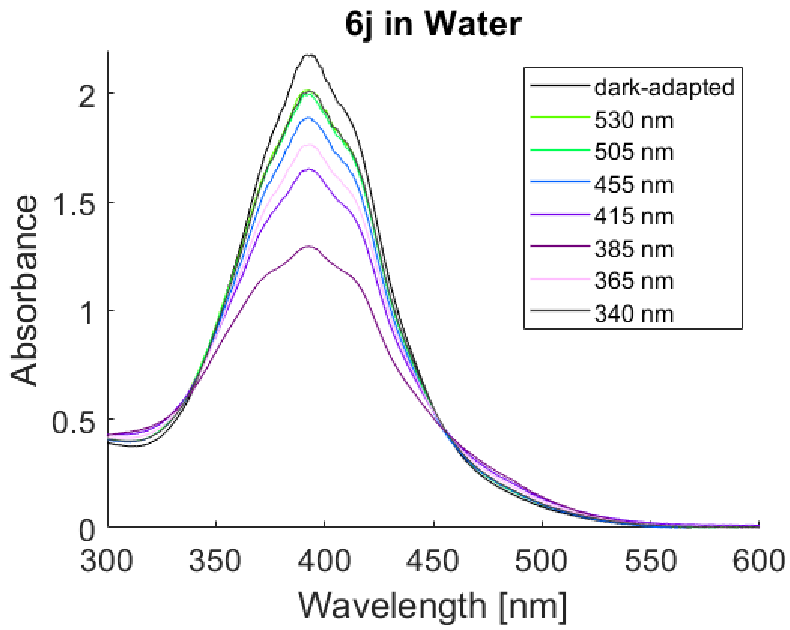

Figure S27: UV/vis absorption spectra of **6j** in water (100 µM) in the dark-adapted state and in PSSs after irradiation at the stated wavelengths; *Note: spectra of the PSS after irradiation at 340, 505, and 530 nm overlap*.

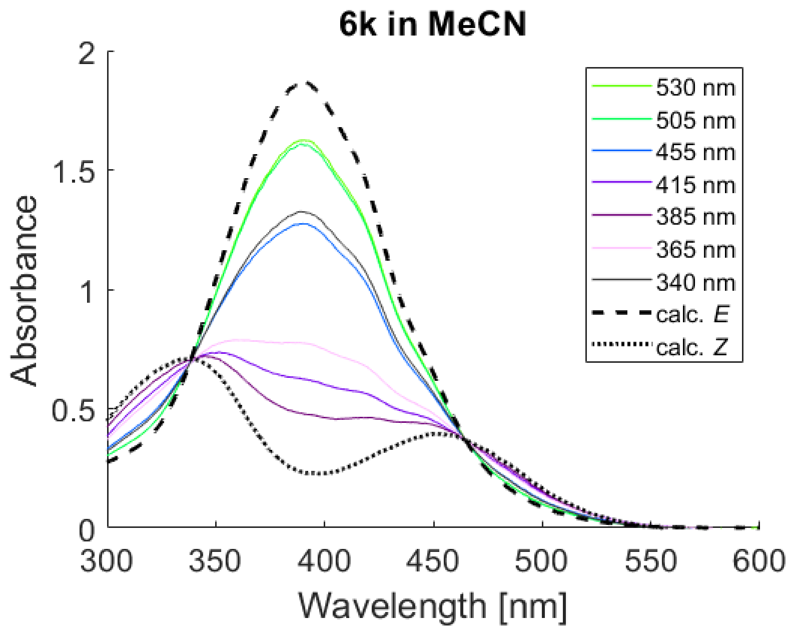

Figure S28: UV/vis absorption spectra of **6k** in MeCN (100 µM) in PSSs after irradiation at the stated wavelengths; calculated spectra are indicated with dashed lines; *Note: spectra of the PSS after irradiation at 505, and 530 nm overlap*.

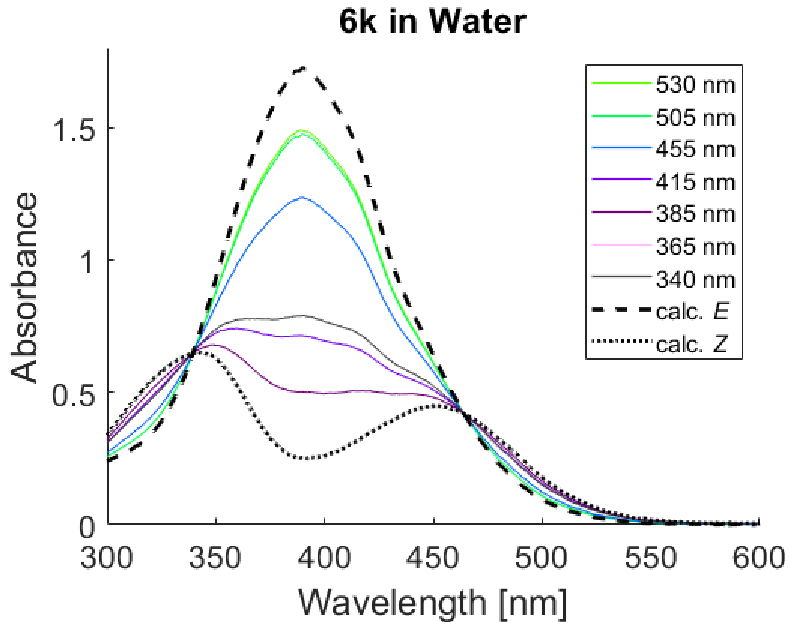

Figure S29: UV/vis absorption spectra of **6k** in water (100 µM) in PSSs after irradiation at the stated wavelengths; calculated spectra are indicated with dashed lines*; Note: spectra of the PSS after irradiation at 365, and 415 nm as well as 505 and 530 nm overlap*.

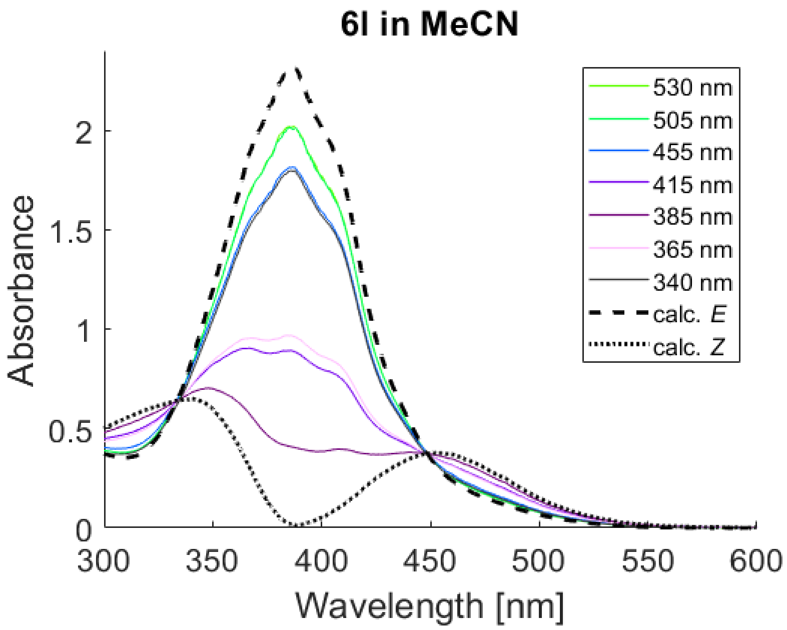

Figure S30: UV/vis absorption spectra of **6l** in MeCN (100 µM) in PSSs after irradiation at the stated wavelengths; calculated spectra are indicated with dashed lines; *Note: spectra of the PSS after irradiation at 340 and 455 nm as well as 505 and 530 nm overlap*.

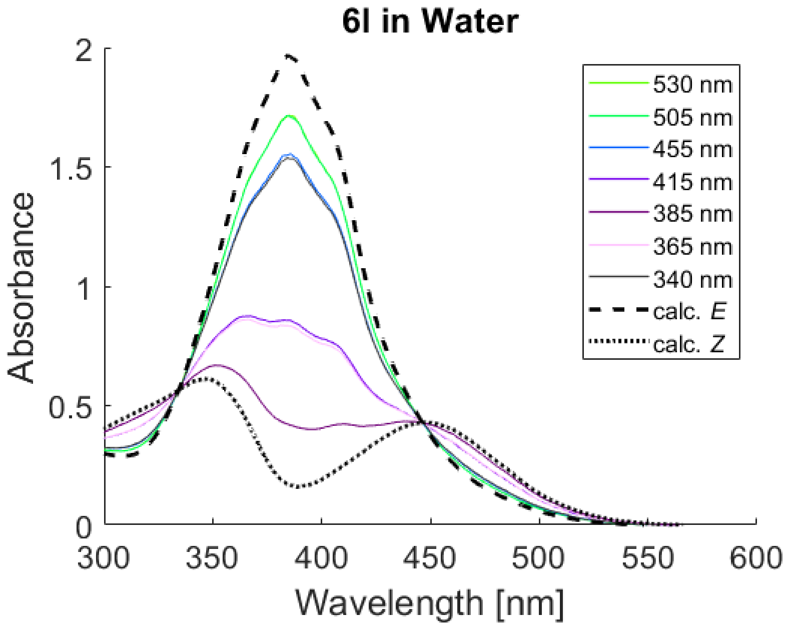

Figure S31: UV/vis absorption spectra of **6l** in water (100 µM) in PSSs after irradiation at the stated wavelengths; calculated spectra are indicated with dashed lines; *Note: spectra of the PSS after irradiation at 340 and 455 nm as well as 505 and 530 nm overlap*.

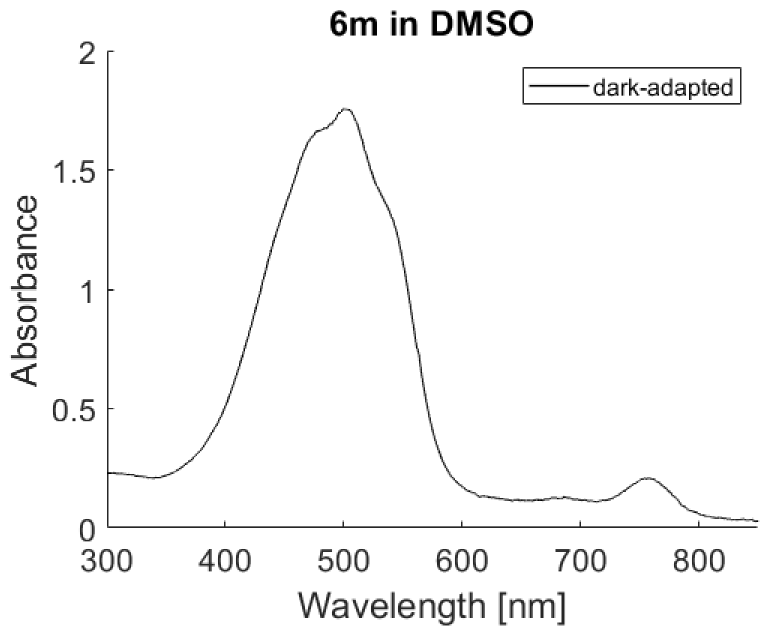

Figure S32: UV/vis absorption spectrum of **6m** in DMSO (50 µM). *Note: Photochromism was not detected which is in accordance with literature reports on 1,4-bis(azo)arenes.*

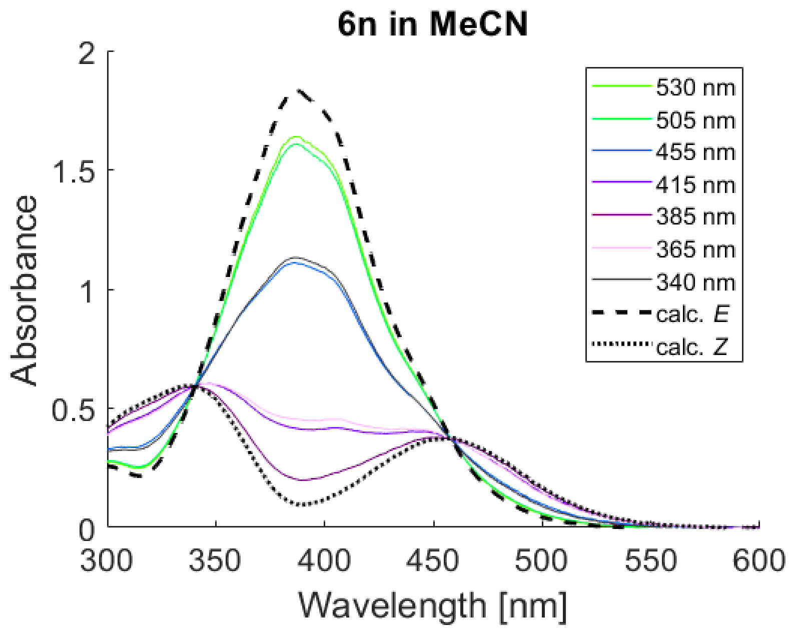

Figure S33: UV/vis absorption spectra of **6n** in MeCN (100 µM) in PSSs after irradiation at the stated wavelengths; calculated spectra are indicated with dashed lines.

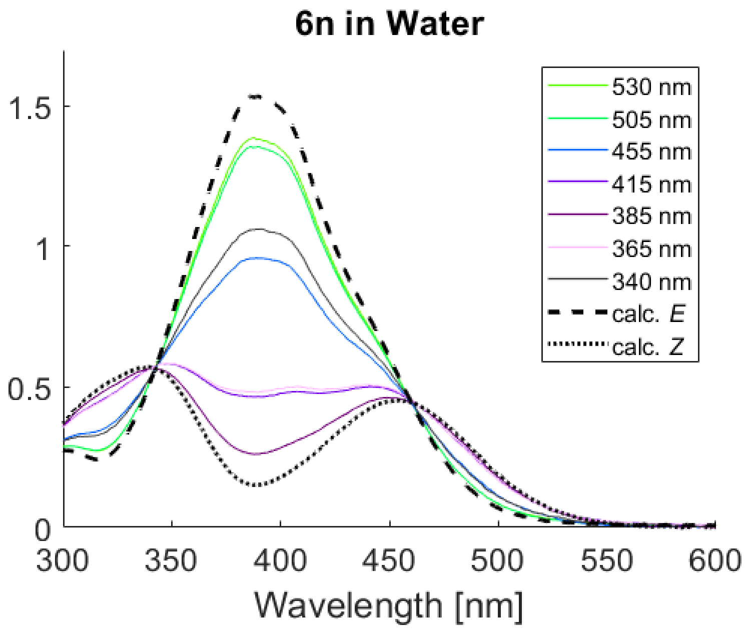

Figure S34: UV/vis absorption spectra of **6n** in water (100 µM) in PSSs after irradiation at the stated wavelengths; calculated spectra are indicated with dashed lines.

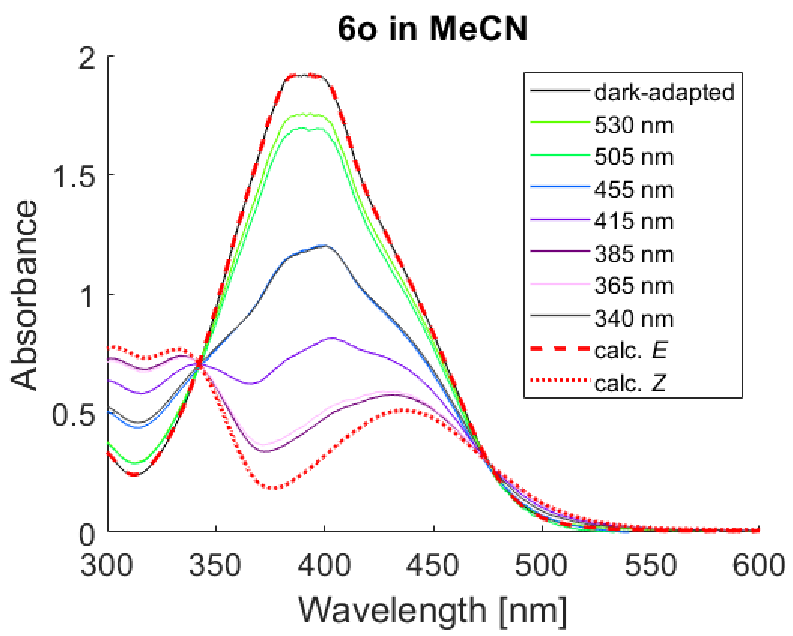

Figure S35: UV/vis absorption spectra of **6o** in MeCN (100 µM) in the dark-adapted state and in PSSs after irradiation at the stated wavelengths; *Notes: spectra of the E and Z isomer (red, dashed) estimated based on physical meaningfulness (no negative absorbance, absorbance of the E spectrum = absorbance in the dark-adapted state) and comparison to* ***6o*** *in water; experimental determination not possible due to peak-broadening in the HPLC chromatogram; spectra of the PSS after irradiation at 340 and 455 nm overlap*.

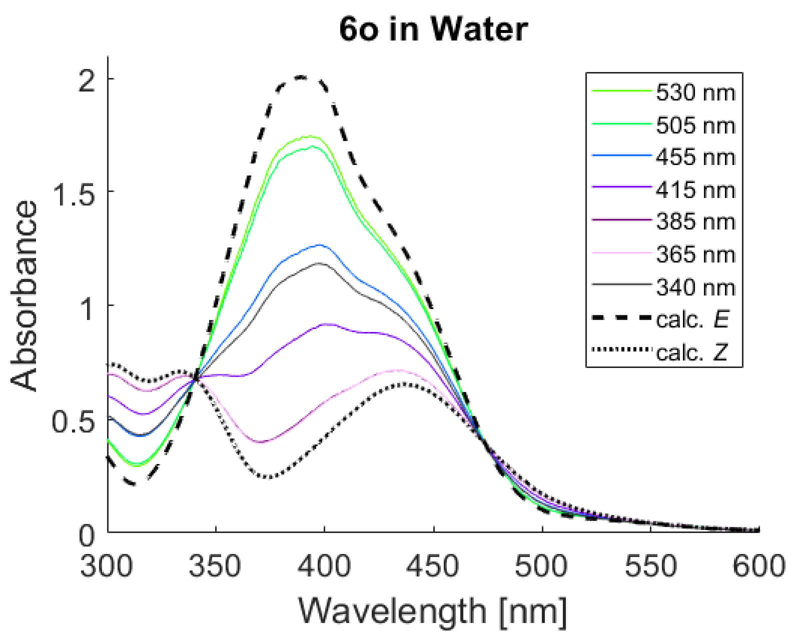

Figure S36: UV/vis absorption spectra of **6o** in water (100 µM) in PSSs after irradiation at the stated wavelengths; calculated spectra are indicated with dashed lines; *Note: spectra of the PSS after irradiation at 365 and 385 nm overlap*.

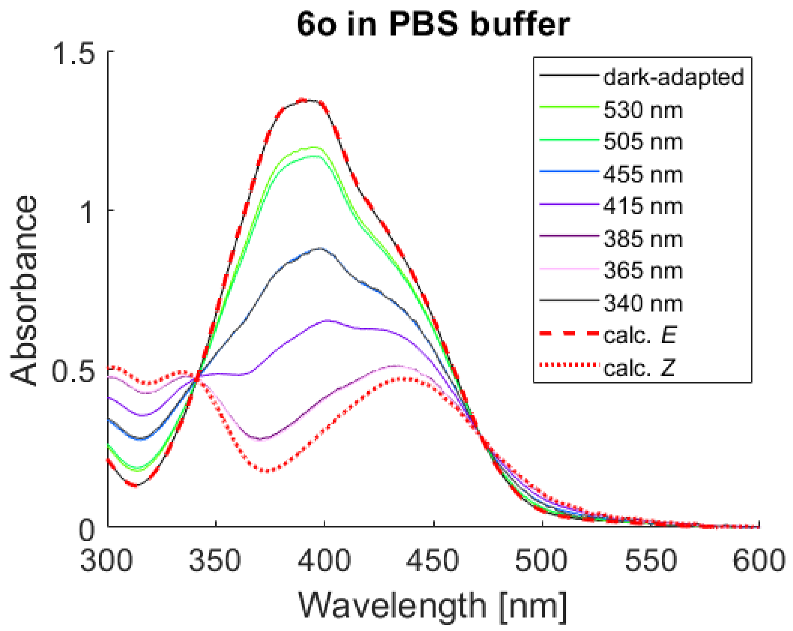

Figure S37: UV/vis absorption spectra of **6o** (70 µM) in PBS buffer (100 mM) in the dark-adapted state and in PSSs after irradiation at the stated wavelengths; *Notes: spectra of the E and Z isomer (red, dashed) estimated based on physical meaningfulness (no negative absorbance, absorbance of the E spectrum = absorbance in the dark-adapted state) and comparison to* ***6o*** *in water: %Z(PSS_{385nm}) = 90%, %E(PSS_{530nm}) = 86%; spectra of the PSS after irradiation at 340 and 455 nm as well as 365 and 385 nm overlap*.

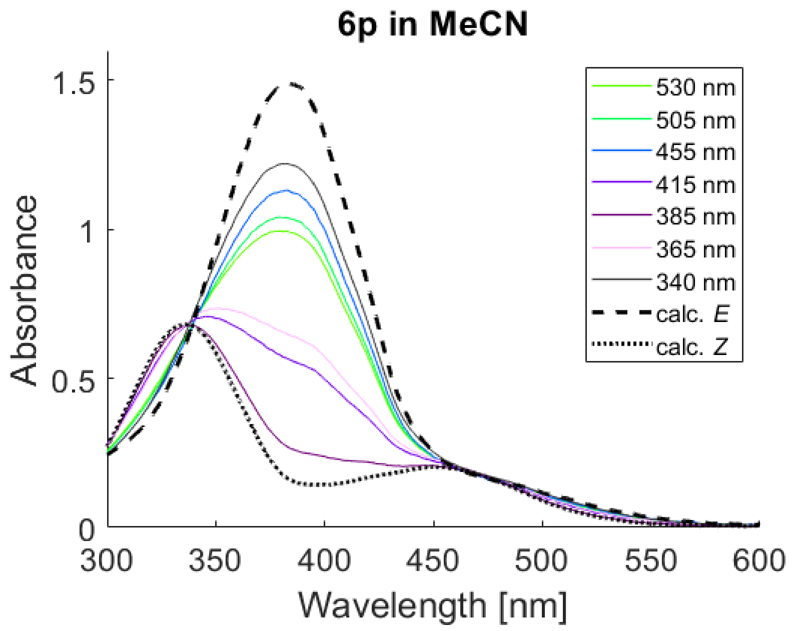

Figure S38: UV/vis absorption spectra of **6p** in MeCN (100 µM) in PSSs after irradiation at the stated wavelengths; calculated spectra are indicated with dashed lines.

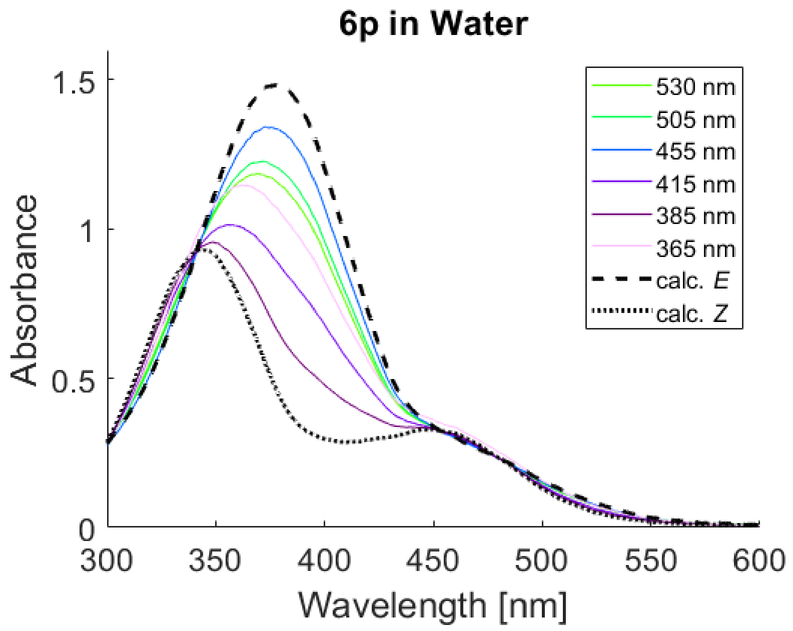

Figure S39: UV/vis absorption spectra of **6p** in water (100 µM) in PSSs after irradiation at the stated wavelengths; calculated spectra are indicated with dashed lines.

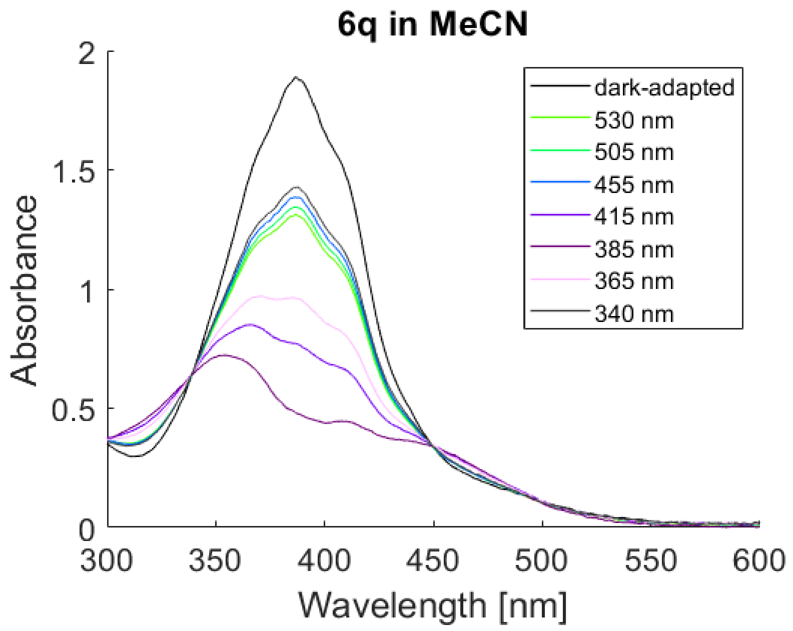

Figure S40: UV/vis absorption spectra of **6q** in MeCN (100 µM) in the dark-adapted state and in PSSs after irradiation at the stated wavelengths.

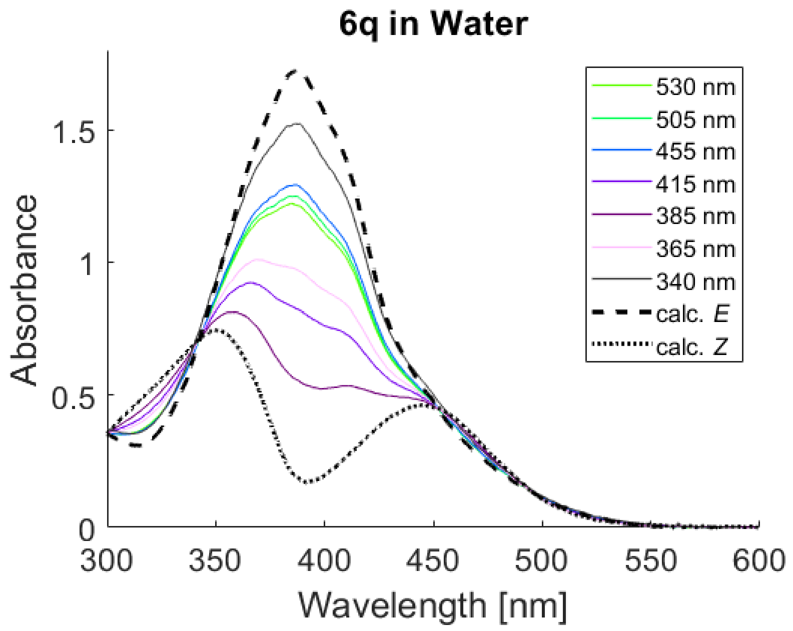

Figure S41: UV/vis absorption spectra of **6q** in water (100 µM) in PSSs after irradiation at the stated wavelengths; calculated spectra are indicated with dashed lines.

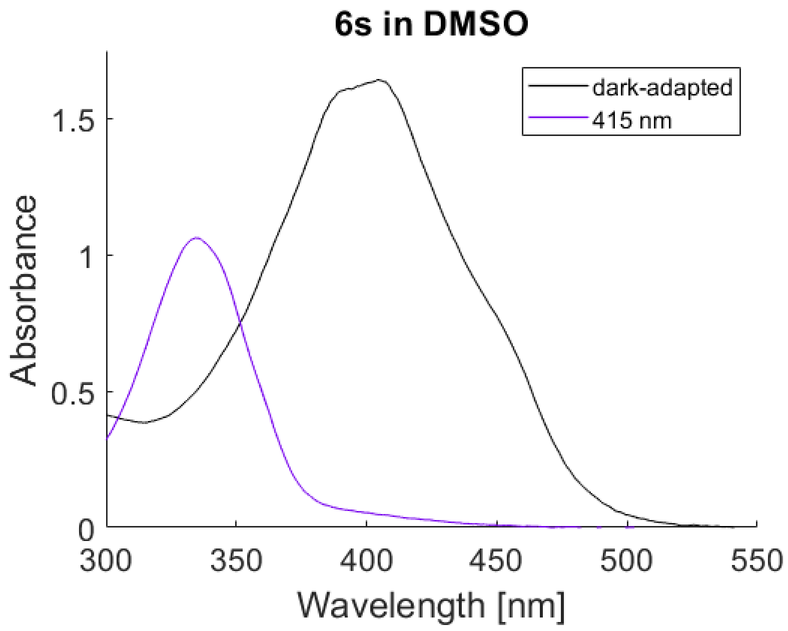

Figure S42: UV/vis absorption spectrum of **6s** in DMSO (100 µM) before and after irradiation at 415 nm for 20 min.

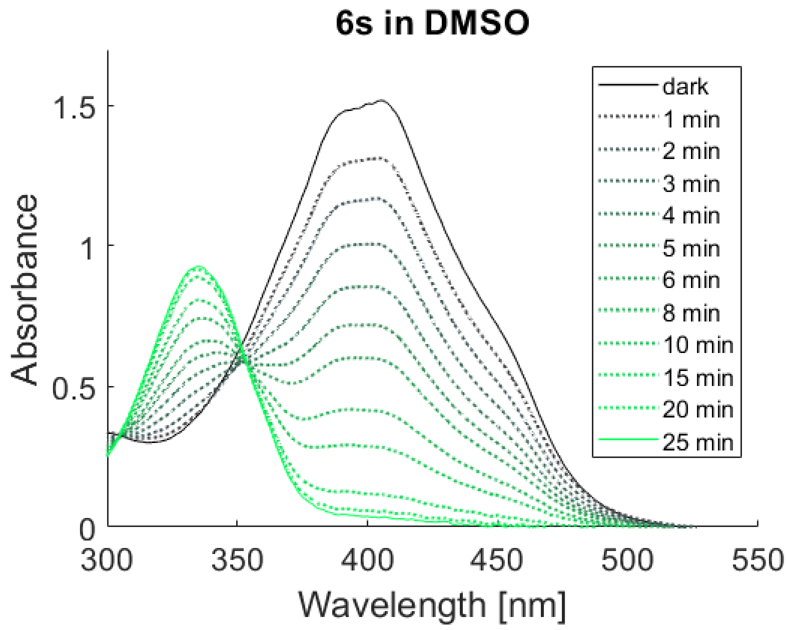

Figure S43: UV/vis absorption spectra of **6s** in DMSO (100 µM) before and after irradiation at 505 nm for 1 – 25 min.

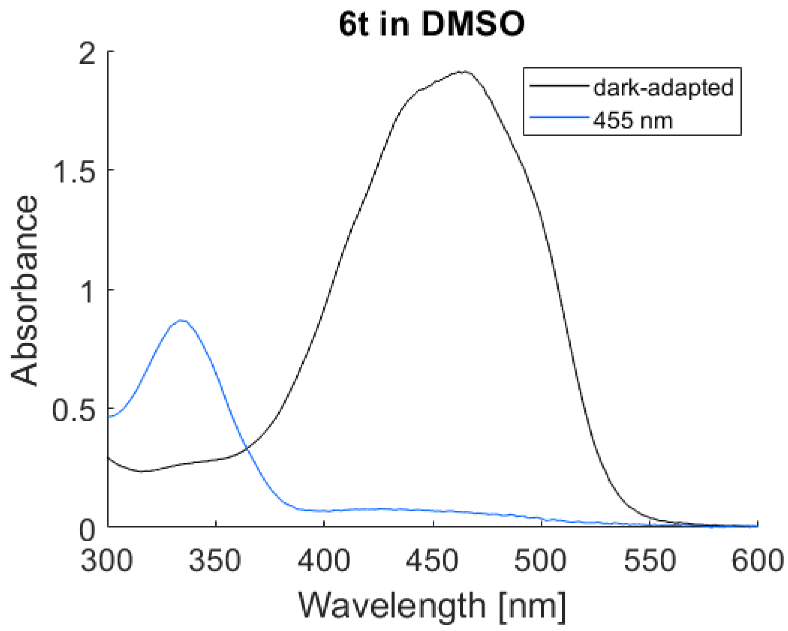

Figure S44: UV/vis absorption spectra of **6t** in DMSO (100 µM) before and after irradiation at 455 nm for 20 min.

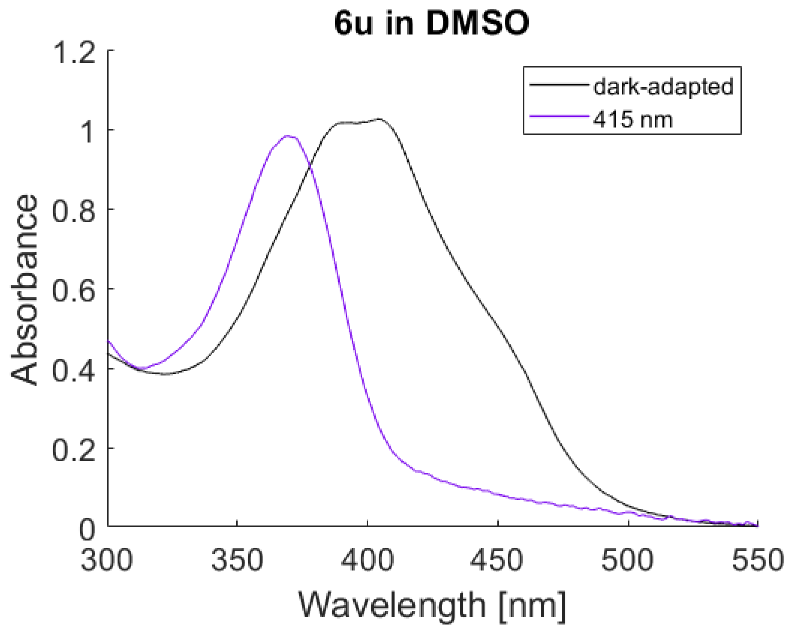

Figure S45: UV/vis absorption spectra of **6r** in DMSO (100 µM) before and after irradiation at 415 nm for 20 min.

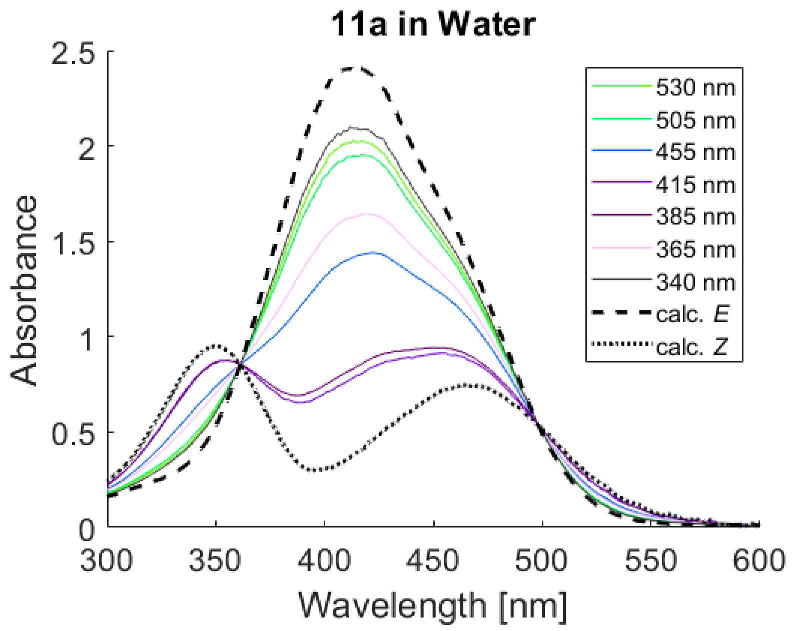

Figure S46: UV/vis absorption spectra of **11a** in water (100 µM) in PSSs after irradiation at the stated wavelengths; calculated spectra are indicated with dashed lines..

Thermal Relaxation Plots

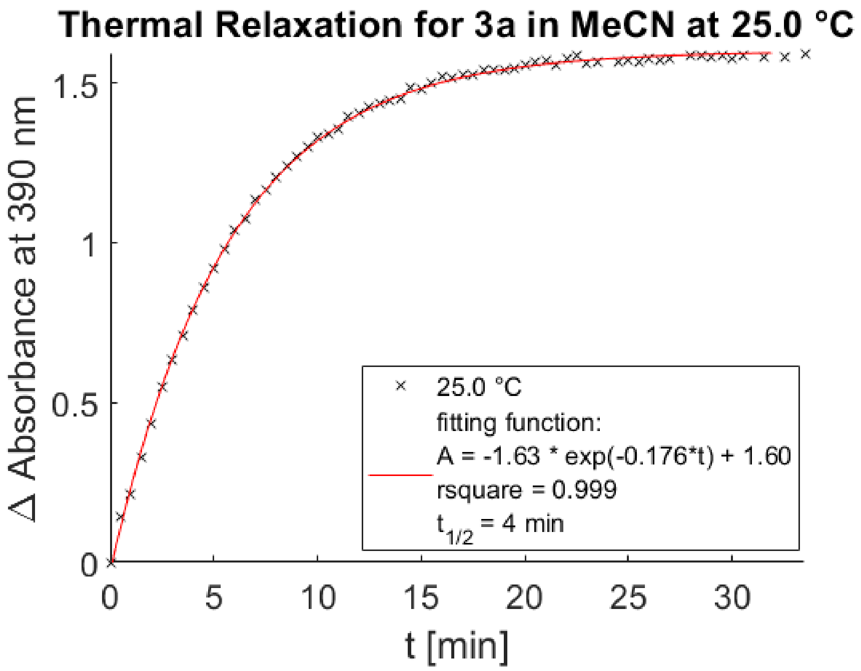

Figure S47: Increase of absorbance at 390 nm and 25.0 °C for a solution of **3a** in MeCN (100 μM) after reaching the photostationary state through irradiation at 385 nm. $t_{1/2}$ is the thermal half-life, calculated from the exponential fitting function assuming a first-order ratelaw.

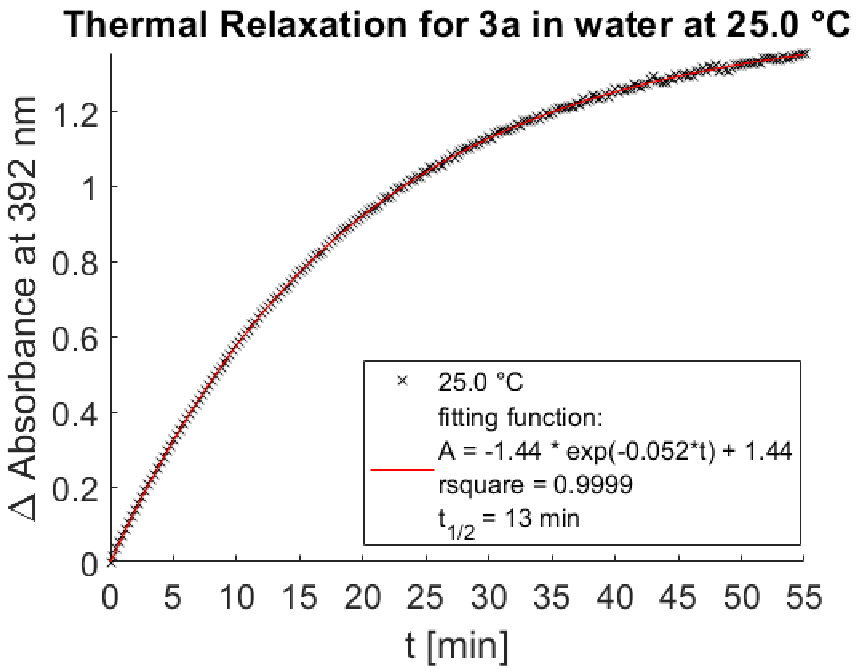

Figure S48: Increase of absorbance at 392 nm and 25.0 °C for a solution of **3a** in water (100 μM) after reaching the photostationary state through irradiation at 385 nm. $t_{1/2}$ is the thermal half-life, calculated from the exponential fitting function assuming a first-order ratelaw.

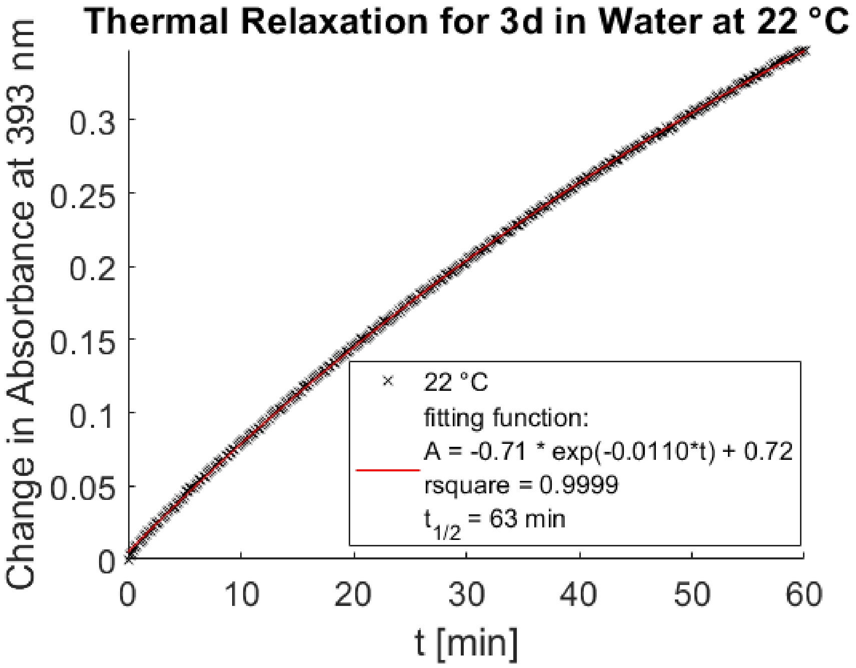

Figure S49: Increase of absorbance at 393 nm and 22 °C for a solution of **3d** in water (100 µM) after reaching the photostationary state through irradiation at 385 nm. $t_{1/2}$ is the thermal half-life, calculated from the exponential fitting function assuming a first-order ratelaw.

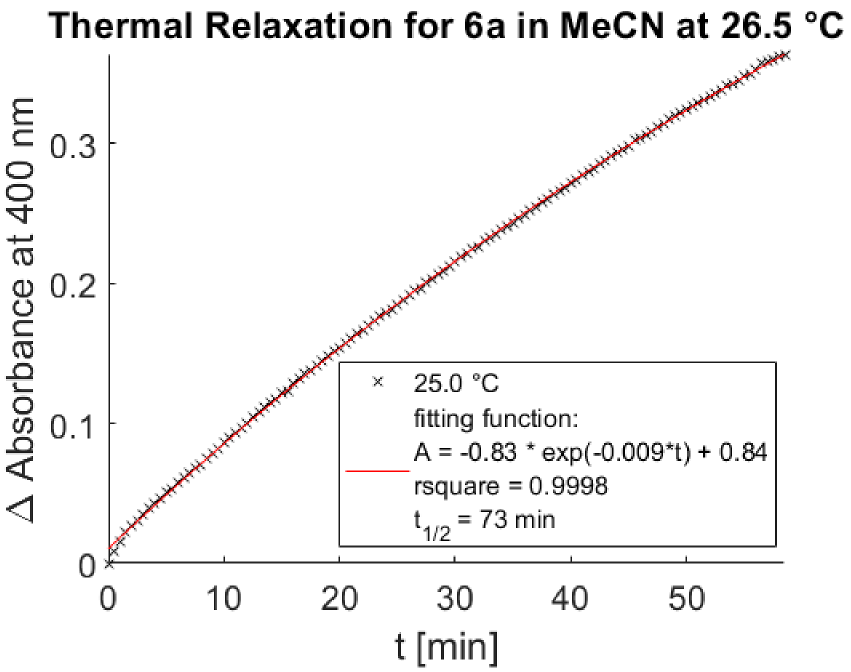

Figure S50: Increase of absorbance at 400 nm and 26.5 °C for a solution of **6a** in MeCN (100 µM) after reaching the photostationary state through irradiation at 385 nm. $t_{1/2}$ is the thermal half-life, calculated from the exponential fitting function assuming a first-order ratelaw.

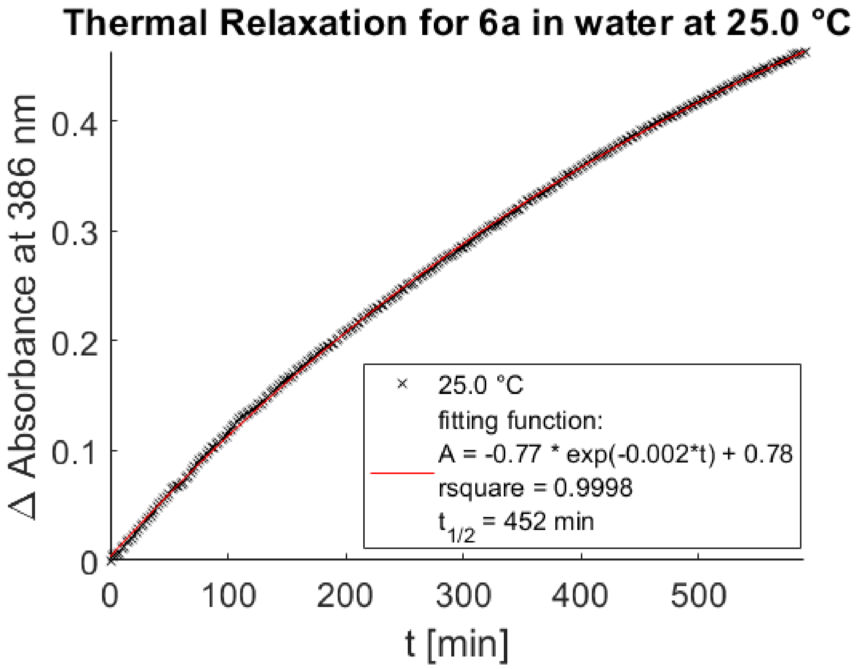

Figure S51: Increase of absorbance at 386 nm and 25.0 °C for a solution of **6a** in water (100 µM) after reaching the photostationary state through irradiation at 385 nm. $t_{1/2}$ is the thermal half-life, calculated from the exponential fitting function assuming a first-order ratelaw.

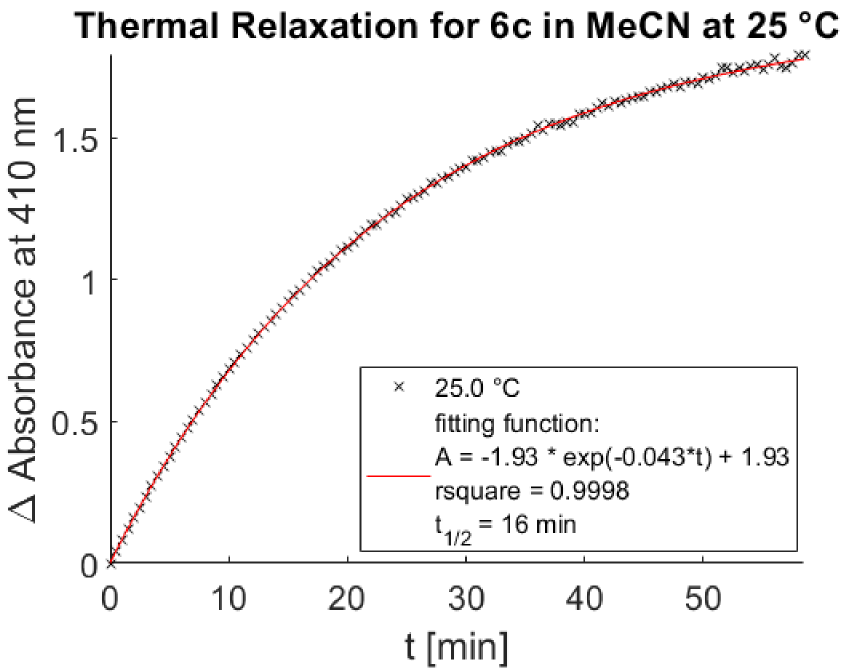

Figure S52: Increase of absorbance at 410 nm and 25 °C for a solution of **6c** in MeCN (100 μM) after reaching the photostationary state through irradiation at 385 nm. $t_{1/2}$ is the thermal half-life, calculated from the exponential fitting function assuming a first-order ratelaw.

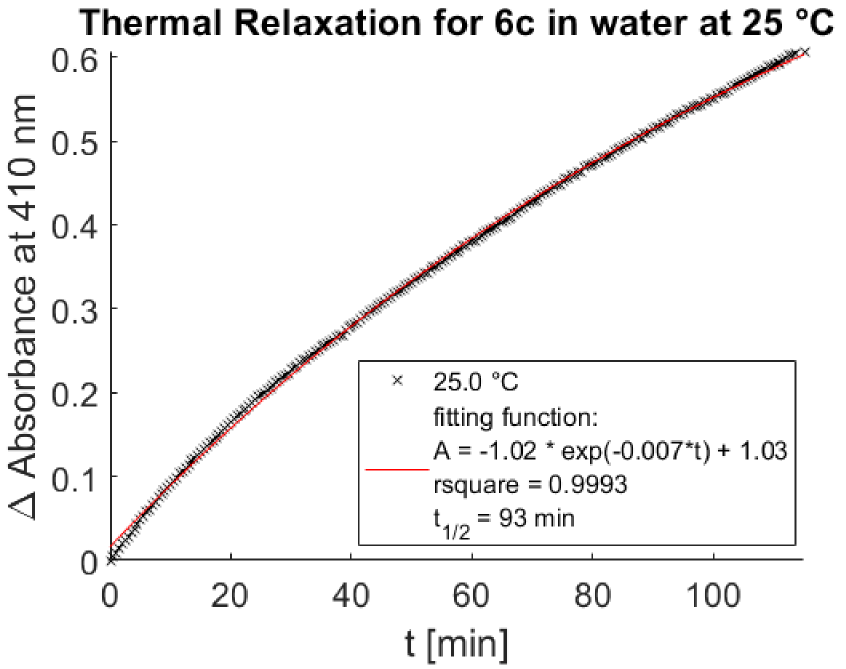

Figure S53: Increase of absorbance at 410 nm and 25 °C for a solution of **6c** in water (100 μM) after reaching the photostationary state through irradiation at 385 nm. $t_{1/2}$ is the thermal half-life, calculated from the exponential fitting function assuming a first-order ratelaw.

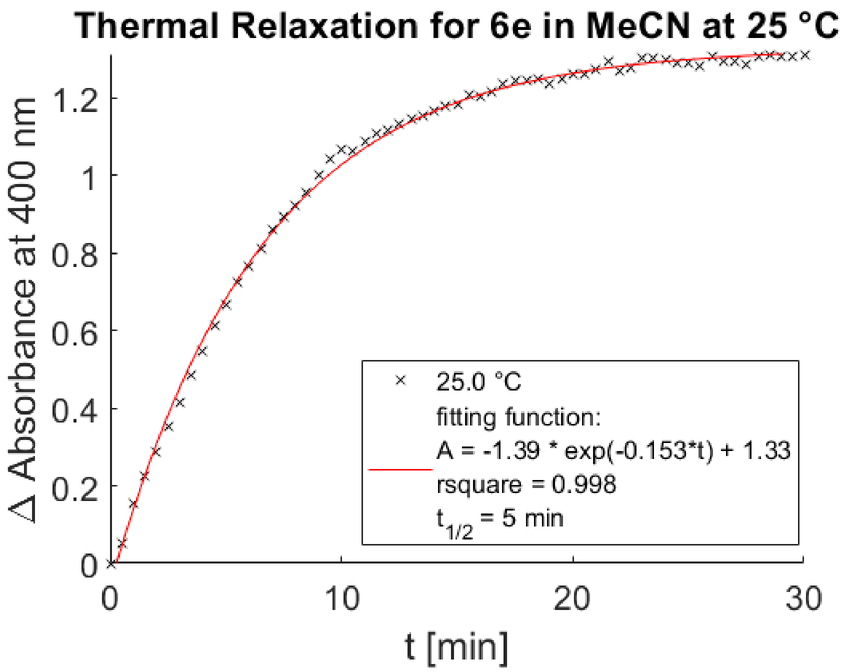

Figure S54: Increase of absorbance at 400 nm and 25 °C for a solution of **6e** in MeCN (100 μM) after reaching the photostationary state through irradiation at 385 nm. $t_{1/2}$ is the thermal half-life, calculated from the exponential fitting function assuming a first-order ratelaw.

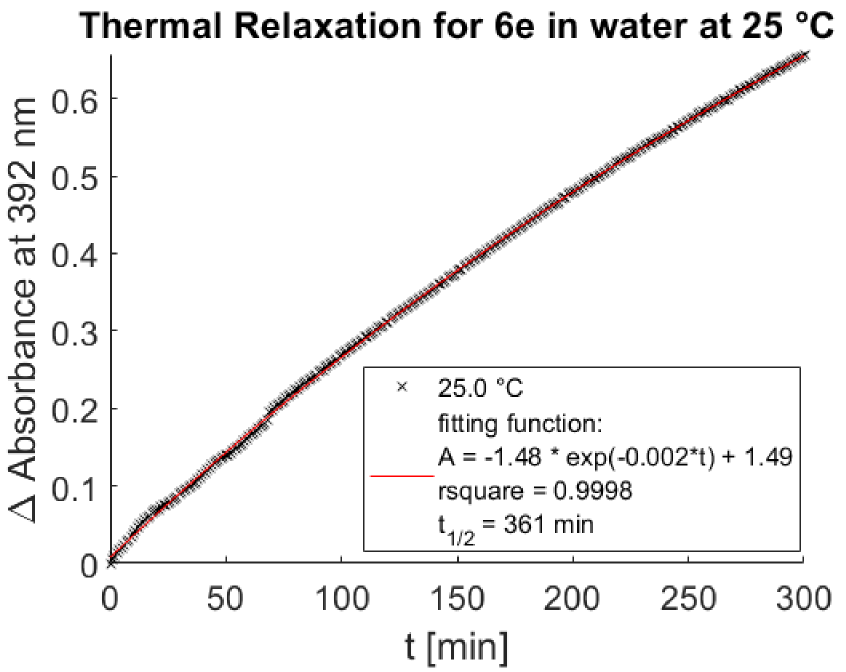

Figure S55: Increase of absorbance at 392 nm and 25 °C for a solution of **6e** in water (100 μM) after reaching the photostationary state through irradiation at 385 nm. $t_{1/2}$ is the thermal half-life, calculated from the exponential fitting function assuming a first-order ratelaw.

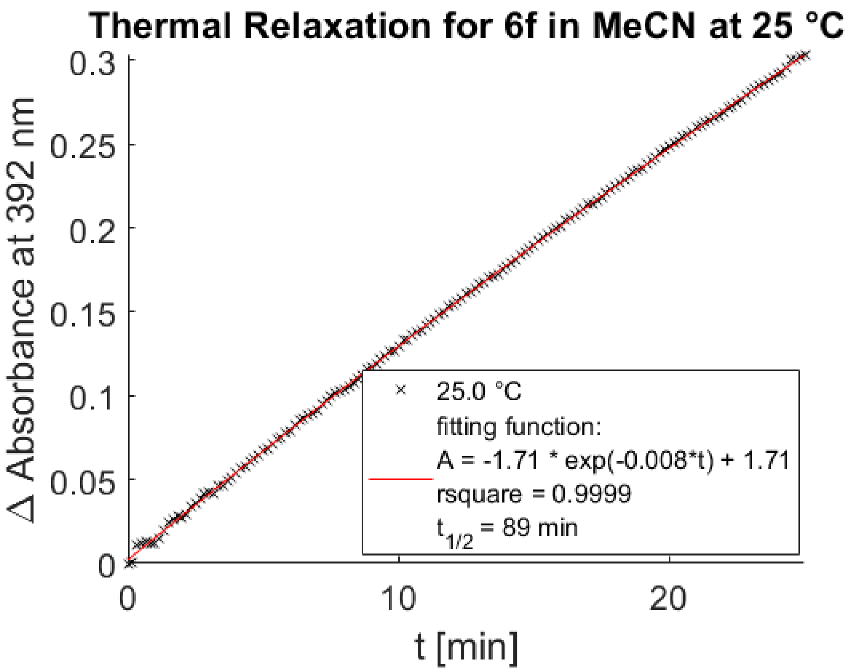

Figure S56: Increase of absorbance at 392 nm and 25 °C for a solution of **6f** in MeCN (100 µM) after reaching the photostationary state through irradiation at 385 nm. $t_{1/2}$ is the thermal half-life, calculated from the exponential fitting function assuming a first-order ratelaw.

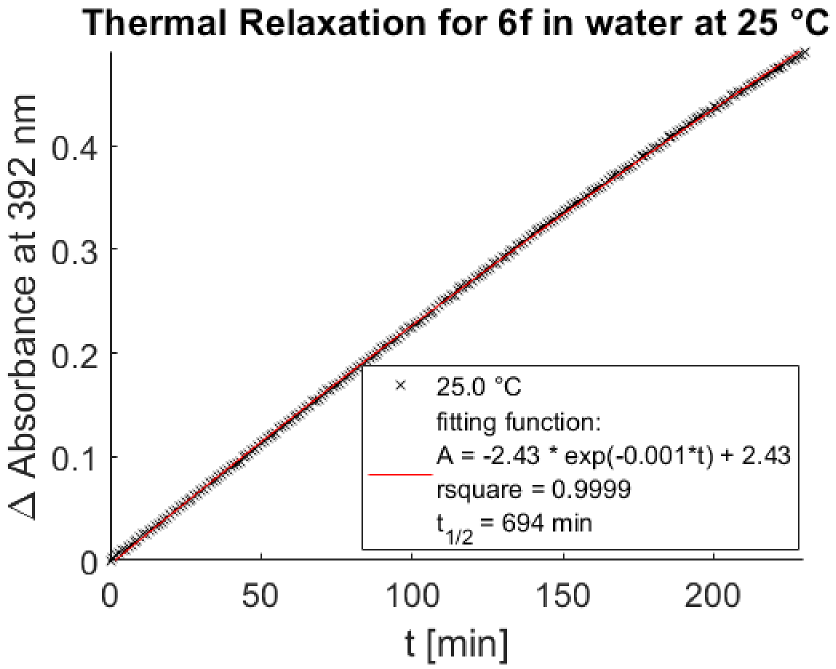

Figure S57: Increase of absorbance at 392 nm and 25 °C for a solution of **6f** in water (100 µM) after reaching the photostationary state through irradiation at 385 nm. $t_{1/2}$ is the thermal half-life, calculated from the exponential fitting function assuming a first-order ratelaw.

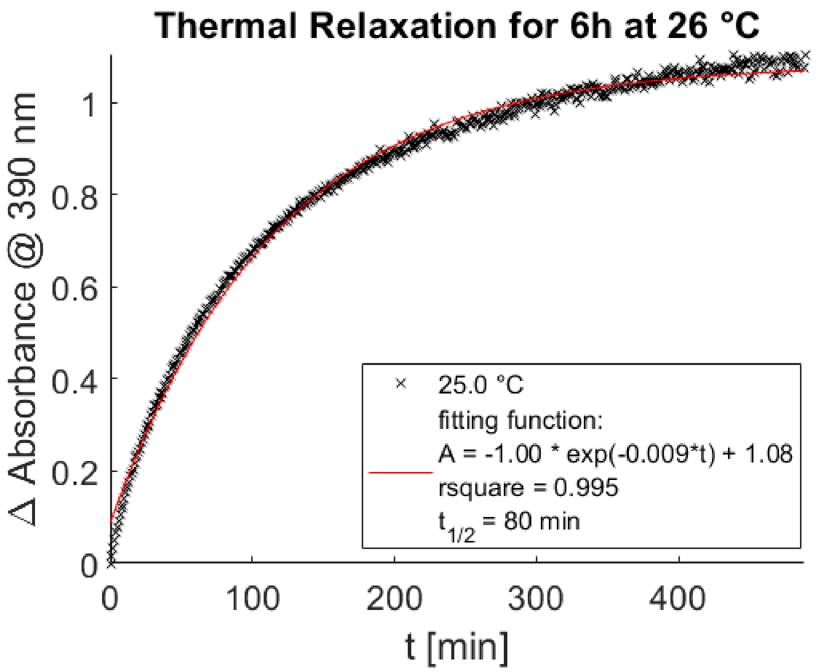

Figure S58: Increase of absorbance at 390 nm and 25 °C for a solution of **6h** in water (100 µM) after reaching the photostationary state through irradiation at 385 nm. $t_{1/2}$ is the thermal half-life, calculated from the exponential fitting function assuming a first-order ratelaw.

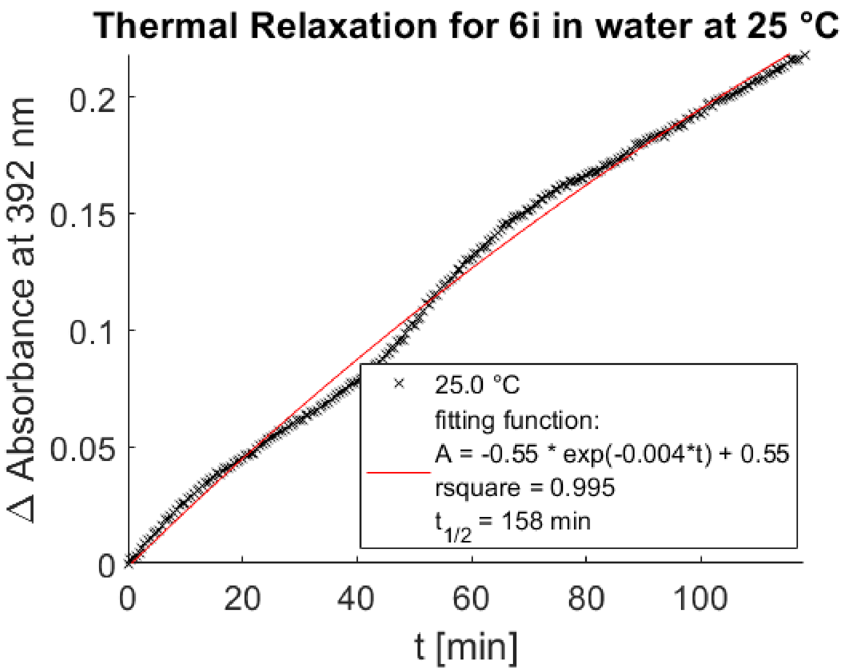

Figure S59: Increase of absorbance at 392 nm and 25 °C for a solution of **6j** in water (100 µM) after reaching the photostationary state through irradiation at 385 nm. $t_{1/2}$ is the thermal half-life, calculated from the exponential fitting function assuming a first-order ratelaw.

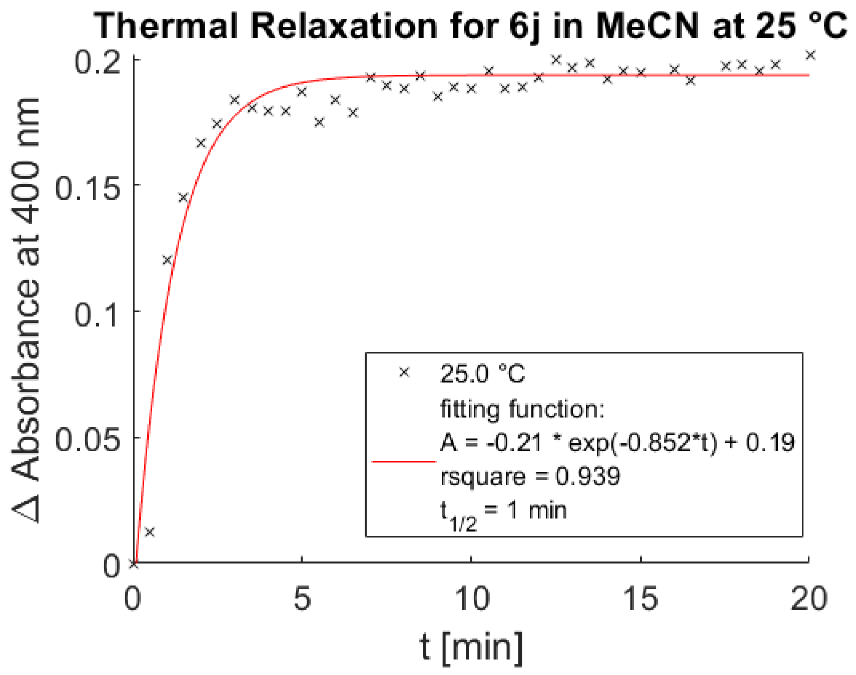

Figure S60: Increase of absorbance at 400 nm and 25 °C for a solution of **6j** in MeCN (100 μM) after reaching the photostationary state through irradiation at 385 nm. $t_{1/2}$ is the thermal half-life, calculated from the exponential fitting function assuming a first-order ratelaw.

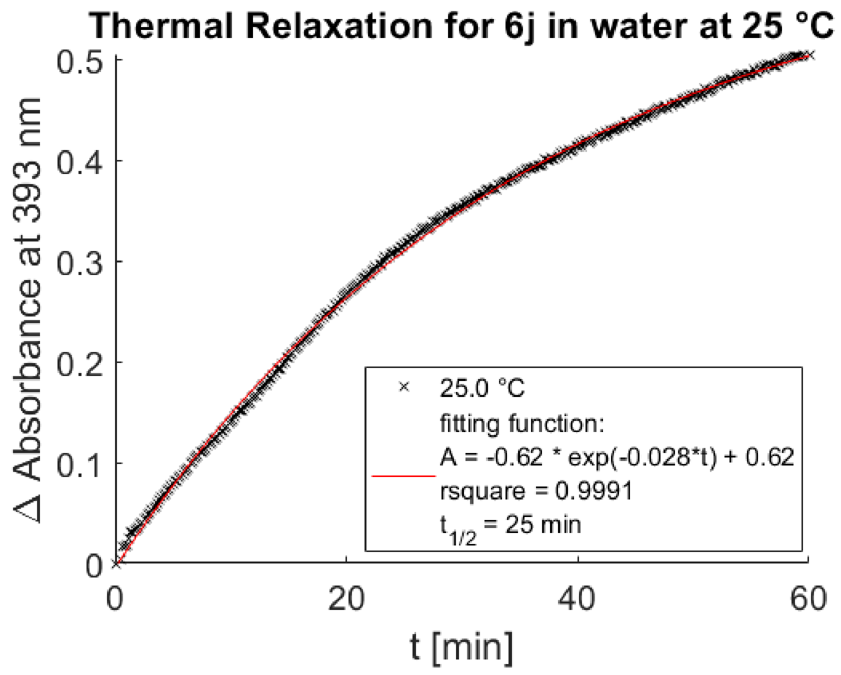

Figure S61: Increase of absorbance at 393 nm and 25 °C for a solution of **6j** in water (100 μM) after reaching the photostationary state through irradiation at 385 nm. $t_{1/2}$ is the thermal half-life, calculated from the exponential fitting function assuming a first-order ratelaw.

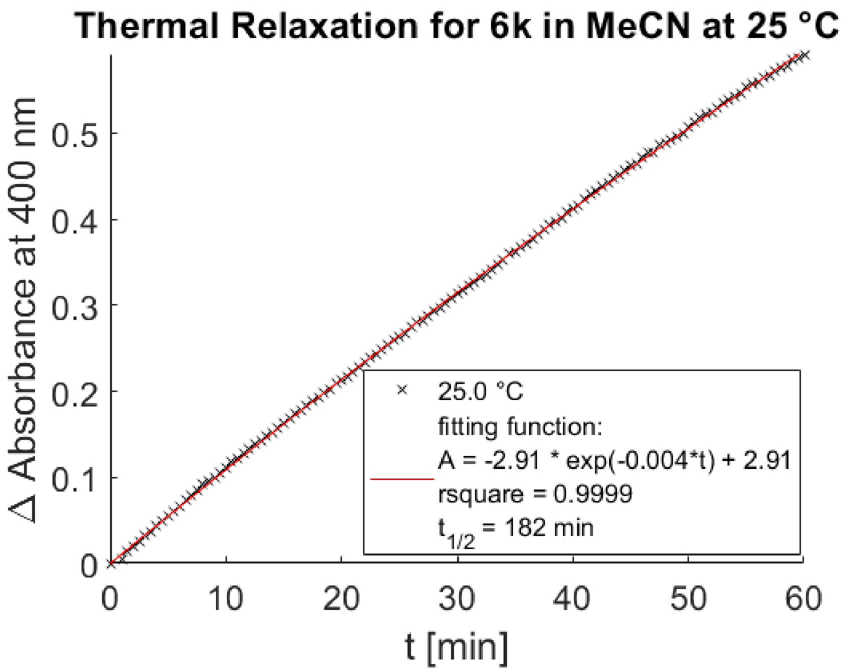

Figure S62: Increase of absorbance at 400 nm and 25 °C for a solution of **6k** in MeCN (100 µM) after reaching the photostationary state through irradiation at 385 nm. $t_{1/2}$ is the thermal half-life, calculated from the exponential fitting function assuming a first-order ratelaw.

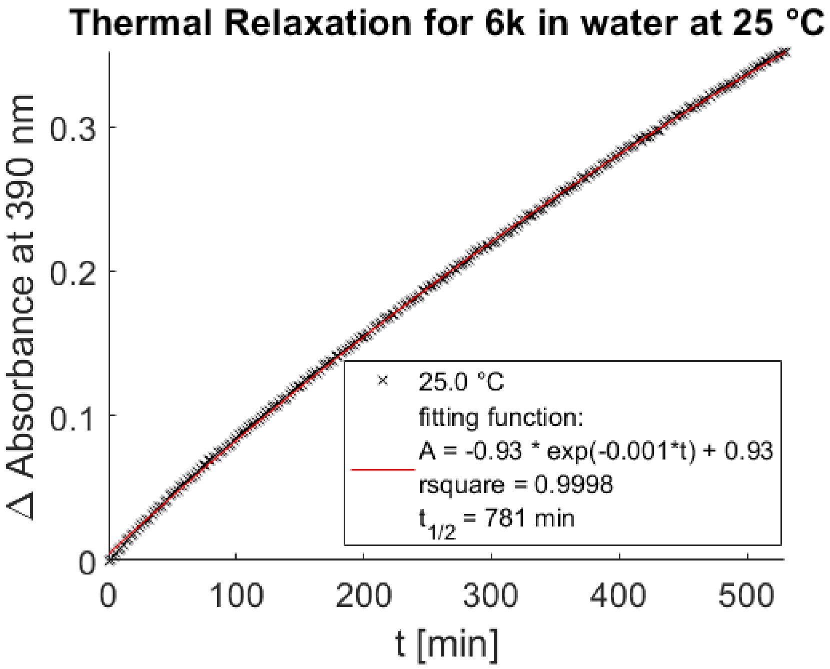

Figure S63: Increase of absorbance at 390 nm and 25 °C for a solution of **6k** in water (100 µM) after reaching the photostationary state through irradiation at 385 nm. $t_{1/2}$ is the thermal half-life, calculated from the exponential fitting function assuming a first-order ratelaw.

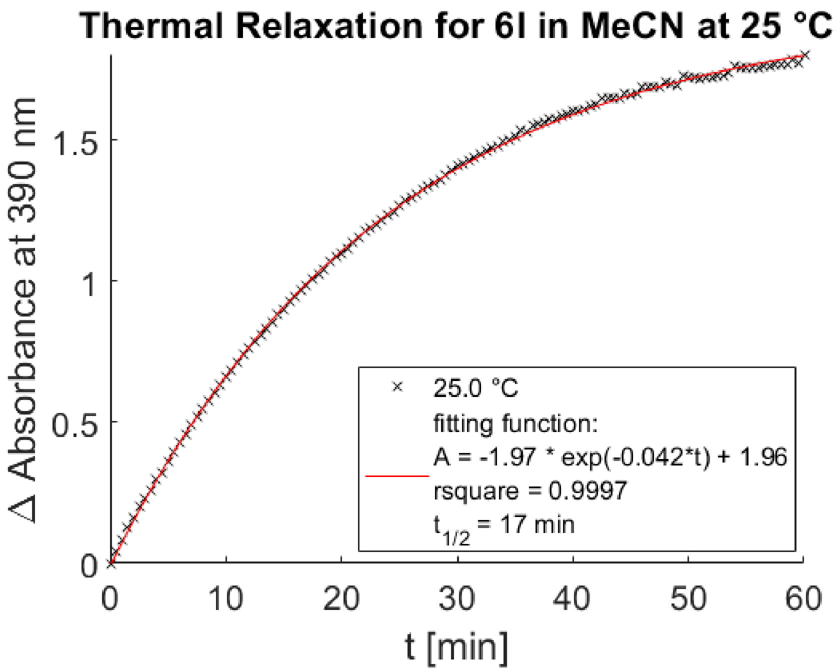

Figure S64: Increase of absorbance at 390 nm and 25 °C for a solution of **6l** in MeCN (100 μM) after reaching the photostationary state through irradiation at 385 nm. $t_{1/2}$ is the thermal half-life, calculated from the exponential fitting function assuming a first-order ratelaw.

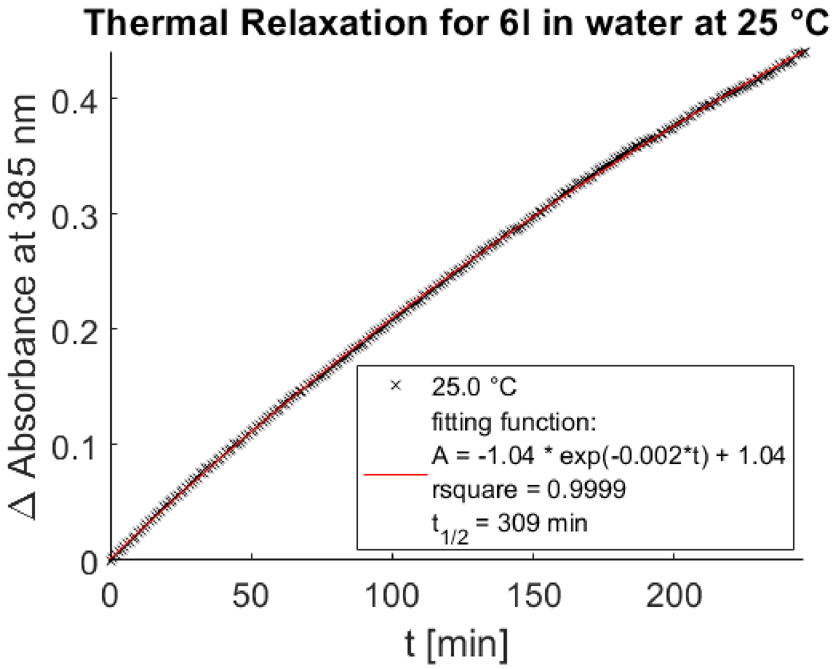

Figure S65: Increase of absorbance at 385 nm and 25 °C for a solution of **6l** in water (100 μM) after reaching the photostationary state through irradiation at 385 nm. $t_{1/2}$ is the thermal half-life, calculated from the exponential fitting function assuming a first-order ratelaw.

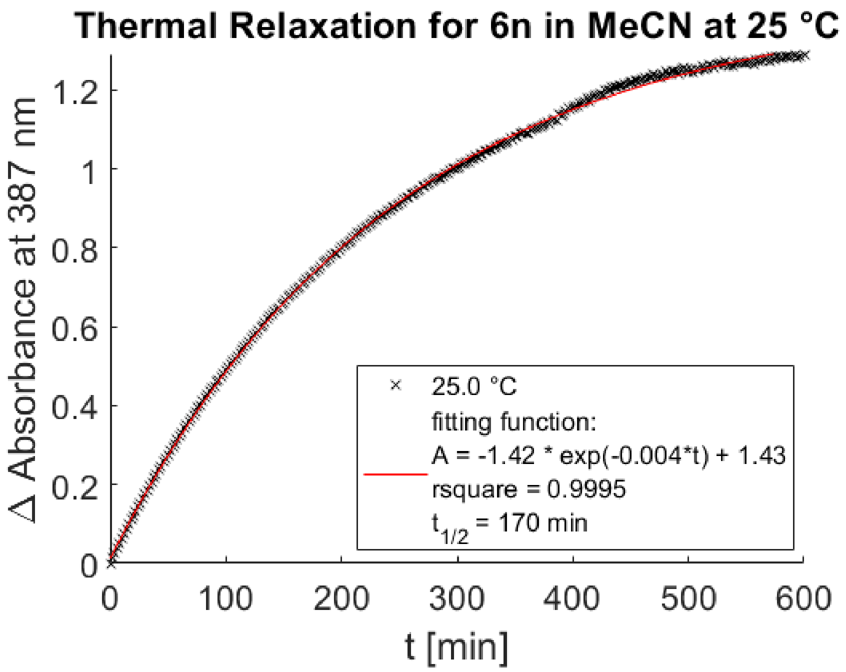

Figure S66: Increase of absorbance at 387 nm and 25 °C for a solution of **6n** in MeCN (100 µM) after reaching the photostationary state through irradiation at 385 nm. $t_{1/2}$ is the thermal half-life, calculated from the exponential fitting function assuming a first-order ratelaw.

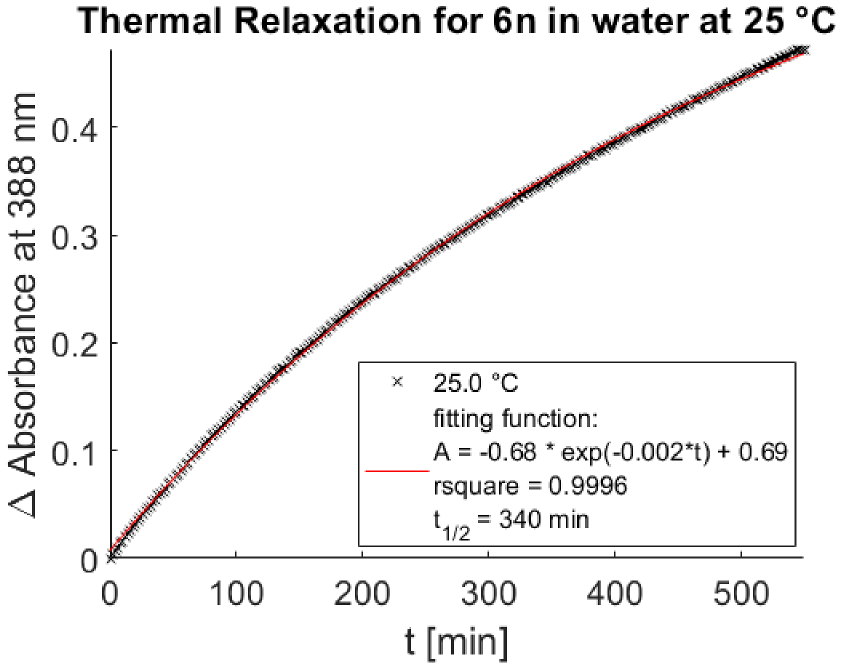

Figure S67: Increase of absorbance at 388 nm and 25 °C for a solution of **6m** in water (100 µM) after reaching the photostationary state through irradiation at 385 nm. $t_{1/2}$ is the thermal half-life, calculated from the exponential fitting function assuming a first-order ratelaw.

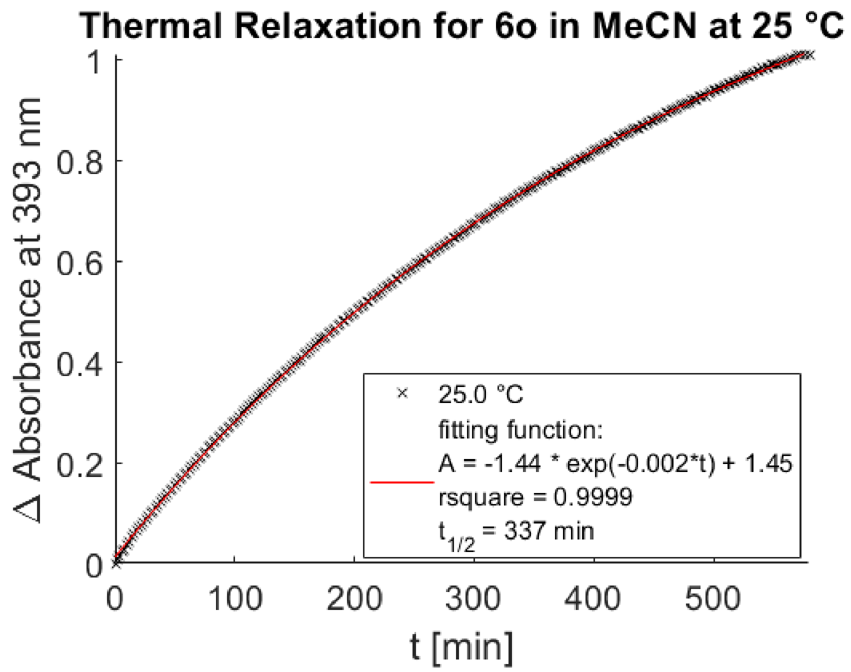

Figure S68: Increase of absorbance at 393 nm and 25 °C for a solution of **6o** in MeCN (100 μM) after reaching the photostationary state through irradiation at 385 nm. $t_{1/2}$ is the thermal half-life, calculated from the exponential fitting function assuming a first-order ratelaw.

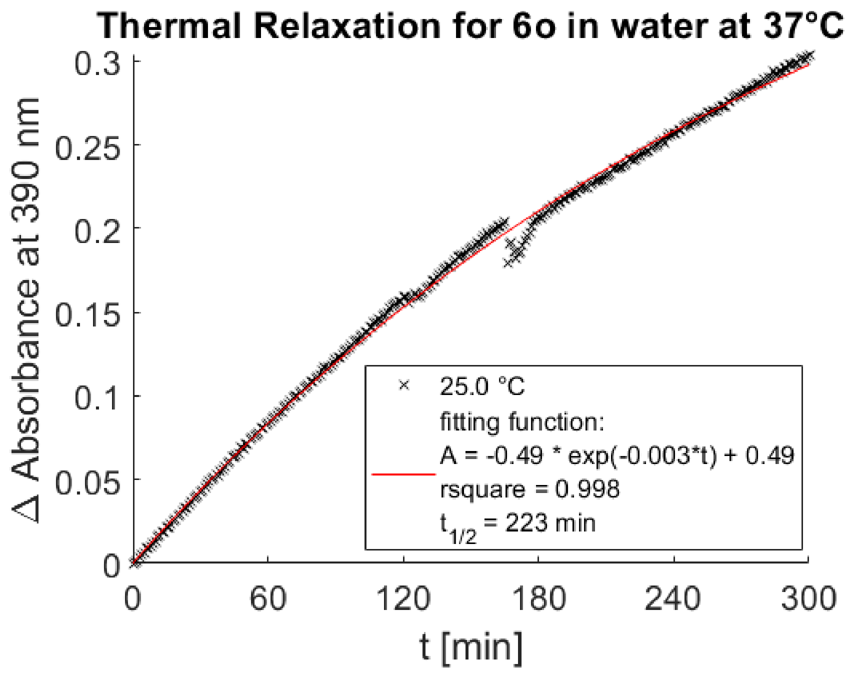

Figure S69: Increase of absorbance at 390 nm and 37 °C for a solution of **6o** in water (100 μM) after reaching the photostationary state through irradiation at 385 nm. $t_{1/2}$ is the thermal half-life, calculated from the exponential fitting function assuming a first-order ratelaw.

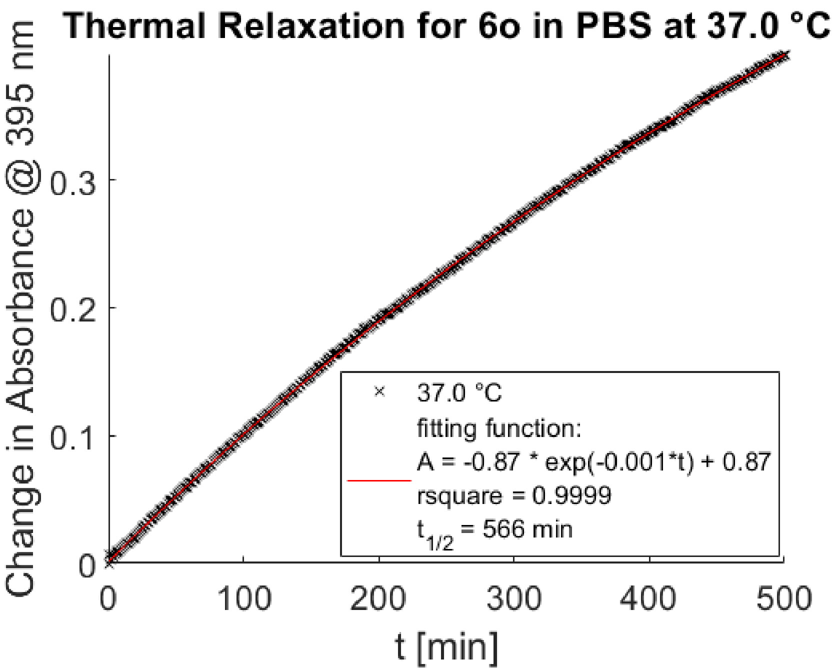

Figure S70: Increase of absorbance at 395 nm and 37 °C for a solution of **6o** (100 µM) in PBS (100 mM) after reaching the photostationary state through irradiation at 385 nm. $t_{1/2}$ is the thermal half-life, calculated from the exponential fitting function assuming a first-order ratelaw.

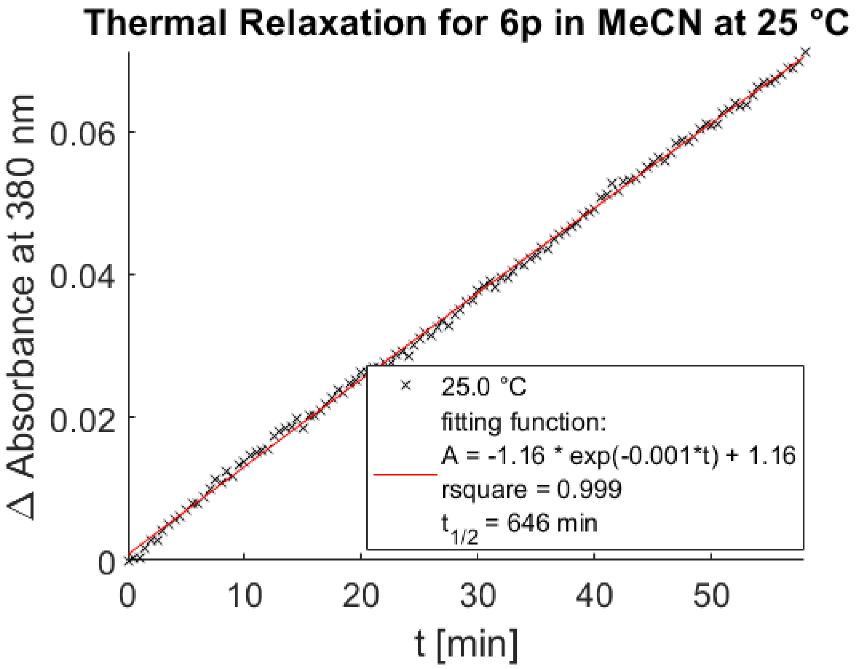

Figure S71: Increase of absorbance at 380 nm and 25 °C for a solution of **6p** in MeCN (100 µM) after reaching the photostationary state through irradiation at 385 nm. $t_{1/2}$ is the thermal half-life, calculated from the exponential fitting function assuming a first-order ratelaw.

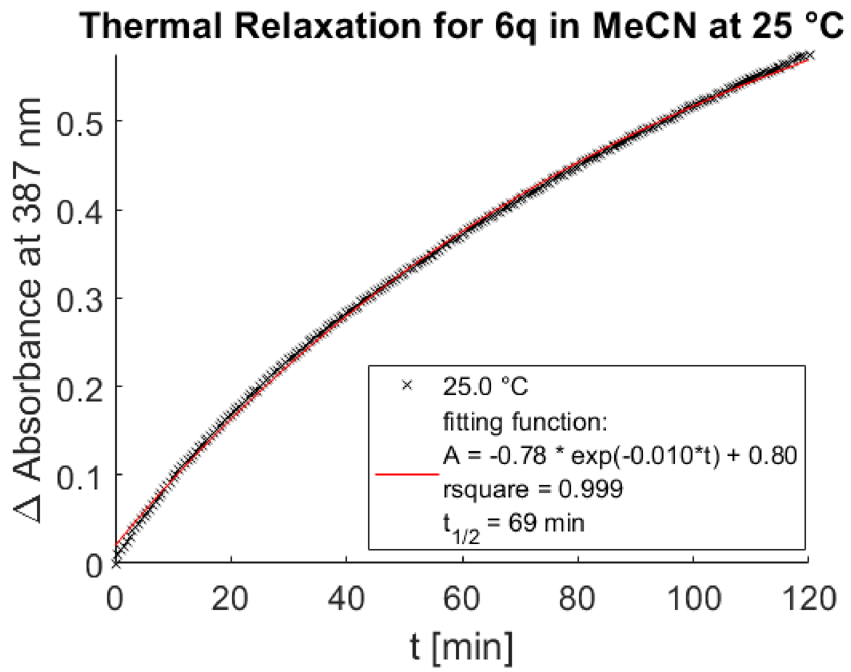

Figure S72: Increase of absorbance at 387 nm and 25 °C for a solution of **6q** in MeCN (100 µM) after reaching the photostationary state through irradiation at 385 nm. $t_{1/2}$ is the thermal half-life, calculated from the exponential fitting function assuming a first-order ratelaw.

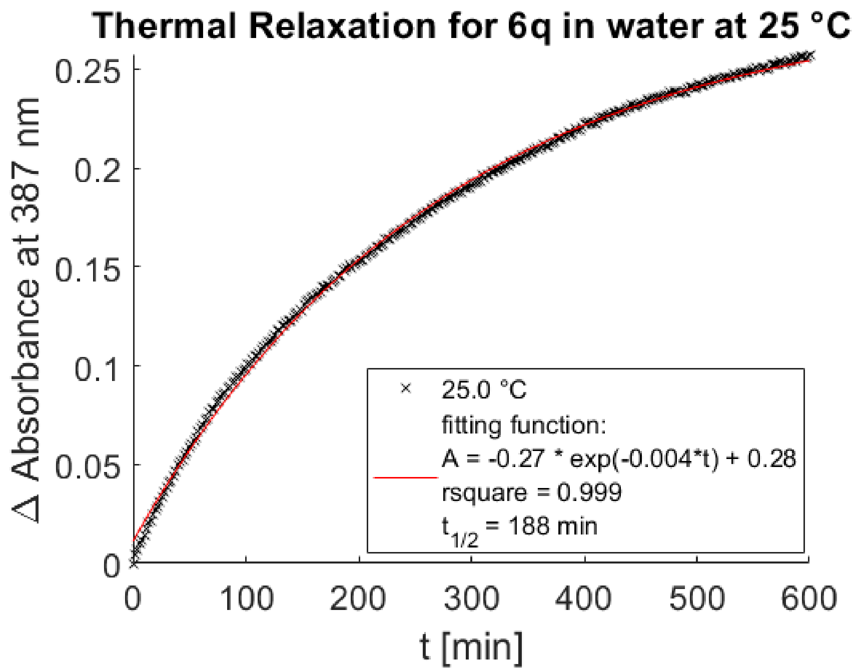

Figure S73: Increase of absorbance at 387 nm and 25 °C for a solution of **6q** in water (100 µM) after reaching the photostationary state through irradiation at 385 nm. $t_{1/2}$ is the thermal half-life, calculated from the exponential fitting function assuming a first-order ratelaw.

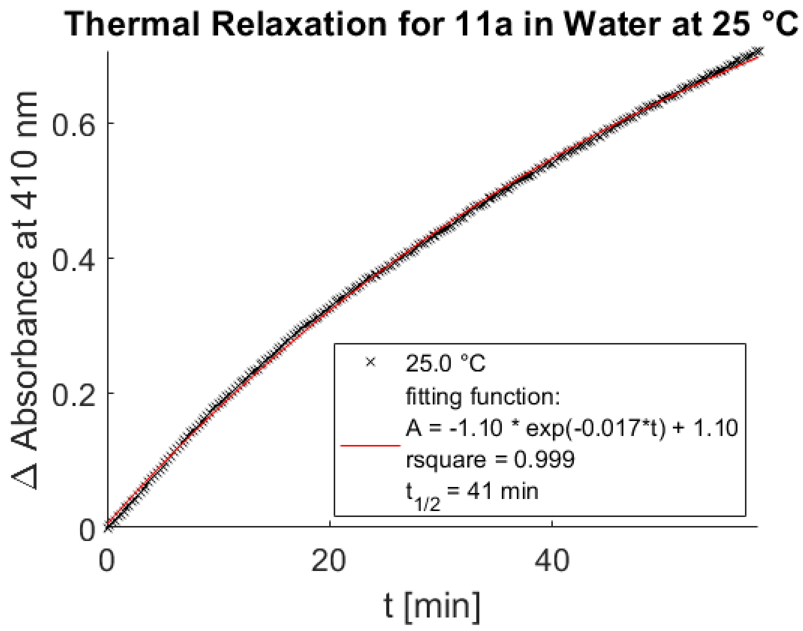

Figure S74: Increase of absorbance at 410 nm and 25 °C for a solution of **11a** in water (100 µM) after reaching the photostationary state through irradiation at 415 nm. $t_{1/2}$ is the thermal half-life, calculated from the exponential fitting function assuming a first-order ratelaw.

Stability Experiments

Dynamic Scanning Calorimetry (DSC)

DSC measurements were performed on a DSC 2500 machine (TA instruments). Samples were weighed into Tzero Hermetic Pans on a fine balance scale and sealed with Tzero Hermetic Lids. Thermograms were recorded under N_2 atmosphere at a heating rate of 10 °C/min. Thermograms are presented with exotherms going down.

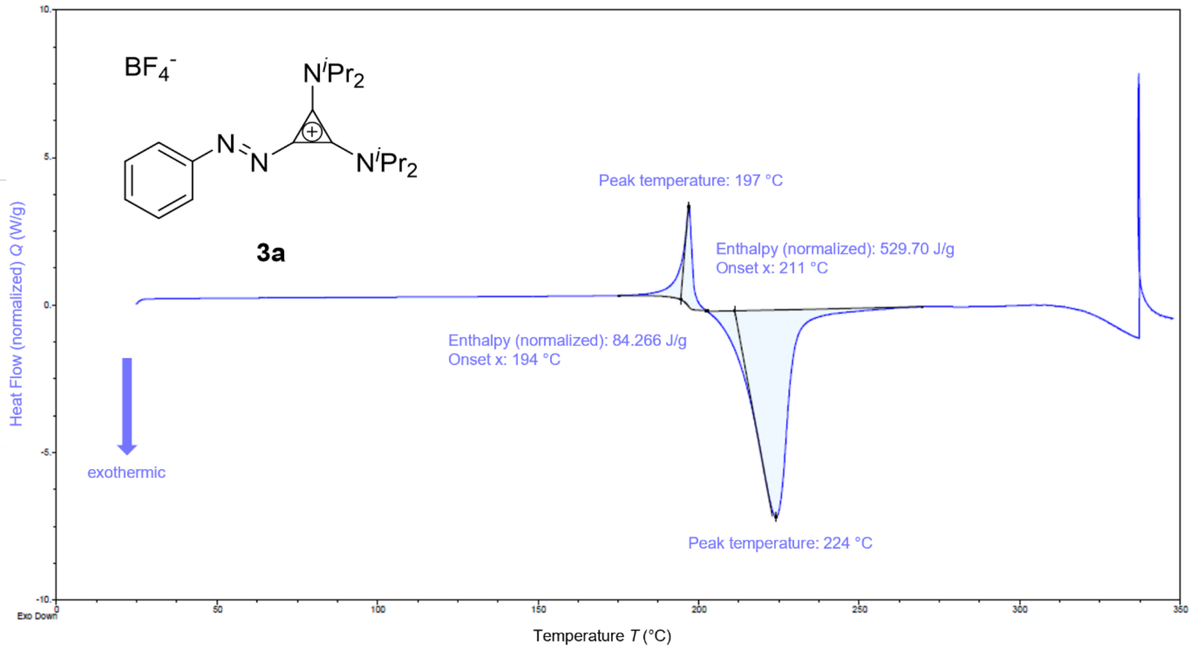

Figure S75: DSC curve of **3a** (solid at ambient temperature, 5.099 mg).

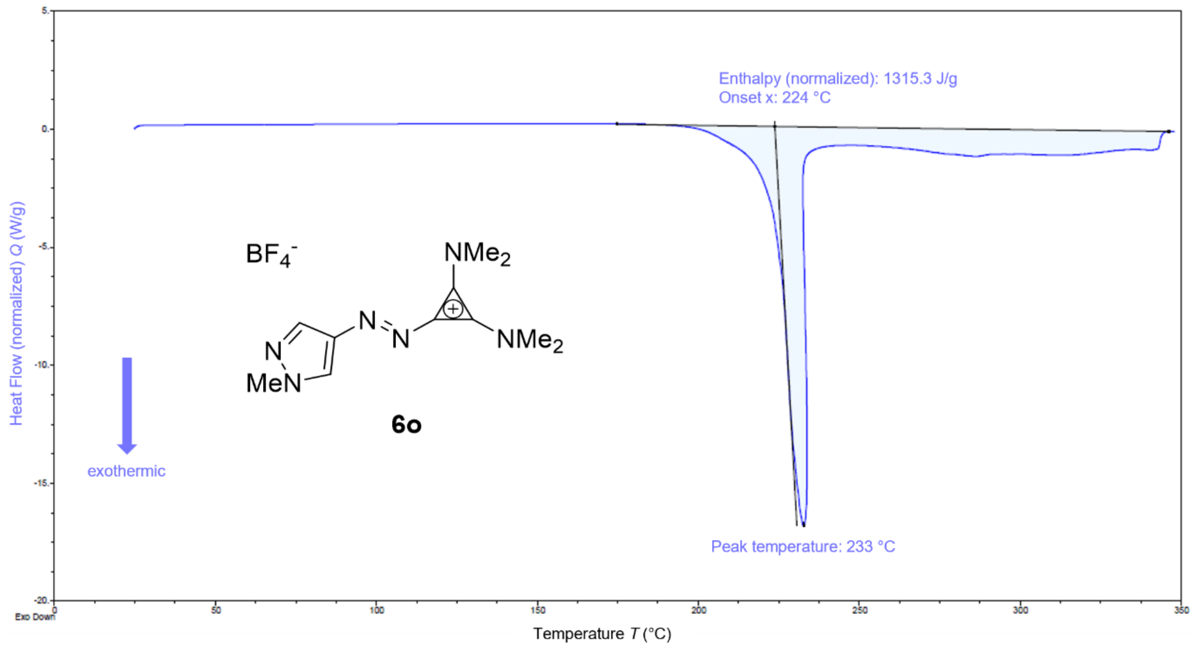

Figure S76: DSC curve of **6o** (solid at ambient temperature, 6.471 mg).

Discussion of DSC Data

Compound **3a** melts at an onset of 194 °C (peak temperature: 197 °C, normalized endothermic enthalpy: 84.3 J/g). Decomposition above 201 °C is accompanied by an exothermic process which evolves over a temperature range of about 50 °C at a maximum heat flow of 7.17 W/g (initial decomposition temperature: 201 °C, onset temperature: 211 °C, peak temperature: 224 °C, normalized exothermic enthalpy: 529.7 J/g).

Compound **6o** was chosen as an example with the lowest C/N ratio of all aryl azocyclopropenium salts prepared in this study. **6o** decomposes above 200 °C accompanied by an exothermic process which evolves over a temperature range of about 150 °C at a maximum heat flow of 16.8 W/g (initial decomposition temperature: 200 °C, onset temperature: 224 °C, peak temperature: 233 °C, normalized exothermic enthalpy: 1315.3 J/g).

Compounds **3a** and **6o** are stable well above 150 °C. Exothermic decomposition starts at 200 °C and takes place over a temperature range of 50 to 150 °C for **3a** and **6o**, respectively. For **3a** the DSC profile suggests no explosive behavior, i.e. no sharp spikes with high heat flow over a short heating range. In the case of **6o**, the heat release creates a larger maximum heat flow and sharper initial peak profile. However, the total heat release of 1315.3 J/g for **6o** occurs over a temperature range that is 100 °C larger than for **3a**. For the initial decomposition event of **6o**, i.e. the temperature range of 50 °C upon the onset of decomposition, the heat release amounts to 890 J/g.

Yoshida Correlation Analysis

To assess the potential explosive behavior of **3a** and **6o**, the obtained DSC data were analyzed applying Yoshida's correlation[10,11] and its more recent adaption by Pfizer (Figure S76).[12] Both models predict shock sensitivity and explosive behavior from DSC measurements based on the onset temperature of exothermic decomposition and the associated heat flow. The threshold defines a mathematical function which indicates potentially explosive materials (values above the curve).

The DSC data points for **3a** are located below the curves and thus no shock sensitivity or explosive behavior is to be expected.

The data points for **6o,** based on the total heat evolution between 200 and 350 °C, are located below Yoshida's correlation curves to predict shock sensitive or explosive properties. However, **6o** is marked potentially shock sensitive and explosive applying Pfizer's modification, which involves a 25% lower energy threshold (Figure S76, in grey). It should be considered that the total heat release amounts over a heating range of 150 °C with a spike at 233 °C. In contrast, explosive compounds are characterized by a large heat evolution over a relatively short

heating period.[35] Thus, the initial decomposition event between 200 °C and the offset of the first spike at 250 °C is considered more suitable to predict explosive properties of **6o**. In this temperature range the heat evolution amounts to 890 J/g. Accordingly, the thermal data are located below Yoshida's correlation curve and the more conservative model by Pfizer (Figure S76, in yellow).

Although **3a** and **6o** are not marked as shock sensitive or potentially explosive, both compounds should be handled with care as their decomposition above 200 °C is exotherm.

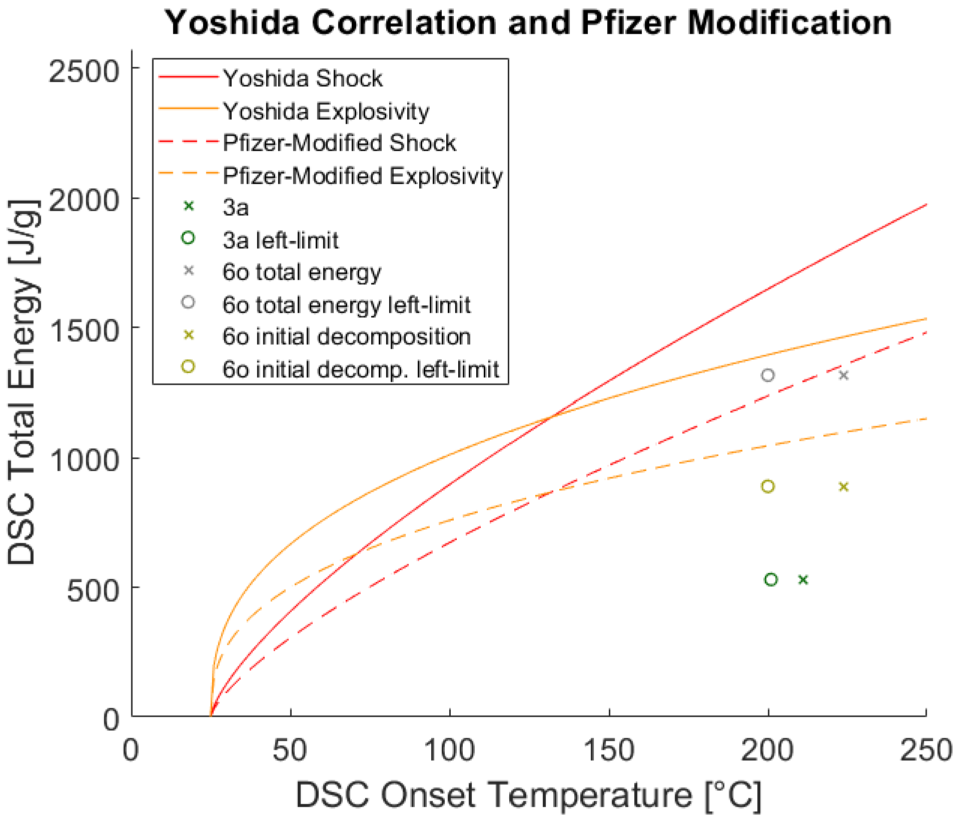

Figure 77: Yoshida's and Pfizer's correlation plot for thermal data of **3a** and **6o**.

Thermal Stability of 6o in D_2O

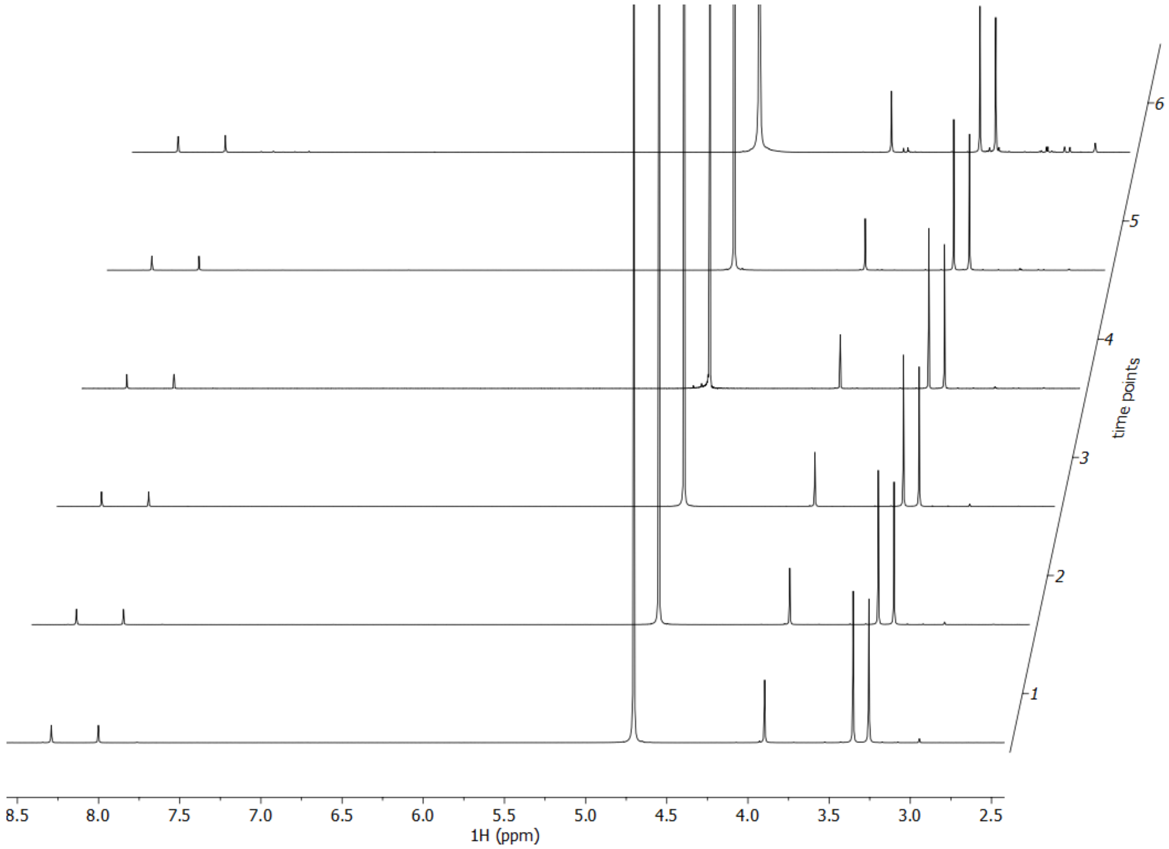

Figure S78: ^{1}H NMR spectra of **6o** in D_2O after [1] 15 min, [2] 42 h, [3] 60 h, [4] 21 d, [5] 5 weeks, and [6] 7 months in the dark under ambient conditions.

Stability in Different Biologically Relevant Media

To assess the stability of aryl azocyclopropenium salts in different biologically relevant media, stock solutions of the respective compounds in water were diluted to the indicated concentration by addition of respective media in a quartz cuvette (d = 1 cm). The cuvette was placed on a UV-vis spectrometer and the absorbance monitored over time at the wavelength indicated. Media containing glutathione (GSH) were degassed by bubbling N_2 through the solution for at least 1 h prior mixing with the stock solutions of aryl azocyclopropenium salts in water.[36]

PBS buffer

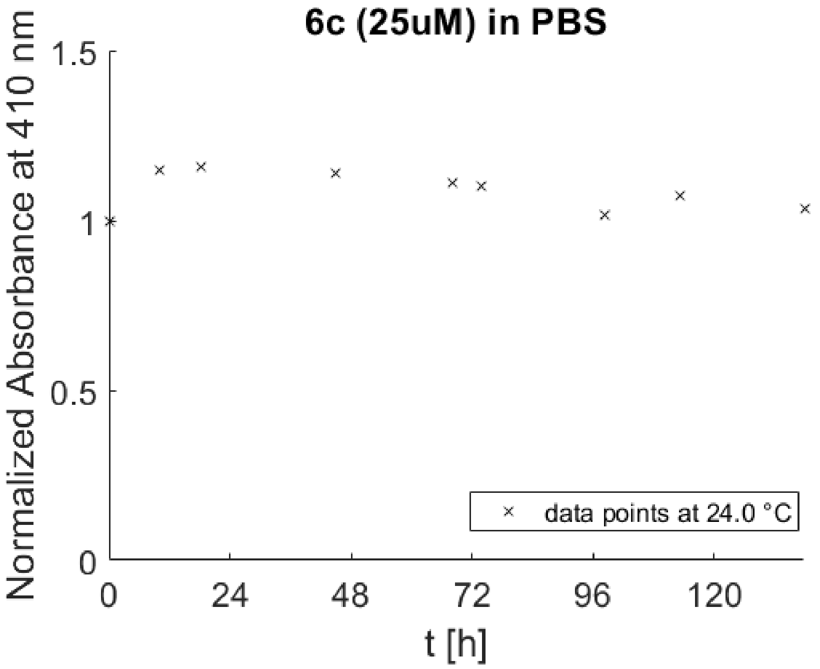

Figure S79: Absorbance at 410 nm for a solution of **6c** (25 μM) in a mixture of 1X PBS (pH = 7.4) and water (19:1).

Luria Broth Miller (Bacterial Growth Medium)

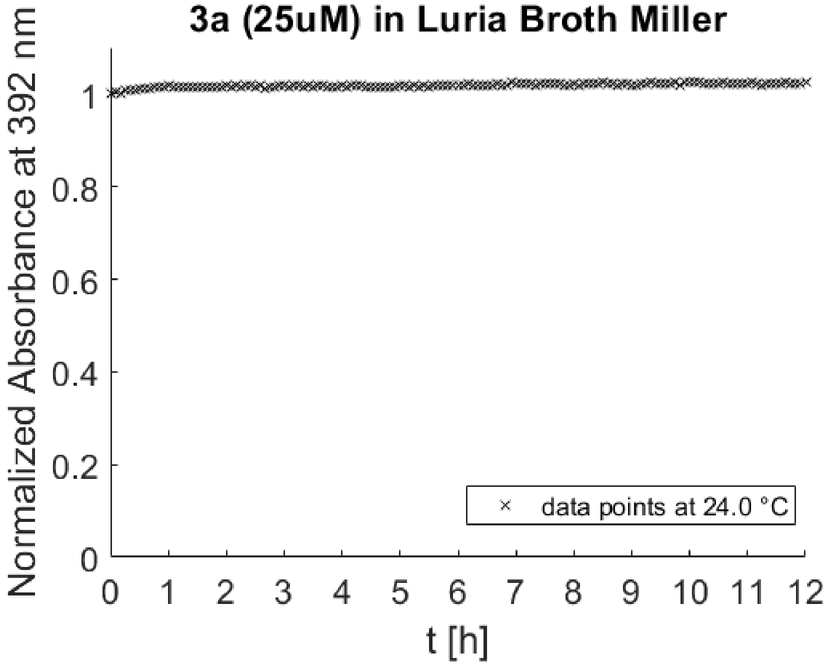

Figure S80: Absorbance at 392 nm for a solution of **3a** (25 µM) in a mixture of Luria Broth Miller and water (19:1).

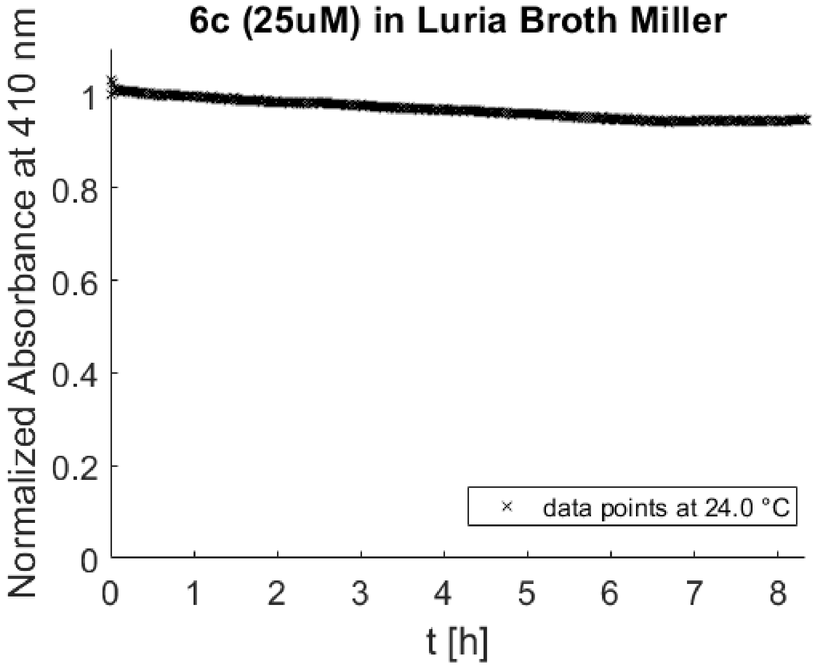

Figure S81: Absorbance at 410 nm for a solution of **6c** (25 µM) in a mixture of Luria Broth Miller and water (19:1).

PBS and Glutathione

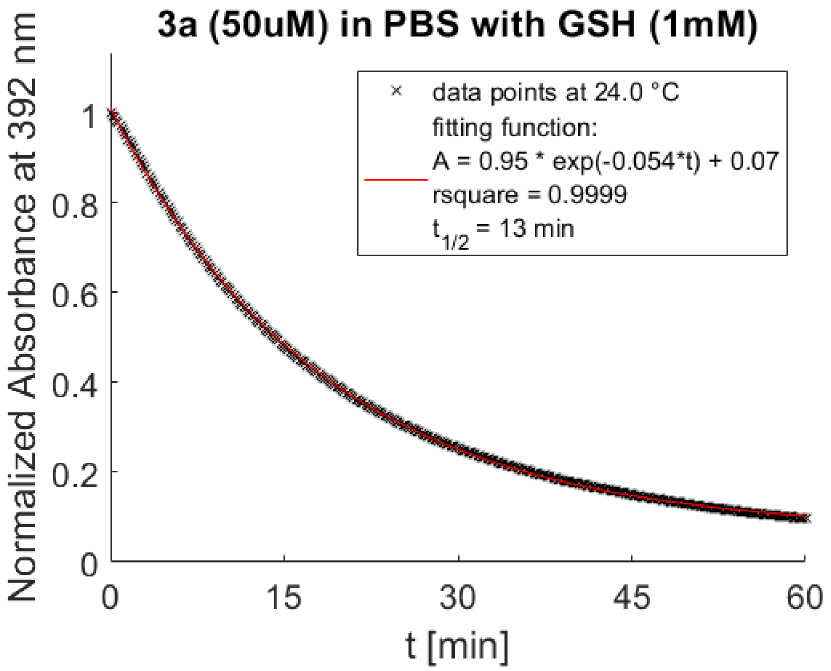

Figure S82: Absorbance at 392 nm for a solution of **3a** (50 μM) in a mixture of PBS buffer (degassed, containing 1 mM glutathione) and water (9:1).

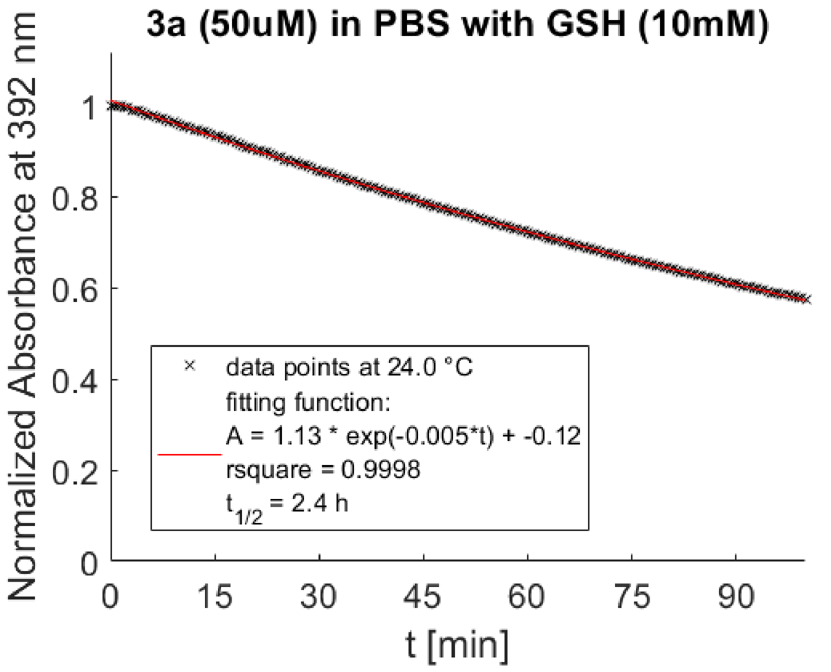

Figure S83: Absorbance at 392 nm for a solution of **3a** (50 μM) in a mixture of PBS buffer (degassed, containing 10 mM glutathione) and water (9:1).

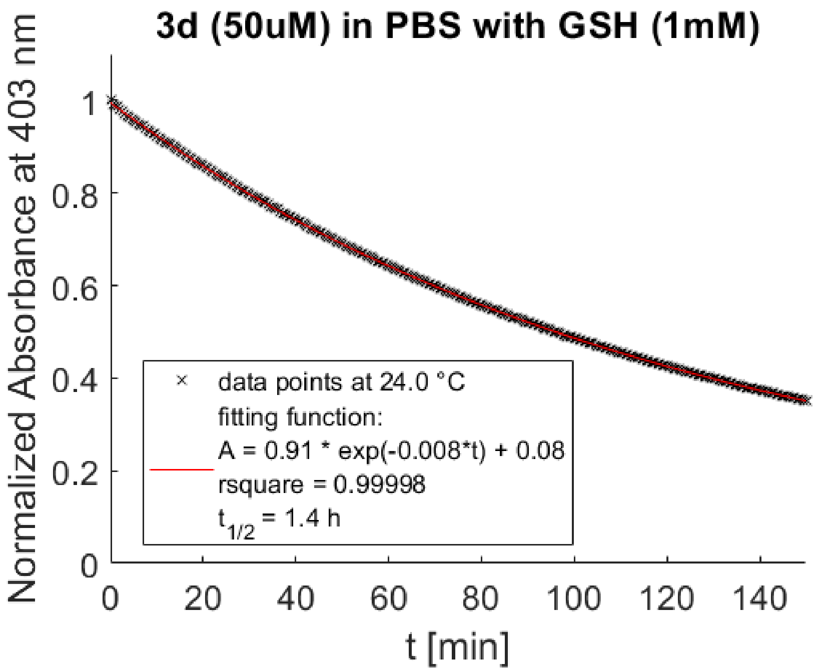

Figure S84: Absorbance at 403 nm for a solution of **3d** (50 µM) in a mixture of PBS buffer (degassed, containing 1 mM glutathione) and water (9:1).

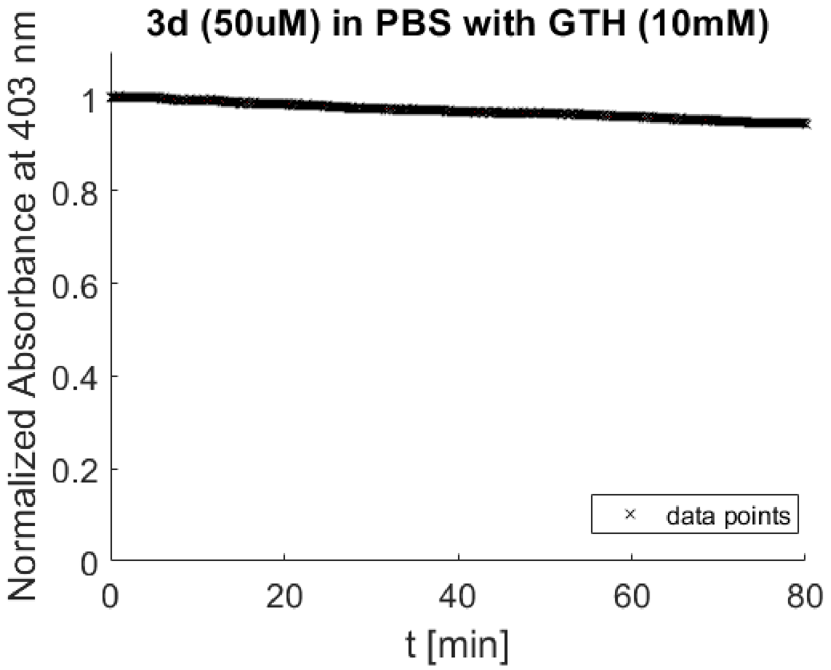

Figure S85: Absorbance at 403 nm for a solution of **3d** (50 µM) in a mixture of PBS buffer (degassed, containing 10 mM glutathione) and water (9:1).

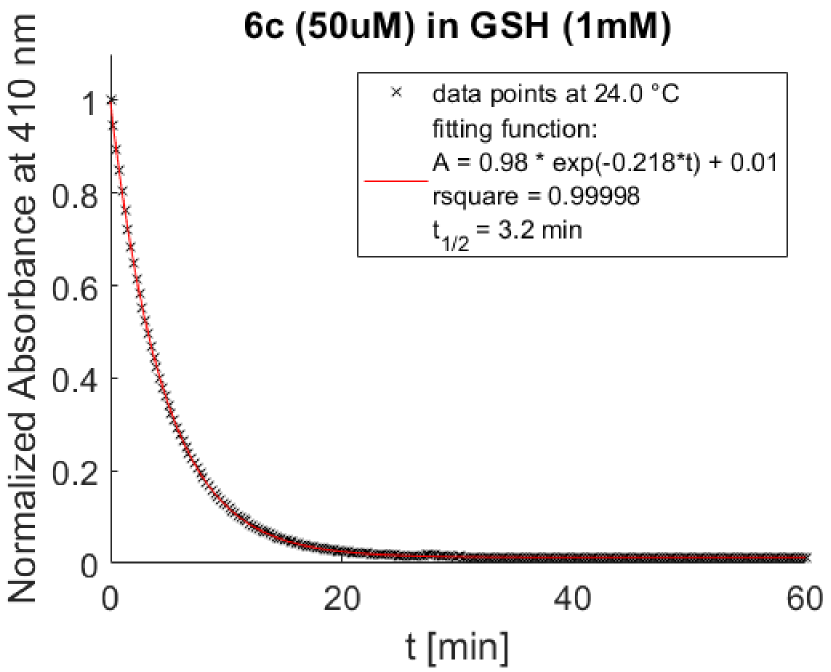

Figure S86 Absorbance at 410 nm for a solution of **6c** (50 μM) in a mixture of PBS buffer (degassed, containing 1 mM glutathione) and water (9:1).

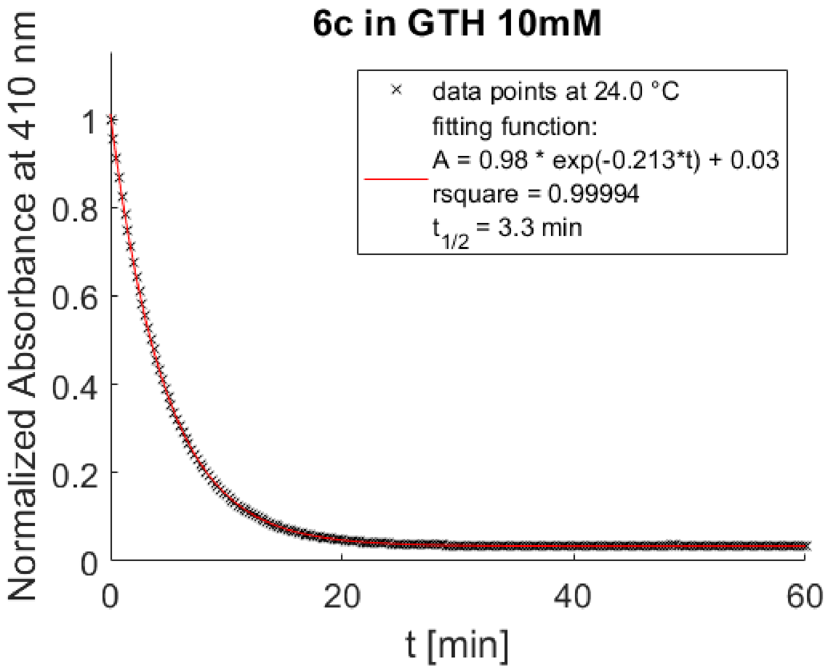

Figure S87: Absorbance at 410 nm for a solution of **6c** (50 μM) in a mixture of PBS buffer (degassed, containing 10 mM glutathione) and water (9:1).

Photostability Experiments

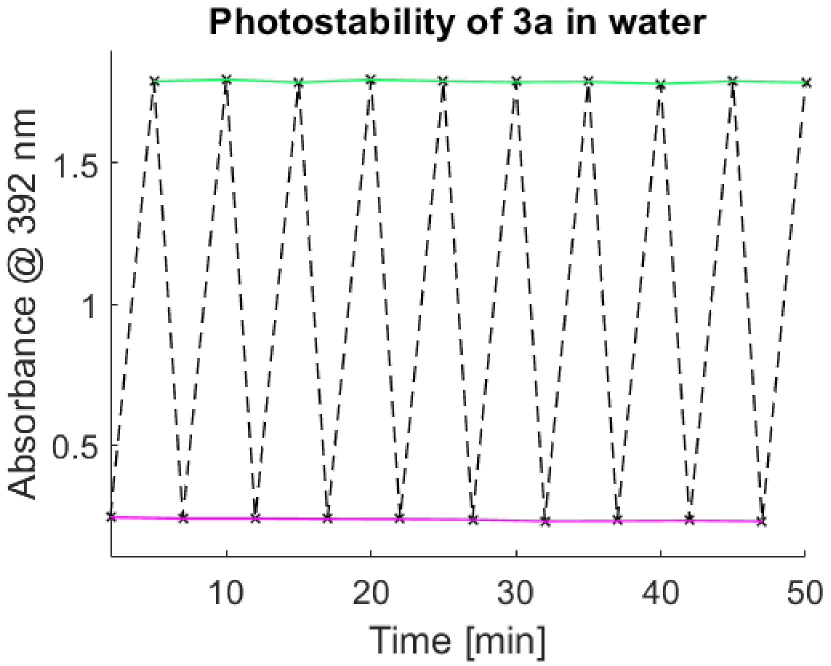

Figure S88: Absorbance at 392 nm for a solution of **3a** (100 µM in water) when alternating irradiation at 385 nm (2 min) and 505 nm (3 min); magenta curve: PSS after irradiation at 385 nm, green curve: PSS after irradiation at 505 nm.

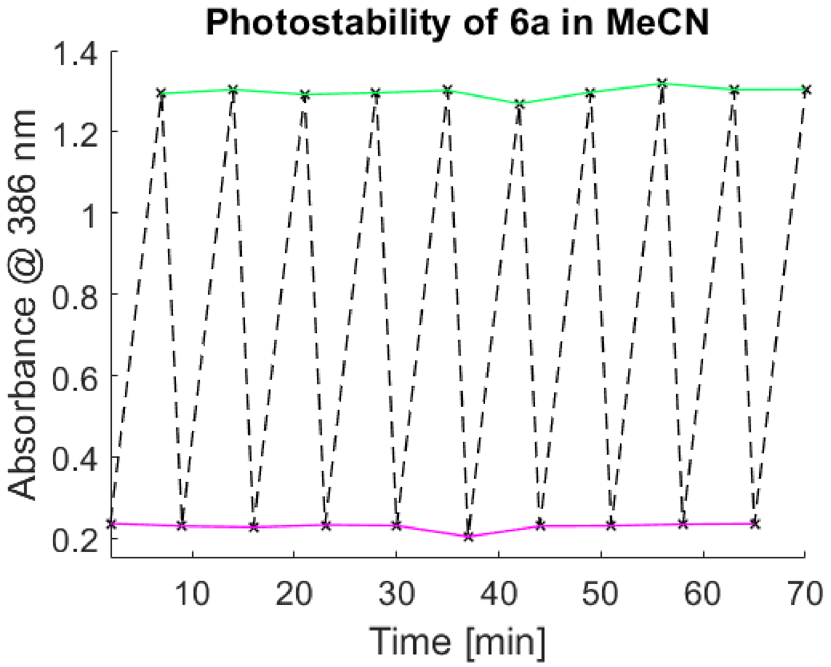

Figure S89: Absorbance at 386 nm for a solution of **6a** (100 µM in MeCN) when alternating irradiation at 385 nm (2 min) and 505 nm (5 min); magenta curve: PSS after irradiation at 385 nm, green curve: PSS after irradiation at 505 nm.

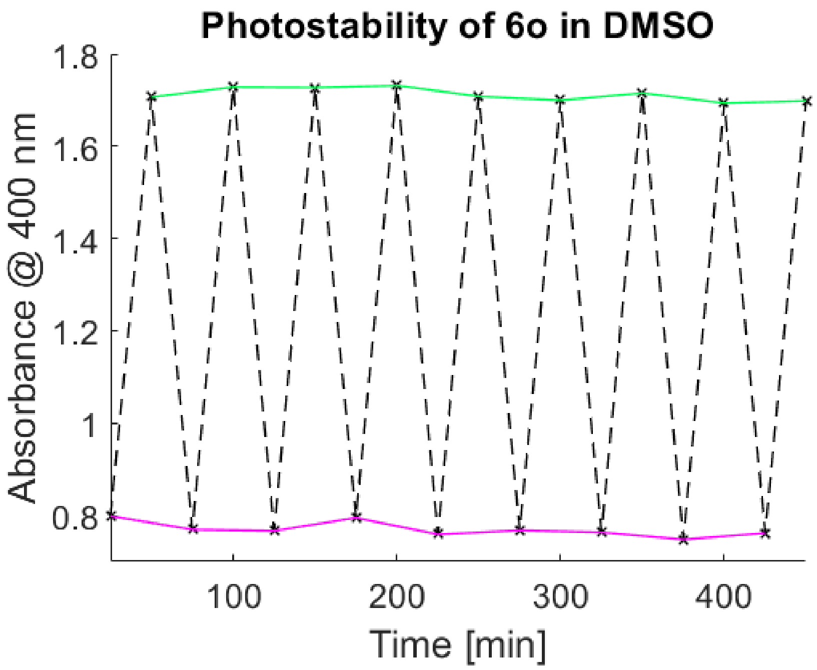

Figure S90: Absorbance at 400 nm for a solution of **6o** (100 µM in DMSO) when alternating irradiation at 415 nm (25 min) and 505 nm (25 min); magenta curve: PSS after irradiation at 415 nm, green curve: PSS after irradiation at 505 nm; irradiation times do not equal the time required to reach the PSS.

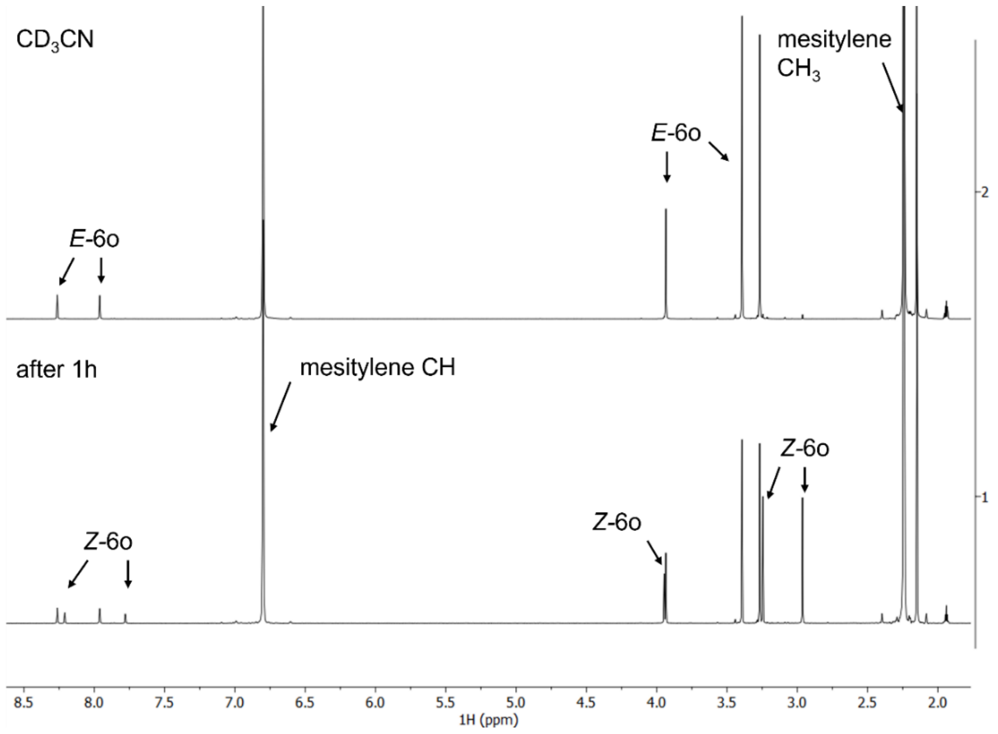

Figure S91: ^{1}H NMR spectra of **6o** in CD_3CN before (2, top) and after (1, bottom) irradiation in the blue light photoreactor (446 nm) for 1 h, comparing the integral intensity of the signals for *E*-**6o** and *Z*-**6o** with mesitylene as internal standard shows no loss of **6o**.

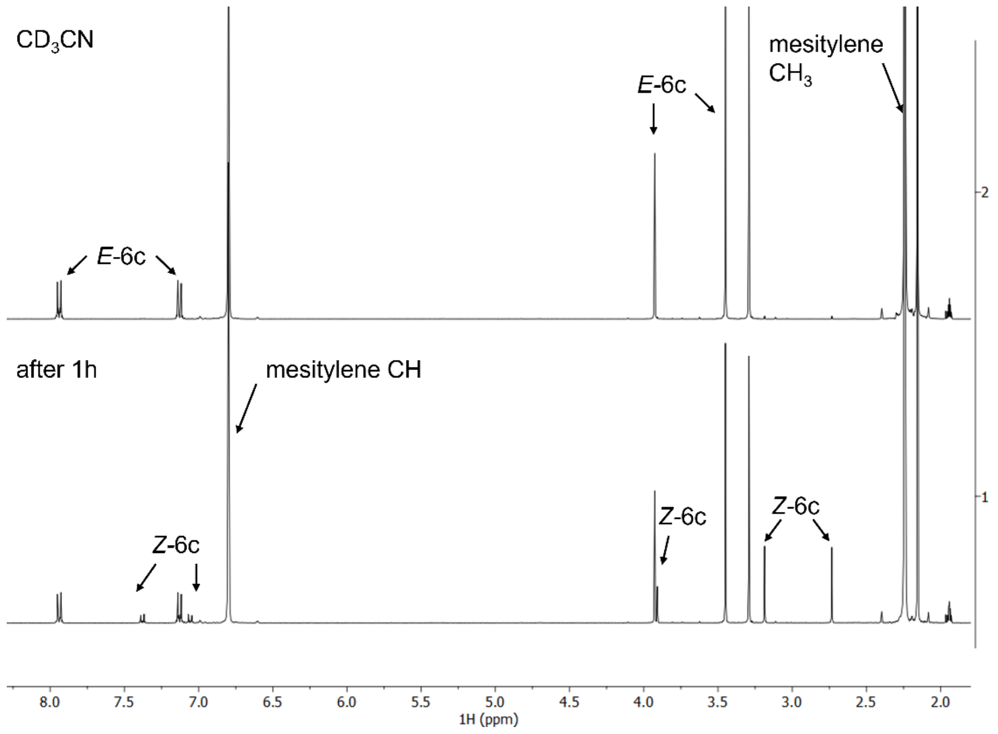

Figure S92: ^{1}H NMR spectra of **6c** in CD_3CN before (2, top) and after (1, bottom) irradiation in the blue light photoreactor (446 nm) for 1 h, comparing the integral intensity of the signals for *E*-**6c** and *Z*-**6c** with mesitylene as internal standard shows no loss of **6c**.

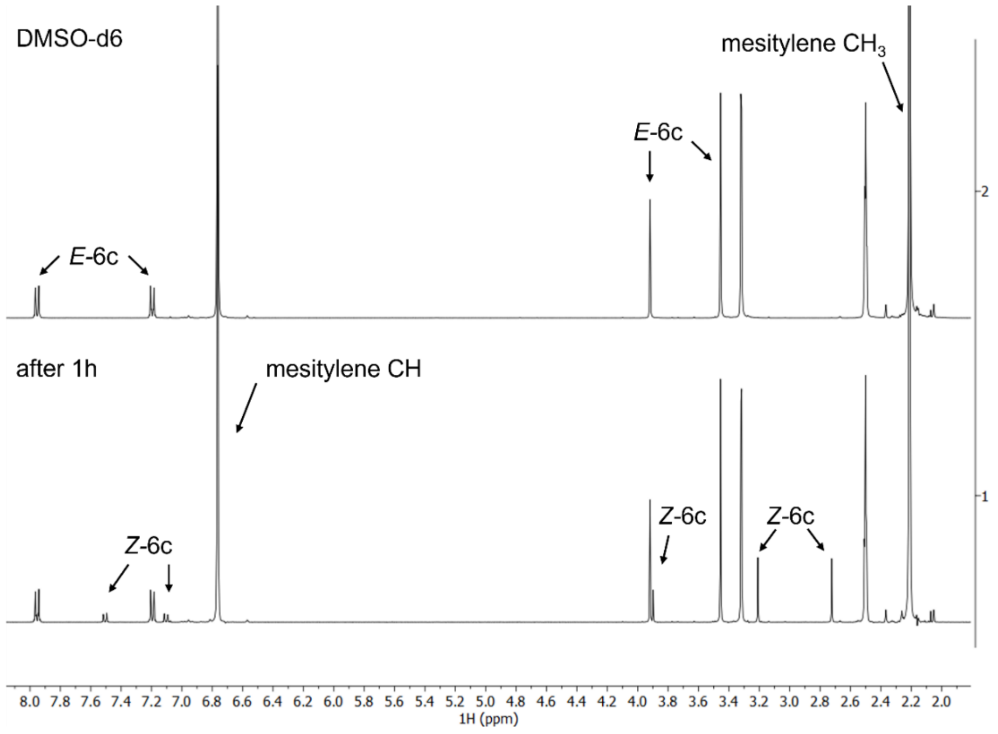

Figure S93: ^{1}H NMR spectra of **6c** in DMSO-d6 before (2, top) and after (1, bottom) irradiation in the blue light photoreactor (446 nm) for 1 h, comparing the integral intensity of the signals for *E*-**6c** and *Z*-**6c** with mesitylene as internal standard shows no loss of **6c**.

NMR Spectra

^{1}H NMR, ^{13}C NMR, and ^{19}F NMR spectrum of compound **1a**

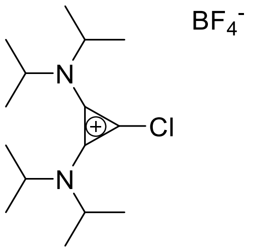

1a

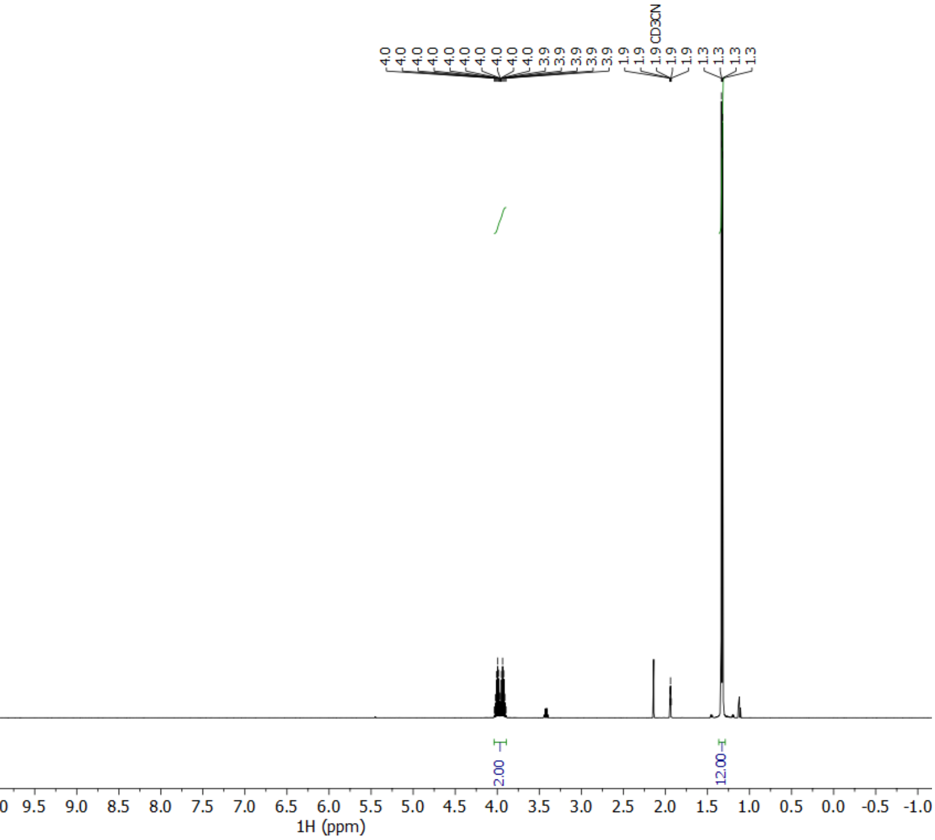

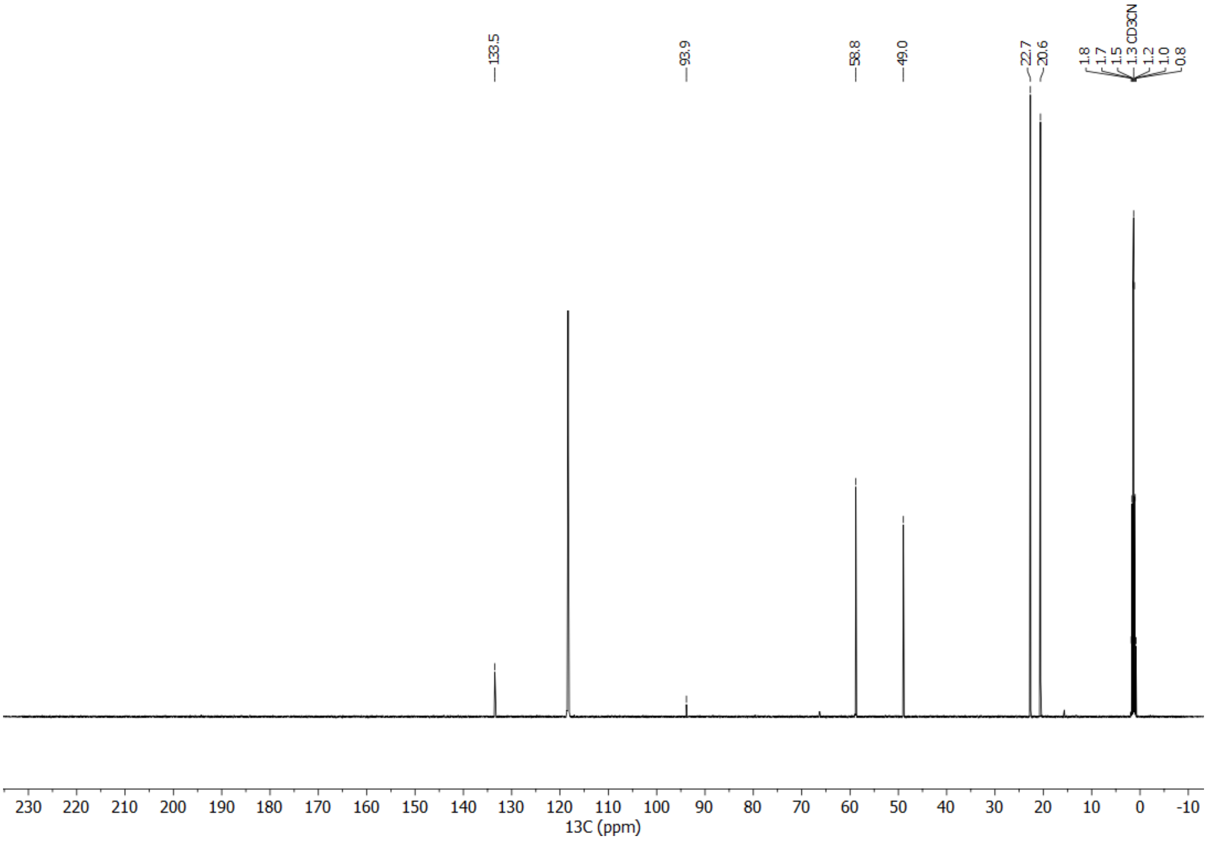

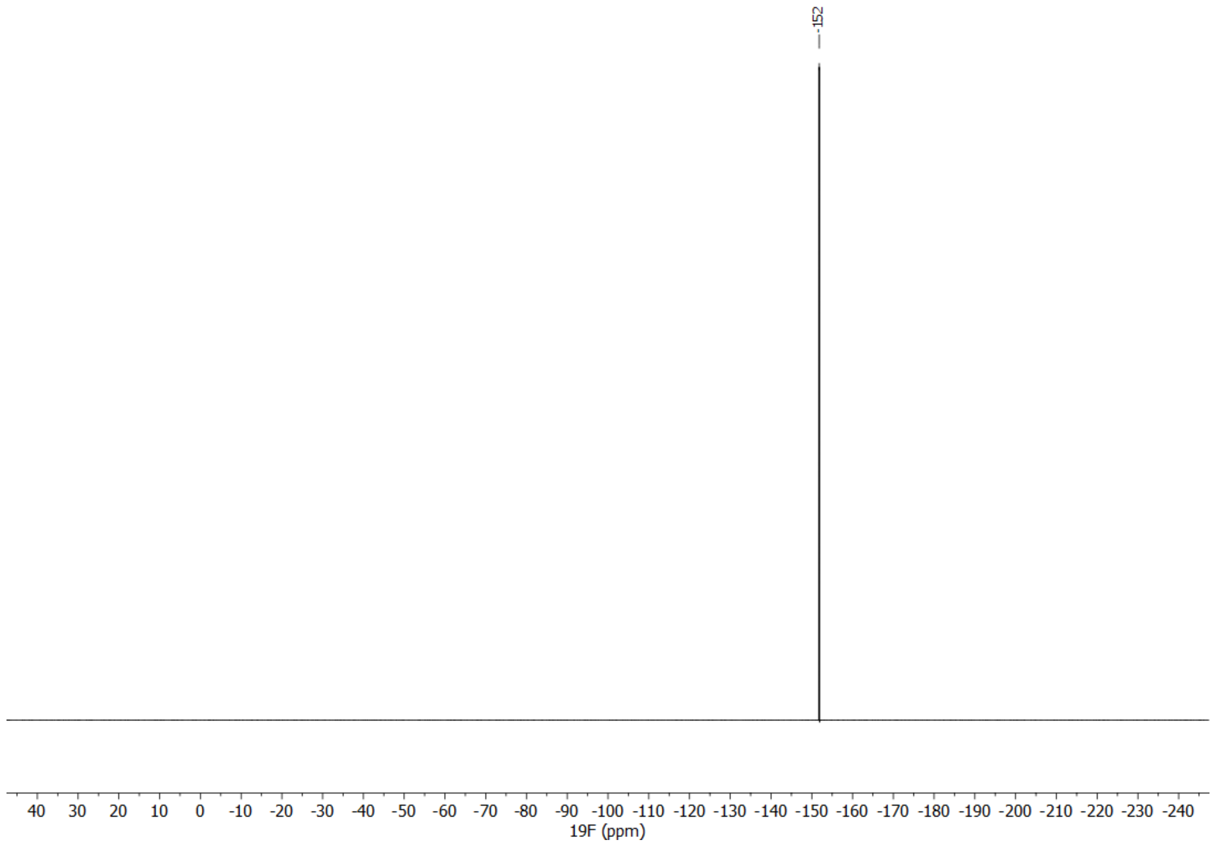
-152
40 30 20 10 0 -10 -20 -30 -40 -50 -60 -70 -80 -90 -100 -110 -120 -130 -140 -150 -160 -170 -180 -190 -200 -210 -220 -230 -240
19F (ppm)

^{1}H NMR and ^{13}C NMR spectrum of compound **1b**

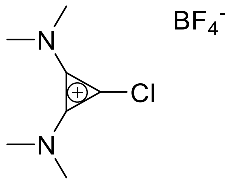

1b

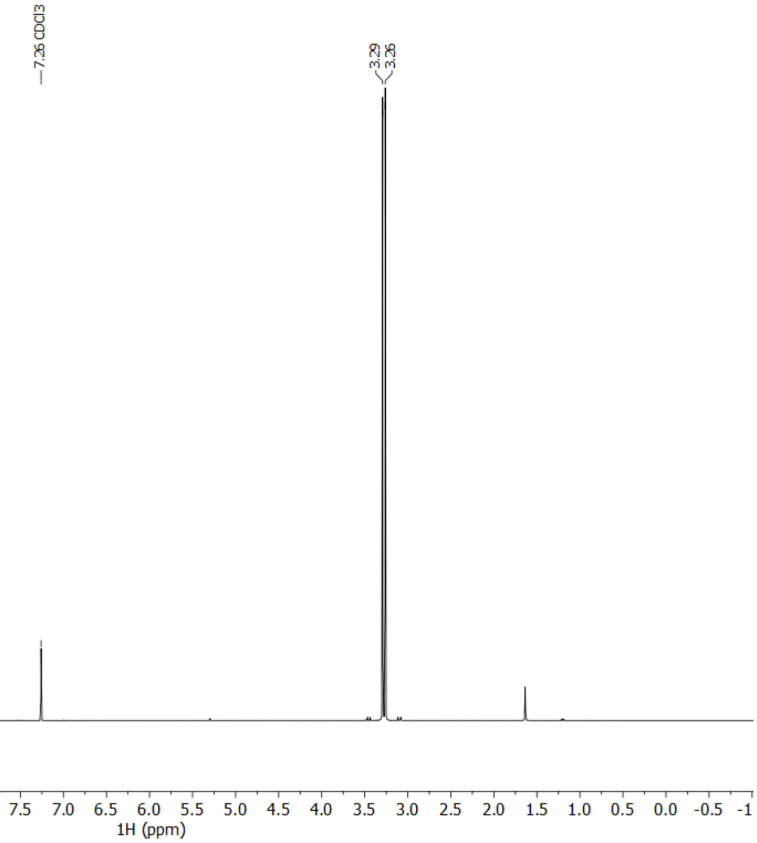

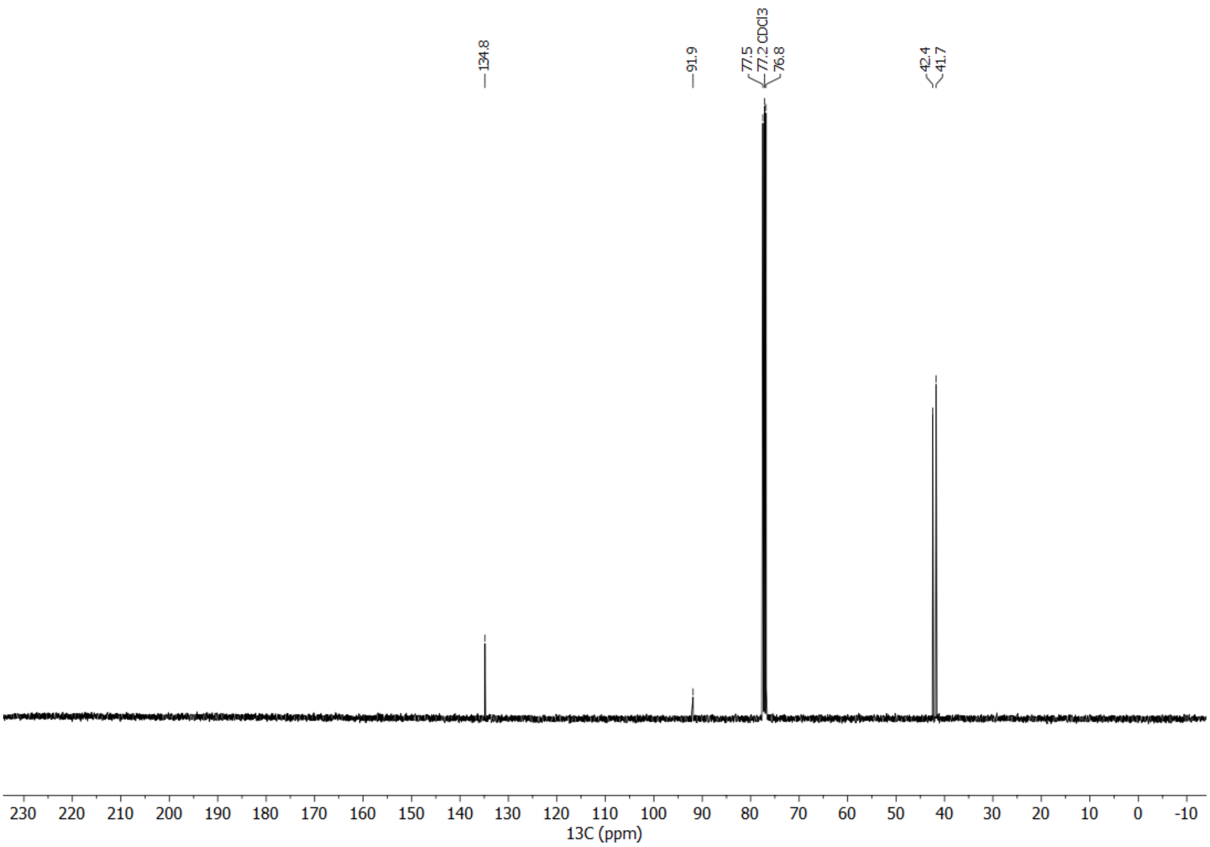

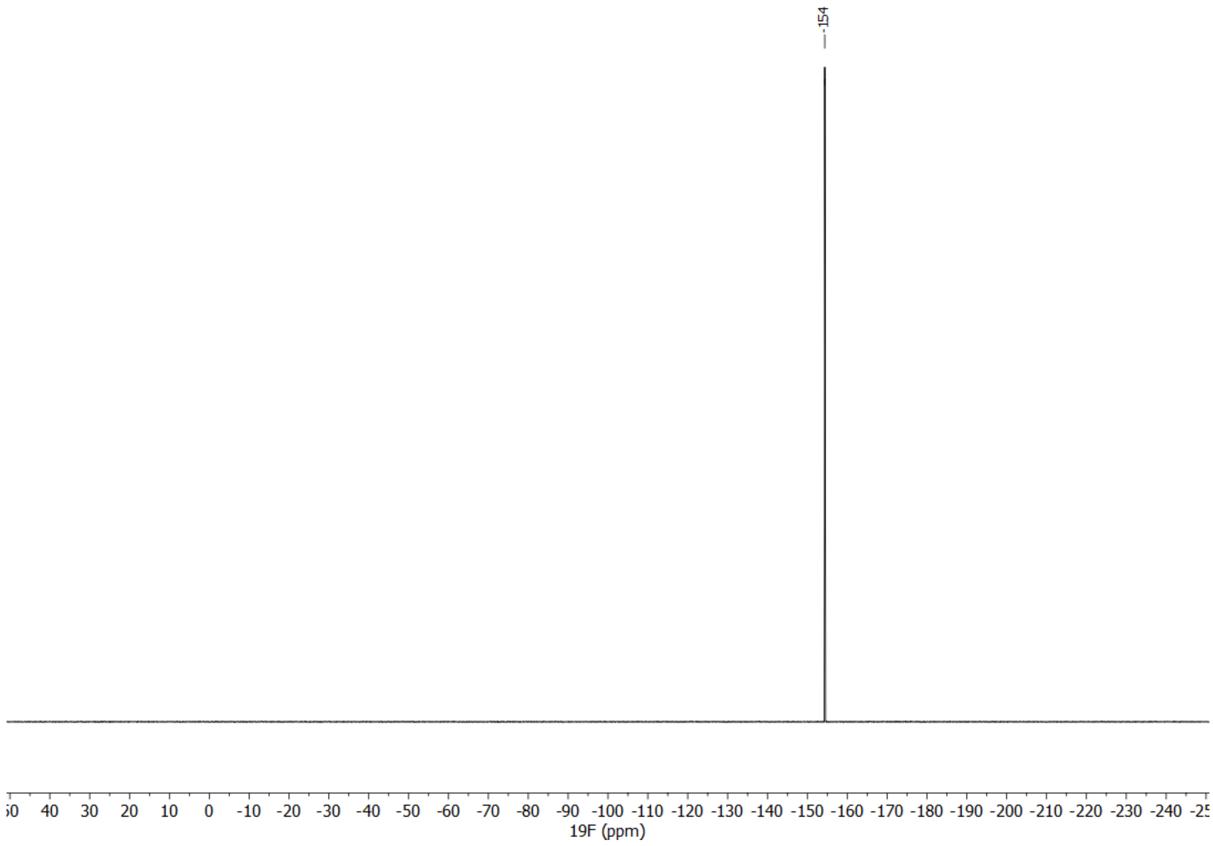

-154
50 40 30 20 10 0 -10 -20 -30 -40 -50 -60 -70 -80 -90 -100 -110 -120 -130 -140 -150 -160 -170 -180 -190 -200 -210 -220 -230 -240 -25
19F (ppm)

^{1}H NMR, ^{13}C NMR, and ^{19}F NMR spectrum of compound **3a**

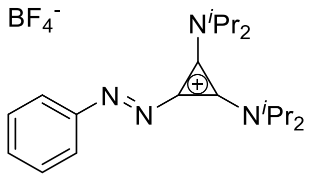

3a

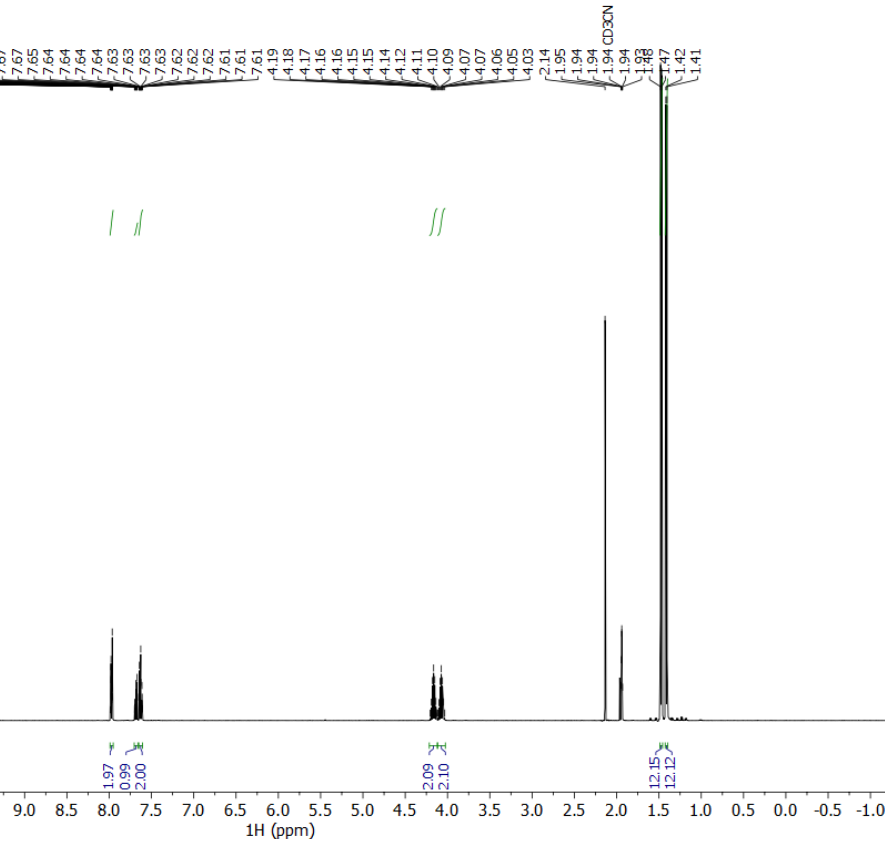

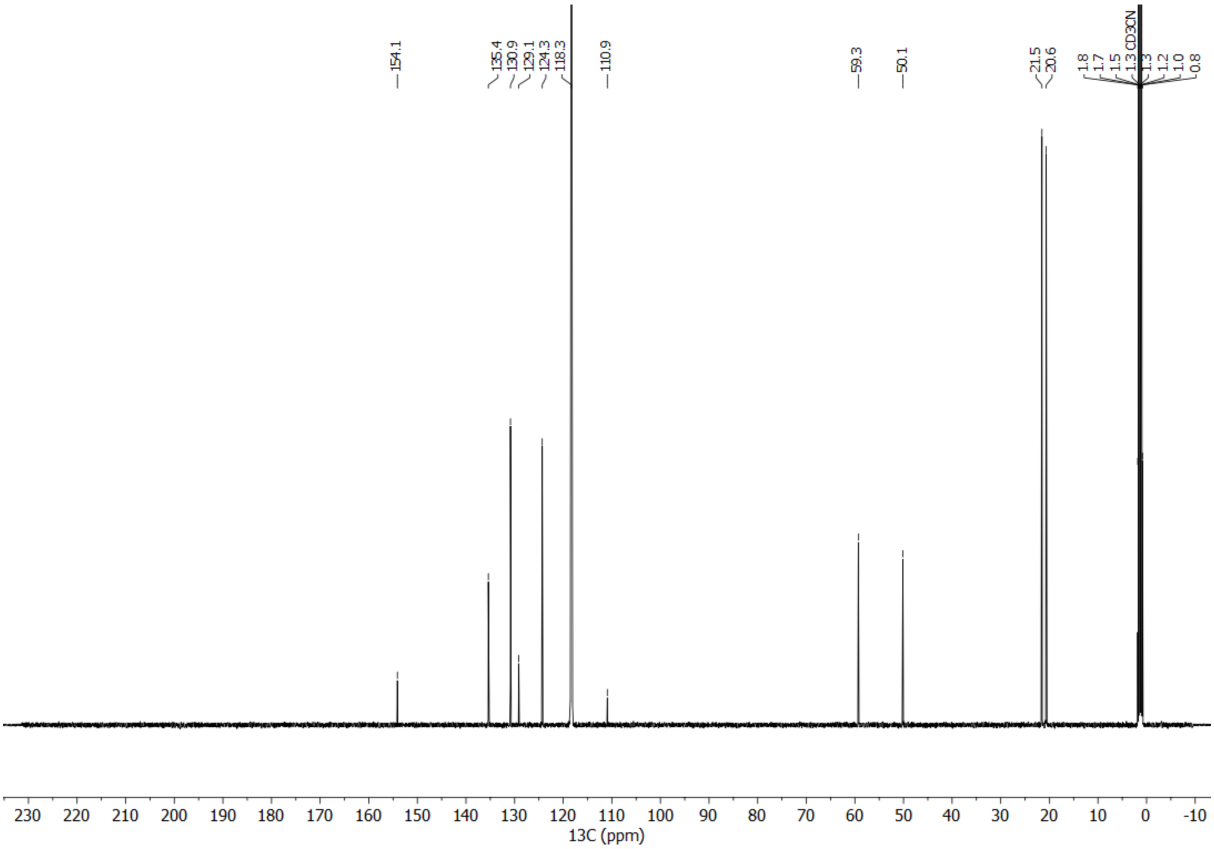

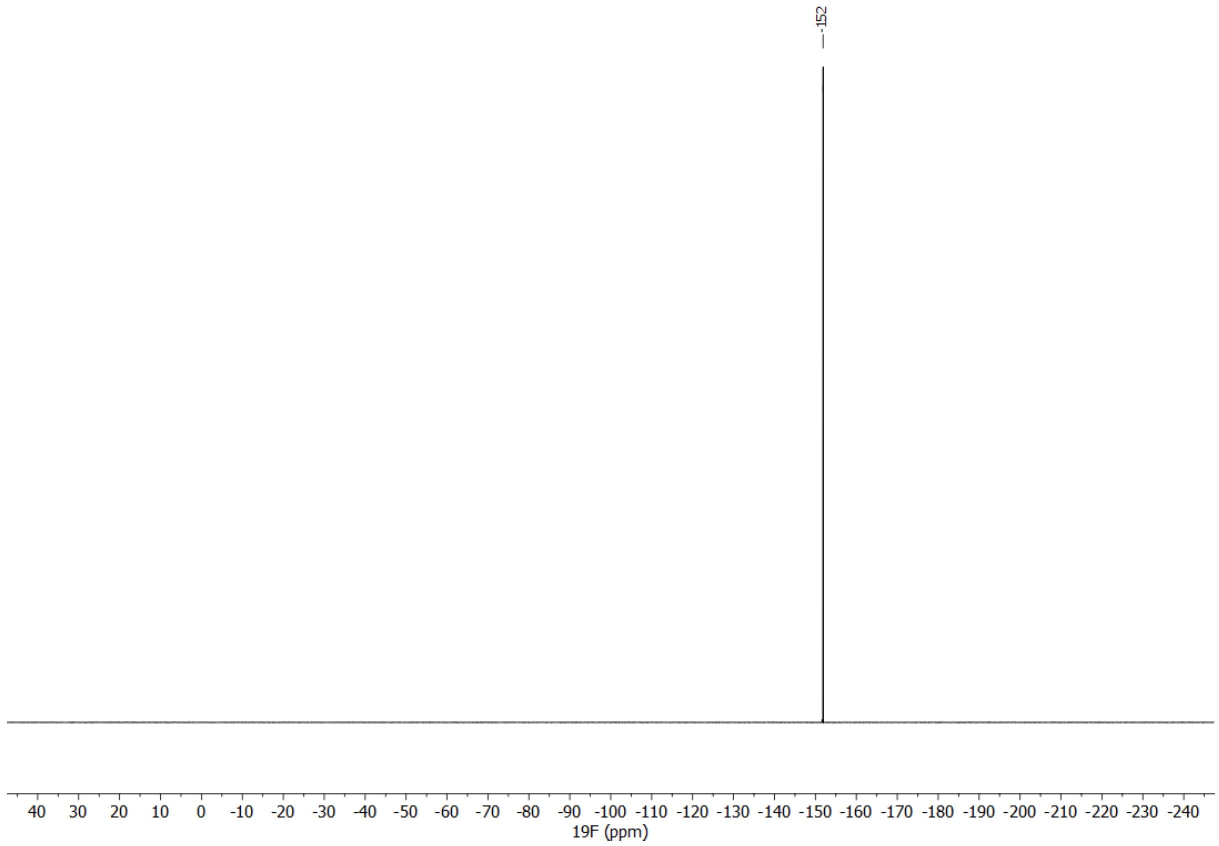

-152
40 30 20 10 0 -10 -20 -30 -40 -50 -60 -70 -80 -90 -100 -110 -120 -130 -140 -150 -160 -170 -180 -190 -200 -210 -220 -230 -240
19F (ppm)

^{1}H NMR, ^{13}C NMR, and ^{19}F NMR spectrum of compound **3b**

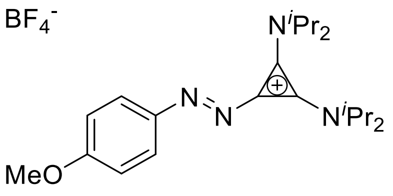

3b

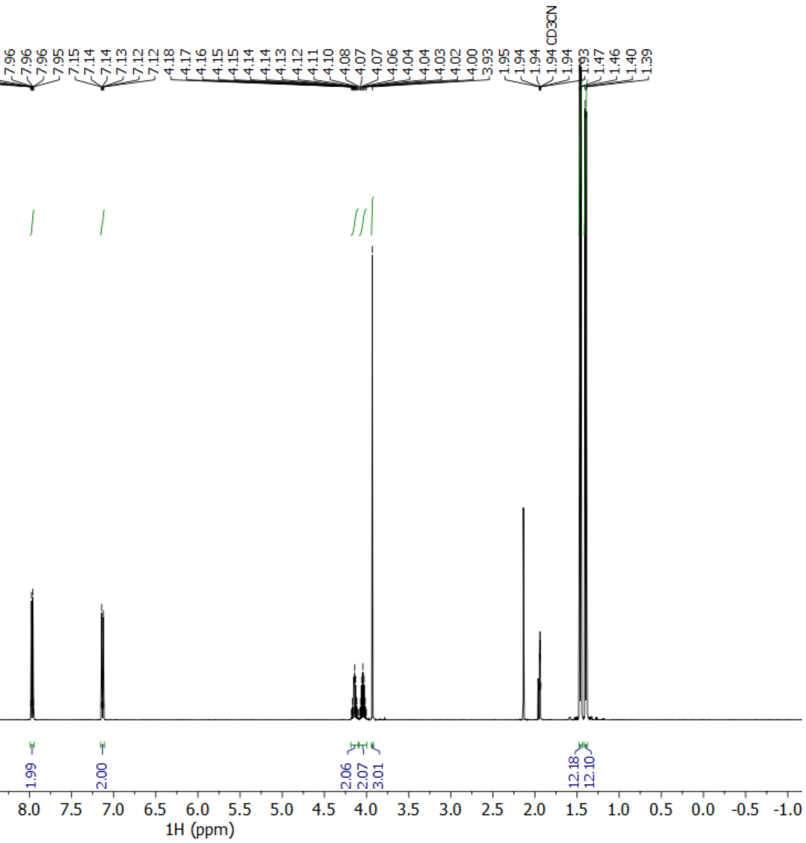

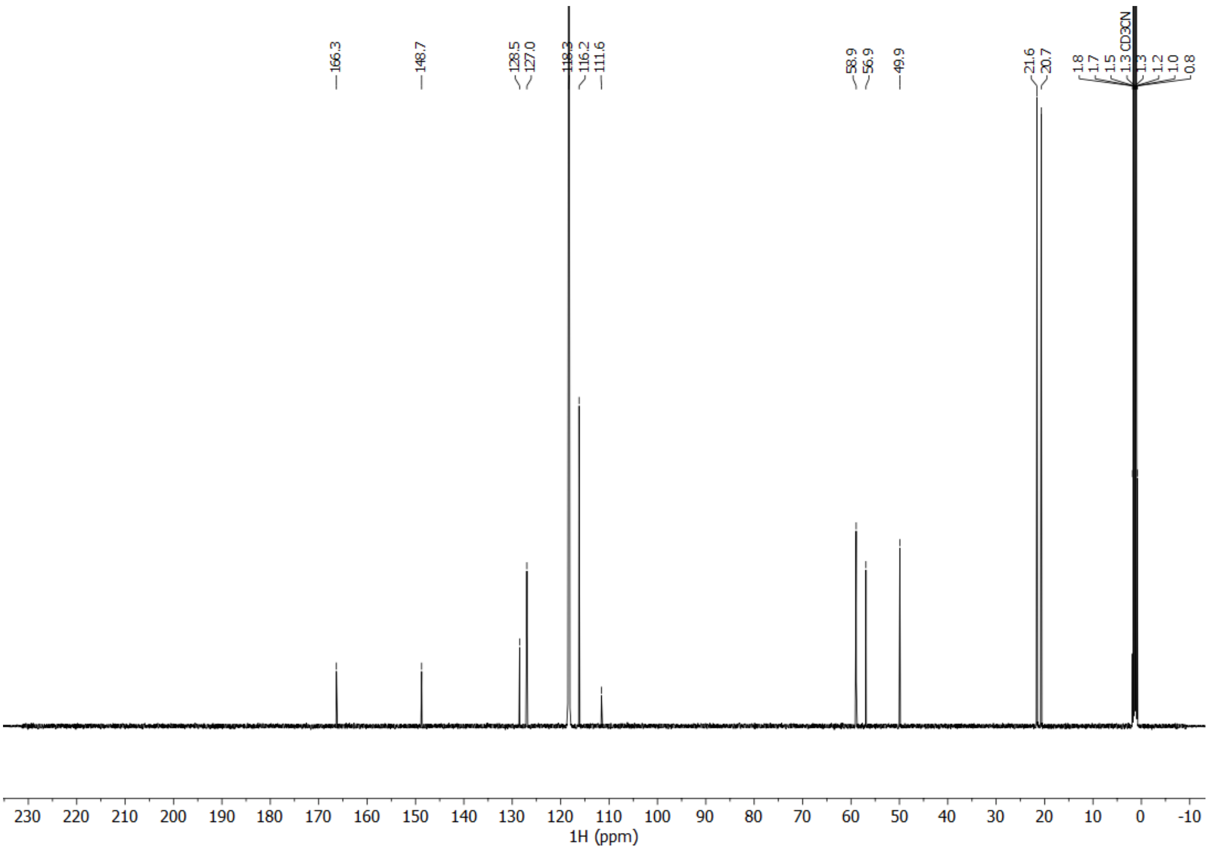

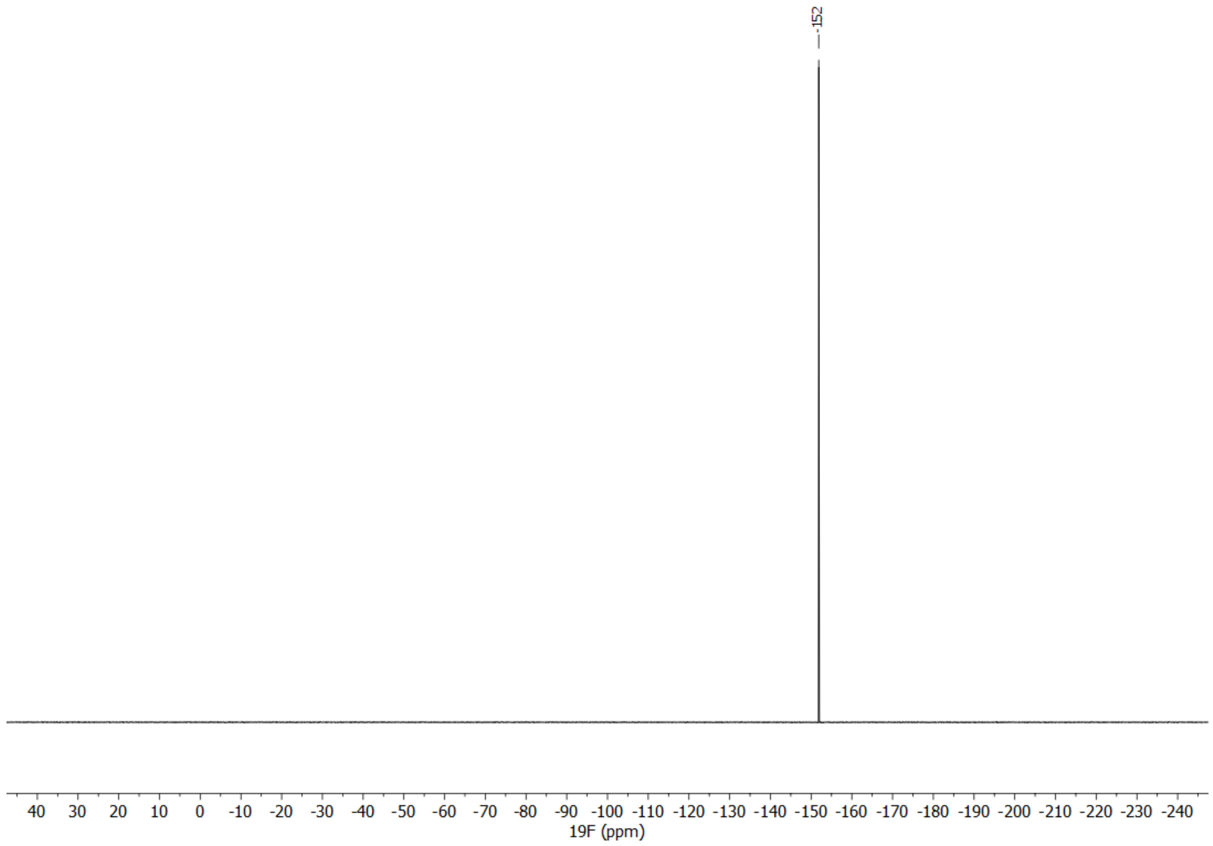
-152
40 30 20 10 0 -10 -20 -30 -40 -50 -60 -70 -80 -90 -100 -110 -120 -130 -140 -150 -160 -170 -180 -190 -200 -210 -220 -230 -240
19F (ppm)

^{1}H NMR, ^{13}C NMR, and ^{19}F NMR spectrum of compound **3c**

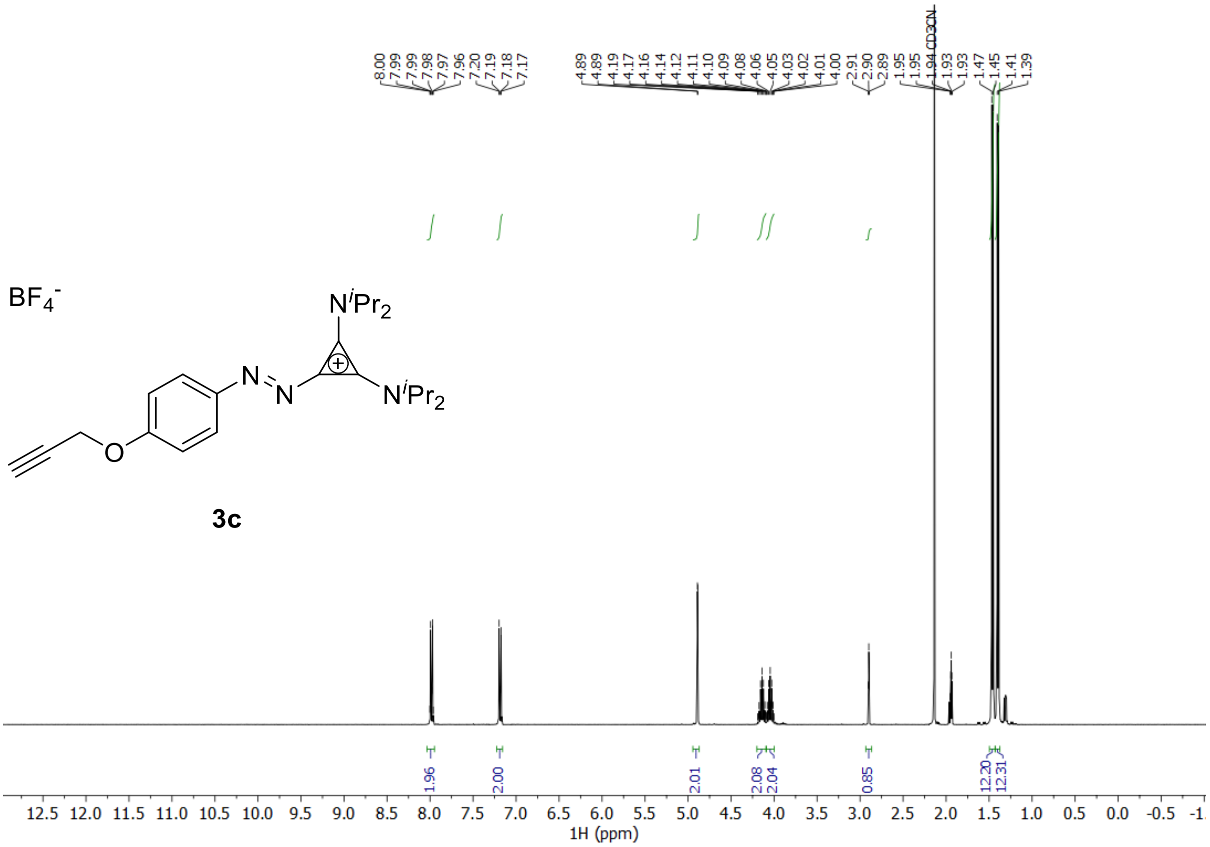

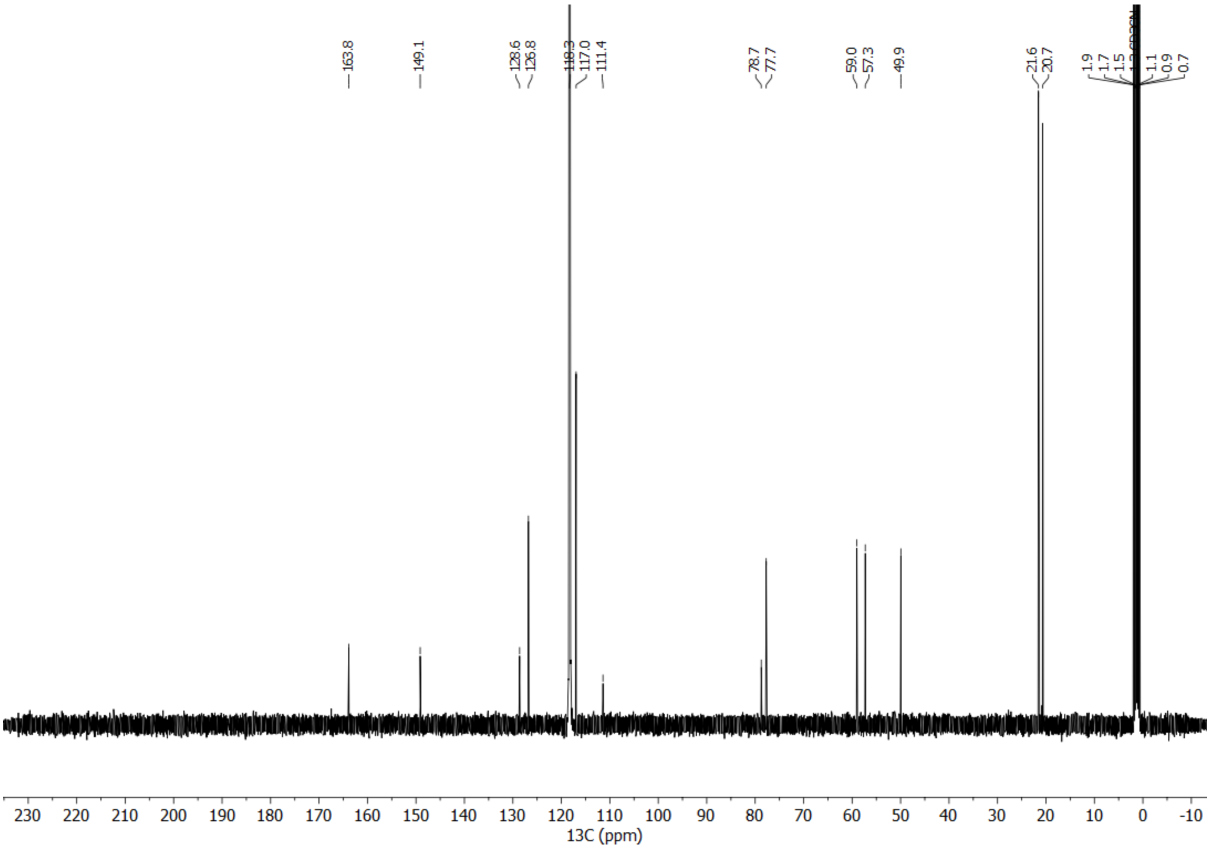

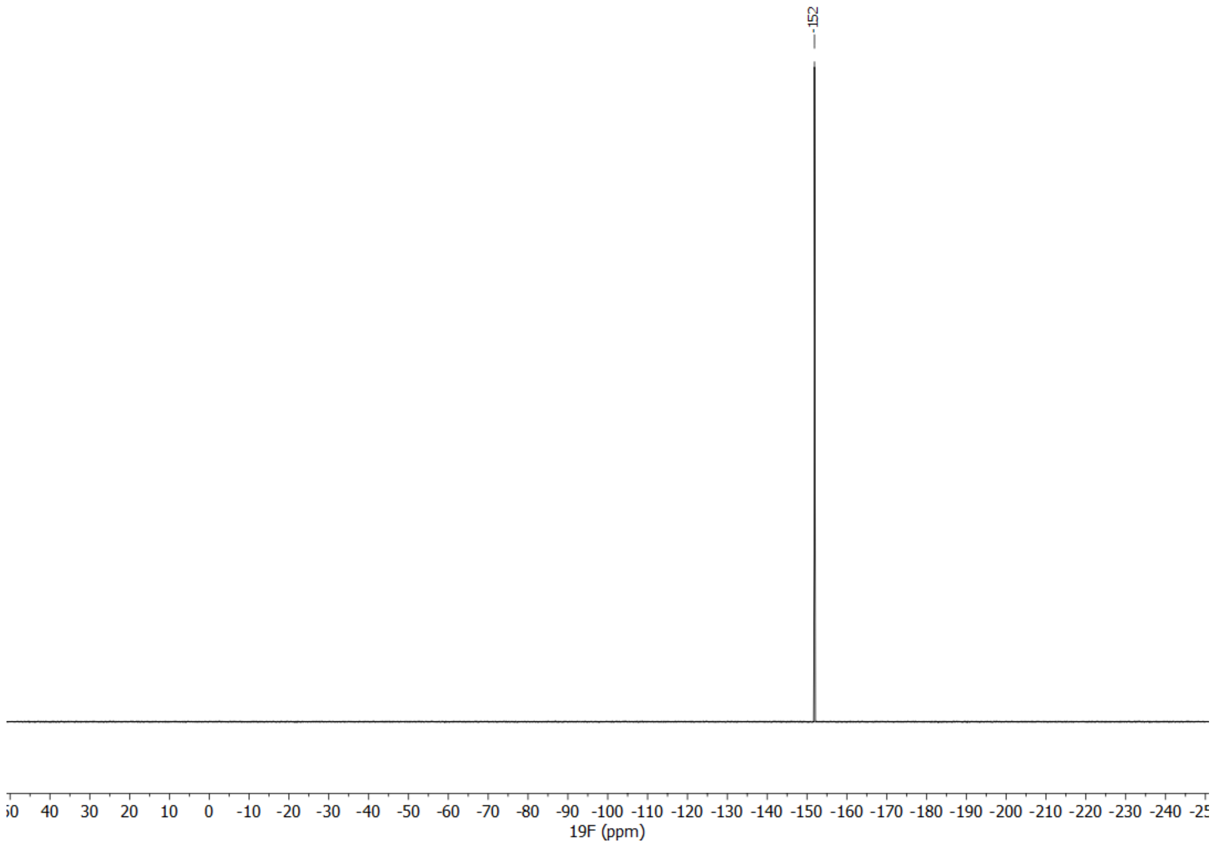
-152
50 40 30 20 10 0 -10 -20 -30 -40 -50 -60 -70 -80 -90 -100 -110 -120 -130 -140 -150 -160 -170 -180 -190 -200 -210 -220 -230 -240 -250
19F (ppm)

^{1}H NMR and ^{13}C NMR spectrum of compound **3d**

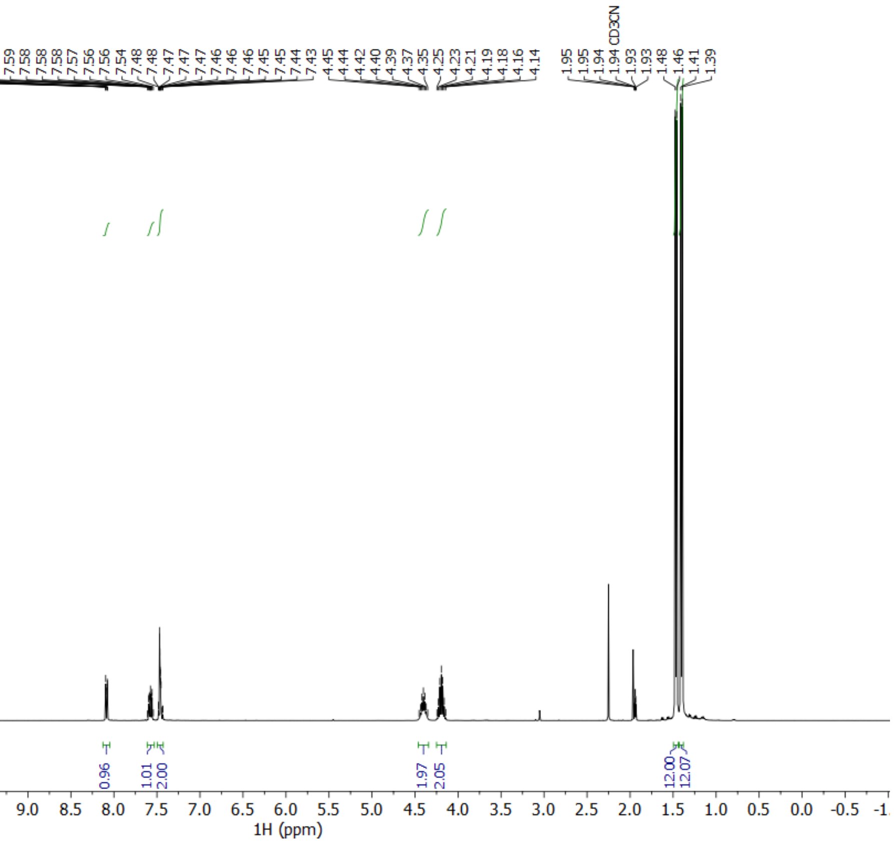

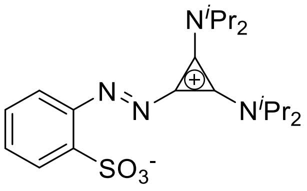

3d

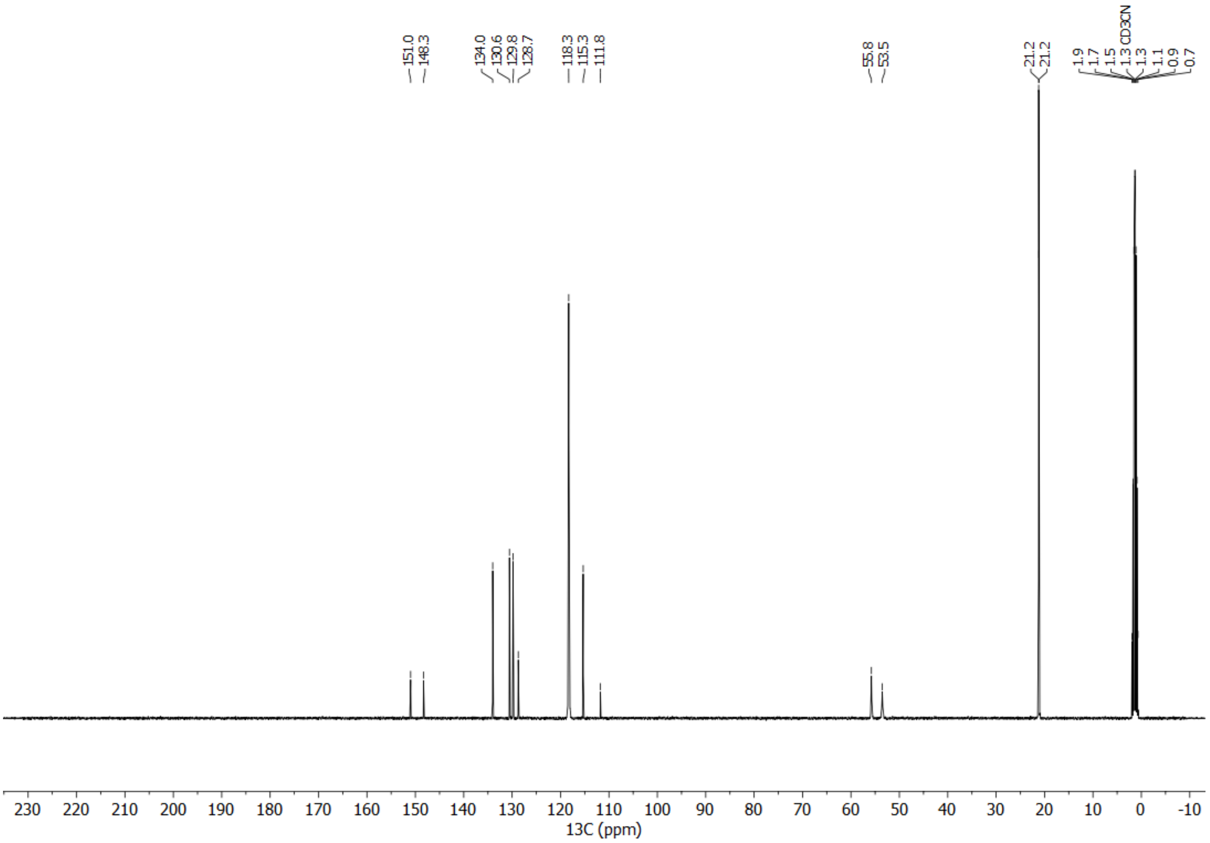

^{1}H NMR and ^{13}C NMR spectrum of compound **SI-4b**

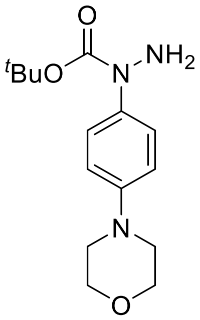

SI-4b

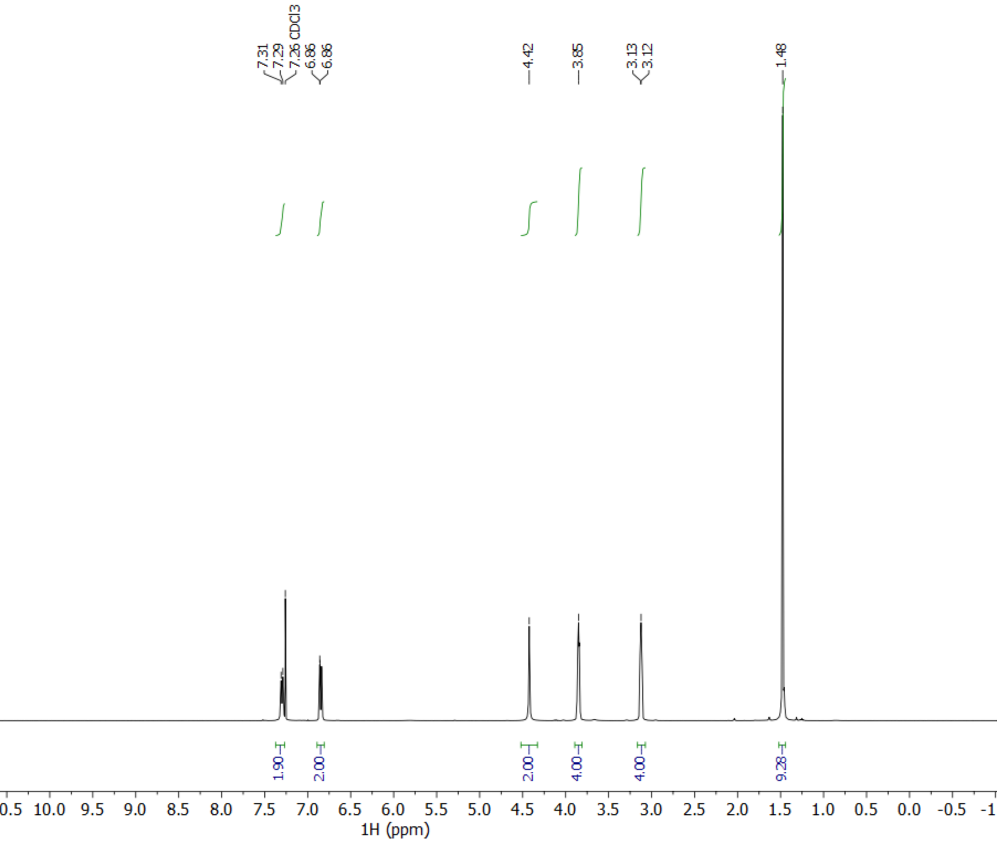

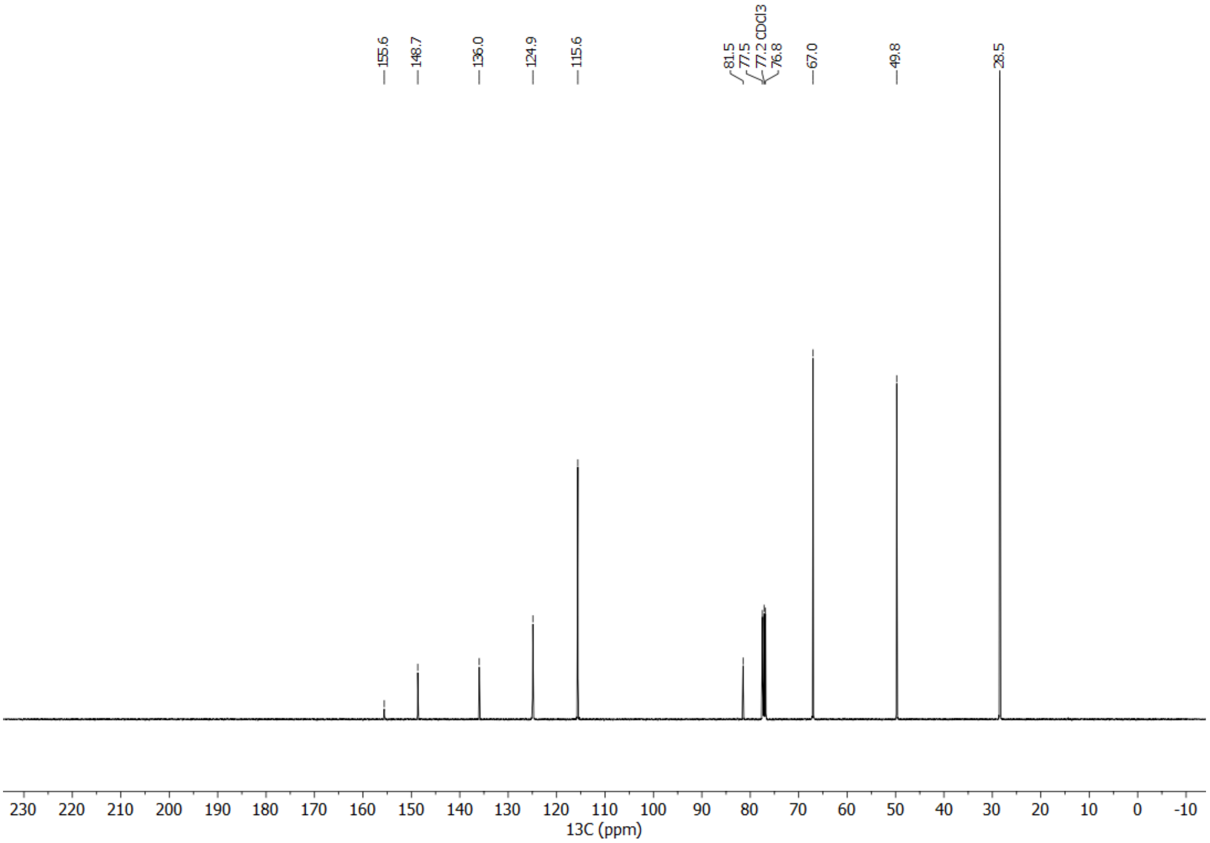

^{1}H NMR and ^{13}C NMR spectrum of compound **SI-4e**

^{1}H NMR and ^{13}C NMR spectrum of compound **SI-4e**

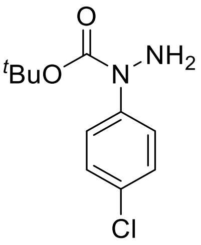

SI-4e

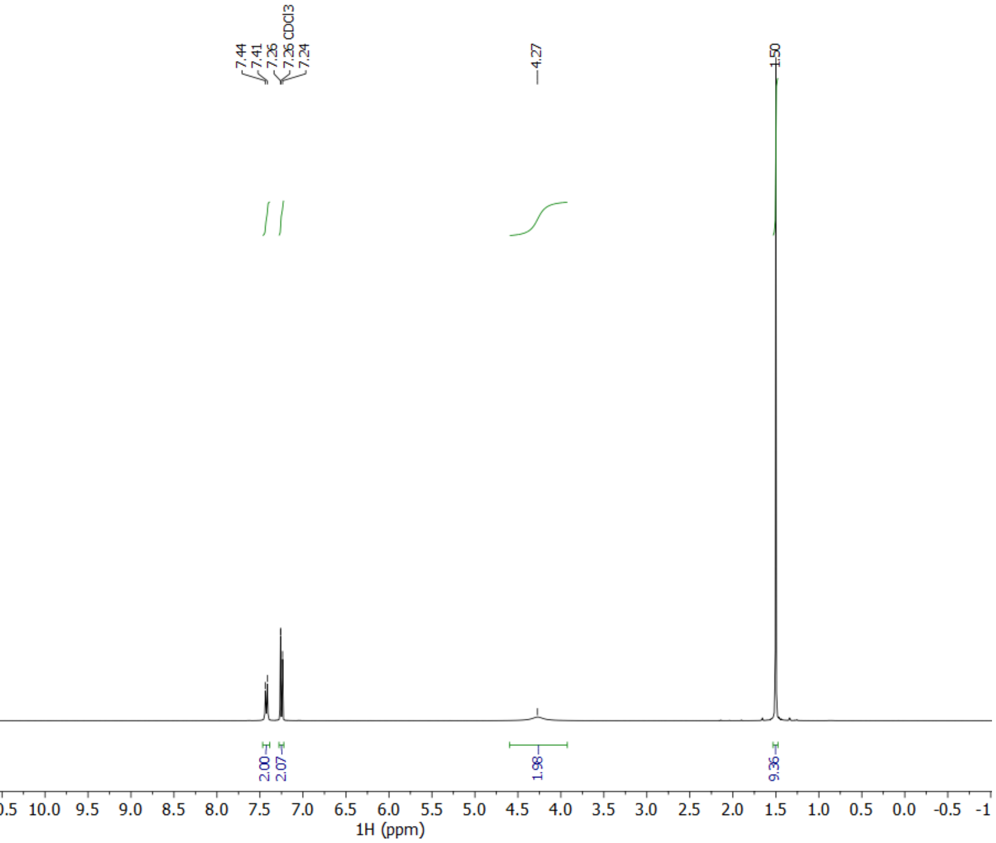

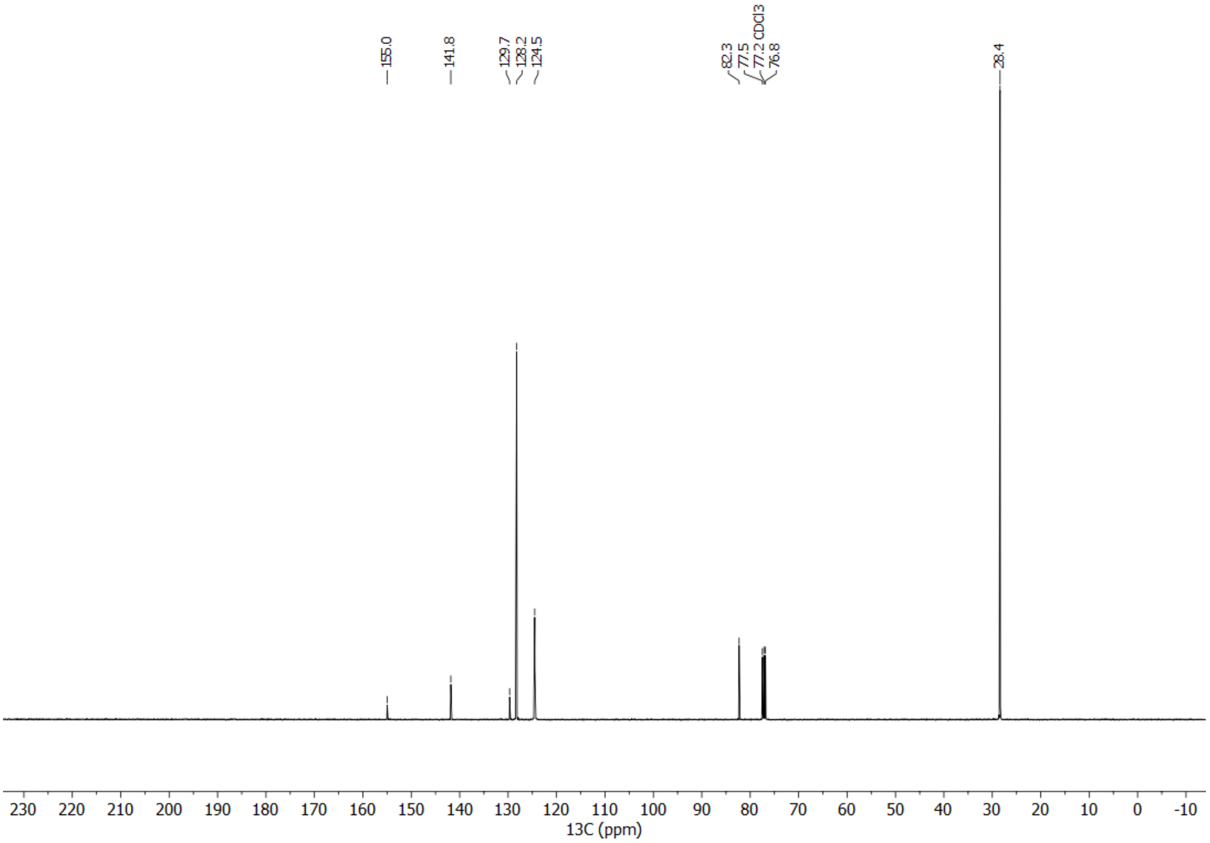

^{1}H NMR, ^{13}C NMR, and ^{19}F NMR spectrum of compound **SI-4g**

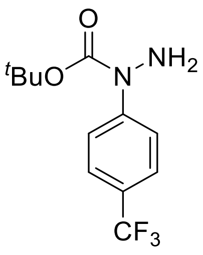

SI-4g

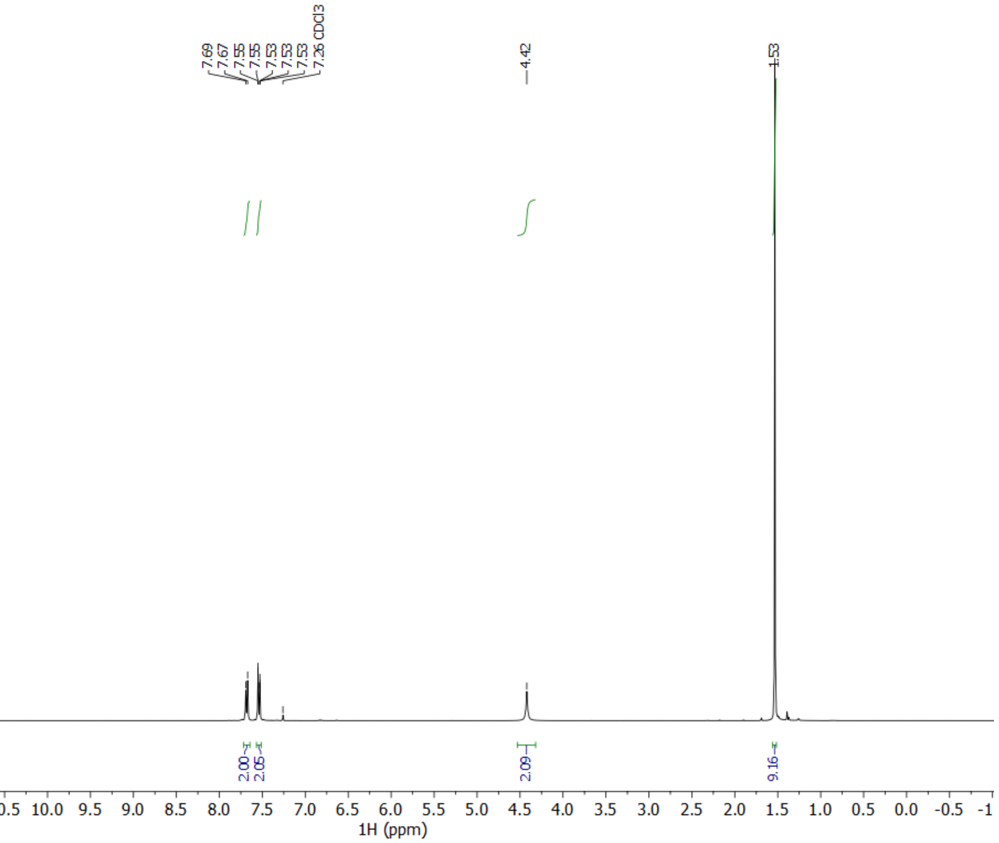

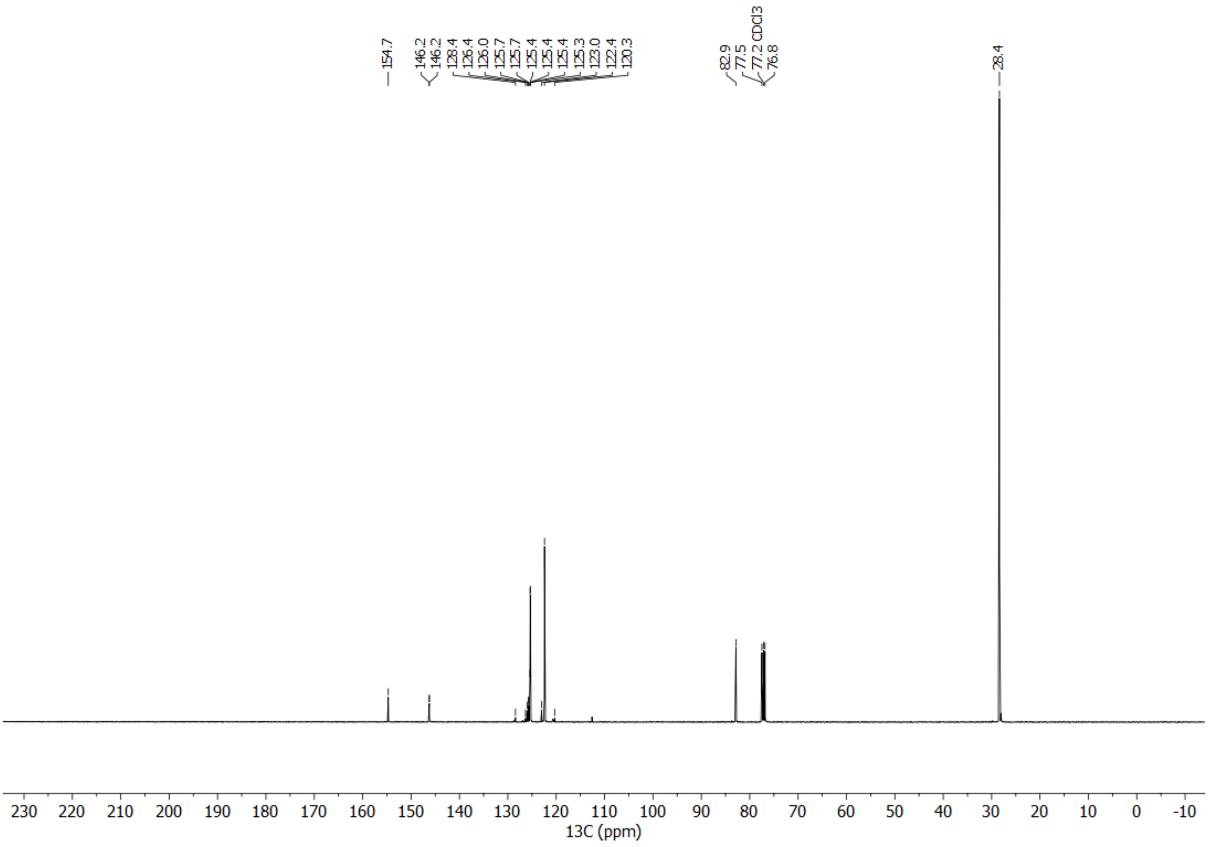

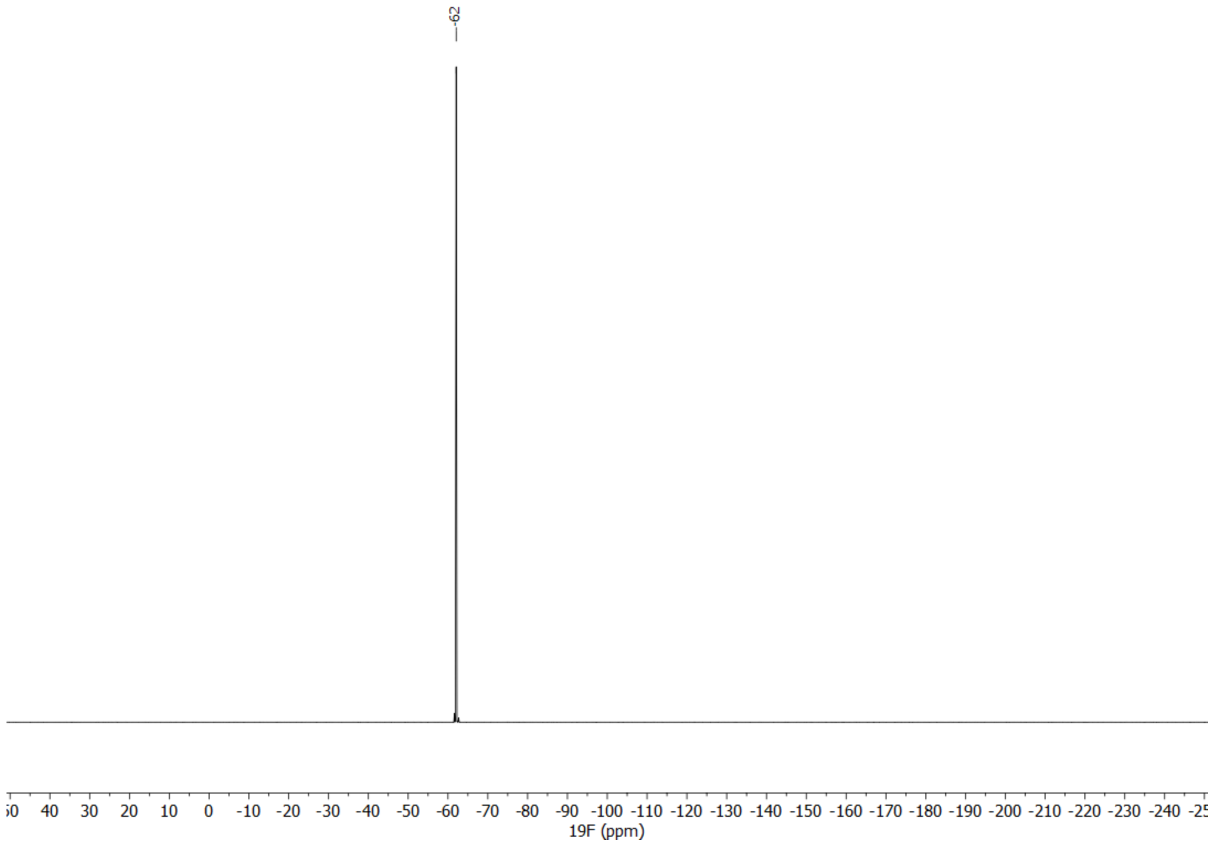
-62
50 40 30 20 10 0 -10 -20 -30 -40 -50 -60 -70 -80 -90 -100 -110 -120 -130 -140 -150 -160 -170 -180 -190 -200 -210 -220 -230 -240 -25
19F (ppm)

^{1}H NMR and ^{13}C NMR spectrum of compound **SI-4h**

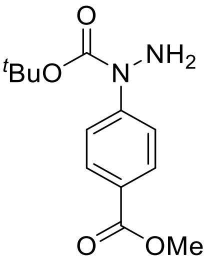

SI-4h

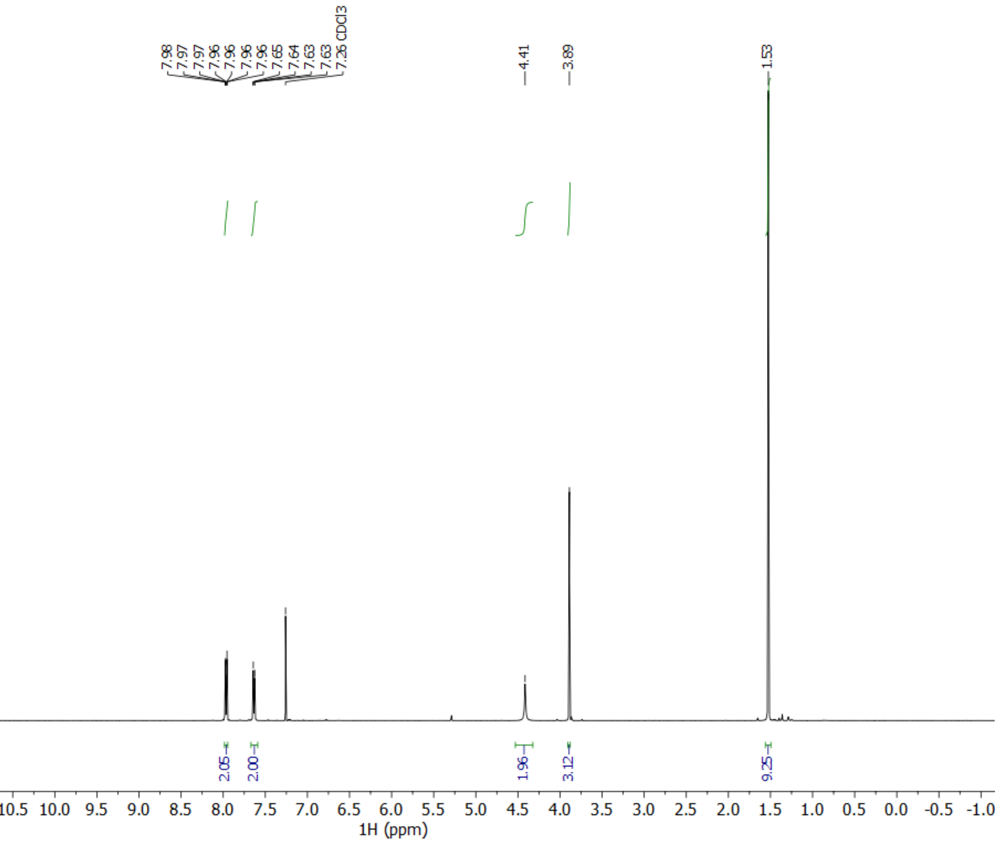

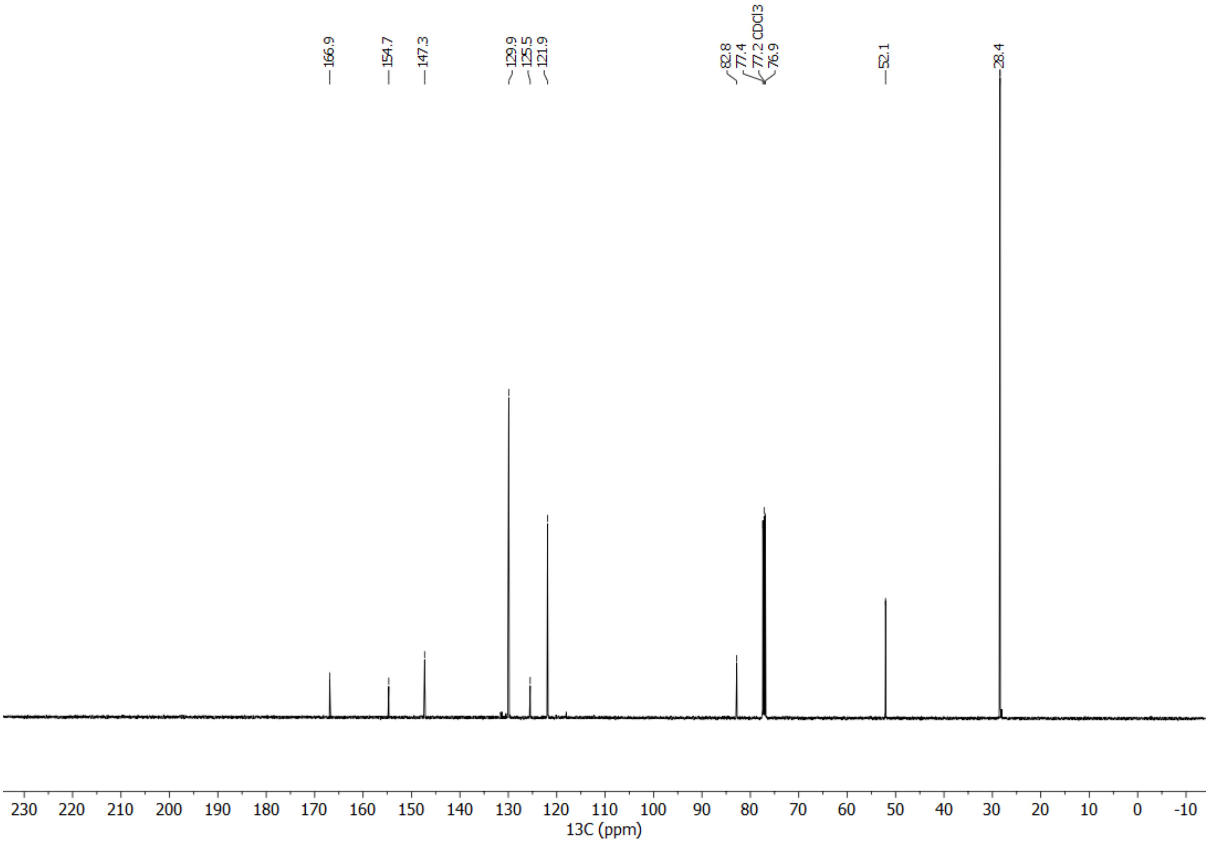

^{1}H NMR and ^{13}C NMR spectrum of compound **SI-4i**

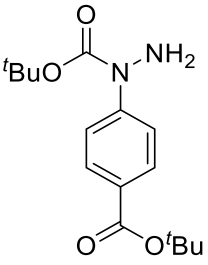

SI-4i

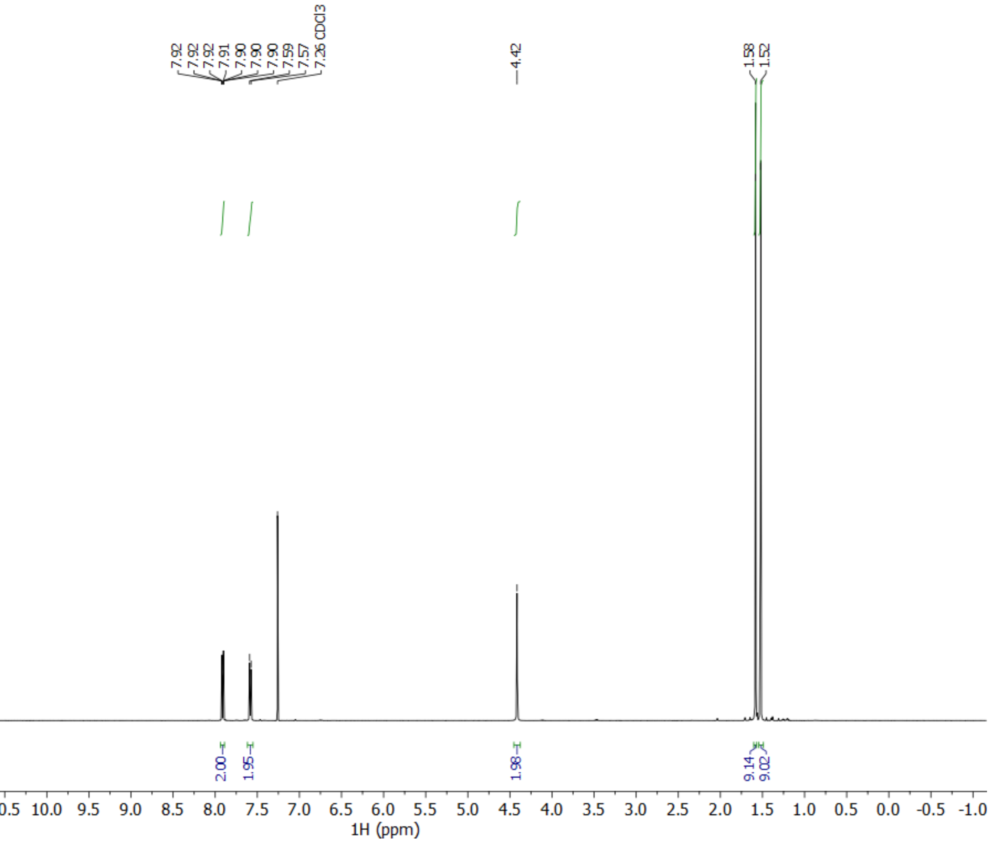

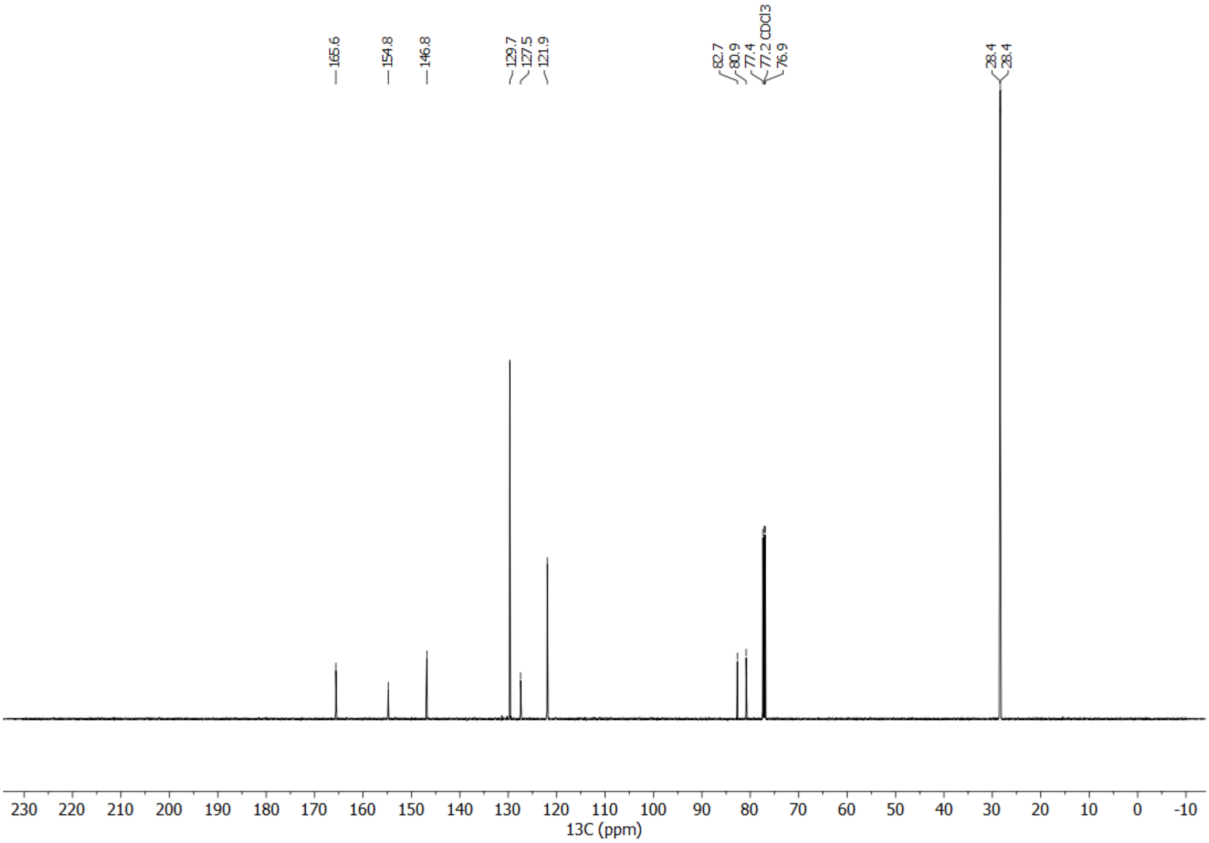

^{1}H NMR and ^{13}C NMR spectrum of compound **SI-4l**

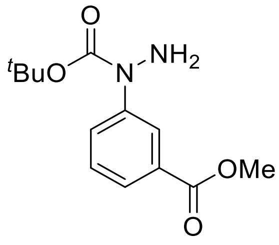

SI-4l

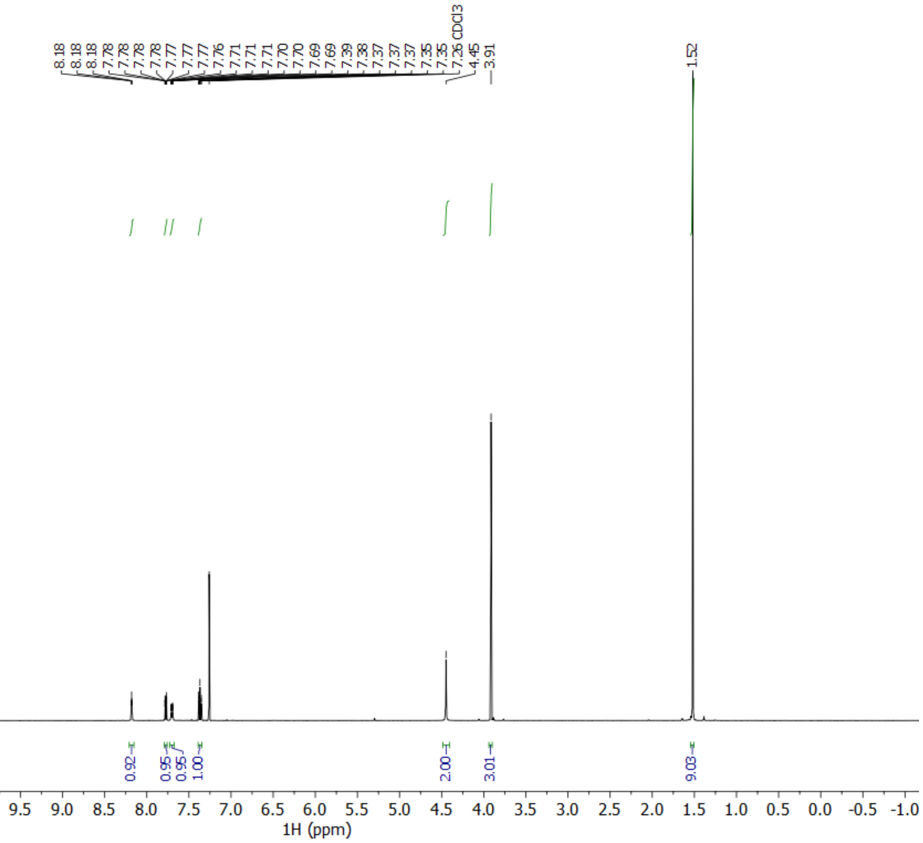

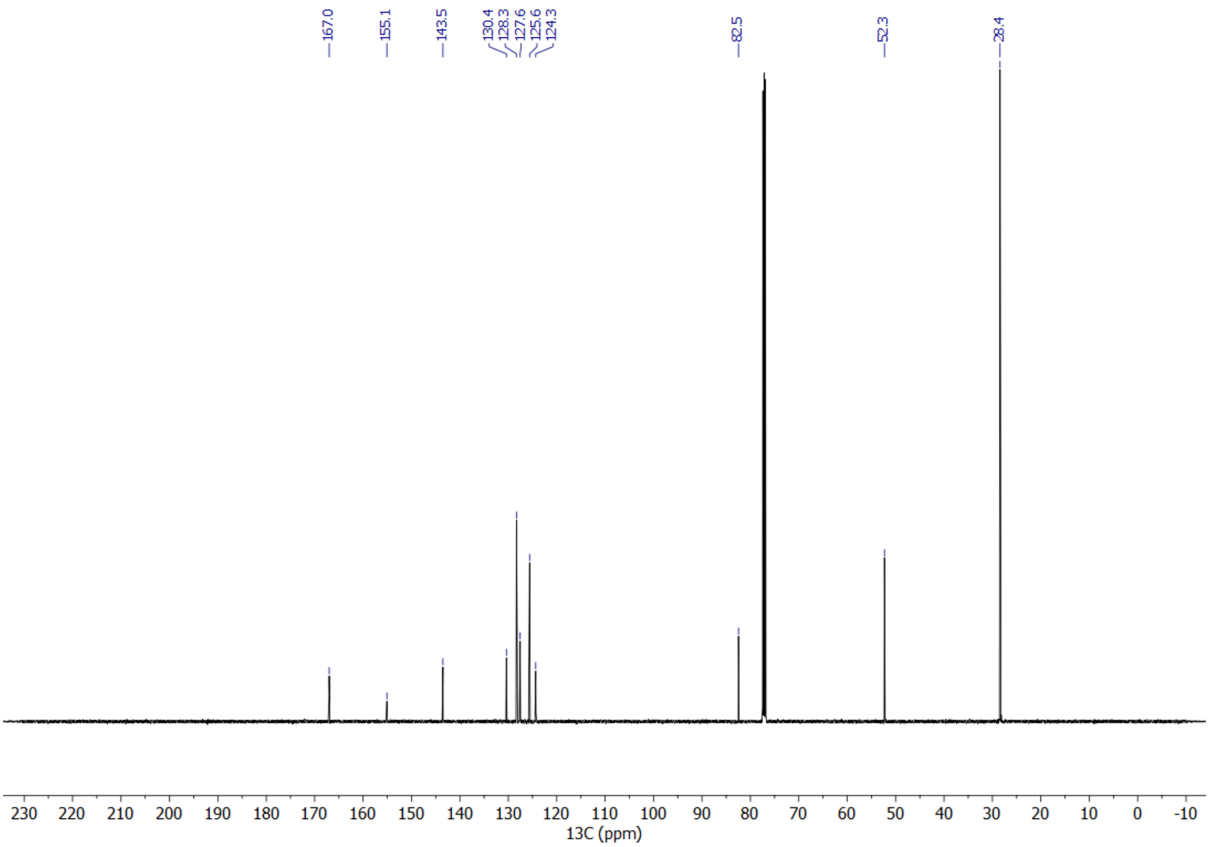

^{1}H NMR and ^{13}C NMR spectrum of compound **SI-4m**

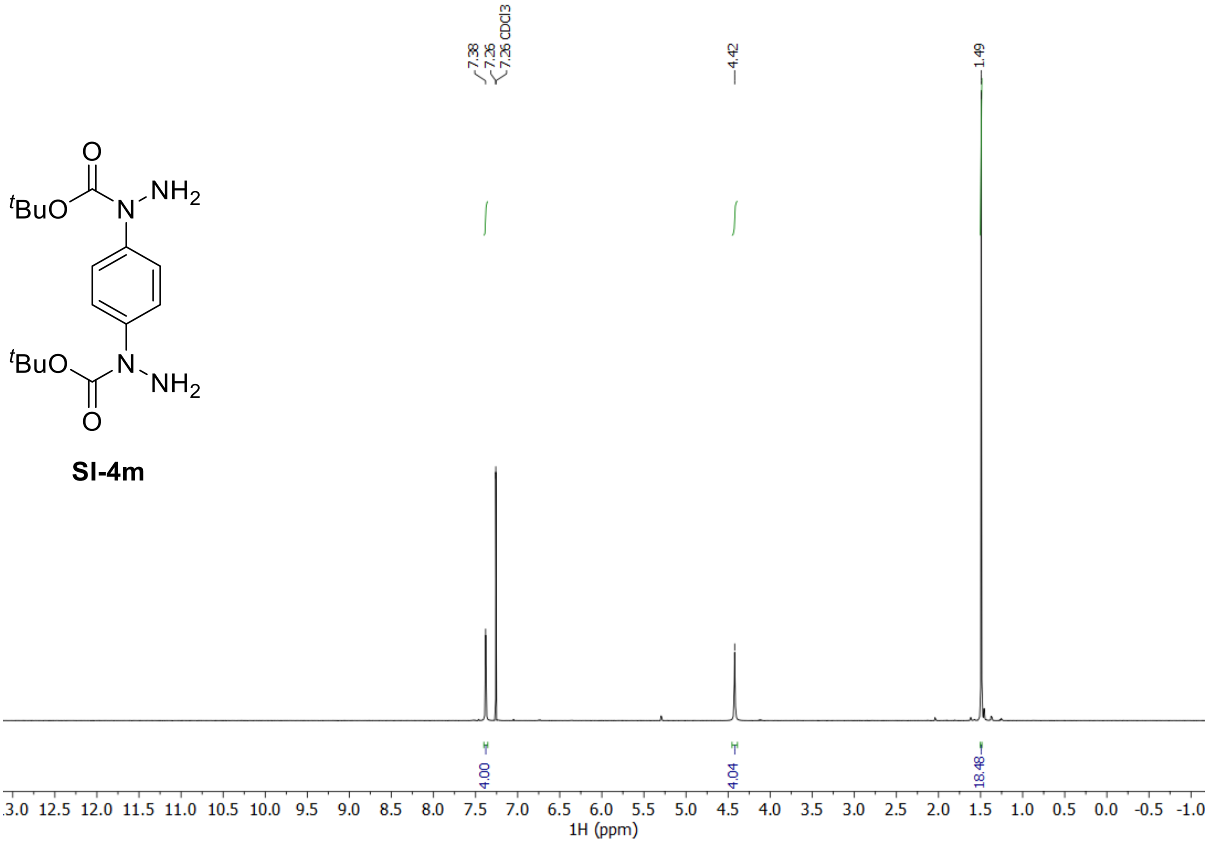

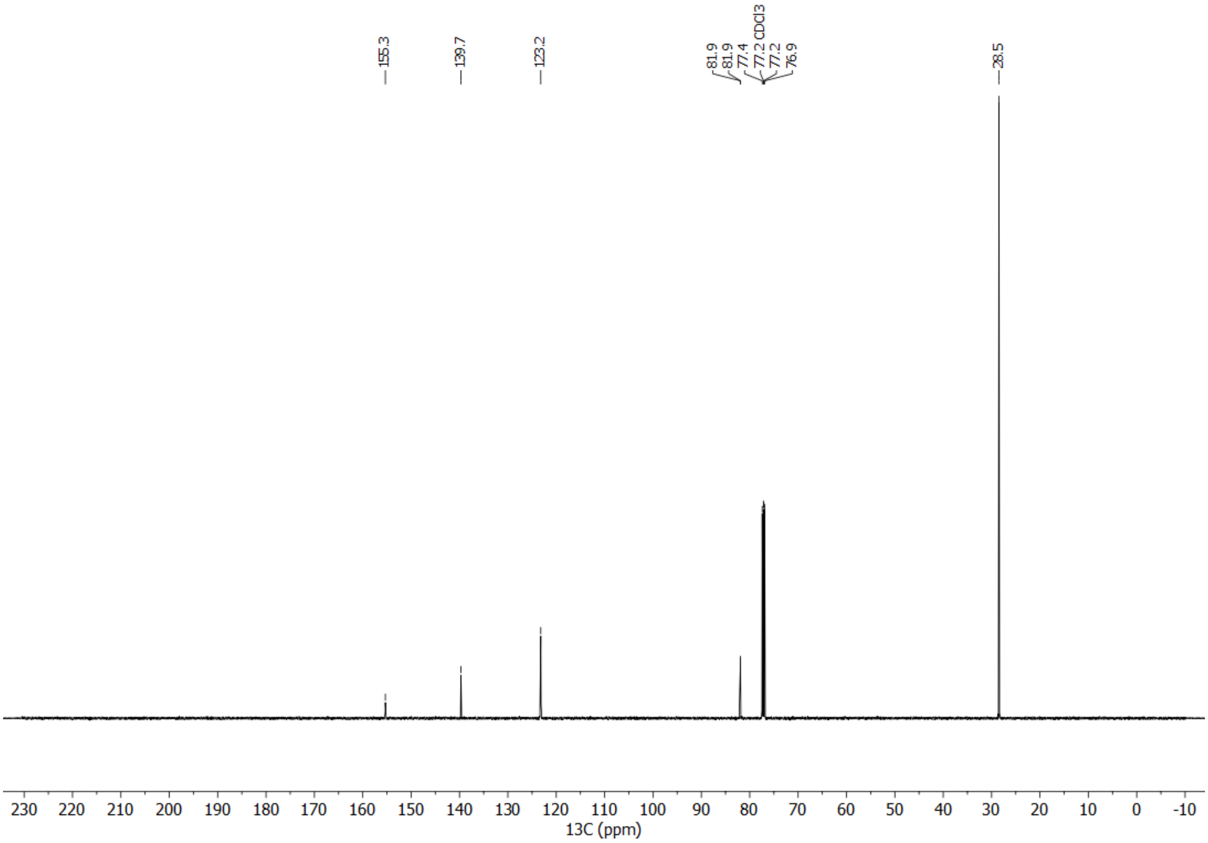

^{1}H NMR and ^{13}C NMR spectrum of compound **SI-4n**

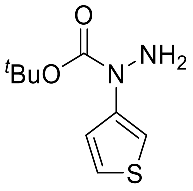

SI-4n

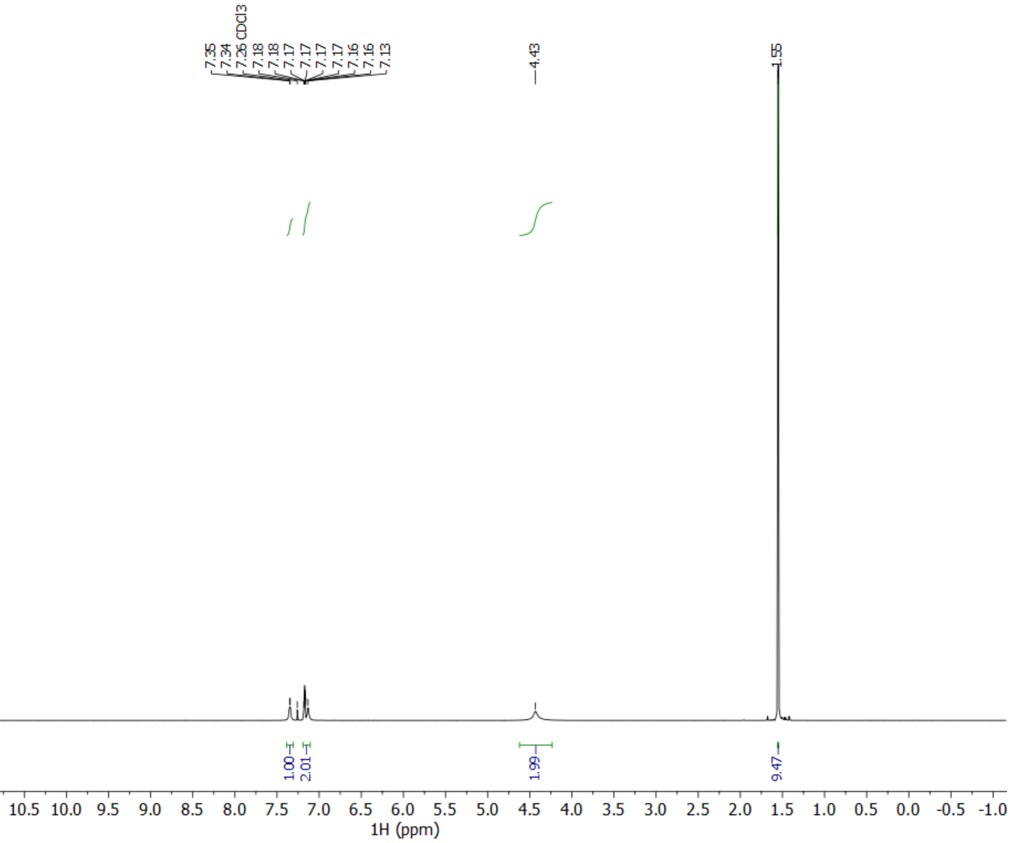

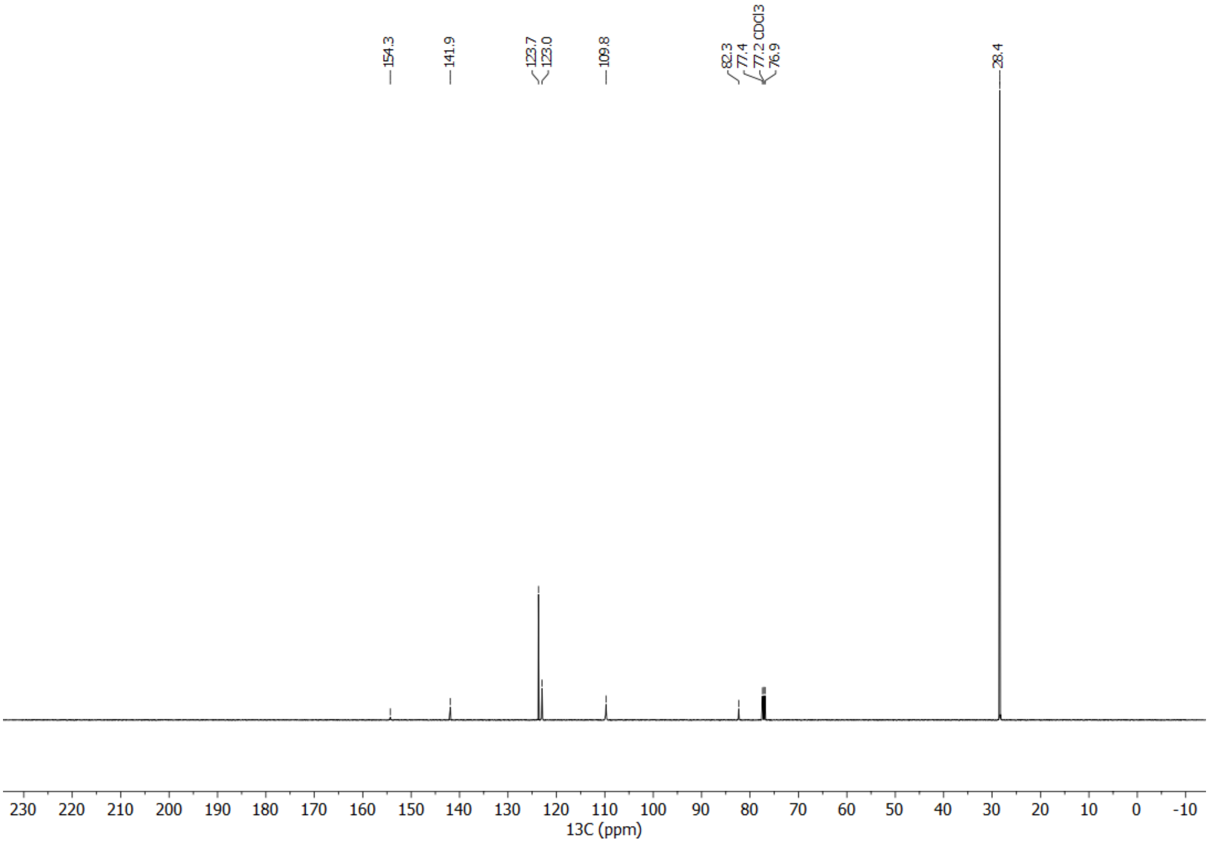

^{1}H NMR and ^{13}C NMR spectrum of compound **SI-4o**

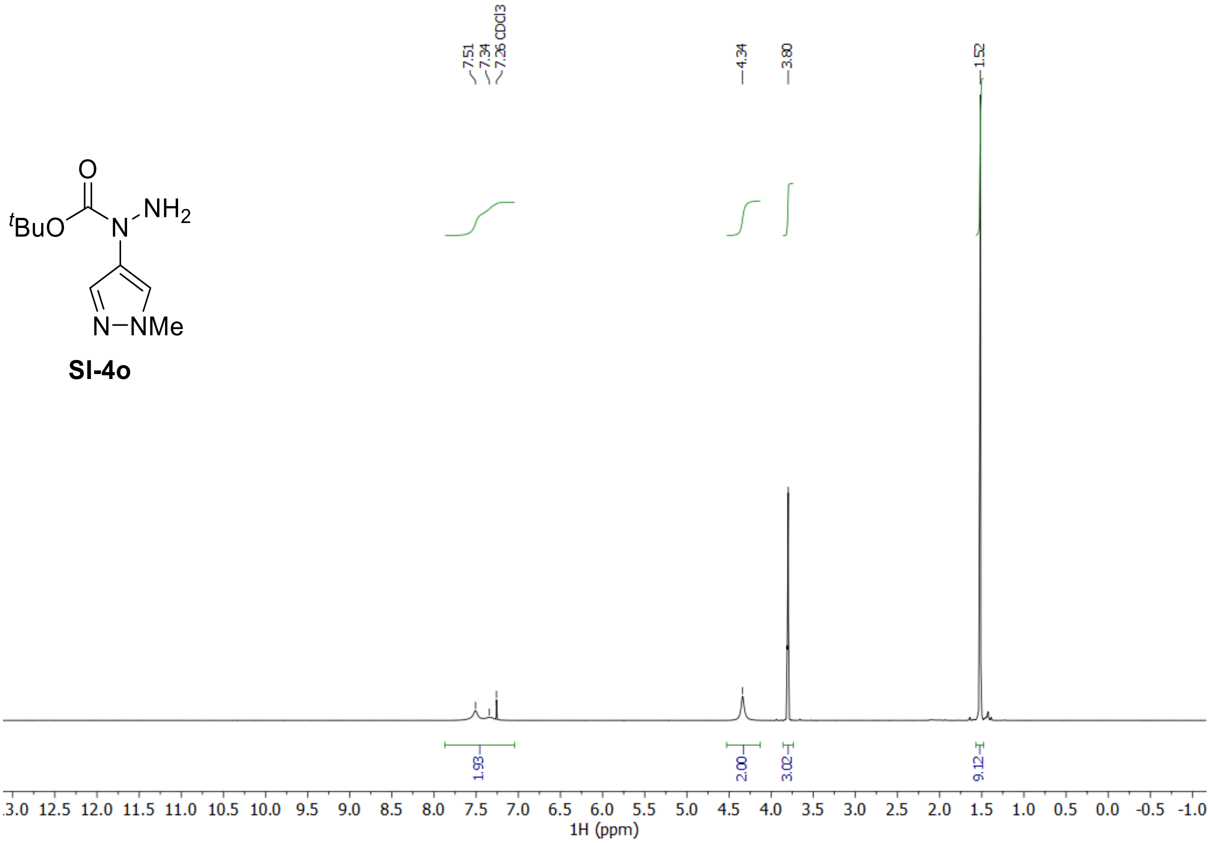

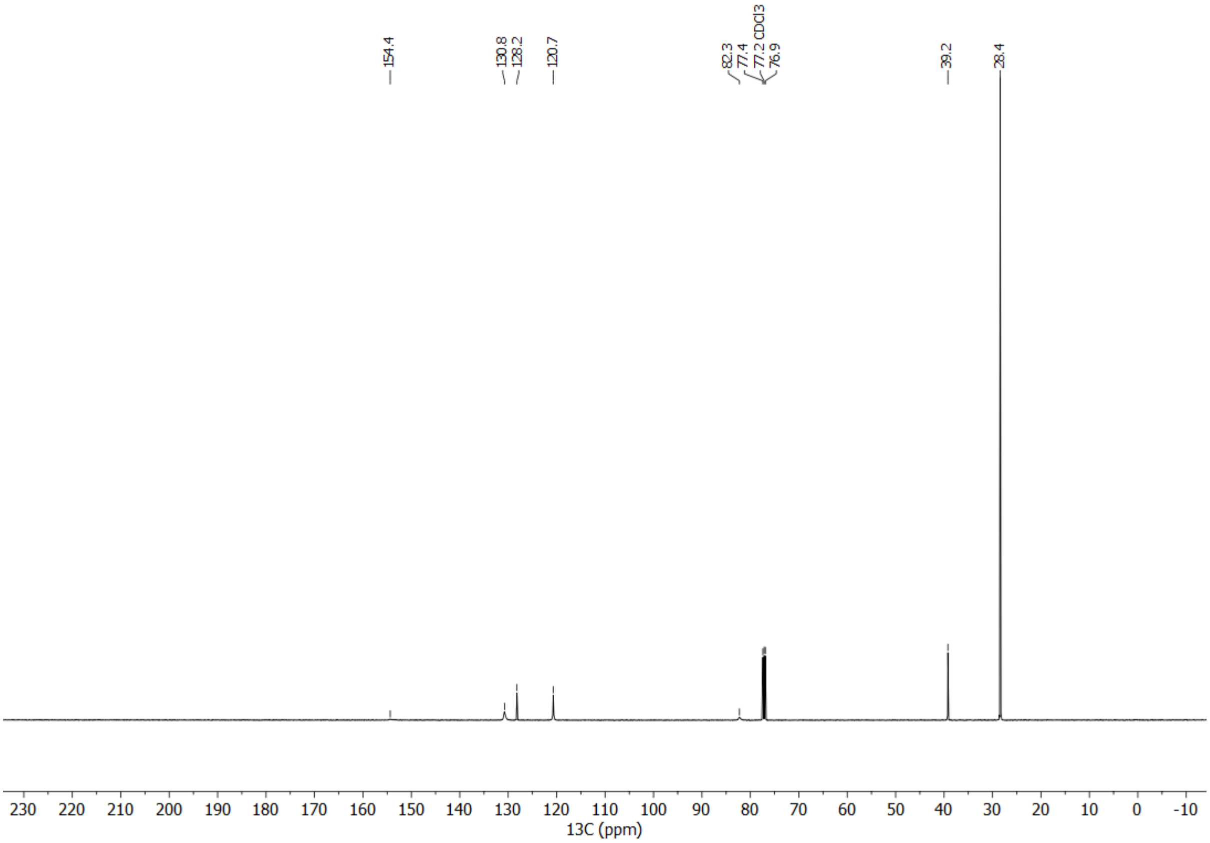

^{1}H NMR and ^{13}C NMR spectrum of compound **SI-4s**

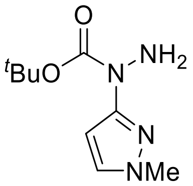

SI-4s

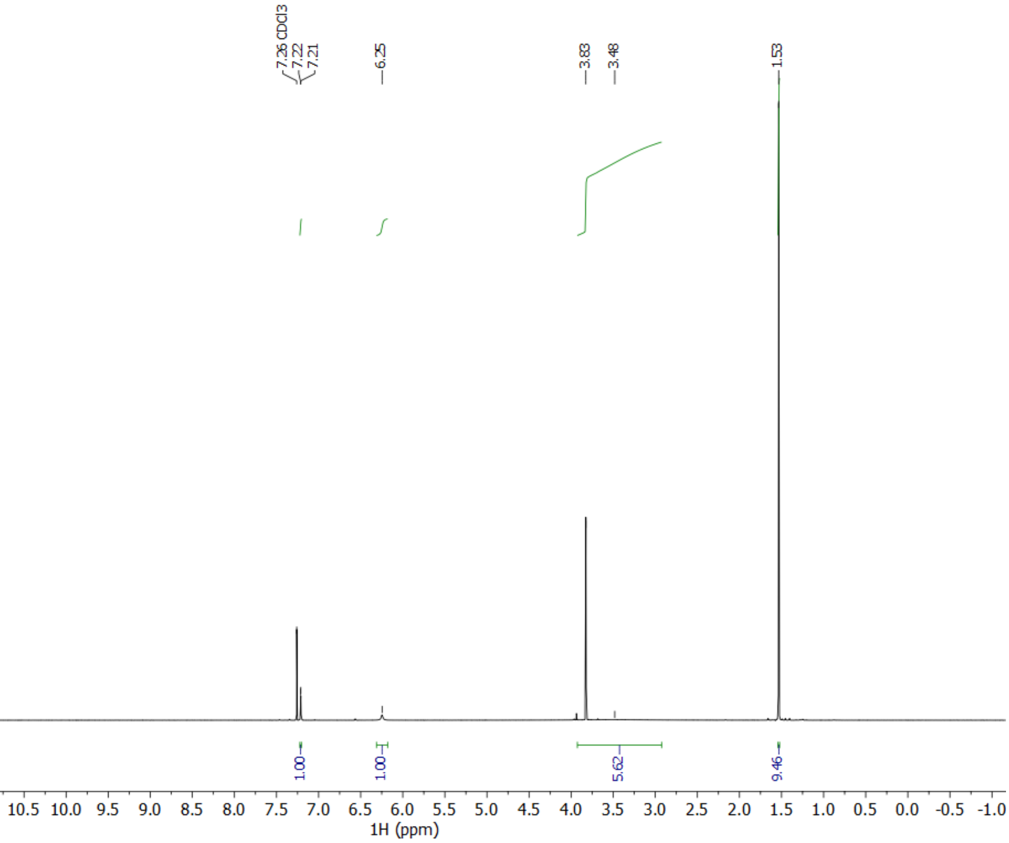

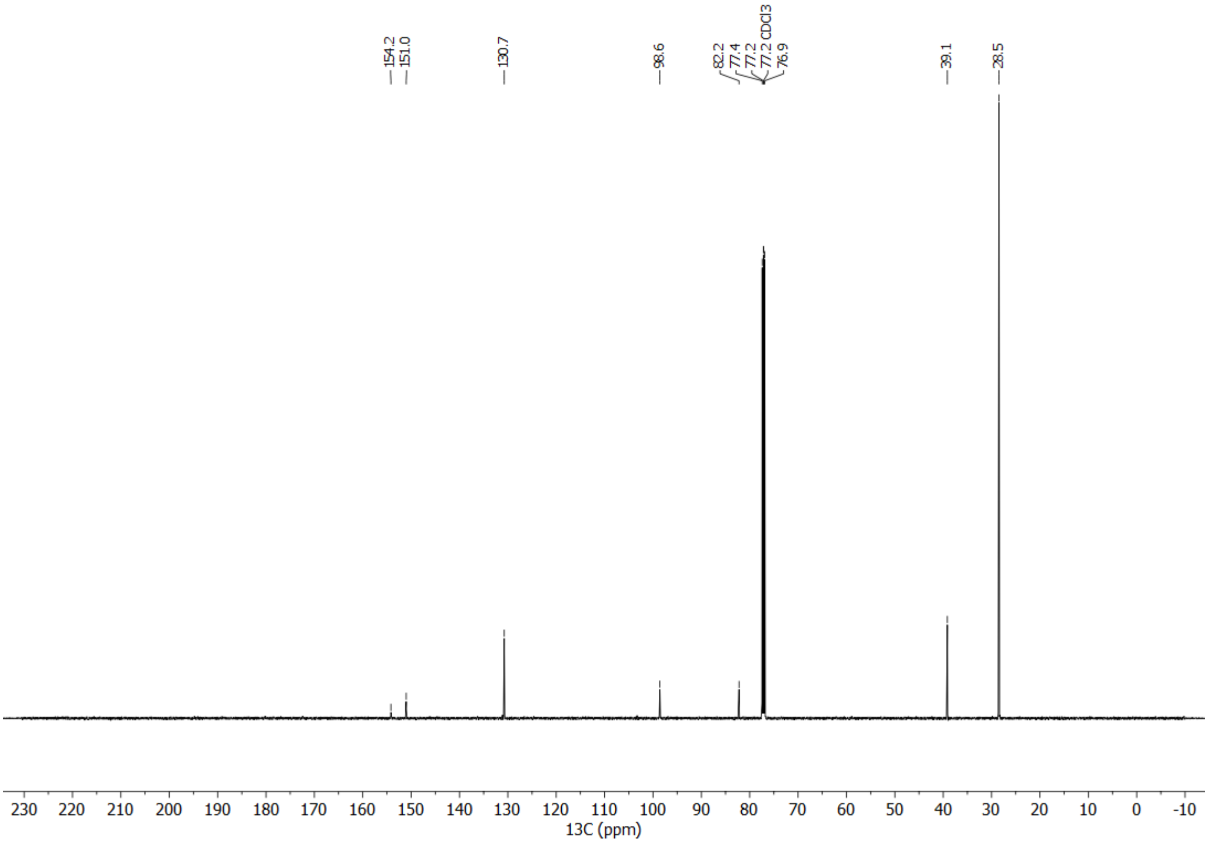

^{1}H NMR and ^{13}C NMR spectrum of compound **SI-4t**

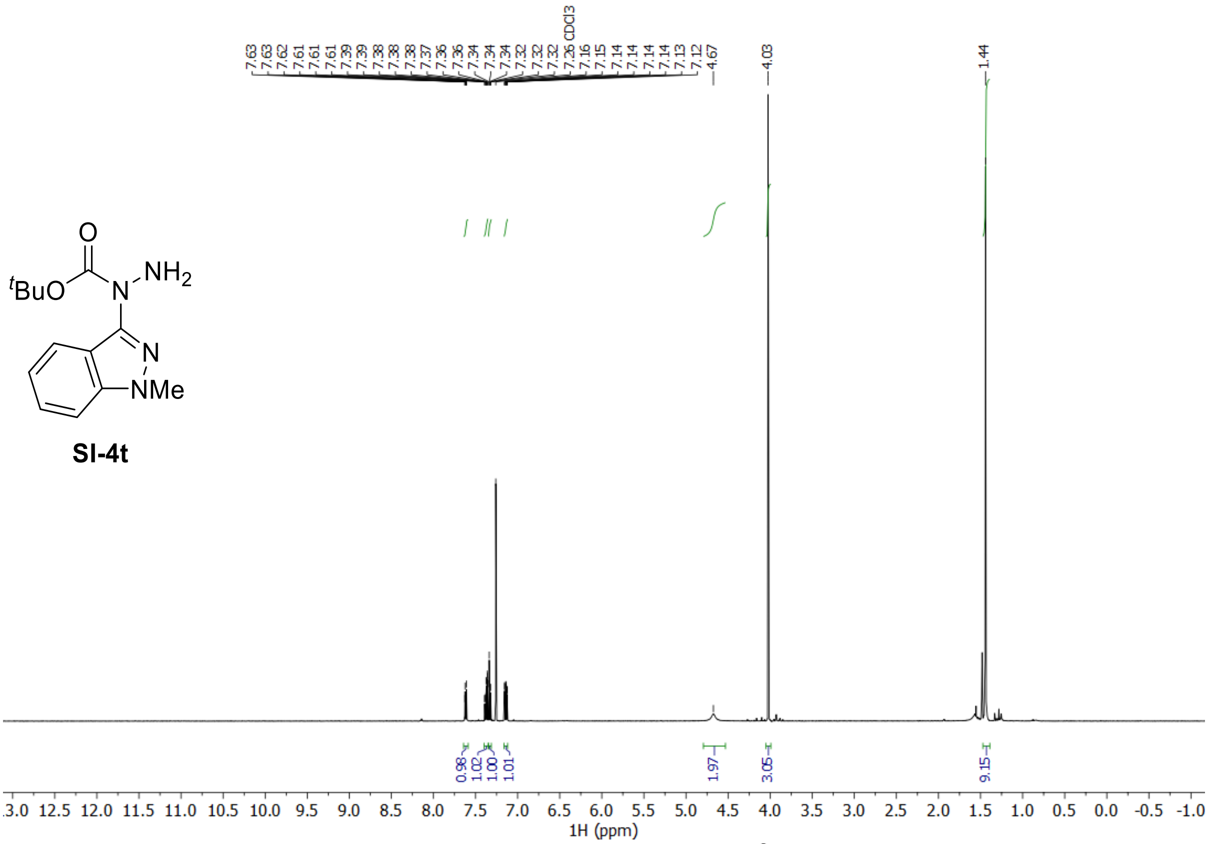

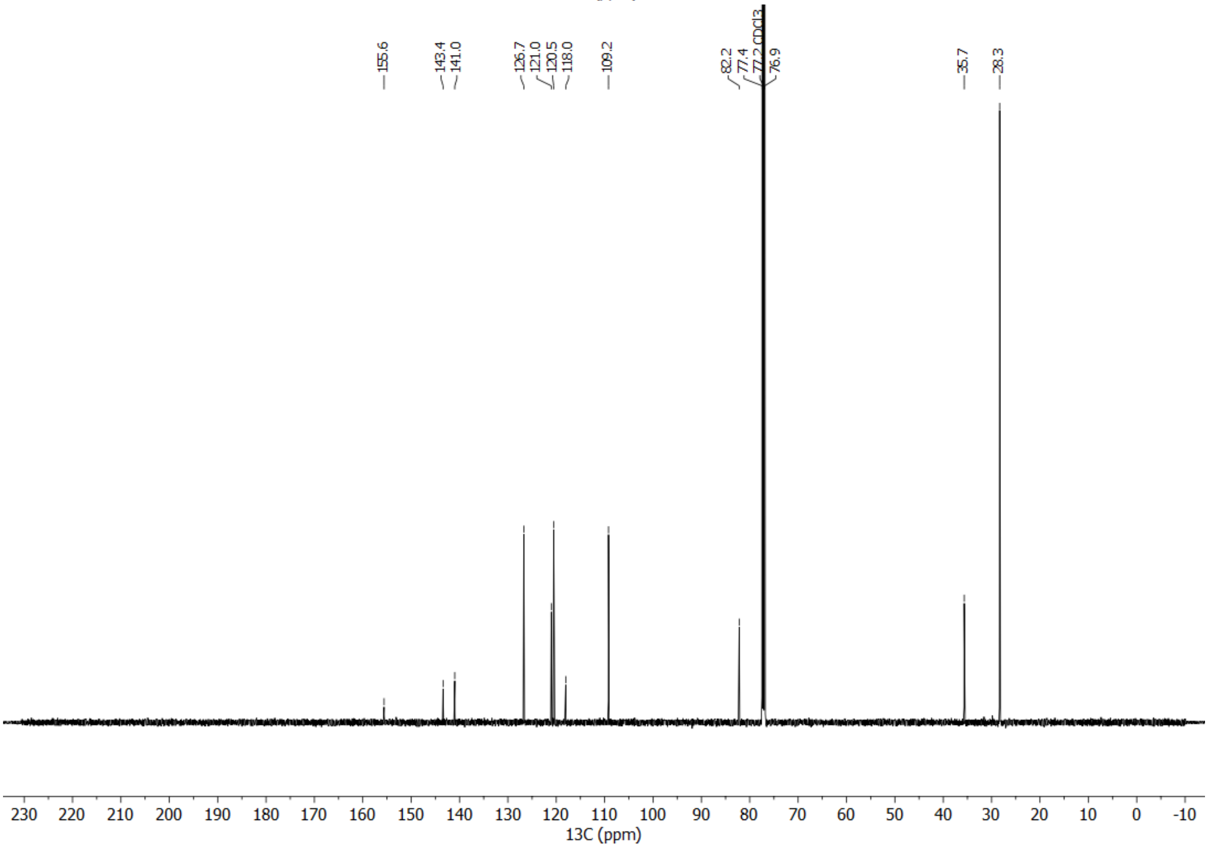

^{1}H NMR and ^{13}C NMR spectrum of compound **SI-4u-2**

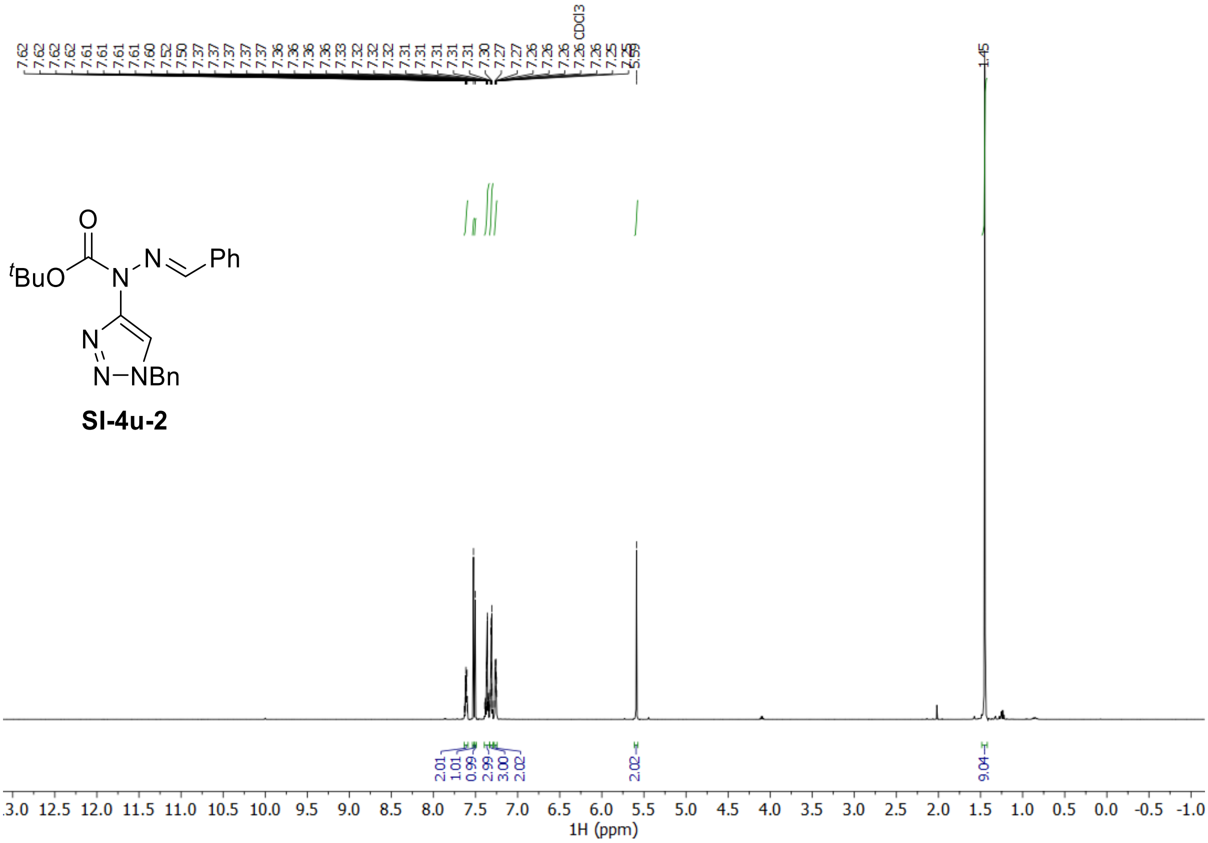

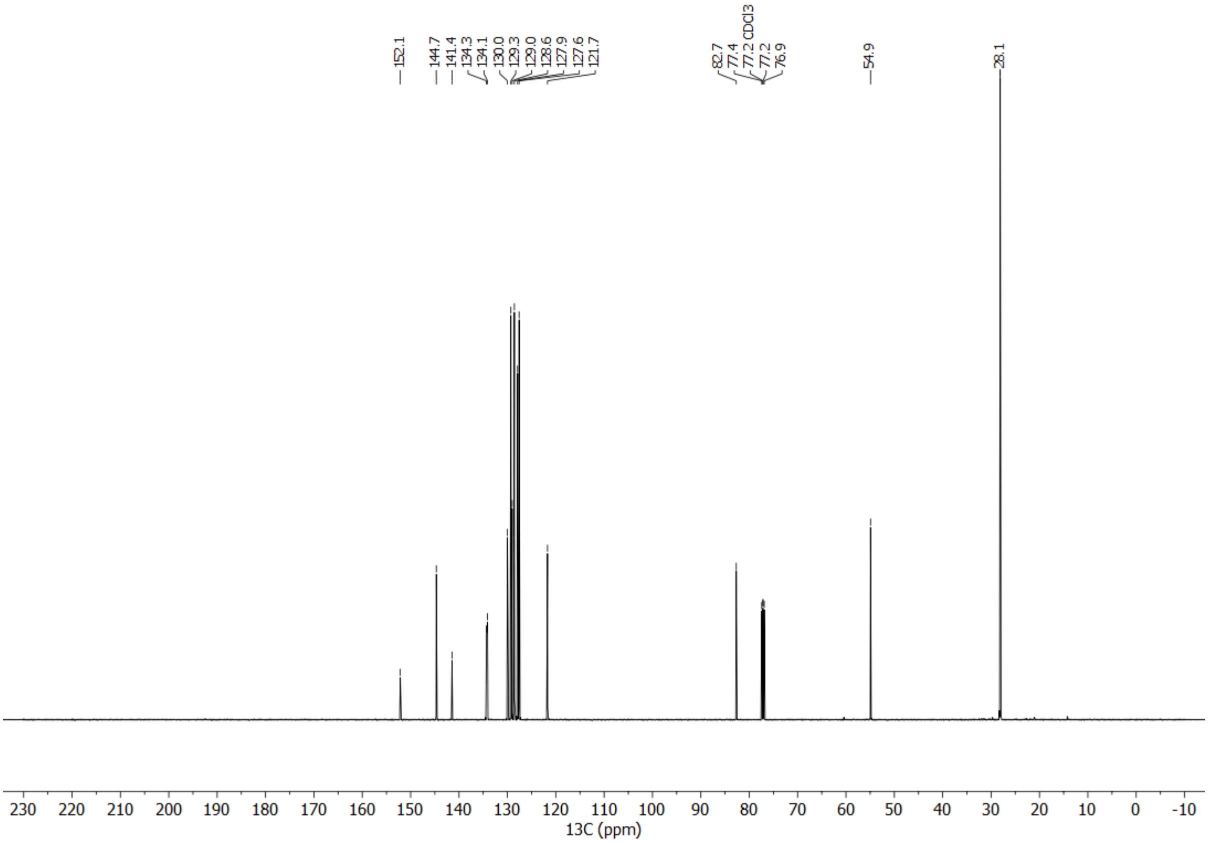

^{1}H NMR and ^{13}C NMR spectrum of compound **SI-4u-3**

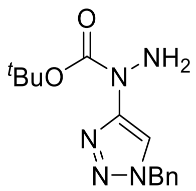

SI-4u-3

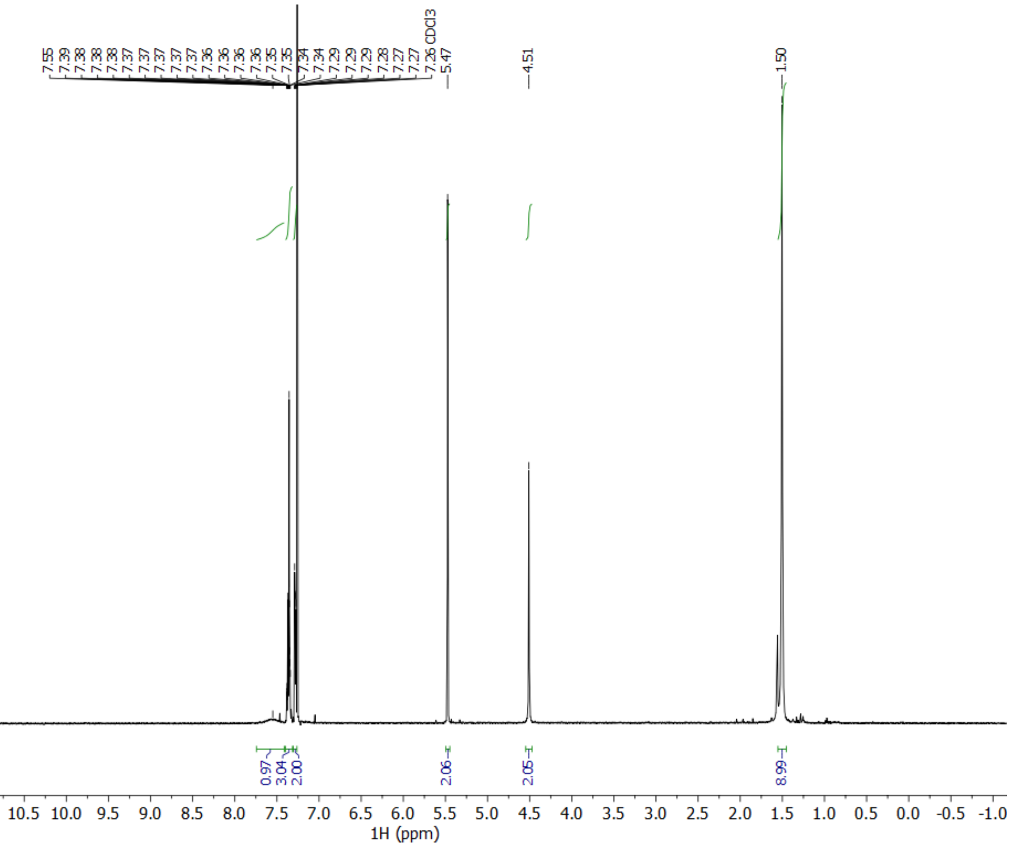

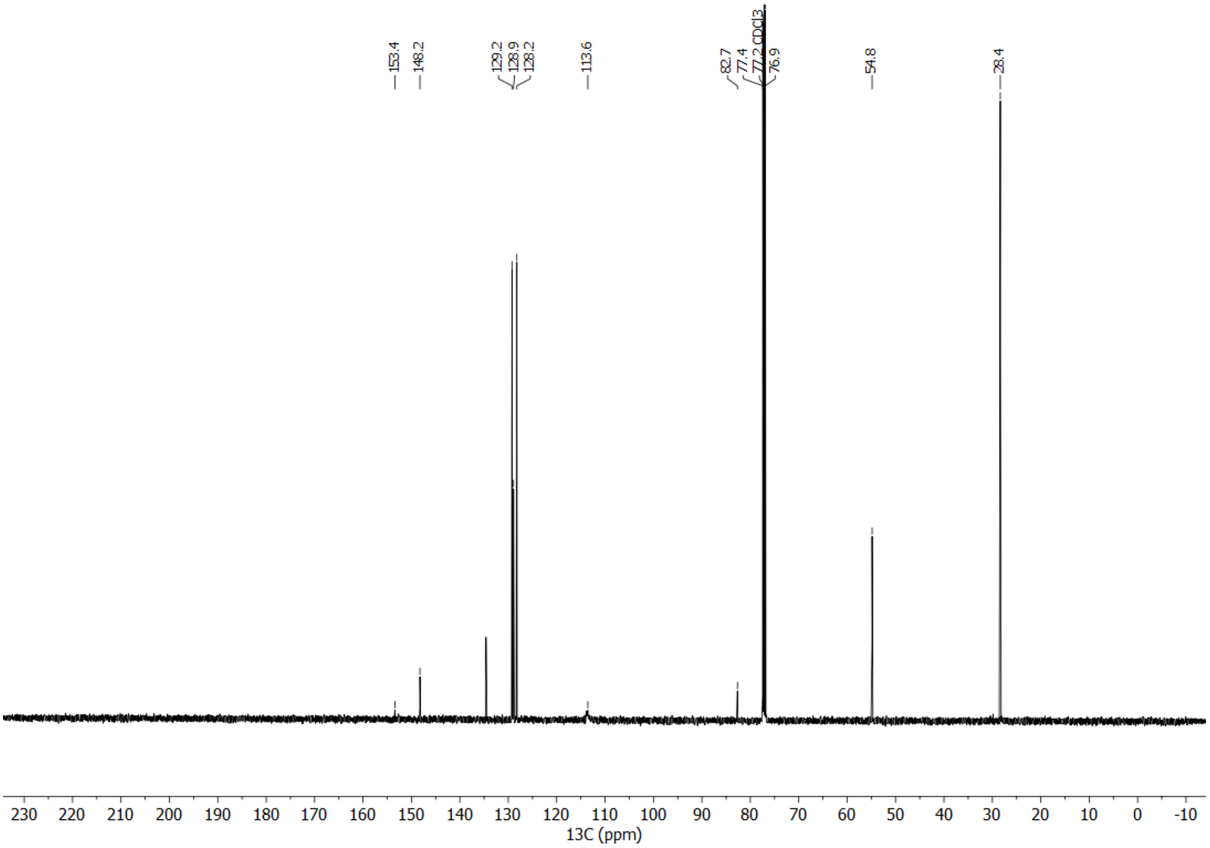

^1H NMR, ^{19}F NMR spectrum of compound **3a**, prepared according to **GP2.2**

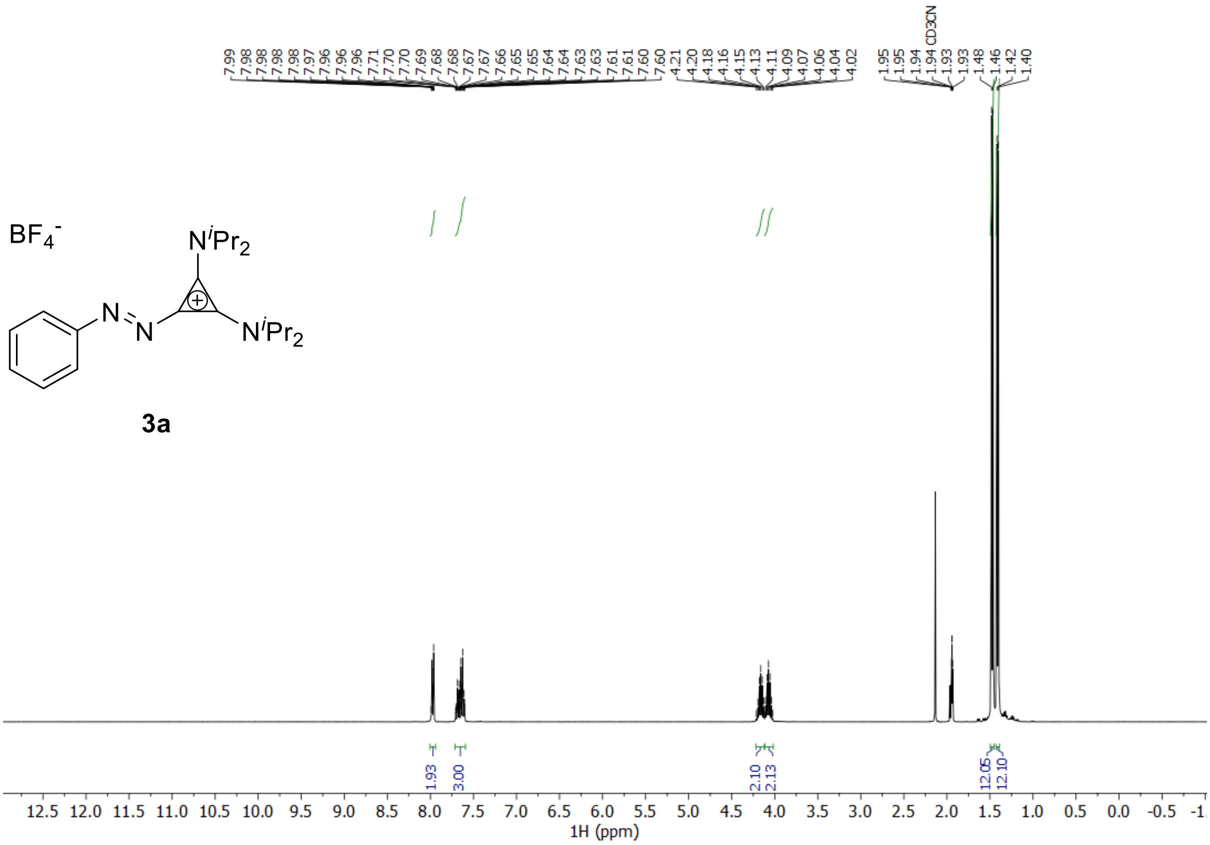

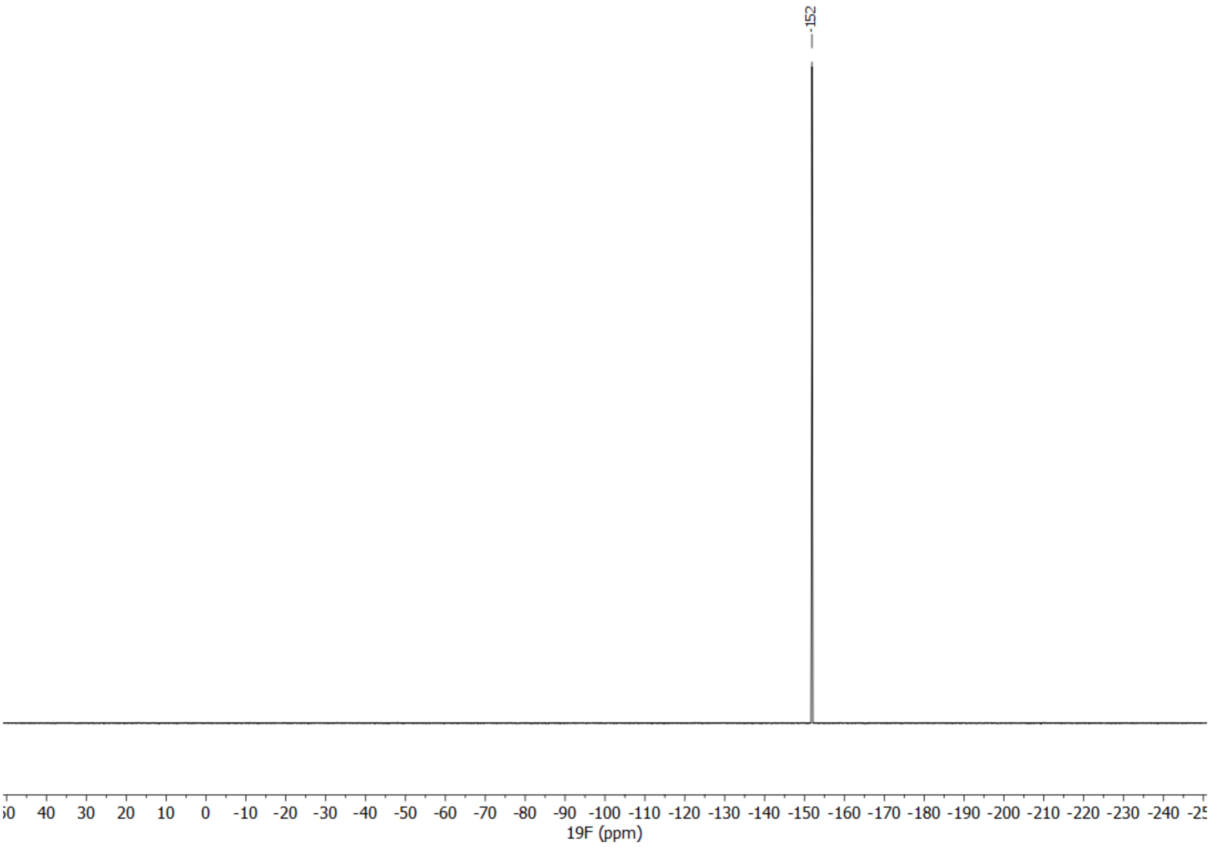

^{1}H NMR, ^{13}C NMR, and ^{19}F NMR spectrum of compound **3e**

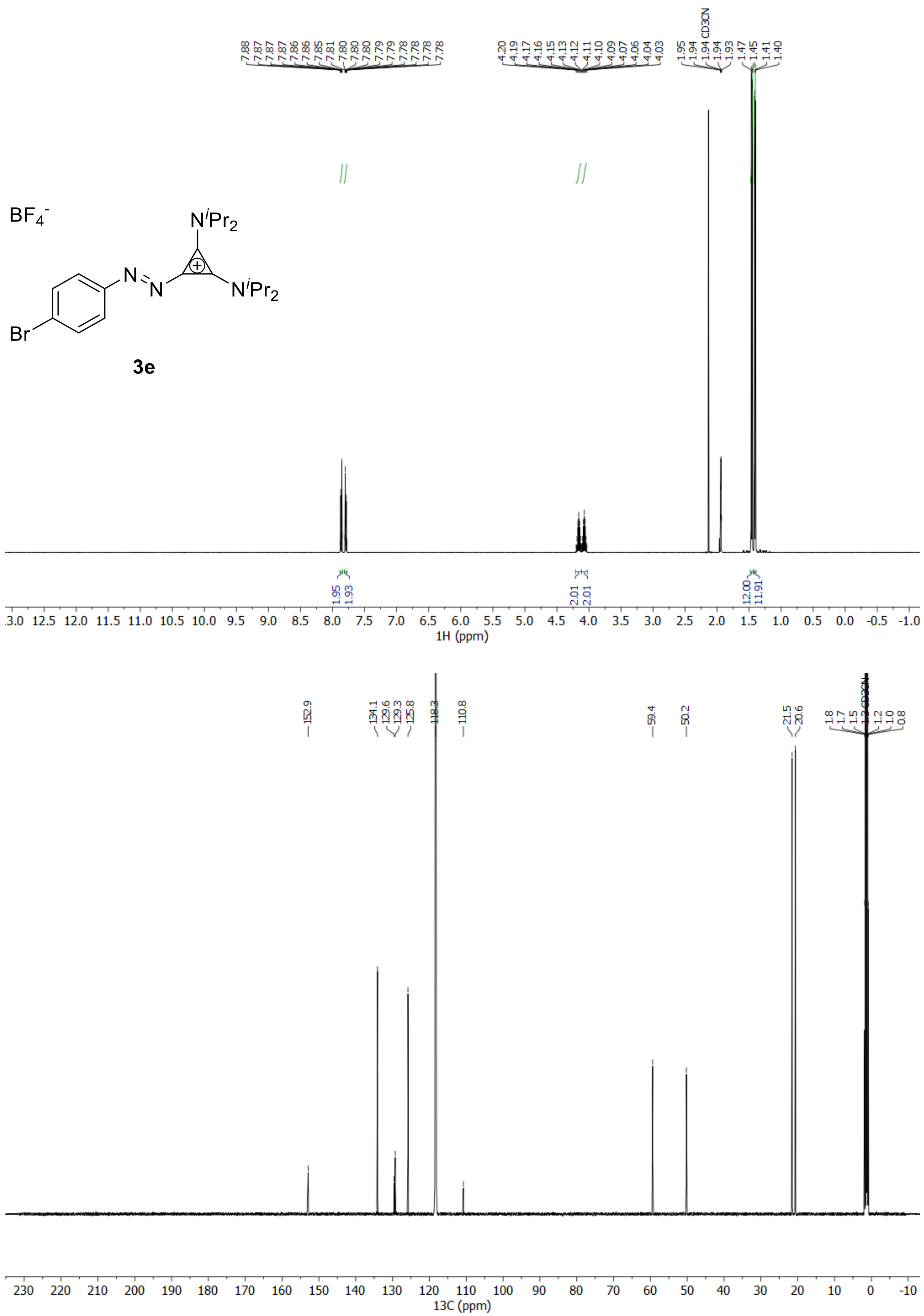

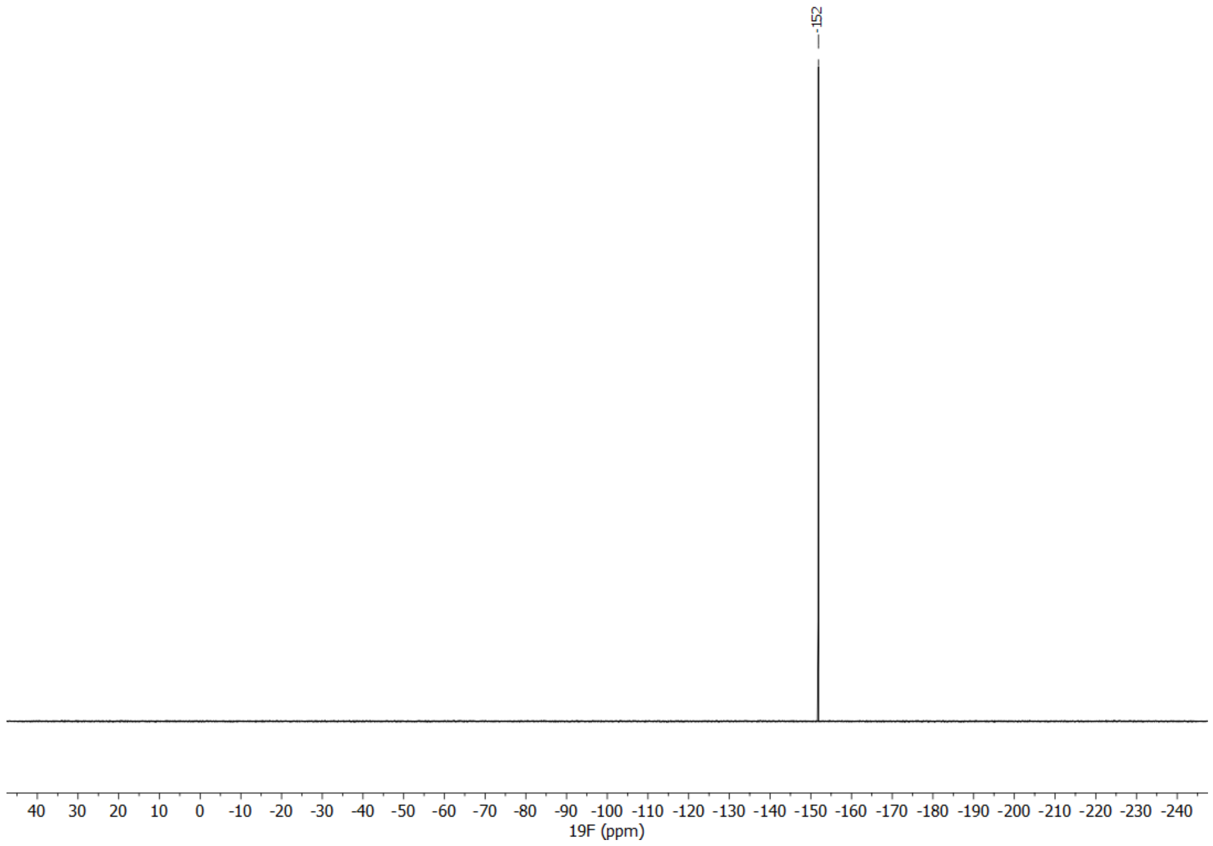
-152
40 30 20 10 0 -10 -20 -30 -40 -50 -60 -70 -80 -90 -100 -110 -120 -130 -140 -150 -160 -170 -180 -190 -200 -210 -220 -230 -240
19F (ppm)

^{1}H NMR, ^{13}C NMR, and ^{19}F NMR spectrum of compound **3f**

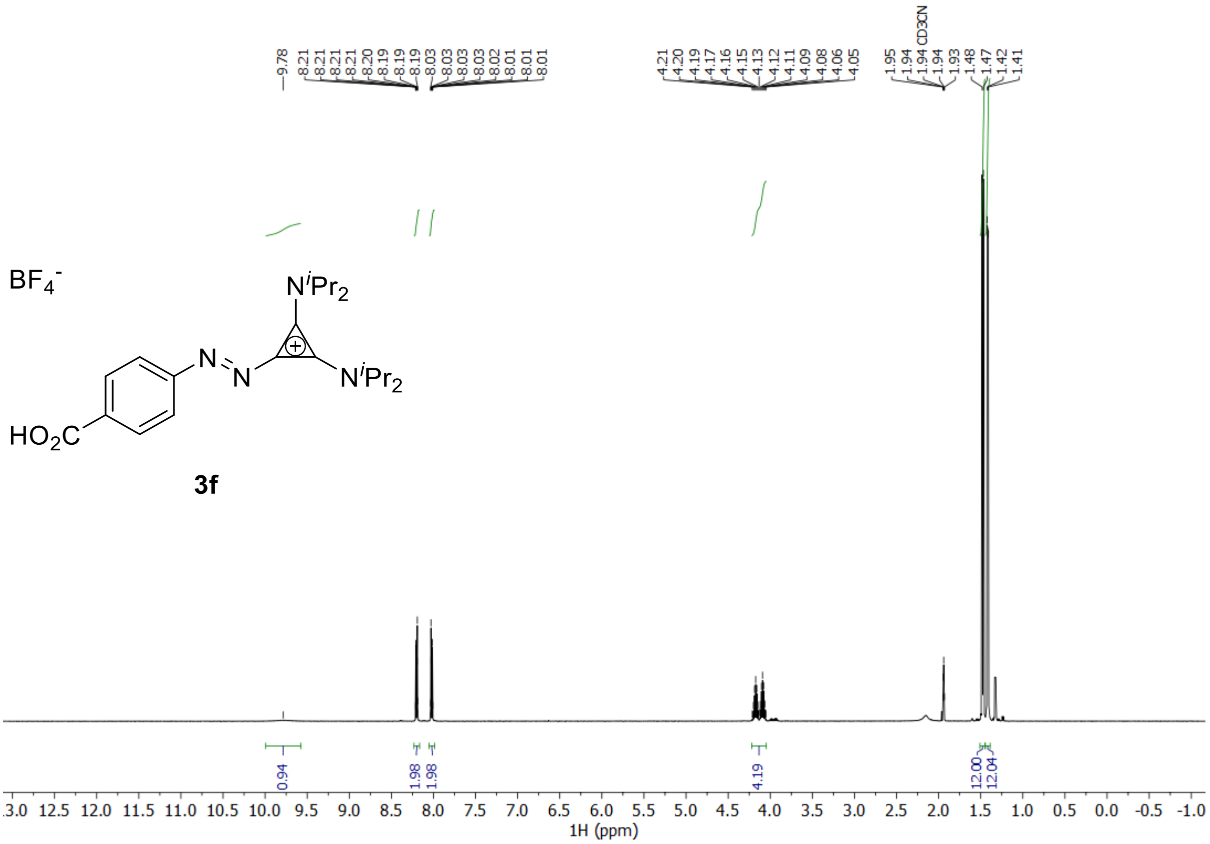

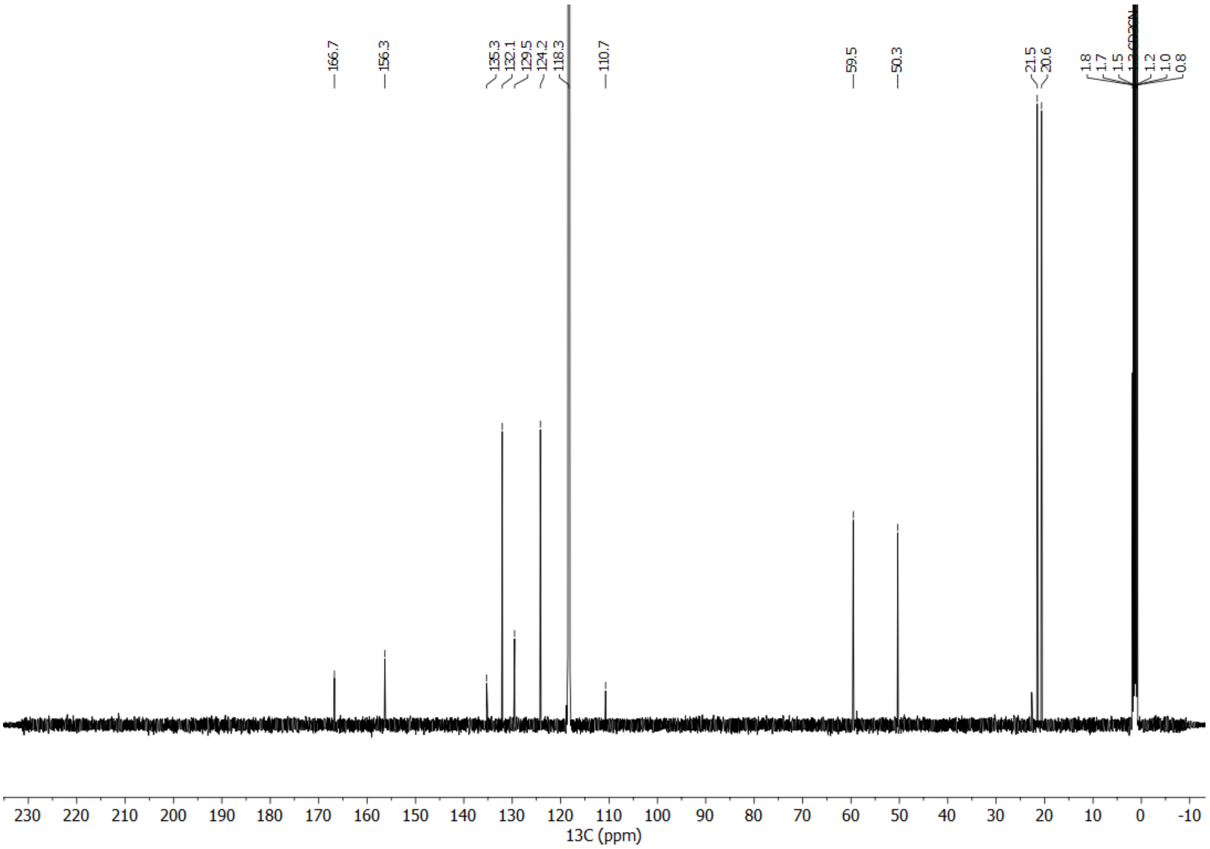

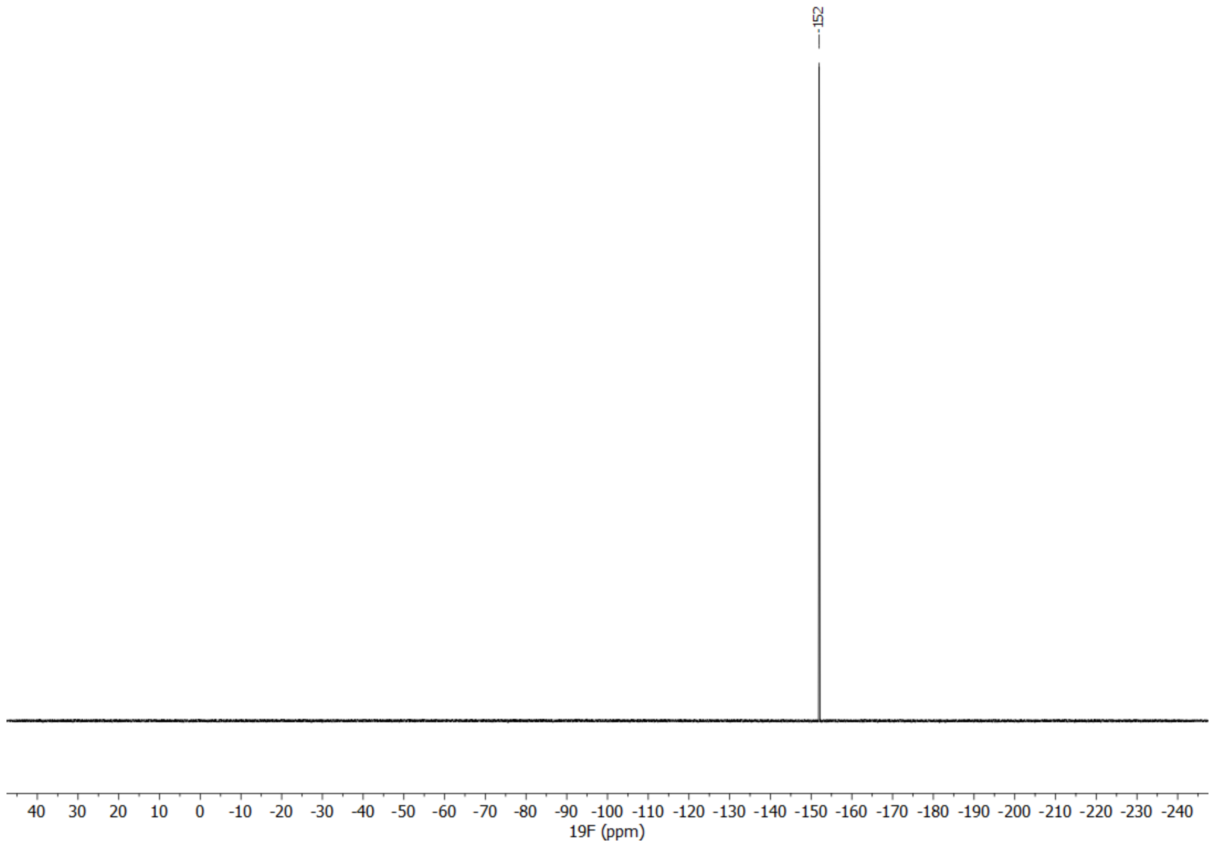
-152
40 30 20 10 0 -10 -20 -30 -40 -50 -60 -70 -80 -90 -100 -110 -120 -130 -140 -150 -160 -170 -180 -190 -200 -210 -220 -230 -240
19F (ppm)

^{1}H NMR, ^{13}C NMR, and ^{19}F NMR spectrum of compound **6a**

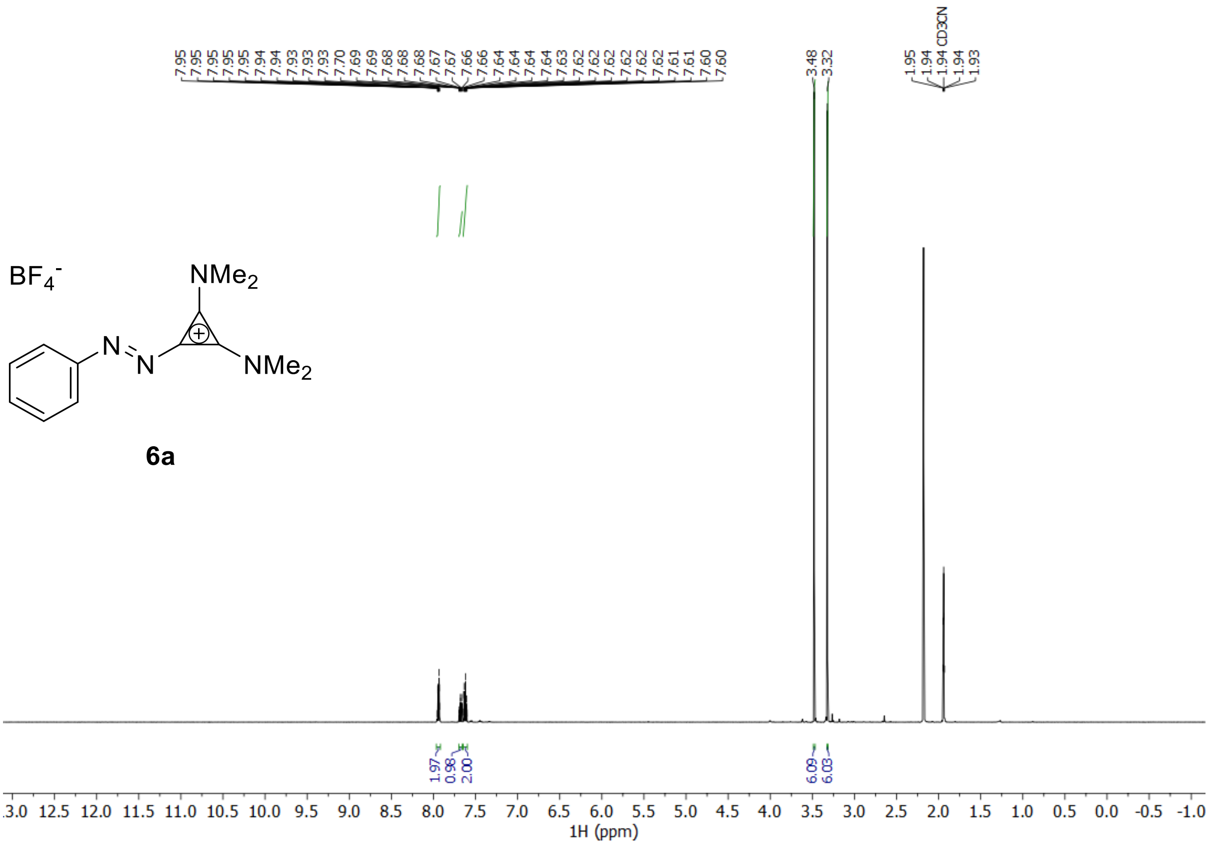

6a

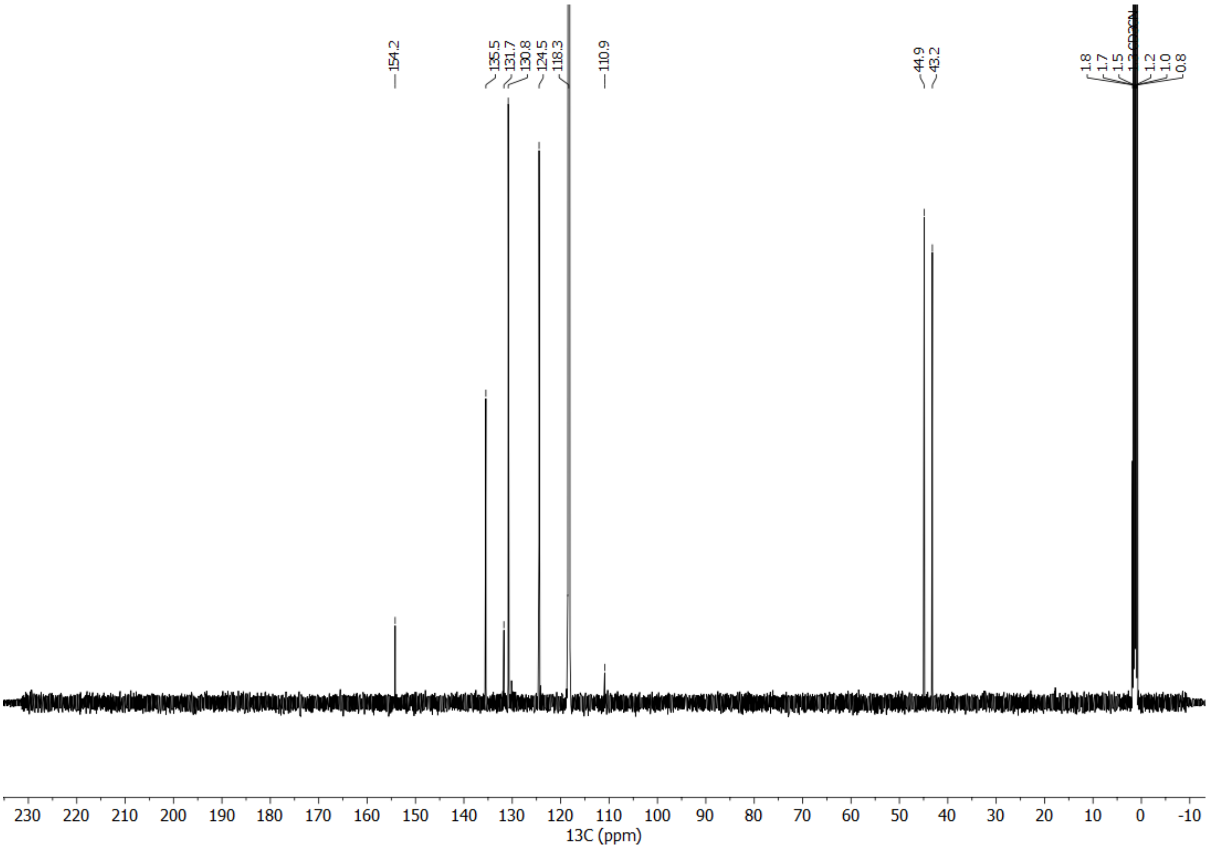

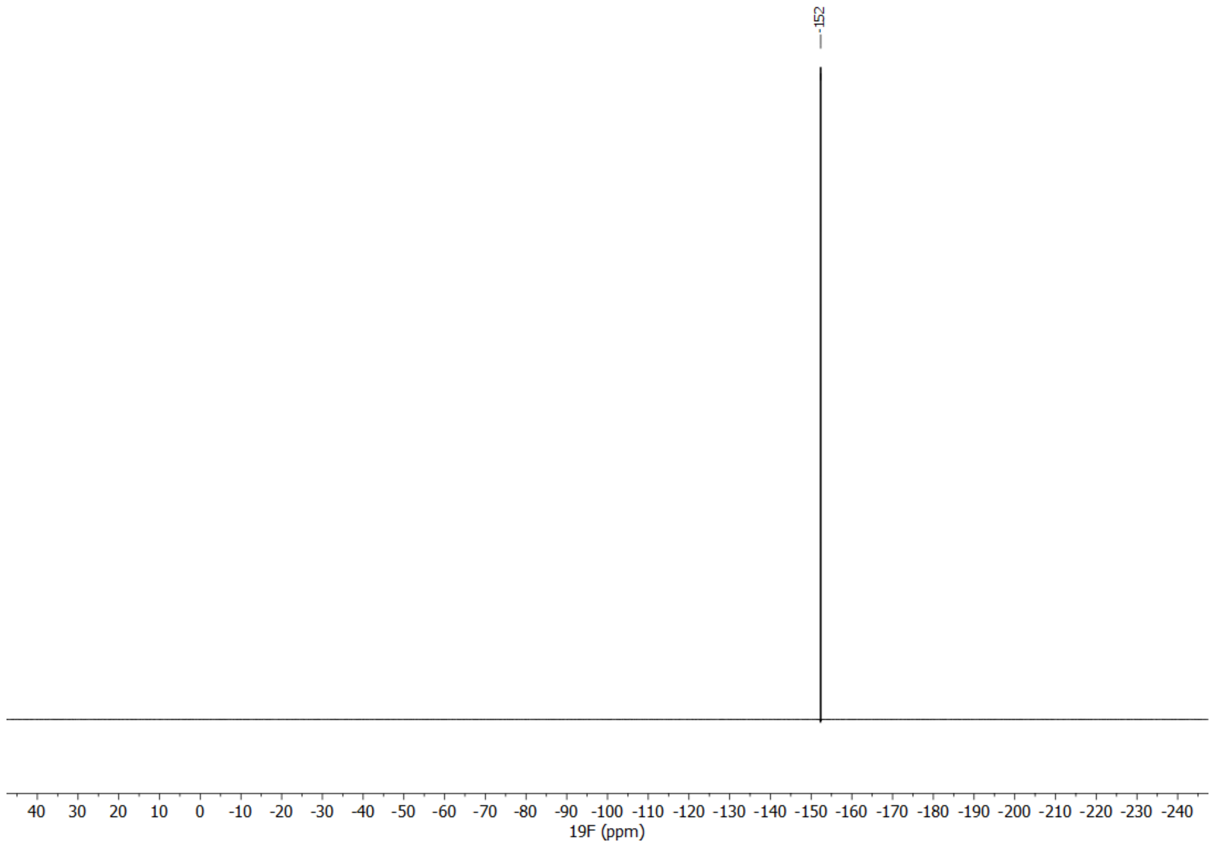
-152
40 30 20 10 0 -10 -20 -30 -40 -50 -60 -70 -80 -90 -100 -110 -120 -130 -140 -150 -160 -170 -180 -190 -200 -210 -220 -230 -240
19F (ppm)

^{1}H NMR, ^{13}C NMR, and ^{19}F NMR spectrum of compound **6b**

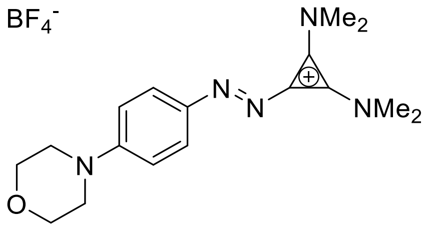

6b

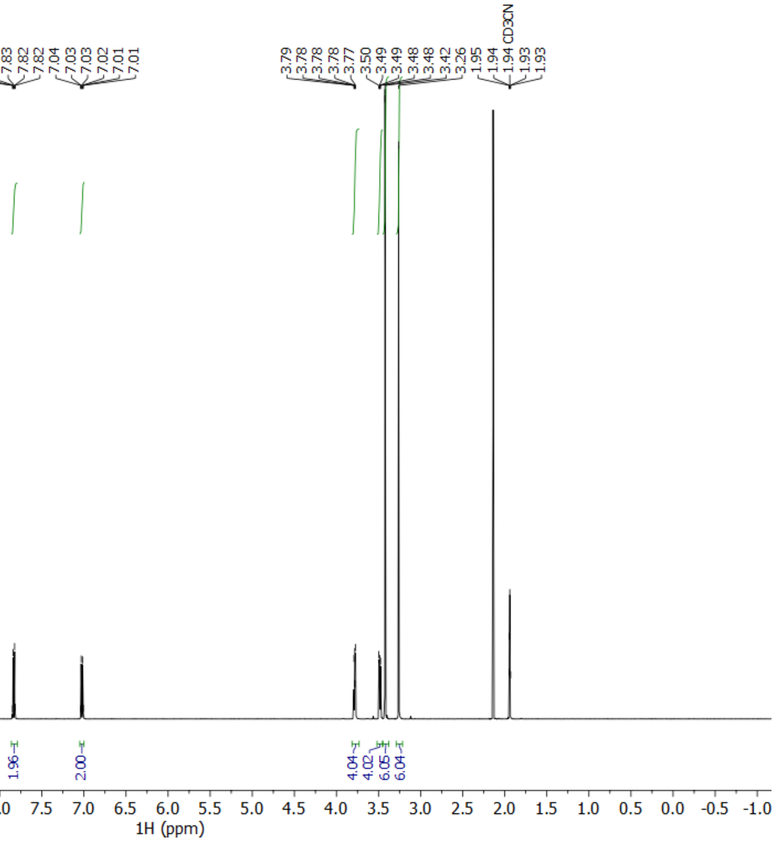

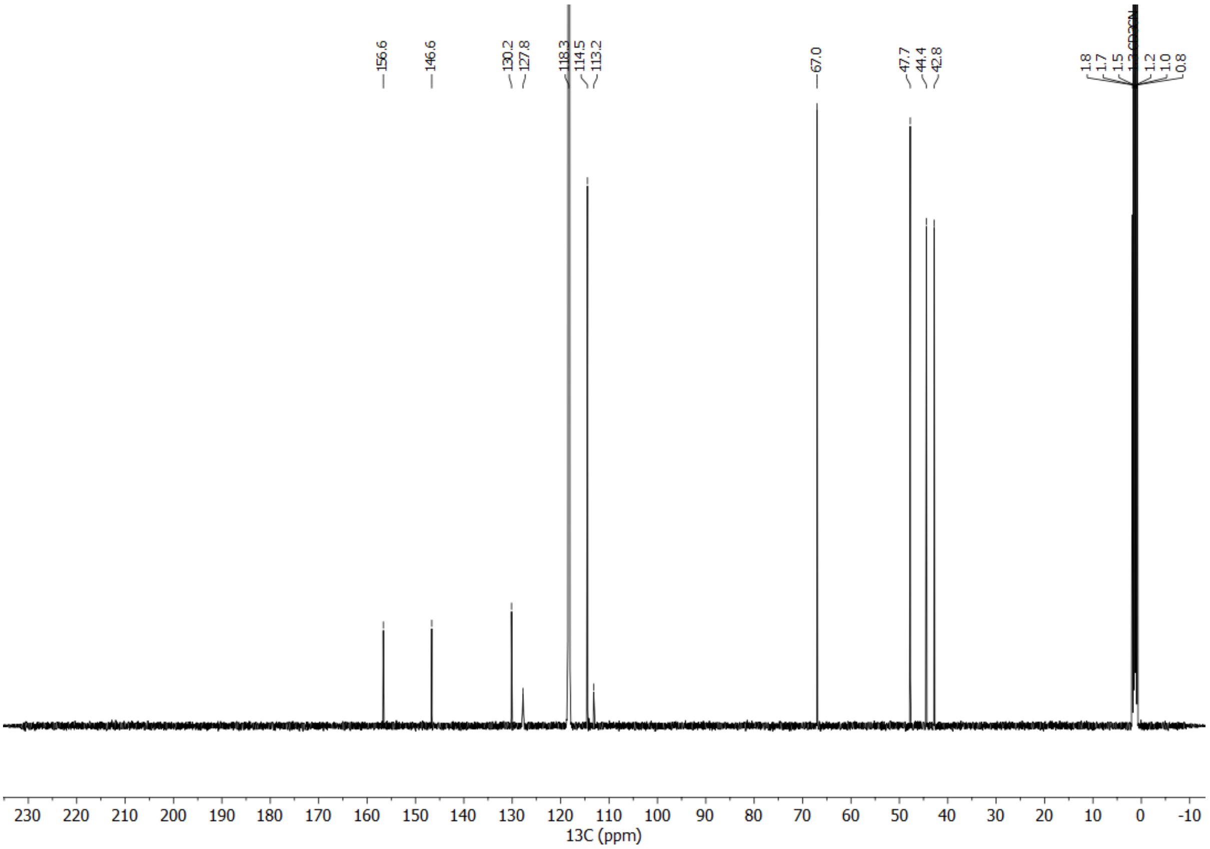

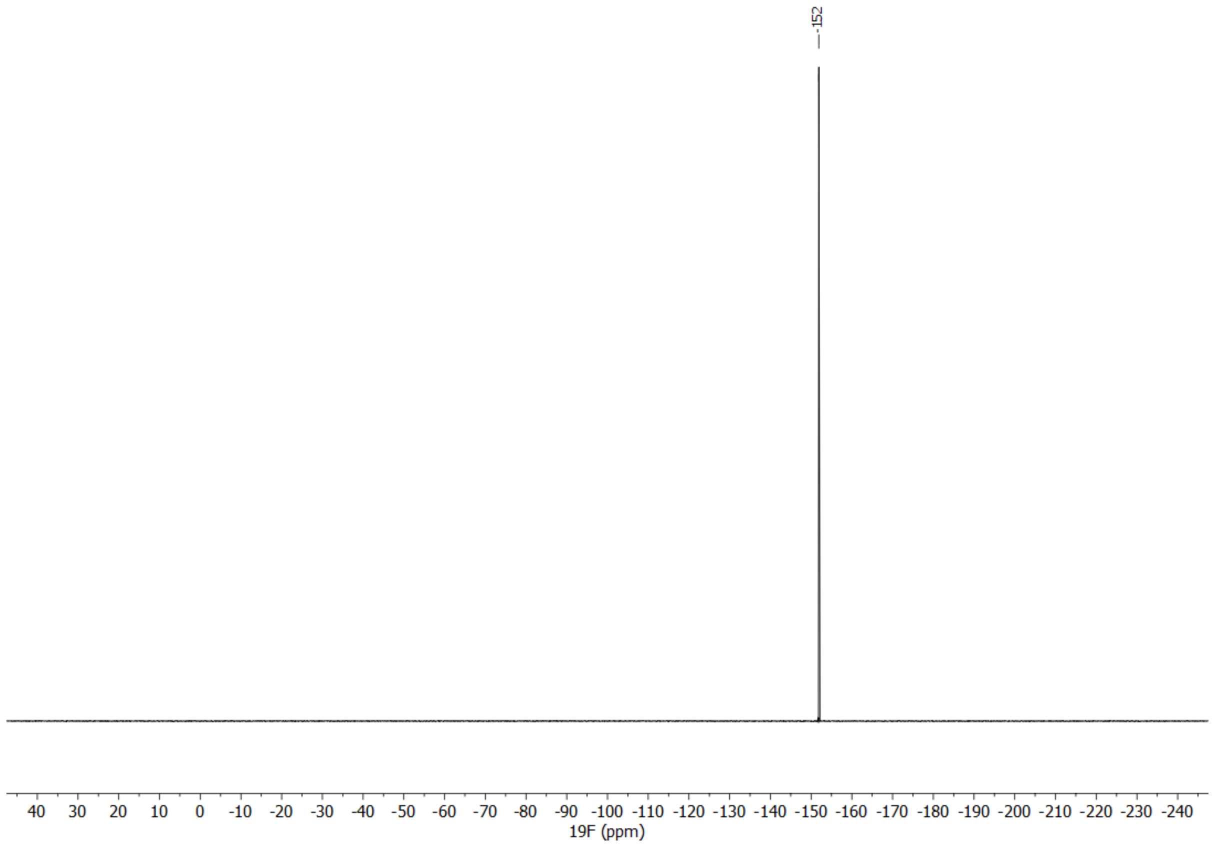
-152
40 30 20 10 0 -10 -20 -30 -40 -50 -60 -70 -80 -90 -100 -110 -120 -130 -140 -150 -160 -170 -180 -190 -200 -210 -220 -230 -240
19F (ppm)

^{1}H NMR, ^{13}C NMR, and ^{19}F NMR spectrum of compound **6c**

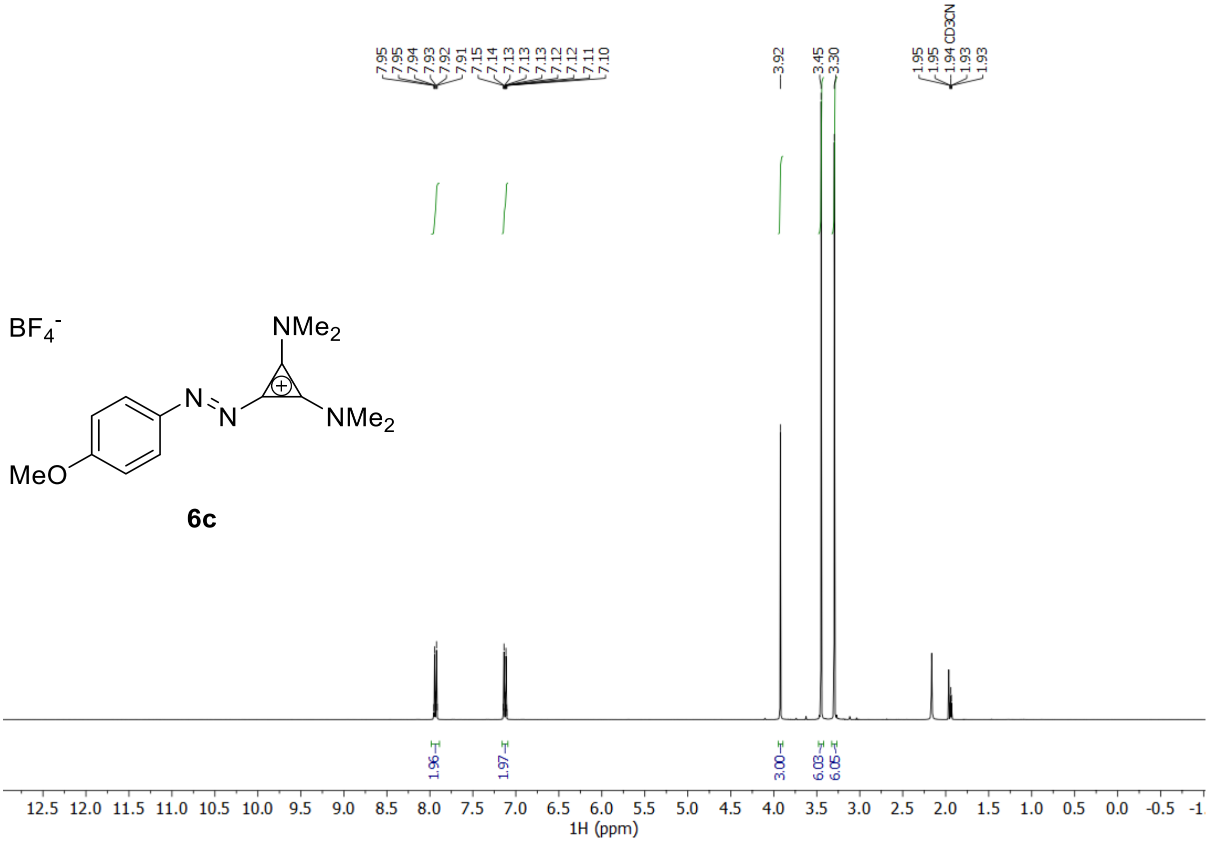

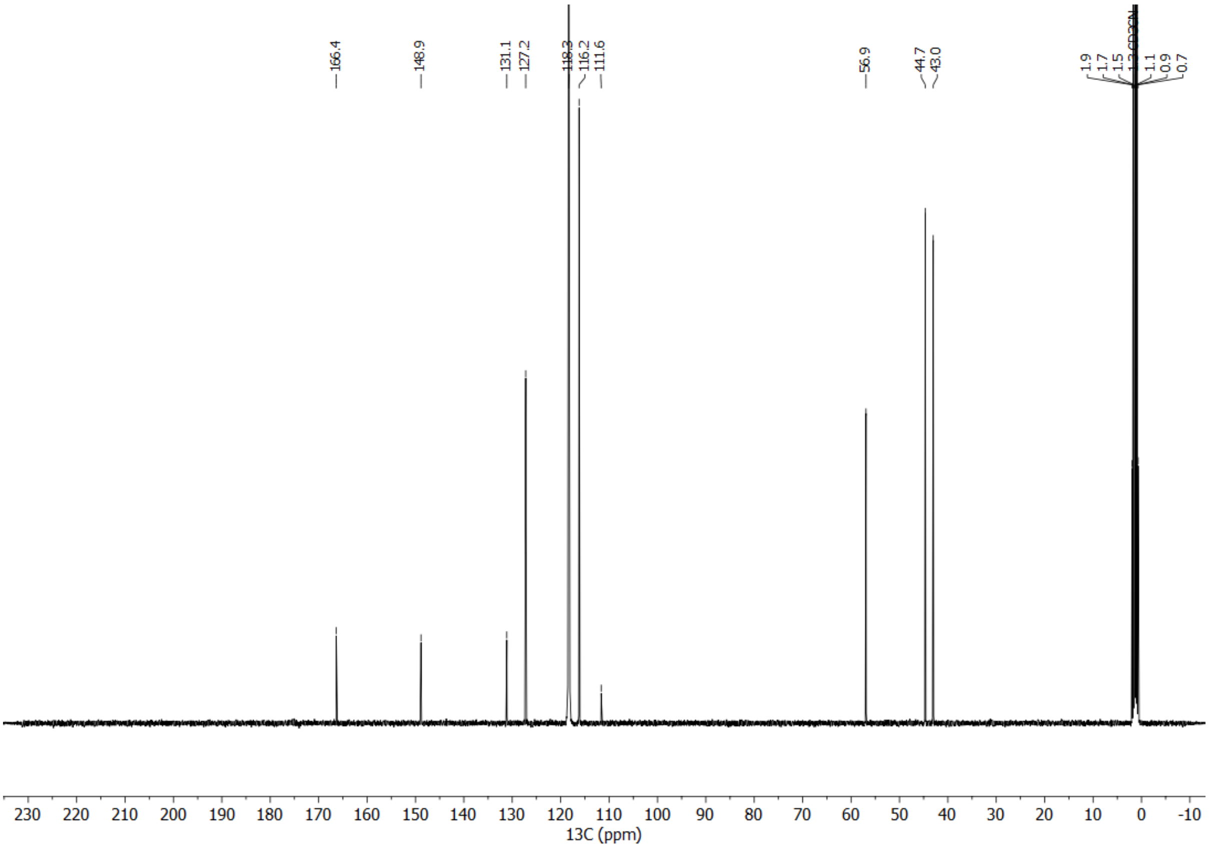

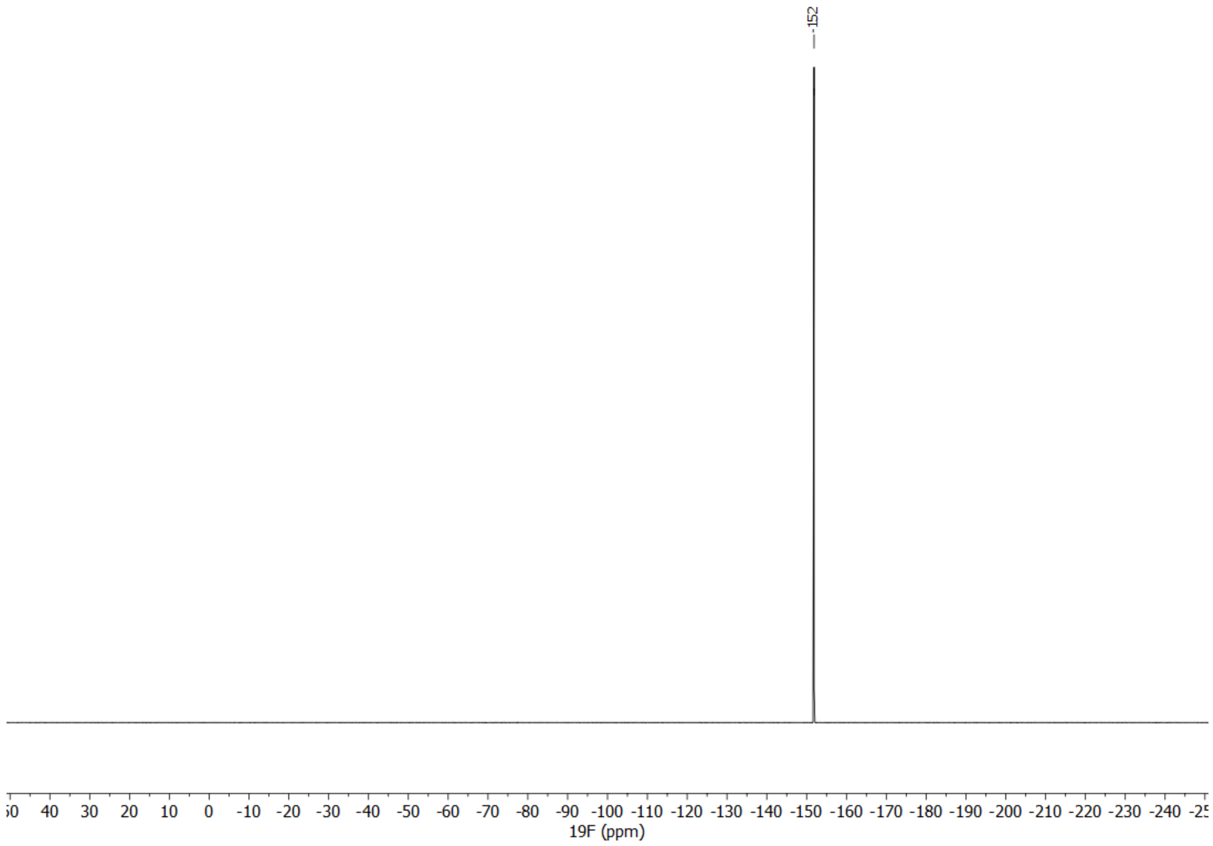
-152
50 40 30 20 10 0 -10 -20 -30 -40 -50 -60 -70 -80 -90 -100 -110 -120 -130 -140 -150 -160 -170 -180 -190 -200 -210 -220 -230 -240 -25
19F (ppm)

^{1}H NMR, ^{13}C NMR, and ^{19}F NMR spectrum of compound **6d**

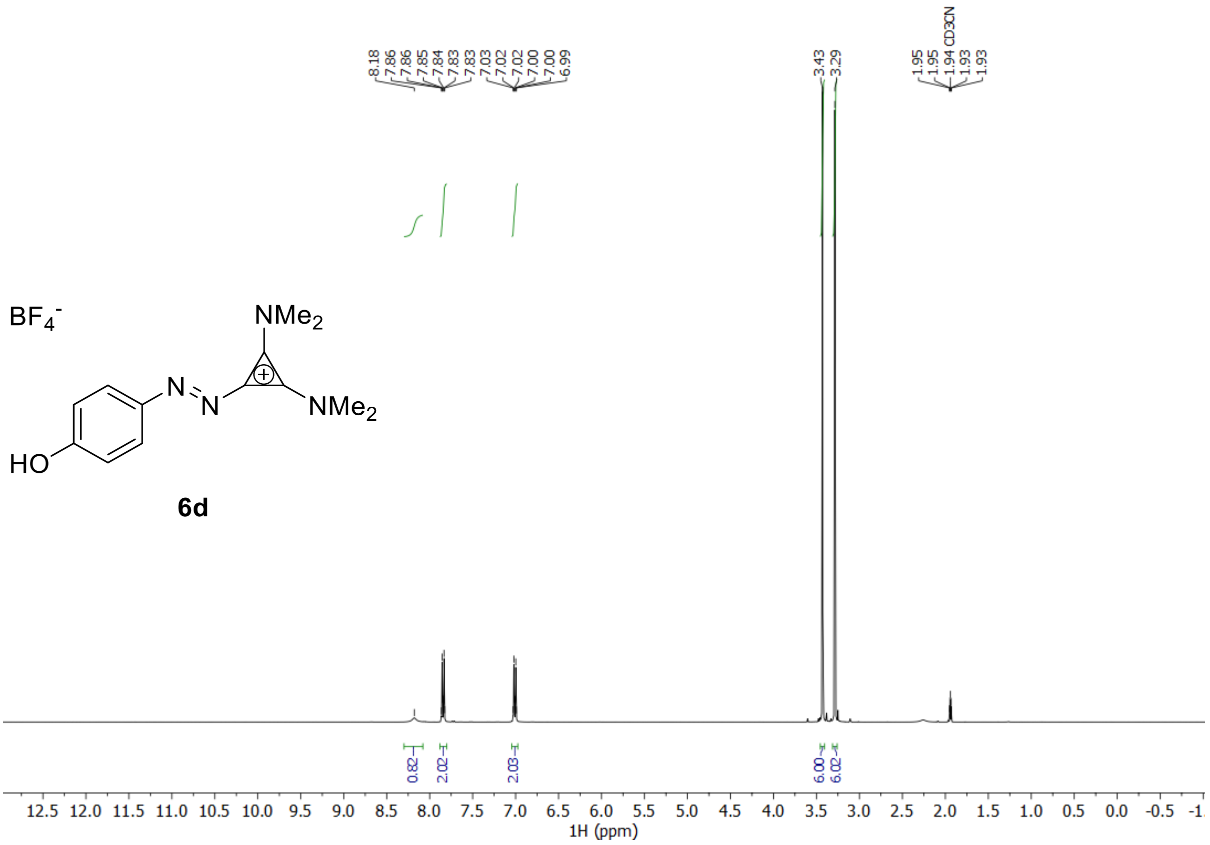

6d

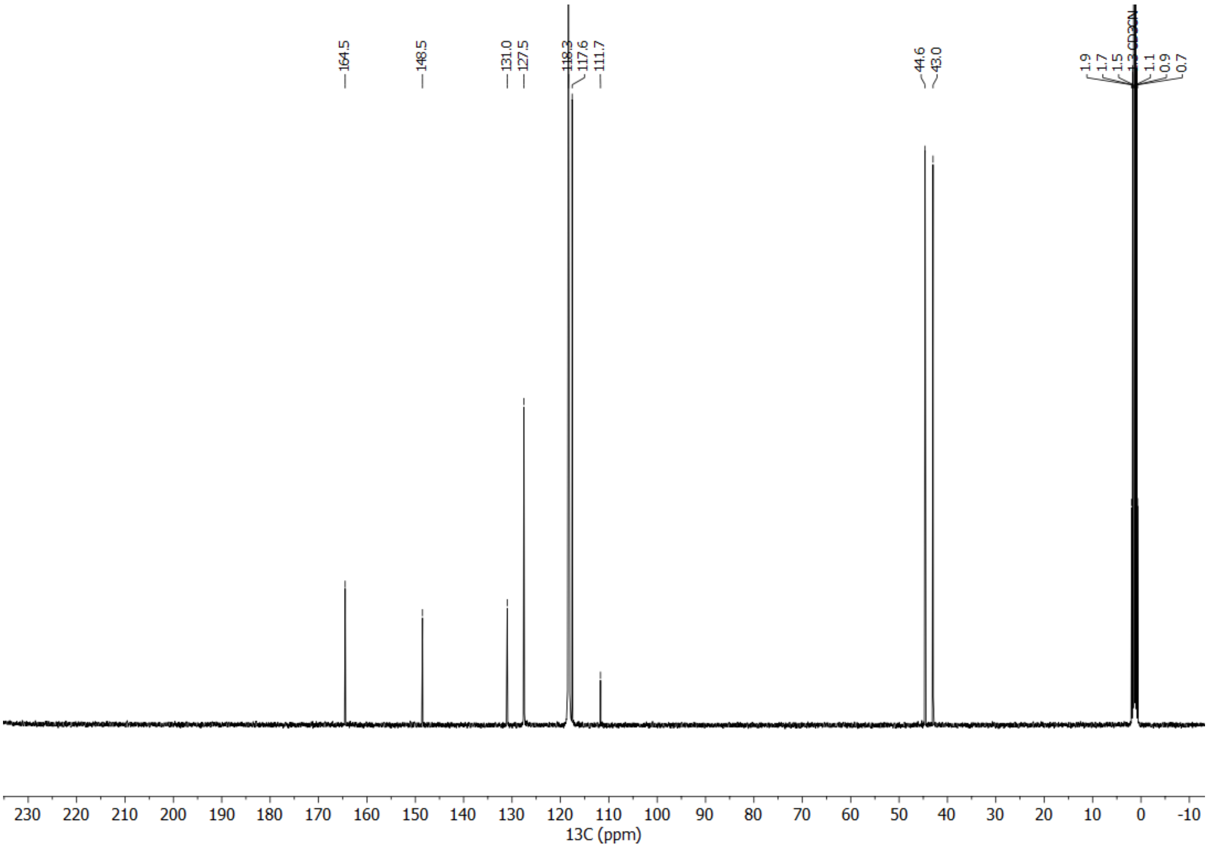

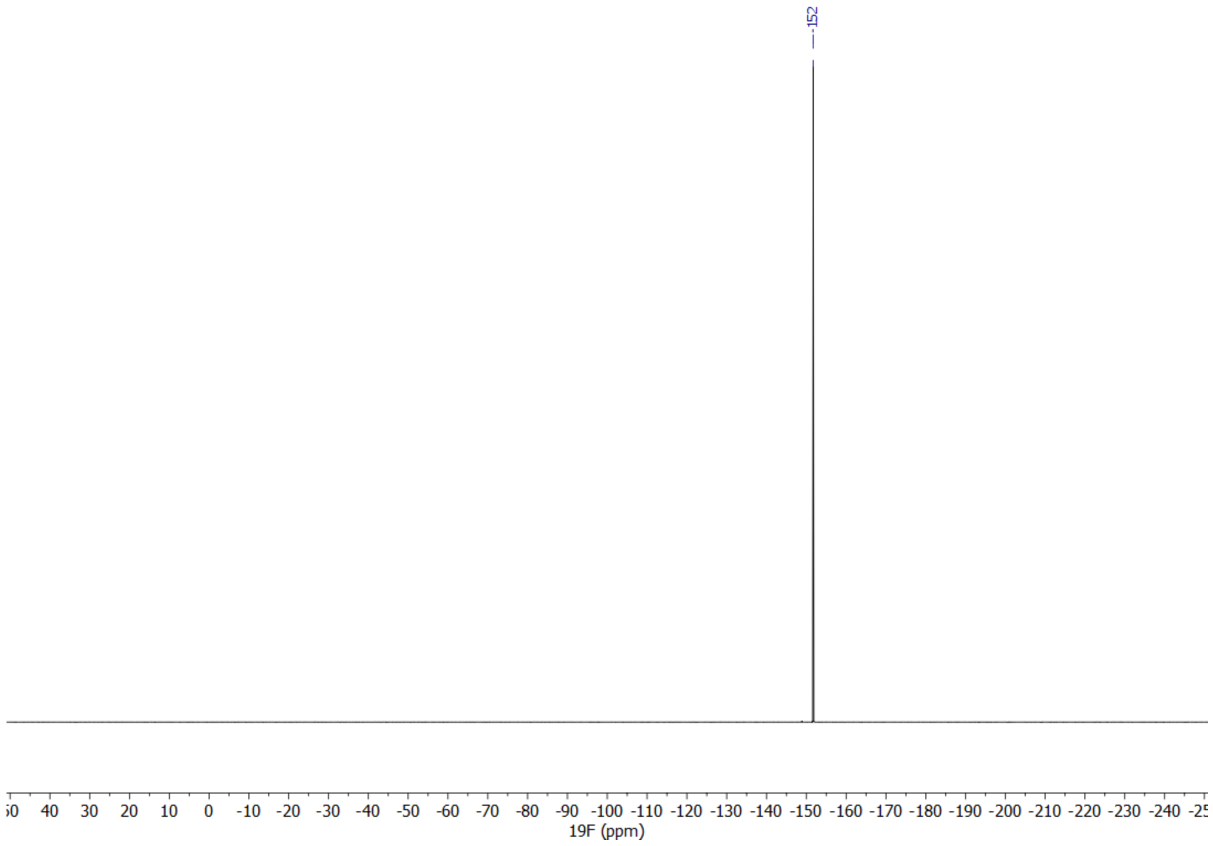

--152
50 40 30 20 10 0 -10 -20 -30 -40 -50 -60 -70 -80 -90 -100 -110 -120 -130 -140 -150 -160 -170 -180 -190 -200 -210 -220 -230 -240 -25
19F (ppm)

^{1}H NMR, ^{13}C NMR, and ^{19}F NMR spectrum of compound **6e**

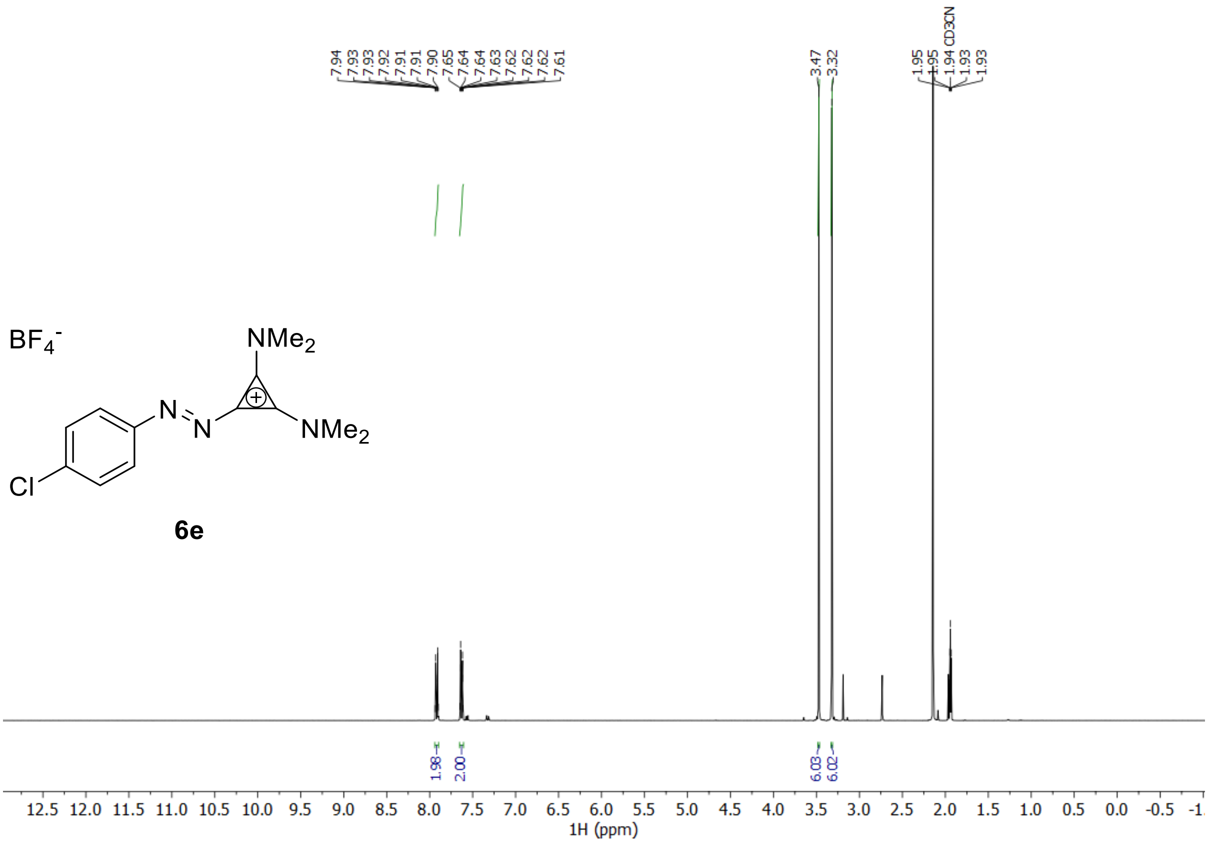

6e

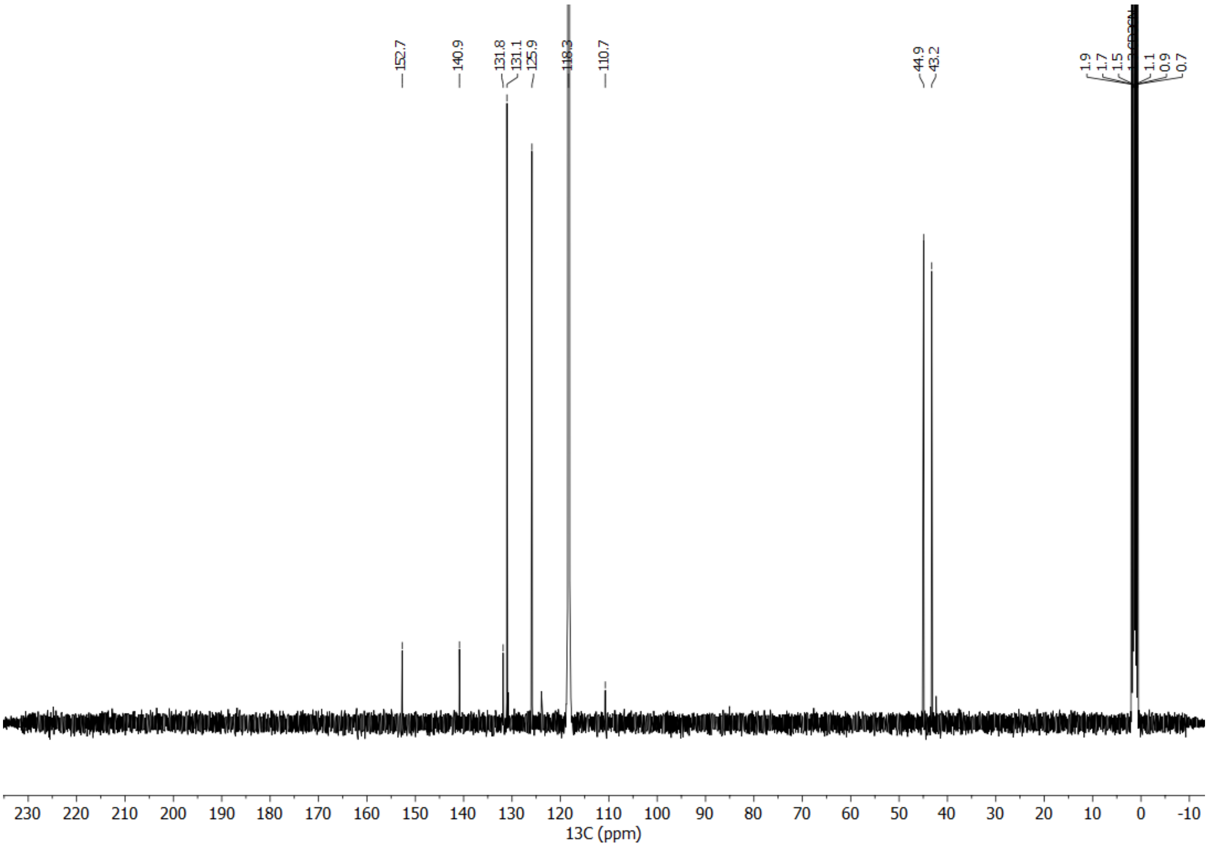

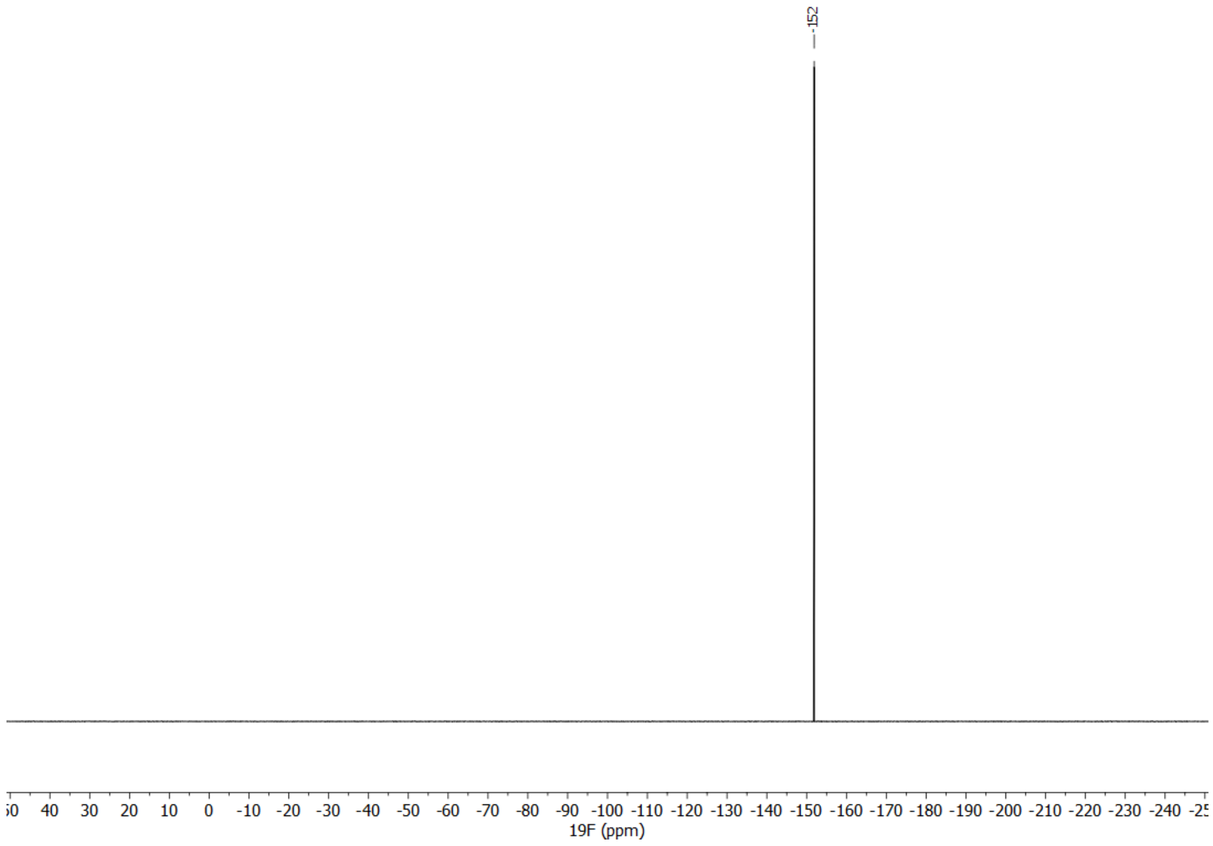

-152
50 40 30 20 10 0 -10 -20 -30 -40 -50 -60 -70 -80 -90 -100 -110 -120 -130 -140 -150 -160 -170 -180 -190 -200 -210 -220 -230 -240 -250
19F (ppm)

^{1}H NMR, ^{13}C NMR, and ^{19}F NMR spectrum of compound **6f**

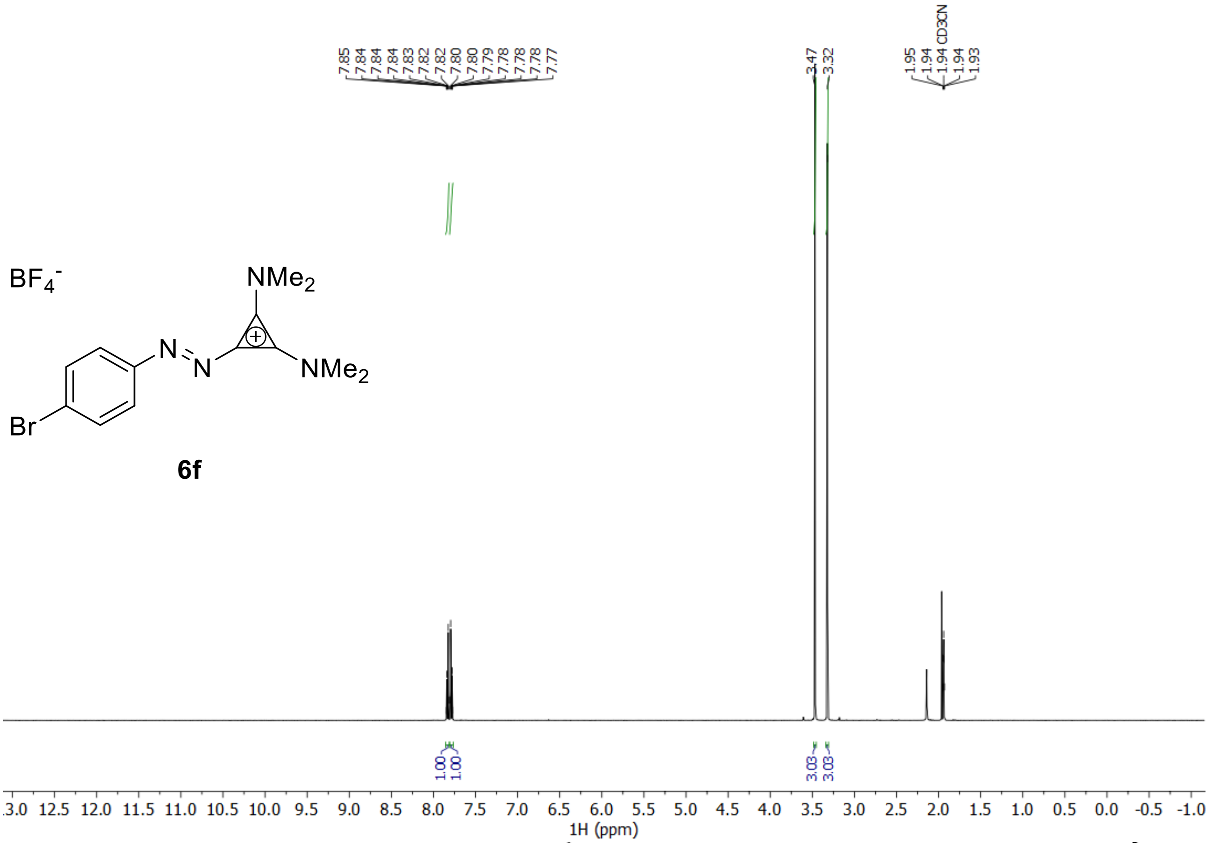

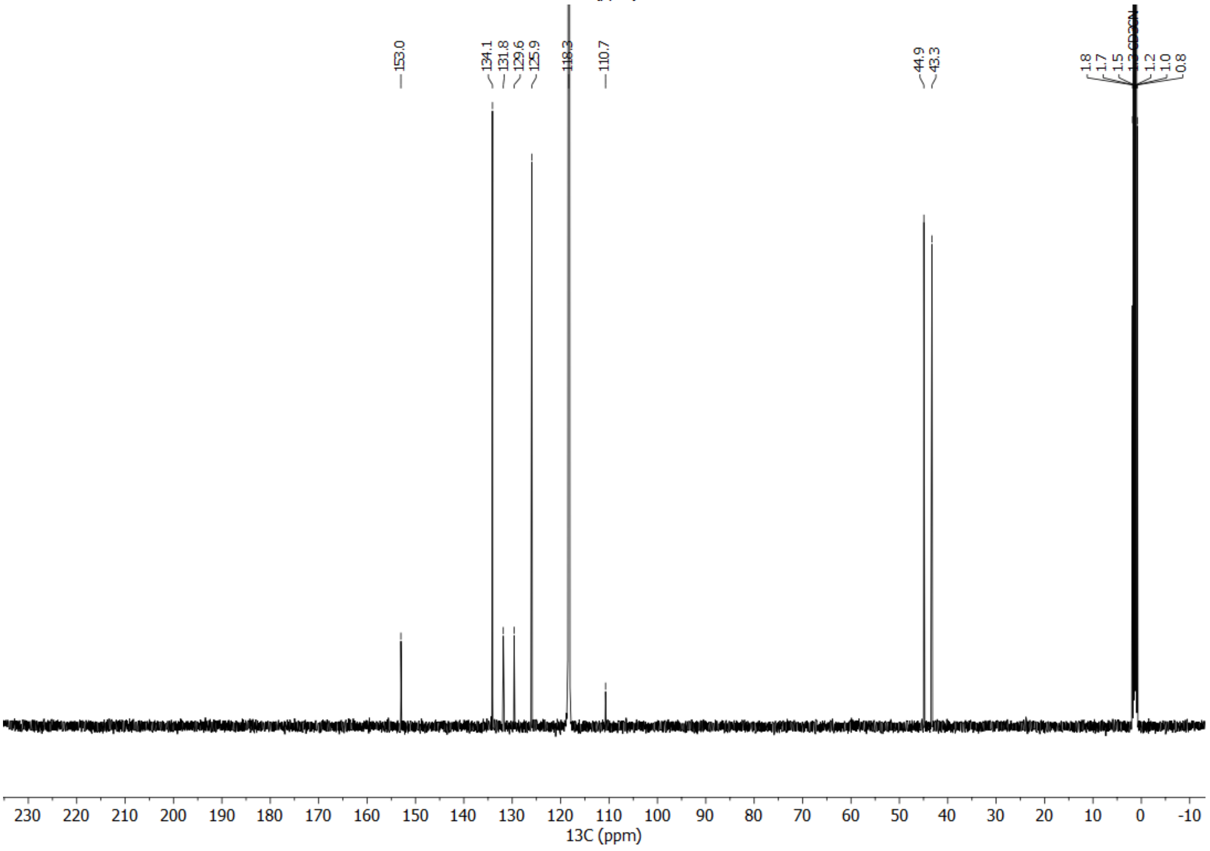

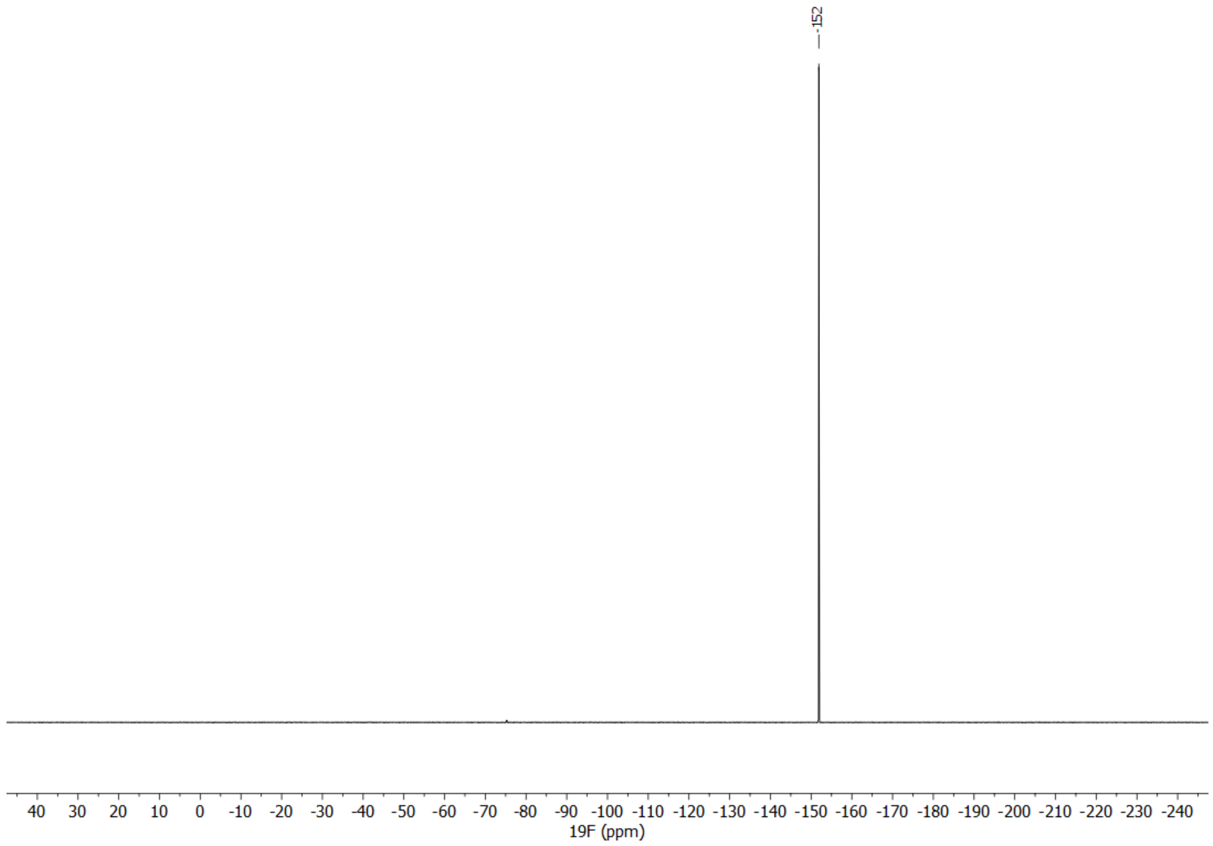
-152
40 30 20 10 0 -10 -20 -30 -40 -50 -60 -70 -80 -90 -100 -110 -120 -130 -140 -150 -160 -170 -180 -190 -200 -210 -220 -230 -240
19F (ppm)

^{1}H NMR, ^{13}C NMR, and ^{19}F NMR spectrum of compound **6g**

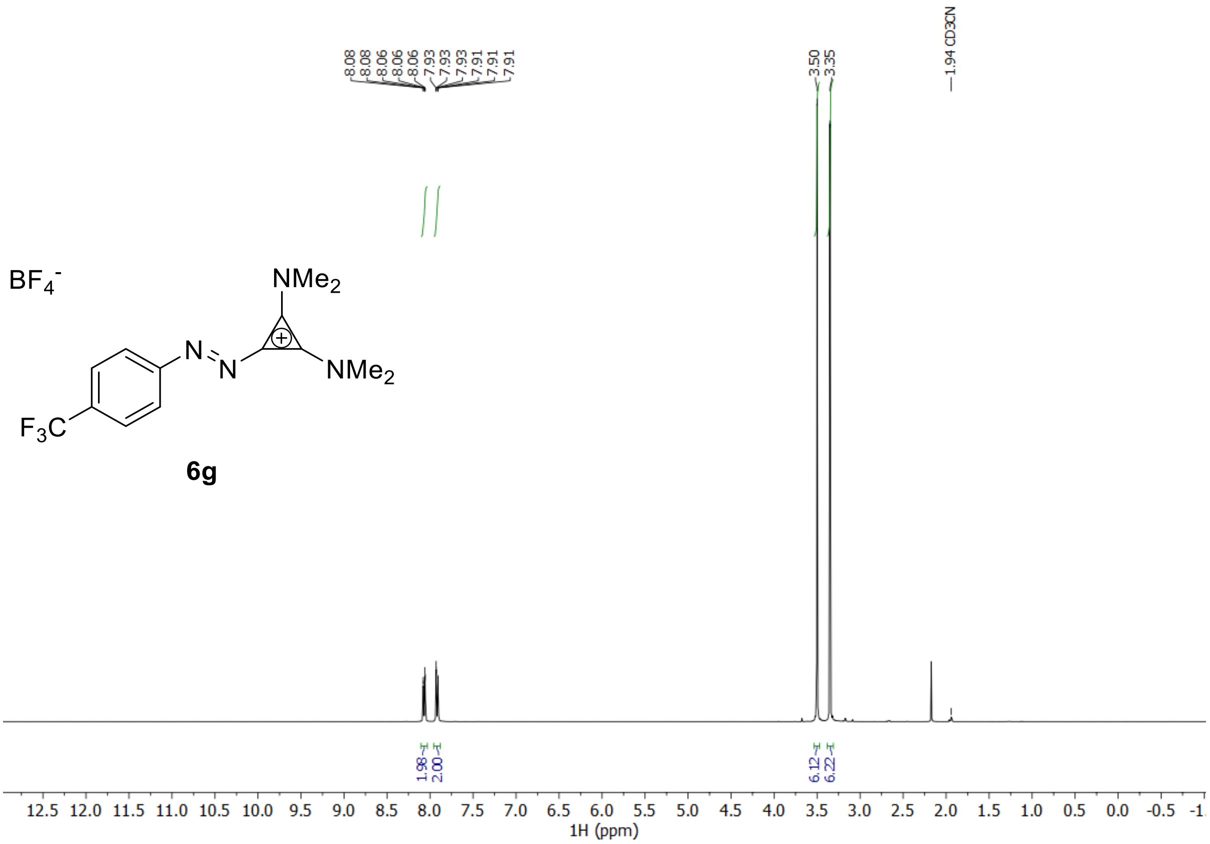

6g

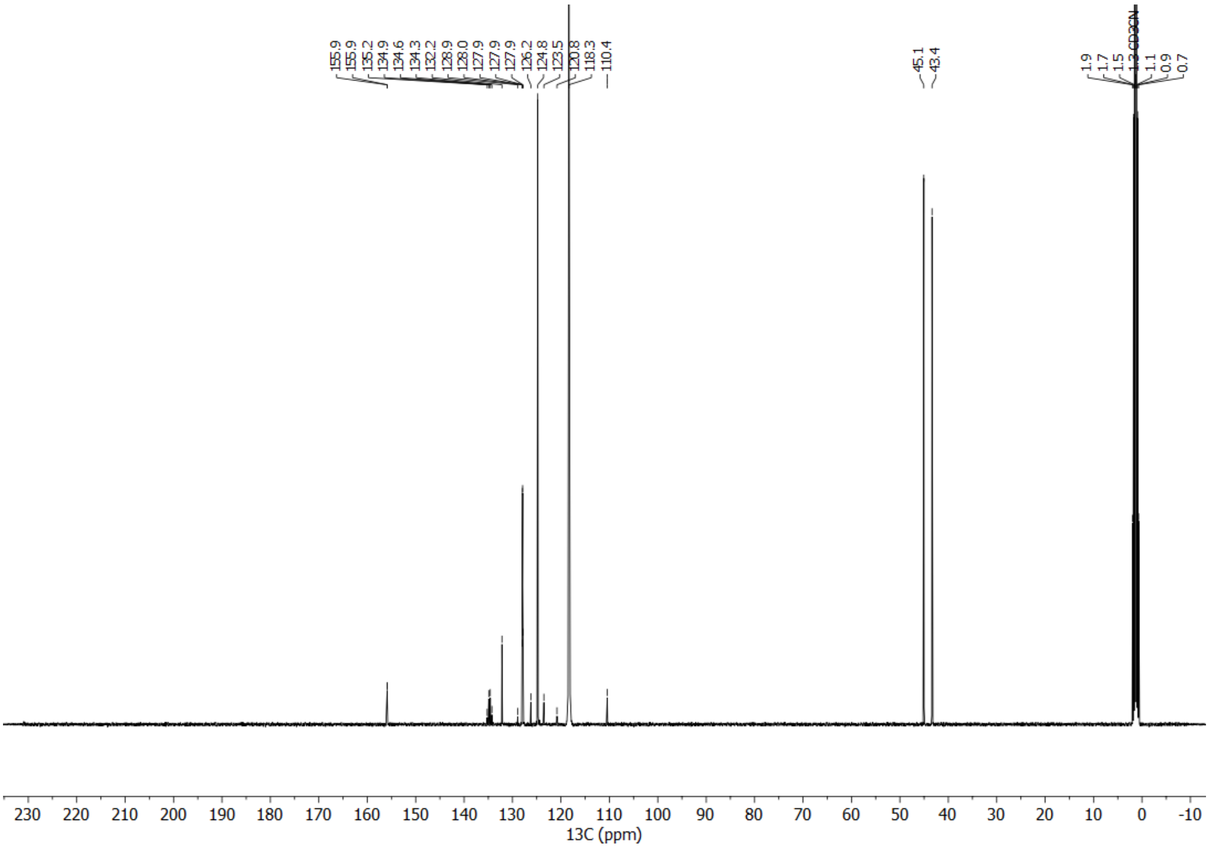

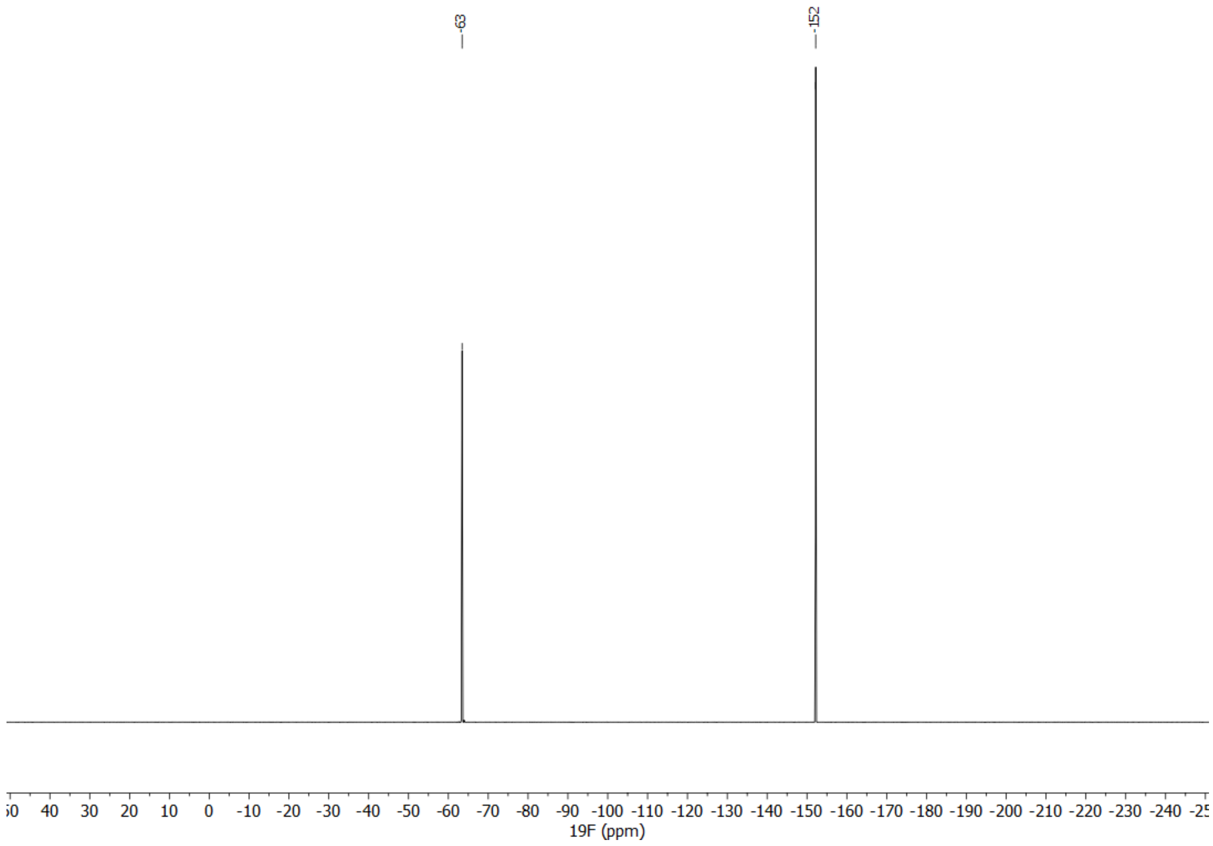
-63
-152
40 30 20 10 0 -10 -20 -30 -40 -50 -60 -70 -80 -90 -100 -110 -120 -130 -140 -150 -160 -170 -180 -190 -200 -210 -220 -230 -240
19F (ppm)

^{1}H NMR, ^{13}C NMR, and ^{19}F NMR spectrum of compound **6h**

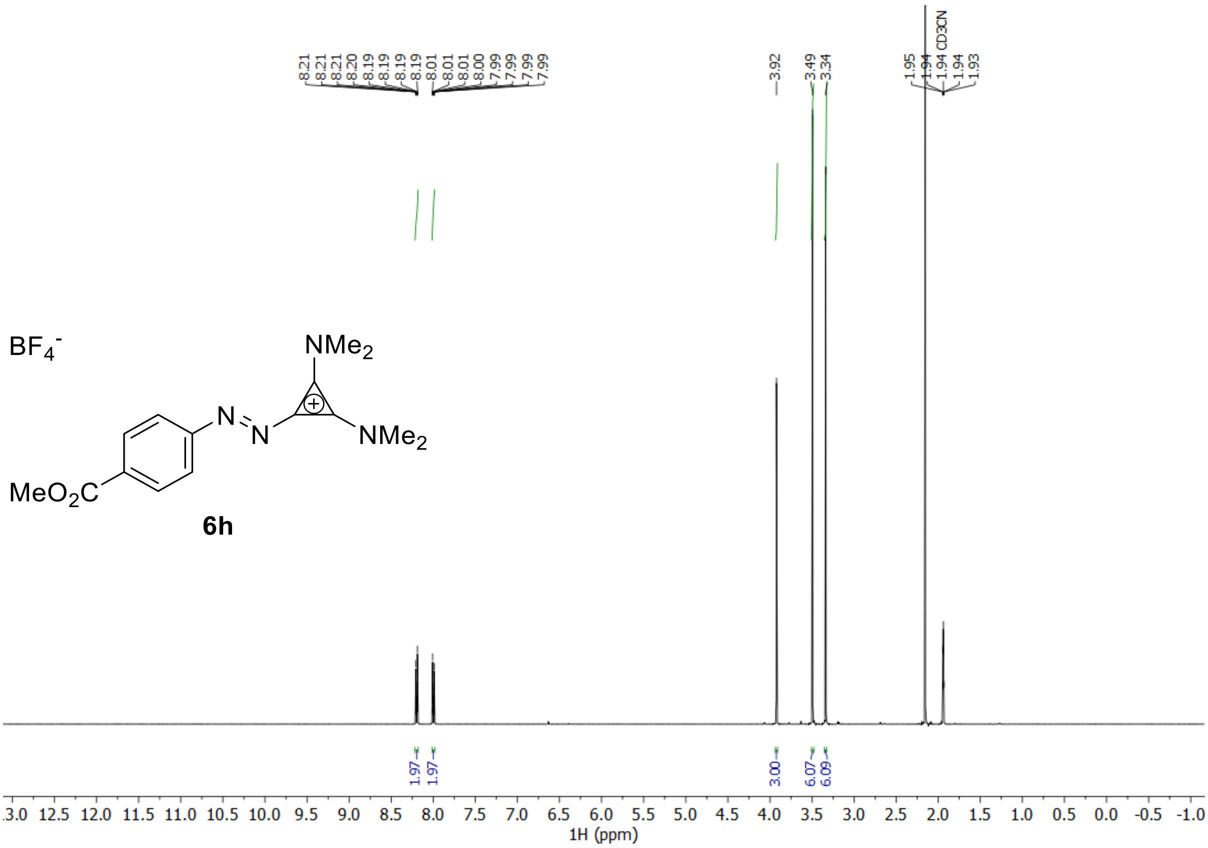

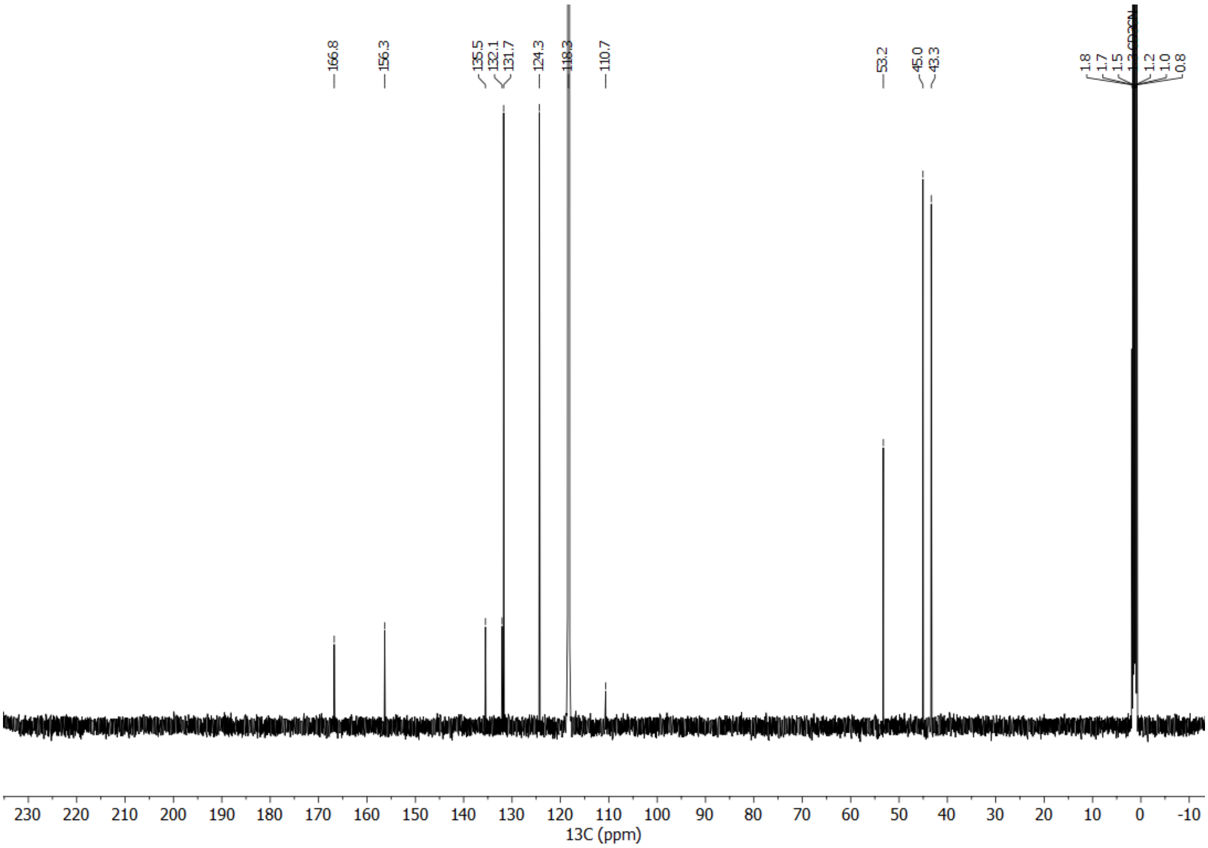

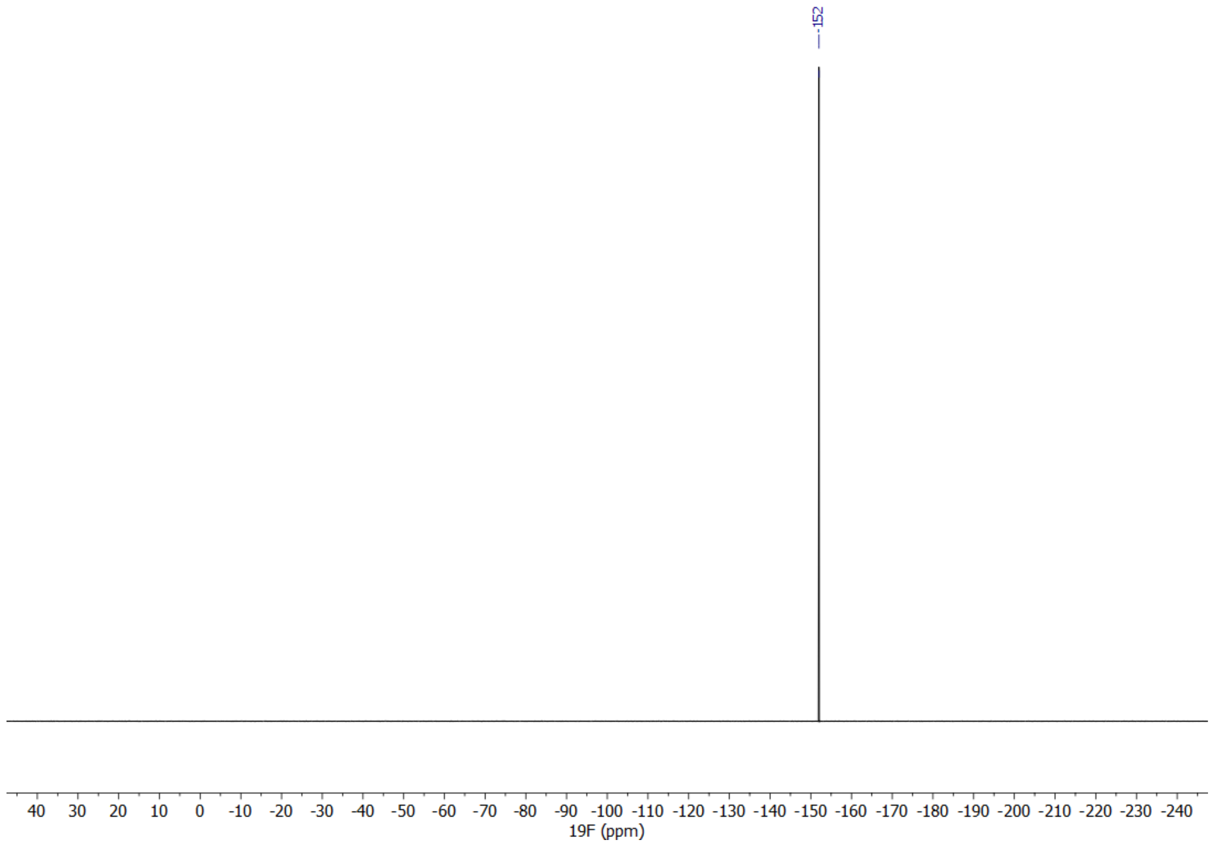
-152
40 30 20 10 0 -10 -20 -30 -40 -50 -60 -70 -80 -90 -100 -110 -120 -130 -140 -150 -160 -170 -180 -190 -200 -210 -220 -230 -240
19F (ppm)

^{1}H NMR, ^{13}C NMR, and ^{19}F NMR spectrum of compound **6i**

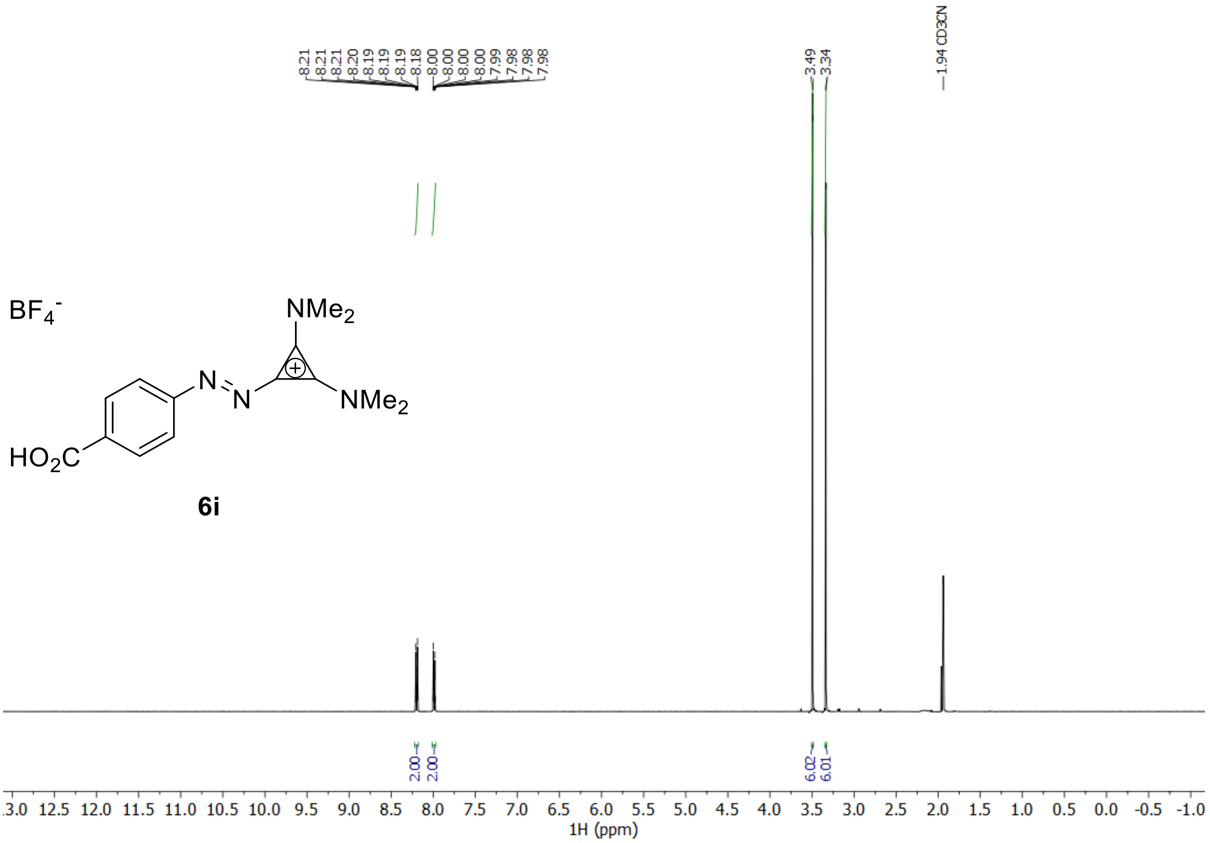

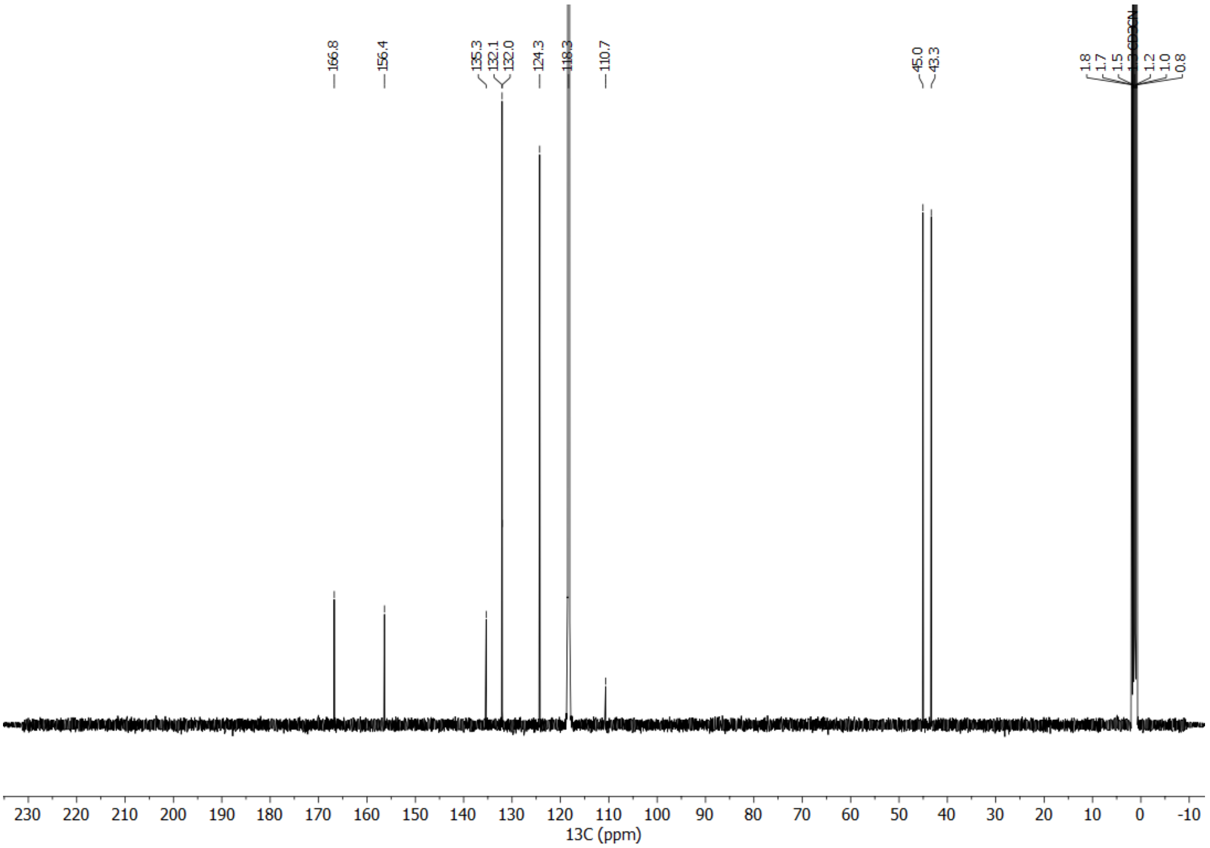

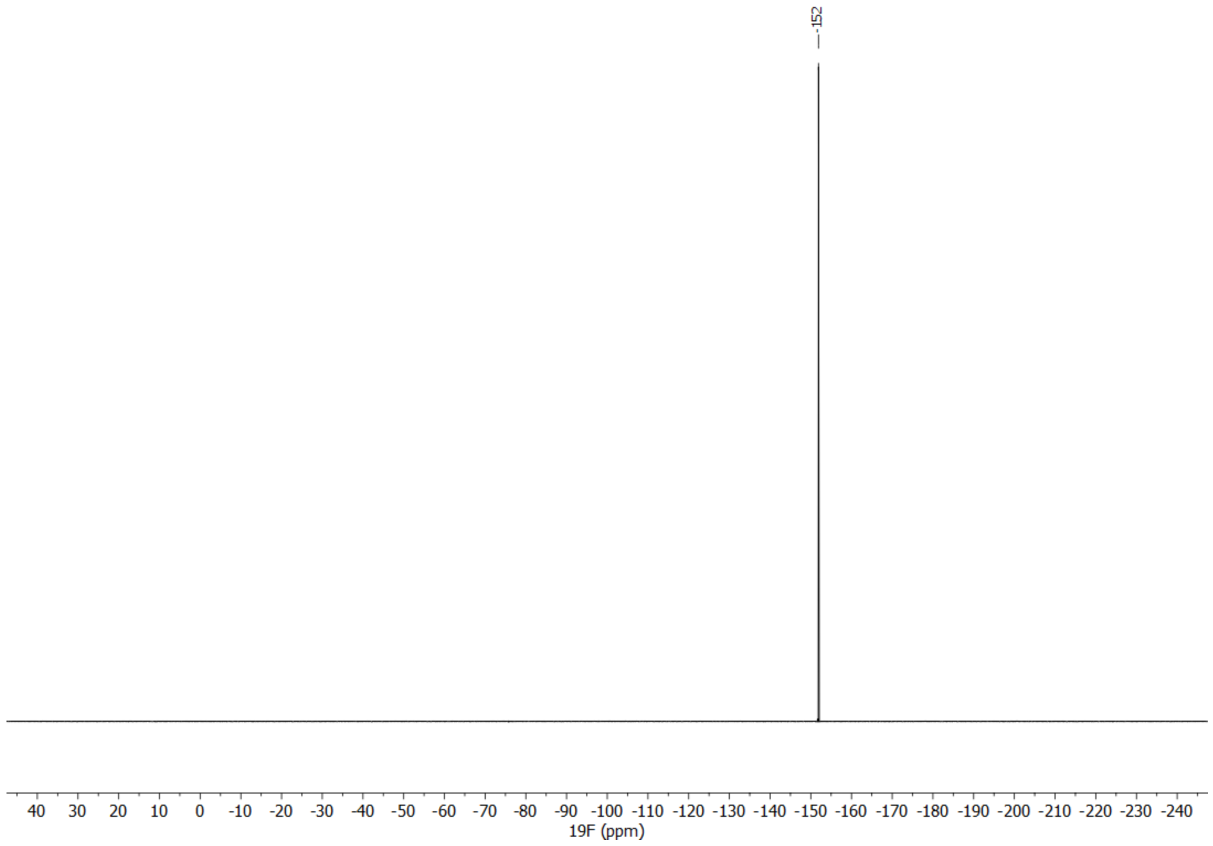
-152
40 30 20 10 0 -10 -20 -30 -40 -50 -60 -70 -80 -90 -100 -110 -120 -130 -140 -150 -160 -170 -180 -190 -200 -210 -220 -230 -240
19F (ppm)

^{1}H NMR, ^{13}C NMR, and ^{19}F NMR spectrum of compound **6j**

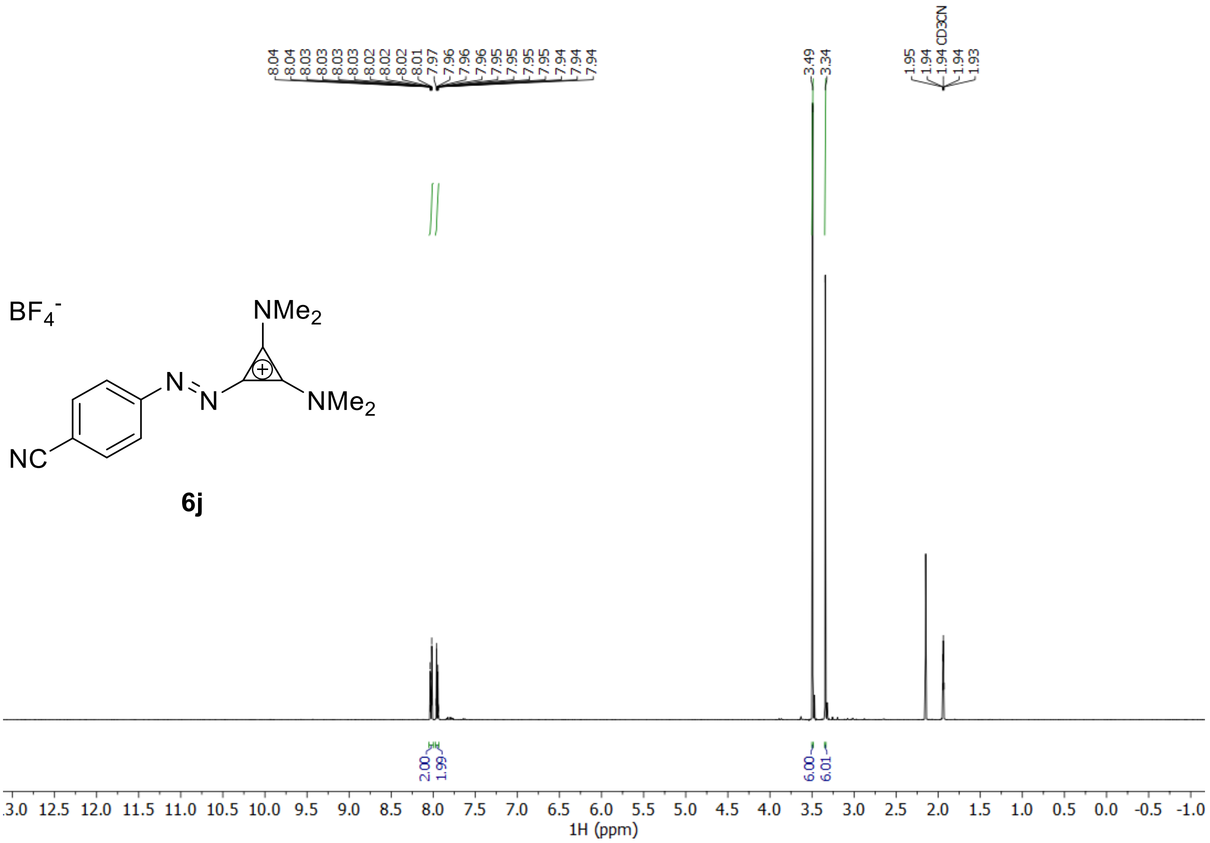

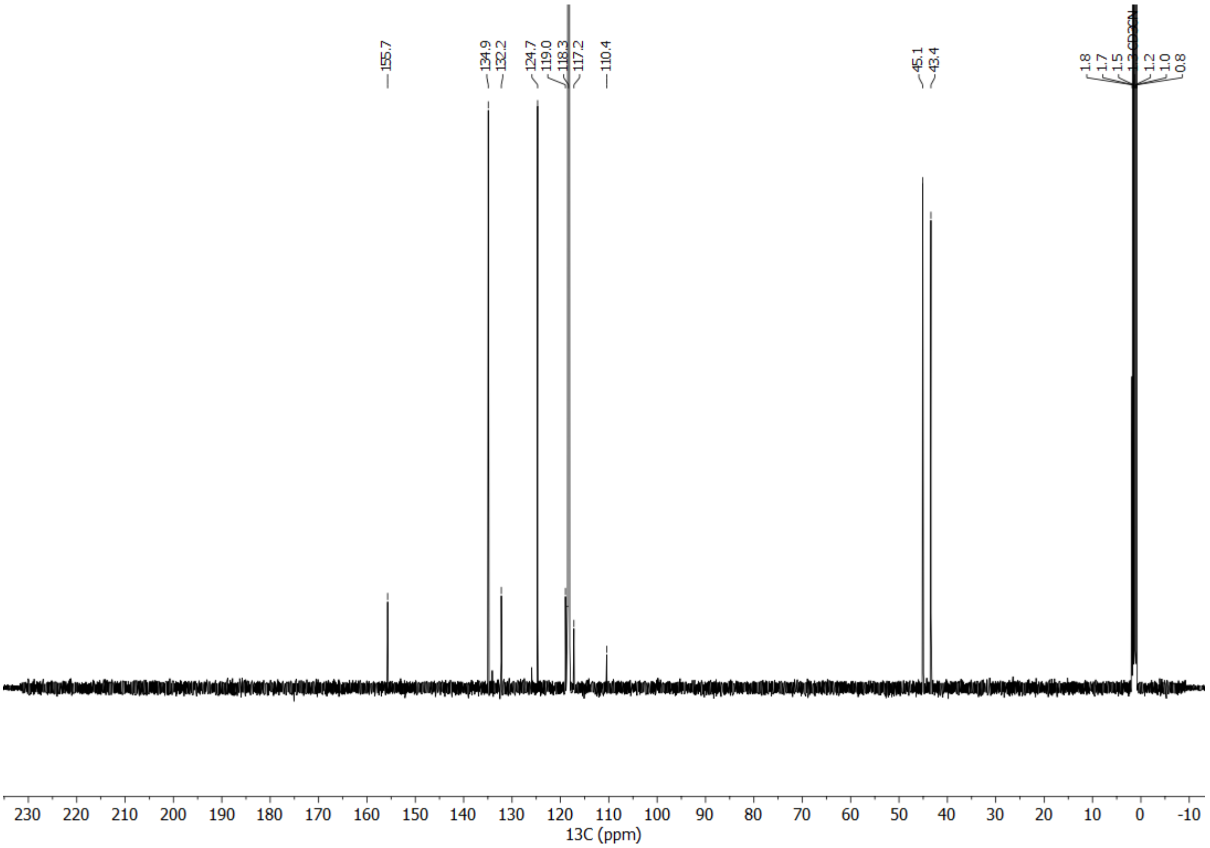

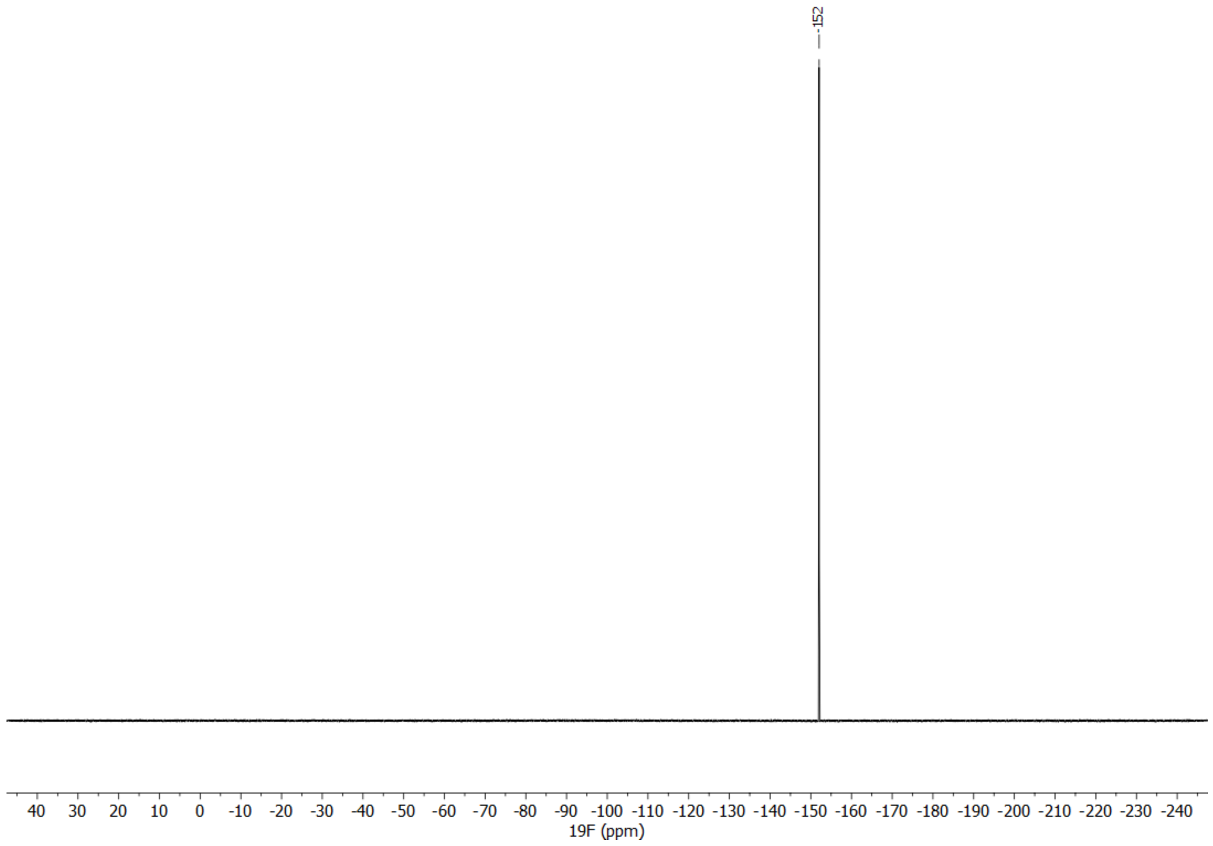
-152
40 30 20 10 0 -10 -20 -30 -40 -50 -60 -70 -80 -90 -100 -110 -120 -130 -140 -150 -160 -170 -180 -190 -200 -210 -220 -230 -240
19F (ppm)

^{1}H NMR, ^{13}C NMR, and ^{19}F NMR spectrum of compound **6k**

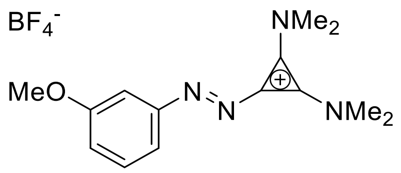

6k

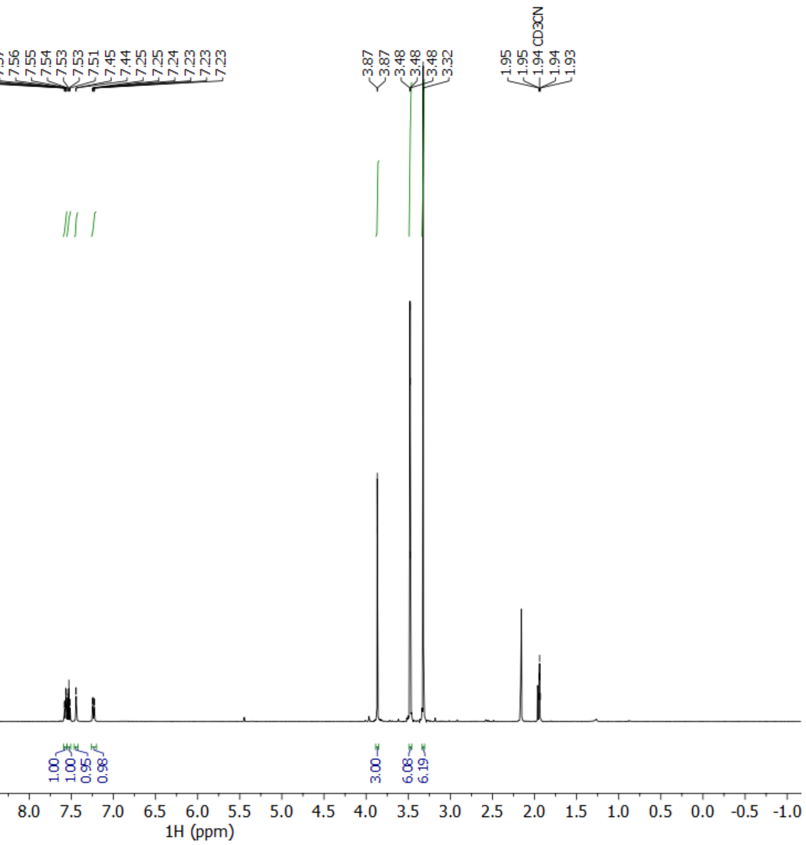

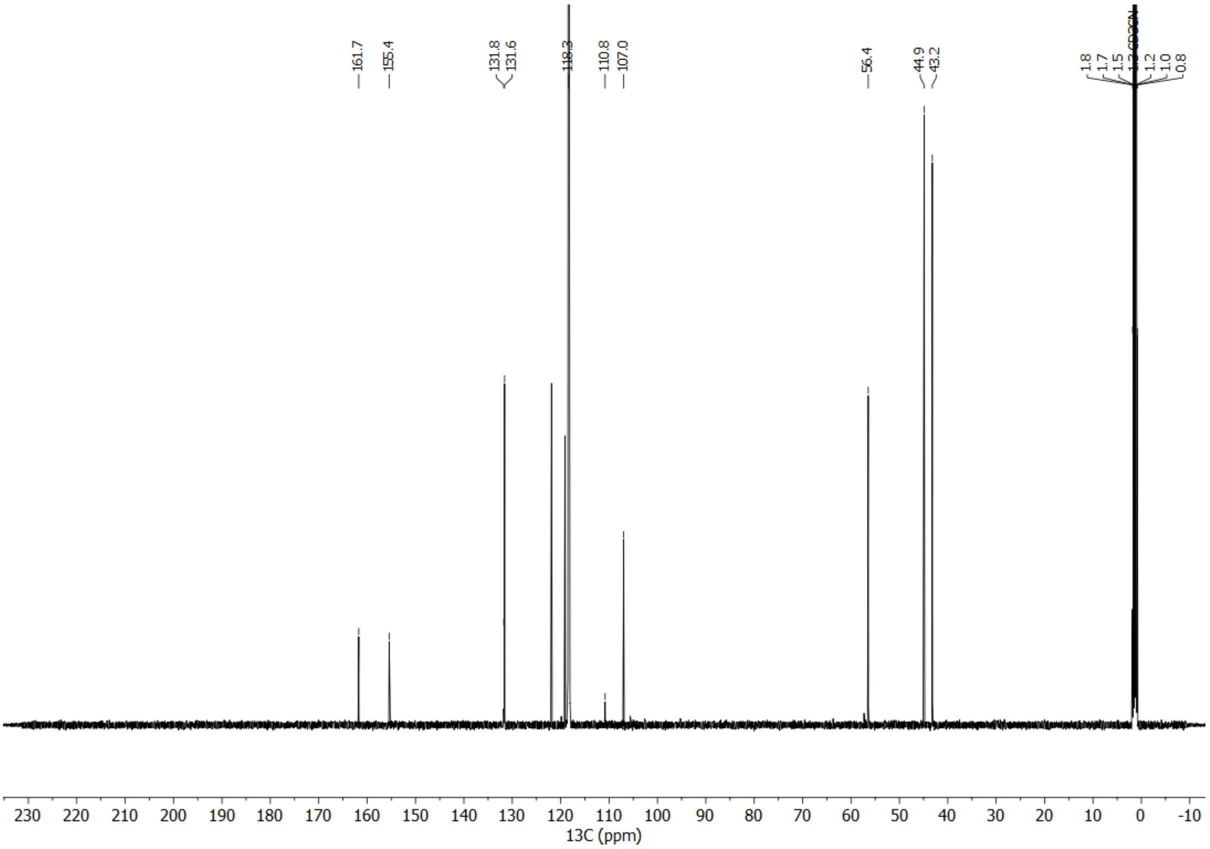

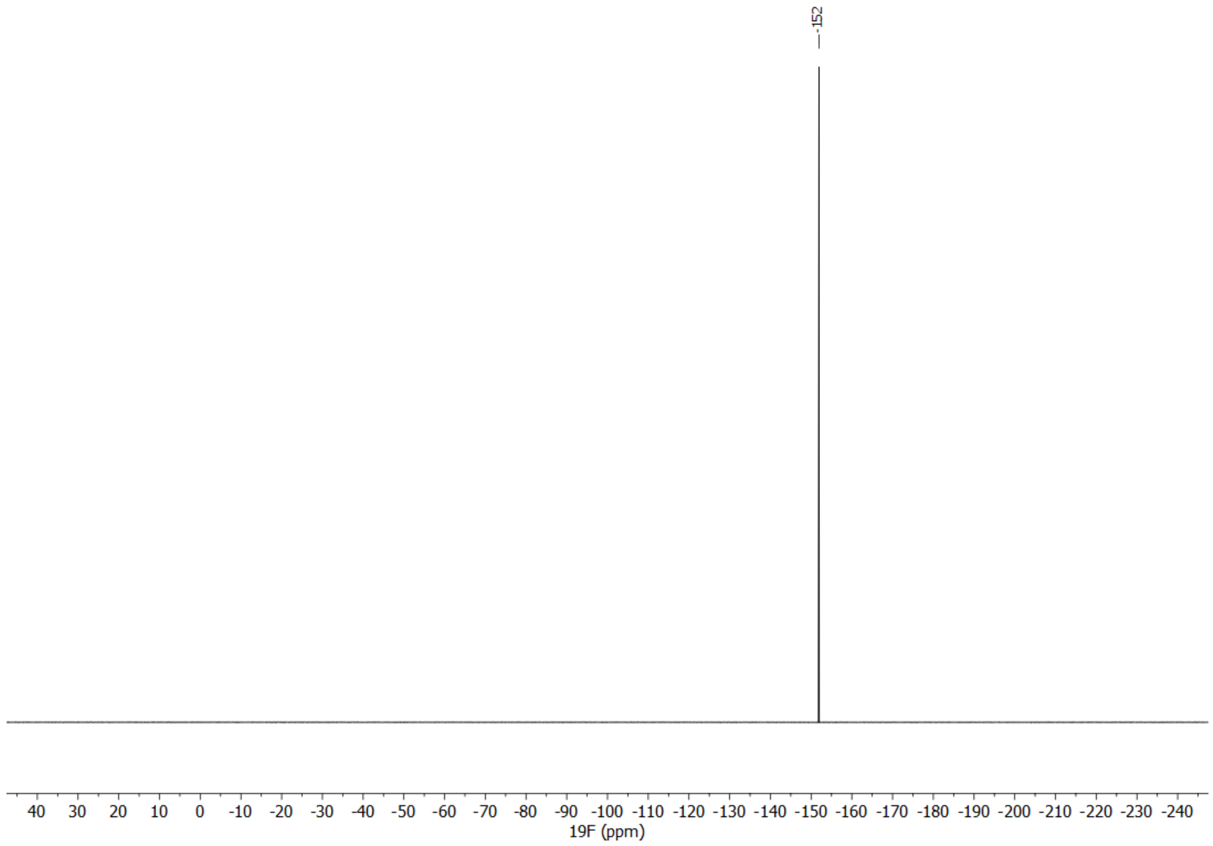
-152
40 30 20 10 0 -10 -20 -30 -40 -50 -60 -70 -80 -90 -100 -110 -120 -130 -140 -150 -160 -170 -180 -190 -200 -210 -220 -230 -240
19F (ppm)

^{1}H NMR, ^{13}C NMR, and ^{19}F NMR spectrum of compound **6l**

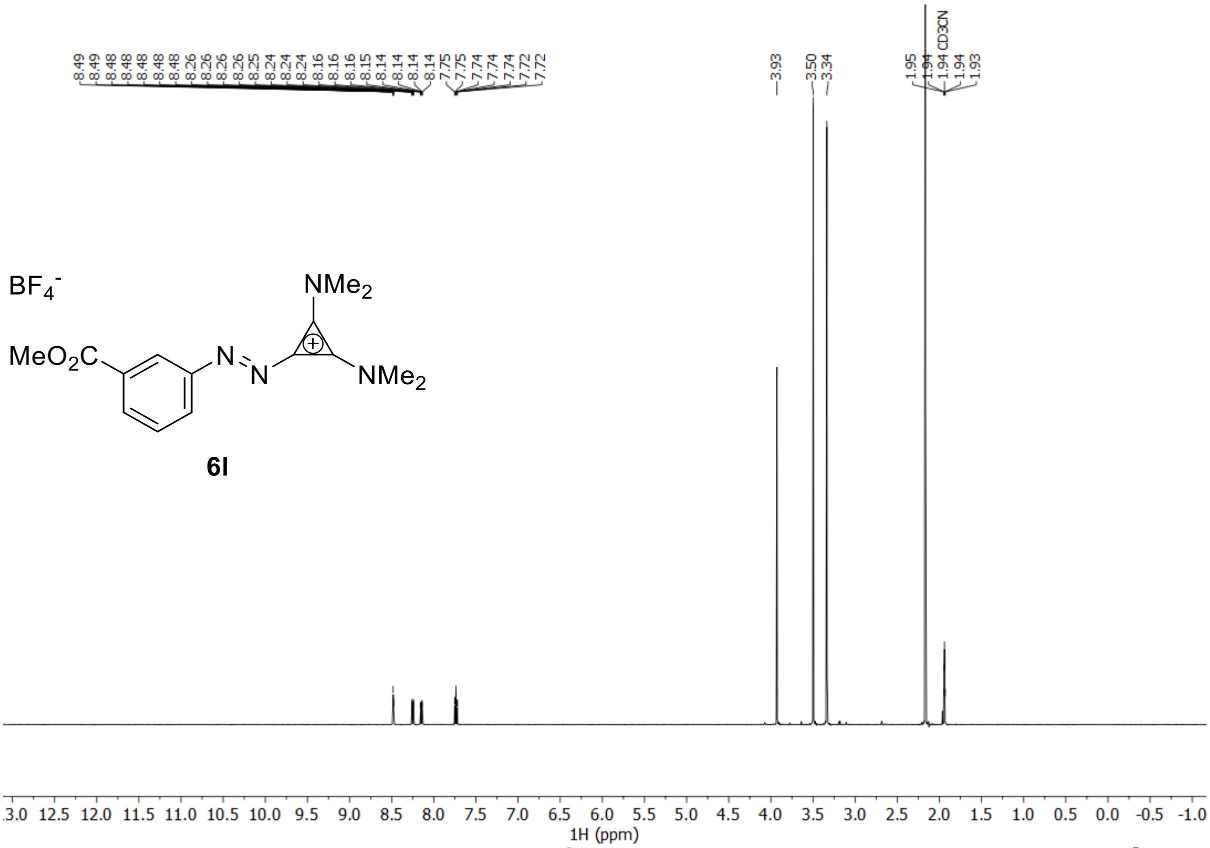

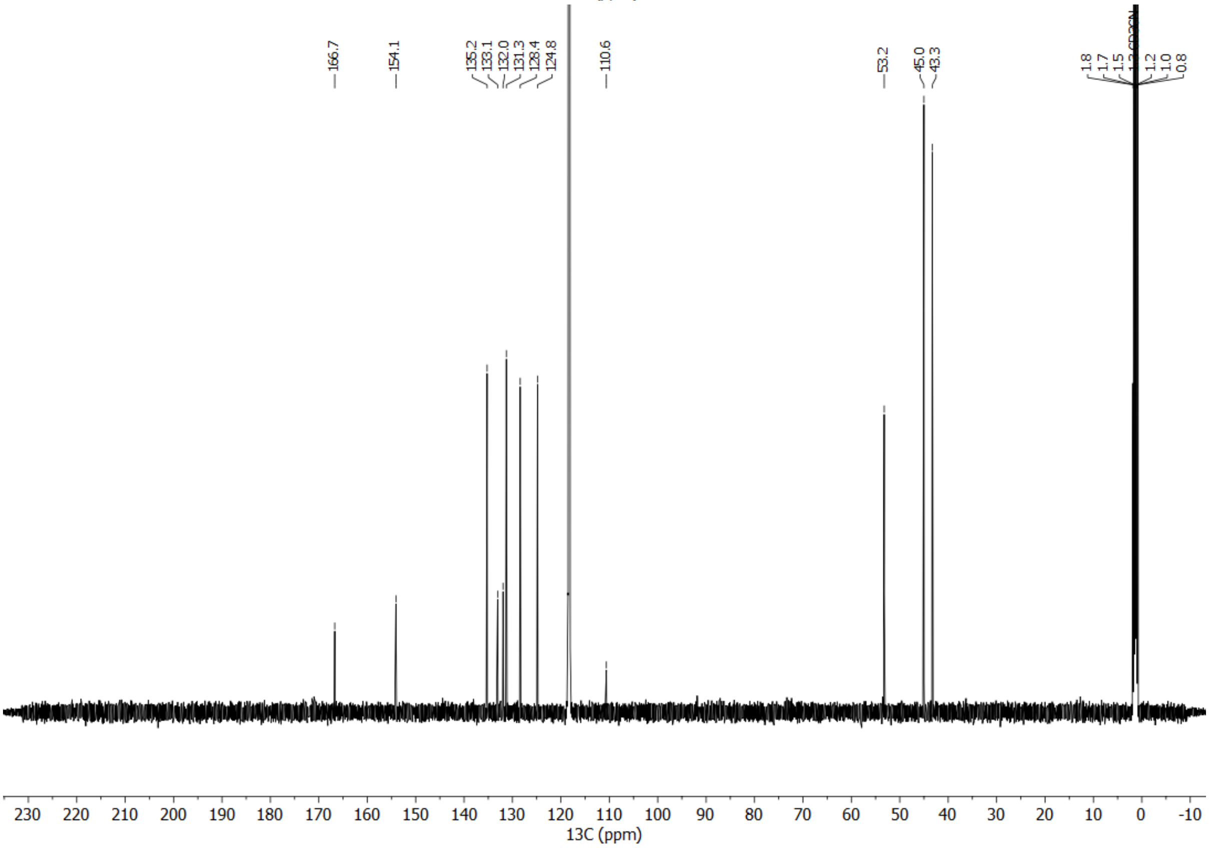

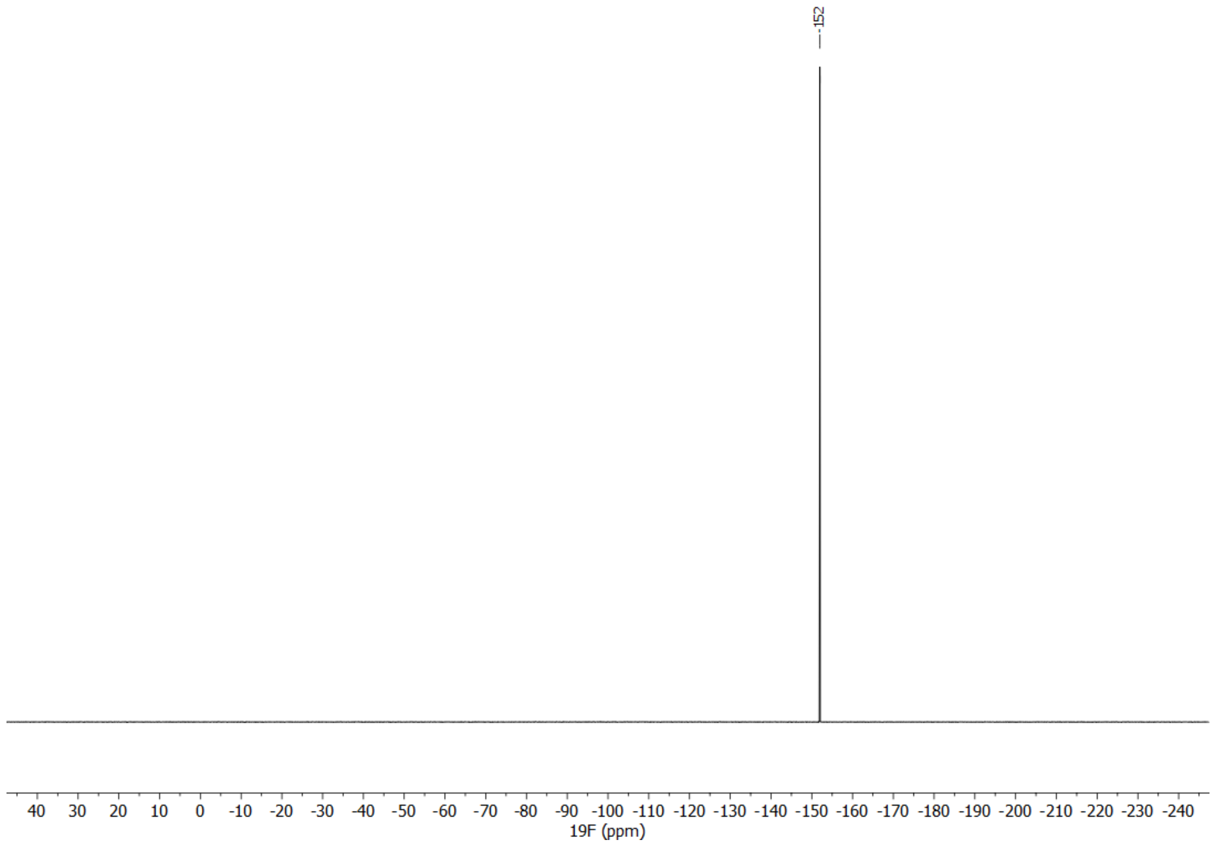
-152
40 30 20 10 0 -10 -20 -30 -40 -50 -60 -70 -80 -90 -100 -110 -120 -130 -140 -150 -160 -170 -180 -190 -200 -210 -220 -230 -240
19F (ppm)

^{1}H NMR, ^{13}C NMR, and ^{19}F NMR spectrum of compound **6m**

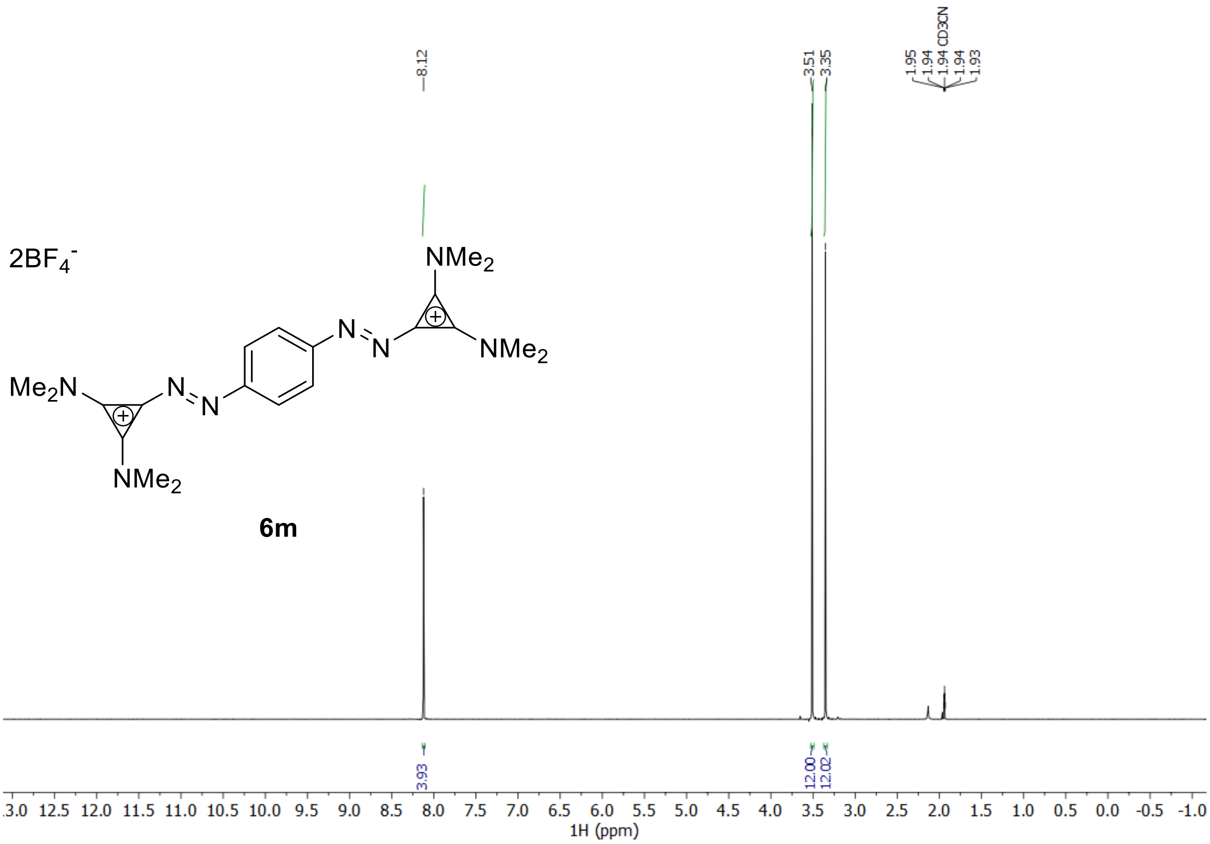

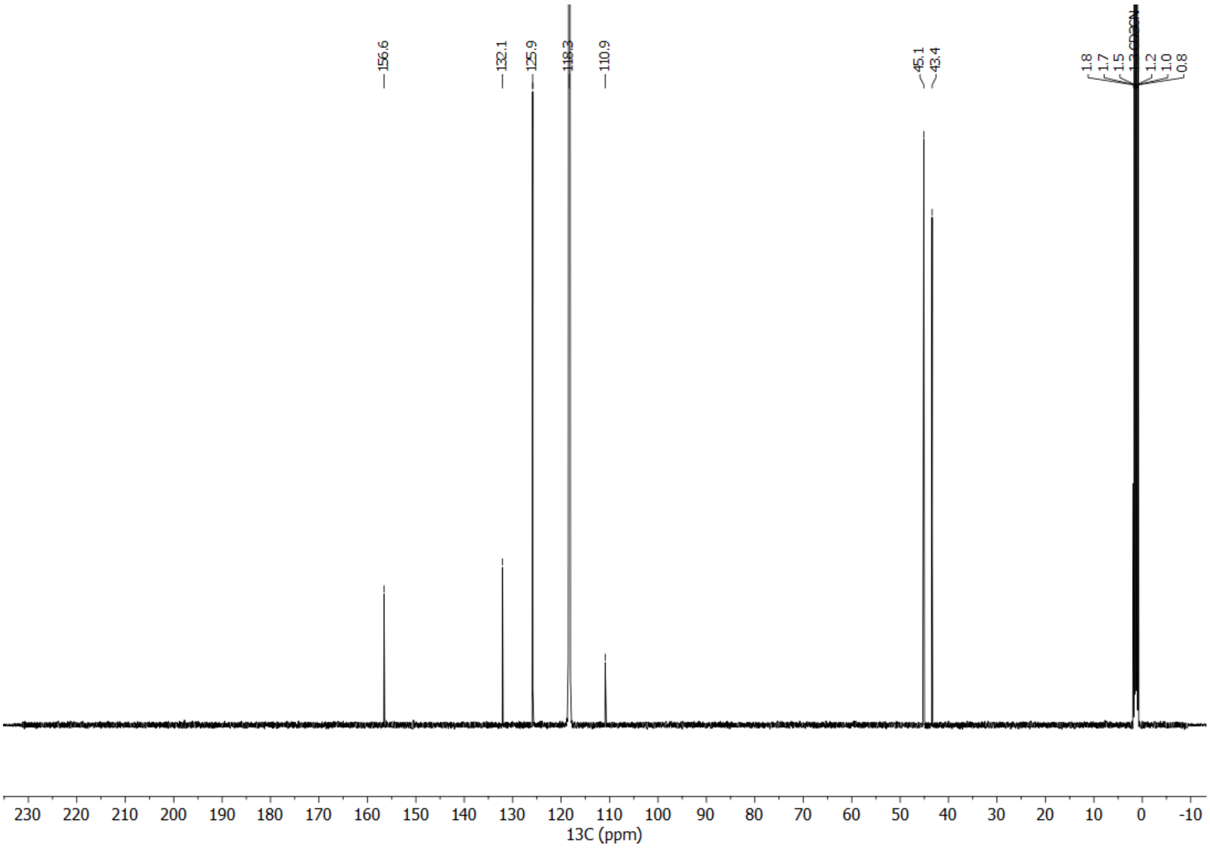

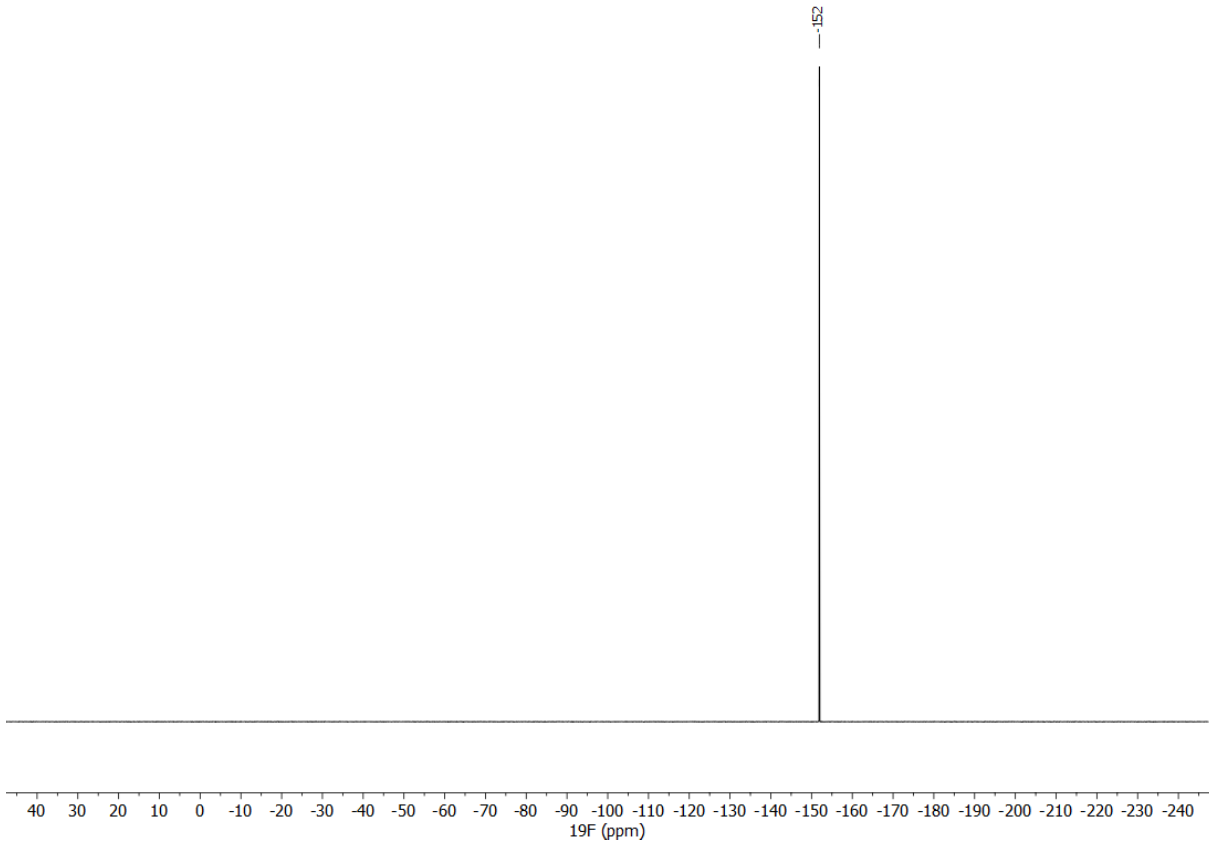
-152
40 30 20 10 0 -10 -20 -30 -40 -50 -60 -70 -80 -90 -100 -110 -120 -130 -140 -150 -160 -170 -180 -190 -200 -210 -220 -230 -240
19F (ppm)

^{1}H NMR spectrum of compound **6n** and **6n'** (2:1) after flash column chromatography

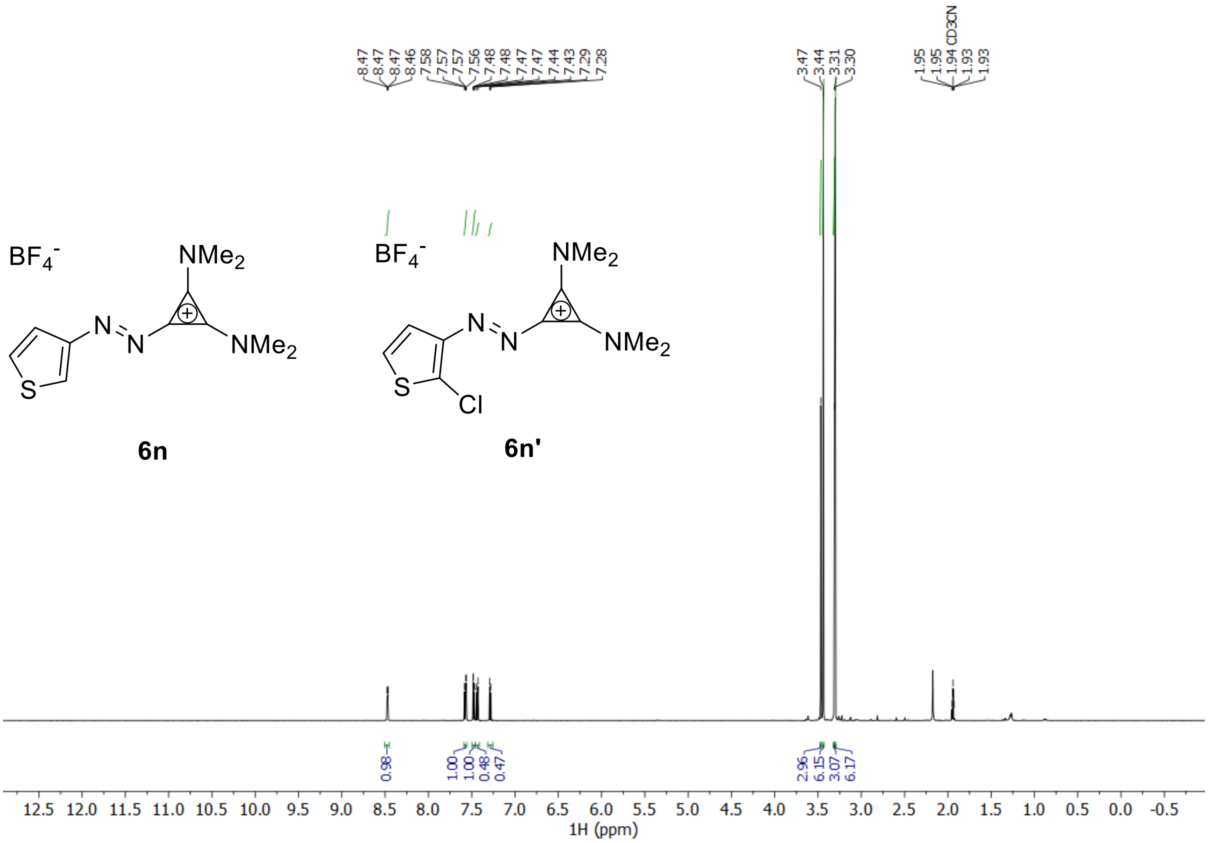

^{1}H NMR, ^{13}C NMR, and ^{19}F NMR spectrum of compound **6n**

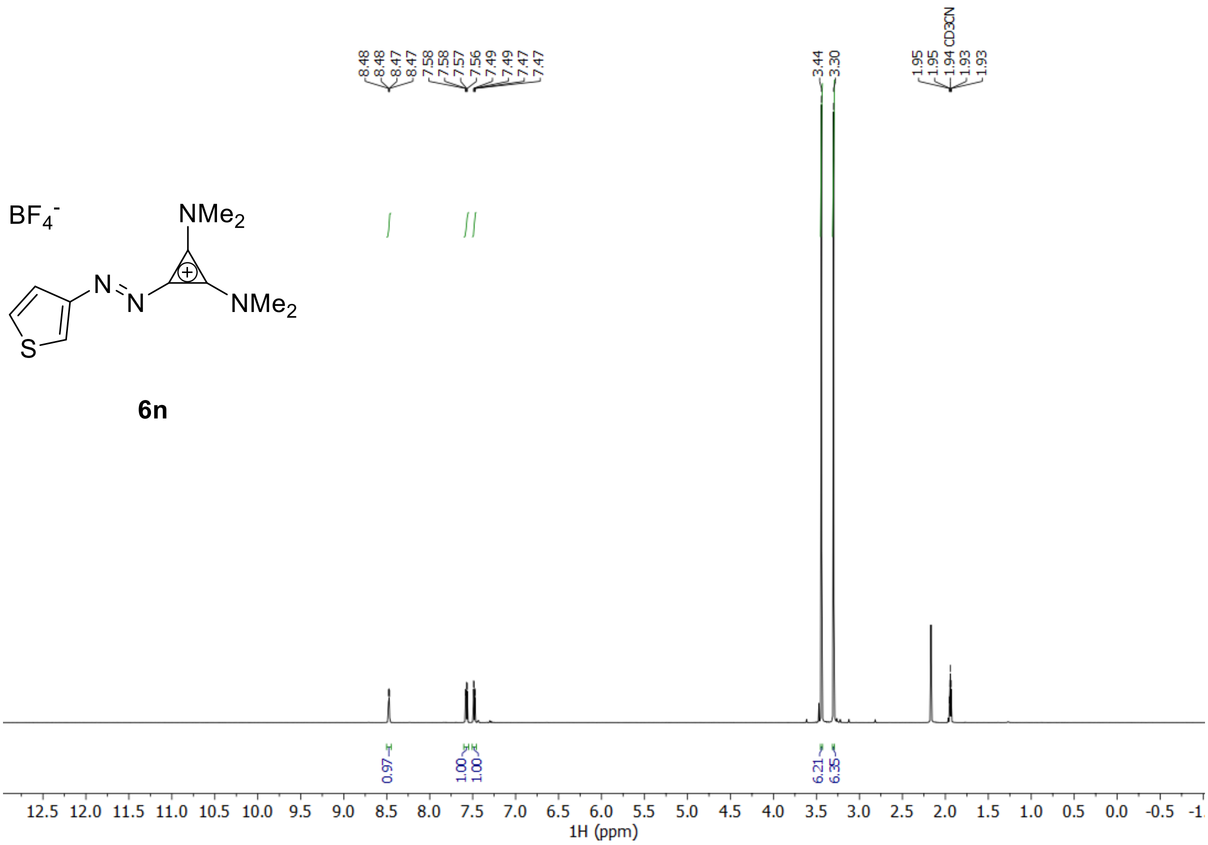

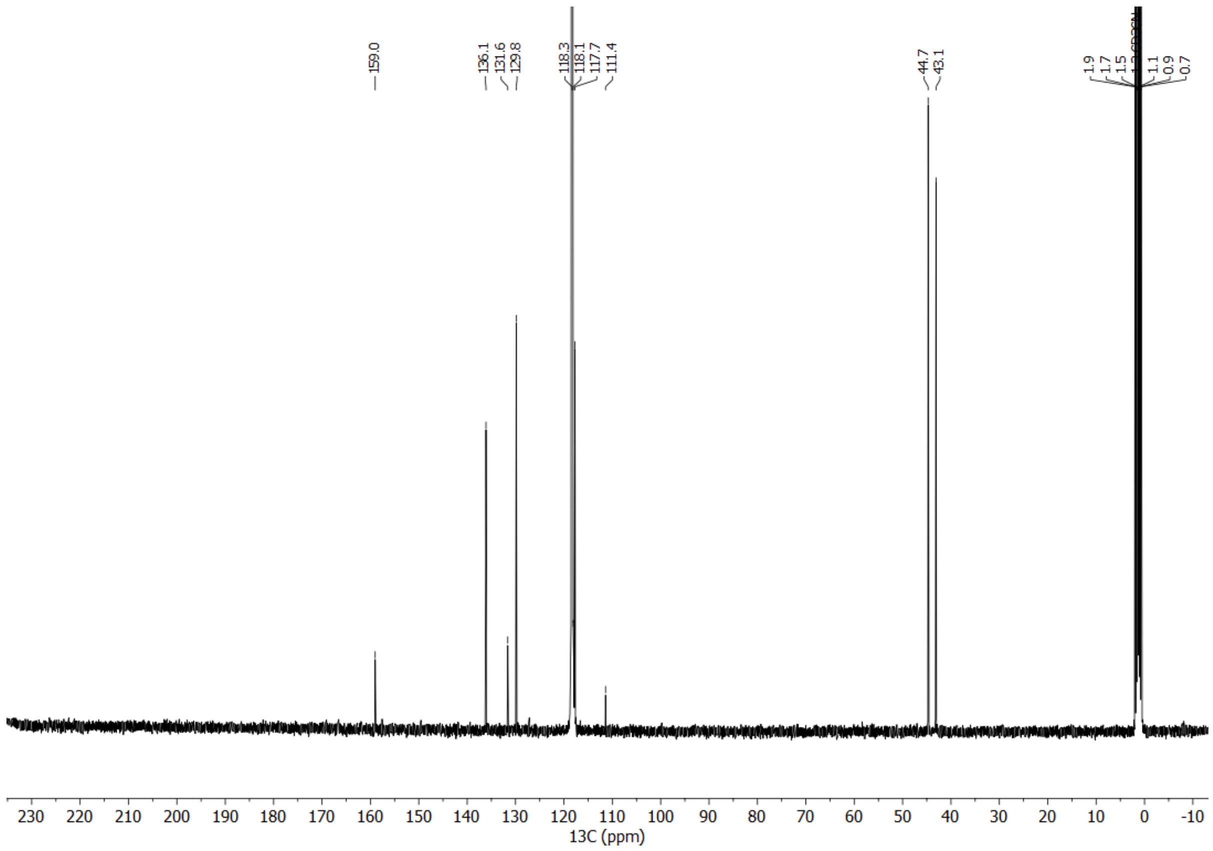
159.0
136.1
131.6
129.8
118.3
118.1
117.7
111.4
44.7
43.1
1.9
1.7
1.5
1.1
0.9
0.7
230 220 210 200 190 180 170 160 150 140 130 120 110 100 90 80 70 60 50 40 30 20 10 0 -10
13C (ppm)

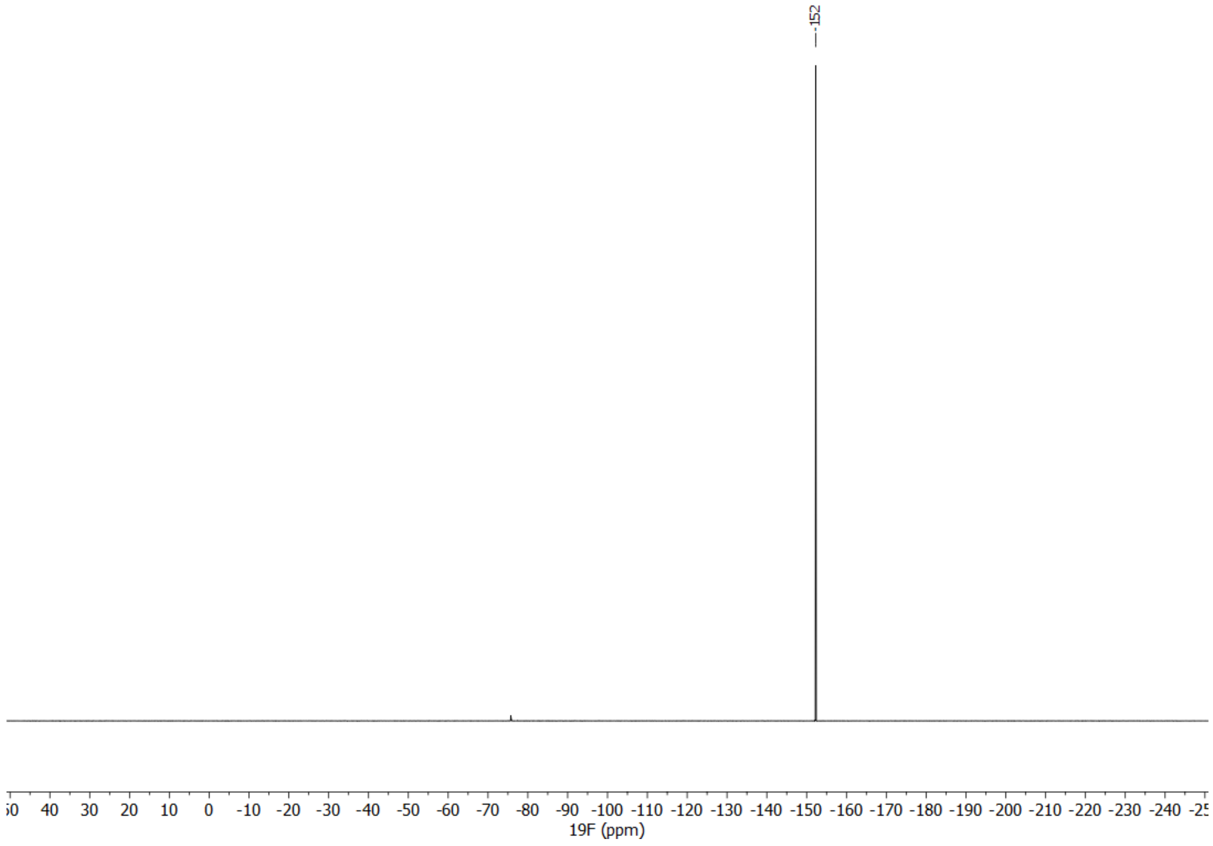
-152
40 30 20 10 0 -10 -20 -30 -40 -50 -60 -70 -80 -90 -100 -110 -120 -130 -140 -150 -160 -170 -180 -190 -200 -210 -220 -230 -240
19F (ppm)

^{1}H NMR, ^{13}C NMR, and ^{19}F NMR spectrum of compounds **6n** and **6n'** (1:2) after precipitation

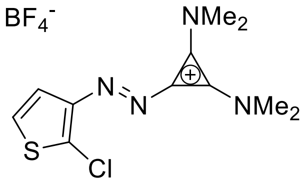

6n'

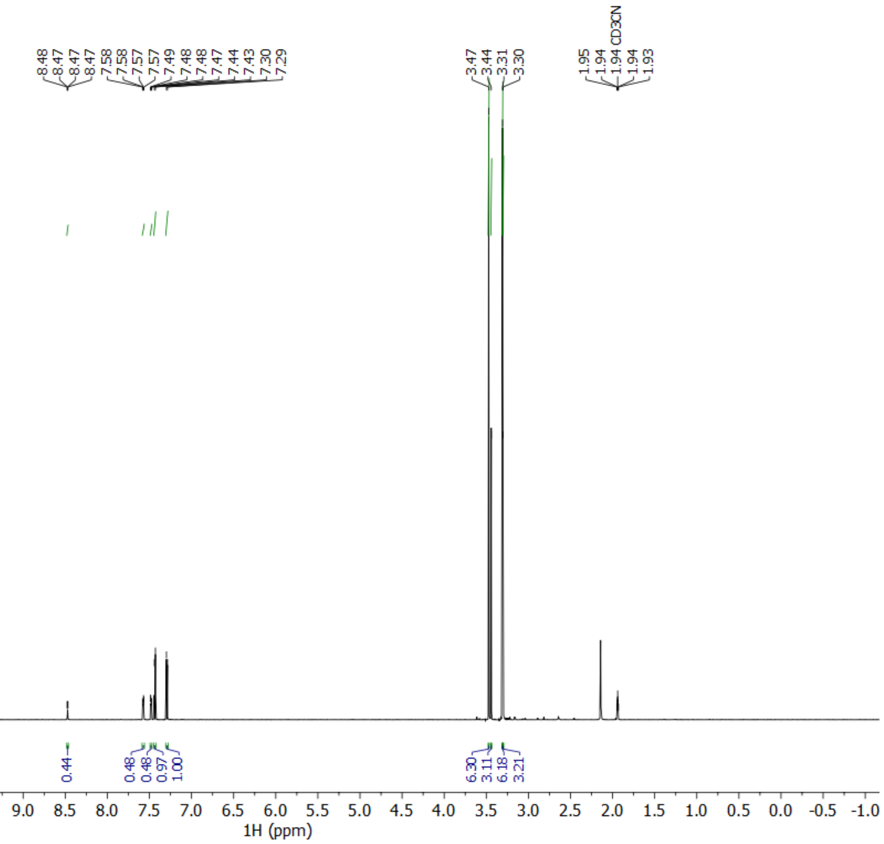

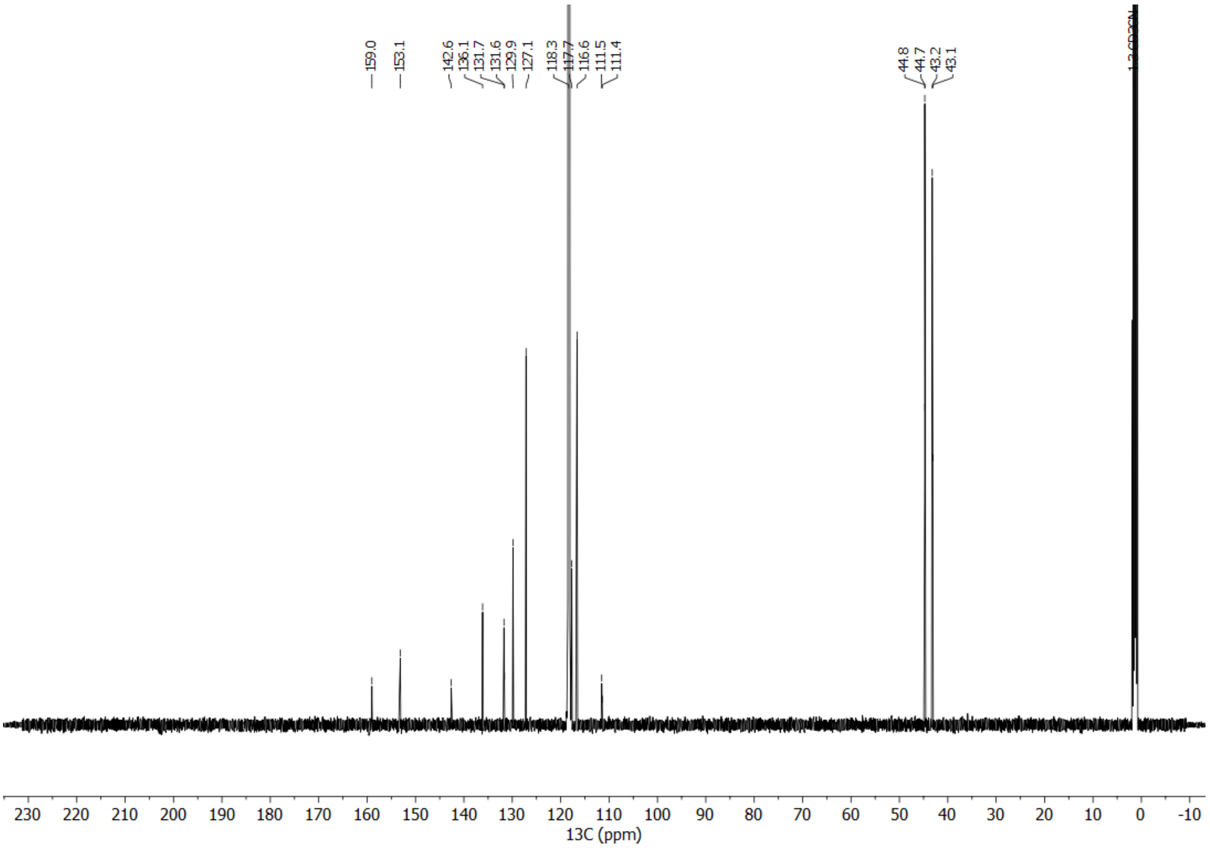

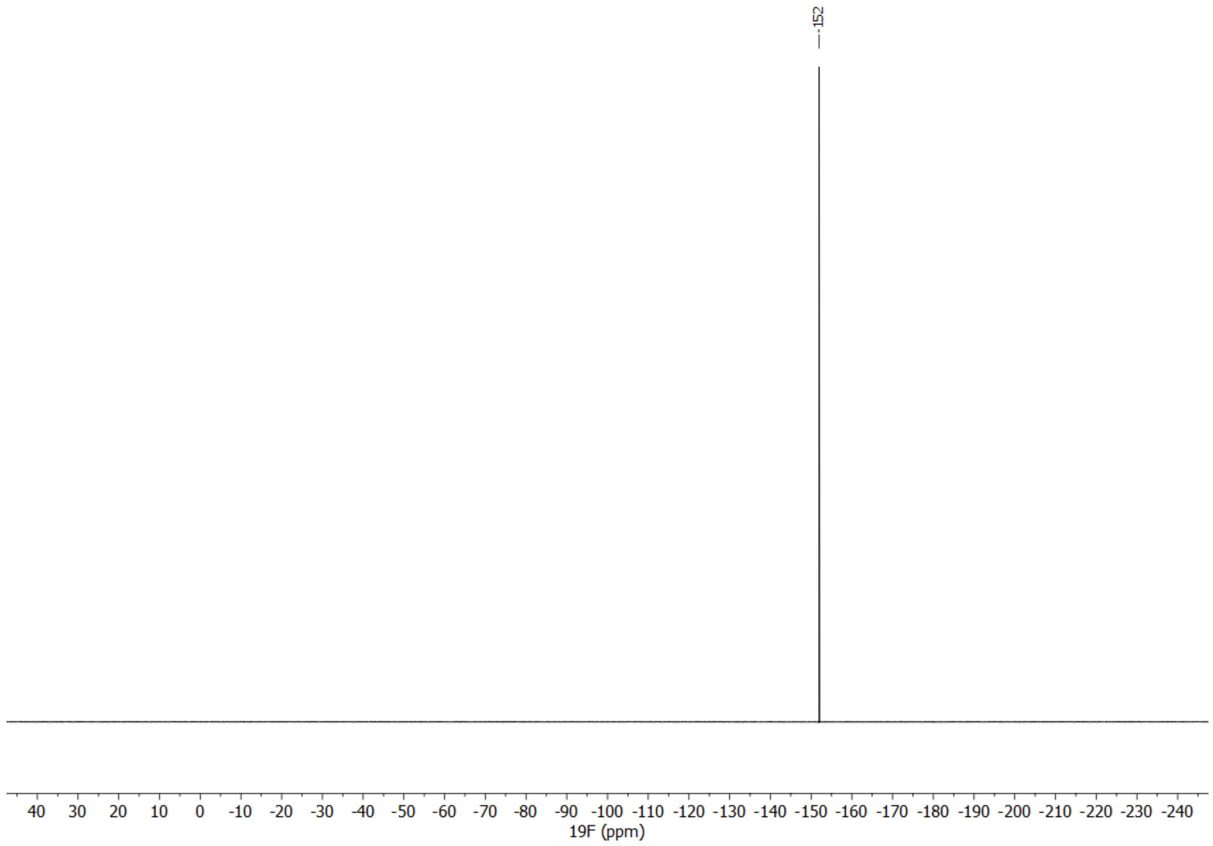
-152
40 30 20 10 0 -10 -20 -30 -40 -50 -60 -70 -80 -90 -100 -110 -120 -130 -140 -150 -160 -170 -180 -190 -200 -210 -220 -230 -240
19F (ppm)

^{1}H NMR, ^{13}C NMR, and ^{19}F NMR spectrum of compound **6o**

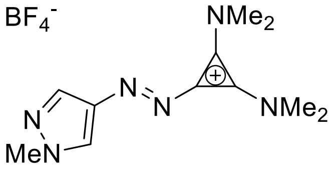

6o

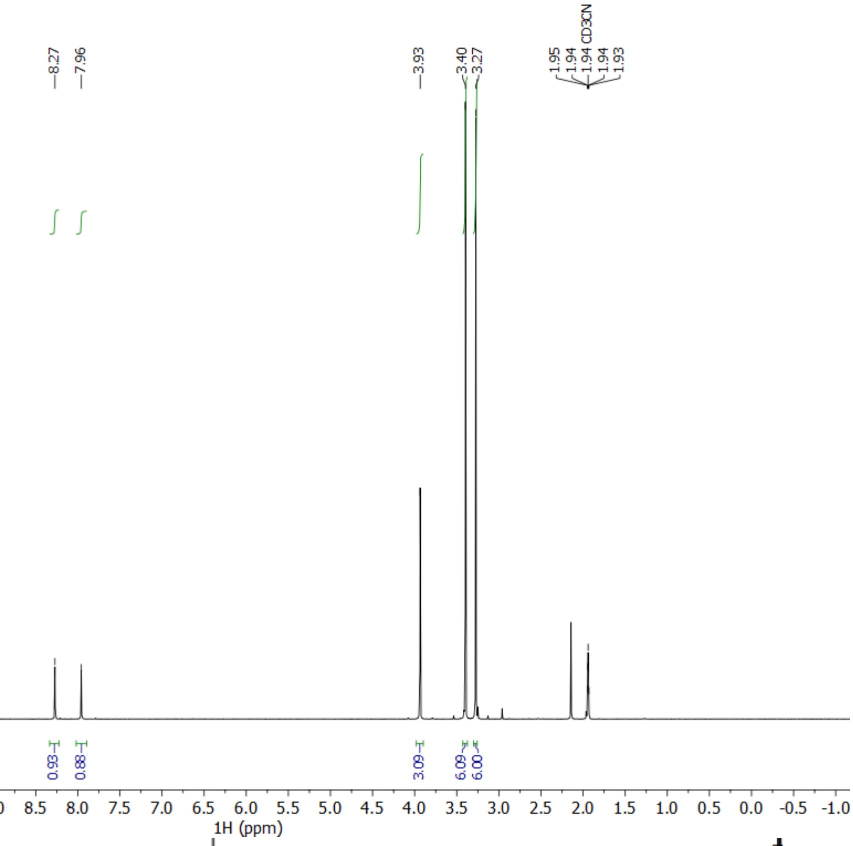

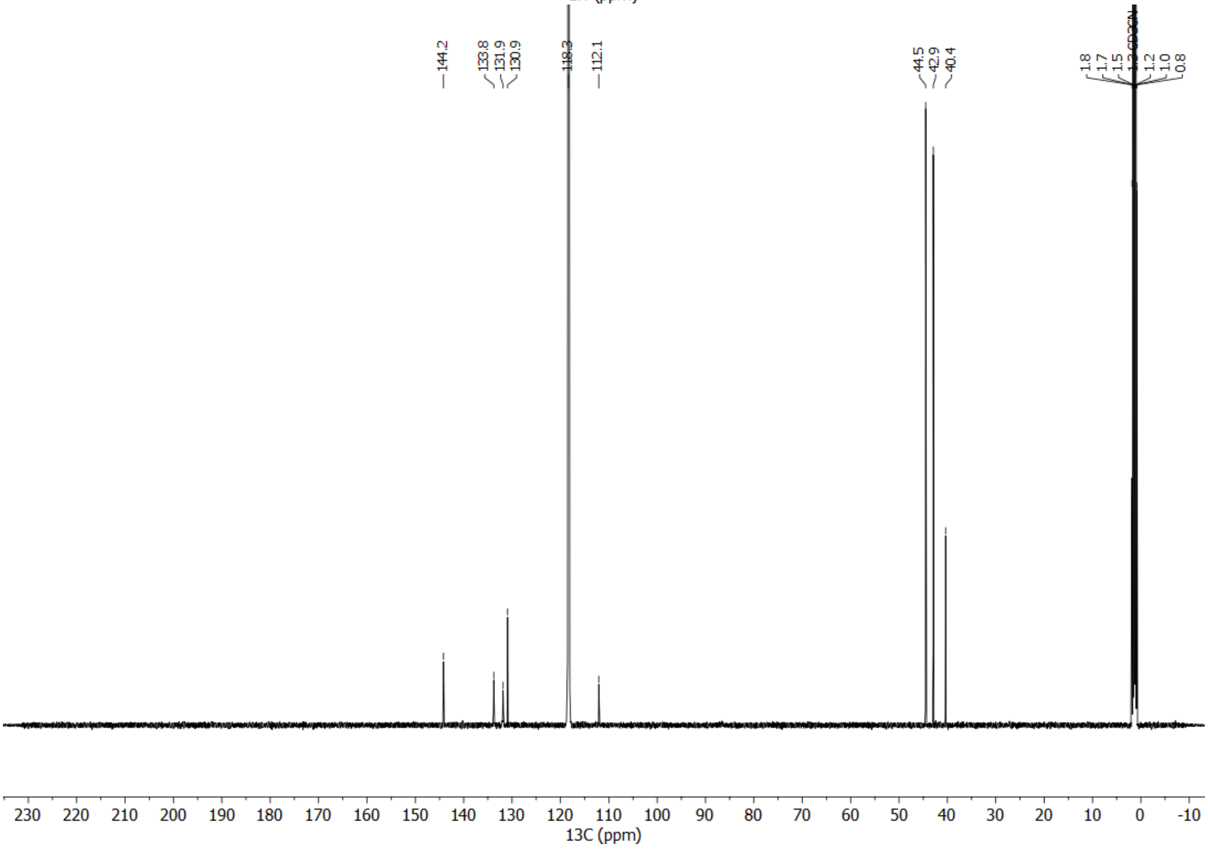

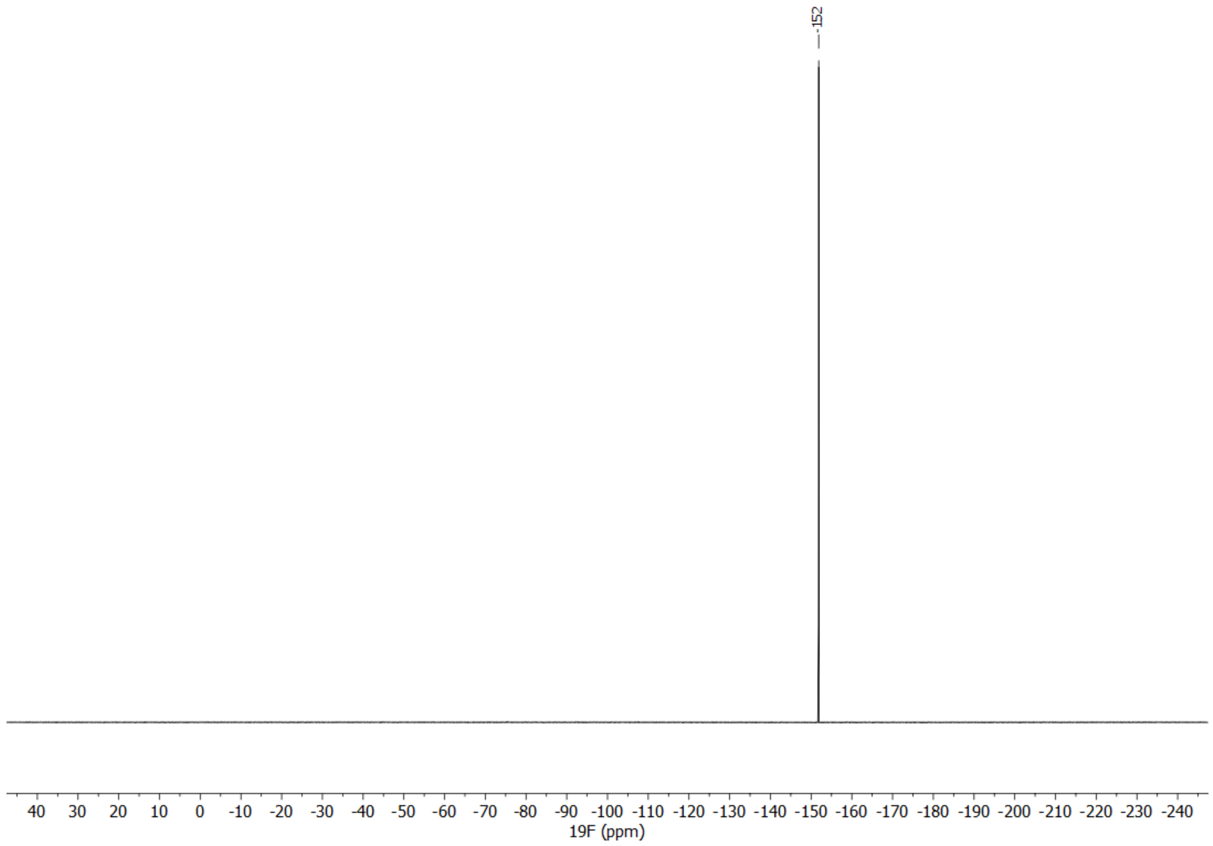
-152
40 30 20 10 0 -10 -20 -30 -40 -50 -60 -70 -80 -90 -100 -110 -120 -130 -140 -150 -160 -170 -180 -190 -200 -210 -220 -230 -240
19F (ppm)

^{1}H NMR, ^{13}C NMR, and ^{19}F NMR spectrum of compound **6p**

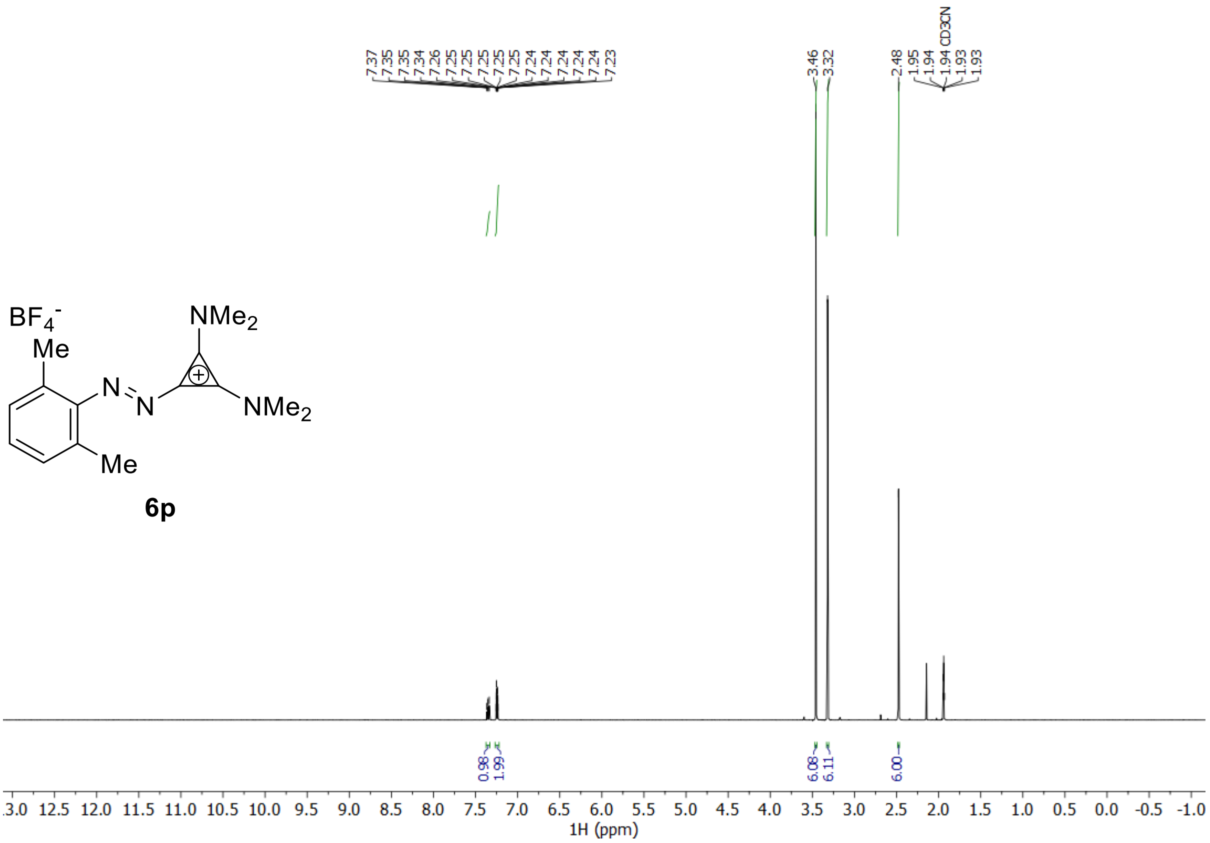

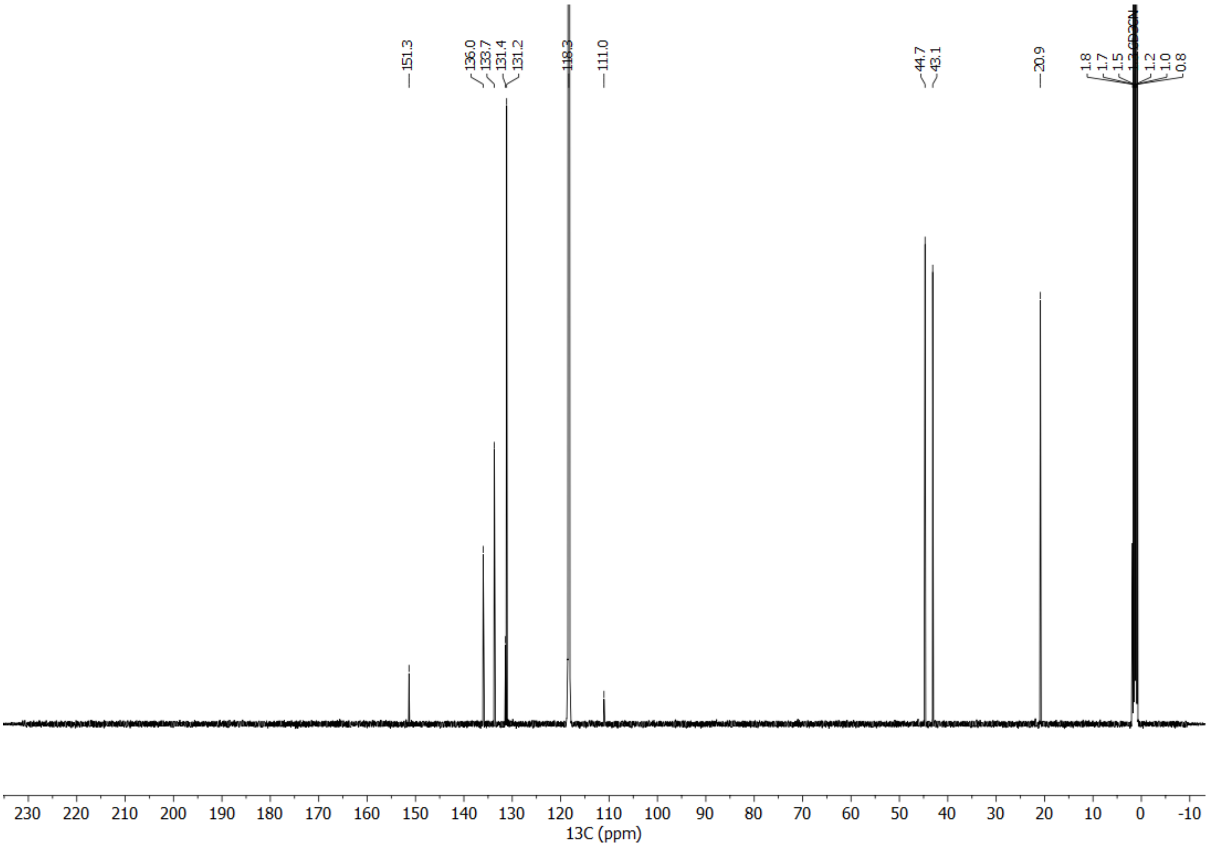

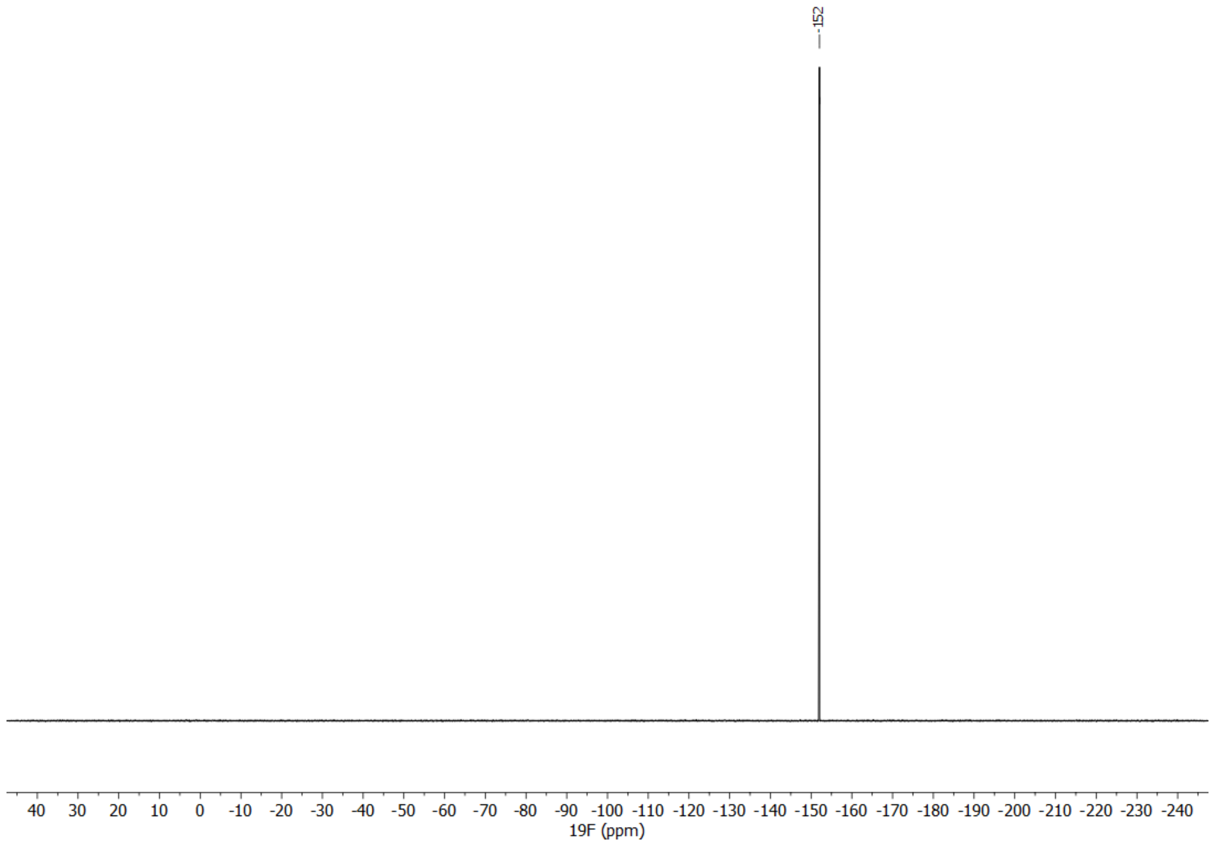
-152
40 30 20 10 0 -10 -20 -30 -40 -50 -60 -70 -80 -90 -100 -110 -120 -130 -140 -150 -160 -170 -180 -190 -200 -210 -220 -230 -240
19F (ppm)

^{1}H NMR, ^{13}C NMR, and ^{19}F NMR spectrum of compound **6q**

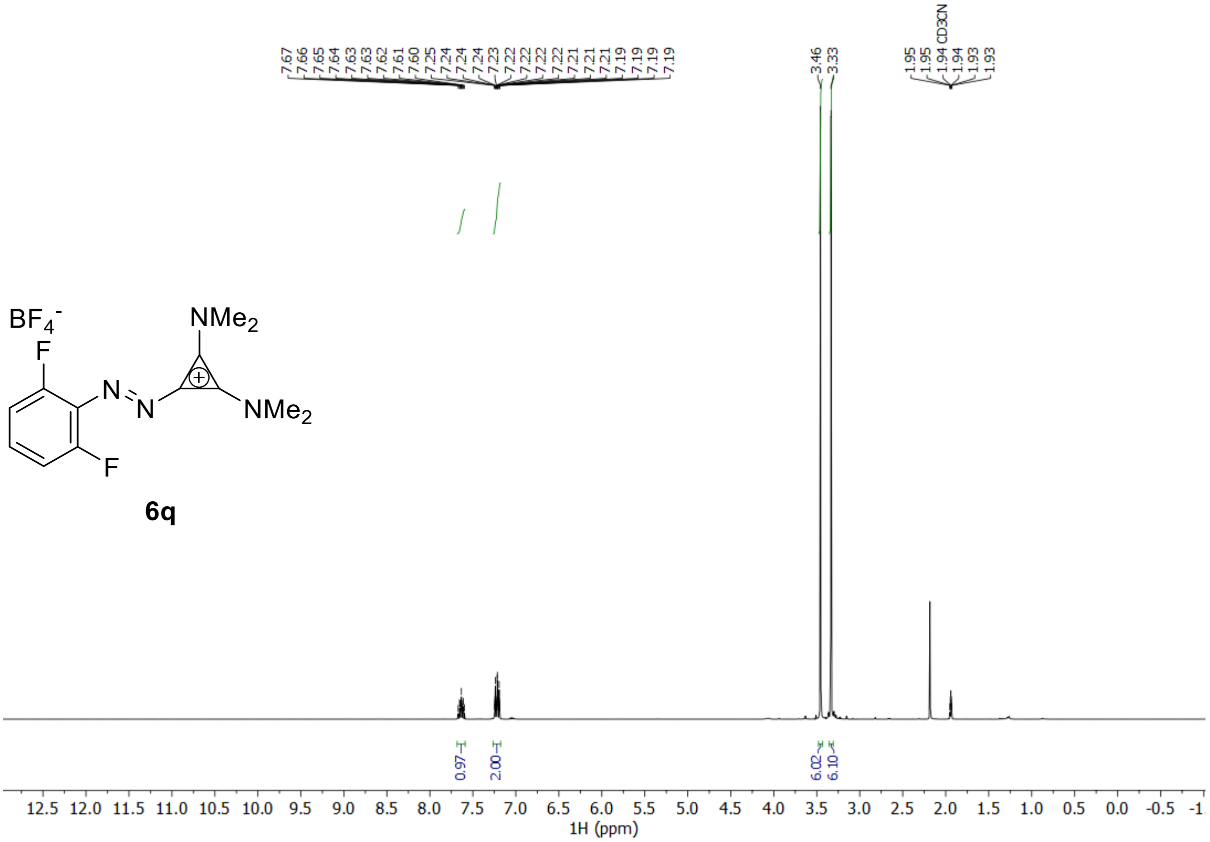

6q

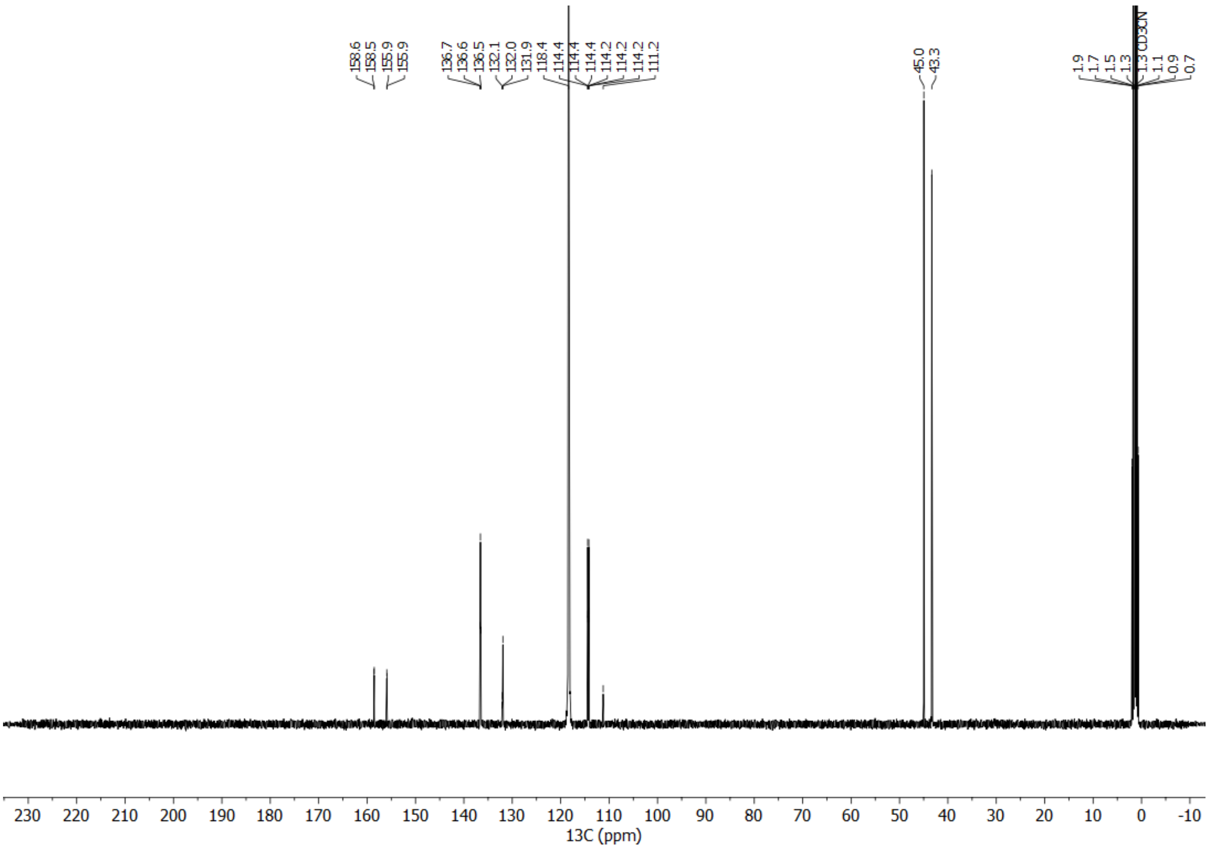

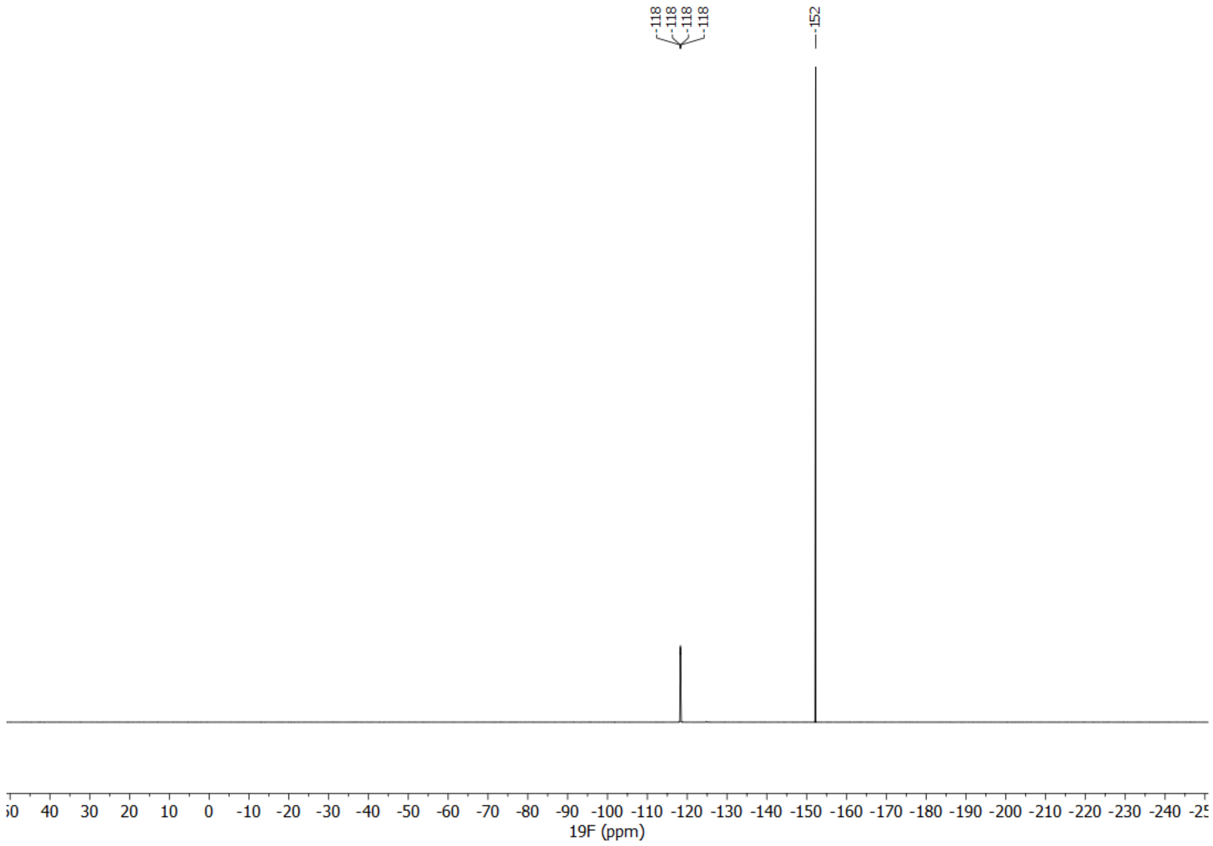

-118
-118
-118
-118
-152
50 40 30 20 10 0 -10 -20 -30 -40 -50 -60 -70 -80 -90 -100 -110 -120 -130 -140 -150 -160 -170 -180 -190 -200 -210 -220 -230 -240 -250
19F (ppm)

^{1}H NMR, ^{13}C NMR, and ^{19}F NMR spectrum of compound **6r**

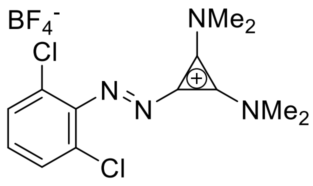

6r

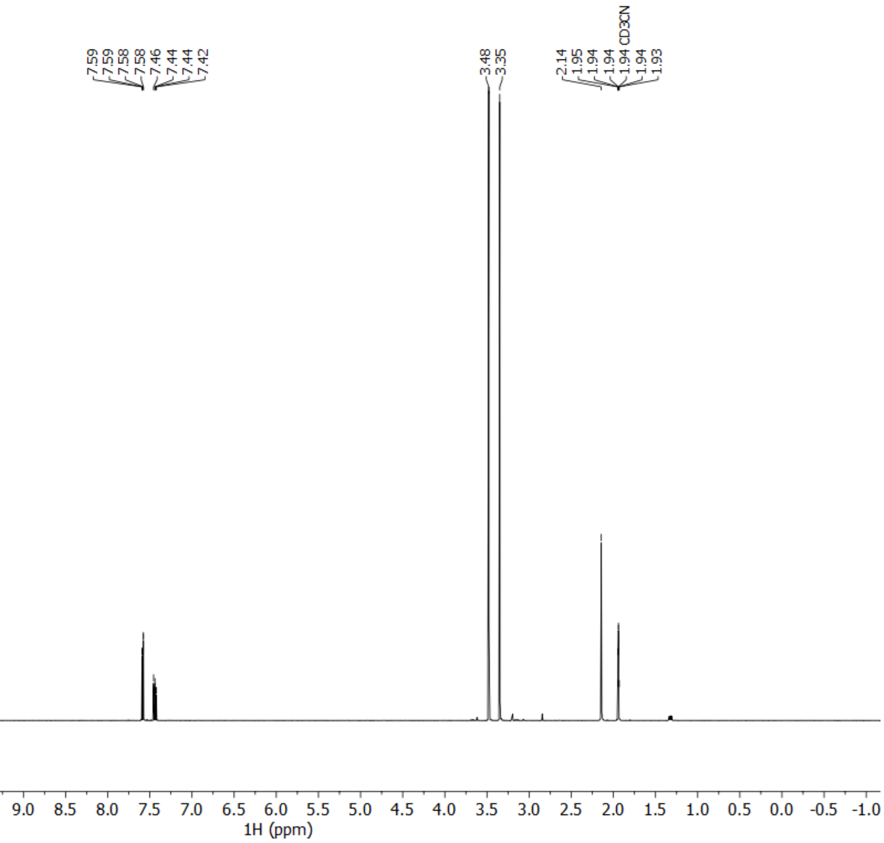

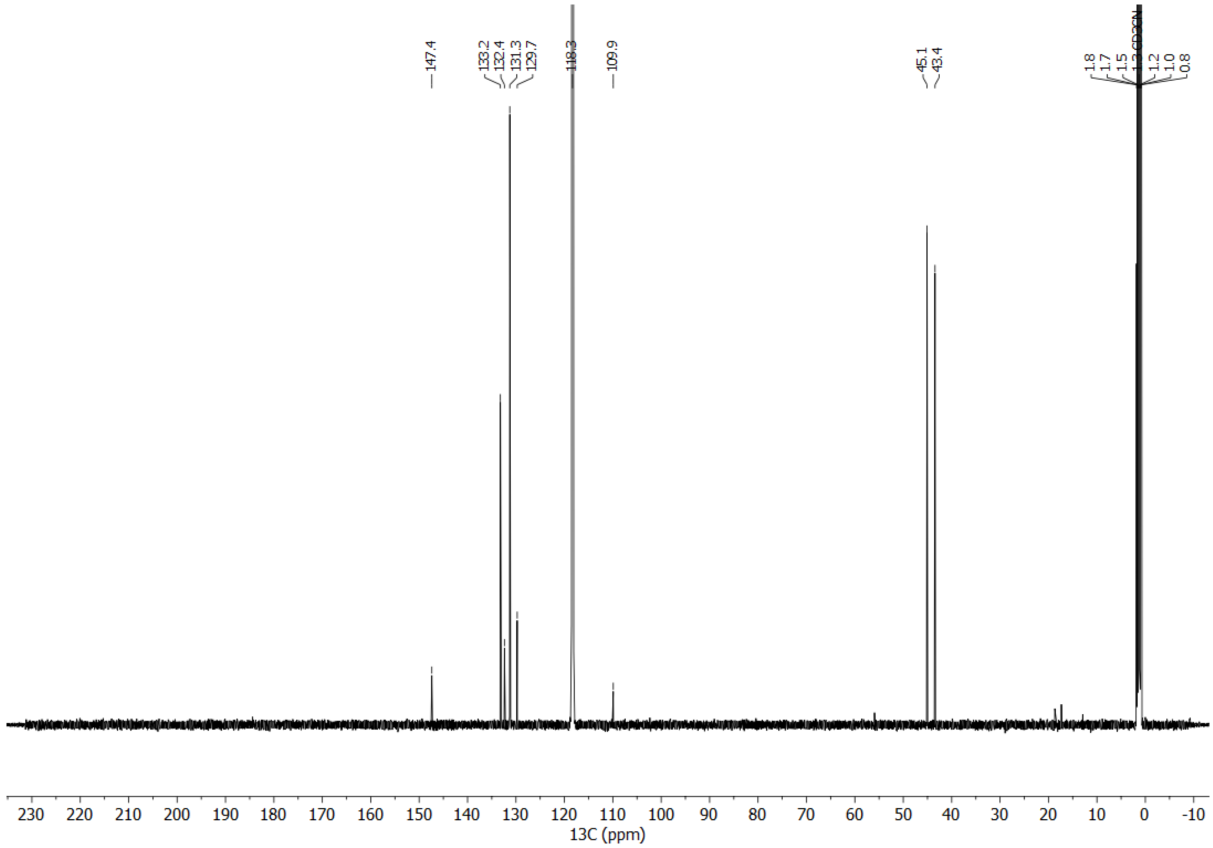

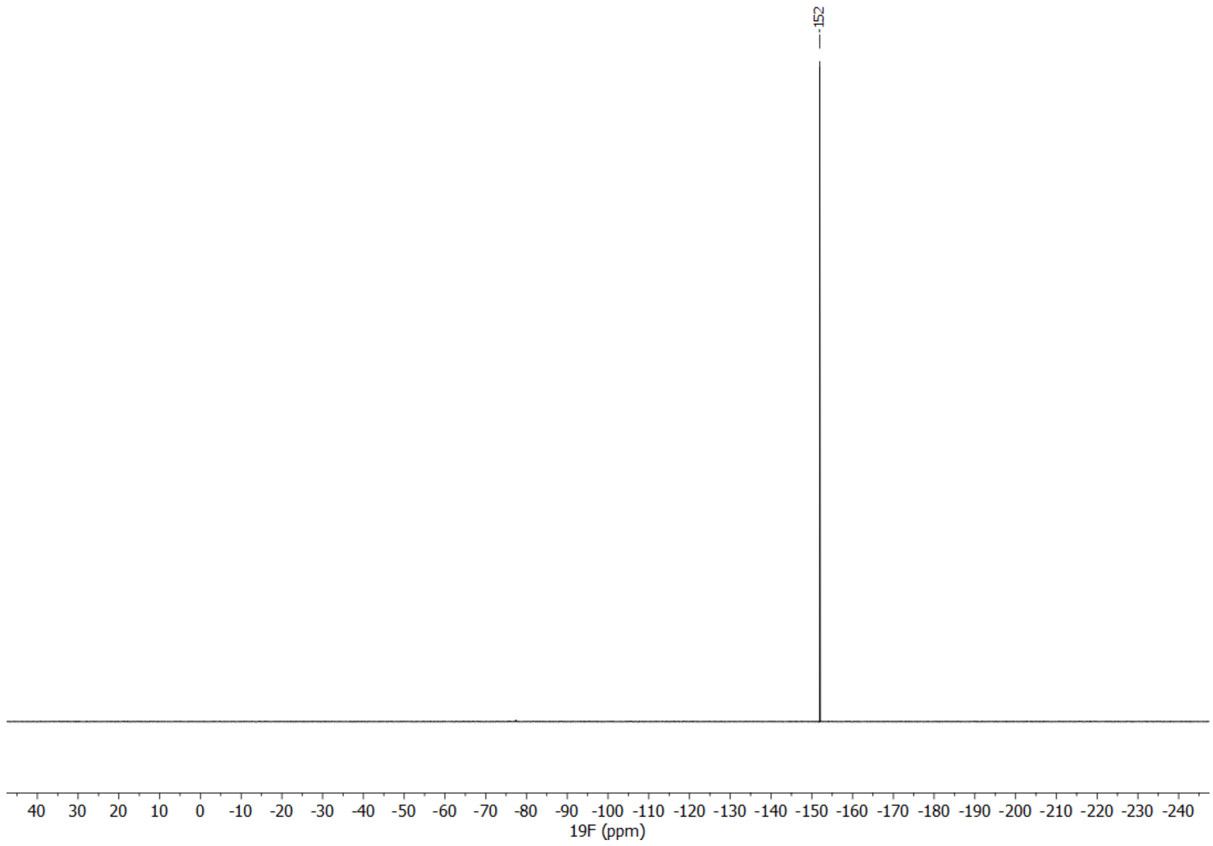
-152
40 30 20 10 0 -10 -20 -30 -40 -50 -60 -70 -80 -90 -100 -110 -120 -130 -140 -150 -160 -170 -180 -190 -200 -210 -220 -230 -240
19F (ppm)

^{1}H NMR, ^{13}C NMR, and ^{19}F NMR spectrum of compound **6s**

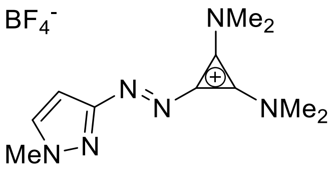

6s

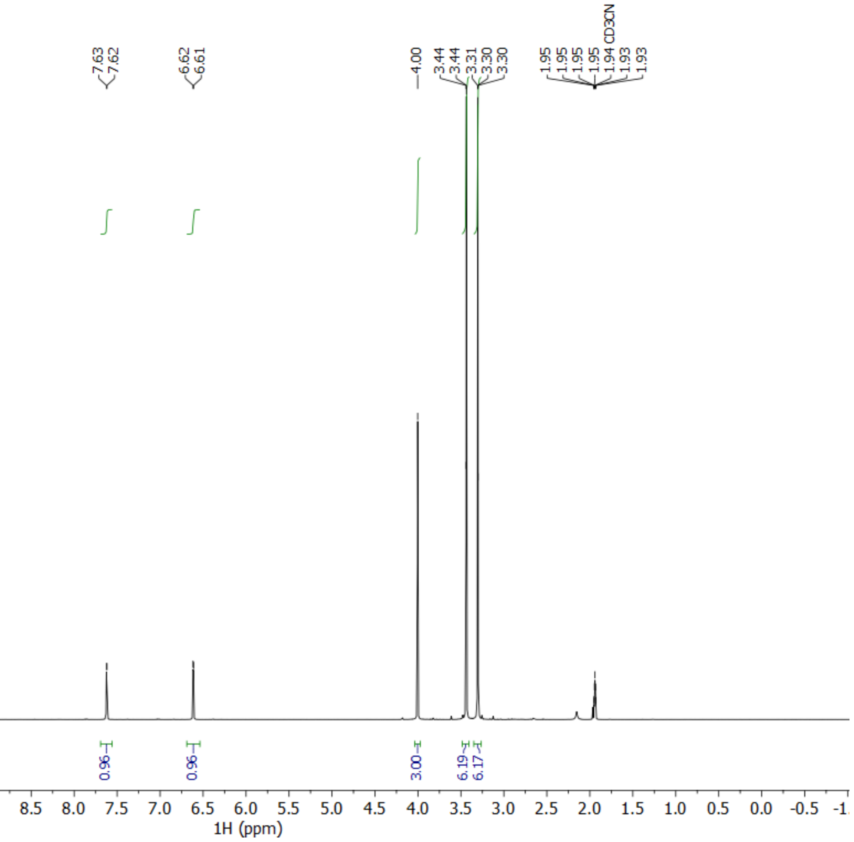

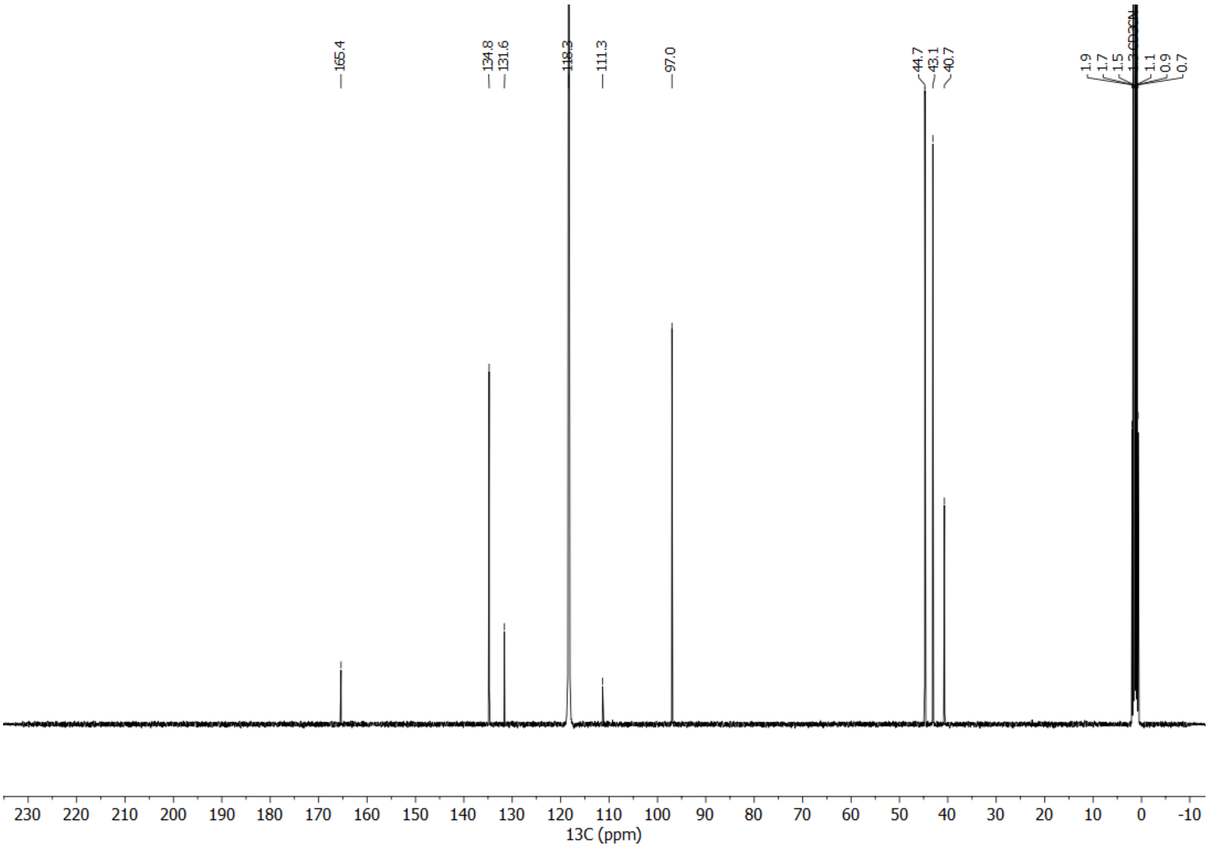

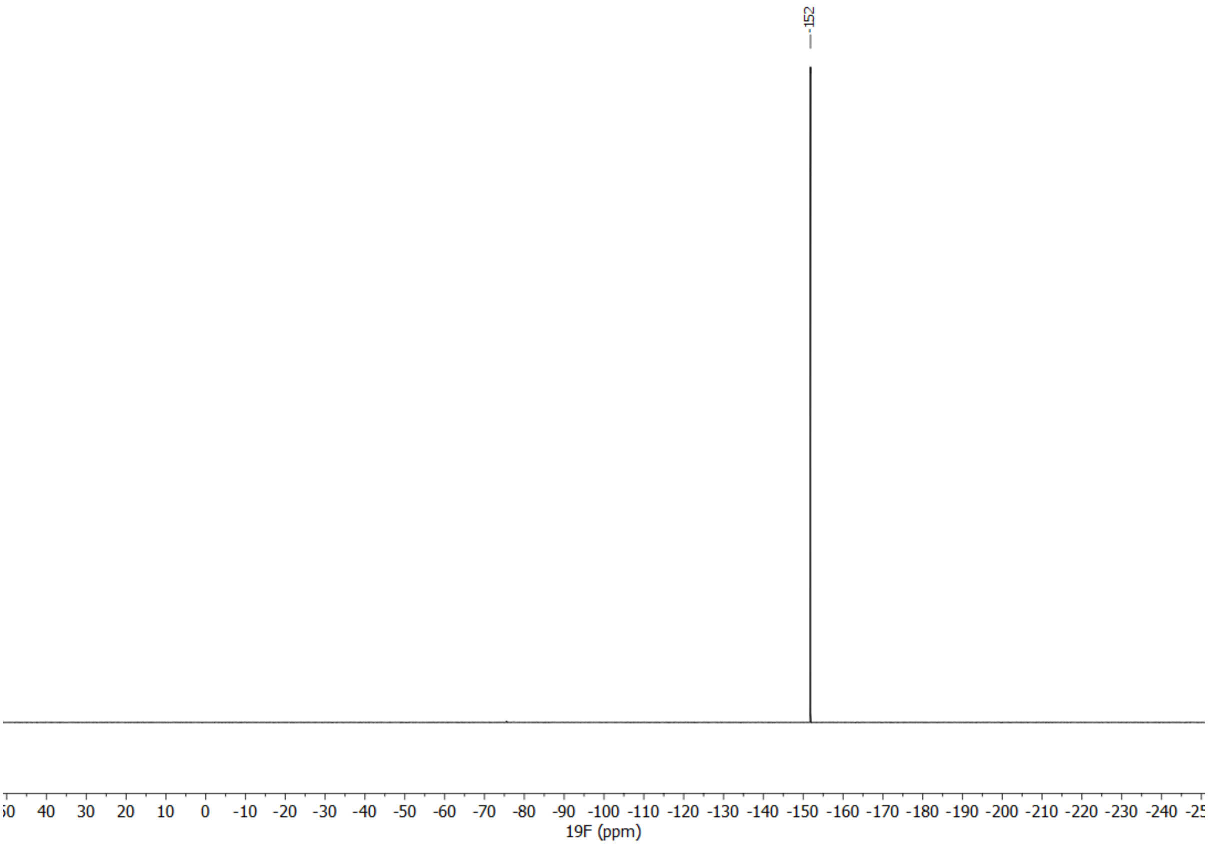
-152
50 40 30 20 10 0 -10 -20 -30 -40 -50 -60 -70 -80 -90 -100 -110 -120 -130 -140 -150 -160 -170 -180 -190 -200 -210 -220 -230 -240 -250
19F (ppm)

^{1}H NMR, ^{13}C NMR, and ^{19}F NMR spectrum of compound **6t**

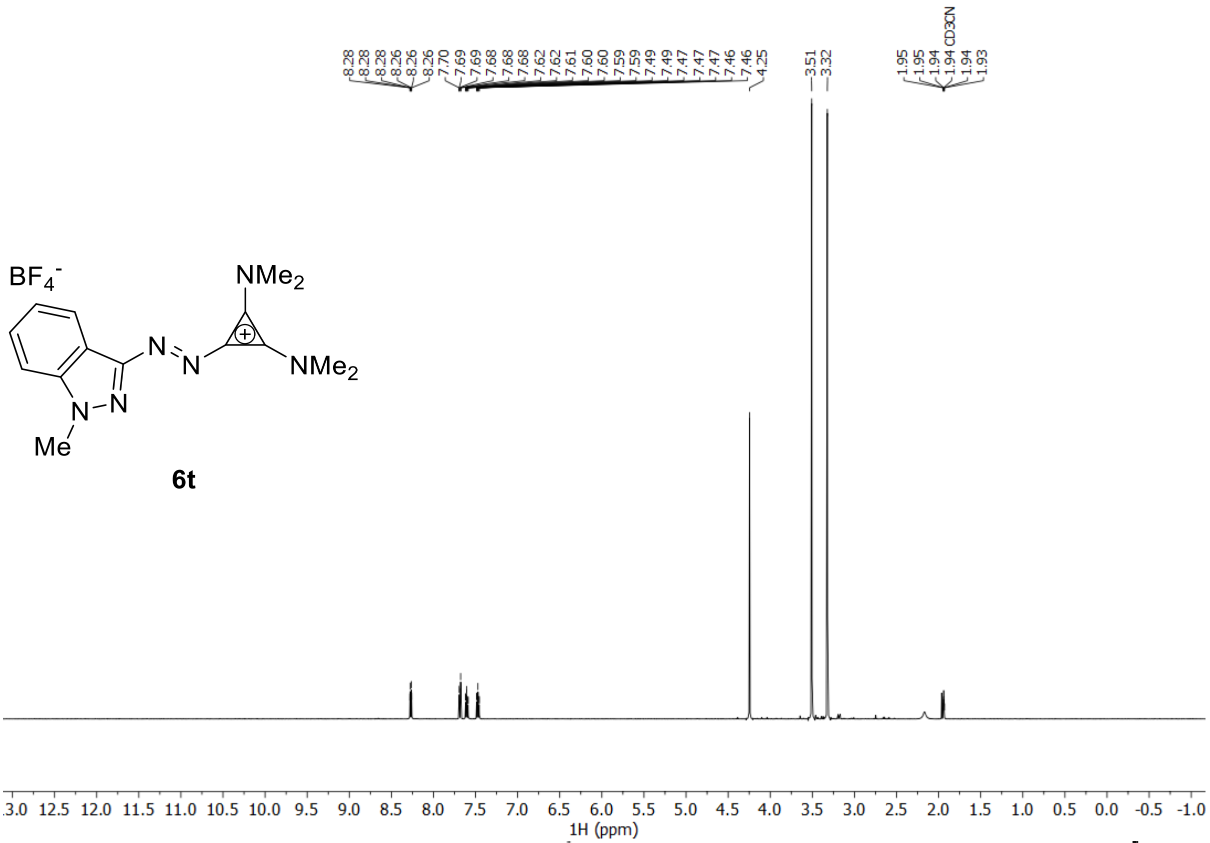

6t

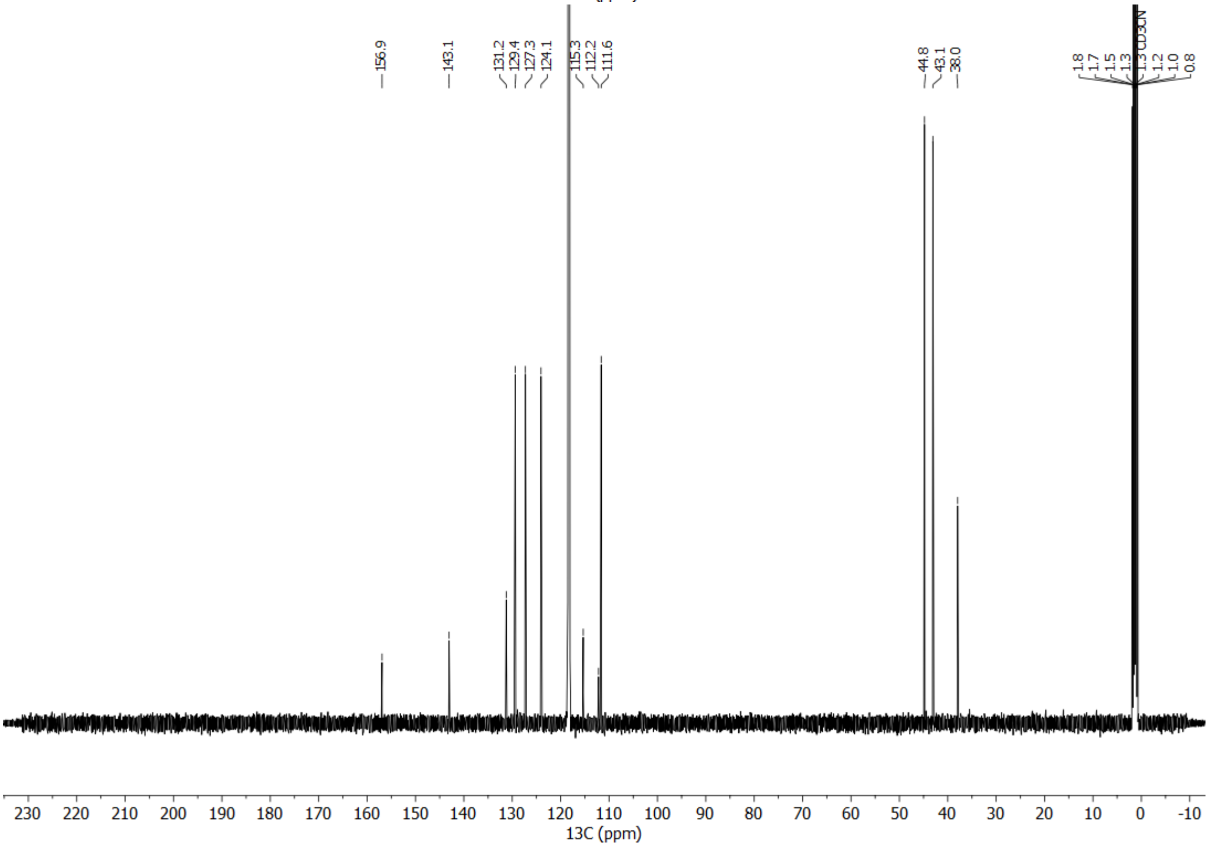

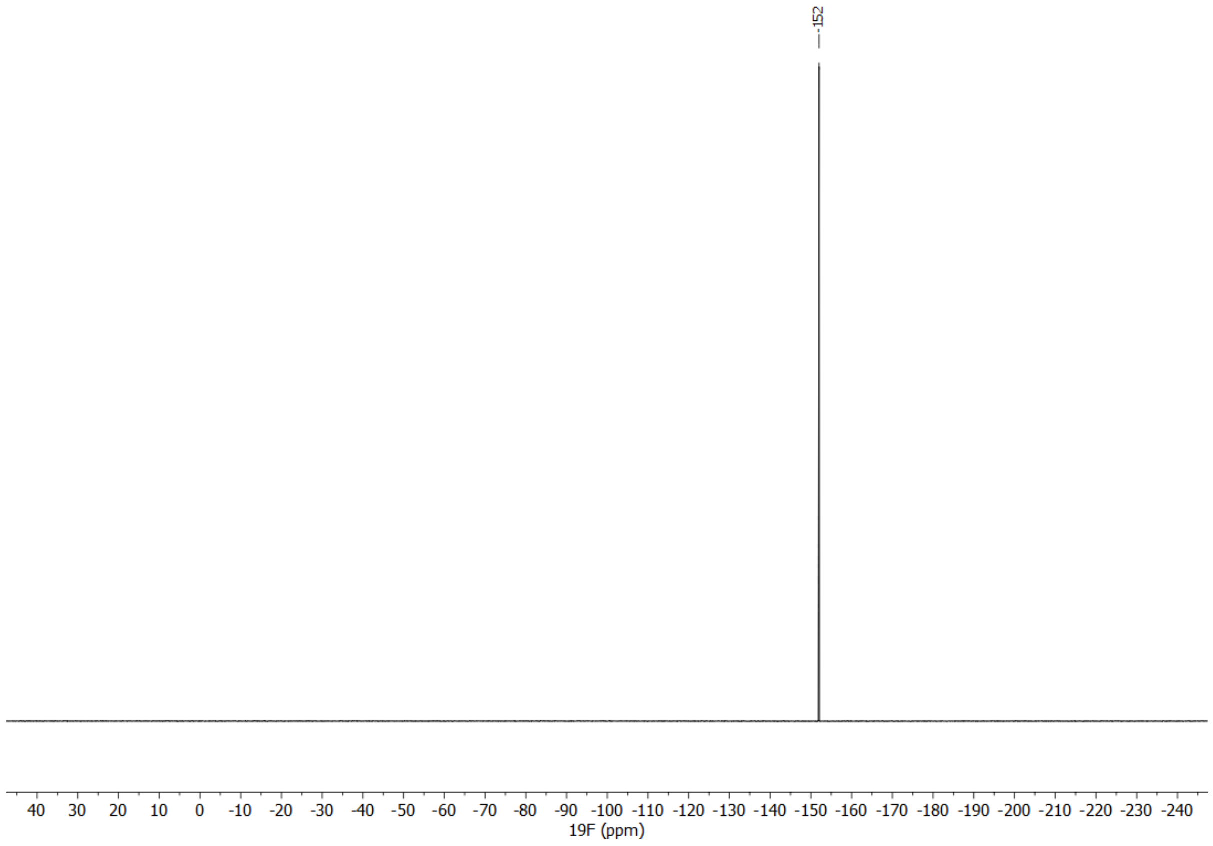

-152
40 30 20 10 0 -10 -20 -30 -40 -50 -60 -70 -80 -90 -100 -110 -120 -130 -140 -150 -160 -170 -180 -190 -200 -210 -220 -230 -240
19F (ppm)

^{1}H NMR, ^{13}C NMR, and ^{19}F NMR spectrum of compound **6u**

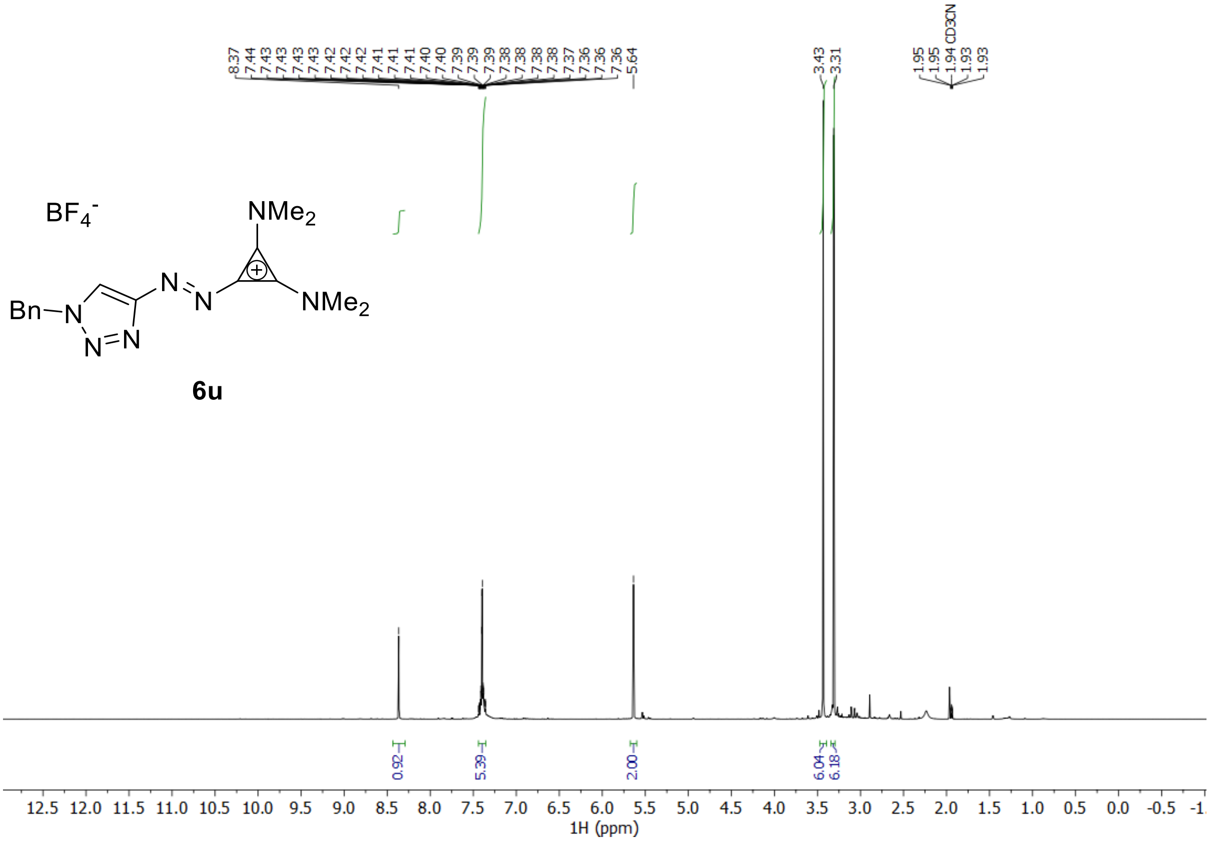

6u

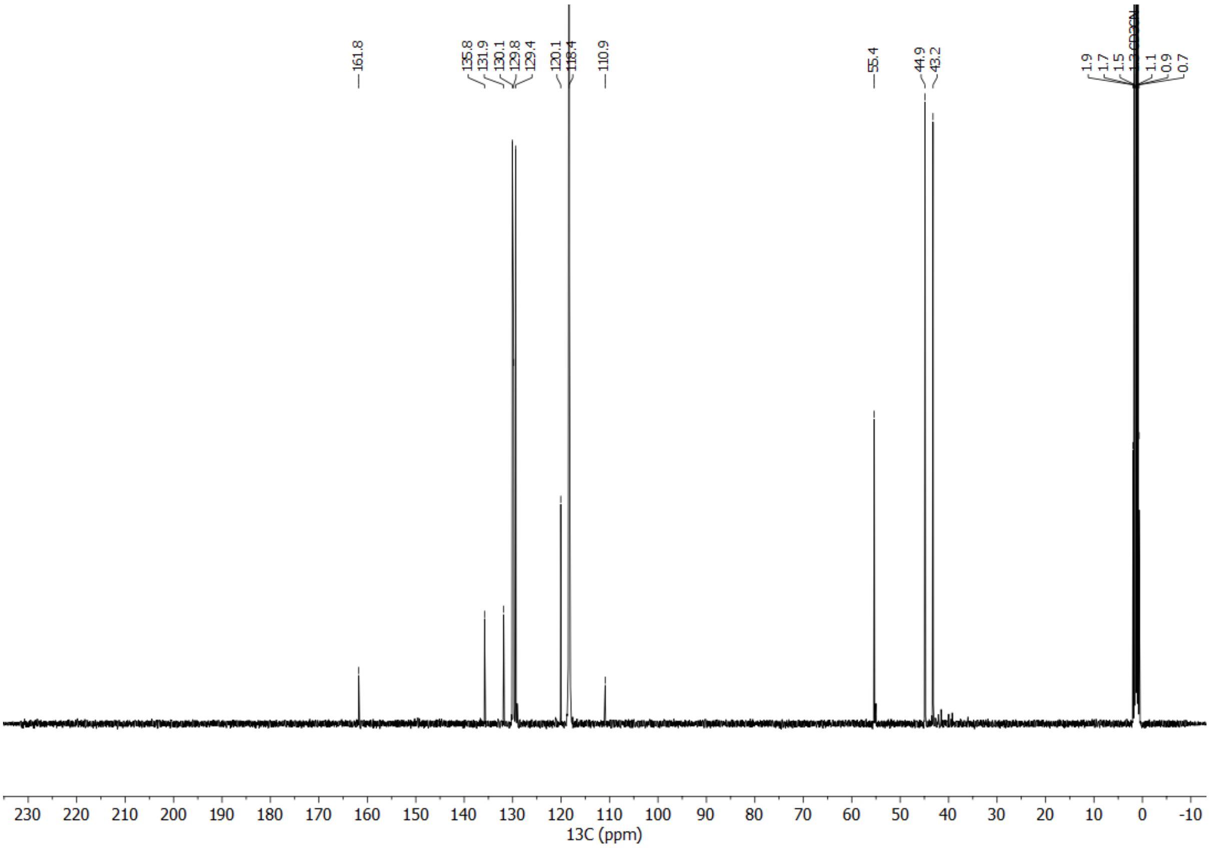

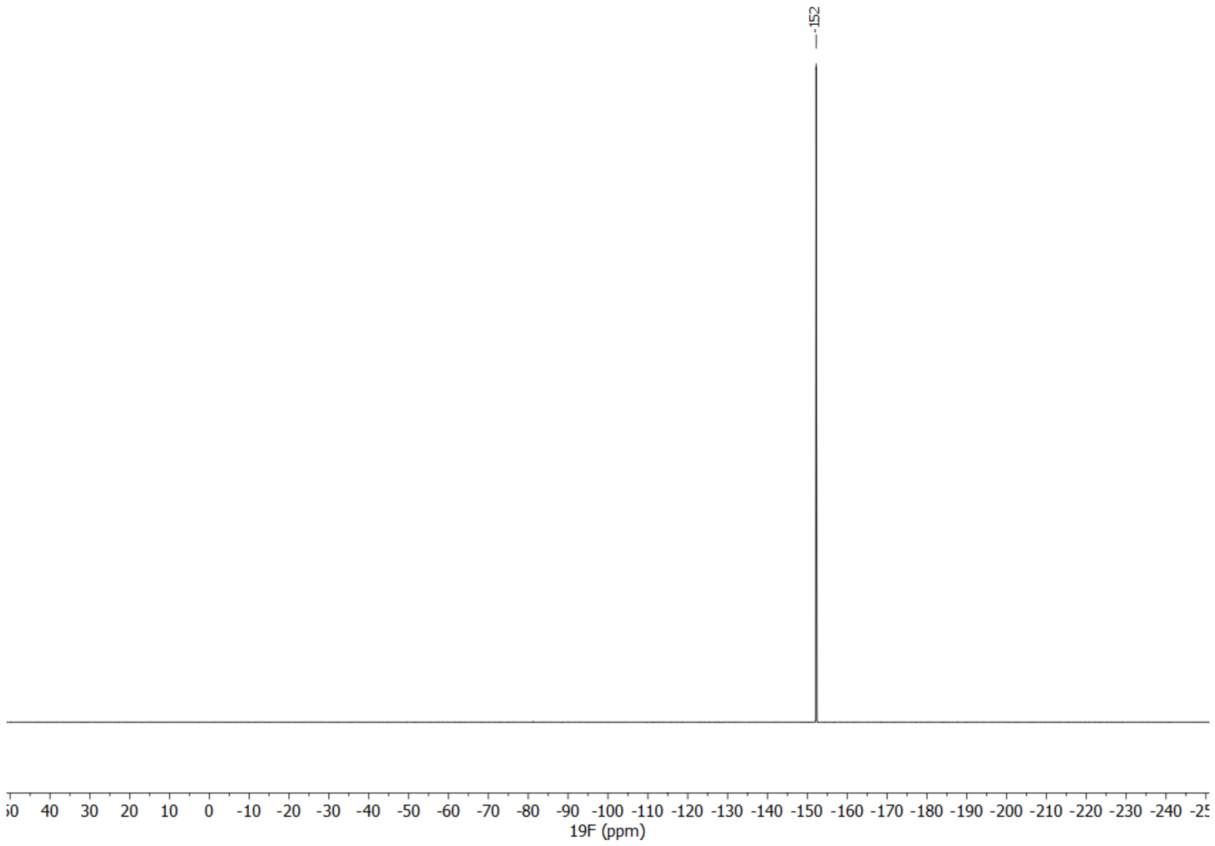
-152
50 40 30 20 10 0 -10 -20 -30 -40 -50 -60 -70 -80 -90 -100 -110 -120 -130 -140 -150 -160 -170 -180 -190 -200 -210 -220 -230 -240 -250
19F (ppm)

^{1}H NMR, ^{13}C NMR, and ^{19}F NMR spectrum of compound **SI-6v**

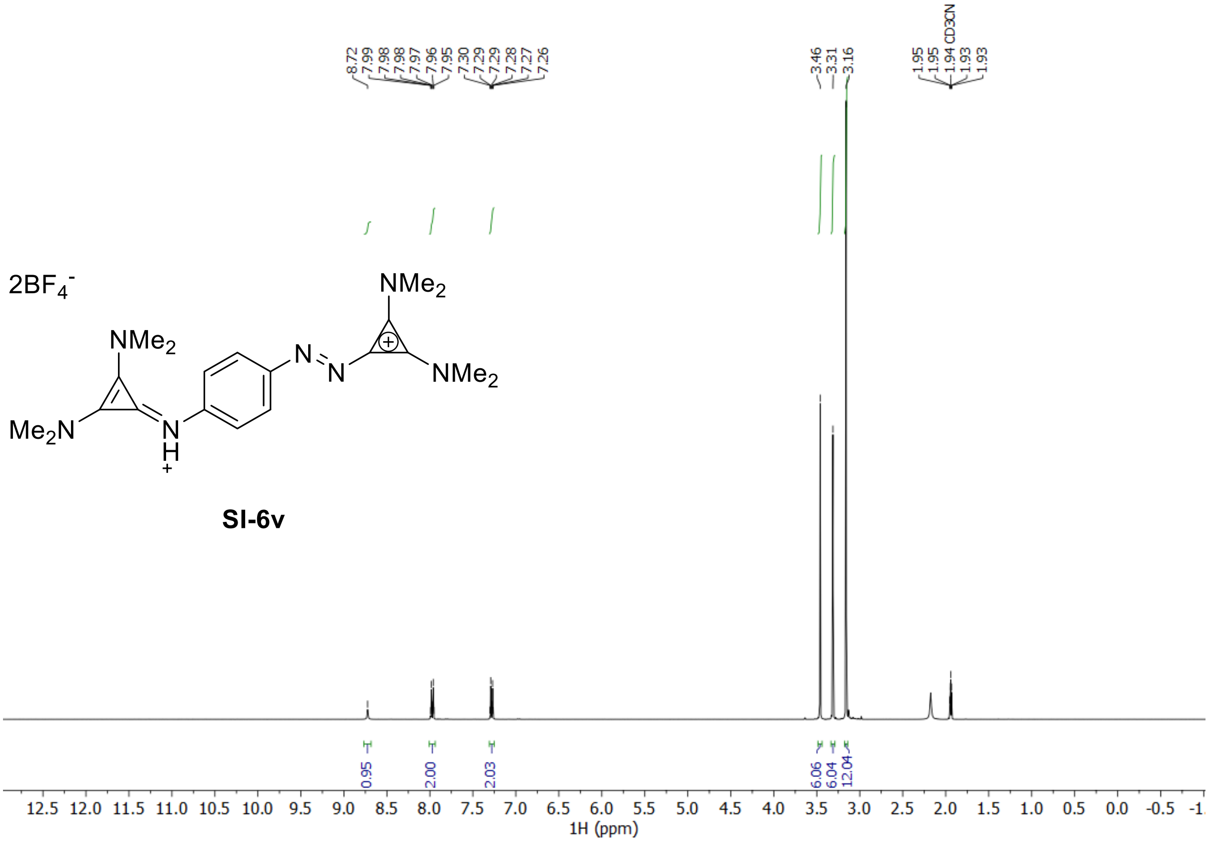

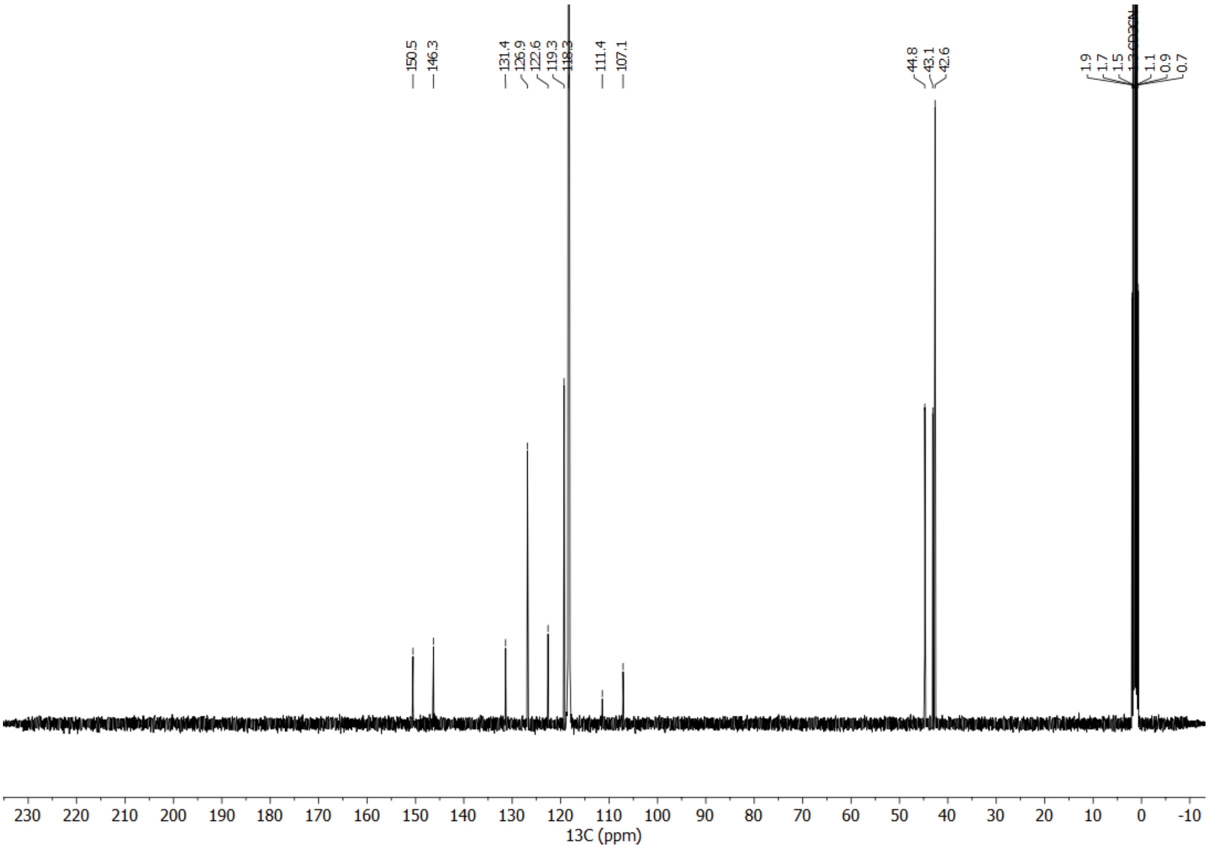

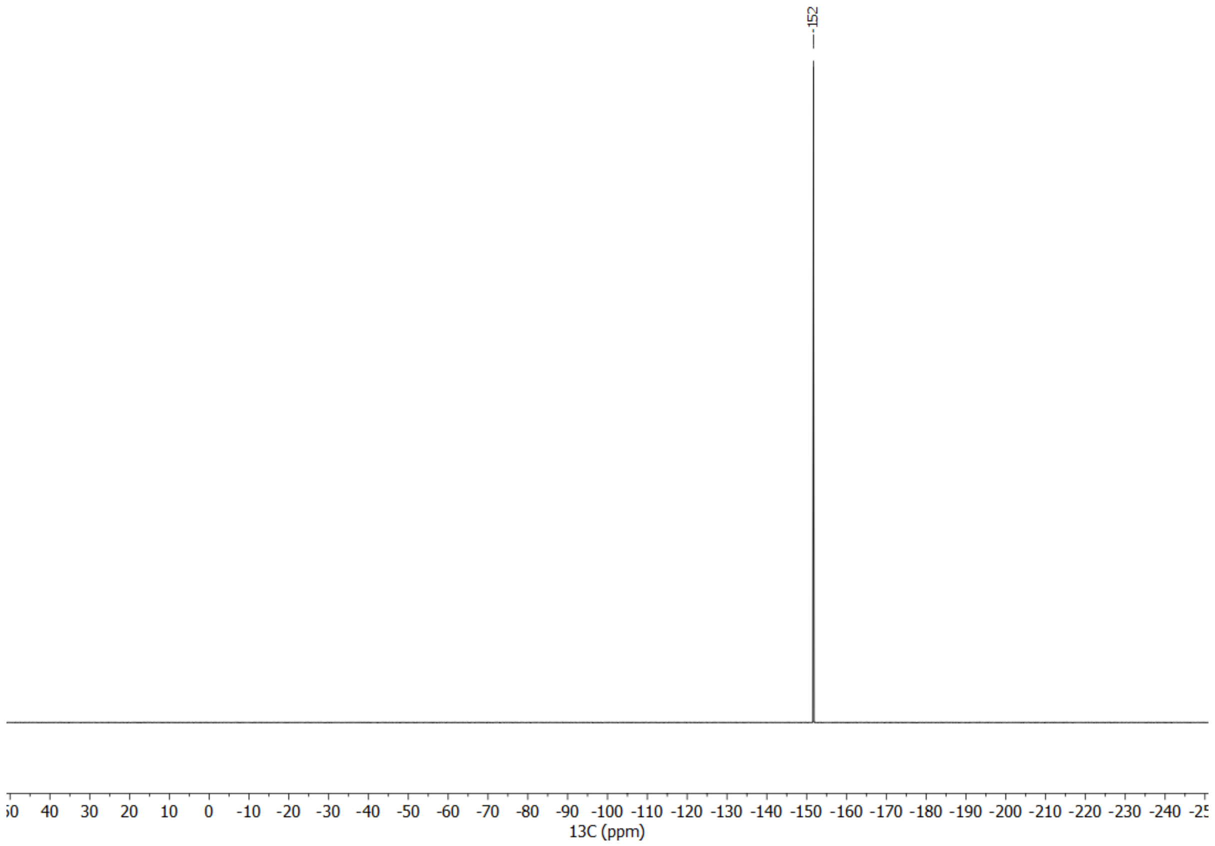
-152
40 30 20 10 0 -10 -20 -30 -40 -50 -60 -70 -80 -90 -100 -110 -120 -130 -140 -150 -160 -170 -180 -190 -200 -210 -220 -230 -240
13C (ppm)

^{1}H NMR, ^{13}C NMR, and ^{19}F NMR spectrum of compound **SI-6v'**

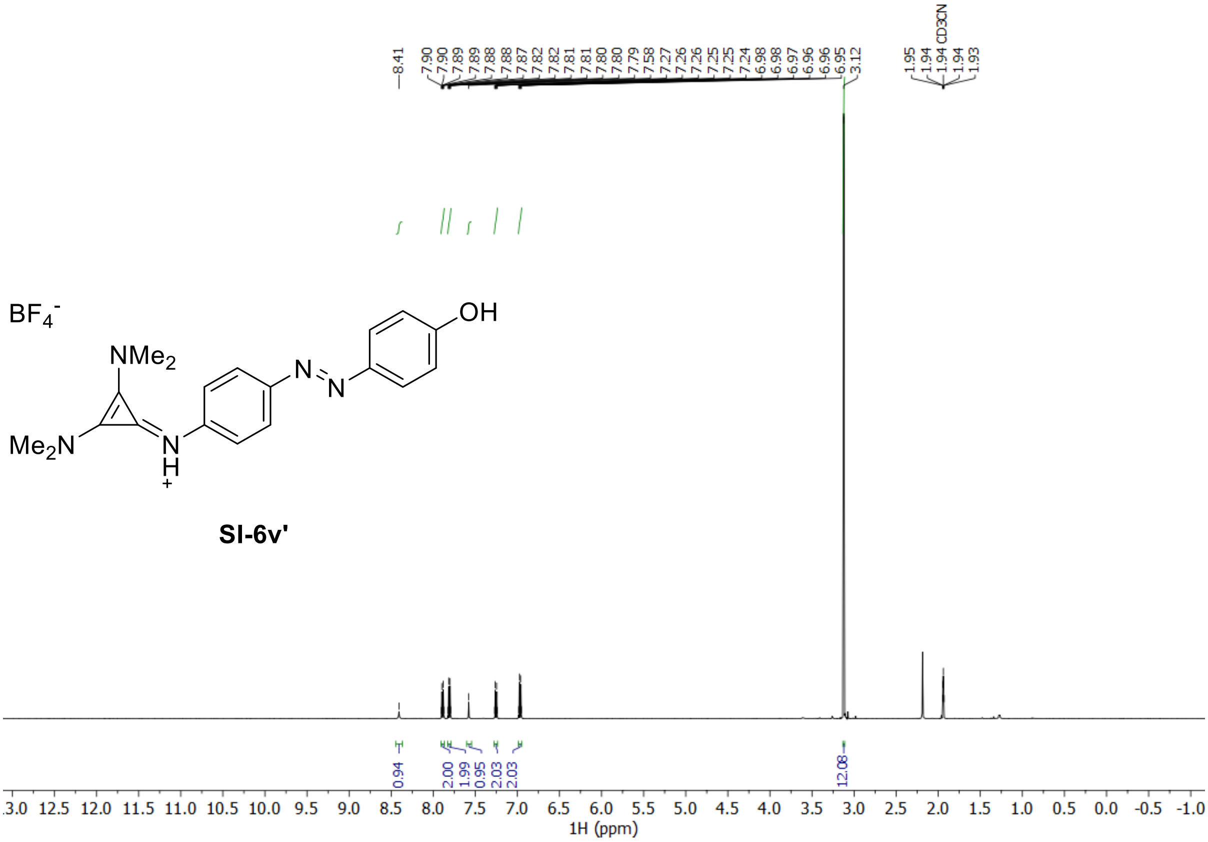

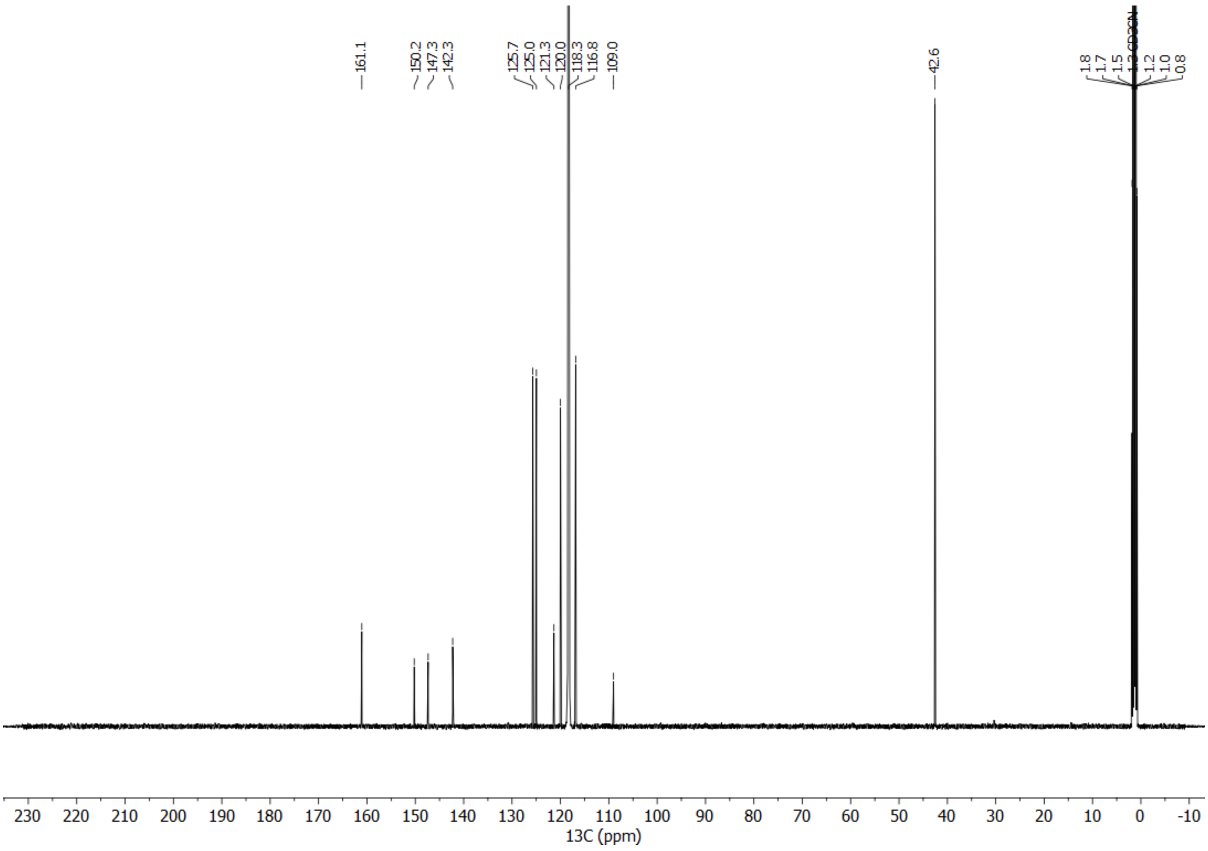

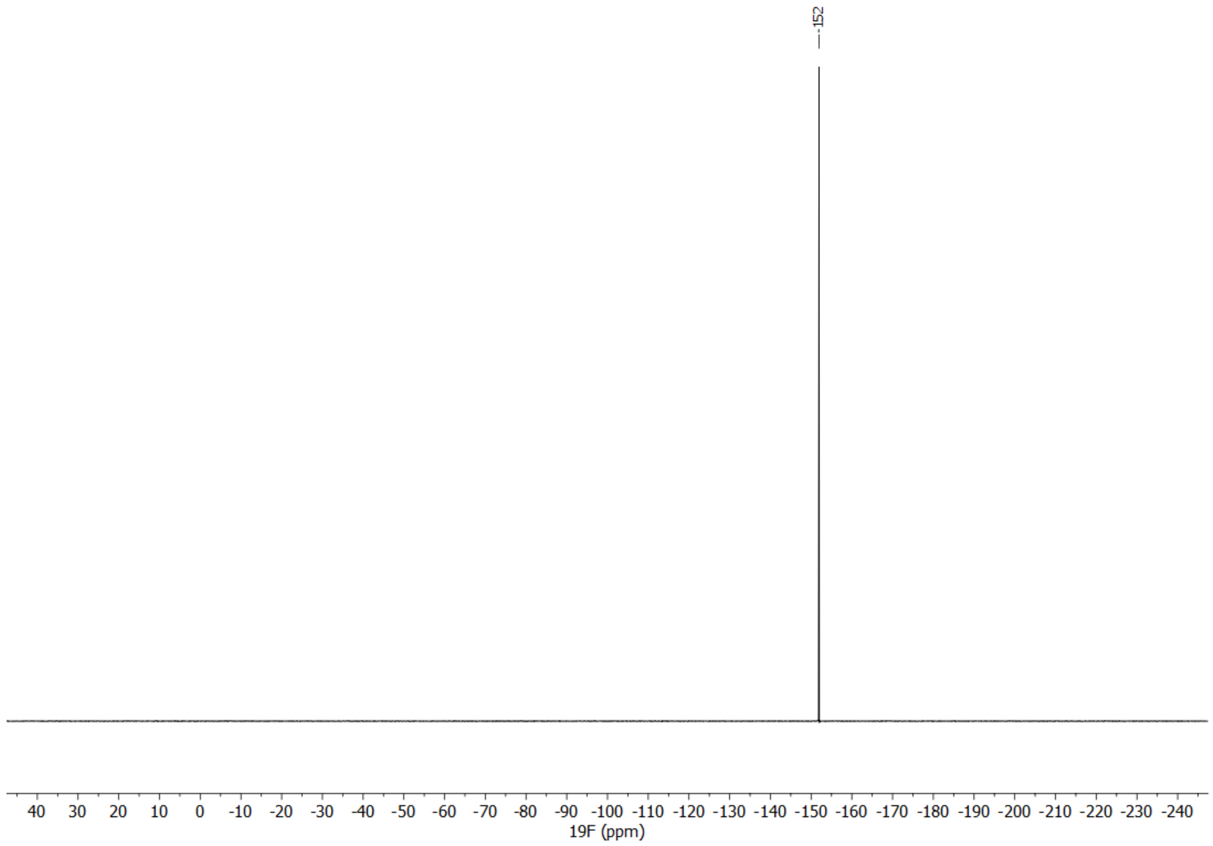
-152
40 30 20 10 0 -10 -20 -30 -40 -50 -60 -70 -80 -90 -100 -110 -120 -130 -140 -150 -160 -170 -180 -190 -200 -210 -220 -230 -240
19F (ppm)

^{1}H NMR and ^{13}C NMR spectrum of compound **8a**

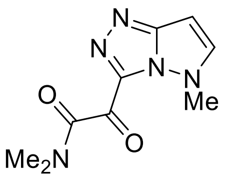

8a

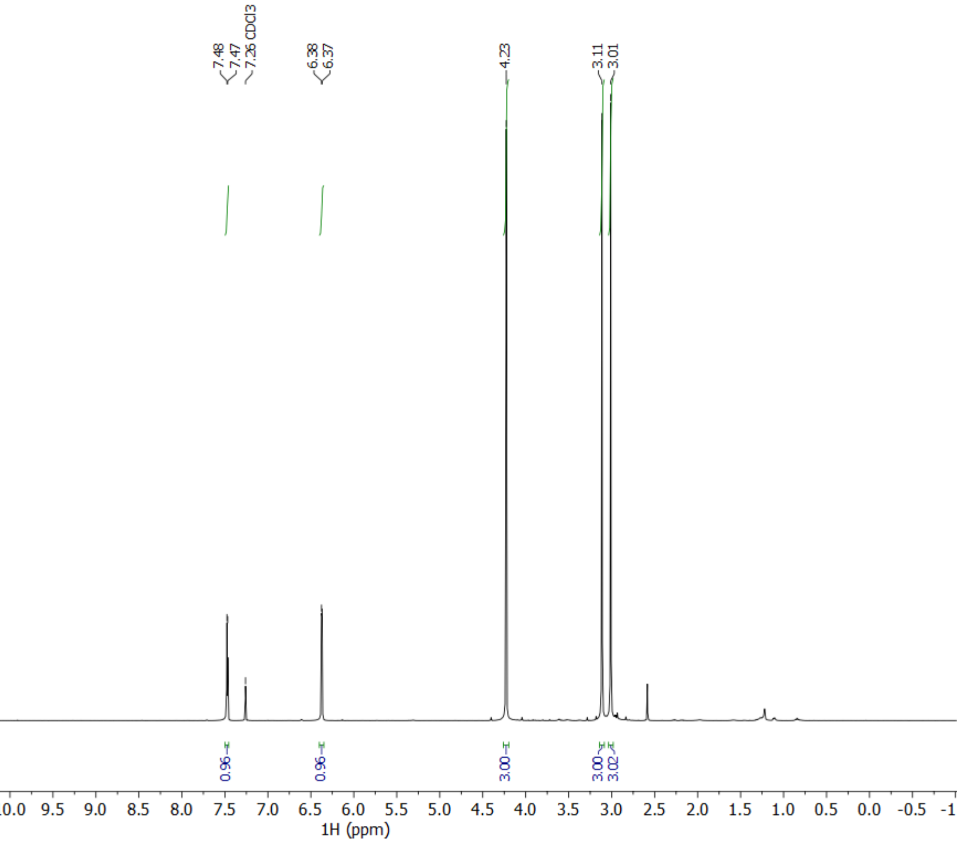

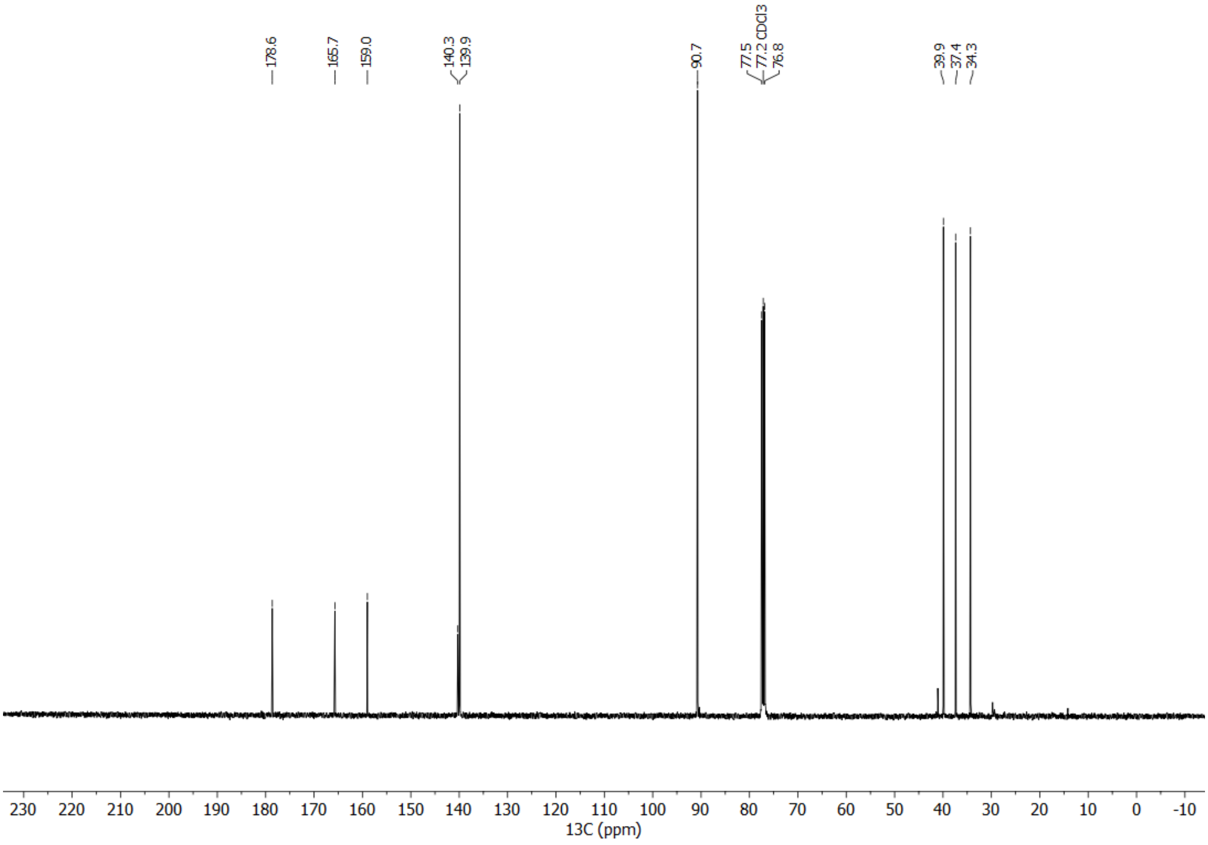

^{1}H NMR and ^{13}C NMR spectrum of compound **8b**

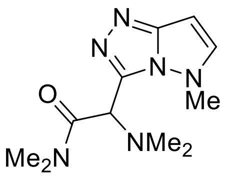

8b

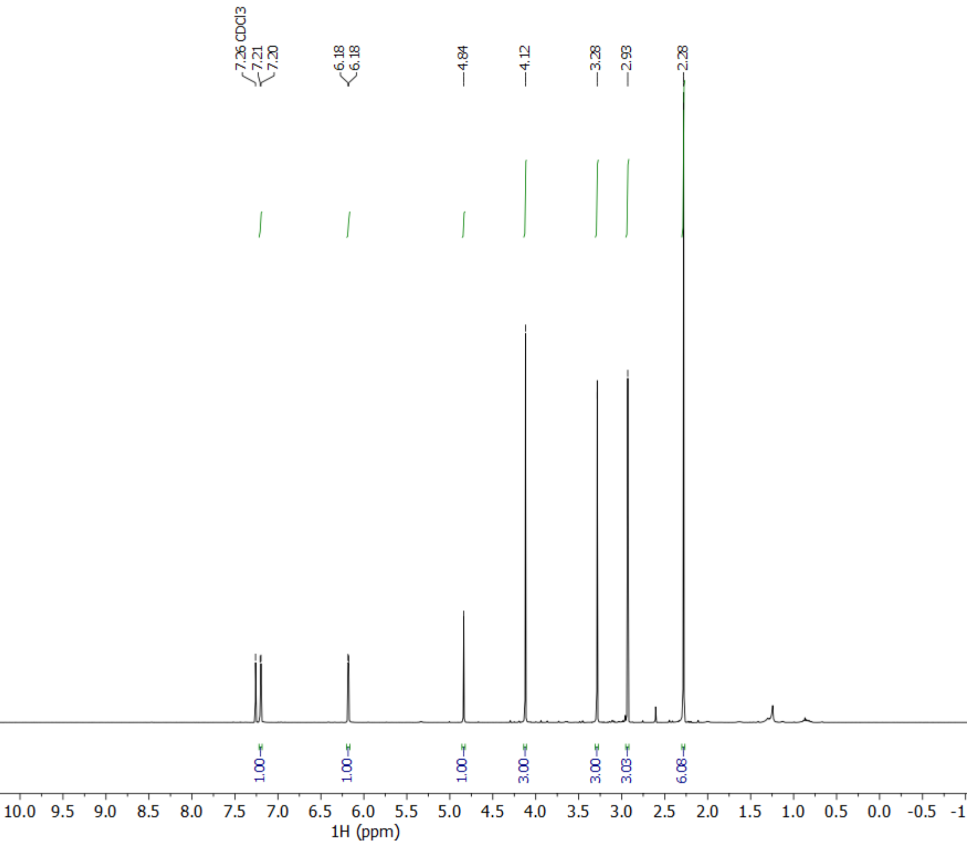

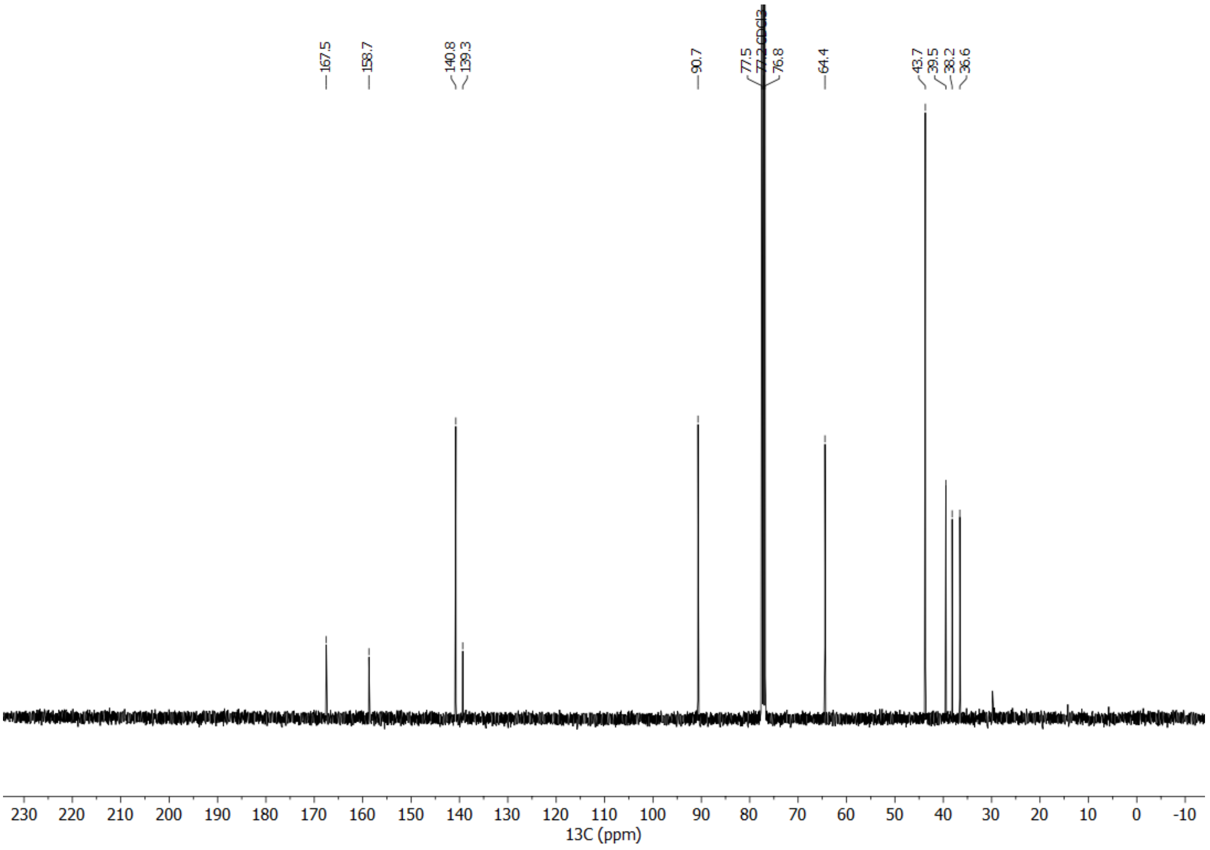

^{1}H NMR and ^{13}C NMR spectrum of compound **8c**

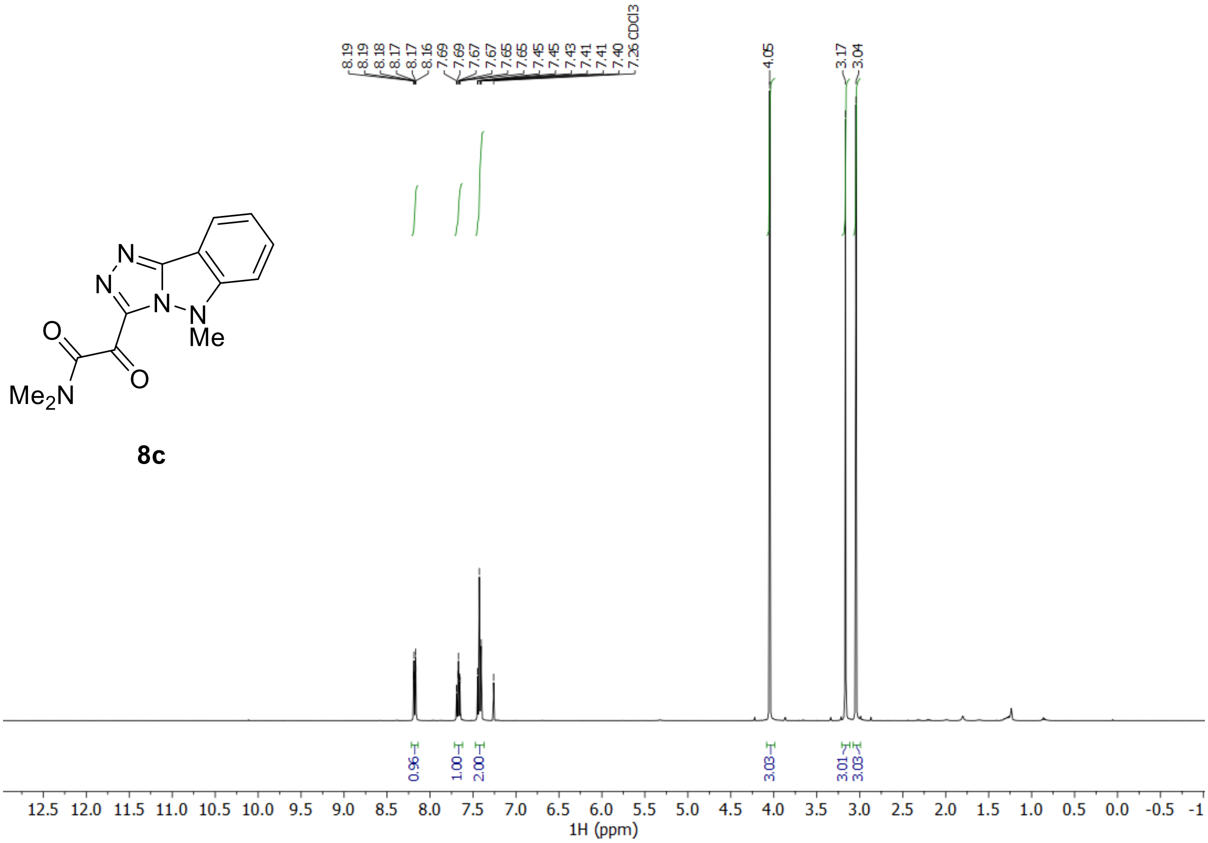

8c

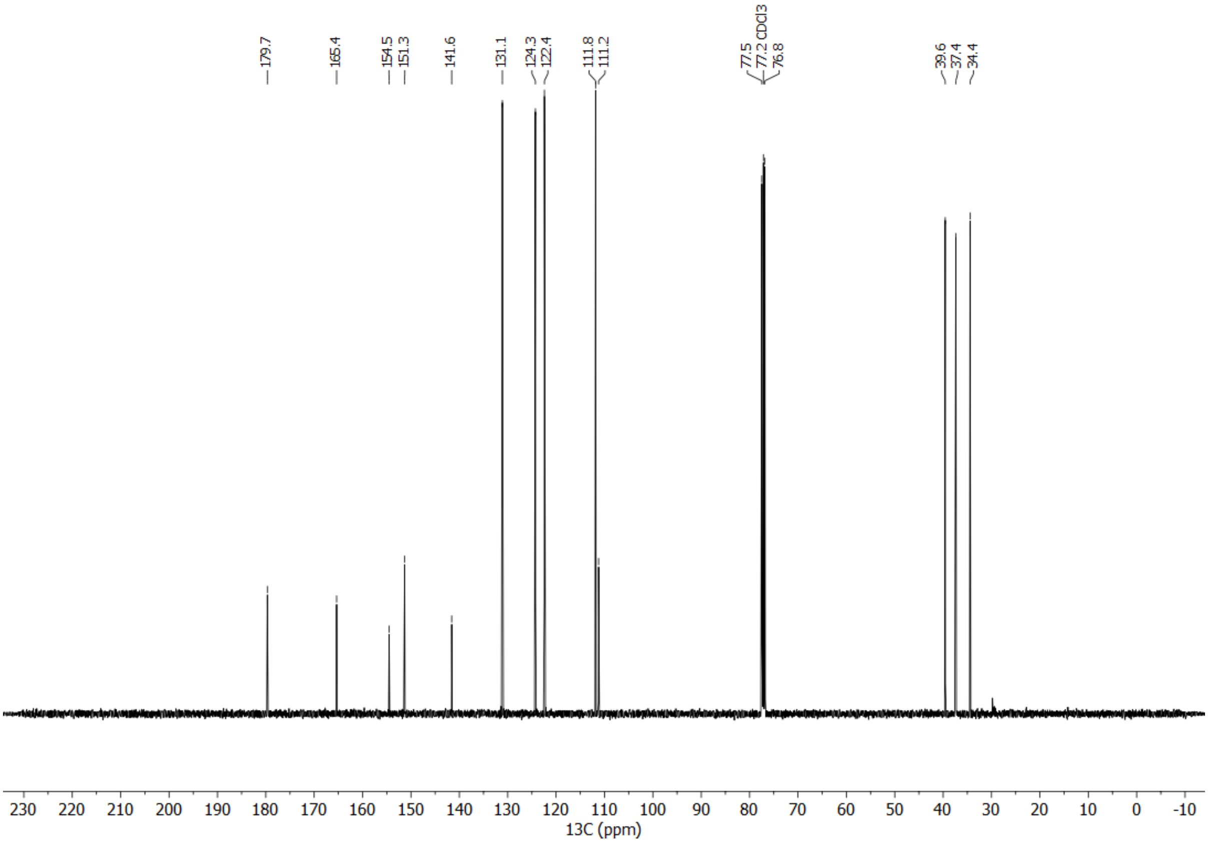

^{1}H NMR and ^{13}C NMR spectrum of compound **8d**

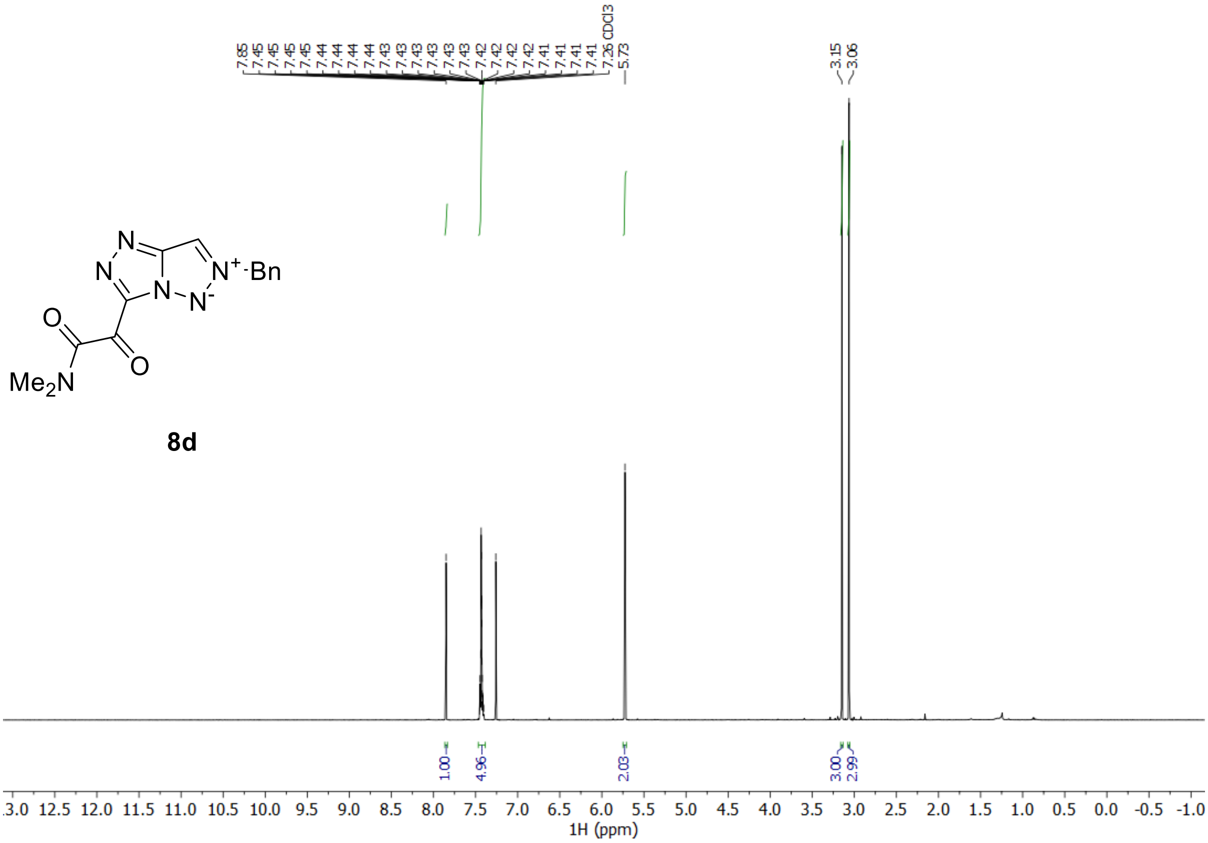

8d

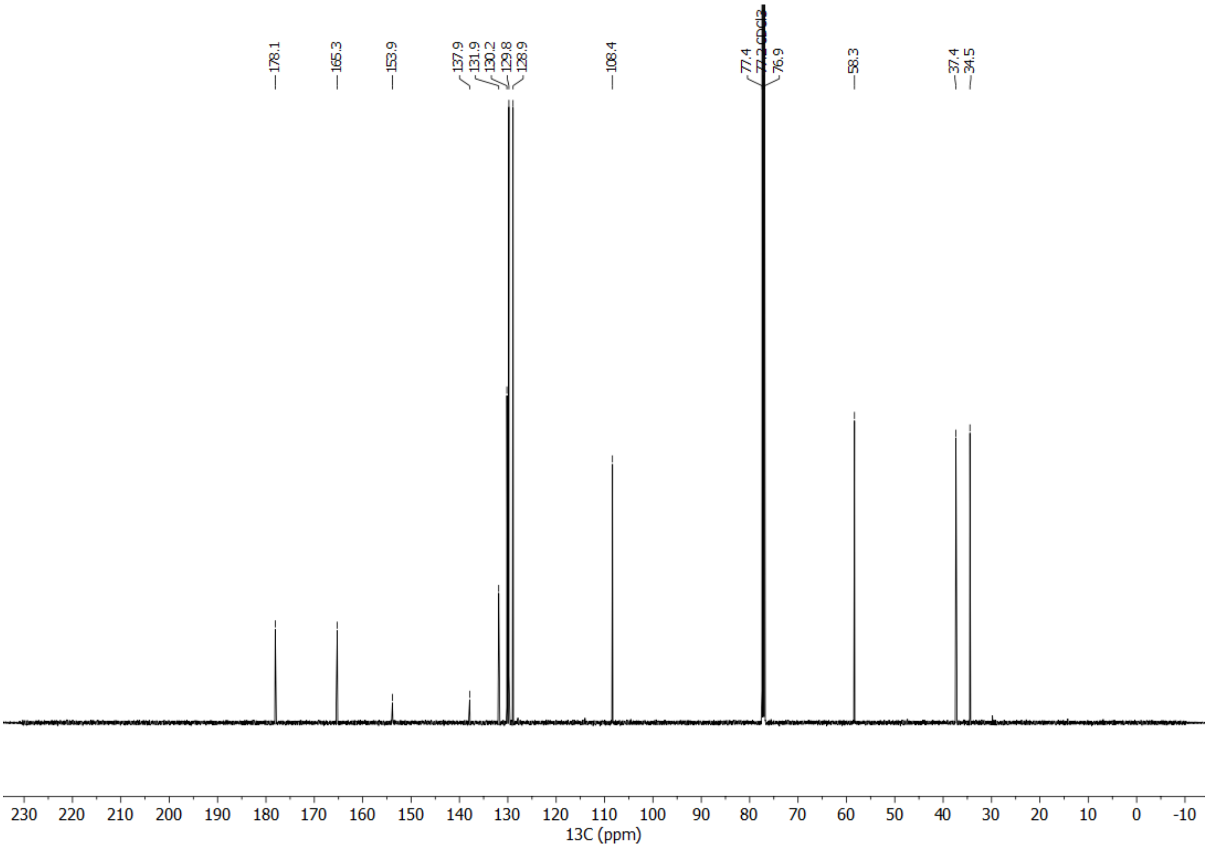

^{1}H NMR, ^{13}C NMR, and ^{19}F NMR spectrum of compound **9a**

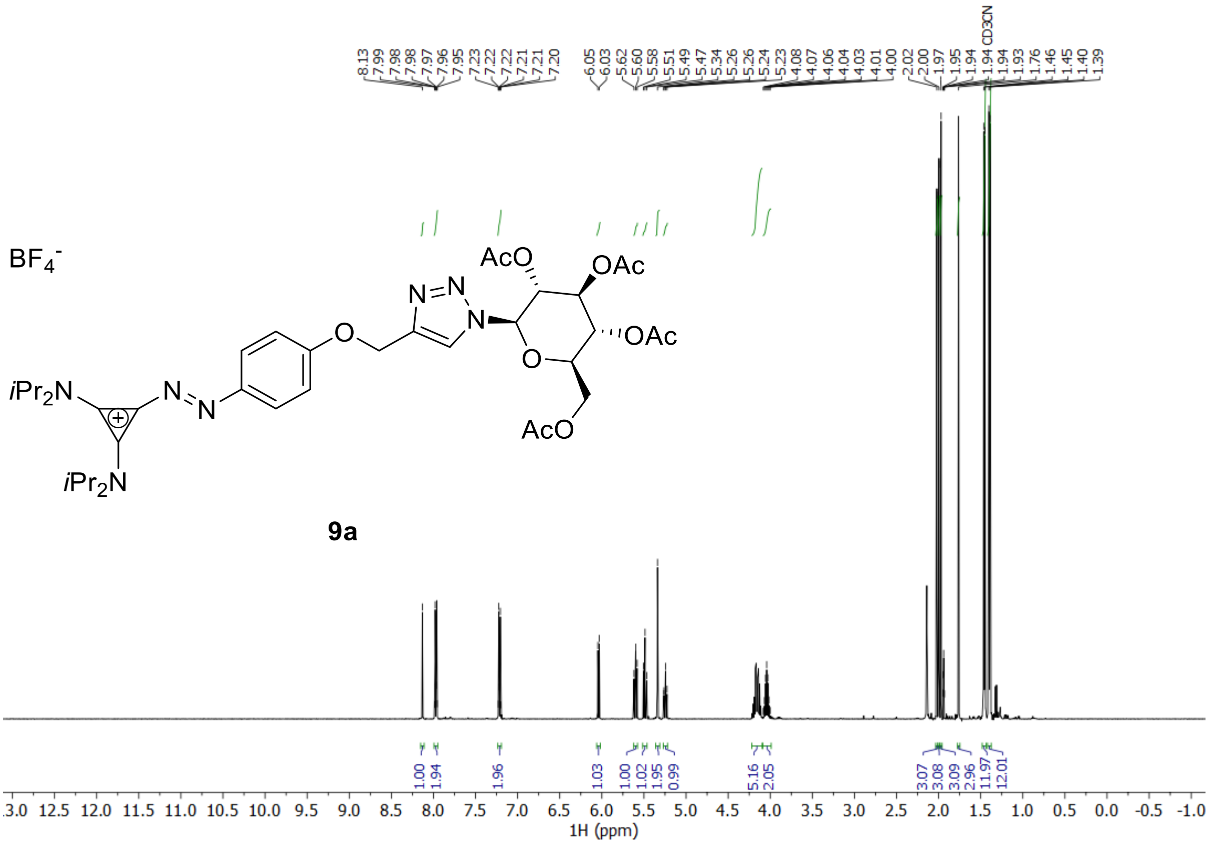

9a

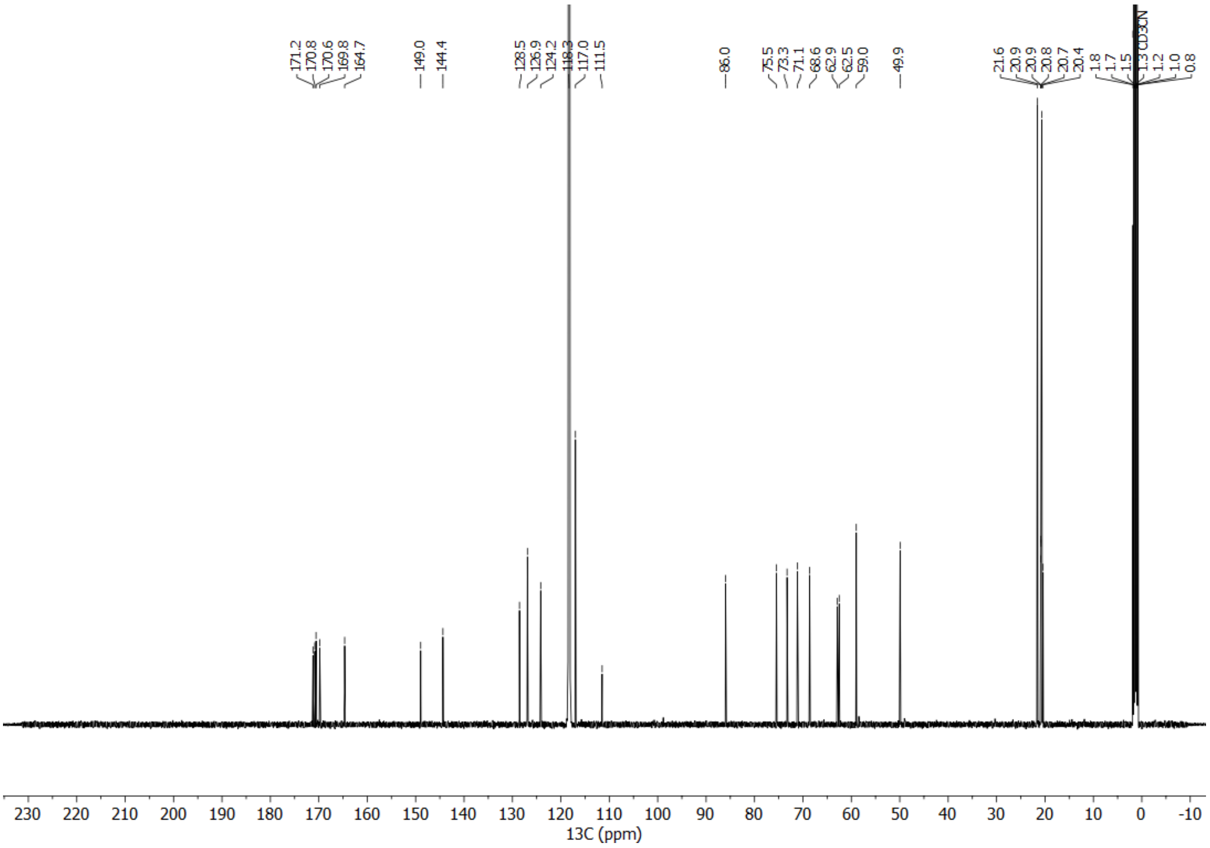

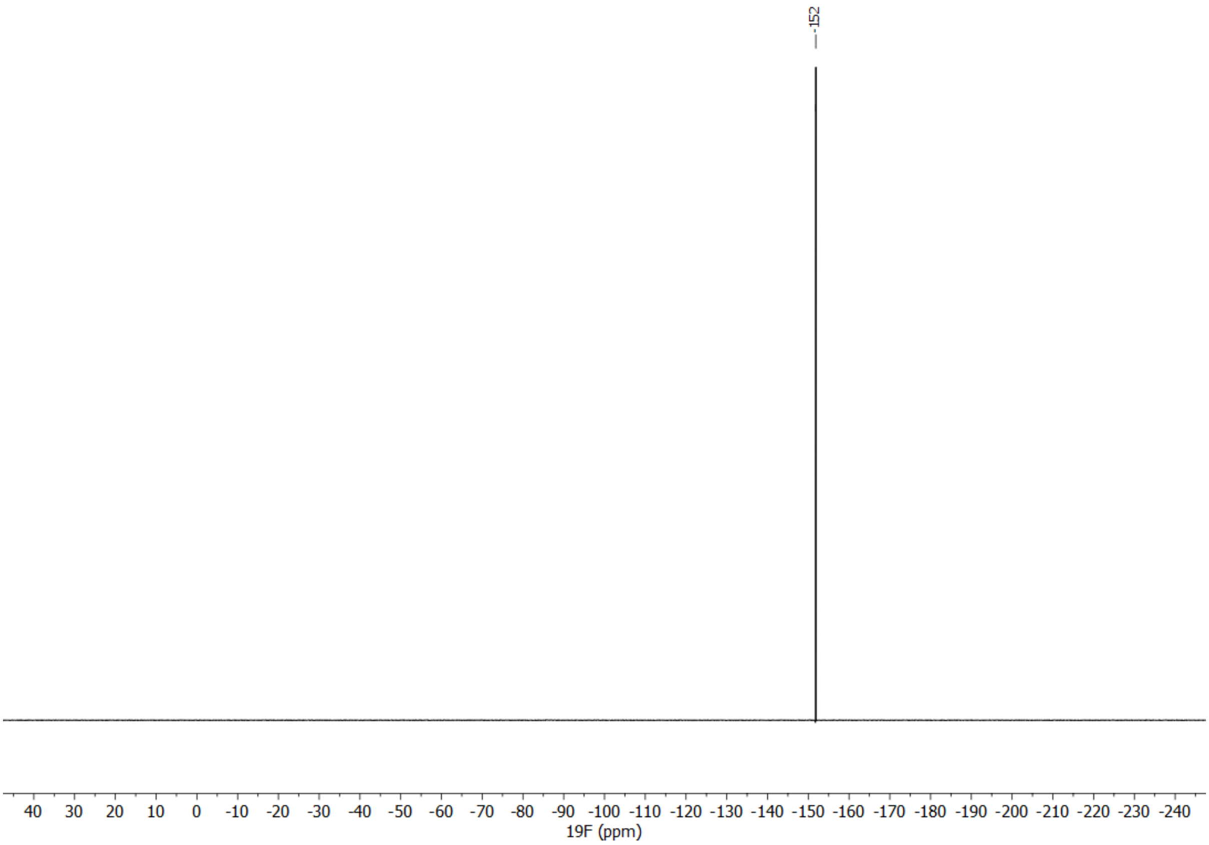
-152
40 30 20 10 0 -10 -20 -30 -40 -50 -60 -70 -80 -90 -100 -110 -120 -130 -140 -150 -160 -170 -180 -190 -200 -210 -220 -230 -240
19F (ppm)

^{1}H NMR, ^{13}C NMR, and ^{19}F NMR spectrum of compound **9b**

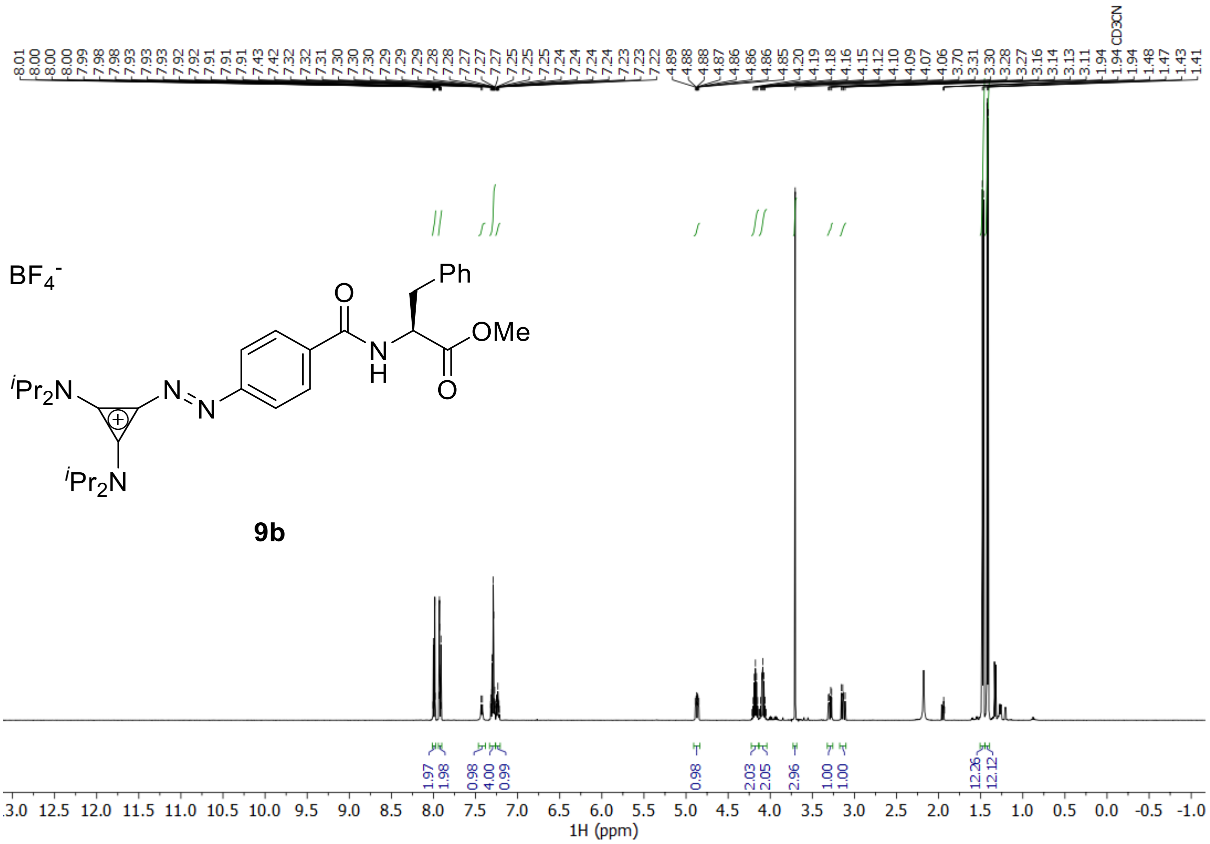

9b

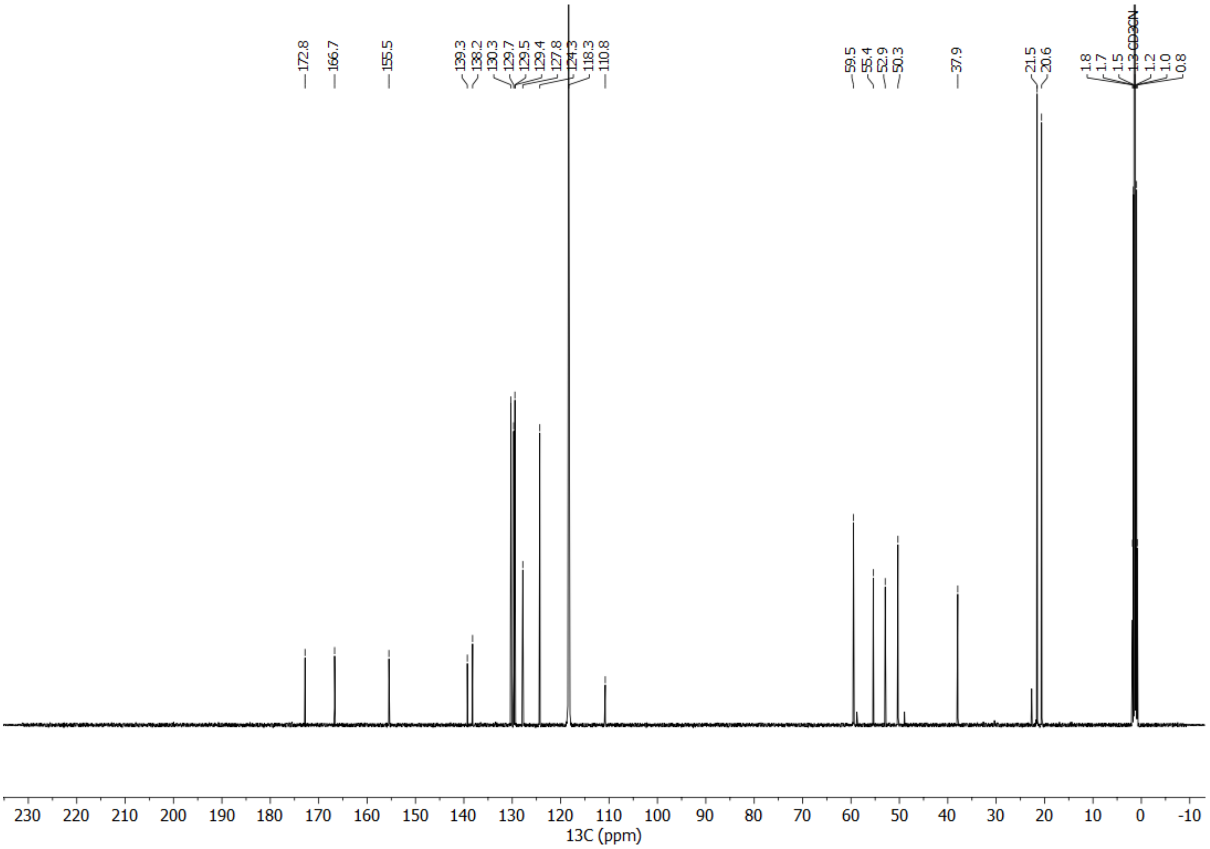

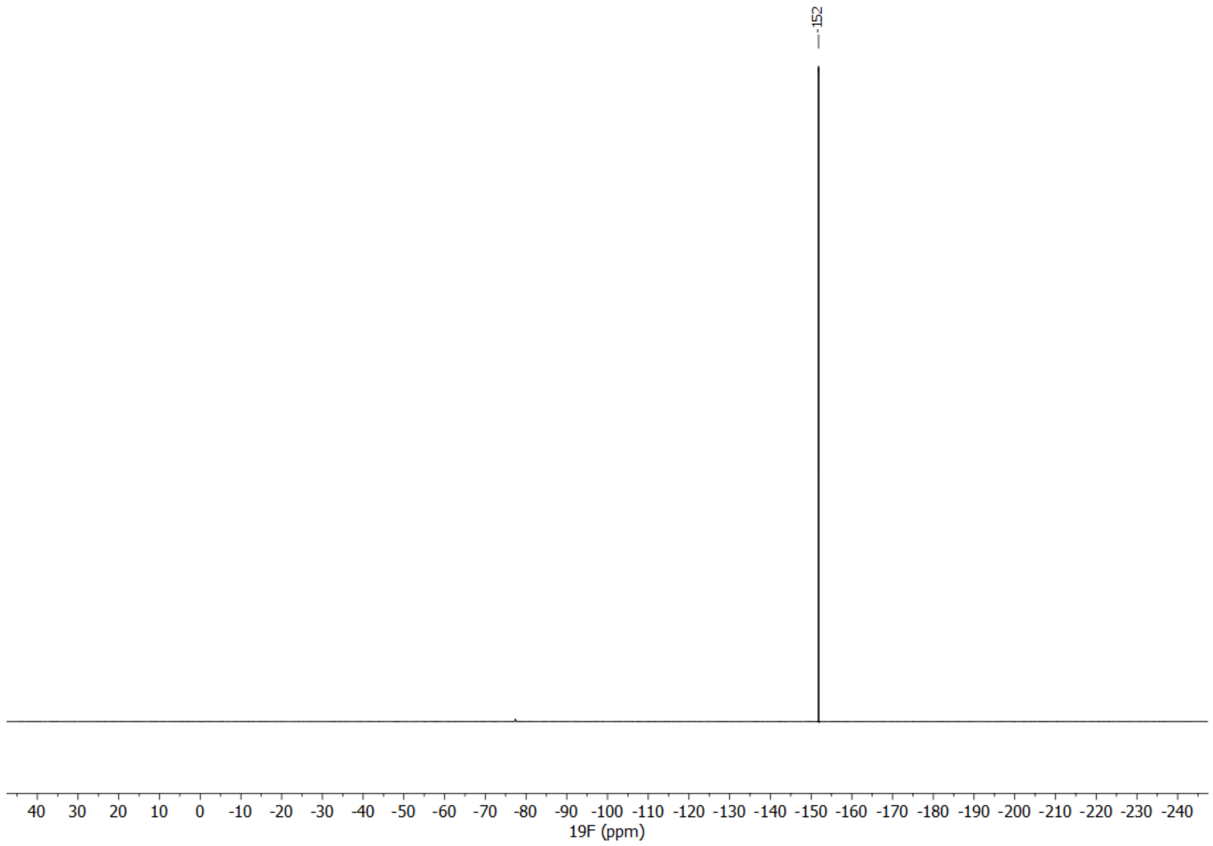

-152
40 30 20 10 0 -10 -20 -30 -40 -50 -60 -70 -80 -90 -100 -110 -120 -130 -140 -150 -160 -170 -180 -190 -200 -210 -220 -230 -240
19F (ppm)

^{1}H NMR and ^{13}C NMR spectrum of compound **10**

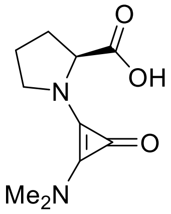

10

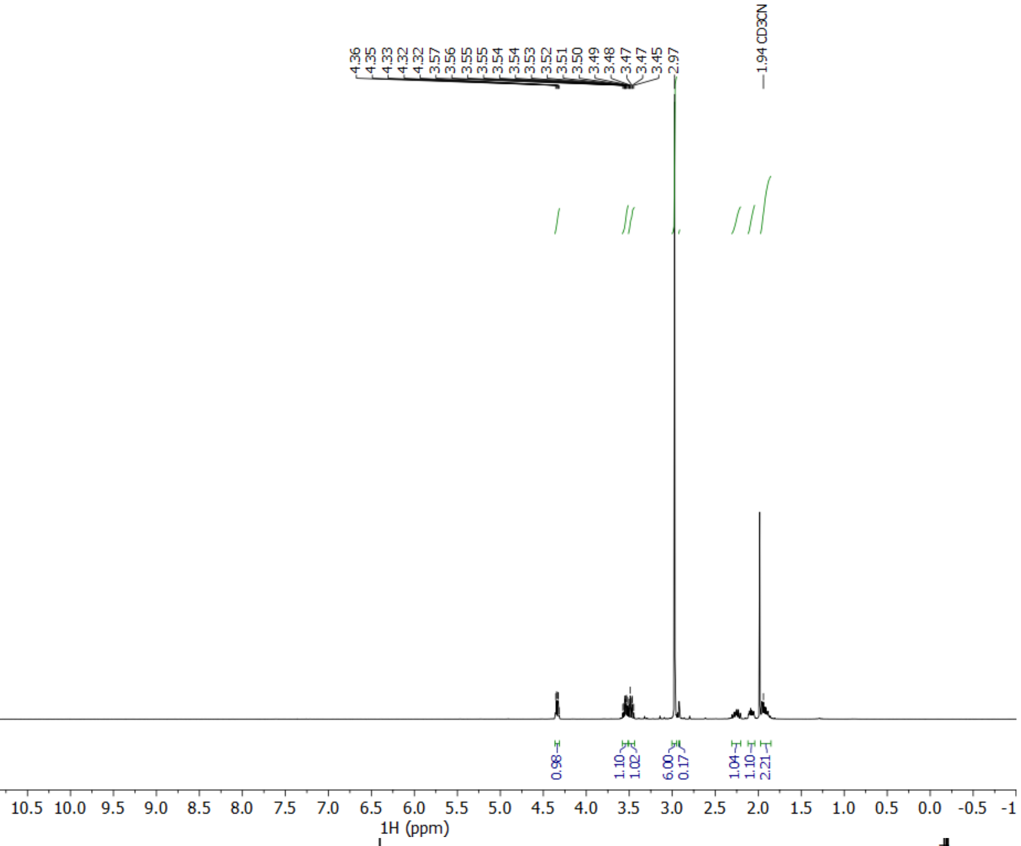

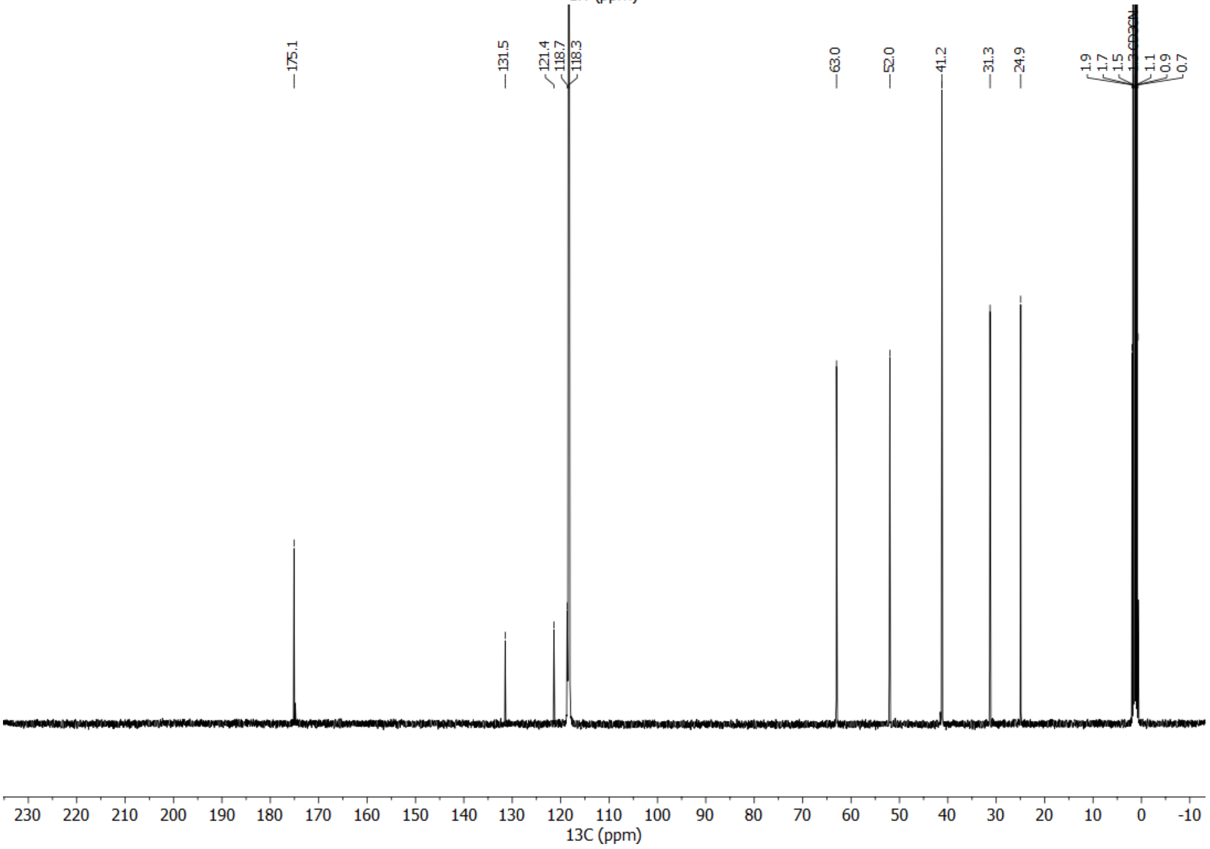

^{1}H NMR, ^{13}C NMR, and ^{19}F NMR spectrum of compound **1c**

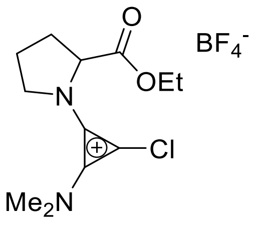

1c

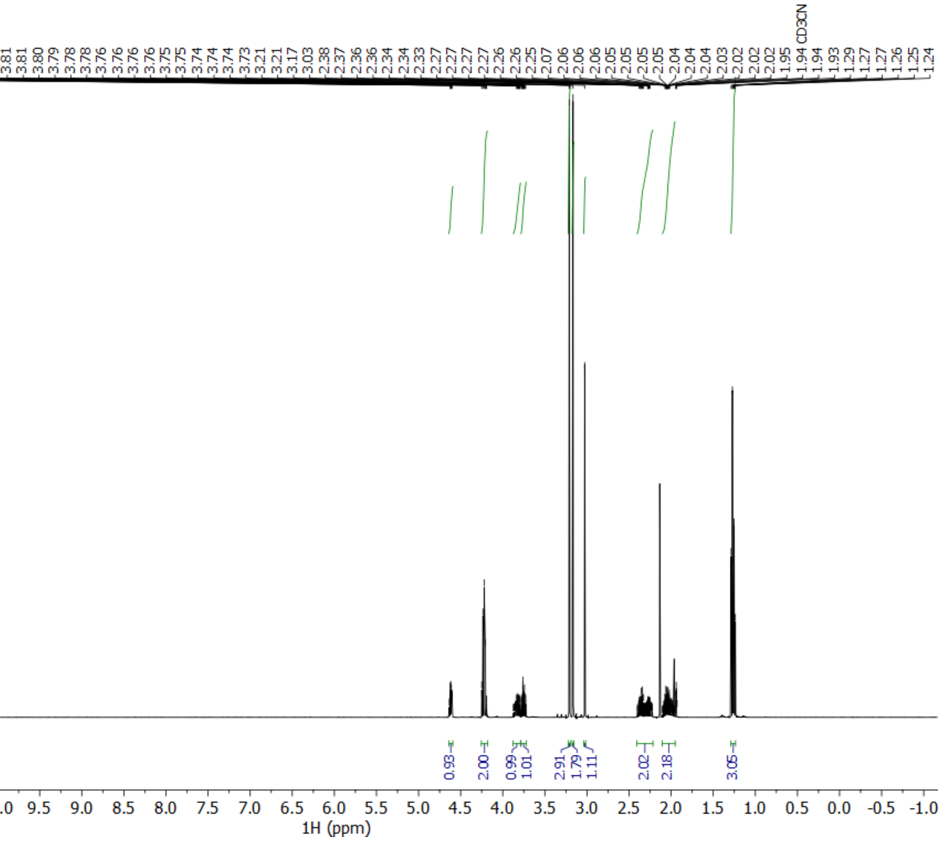

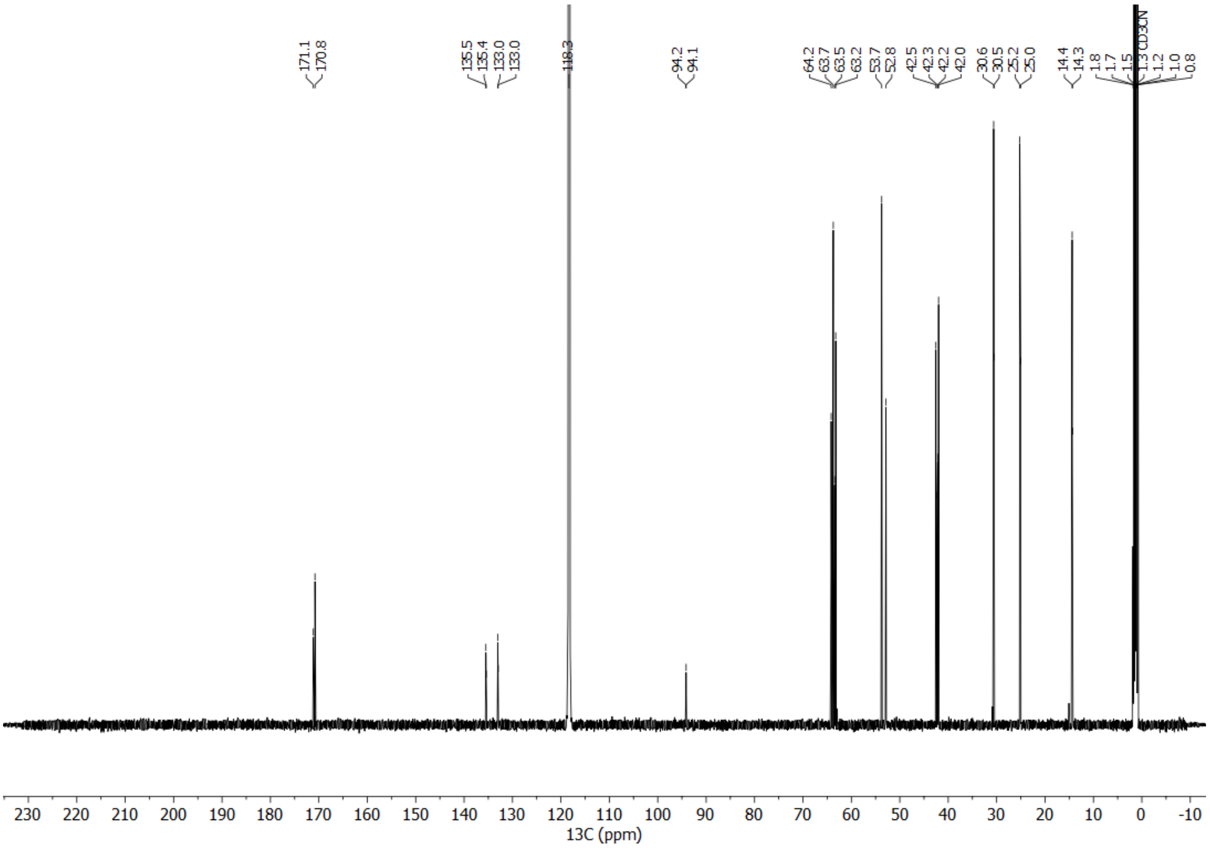

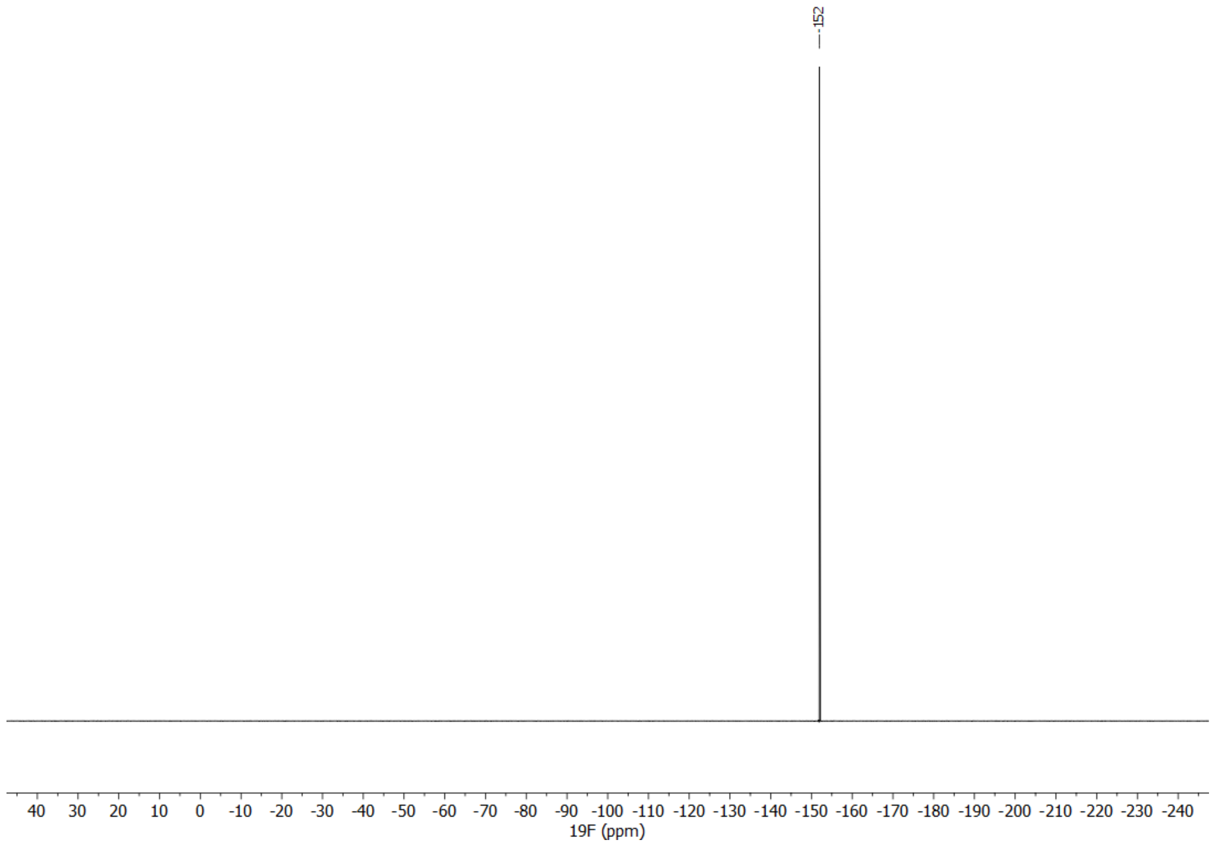
-152
40 30 20 10 0 -10 -20 -30 -40 -50 -60 -70 -80 -90 -100 -110 -120 -130 -140 -150 -160 -170 -180 -190 -200 -210 -220 -230 -240
19F (ppm)

^{1}H NMR, ^{13}C NMR, and ^{19}F NMR spectrum of compound **11a**

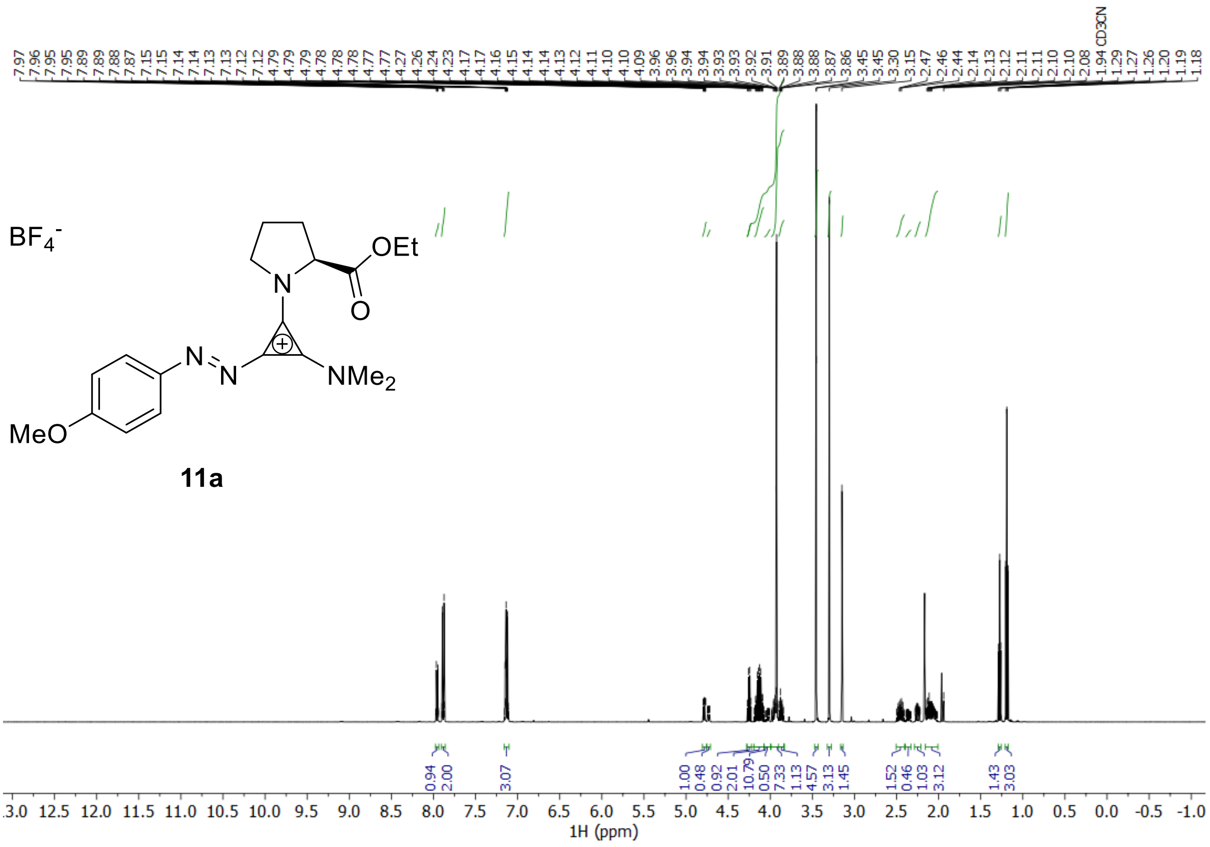

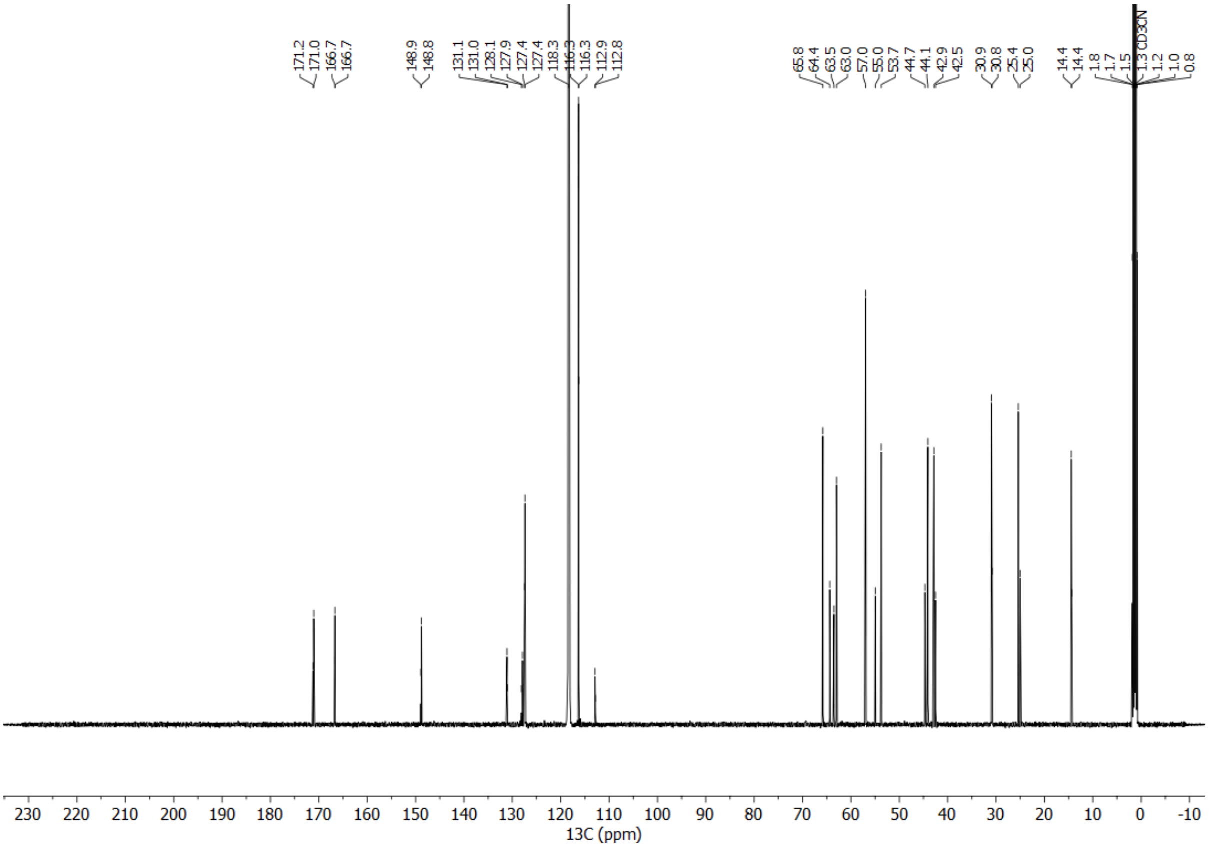

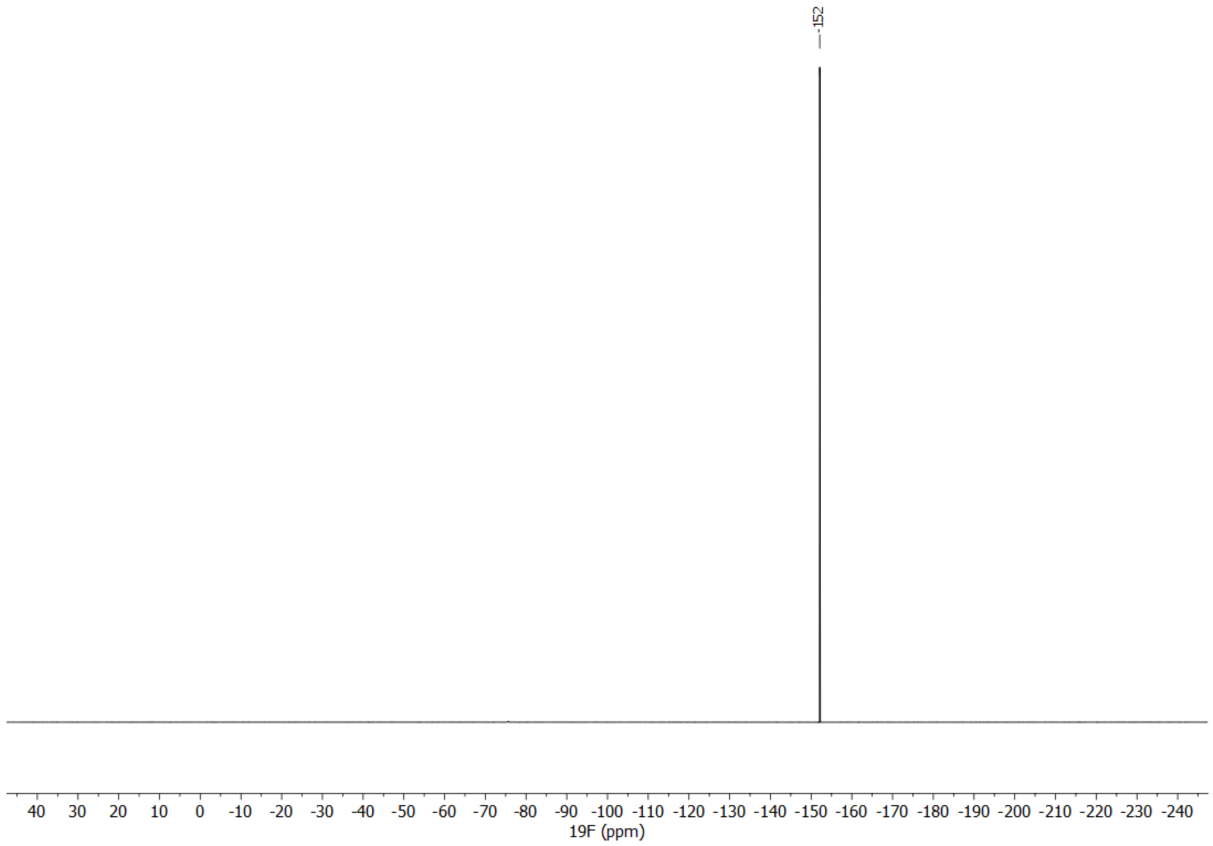
-152
40 30 20 10 0 -10 -20 -30 -40 -50 -60 -70 -80 -90 -100 -110 -120 -130 -140 -150 -160 -170 -180 -190 -200 -210 -220 -230 -240
19F (ppm)

^{1}H NMR, ^{13}C NMR, and ^{19}F NMR spectrum of compound **11b**

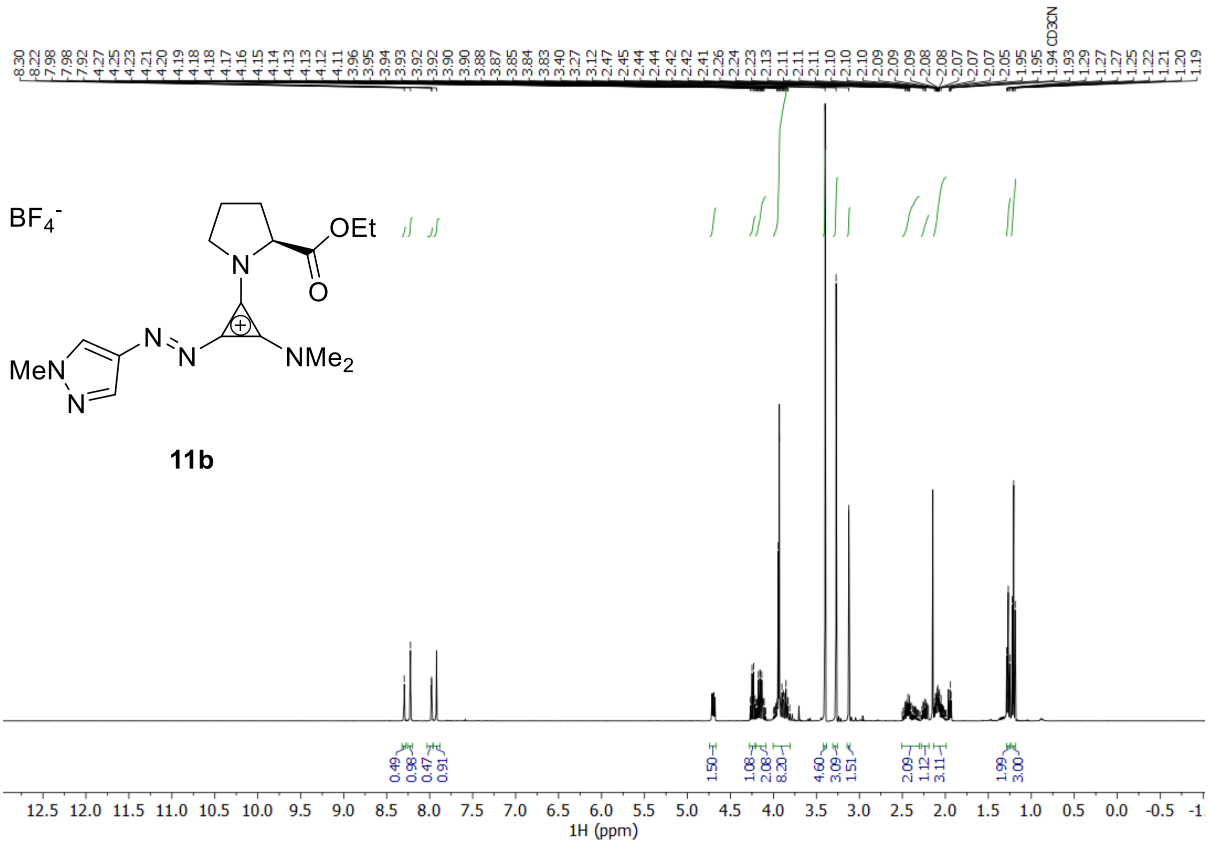

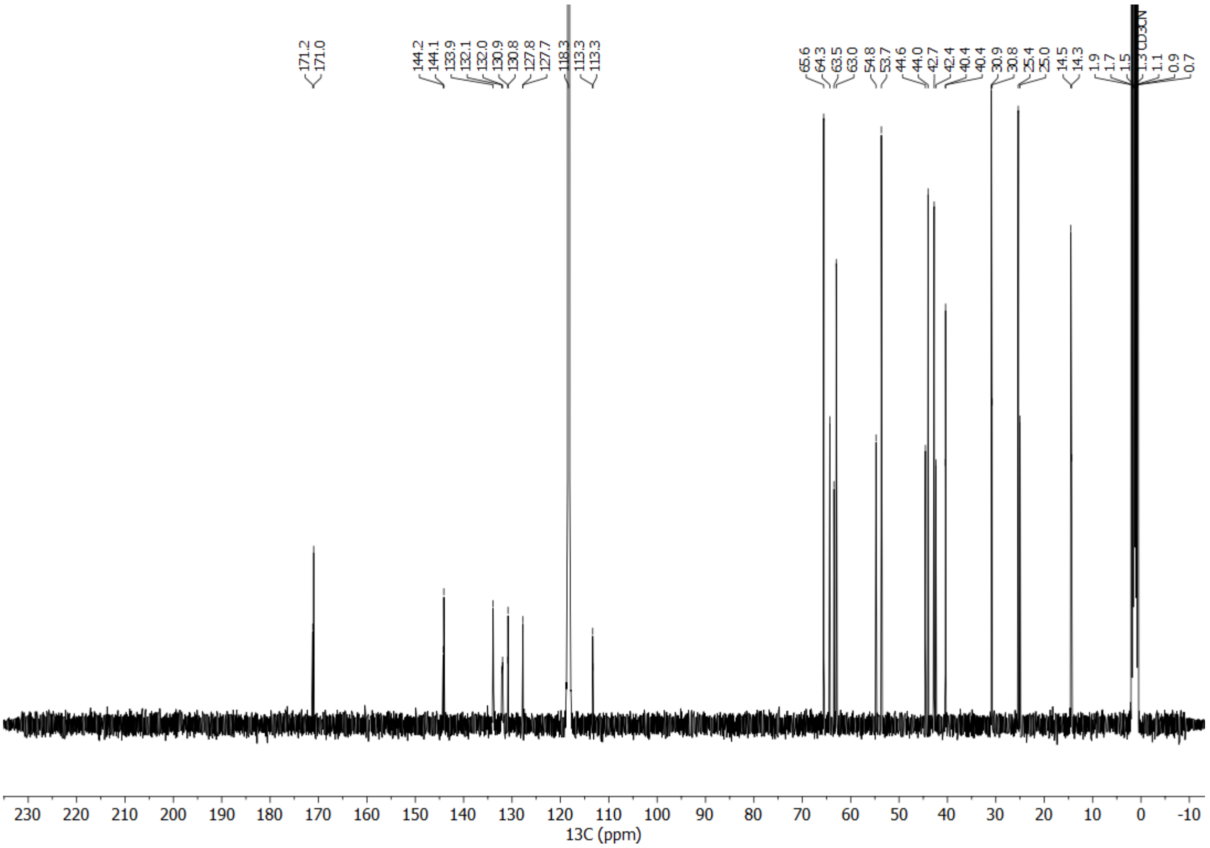

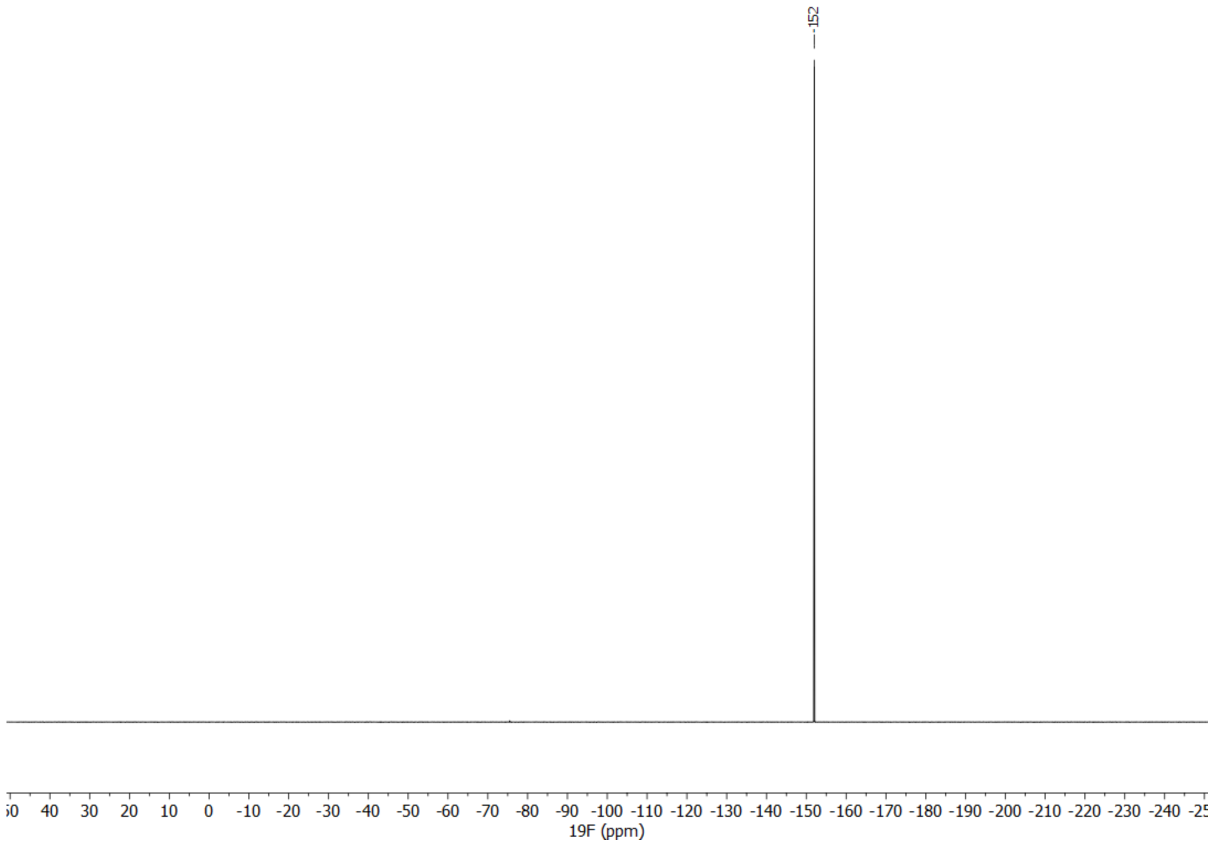
-152
50 40 30 20 10 0 -10 -20 -30 -40 -50 -60 -70 -80 -90 -100 -110 -120 -130 -140 -150 -160 -170 -180 -190 -200 -210 -220 -230 -240 -25
19F (ppm)

HPLC Traces

6a in MeCN 385 nm

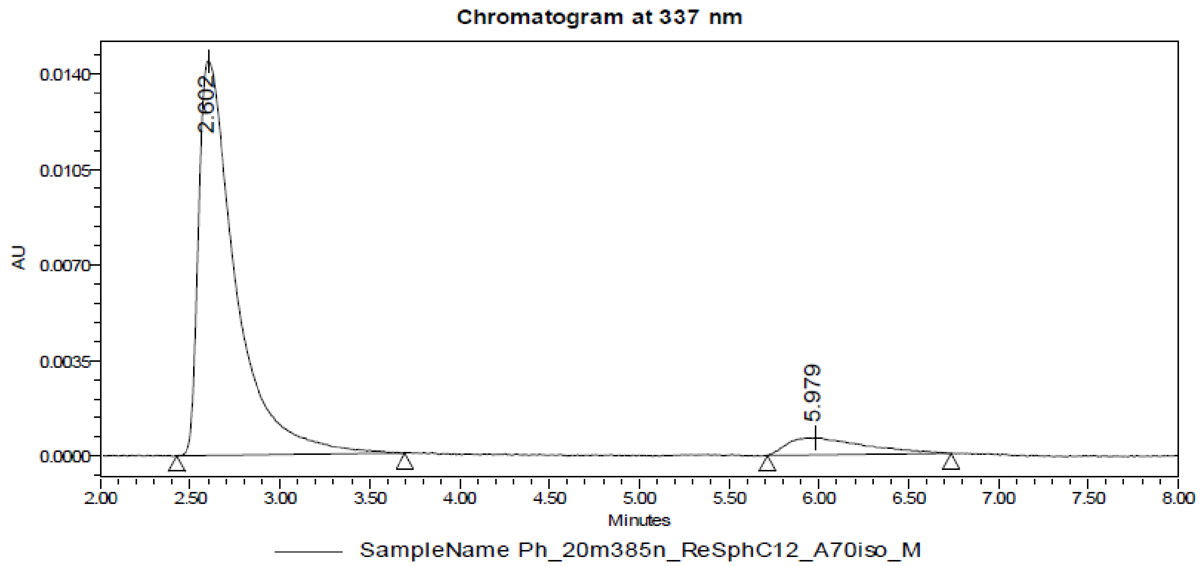

Peak Results

	Name	RT	Height	Area	% Area
1		2.602	14468	193676	90.85
2		5.979	638	19516	9.15

Figure S94: Chromatogram of **6a** after irradiation at 385 nm in MeCN (100 µM) extracted at 337 nm; eluent mixture: MeCN/water (3:7) containing 0.1% formic acid.

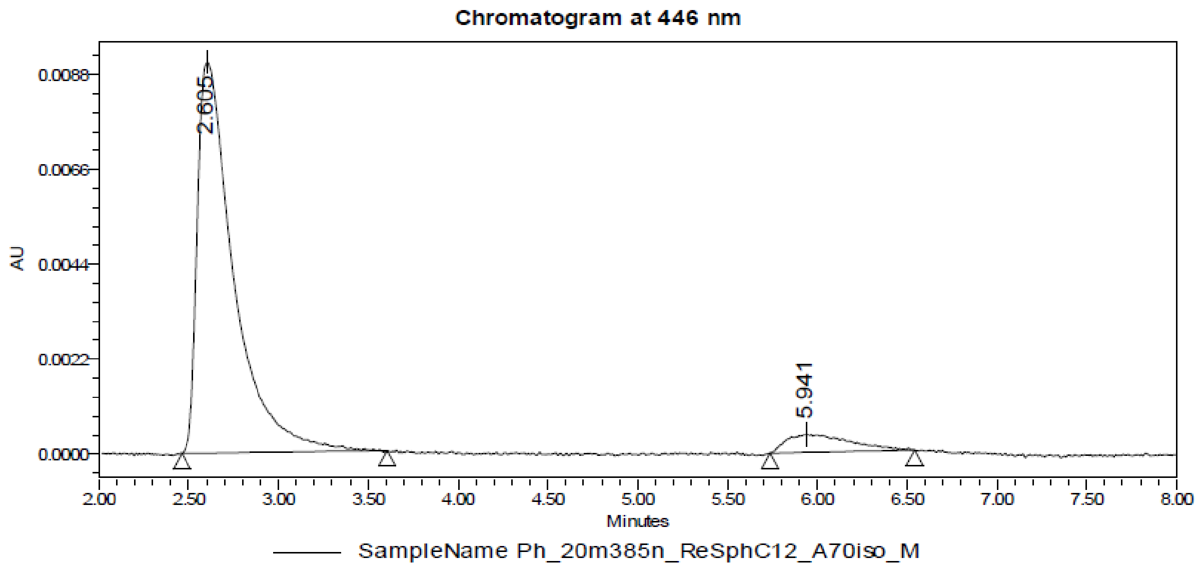

Peak Results

	Name	RT	Height	Area	% Area
1		2.605	9080	119023	92.01
2		5.941	423	10338	7.99

Figure S95: Chromatogram of **6a** after irradiation at 385 nm in MeCN (100 µM) extracted at 446 nm; eluent mixture: MeCN/water (3:7) containing 0.1% formic acid.

505 nm

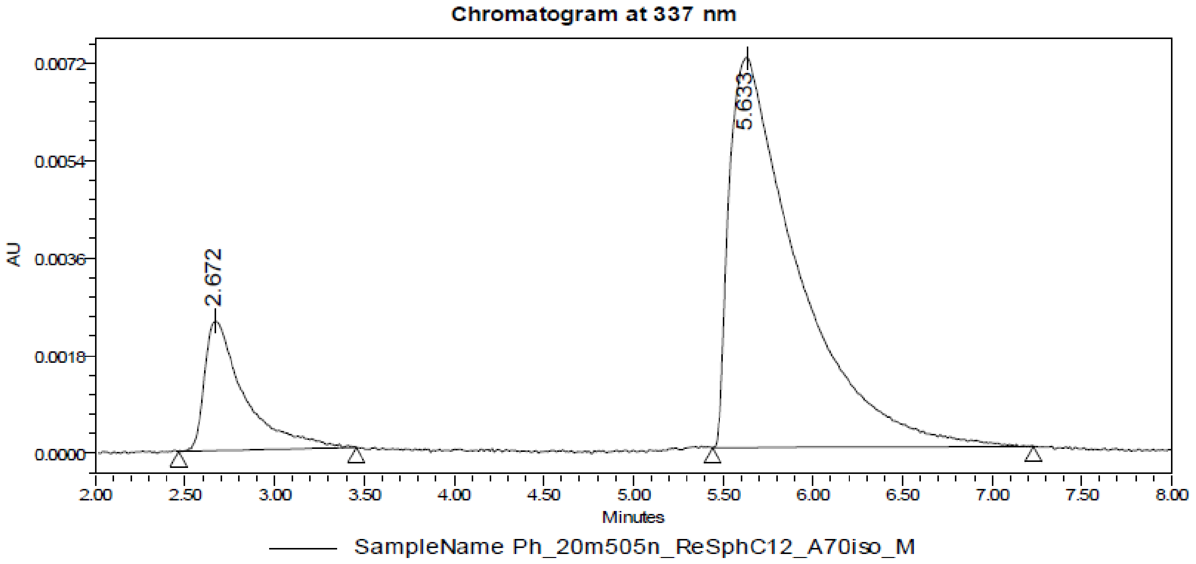

Peak Results

	Name	RT	Height	Area	% Area
1		2.672	2391	25599	11.11
2		5.633	7191	204850	88.89

Figure S96: Chromatogram of **6a** after irradiation at 505 nm in MeCN (100 µM) extracted at 337 nm; eluent mixture: MeCN/water (3:7) containing 0.1% formic acid.

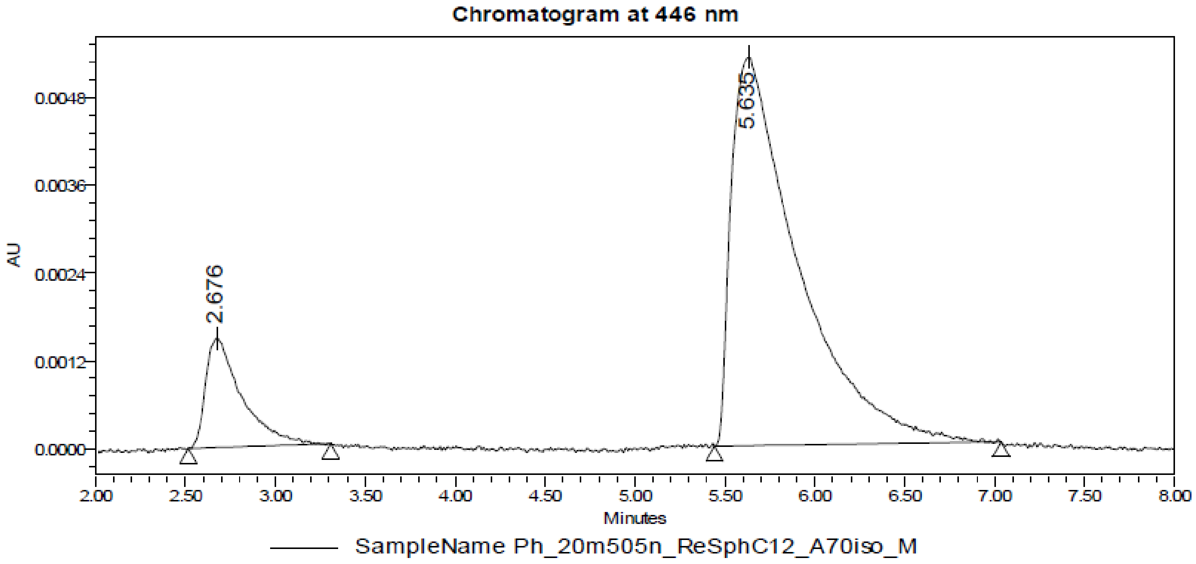

Peak Results

	Name	RT	Height	Area	% Area
1		2.676	1492	14439	8.96
2		5.635	5310	146772	91.04

Figure S97: Chromatogram of **6a** after irradiation at 505 nm in MeCN (100 µM) extracted at 446 nm; eluent mixture: MeCN/water (3:7) containing 0.1% formic acid.

6a in water

385 nm

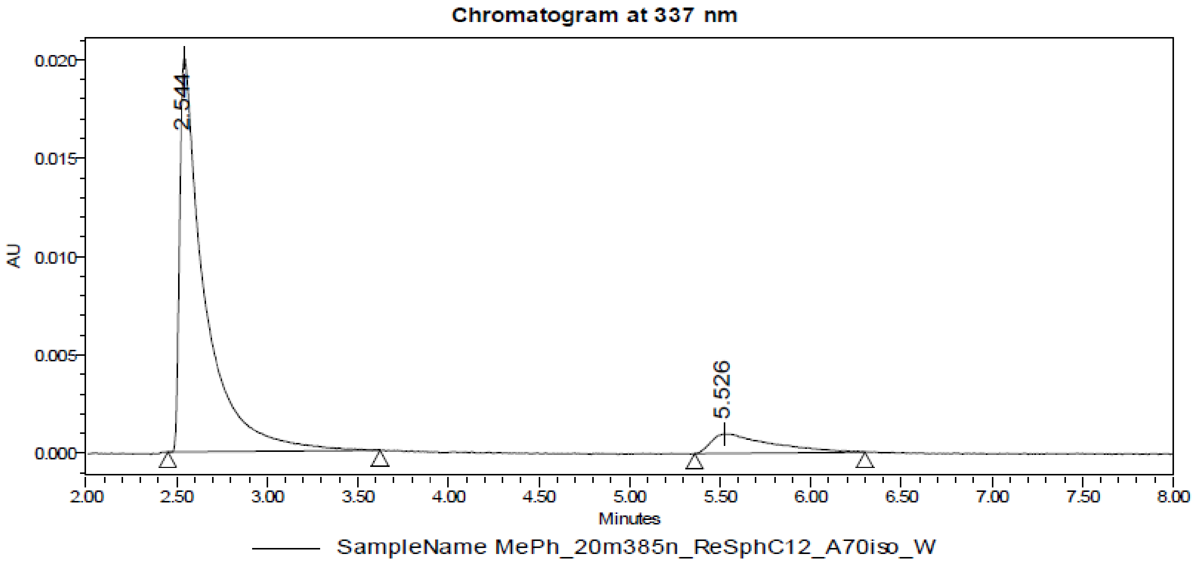

Peak Results

	Name	RT	Height	Area	% Area
1		2.544	20036	192536	89.72
2		5.526	976	22068	10.28

Figure S98: Chromatogram of **6a** after irradiation at 385 nm in water (100 µM) extracted at 337 nm; eluent mixture: MeCN/water (3:7) containing 0.1% formic acid.

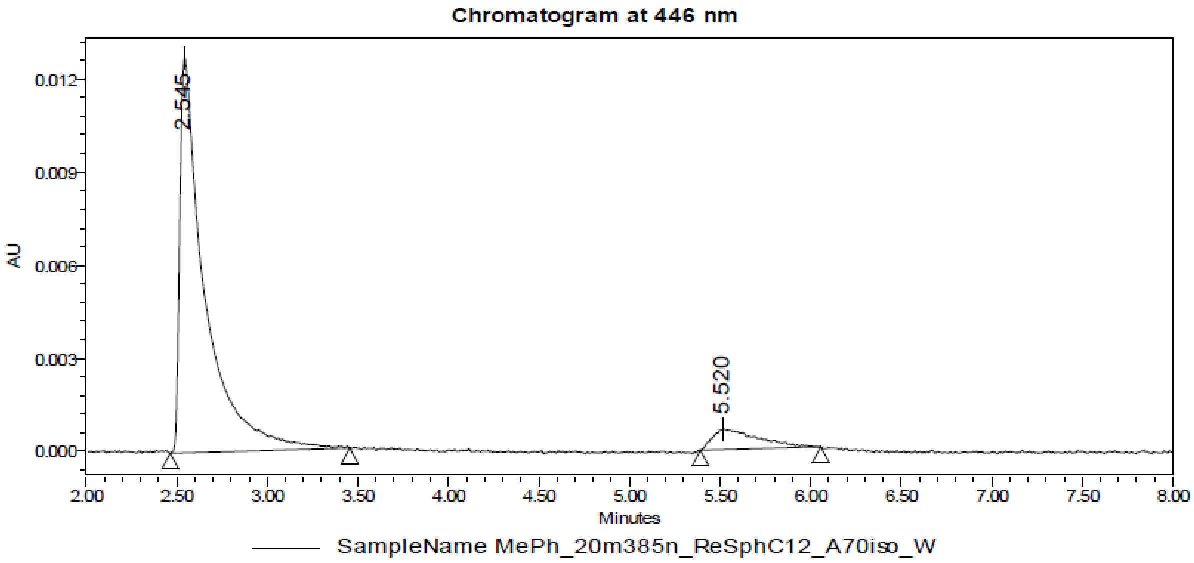

Peak Results

	Name	RT	Height	Area	% Area
1		2.545	12734	122769	91.35
2		5.520	661	11632	8.65

Figure S99: Chromatogram of **6a** after irradiation at 385 nm in water (100 µM) extracted at 446 nm; eluent mixture: MeCN/water (3:7) containing 0.1% formic acid.

505 nm

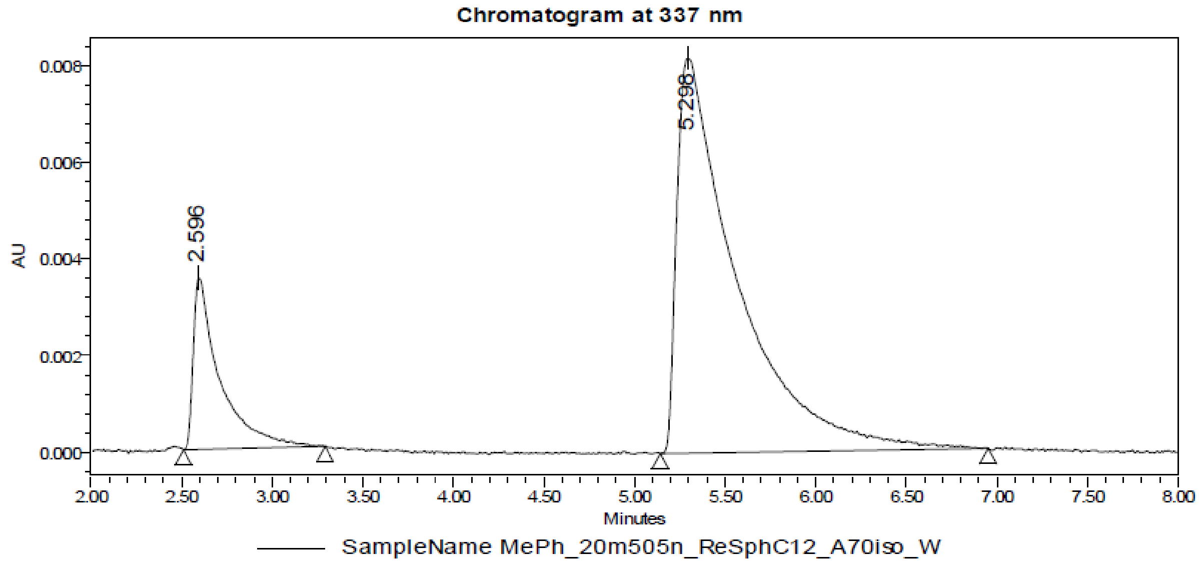

Peak Results

	Name	RT	Height	Area	% Area
1		2.596	3544	35810	16.01
2		5.298	8174	187806	83.99

Figure S100: Chromatogram of **6a** after irradiation at 505 nm in water (100 µM) extracted at 337 nm; eluent mixture: MeCN/water (3:7) containing 0.1% formic acid.

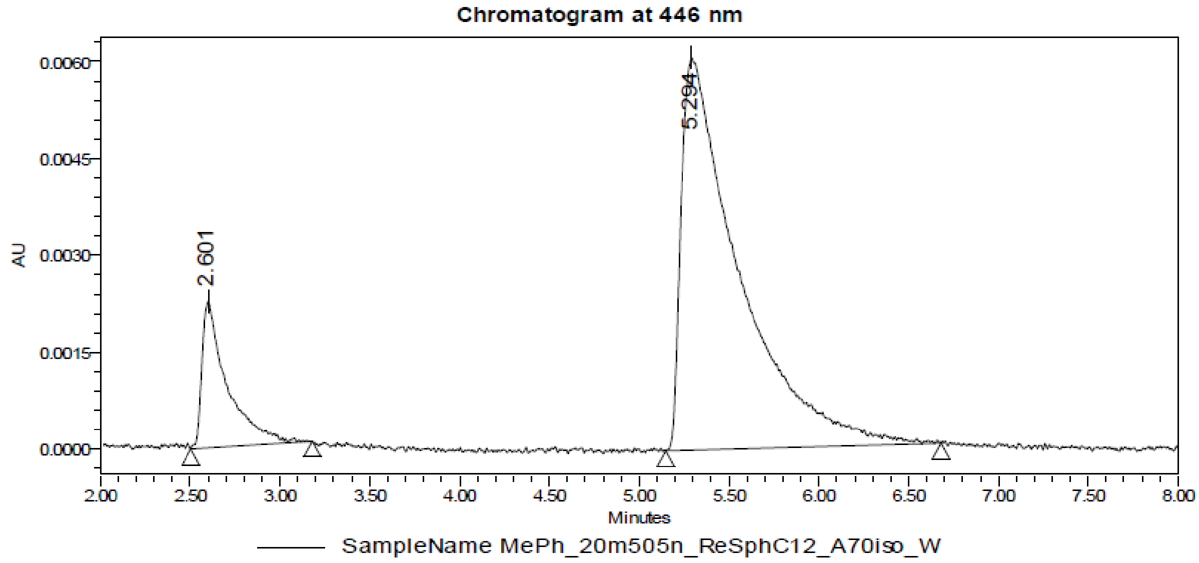

Peak Results

	Name	RT	Height	Area	% Area
1		2.601	2268	22082	13.95
2		5.294	6083	136222	86.05

Figure S101: Chromatogram of **6a** after irradiation at 505 nm in water (100 µM) extracted at 446 nm; eluent mixture: MeCN/water (3:7) containing 0.1% formic acid.

6c in MeCN

385 nm

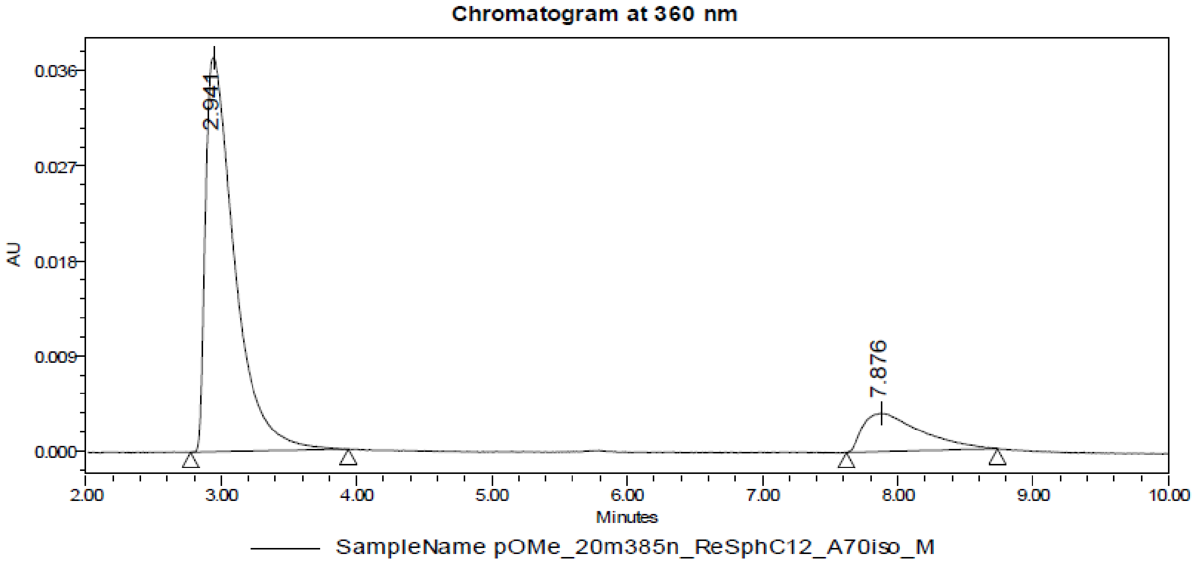

Peak Results

	Name	RT	Height	Area	% Area
1		2.941	37303	581563	83.06
2		7.876	3636	118604	16.94

Figure S102: Chromatogram of **6c** after irradiation at 385 nm in MeCN (100 µM) extracted at 360 nm; eluent mixture: MeCN/water (3:7) containing 0.1% formic acid.

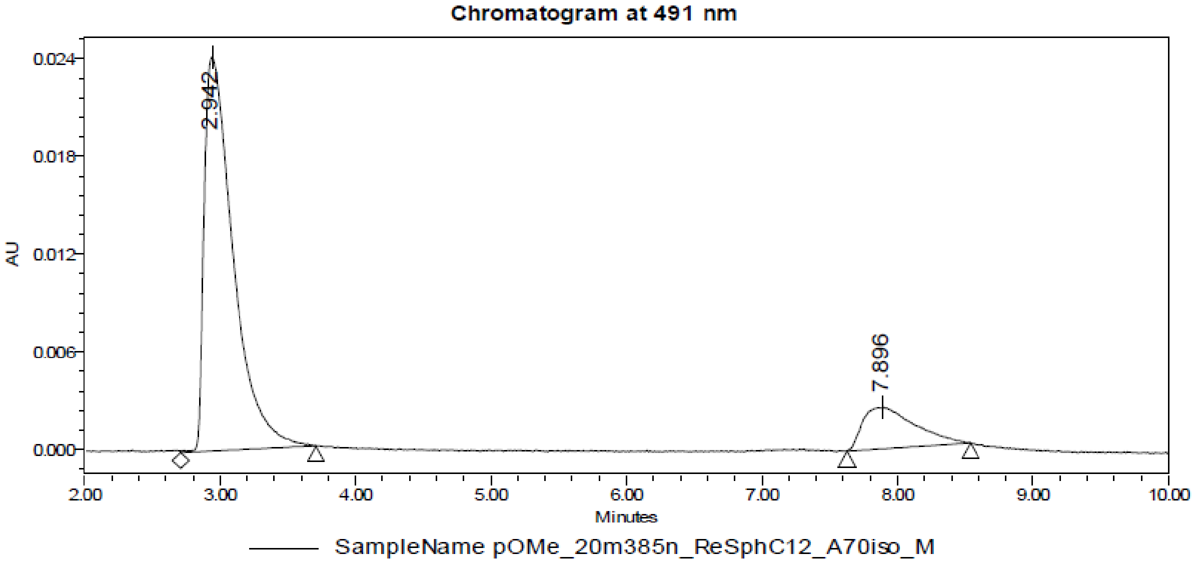

Peak Results

	Name	RT	Height	Area	% Area
1		2.942	24188	374235	83.14
2		7.896	2559	75892	16.86

Figure S103: Chromatogram of **6c** after irradiation at 385 nm in MeCN (100 µM) extracted at 491 nm; eluent mixture: MeCN/water (3:7) containing 0.1% formic acid.

415 nm

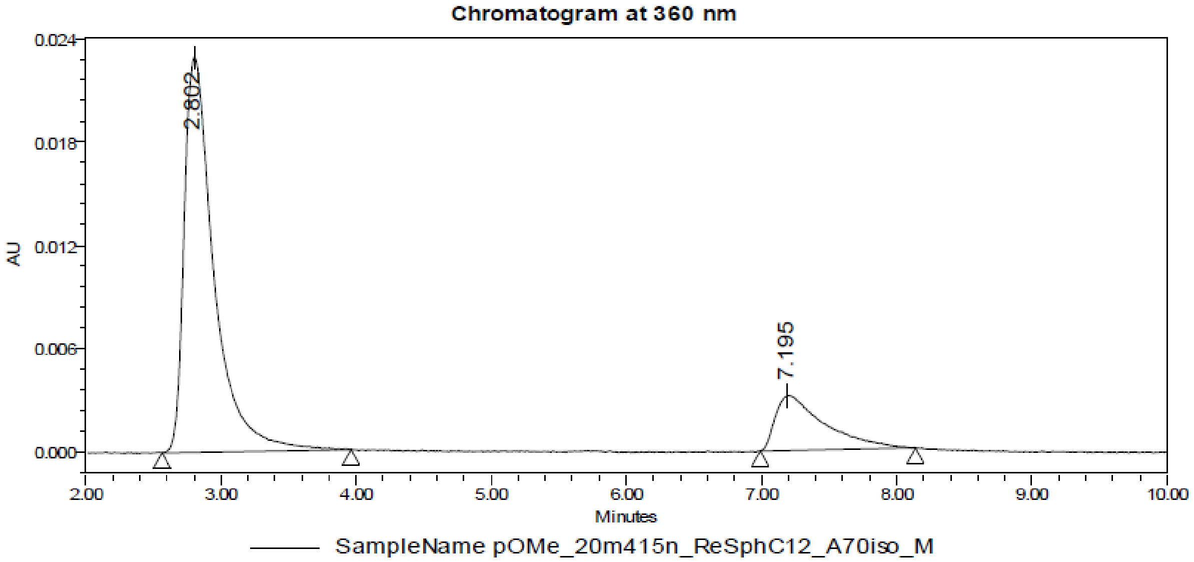

Peak Results

	Name	RT	Height	Area	% Area
1		2.802	22898	352165	81.58
2		7.195	3171	79504	18.42

Figure S104: Chromatogram of **6c** after irradiation at 415 nm in MeCN (100 µM) extracted at 360 nm; eluent mixture: MeCN/water (3:7) containing 0.1% formic acid.

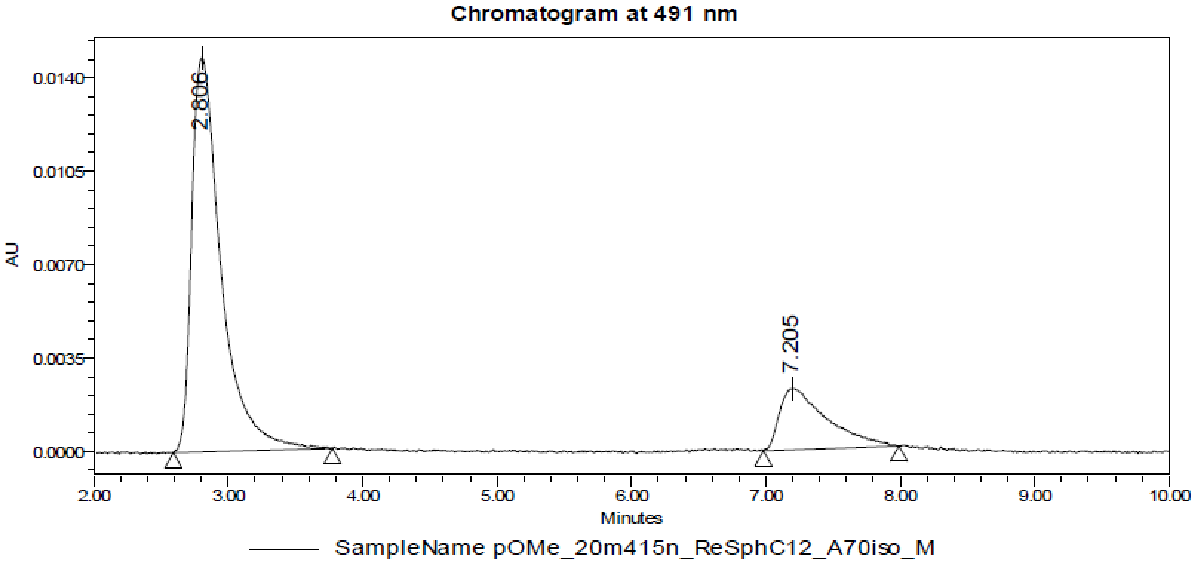

Peak Results

	Name	RT	Height	Area	% Area
1		2.806	14787	225145	80.18
2		7.205	2293	55649	19.82

Figure S105: Chromatogram of **6c** after irradiation at 415 nm in MeCN (100 µM) extracted at 491 nm; eluent mixture: MeCN/water (3:7) containing 0.1% formic acid.

505 nm

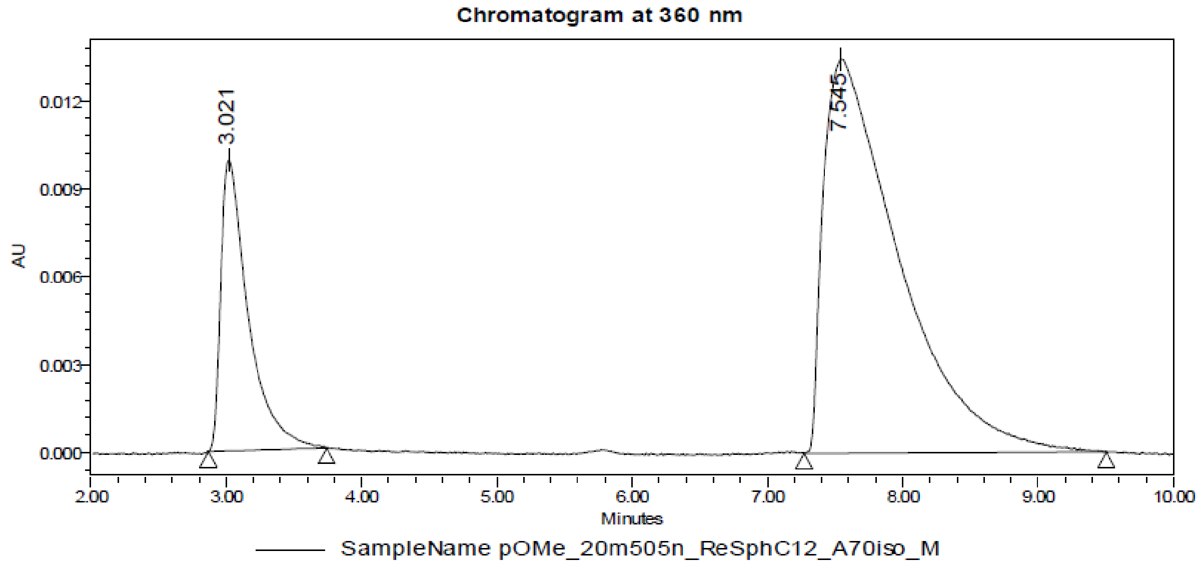

Peak Results

	Name	RT	Height	Area	% Area
1		3.021	9922	139350	22.10
2		7.545	13446	491157	77.90

Figure S106: Chromatogram of **6c** after irradiation at 505 nm in MeCN (100 μM) extracted at 360 nm; eluent mixture: MeCN/water (3:7) containing 0.1% formic acid.

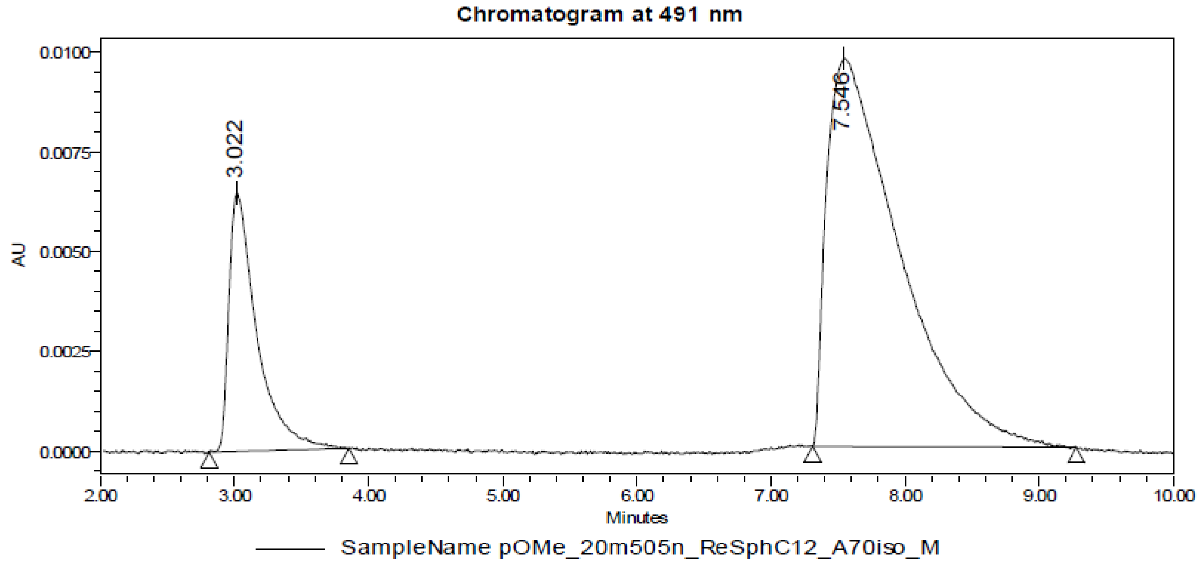

Peak Results

	Name	RT	Height	Area	% Area
1		3.022	6468	92208	21.01
2		7.546	9734	346610	78.99

Figure S107: Chromatogram of **6c** after irradiation at 505 nm in MeCN (100 μM) extracted at 491 nm; eluent mixture: MeCN/water (3:7) containing 0.1% formic acid.

530 nm

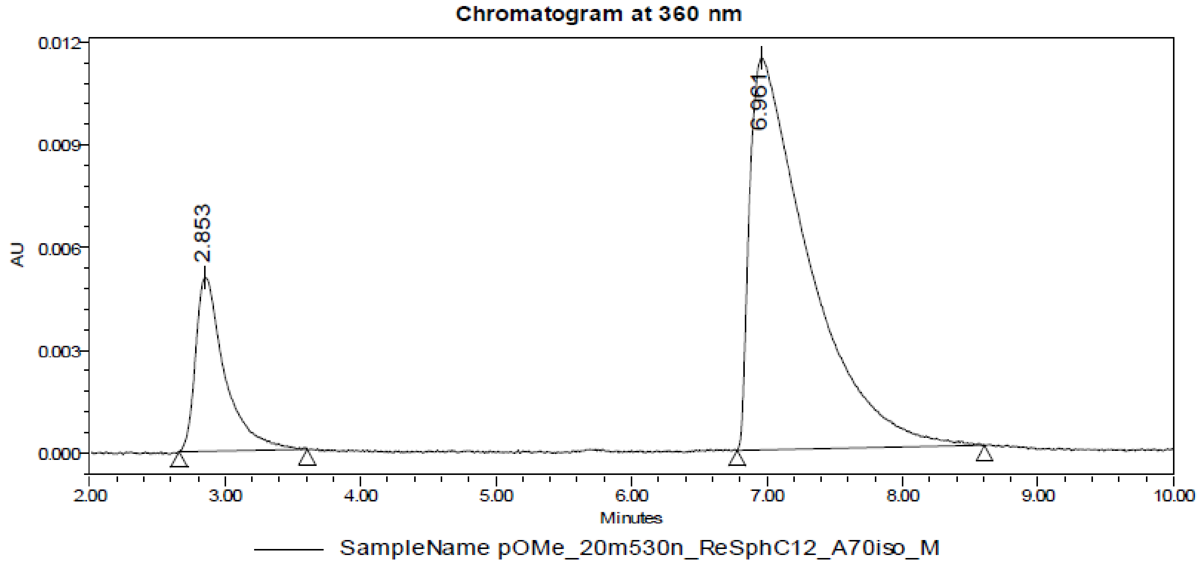

Peak Results

	Name	RT	Height	Area	% Area
1		2.853	5081	76473	17.94
2		6.961	11470	349713	82.06

Figure S108: Chromatogram of **6c** after irradiation at 530 nm in MeCN (100 µM) extracted at 360 nm; eluent mixture: MeCN/water (3:7) containing 0.1% formic acid.

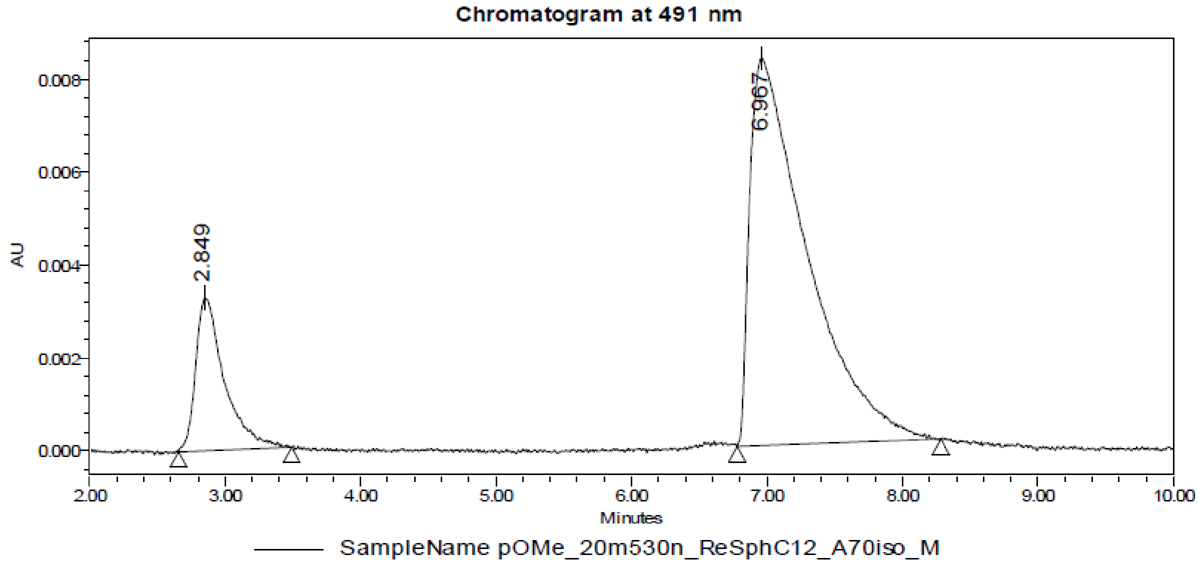

Peak Results

	Name	RT	Height	Area	% Area
1		2.849	3302	48857	16.56
2		6.967	8342	246154	83.44

Figure S109: Chromatogram of **6c** after irradiation at 530 nm in MeCN (100 µM) extracted at 491 nm; eluent mixture: MeCN/water (3:7) containing 0.1% formic acid.

6c in water

385 nm

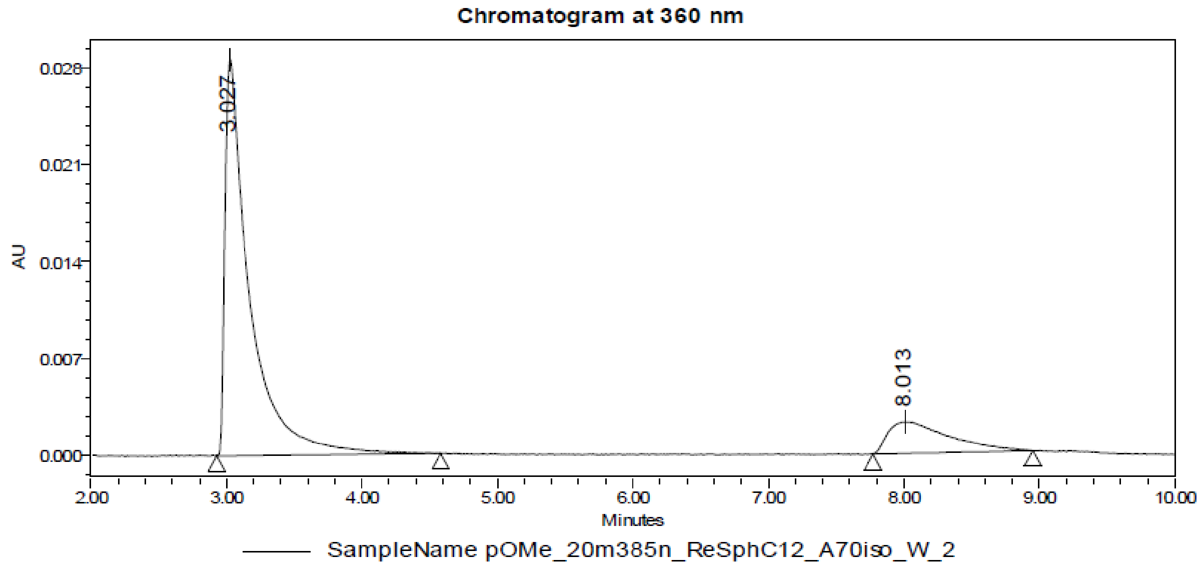

Peak Results

	Name	RT	Height	Area	% Area
1		3.027	28639	332767	81.81
2		8.013	2286	73977	18.19

Figure S110: Chromatogram of **6c** after irradiation at 385 nm in water (100 µM) extracted at 360 nm; eluent mixture: MeCN/water (3:7) containing 0.1% formic acid.

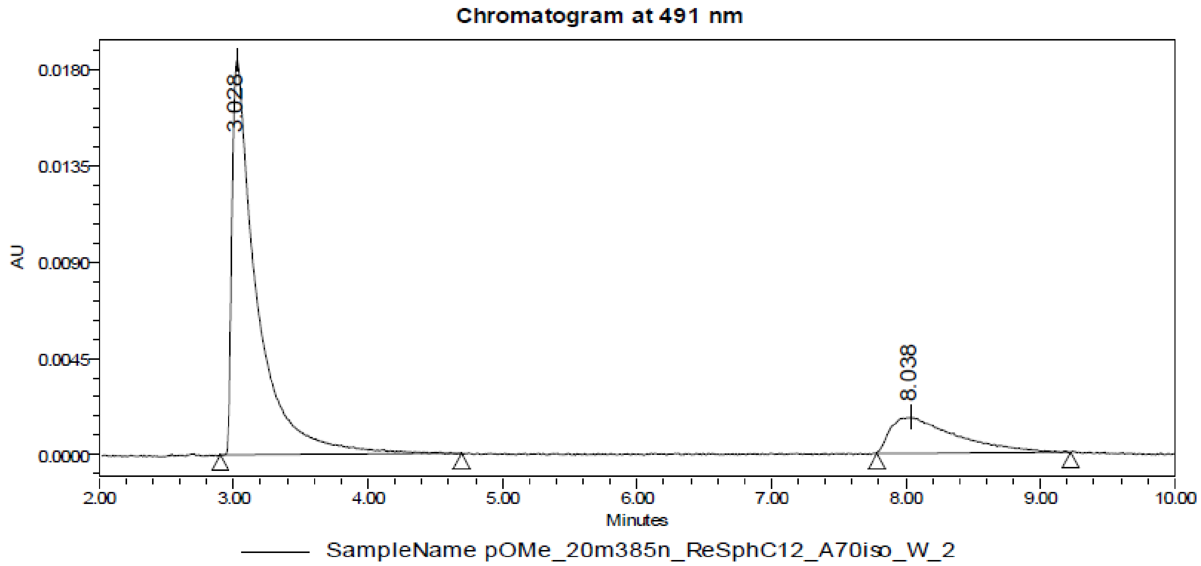

Peak Results

	Name	RT	Height	Area	% Area
1		3.028	18470	215310	78.72
2		8.038	1679	58202	21.28

Figure S111: Chromatogram of **6c** after irradiation at 385 nm in water (100 µM) extracted at 491 nm; eluent mixture: MeCN/water (3:7) containing 0.1% formic acid.

415 nm

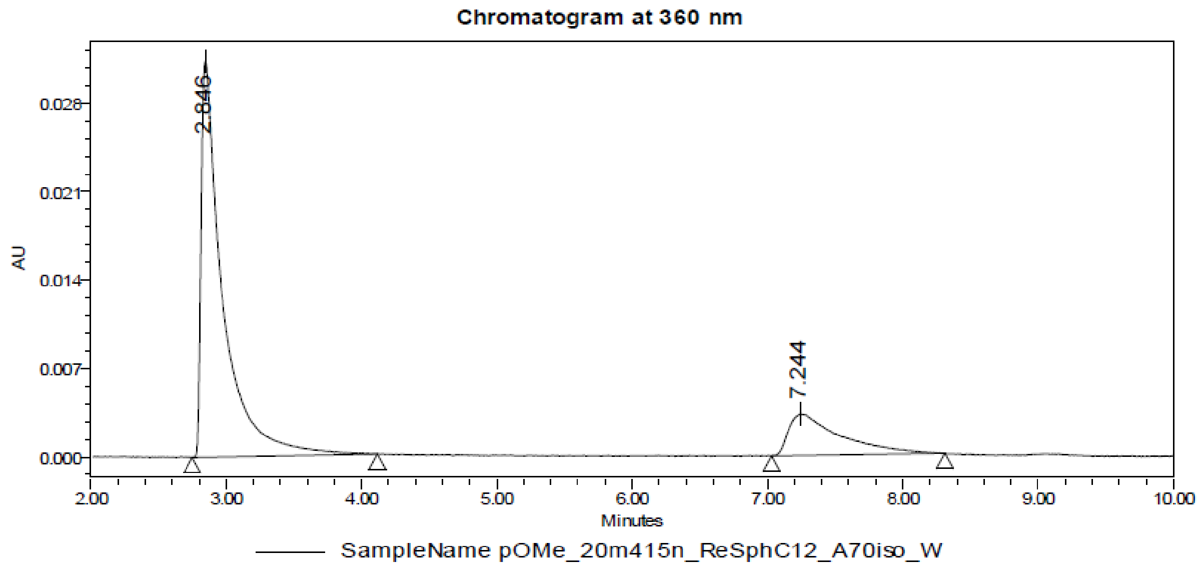

Peak Results

	Name	RT	Height	Area	% Area
1		2.846	31351	346024	80.02
2		7.244	3277	86394	19.98

Figure S112: Chromatogram of **6c** after irradiation at 415 nm in water (100 µM) extracted at 360 nm; eluent mixture: MeCN/water (3:7) containing 0.1% formic acid.

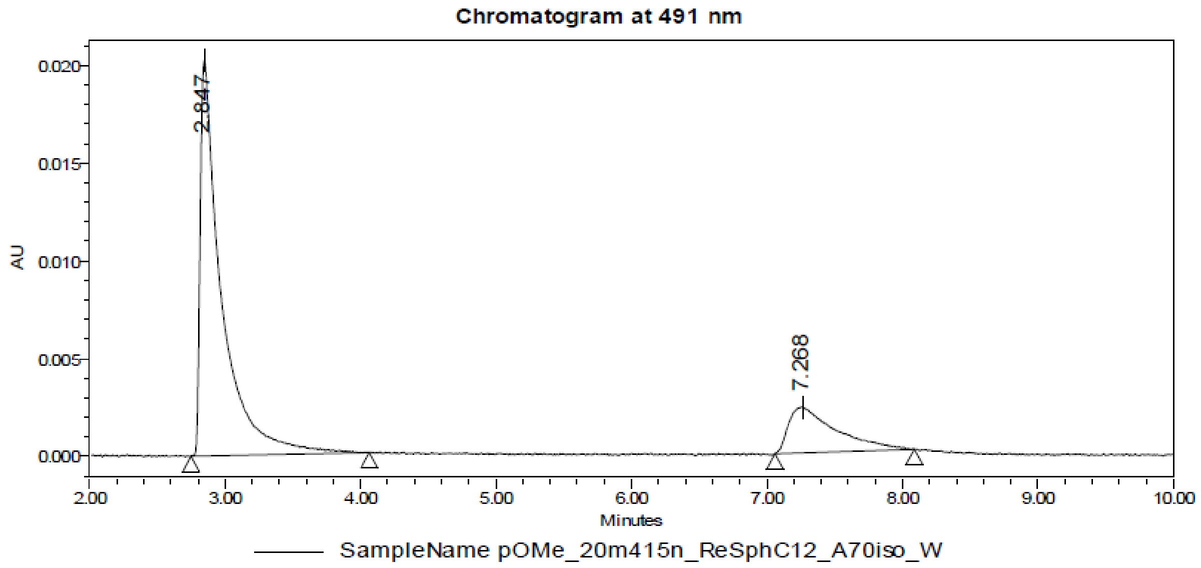

Peak Results

	Name	RT	Height	Area	% Area
1		2.847	20266	223750	79.63
2		7.268	2356	57222	20.37

Figure S113: Chromatogram of **6c** after irradiation at 415 nm in water (100 µM) extracted at 491 nm; eluent mixture: MeCN/water (3:7) containing 0.1% formic acid.

505 nm

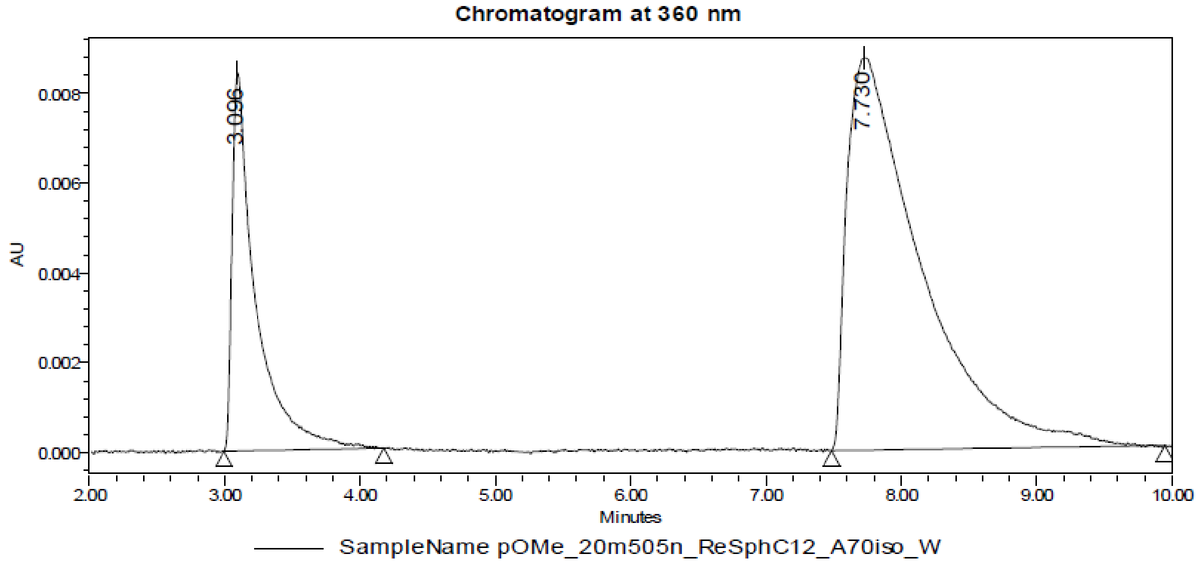

Peak Results

	Name	RT	Height	Area	% Area
1		3.096	8422	98667	22.30
2		7.730	8733	343806	77.70

Figure S114: Chromatogram of **6c** after irradiation at 505 nm in water (100 µM) extracted at 360 nm; eluent mixture: MeCN/water (3:7) containing 0.1% formic acid.

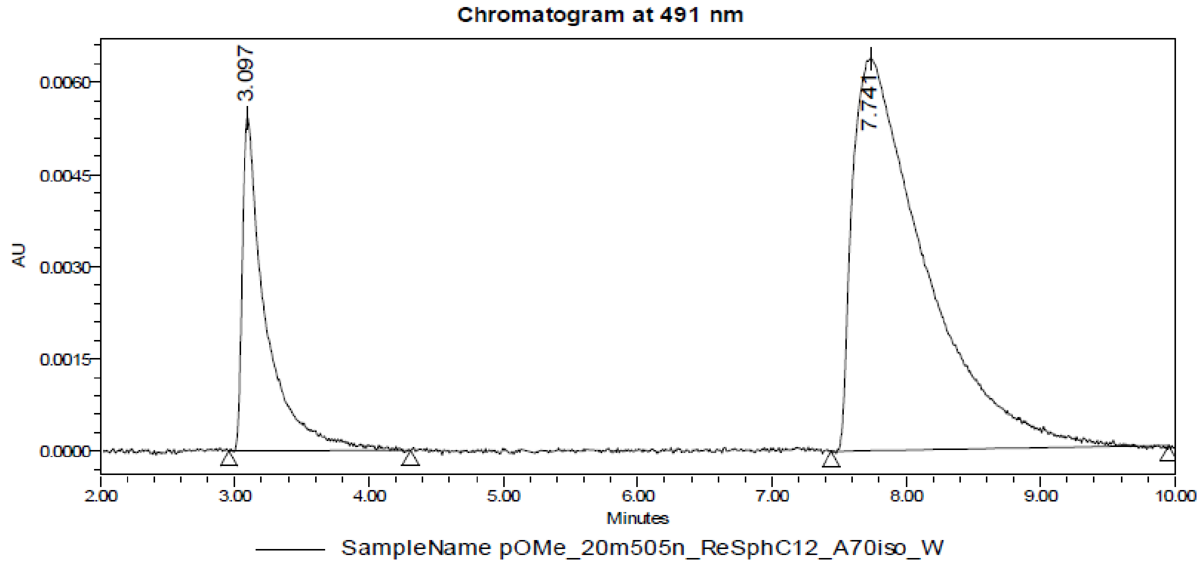

Peak Results

	Name	RT	Height	Area	% Area
1		3.097	5429	64856	20.77
2		7.741	6376	247375	79.23

Figure S115: Chromatogram of **6c** after irradiation at 505 nm in water (100 µM) extracted at 491 nm; eluent mixture: MeCN/water (3:7) containing 0.1% formic acid.

530 nm

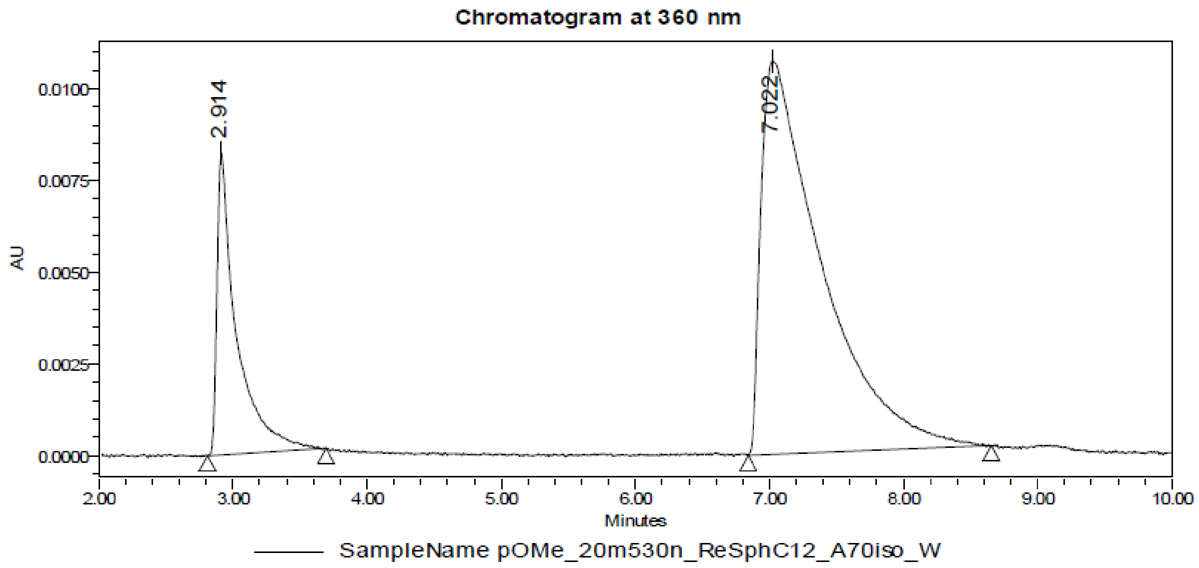

Peak Results

	Name	RT	Height	Area	% Area
1		2.914	8234	84200	20.01
2		7.022	10720	336518	79.99

Figure S116: Chromatogram of **6c** after irradiation at 530 nm in water (100 µM) extracted at 360 nm; eluent mixture: MeCN/water (3:7) containing 0.1% formic acid.

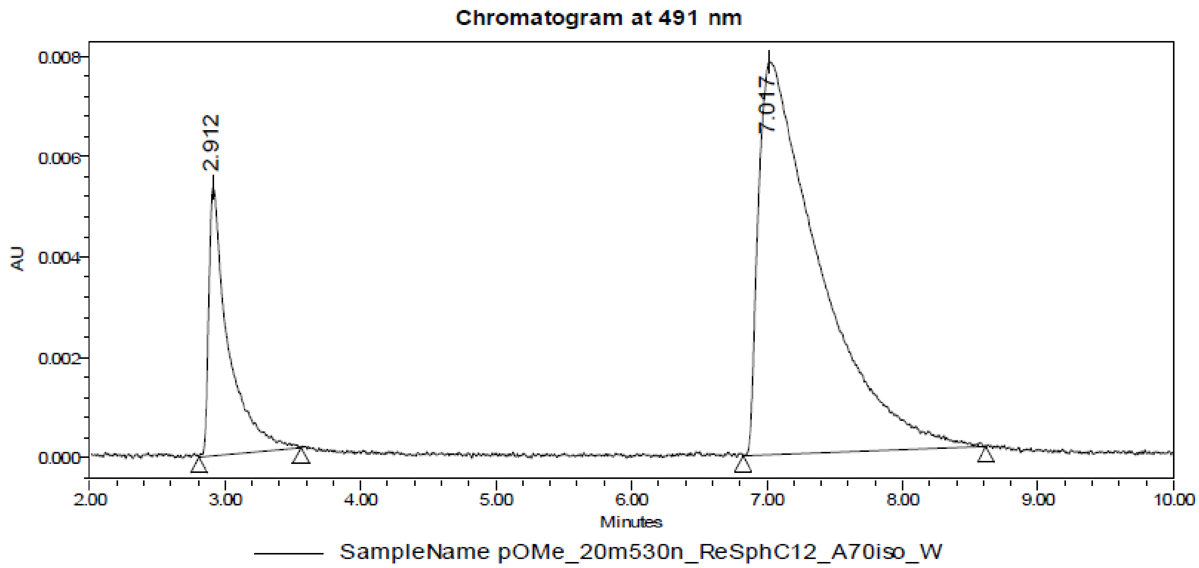

Peak Results

	Name	RT	Height	Area	% Area
1		2.912	5361	54123	17.99
2		7.017	7850	246777	82.01

Figure S117: Chromatogram of **6c** after irradiation at 530 nm in water (100 µM) extracted at 491 nm; eluent mixture: MeCN/water (3:7) containing 0.1% formic acid.

6e in water

385 nm

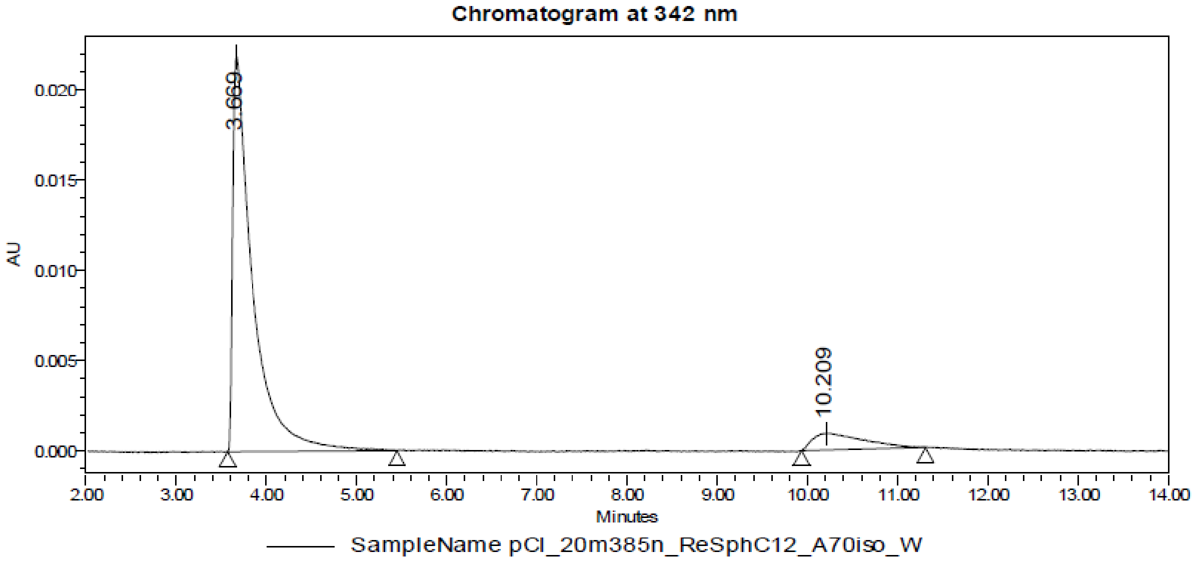

Peak Results

	Name	RT	Height	Area	% Area
1		3.669	21874	332865	90.44
2		10.209	914	35198	9.56

Figure S118: Chromatogram of **6e** after irradiation at 385 nm in water (100 µM) extracted at 342 nm; eluent mixture: MeCN/water (3:7) containing 0.1% formic acid.

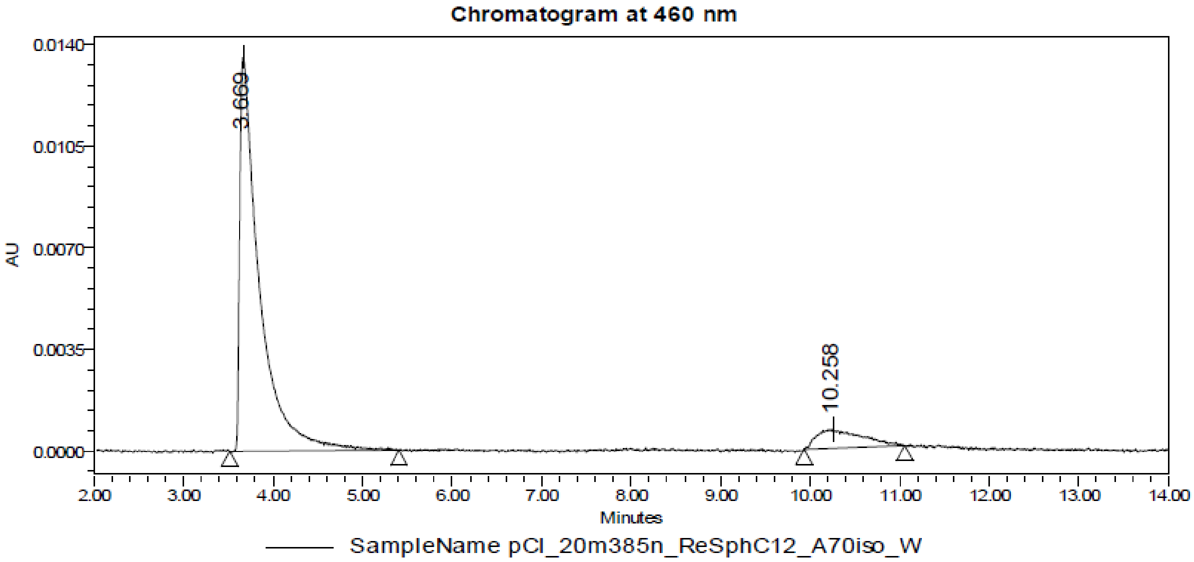

Peak Results

	Name	RT	Height	Area	% Area
1		3.669	13588	207600	90.18
2		10.258	658	22612	9.82

Figure S119: Chromatogram of **6e** after irradiation at 385 nm in water (100 µM) extracted at 460 nm; eluent mixture: MeCN/water (3:7) containing 0.1% formic acid.

505 nm

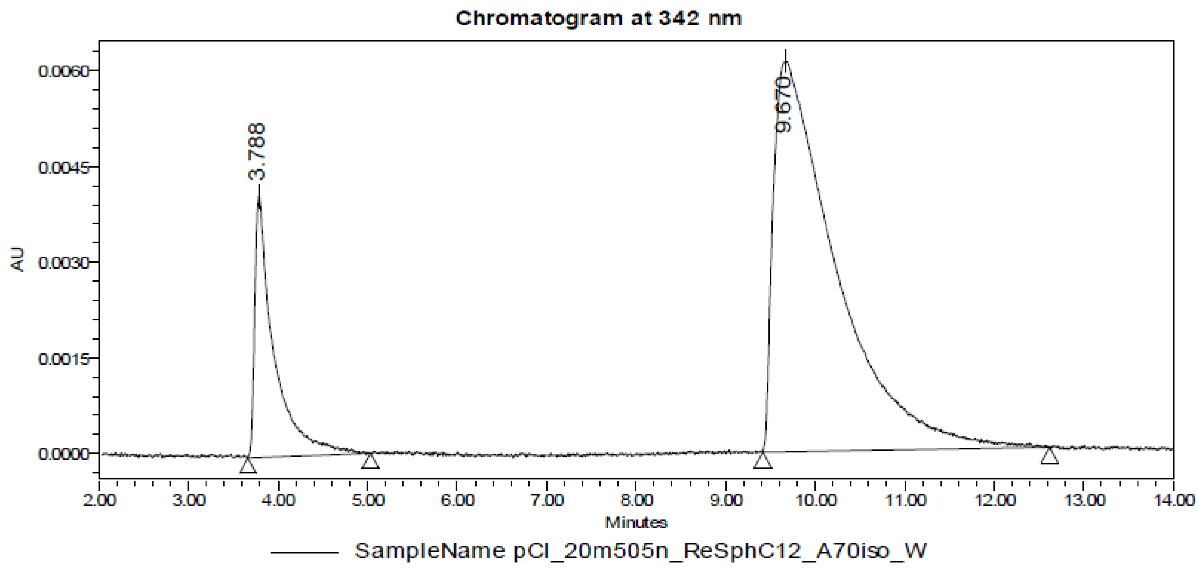

Peak Results

	Name	RT	Height	Area	% Area
1		3.788	4094	60724	16.96
2		9.670	6121	297294	83.04

Figure S120: Chromatogram of **6e** after irradiation at 505 nm in water (100 µM) extracted at 342 nm; eluent mixture: MeCN/water (3:7) containing 0.1% formic acid.

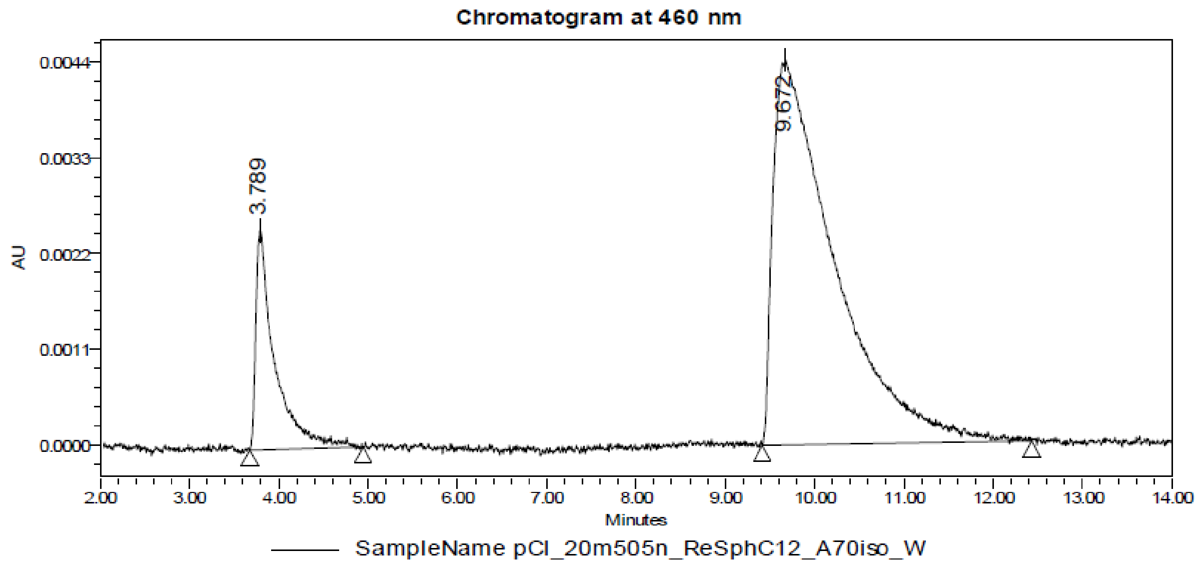

Peak Results

	Name	RT	Height	Area	% Area
1		3.789	2516	37086	14.92
2		9.672	4426	21145[illegible]	85.08

Figure S121: Chromatogram of **6e** after irradiation at 505 nm in water (100 µM) extracted at 460 nm; eluent mixture: MeCN/water (3:7) containing 0.1% formic acid.

6f in MeCN

385 nm

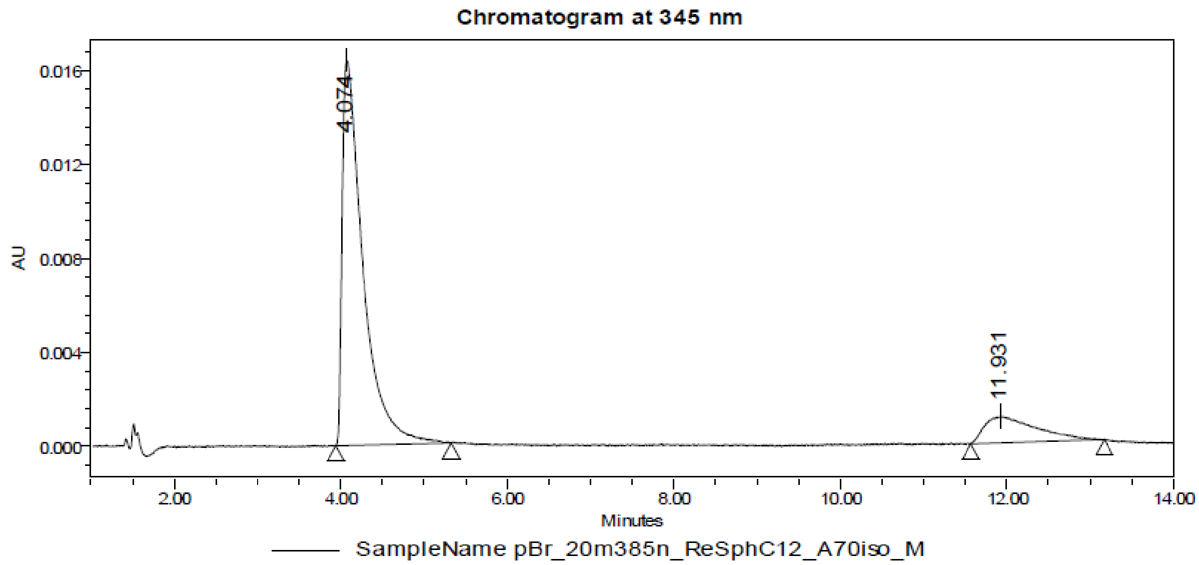

Peak Results

	Name	RT	Height	Area	% Area
1		4.074	16409	282345	85.38
2		11.931	1131	48338	14.62

Figure S122: Chromatogram of **6f** after irradiation at 385 nm in MeCN (100 µM) extracted at 345 nm; eluent mixture: MeCN/water (3:7) containing 0.1% formic acid.

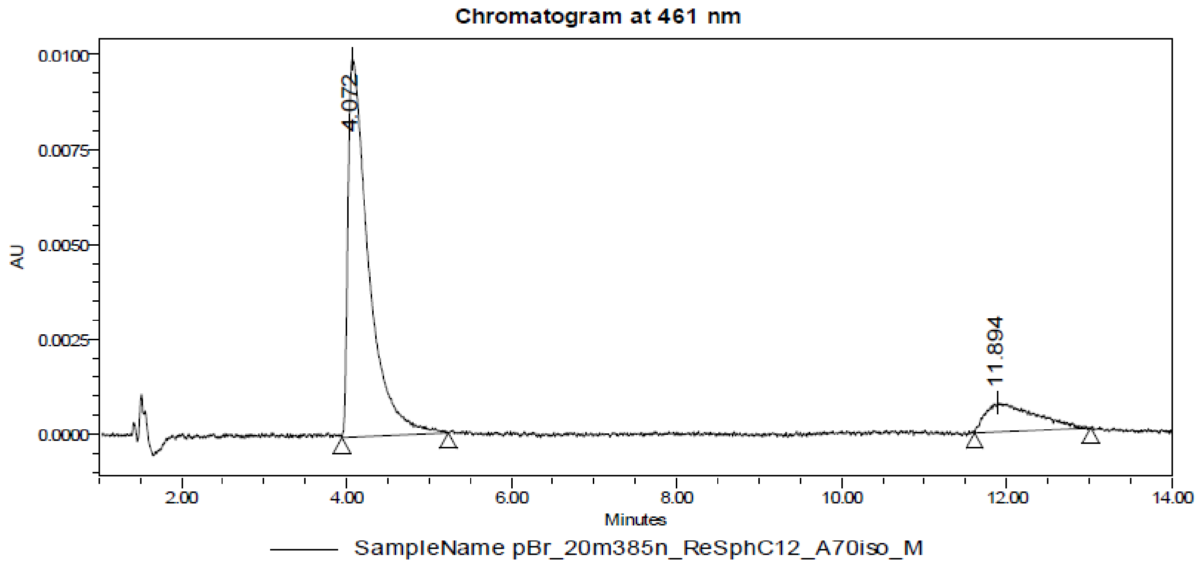

Peak Results

	Name	RT	Height	Area	% Area
1		4.072	9952	172762	84.58
2		11.894	770	31495	15.42

Figure S123: Chromatogram of **6f** after irradiation at 385 nm in MeCN (100 µM) extracted at 461 nm; eluent mixture: MeCN/water (3:7) containing 0.1% formic acid.

505 nm

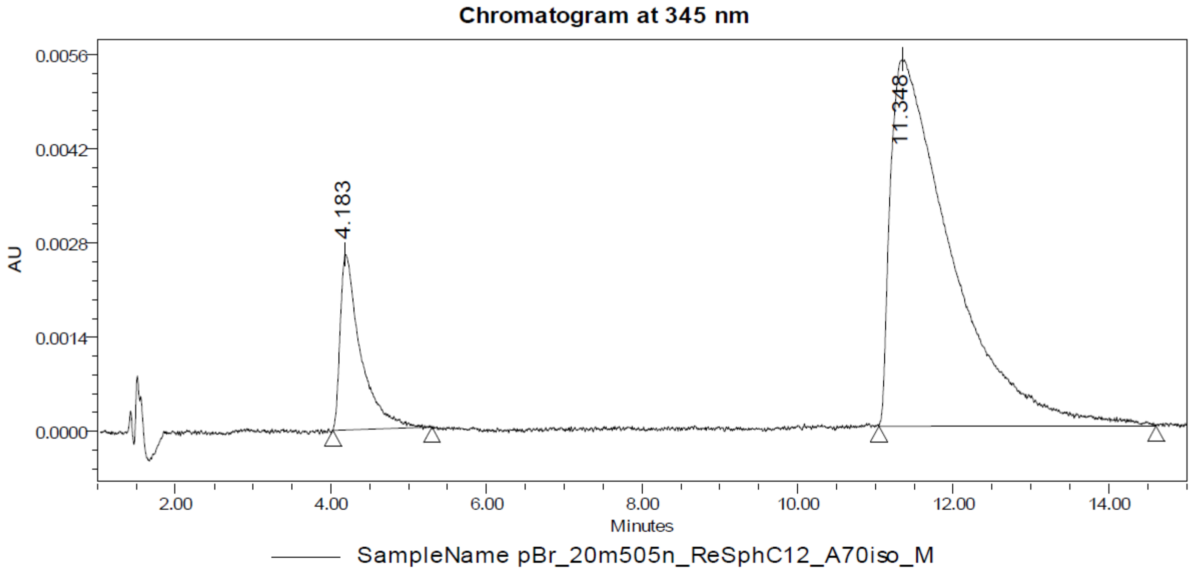

Peak Results

	Name	RT	Height	Area	% Area
1		4.183	2607	47799	13.86
2		11.348	5449	297147	86.14

Figure S124: Chromatogram of **6f** after irradiation at 505 nm in MeCN (100 µM) extracted at 345 nm; eluent mixture: MeCN/water (3:7) containing 0.1% formic acid.

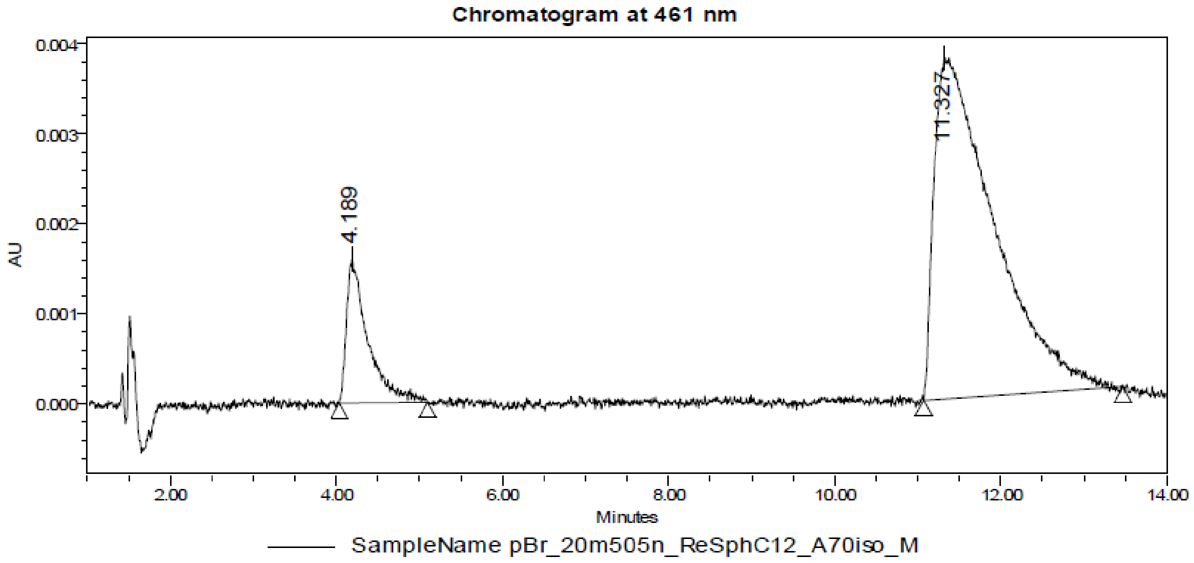

Peak Results

	Name	RT	Height	Area	% Area
1		4.189	1592	28535	13.07
2		11.327	3793	189811	86.93

Figure S125: Chromatogram of **6f** after irradiation at 505 nm in MeCN (100 µM) extracted at 461 nm; eluent mixture: MeCN/water (3:7) containing 0.1% formic acid.

6f in water

385 nm

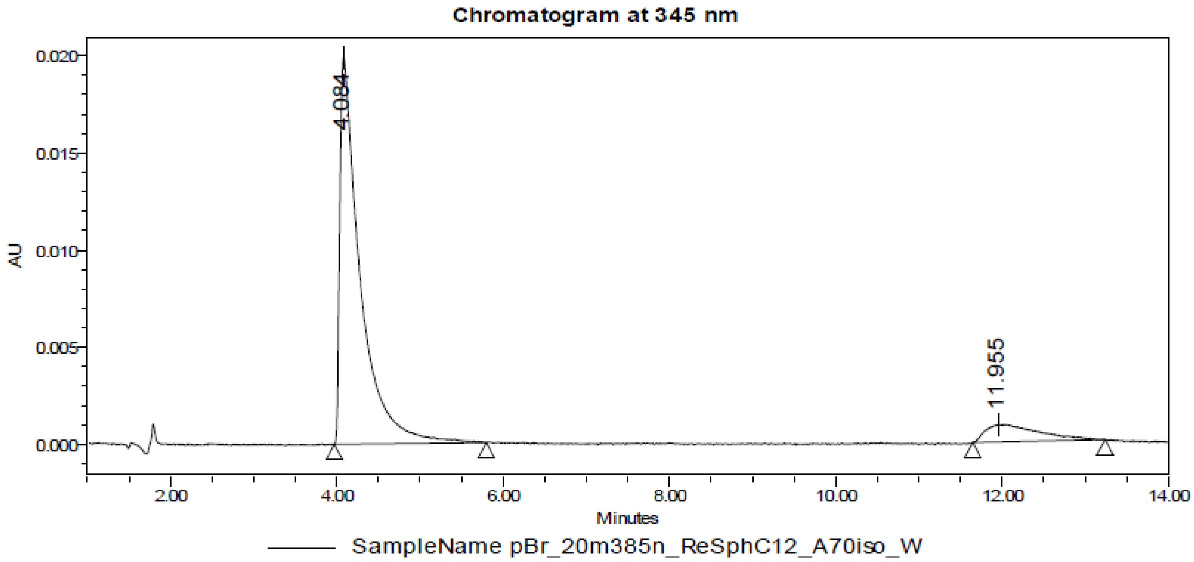

Peak Results

	Name	RT	Height	Area	% Area
1		4.084	19876	336109	89.68
2		11.955	896	38669	10.32

Figure S126: Chromatogram of **6f** after irradiation at 385 nm in water (100 µM) extracted at 345 nm; eluent mixture: MeCN/water (3:7) containing 0.1% formic acid.

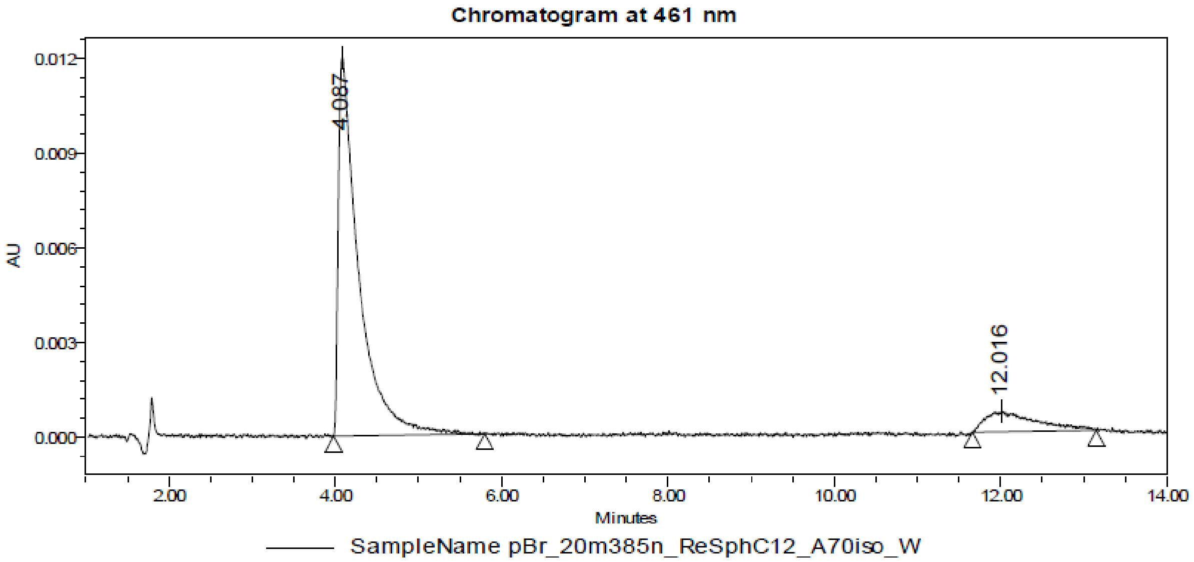

Peak Results

	Name	RT	Height	Area	% Area
1		4.087	11979	202483	88.08
2		12.016	655	27390	11.92

Figure S127: Chromatogram of **6f** after irradiation at 385 nm in water (100 µM) extracted at 461 nm; eluent mixture: MeCN/water (3:7) containing 0.1% formic acid.

505 nm

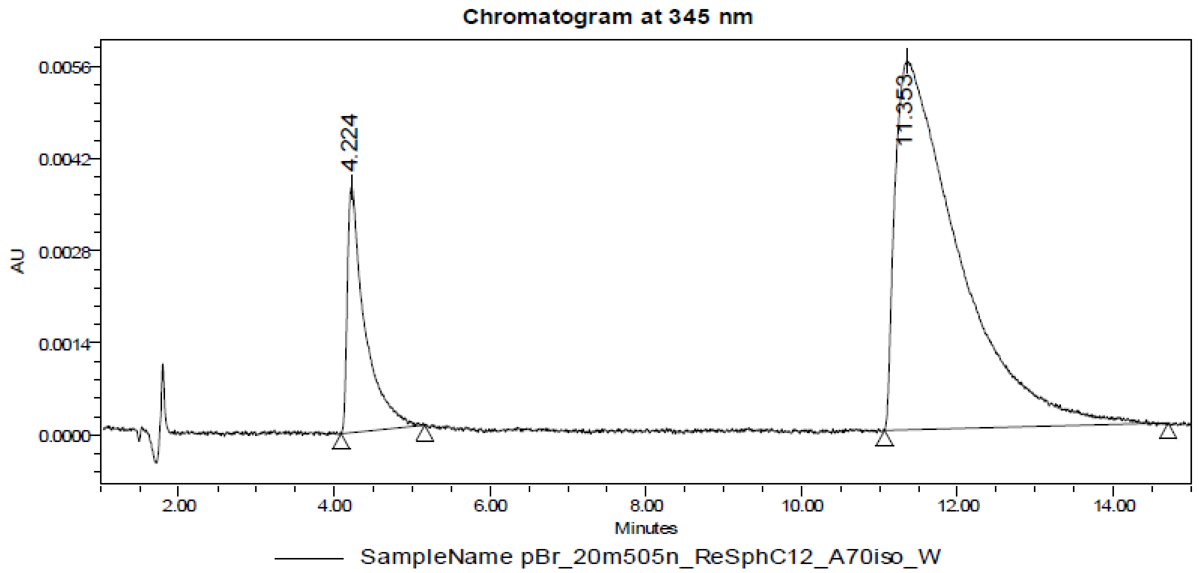

Peak Results

	Name	RT	Height	Area	% Area
1		4.224	3723	57630	15.35
2		11.353	5611	317848	84.65

Figure S128: Chromatogram of **6f** after irradiation at 505 nm in water (100 μM) extracted at 345 nm; eluent mixture: MeCN/water (3:7) containing 0.1% formic acid.

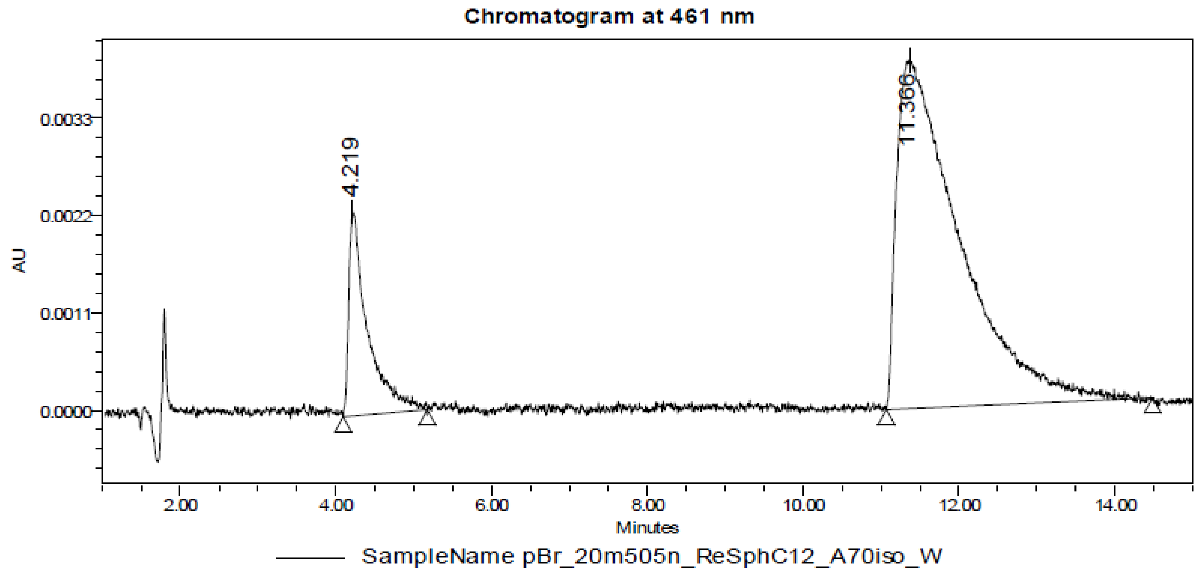

Peak Results

	Name	RT	Height	Area	% Area
1		4.219	2305	38159	14.70
2		11.366	3942	221479	85.30

Figure S129: Chromatogram of **6f** after irradiation at 505 nm water (100 μM) extracted at 461 nm; eluent mixture: MeCN/water (3:7) containing 0.1% formic acid.

6h in water

385 nm

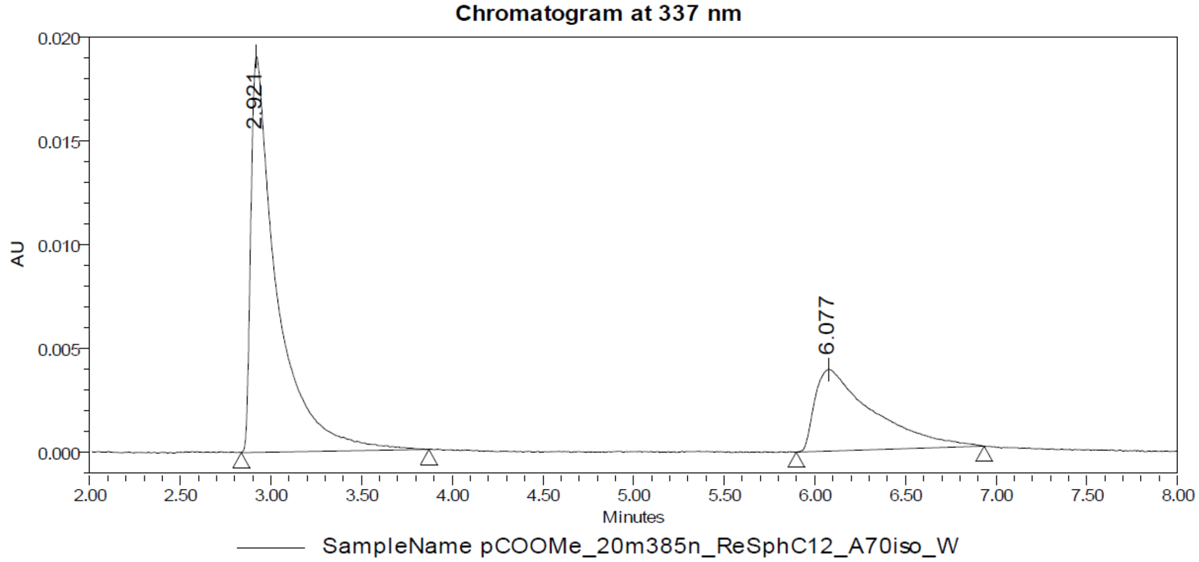

Peak Results

	Name	RT	Height	Area	% Area
1		2.921	19065	191080	68.64
2		6.077	3930	87316	31.36

Figure S130: Chromatogram of **6h** after irradiation at 385 nm in water (100 µM) extracted at 337 nm; eluent mixture: MeCN/water (3:7) containing 0.1% formic acid.

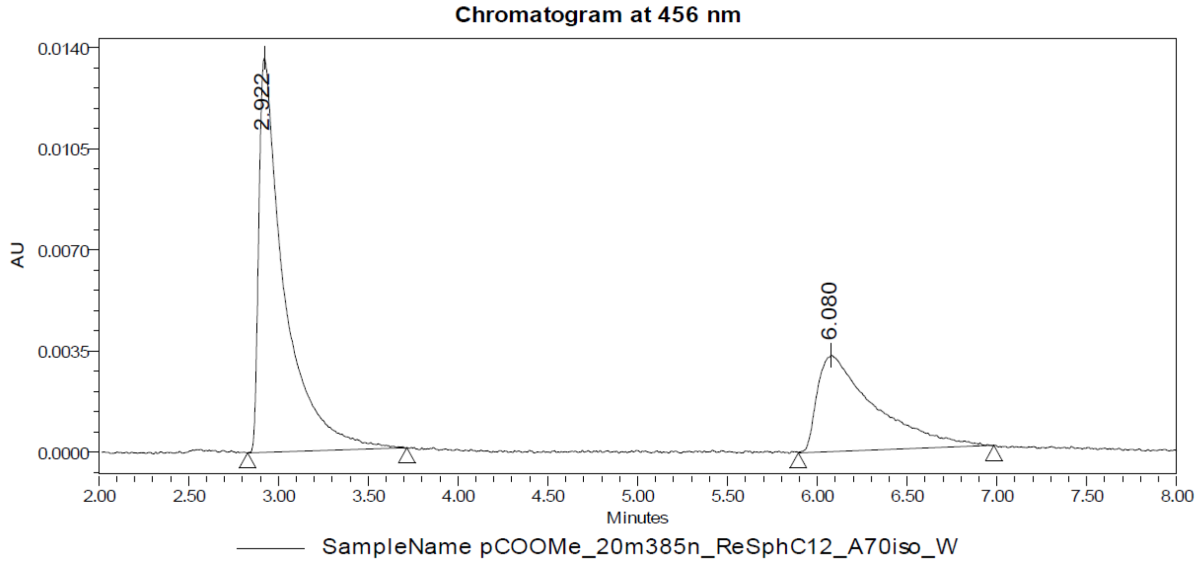

Peak Results

	Name	RT	Height	Area	% Area
1		2.922	13641	133983	64.57
2		6.080	3328	73505	35.43

Figure S131: Chromatogram of **6h** after irradiation at 385 nm in water (100 µM) extracted at 456 nm; eluent mixture: MeCN/water (3:7) containing 0.1% formic acid.

505 nm

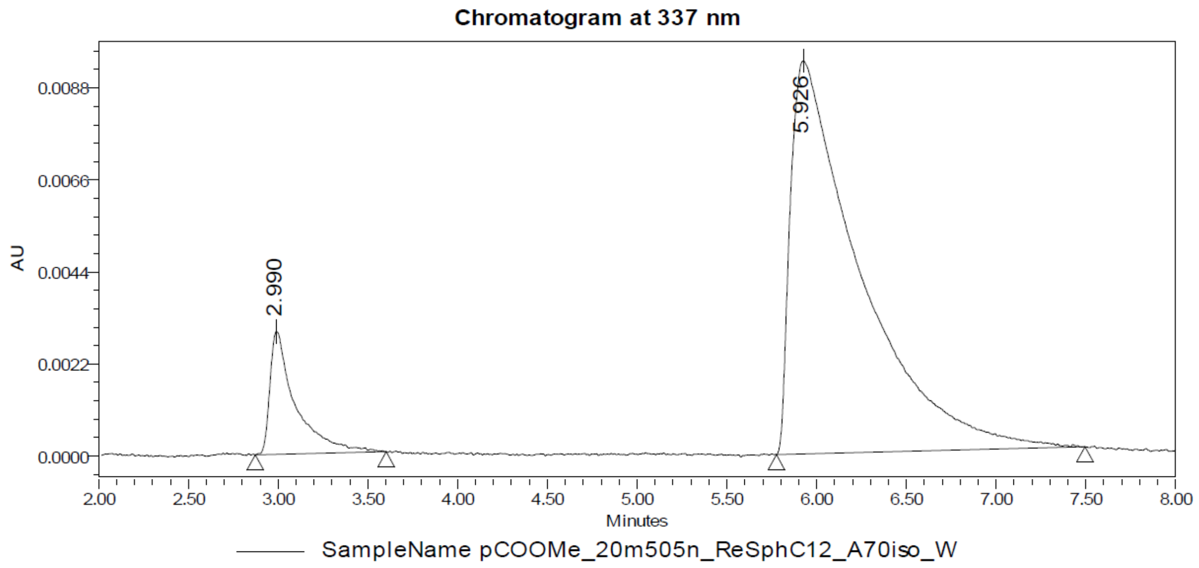

Peak Results

	Name	RT	Height	Area	% Area
1		2.990	2933	29103	10.32
2		5.926	9397	252969	89.68

Figure S132: Chromatogram of **6h** after irradiation at 505 nm in water (100 µM) extracted at 337 nm; eluent mixture: MeCN/water (3:7) containing 0.1% formic acid.

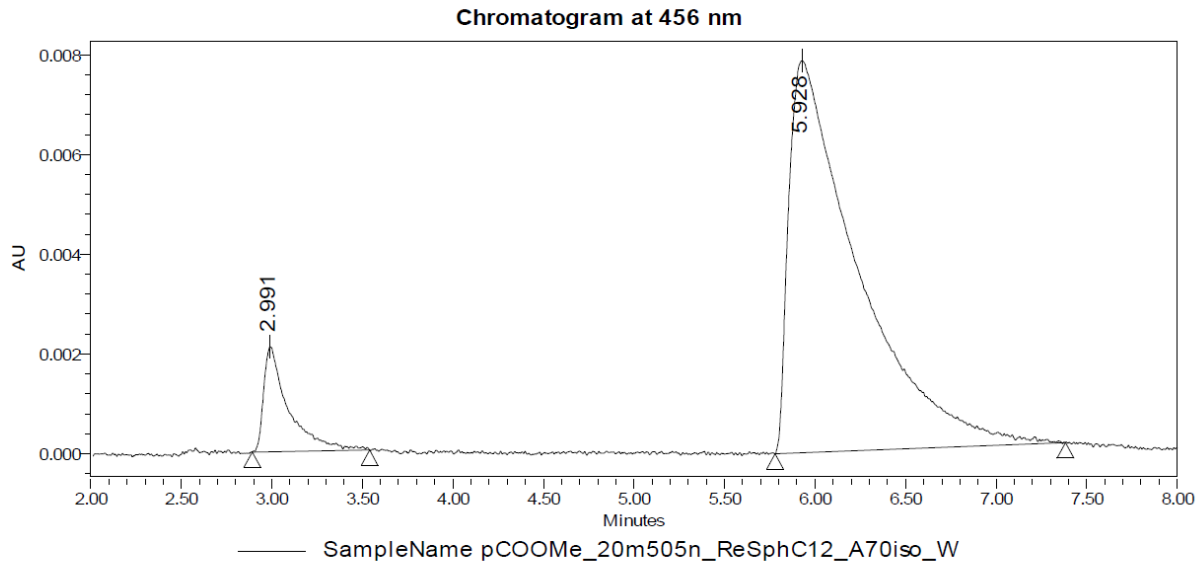

Peak Results

	Name	RT	Height	Area	% Area
1		2.991	2103	20133	8.81
2		5.928	7864	208261	91.19

Figure S133: Chromatogram of **6h** after irradiation at 505 nm in water (100 µM) extracted at 456 nm; eluent mixture: MeCN/water (3:7) containing 0.1% formic acid.

6k in MeCN

385 nm

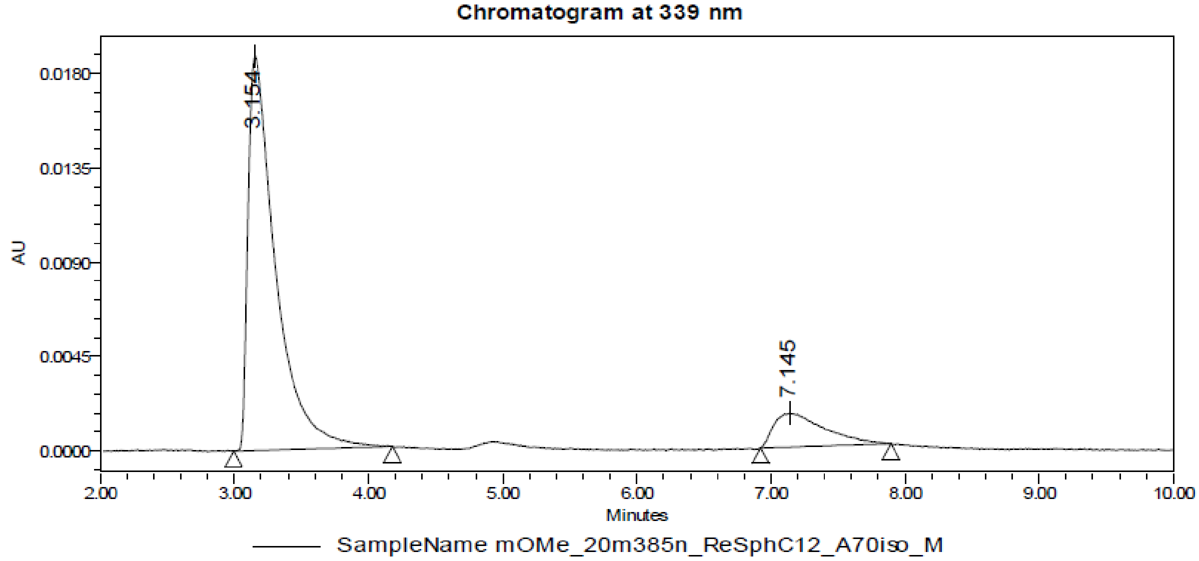

Peak Results

	Name	RT	Height	Area	% Area
1		3.154	18829	285694	85.55
2		7.145	1614	48258	14.45

Figure S134: Chromatogram of **6k** after irradiation at 385 nm in MeCN (100 µM) extracted at 339 nm; eluent mixture: MeCN/water (3:7) containing 0.1% formic acid.

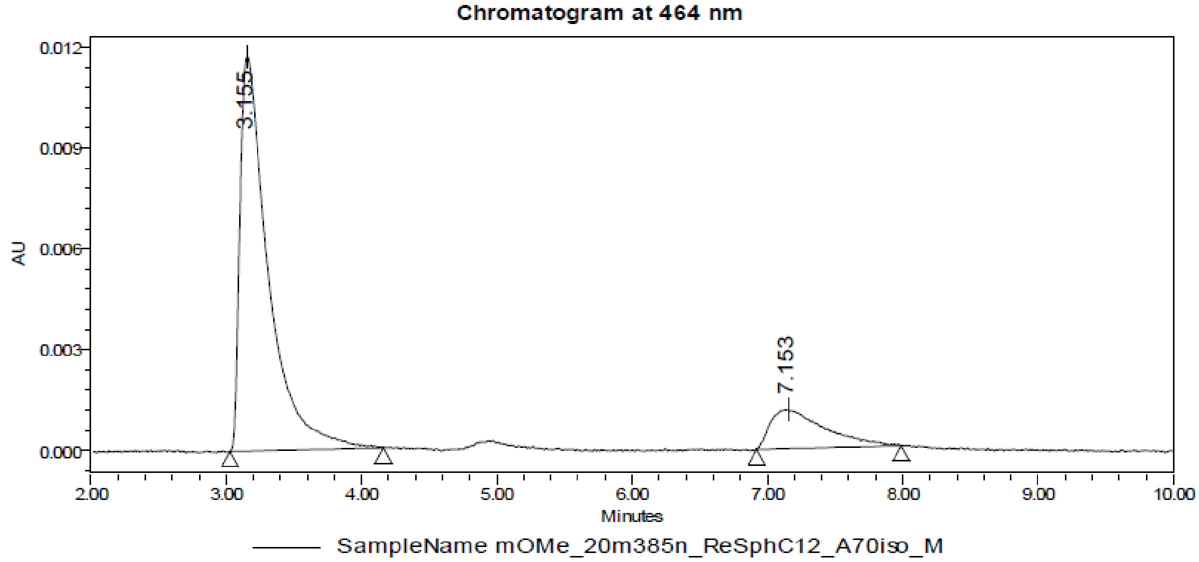

Peak Results

	Name	RT	Height	Area	% Area
1		3.155	11736	180508	82.97
2		7.153	1160	37053	17.03

Figure S135: Chromatogram of **6k** after irradiation at 385 nm in MeCN (100 µM) extracted at 464 nm; eluent mixture: MeCN/water (3:7) containing 0.1% formic acid.

505 nm

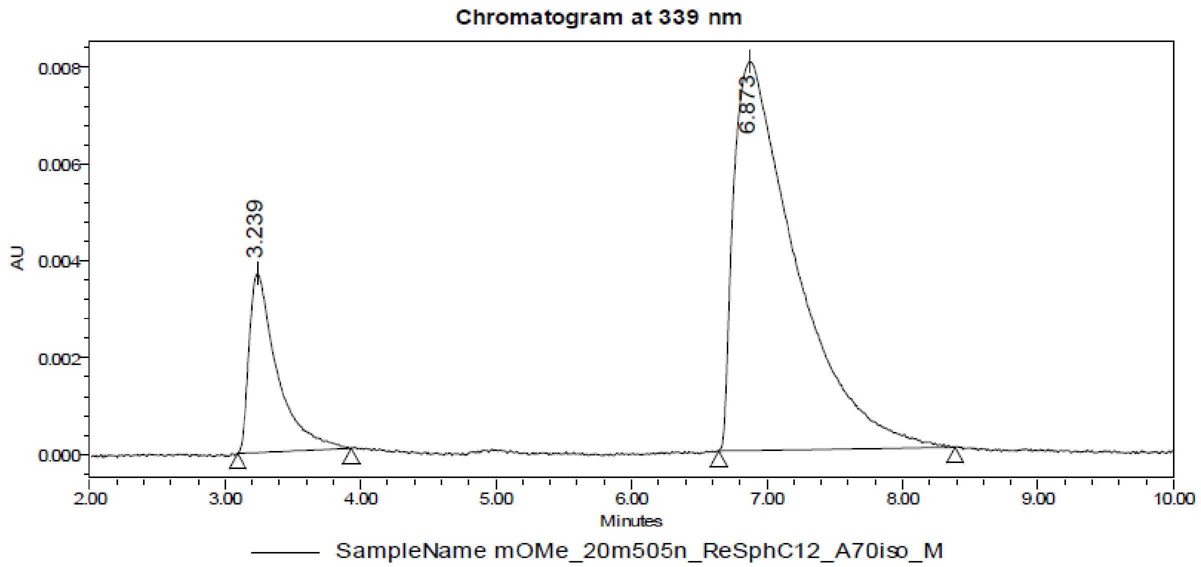

Peak Results

	Name	RT	Height	Area	% Area
1		3.239	3706	48204	15.56
2		6.873	8052	261509	84.44

Figure S136: Chromatogram of **6k** after irradiation at 505 nm in MeCN (100 µM) extracted at 339 nm; eluent mixture: MeCN/water (3:7) containing 0.1% formic acid.

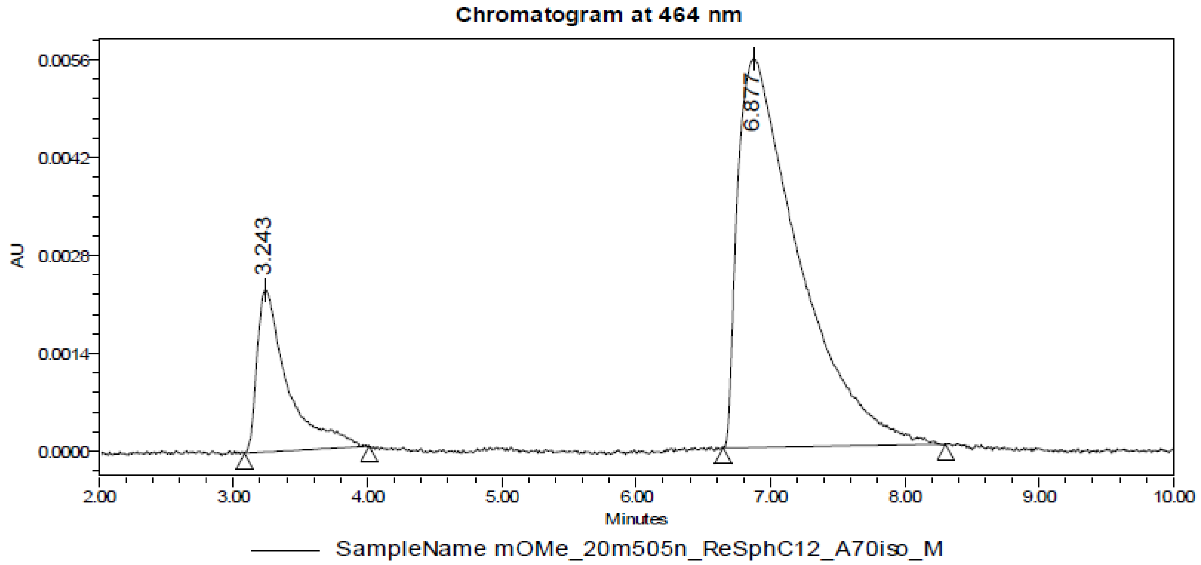

Peak Results

	Name	RT	Height	Area	% Area
1		3.243	2315	33096	15.59
2		6.877	5557	179135	84.41

Figure S137: Chromatogram of **6k** after irradiation at 505 nm in MeCN (100 µM) extracted at 464 nm; eluent mixture: MeCN/water (3:7) containing 0.1% formic acid.

6k in water

385 nm

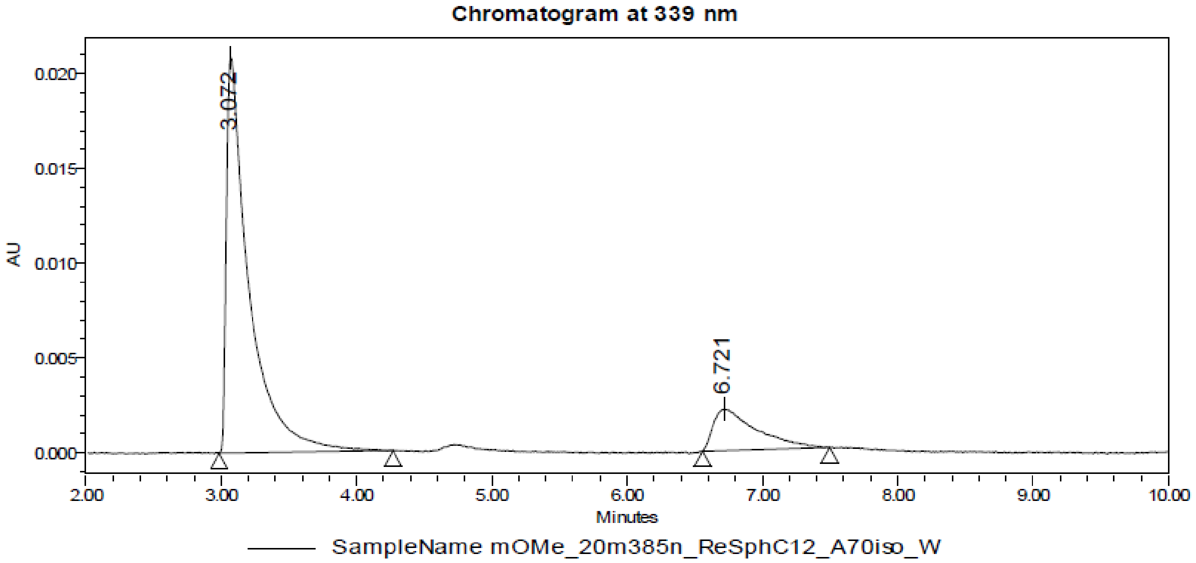

Peak Results

	Name	RT	Height	Area	% Area
1		3.072	20867	241423	83.30
2		6.721	2182	48412	16.70

Figure S138: Chromatogram of **6k** after irradiation at 385 nm in water (100 µM) extracted at 339 nm; eluent mixture: MeCN/water (3:7) containing 0.1% formic acid.

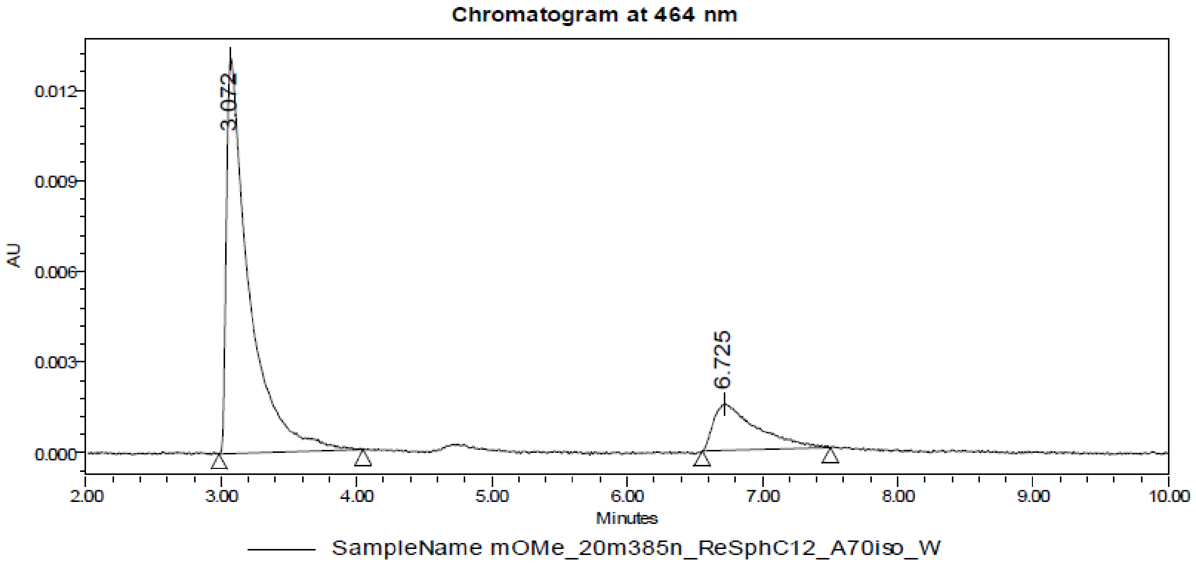

Peak Results

	Name	RT	Height	Area	% Area
1		3.072	13071	150654	81.70
2		6.725	1534	33752	18.30

Figure S139: Chromatogram of **6k** after irradiation at 385 nm in water (100 µM) extracted at 464 nm; eluent mixture: MeCN/water (3:7) containing 0.1% formic acid.

505 nm

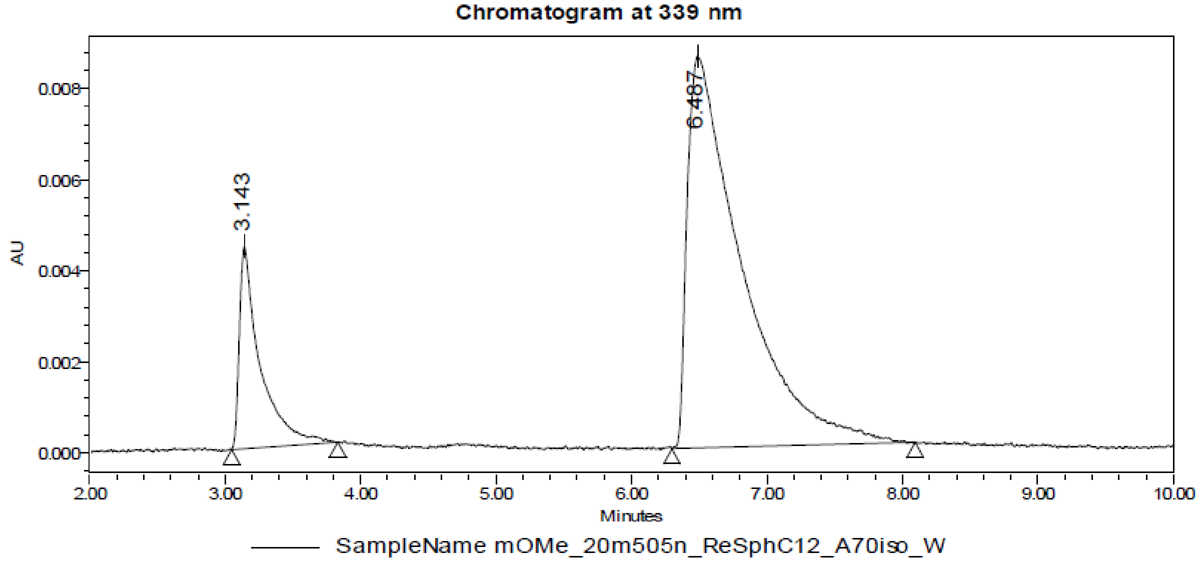

Peak Results

	Name	RT	Height	Area	% Area
1		3.143	4445	48099	16.72
2		6.487	8604	239529	83.28

Figure S140: Chromatogram of **6k** after irradiation at 505 nm in water (100 µM) extracted at 339 nm; eluent mixture: MeCN/water (3:7) containing 0.1% formic acid.

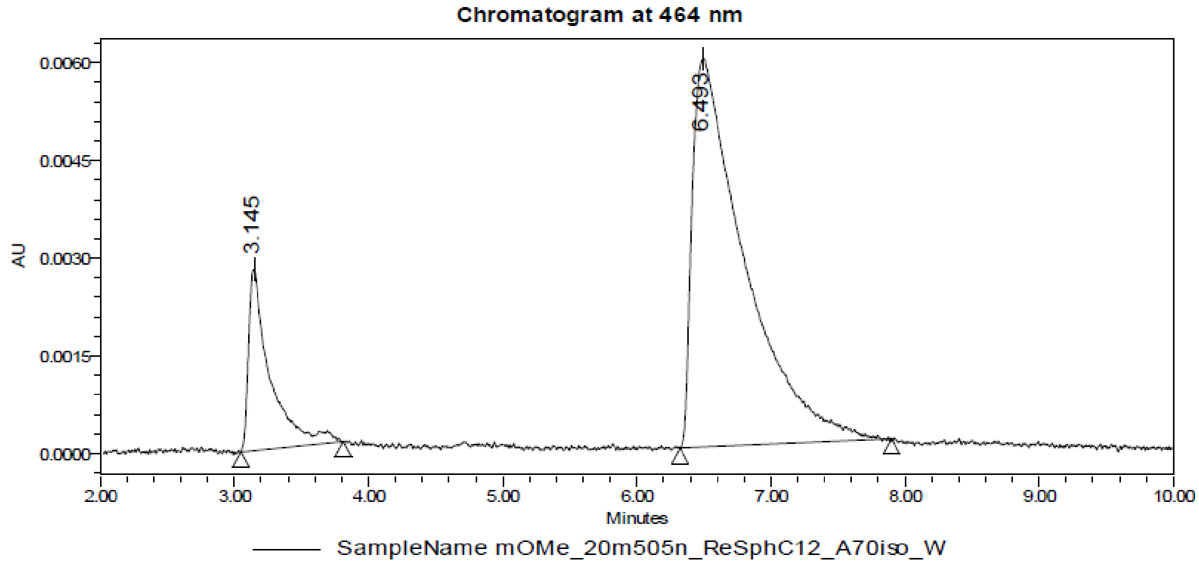

Peak Results

	Name	RT	Height	Area	% Area
1		3.145	2789	31503	16.29
2		6.493	5970	161842	83.71

Figure S141: Chromatogram of **6k** after irradiation at 505 nm in water (100 µM) extracted at 464 nm; eluent mixture: MeCN/water (3:7) containing 0.1% formic acid.

6l in MeCN

385 nm

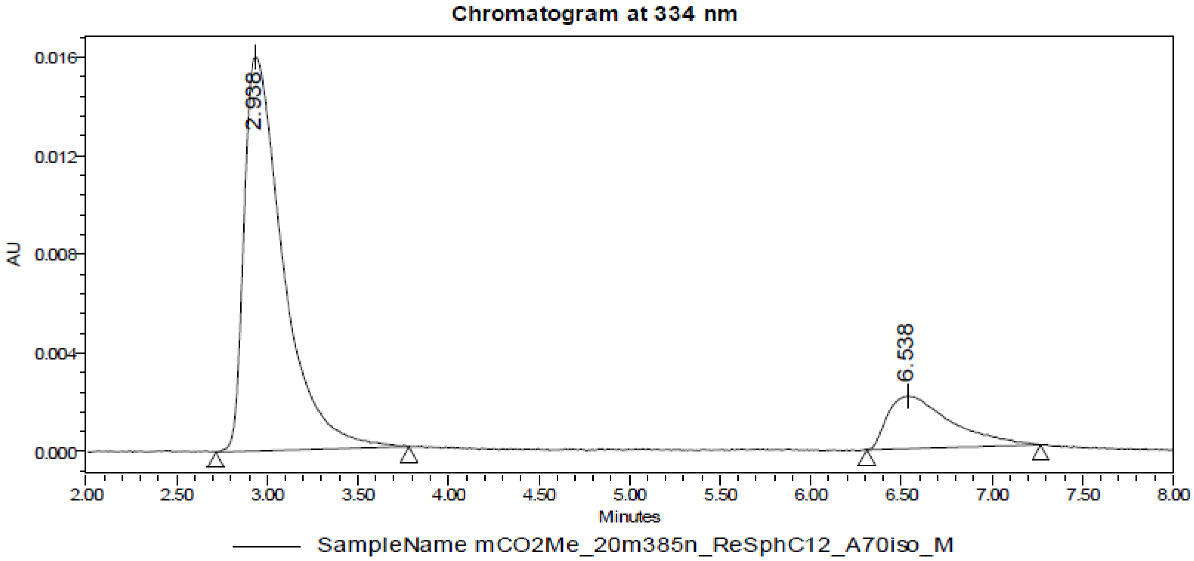

Peak Results

	Name	RT	Height	Area	% Area
1		2.938	15995	242127	83.69
2		6.538	2128	47187	16.31

Figure S142: Chromatogram of **6l** after irradiation at 385 nm in MeCN (100 µM) extracted at 334 nm; eluent mixture: MeCN/water (3:7) containing 0.1% formic acid.

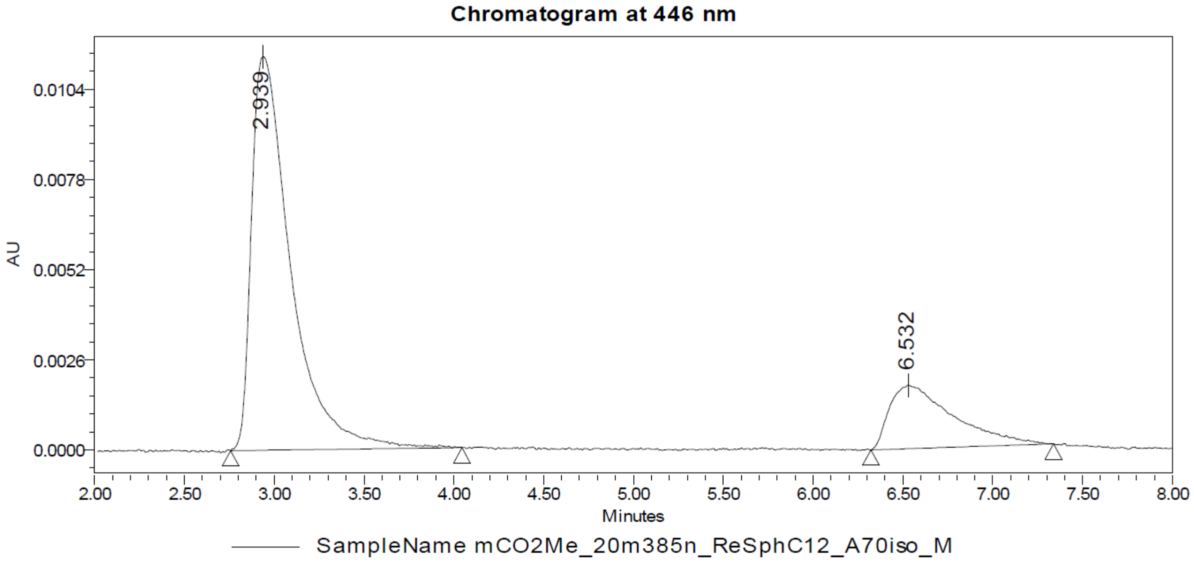

Peak Results

	Name	RT	Height	Area	% Area
1		2.939	11354	174709	80.91
2		6.532	1833	41228	19.09

Figure S143: Chromatogram of **6l** after irradiation at 385 nm in MeCN (100 µM) extracted at 446 nm; eluent mixture: MeCN/water (3:7) containing 0.1% formic acid.

505 nm

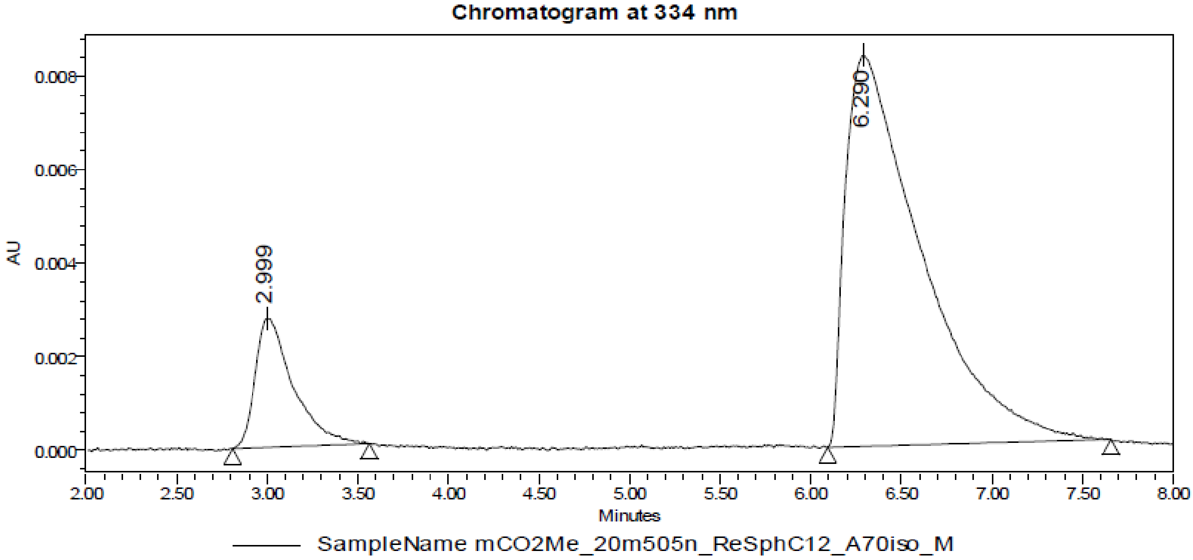

Peak Results

	Name	RT	Height	Area	% Area
1		2.999	2748	36173	13.72
2		6.290	8373	227573	86.28

Figure S144: Chromatogram of **6l** after irradiation at 505 nm in MeCN (100 µM) extracted at 334 nm; eluent mixture: MeCN/water (3:7) containing 0.1% formic acid.

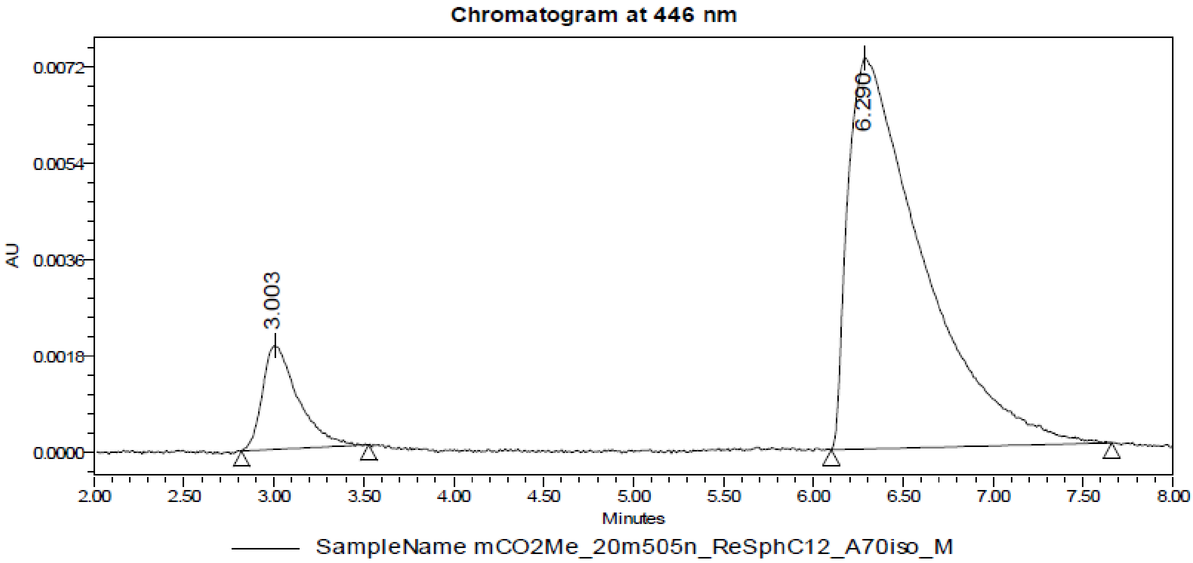

Peak Results

	Name	RT	Height	Area	% Area
1		3.003	1949	24211	10.90
2		6.290	7336	197818	89.10

Figure S145: Chromatogram of **6l** after irradiation at 505 nm in MeCN (100 µM) extracted at 446 nm; eluent mixture: MeCN/water (3:7) containing 0.1% formic acid.

6l in water

385 nm

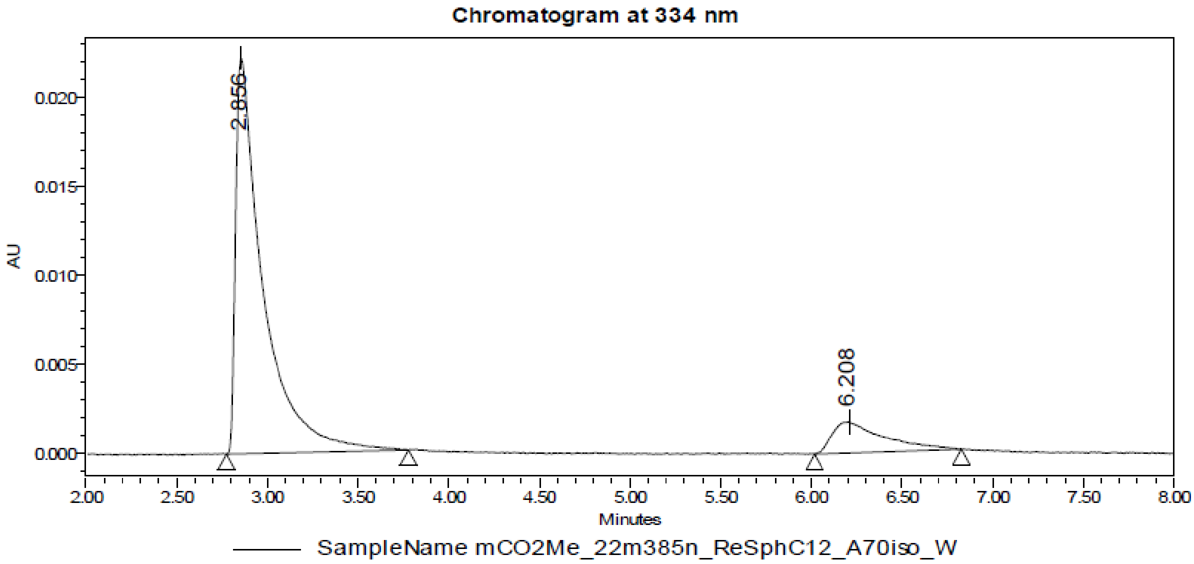

Peak Results

	Name	RT	Height	Area	% Area
1		2.856	22186	234488	87.28
2		6.208	1753	34186	12.72

Figure S146: Chromatogram of **6l** after irradiation at 385 nm in water (100 µM) extracted at 334 nm; eluent mixture: MeCN/water (3:7) containing 0.1% formic acid.

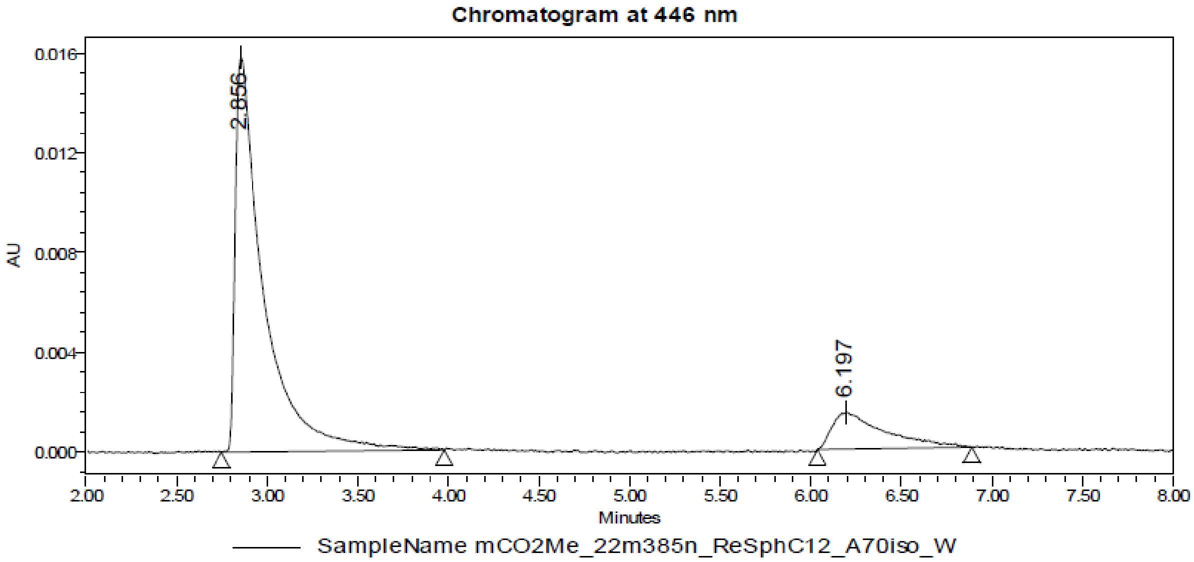

Peak Results

	Name	RT	Height	Area	% Area
1		2.856	15836	171552	85.25
2		6.197	1468	29693	14.75

Figure S147: Chromatogram of **6l** after irradiation at 385 nm in water (100 µM) extracted at 446 nm; eluent mixture: MeCN/water (3:7) containing 0.1% formic acid.

505 nm

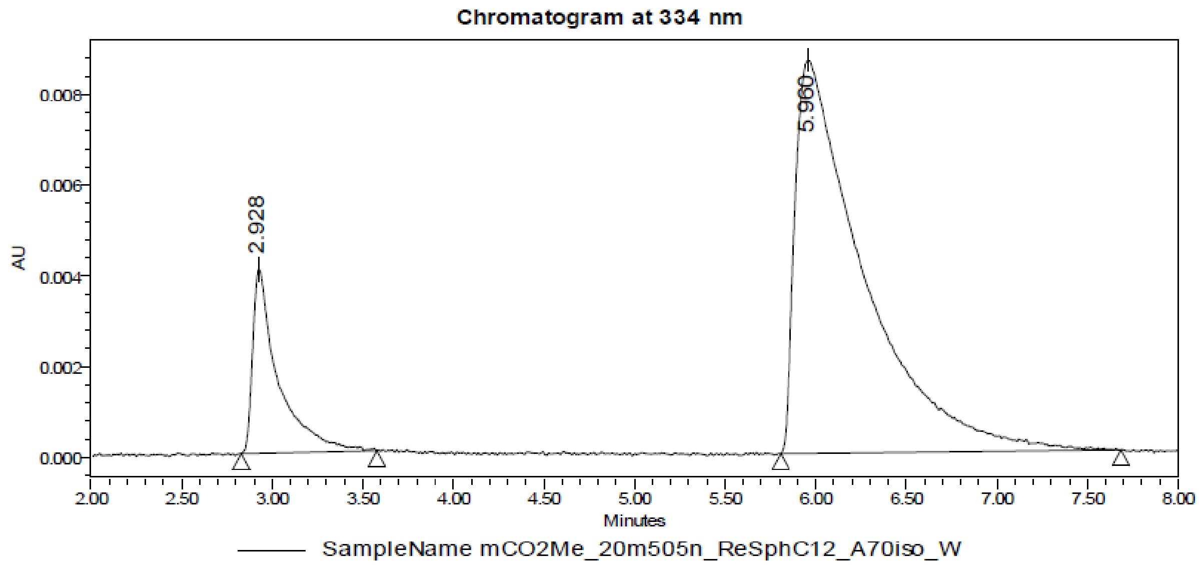

Peak Results

	Name	RT	Height	Area	% Area
1		2.928	4062	40208	15.02
2		5.960	8668	227549	84.98

Figure S148: Chromatogram of **6l** after irradiation at 505 nm in water (100 μM) extracted at 334 nm; eluent mixture: MeCN/water (3:7) containing 0.1% formic acid.

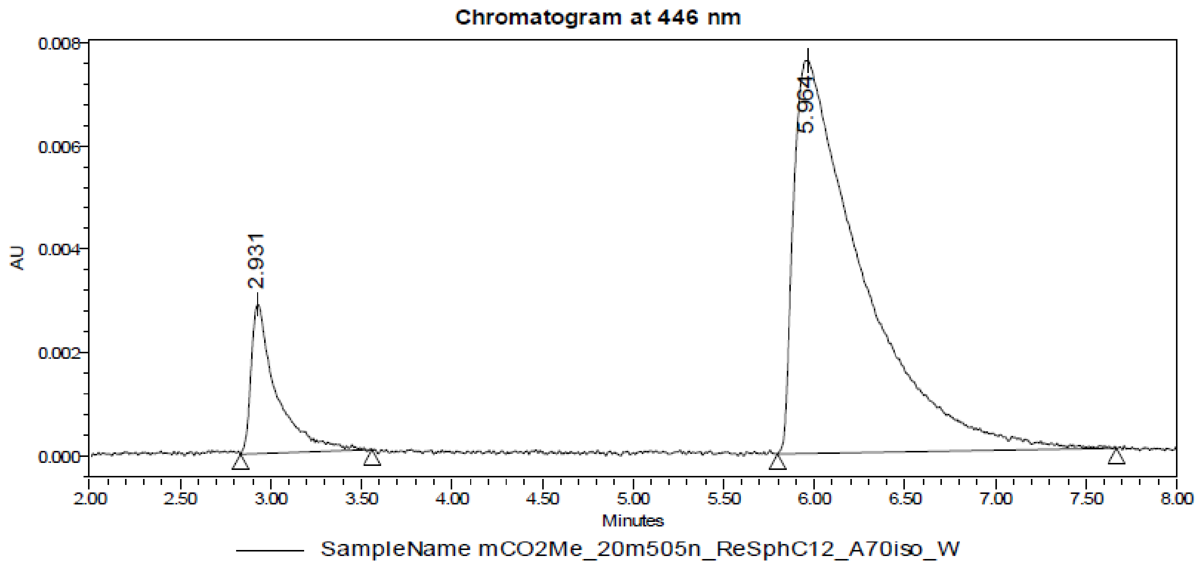

Peak Results

	Name	RT	Height	Area	% Area
1		2.931	2891	28418	12.39
2		5.964	7623	200885	87.61

Figure S149: Chromatogram of **6l** after irradiation at 505 nm in water (100 μM) extracted at 446 nm; eluent mixture: MeCN/water (3:7) containing 0.1% formic acid.

6n in MeCN

385 nm

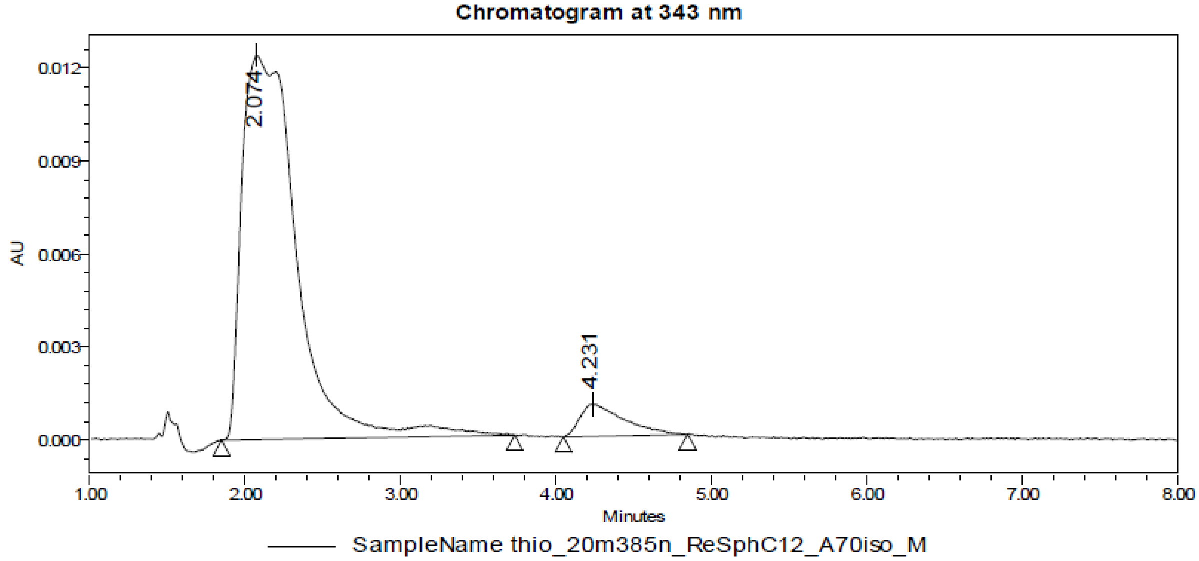

Peak Results

	Name	RT	Height	Area	% Area
1		2.074	12406	305723	93.75
2		4.231	1029	20396	6.25

Figure S150: Chromatogram of **6n** after irradiation at 385 nm in MeCN (100 µM) extracted at 343 nm; eluent mixture: MeCN/water (3:7) containing 0.1% formic acid.

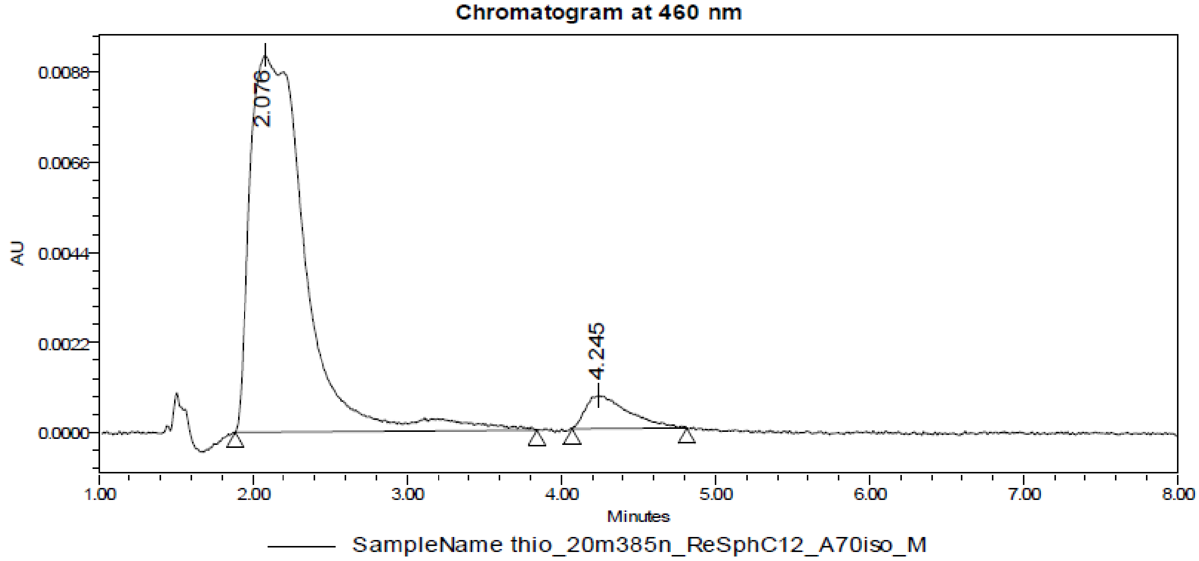

Peak Results

	Name	RT	Height	Area	% Area
1		2.076	9200	228153	93.61
2		4.245	810	15573	6.39

Figure S151: Chromatogram of **6n** after irradiation at 385 nm in MeCN (100 µM) extracted at 460 nm; eluent mixture: MeCN/water (3:7) containing 0.1% formic acid.

505 nm

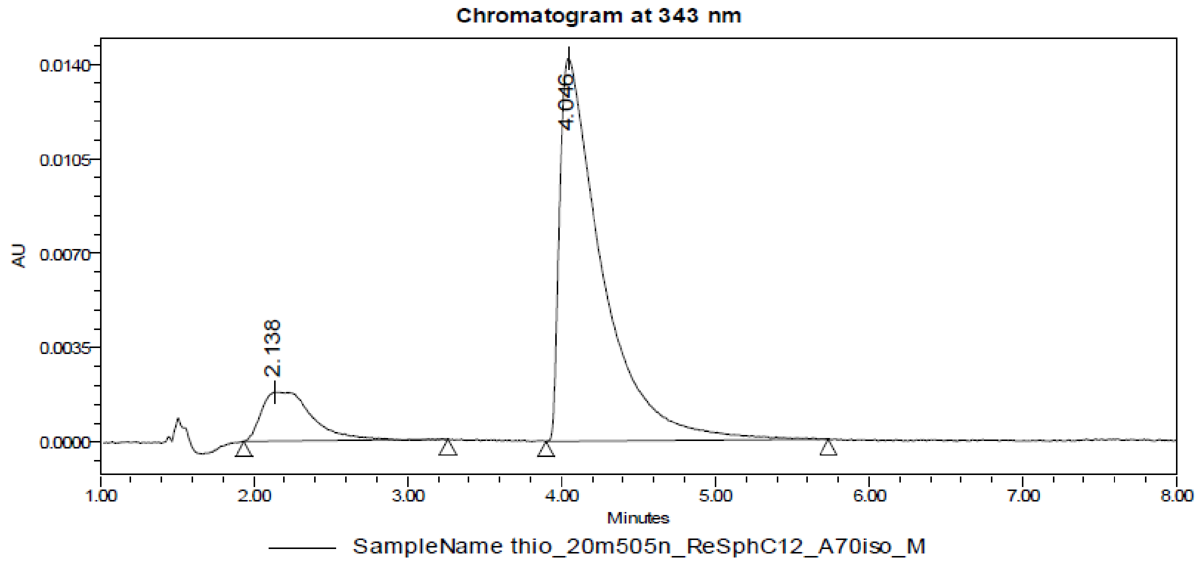

Peak Results

	Name	RT	Height	Area	% Area
1		2.138	1816	41678	13.25
2		4.046	14180	272910	86.75

Figure S152: Chromatogram of **6n** after irradiation at 505 nm in MeCN (100 µM) extracted at 343 nm; eluent mixture: MeCN/water (3:7) containing 0.1% formic acid.

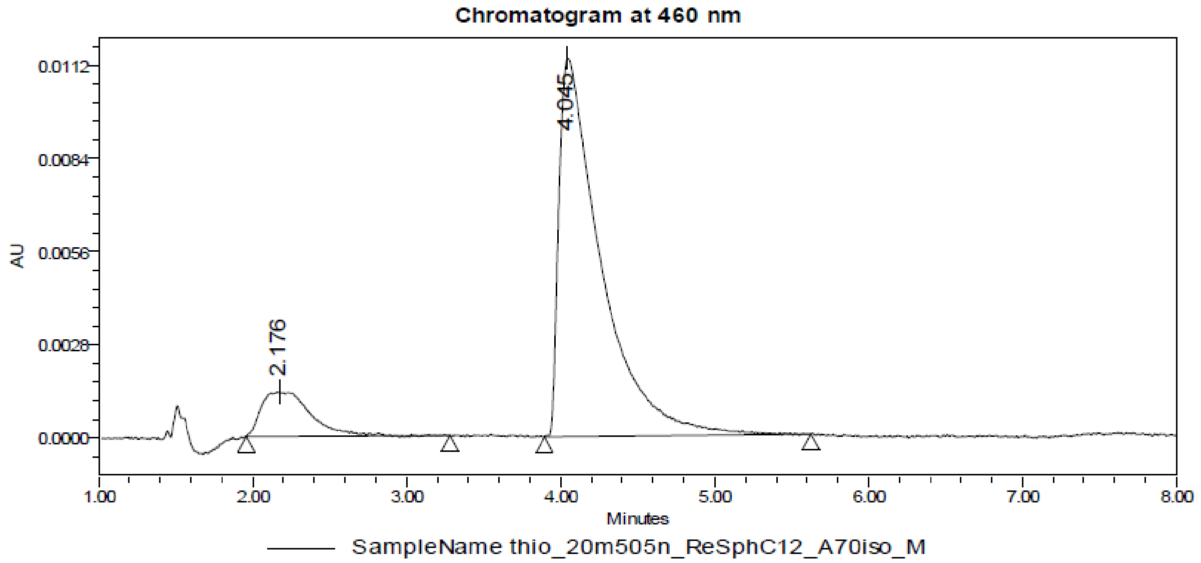

Peak Results

	Name	RT	Height	Area	% Area
1		2.176	1339	30131	12.24
2		4.045	11381	216082	87.76

Figure S153: Chromatogram of **6n** after irradiation at 505 nm in MeCN (100 µM) extracted at 460 nm; eluent mixture: MeCN/water (3:7) containing 0.1% formic acid.

6n in water

385 nm

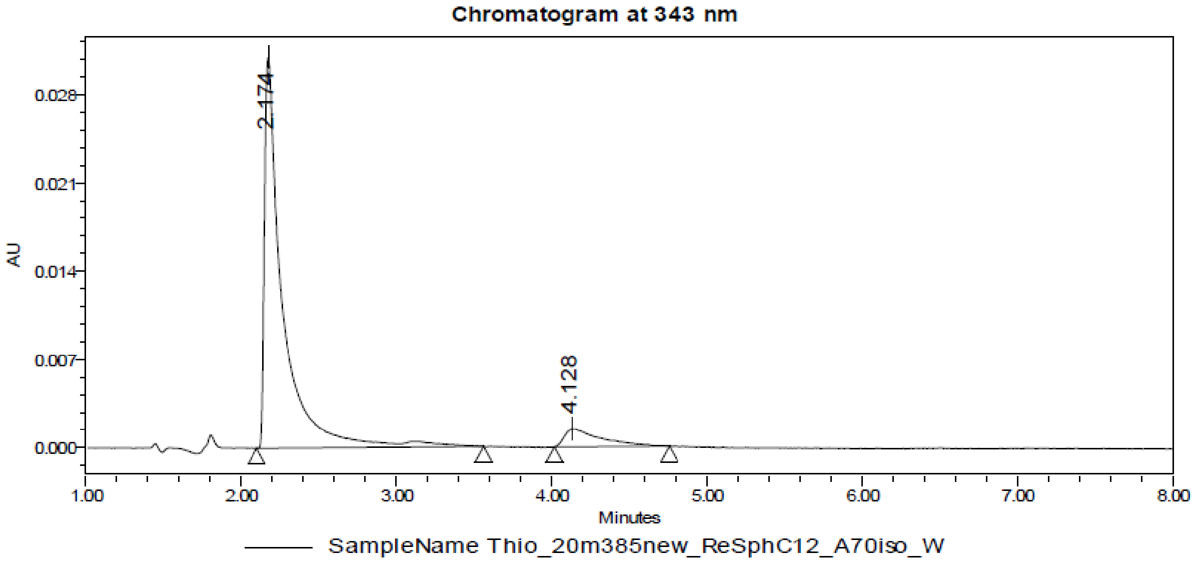

Peak Results

	Name	RT	Height	Area	% Area
1		2.174	31028	251715	91.53
2		4.128	1435	23300	8.47

Figure S154: Chromatogram of **6n** after irradiation at 385 nm in water (100 µM) extracted at 343 nm; eluent mixture: MeCN/water (3:7) containing 0.1% formic acid.

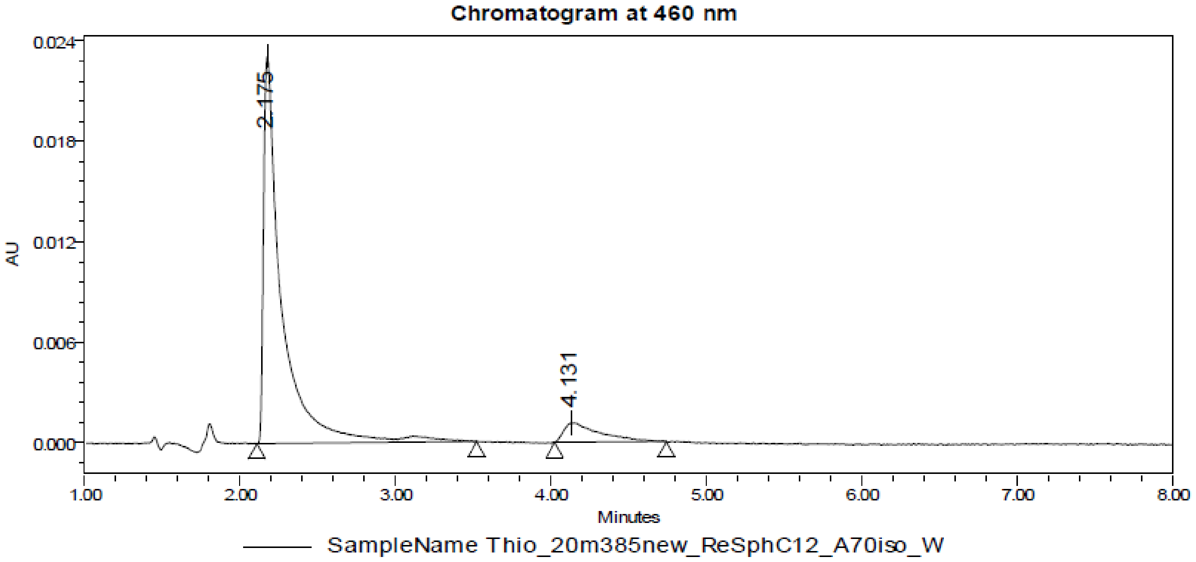

Peak Results

	Name	RT	Height	Area	% Area
1		2.175	23148	186958	91.10
2		4.131	1135	18266	8.90

Figure S155: Chromatogram of **6n** after irradiation at 385 nm in water (100 µM) extracted at 460 nm; eluent mixture: MeCN/water (3:7) containing 0.1% formic acid.

505 nm

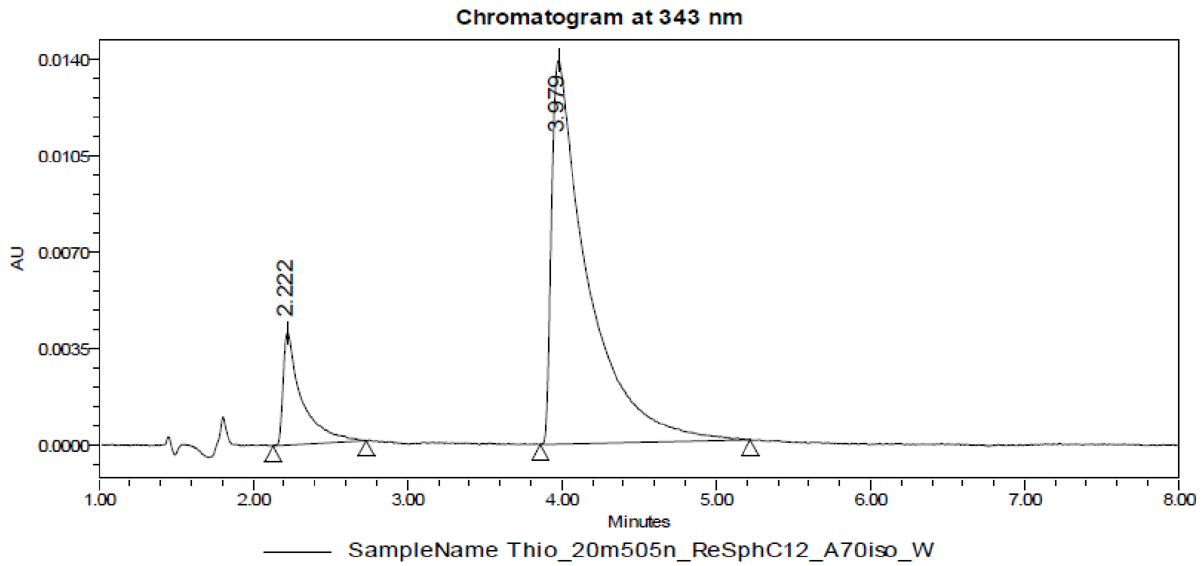

Peak Results

	Name	RT	Height	Area	% Area
1		2.222	4086	34246	13.06
2		3.979	13935	228073	86.94

Figure S156: Chromatogram of **6n** after irradiation at 505 nm in water (100 µM) extracted at 343 nm; eluent mixture: MeCN/water (3:7) containing 0.1% formic acid.

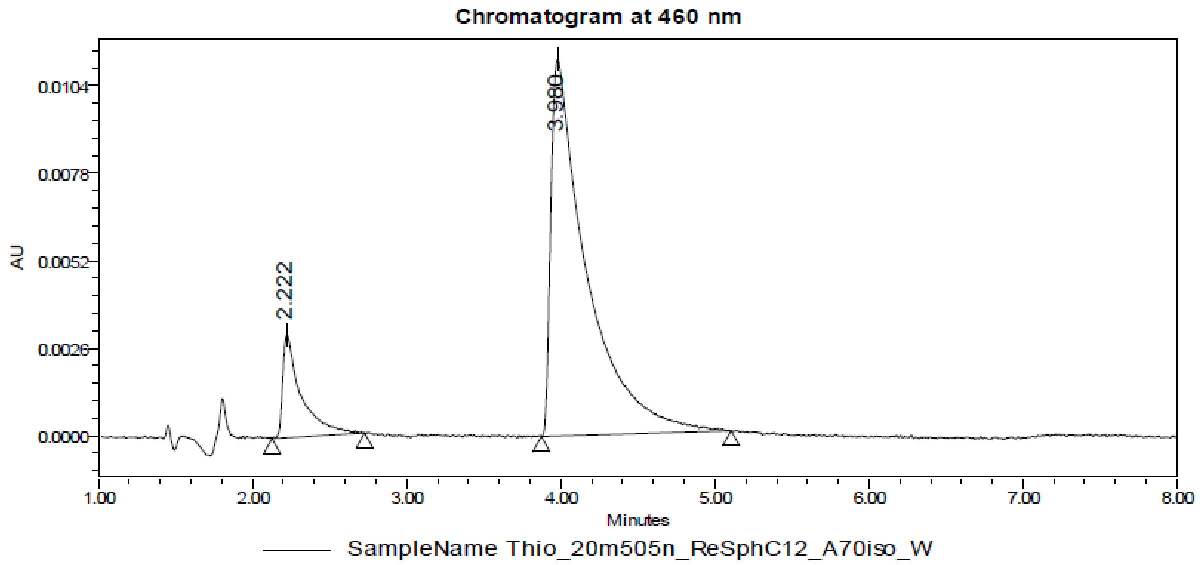

Peak Results

	Name	RT	Height	Area	% Area
1		2.222	3047	25402	12.34
2		3.980	1118[illegible]	180506	87.66

Figure S157: Chromatogram of **6n** after irradiation at 505 nm in water (100 µM) extracted at 460 nm; eluent mixture: MeCN/water (3:7) containing 0.1% formic acid.

6o in water

385 nm

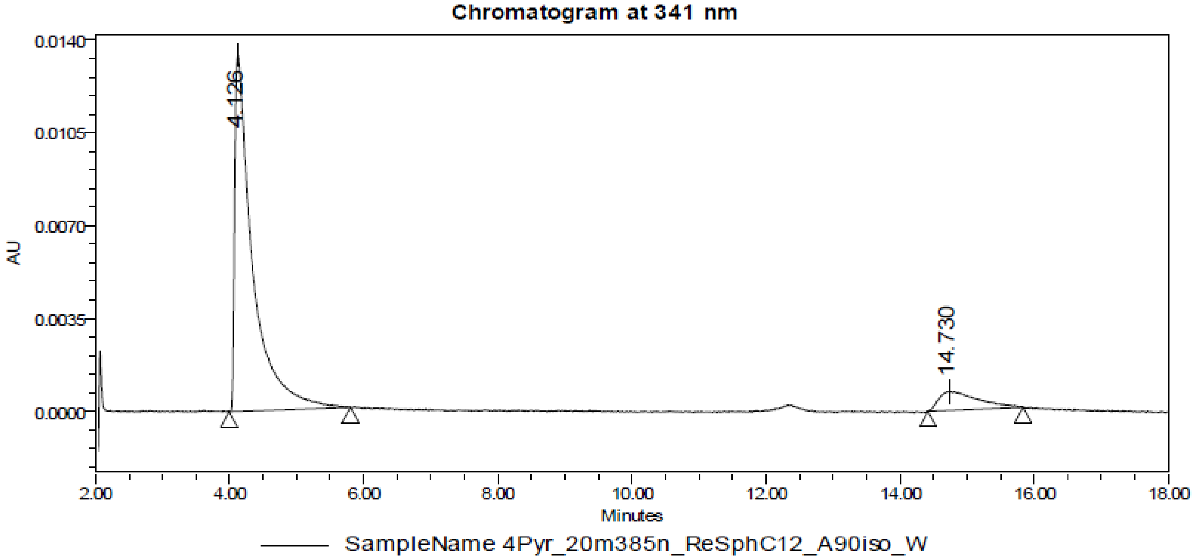

Peak Results

	Name	RT	Height	Area	% Area
1		4.126	13429	248332	89.84
2		14.730	708	28079	10.16

Figure S158: Chromatogram of **6o** after irradiation at 385 nm (100 µM, water) extracted at 341 nm; eluent mixture: MeCN/water (1:9) containing 0.1% formic acid.

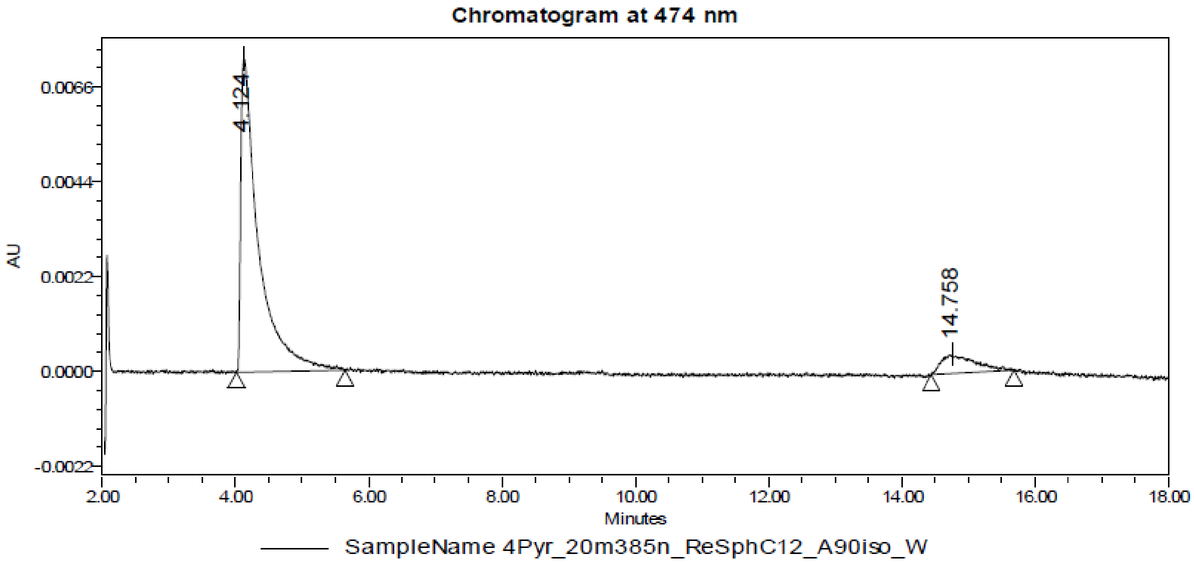

Peak Results

	Name	RT	Height	Area	% Area
1		4.124	7287	134579	89.98
2		14.758	442	14986	10.02

Figure S159: Chromatogram of **6o** after irradiation at 385 nm (100 µM, water) extracted at 474 nm; eluent mixture: MeCN/water (1:9) containing 0.1% formic acid.

505 nm

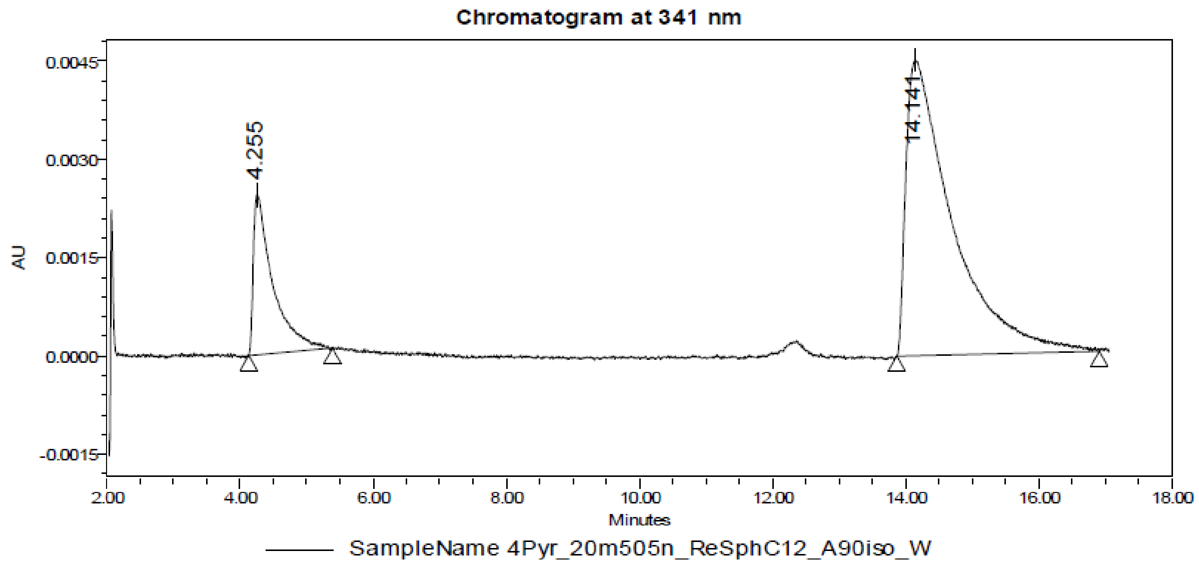

Peak Results

	Name	RT	Height	Area	% Area
1		4.255	2441	49853	18.64
2		14.141	4512	217558	81.36

Figure S160: Chromatogram of **6o** after irradiation at 505 nm (100 µM, water) extracted at 341 nm; eluent mixture: MeCN/water (1:9) containing 0.1% formic acid.

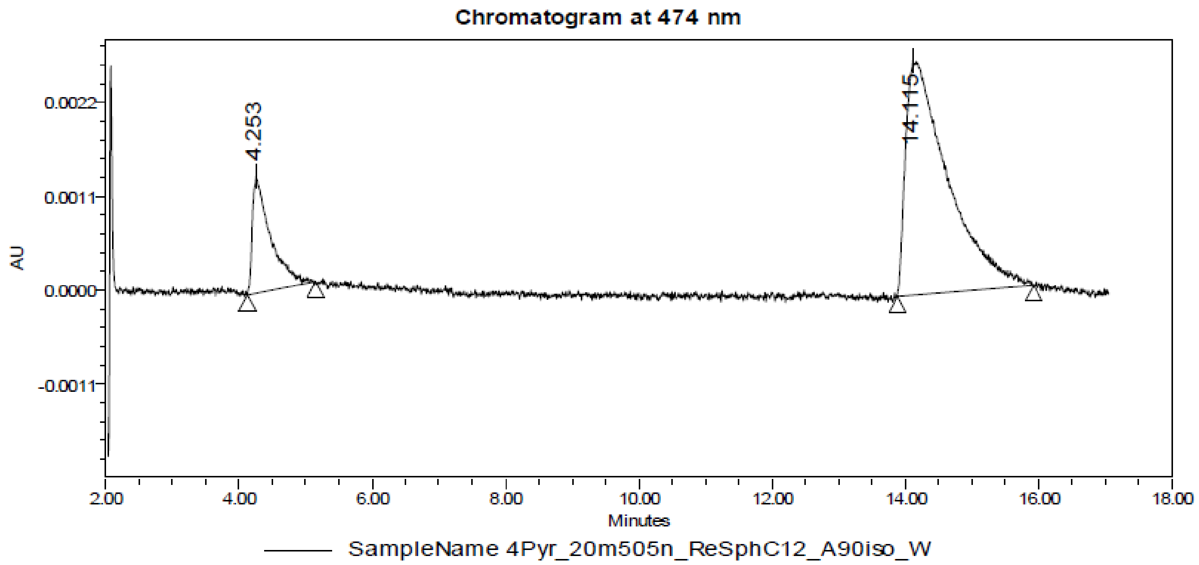

Peak Results

	Name	RT	Height	Area	% Area
1		4.253	1376	26308	17.89
2		14.115	2755	120712	82.11

Figure S161: Chromatogram of **6o** after irradiation at 505 nm (100 µM, water) extracted at 474 nm; eluent mixture: MeCN/water (1:9) containing 0.1% formic acid.

6p in MeCN

385 nm

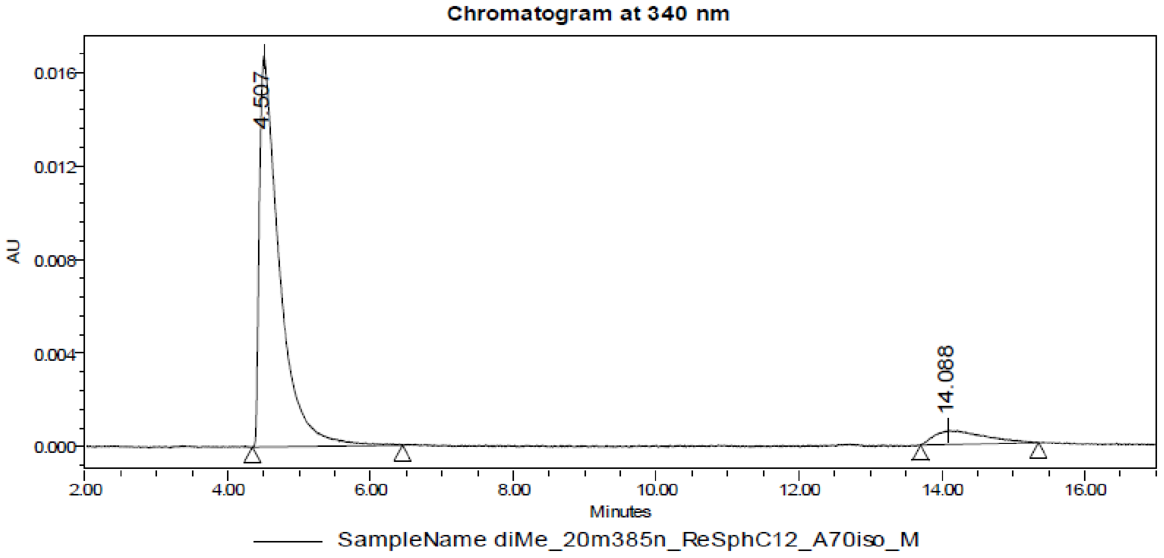

Peak Results

	Name	RT	Height	Area	% Area
1		4.507	16696	329260	91.98
2		14.088	600	28708	8.02

Figure S162: Chromatogram of **6p** after irradiation at 385 nm (100 µM, MeCN) extracted at 340 nm; eluent mixture: MeCN/water (3:7) containing 0.1% formic acid.

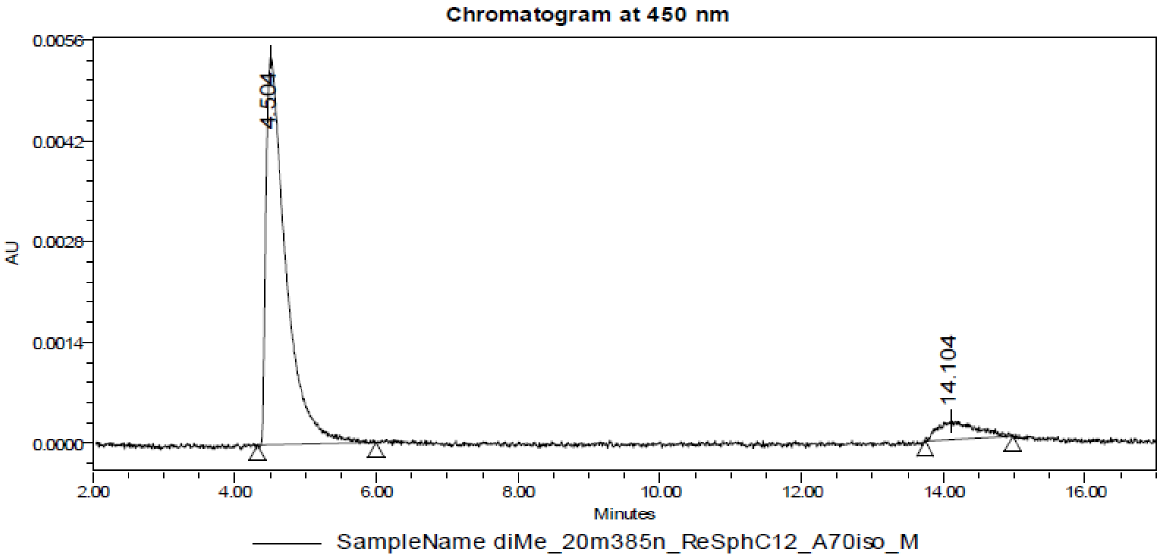

Peak Results

	Name	RT	Height	Area	% Area
1		4.504	5382	104290	91.82
2		14.104	258	9287	8.18

Figure S163: Chromatogram of **6p** after irradiation at 385 nm (100 µM, MeCN) extracted at 450 nm; eluent mixture: MeCN/water (3:7) containing 0.1% formic acid.

505 nm

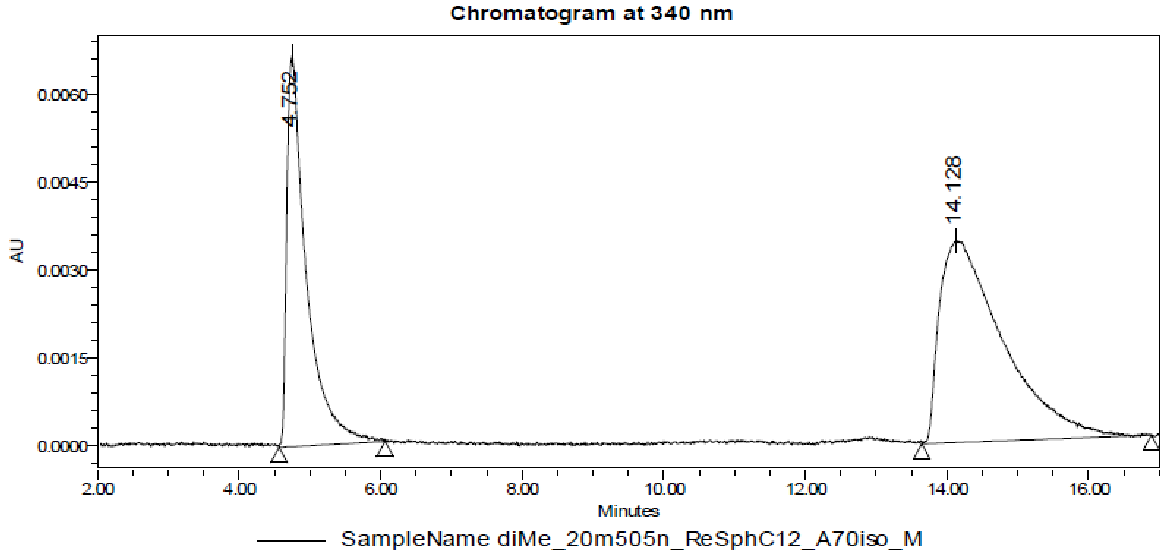

Peak Results

	Name	RT	Height	Area	% Area
1		4.752	6682	130164	37.23
2		14.128	3458	219446	62.77

Figure S164: Chromatogram of **6p** after irradiation at 505 nm (100 µM, MeCN) extracted at 340 nm; eluent mixture: MeCN/water (3:7) containing 0.1% formic acid.

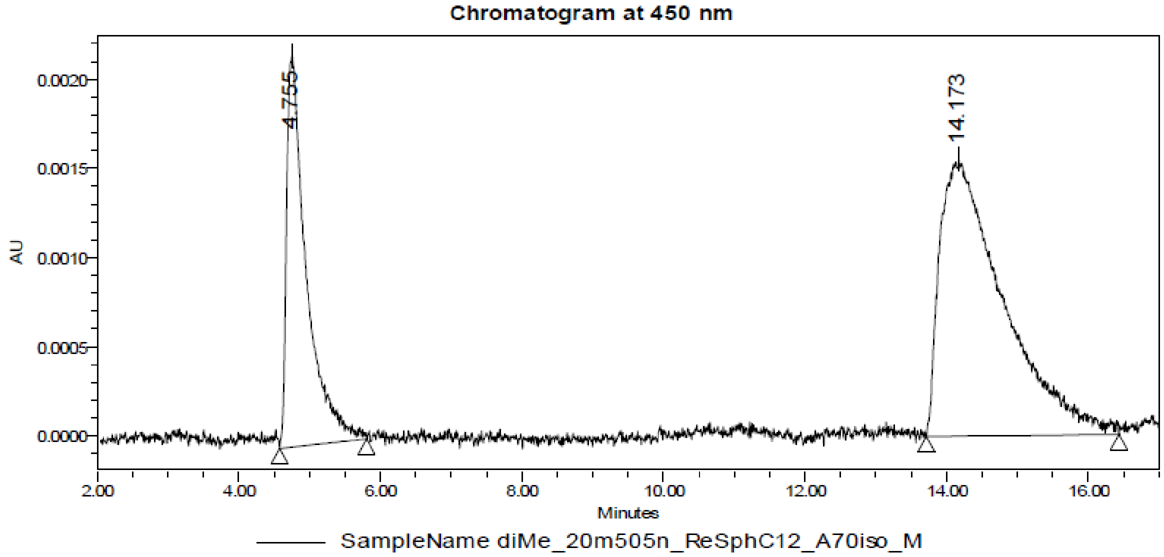

Peak Results

	Name	RT	Height	Area	% Area
1		4.755	2196	43561	30.62
2		14.173	1552	98718	69.38

Figure S165: Chromatogram of **6p** after irradiation at 505 nm (100 µM, MeCN) extracted at 450 nm; eluent mixture: MeCN/water (3:7) containing 0.1% formic acid.

6p in water

385 nm

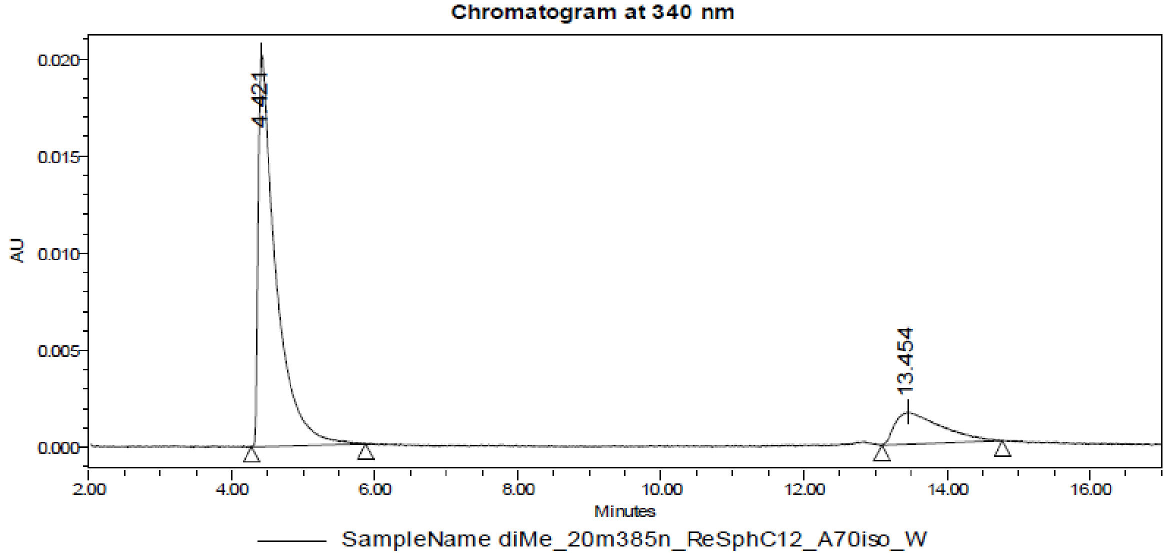

Peak Results

	Name	RT	Height	Area	% Area
1		4.421	20199	347608	82.33
2		13.454	1664	74620	17.67

Figure S166: Chromatogram of **6p** after irradiation at 385 nm (100 µM, water) extracted at 340 nm; eluent mixture: MeCN/water (3:7) containing 0.1% formic acid.

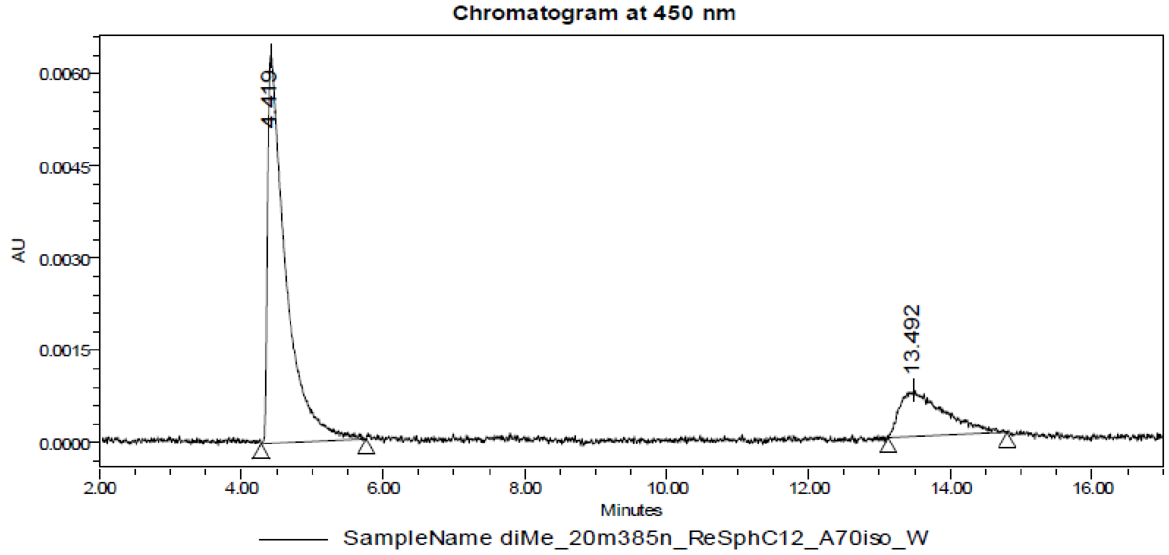

Peak Results

	Name	RT	Height	Area	% Area
1		4.419	6314	112757	78.01
2		13.492	750	31791	21.99

Figure S167: Chromatogram of **6p** after irradiation at 385 nm (100 µM, water) extracted at 450 nm; eluent mixture: MeCN/water (3:7) containing 0.1% formic acid.

505 nm

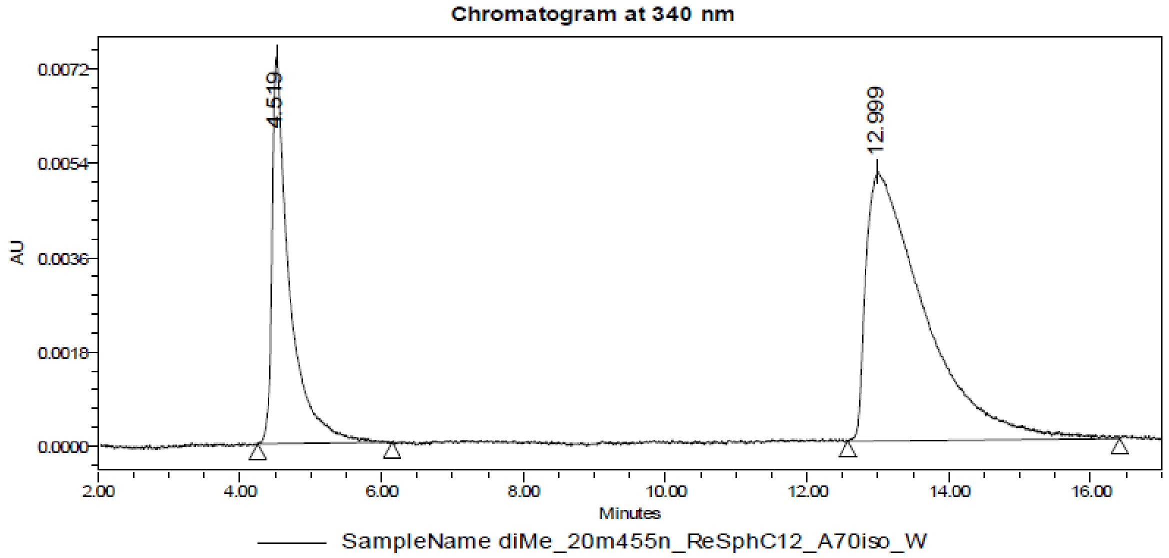

Peak Results

	Name	RT	Height	Area	% Area
1		4.519	7395	125827	29.87
2		12.999	5139	295466	70.13

Figure S168: Chromatogram of **6p** after irradiation at 505 nm (100 µM, water) extracted at 340 nm; eluent mixture: MeCN/water (3:7) containing 0.1% formic acid.

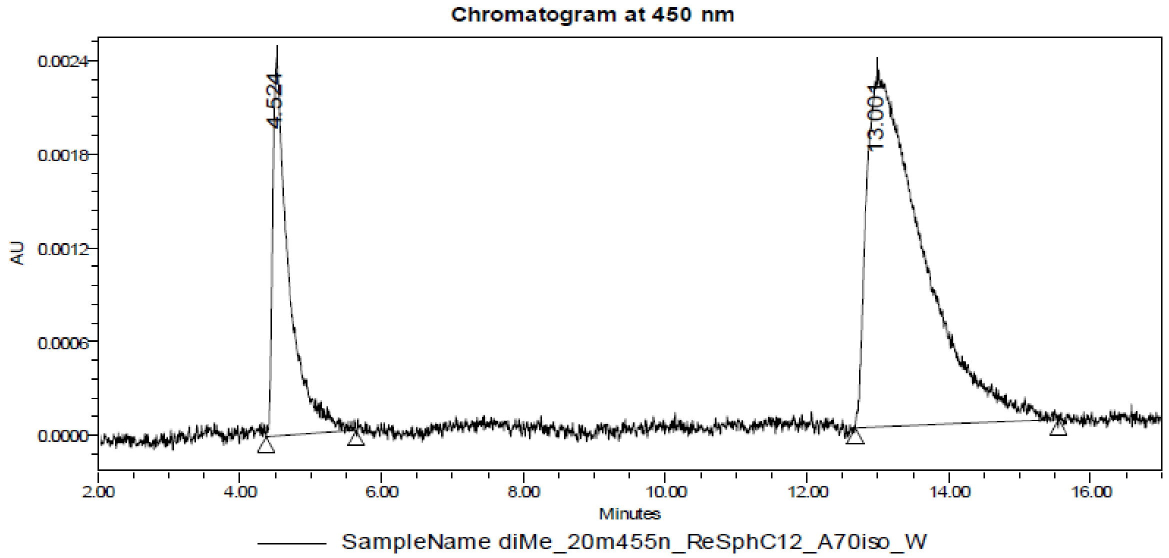

Peak Results

	Name	RT	Height	Area	% Area
1		4.524	2424	38592	23.41
2		13.001	2294	126255	76.59

Figure S169: Chromatogram of **6p** after irradiation at 505 nm (100 µM, water) extracted at 450 nm; eluent mixture: MeCN/water (3:7) containing 0.1% formic acid.

6q in water

385 nm

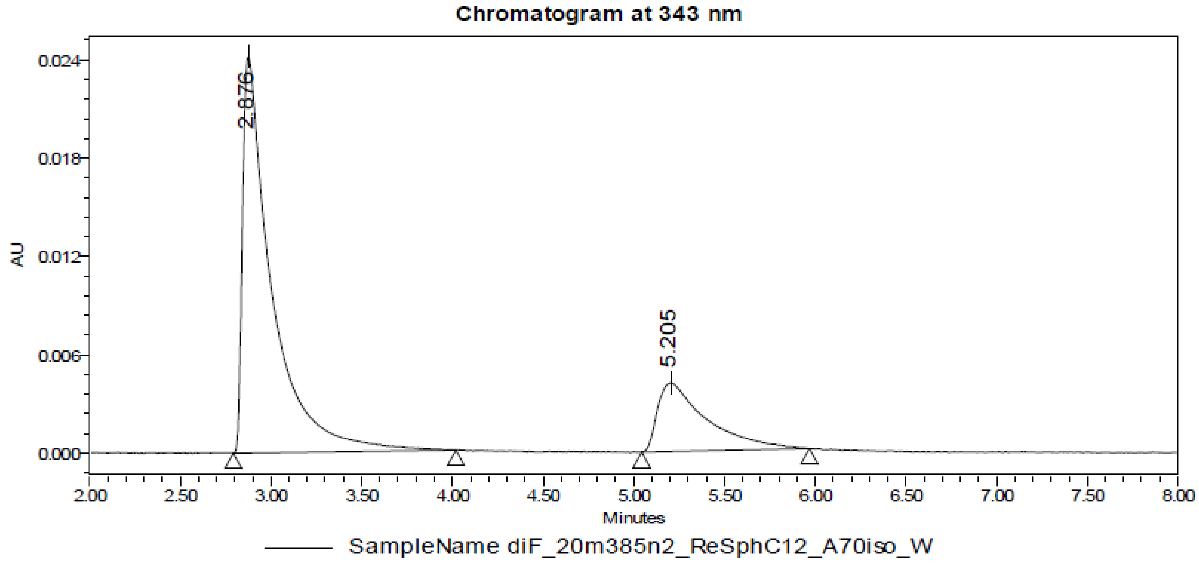

Peak Results

	Name	RT	Height	Area	% Area
1		2.876	24174	271141	77.43
2		5.205	4201	79021	22.57

Figure S170: Chromatogram of **6q** after irradiation at 385 nm (100 µM, water) extracted at 343 nm; eluent mixture: MeCN/water (3:7) containing 0.1% formic acid.

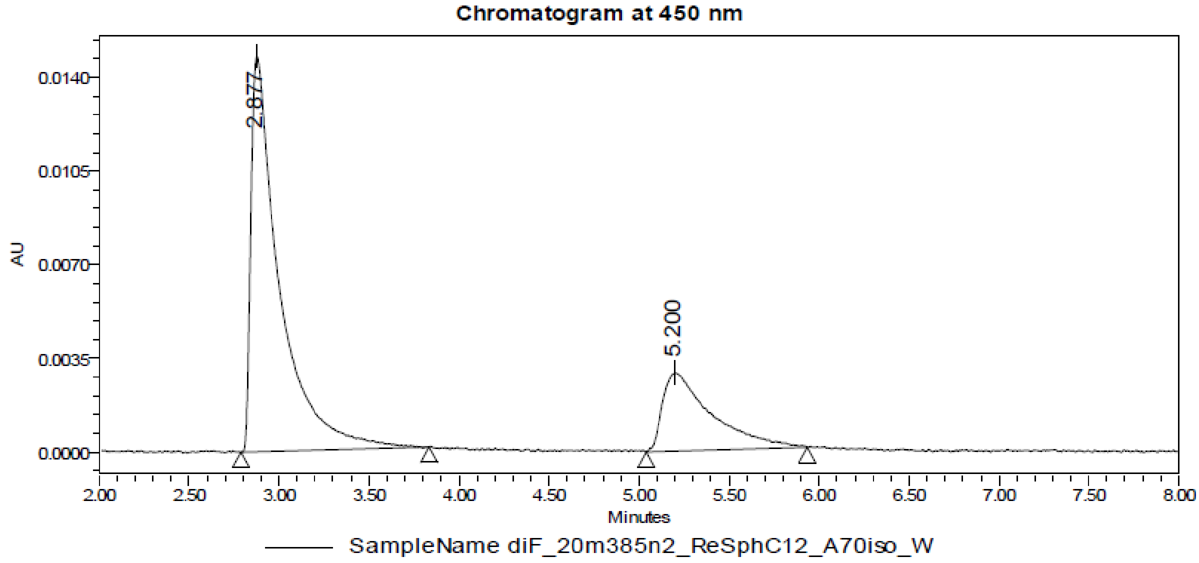

Peak Results

	Name	RT	Height	Area	% Area
1		2.877	14787	162922	74.76
2		5.200	2931	55004	25.24

Figure S171: Chromatogram of **6q** after irradiation at 385 nm (100 µM, water) extracted at 450 nm; eluent mixture: MeCN/water (3:7) containing 0.1% formic acid.

505 nm

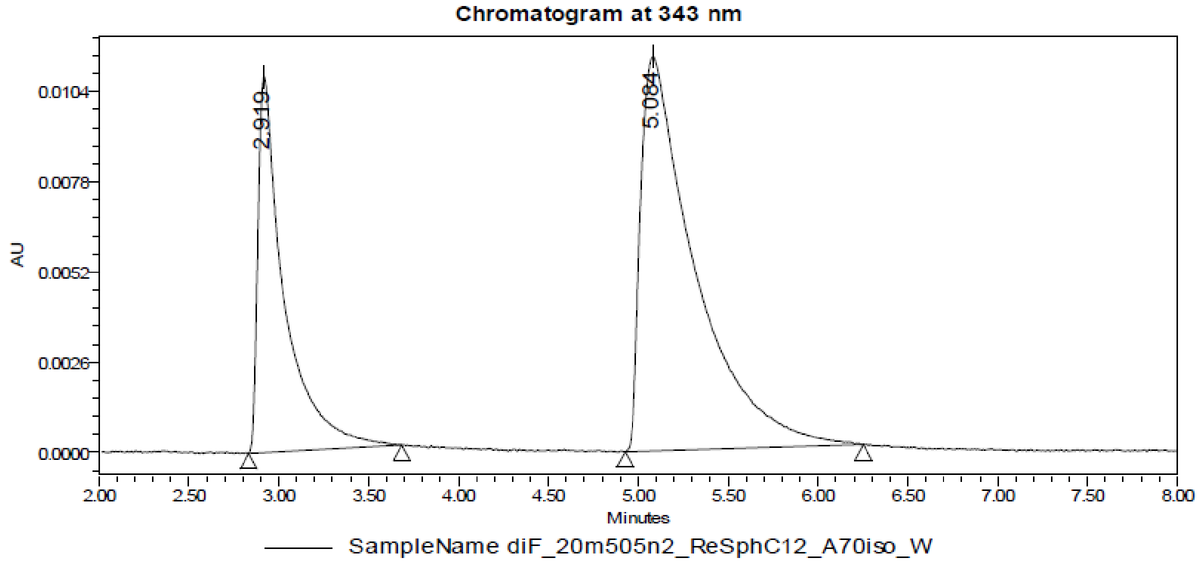

Peak Results

	Name	RT	Height	Area	% Area
1		2.919	10830	110478	31.60
2		5.084	11368	239158	68.40

Figure S172: Chromatogram of **6q** after irradiation at 385 nm (100 µM, water) extracted at 343 nm; eluent mixture: MeCN/water (3:7) containing 0.1% formic acid.

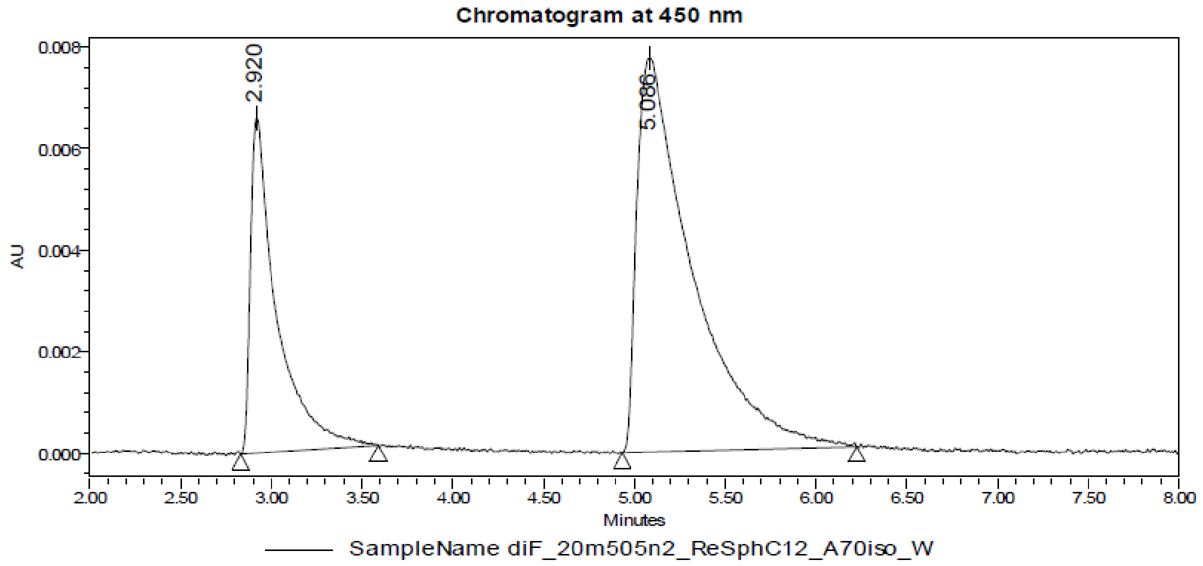

Peak Results

	Name	RT	Height	Area	% Area
1		2.920	6605	66549	28.78
2		5.086	7759	164680	71.22

Figure S173: Chromatogram of **6q** after irradiation at 505 nm (100 µM, water) extracted at 450 nm; eluent mixture: MeCN/water (3:7) containing 0.1% formic acid.

11a in water

415 nm

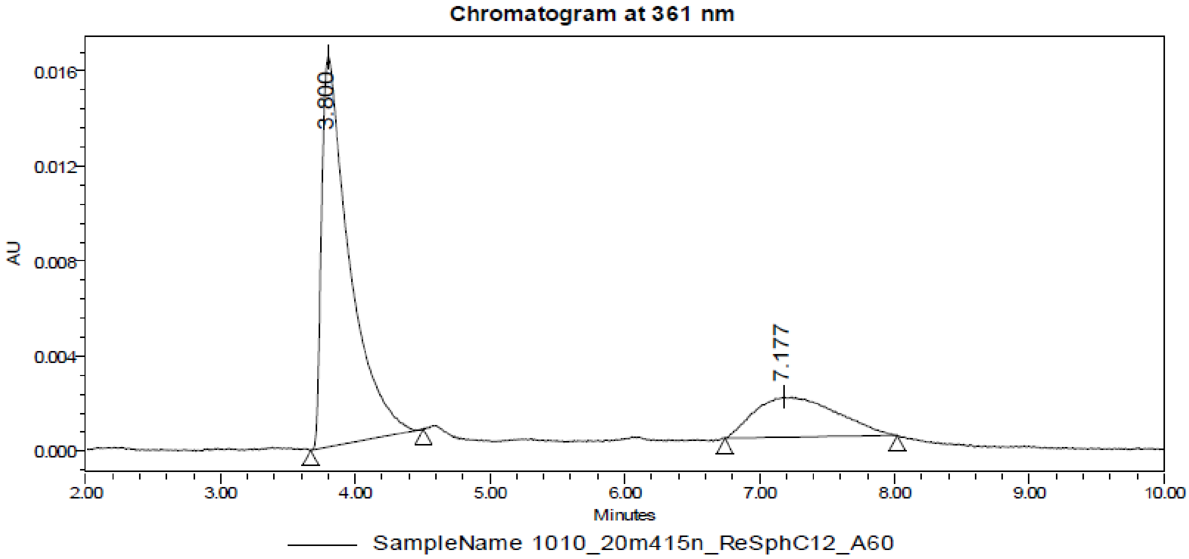

Peak Results

	Name	RT	Height	Area	% Area
1		3.800	16445	253348	78.17
2		7.177	1699	70767	21.83

Figure S174: Chromatogram of **11a** after irradiation at 415 nm (100 µM, water) extracted at 361 nm; eluent mixture: MeCN/water (4:6) containing 0.1% formic acid.

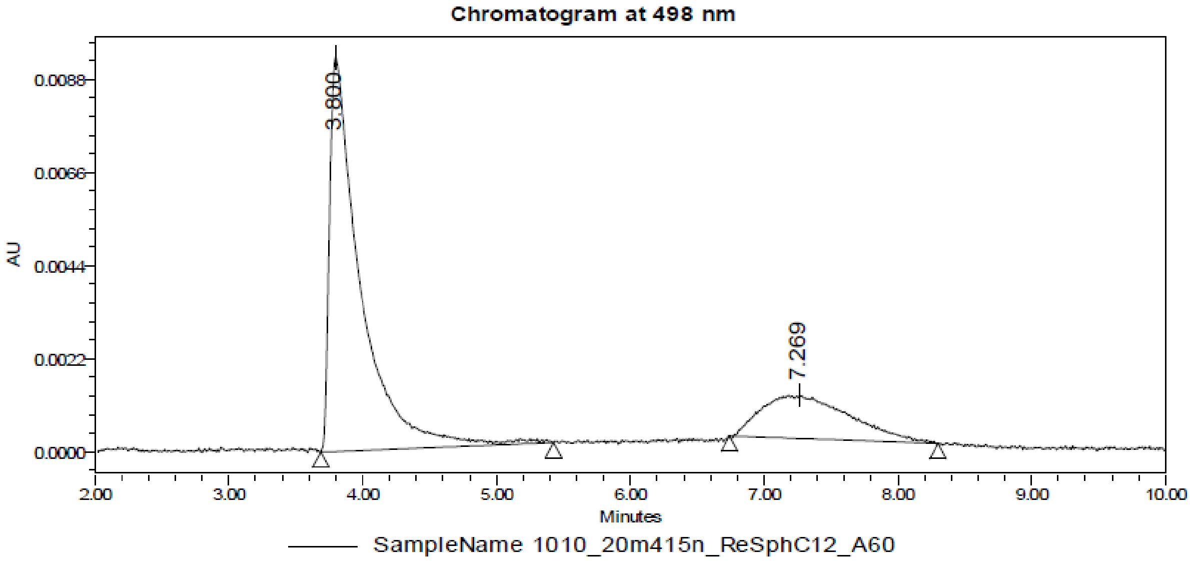

Peak Results

	Name	RT	Height	Area	% Area
1		3.800	9325	160844	77.67
2		7.269	1026	46239	22.33

Figure S175: Chromatogram of **11a** after irradiation at 415 nm (100 µM, water) extracted at 498 nm; eluent mixture: MeCN/water (4:6) containing 0.1% formic acid.

530 nm

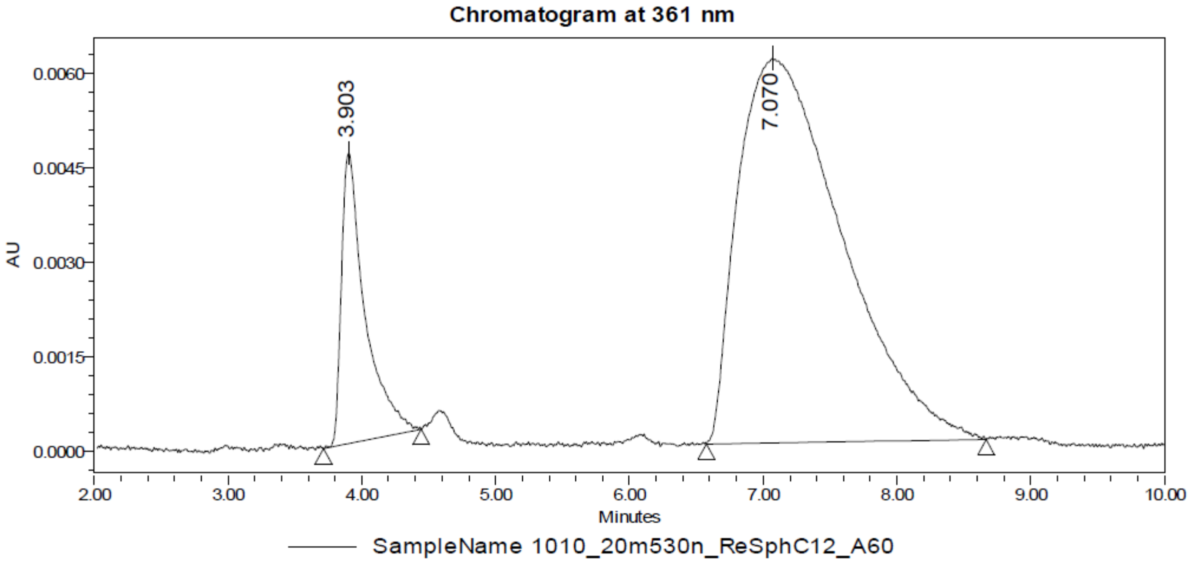

Peak Results

	Name	RT	Height	Area	% Area
1		3.903	4608	56266	13.76
2		7.070	6105	352678	86.24

Figure S176: Chromatogram of **11a** after irradiation at 530 nm (100 µM, water) extracted at 361 nm; eluent mixture: MeCN/water (4:6) containing 0.1% formic acid.

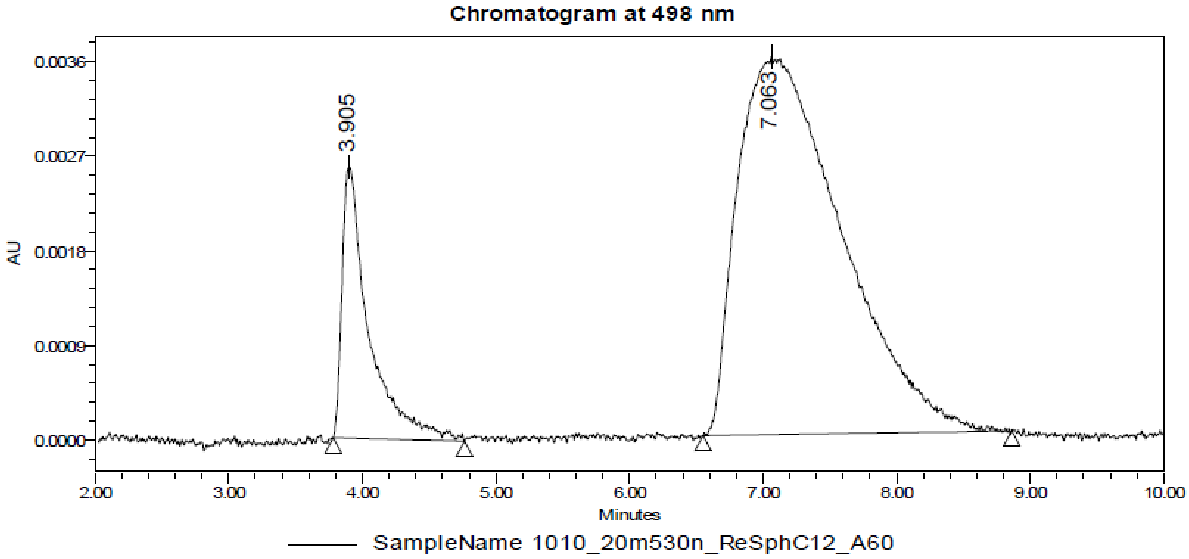

Peak Results

	Name	RT	Height	Area	% Area
1		3.905	2582	35533	14.61
2		7.063	3596	207711	85.39

Figure S177: Chromatogram of **11a** after irradiation at 530 nm (100 µM, water) extracted at 498 nm; eluent mixture: MeCN/water (4:6) containing 0.1% formic acid.

References

(1) Hanson, P.; Jones, J. R.; Taylor, A. B.; Walton, P. H.; Timms, A. W. Sandmeyer Reactions. Part 7.1 An Investigation into the Reduction Steps of Sandmeyer Hydroxylation and Chlorination Reactions. *Journal of the Chemical Society, Perkin Transactions 2* **2002**, No. 6, 1135–1150. https://doi.org/10.1039/b200748g.

(2) Kovács, S.; Bayarmagnai, B.; Aillerie, A.; Gooßen, L. J. Practical Reagents and Methods for Nucleophilic and Electrophilic Phosphorothiolations. *Adv Synth Catal* **2018**, *360* (10), 1913–1918. https://doi.org/10.1002/adsc.201701549.

(3) Pfaff, P.; Anderl, F.; Fink, M.; Balkenhohl, M.; Carreira, E. M. Azoacetylenes for the Synthesis of Arylazotriazole Photoswitches. *J Am Chem Soc* **2021**, *143*, 14495–14501. https://doi.org/10.1021/jacs.1c06014.

(4) Kloc, K.; Mlochowski, J.; Mhizha, S. Bis(2-Chlorosulfonylphenyl) Diselenide—the Substrate for Organoselenium Sulfonamides. *Synth Commun* **1997**, *27*, 4049–4057. https://doi.org/10.1080/00397919708005450.

(5) Ruck, R. T.; Huffman, M. A.; Stewart, G. W.; Cleator, E.; Kandur, W. V.; Kim, M. M.; Zhao, D. Route Development and Multikilogram GMP Delivery of a Somatostatin Receptor Antagonist. *Org Process Res Dev* **2012**, *16* (8), 1329–1337. https://doi.org/10.1021/op300128c.

(6) Carlin, R. B.; Carlson, D. P. The Fischer Indole Synthesis. VI. A Non-Aromatic Intermediate and a New Class of Hydroindoles [1]. *J Am Chem Soc* **1959**, *81* (17), 4673–4682. https://doi.org/10.1021/ja01526a057.

(7) Kanouni, T.; Stafford, J. A.; Veal, M. J.; Wallace, M. B. Histone Demethylase Inhibitors. US 20140171432A1, 2014.

(8) Thomas, G. B.; Rader, L. H.; Park, J.; Abezgauz, L.; Danino, D.; DeShong, P.; English, D. S. Carbohydrate Modified Catanionic Vesicles: Probing Multivalent Binding at the Bilayer Interface. *J Am Chem Soc* **2009**, *131* (15), 5471–5477. https://doi.org/10.1021/ja8076439.

(9) Schotten, C.; Leprevost, S. K.; Yong, L. M.; Hughes, C. E.; Harris, K. D. M.; Browne, D. L. Comparison of the Thermal Stabilities of Diazonium Salts and Their Corresponding Triazenes. *Org Process Res Dev* **2020**, *24* (10), 2336–2341. https://doi.org/10.1021/acs.oprd.0c00162.

(10) Yoshida, T.; Wada, Y.; Foster, N. Experimental Evaluation of Fire and Explosion Hazards of Reactive Substances. In *Safety of Reactive Chemicals and Pyrotechnics*; Elsevier Science, 1995; Vol. 5.

(11) Yoshida, T.; Yoshizawa, F.; Ito, M.; Matsunaga, T.; Watanabe, M.; Tamura, M. Prediction of Fire and Explosion Hazards of Reactive Chemicals (Part 1). Estimation of Explosive Properties of Self-Reactive Chemicals from SC-DSC Data. *Kogyo Kayaku* **1987**, *48* (5), 311–316.

(12) Sperry, J. B.; Minteer, C. J.; Tao, J.; Johnson, R.; Duzguner, R.; Hawksworth, M.; Oke, S.; Richardson, P. F.; Barnhart, R.; Bill, D. R.; Giusto, R. A.; Weaver, J. D. Thermal Stability Assessment of Peptide Coupling Reagents Commonly Used in Pharmaceutical Manufacturing. *Org Process Res Dev* **2018**, *22* (9), 1262–1275. https://doi.org/10.1021/acs.oprd.8b00193.

(13) Jelier, B. J.; Tripet, P. F.; Pietrasiak, E.; Franzoni, I.; Jeschke, G.; Togni, A. Radical Trifluoromethoxylation of Arenes Triggered by a Visible-Light-Mediated N−O Bond Redox Fragmentation. *Angewandte Chemie International Edition* **2018**, *57* (42), 13784–13789. https://doi.org/10.1002/anie.201806296.

(14) Yoshida, Z.; Tawara, Y. Aminocyclopropenium Ion. *J Am Chem Soc* **1971**, *93* (10), 2573–2574. https://doi.org/10.1021/ja00739a057.

(15) Lavallo, V.; Ishida, Y.; Donnadieu, B.; Bertrand, G. Isolation of Cyclopropenylidene–Lithium Adducts: The Weiss–Yoshida Reagent. *Angewandte Chemie International Edition* **2006**, *45* (40), 6652–6655. https://doi.org/10.1002/anie.200602701.

(16) Smajlagic, I.; Durán, R.; Pilkington, M.; Dudding, T. Cyclopropenium Enhanced Thiourea Catalysis. *J Org Chem* **2018**, *83* (22), 13973–13980. https://doi.org/10.1021/acs.joc.8b02321.

(17) Weiss, R.; Schloter, K. Stable Radical Dications. *Tetrahedron Lett* **1975**, *16* (40), 3491–3494. https://doi.org/10.1016/S0040-4039(00)91392-3.

(18) Xu, J.; Xian, A.; Li, Z.; Liu, J.; Zhang, Z.; Yan, R.; Gao, L.; Liu, B.; Zhao, L.; Guo, K. A Strained Ion Pair Permits Carbon Dioxide Fixation at Atmospheric Pressure by C–H H-Bonding Organocatalysis. *J Org Chem* **2021**, *86* (4), 3422–3432. https://doi.org/10.1021/acs.joc.0c02790.

(19) Wilcox, C.; Breslow, R. A Convenient Synthesis of Bis-Dialkylaminoacetylenes. *Tetrahedron Lett* **1980**, *21* (34), 3241–3242. https://doi.org/10.1016/S0040-4039(00)78656-4.

(20) Walst, K. J.; Yunis, R.; Bayley, P. M.; MacFarlane, D. R.; Ward, C. J.; Wang, R.; Curnow, O. J. Synthesis and Physical Properties of Tris(Dialkylamino)Cyclopropenium Bistriflamide Ionic Liquids. *RSC Adv* **2015**, *5* (49), 39565–39579. https://doi.org/10.1039/C5RA05254H.

(21) Landau, A.; Seitz, G. Pseudooxokohlenstoff-Anionen Der Semidreiecksäure. *Chem Ber* **1991**, *124* (3), 665–669. https://doi.org/10.1002/cber.19911240334.

(22) Kozma, Á.; Gopakumar, G.; Farès, C.; Thiel, W.; Alcarazo, M. Synthesis and Structure of Carbene-Stabilized N-Centered Cations $[L_2N]^+$, $[L_2NR]^{2+}$, $[LNR_3]^{2+}$, and $[L_3N]^{3+}$. *Chemistry – A European Journal* **2013**, *19* (11), 3542–3546. https://doi.org/10.1002/chem.201204186.

(23) Lavallo, V.; Ishida, Y.; Donnadieu, B.; Bertrand, G. Isolation of Cyclopropenylidene–Lithium Adducts: The Weiss–Yoshida Reagent. *Angewandte Chemie International Edition* **2006**, *45* (40), 6652–6655. https://doi.org/10.1002/anie.200602701.

(24) Wolter, M.; Klapars, A.; Buchwald, S. L. Synthesis of *N* -Aryl Hydrazides by Copper-Catalyzed Coupling of Hydrazides with Aryl Iodides. *Org Lett* **2001**, *3*, 3803–3805. https://doi.org/10.1021/ol0168216.

(25) Angelovski, G.; Keränen, M. D.; Linnepe, P.; Grudzielanek, S.; Eilbracht, P. A Rapid and Reliable Assay for Regioselectivity Using Fluorescence Spectroscopy. *Adv Synth Catal* **2006**, *348* (10–11), 1193–1199. https://doi.org/10.1002/adsc.200606047.

(26) Lam, M. S.; Lee, H. W.; Chan, A. S. C.; Kwong, F. Y. Copper(I)-Picolinic Acid Catalyzed N-Arylation of Hydrazides. *Tetrahedron Lett* **2008**, *49* (43), 6192–6194. https://doi.org/10.1016/j.tetlet.2008.08.050.

(27) Jiang, L.; Lu, X.; Zhang, H.; Jiang, Y.; Ma, D. CuI/4-Hydro- <scp>l</Scp> -Proline as a More Effective Catalytic System for Coupling of Aryl Bromides with *N* -Boc Hydrazine and Aqueous Ammonia. *J Org Chem* **2009**, *74* (12), 4542–4546. https://doi.org/10.1021/jo9006738.

(28) Bléger, D.; Dokić, J.; Peters, M. V.; Grubert, L.; Saalfrank, P.; Hecht, S. Electronic Decoupling Approach to Quantitative Photoswitching in Linear Multiazobenzene Architectures. *J Phys Chem B* **2011**, *115* (33), 9930–9940. https://doi.org/10.1021/jp2044114.

(29) Le Du, E.; Borrel, J.; Waser, J. Copper-Catalyzed Alkynylation of Hydrazides: An Easy Access to Functionalized Azadipeptides. *Org Lett* **2022**, *24* (36), 6614–6618. https://doi.org/10.1021/acs.orglett.2c02625.

(30) Rigaku Oxford Diffraction. *CrysAlisPro and ABSPACK*; 2016.

(31) Sheldrick, G. M. *SHELXT* – Integrated Space-Group and Crystal-Structure Determination. *Acta Crystallogr A Found Adv* **2015**, *71* (1), 3–8. https://doi.org/10.1107/S2053273314026370.

(32) Sheldrick, G. M. A Short History of *SHELX*. *Acta Crystallogr A* **2008**, *64* (1), 112–122. https://doi.org/10.1107/S0108767307043930.

(33) Sheldrick, G. M. Crystal Structure Refinement with *SHELXL*. *Acta Crystallogr C Struct Chem* **2015**, *71* (1), 3–8. https://doi.org/10.1107/S2053229614024218.

(34) Dolomanov, O. V.; Bourhis, L. J.; Gildea, R. J.; Howard, J. A. K.; Puschmann, H. *OLEX2*: A Complete Structure Solution, Refinement and Analysis Program. *J Appl Crystallogr* **2009**, *42* (2), 339–341. https://doi.org/10.1107/S0021889808042726.

(35) Folly, P. Thermal Stability of Explosives. *Chimia (Aarau)* **2004**, *58* (6), 394. https://doi.org/10.2533/000942904777677759.

(36) Kerckhoffs, A.; Christensen, K. E.; Langton, M. J. Fast Relaxing Red and Near-IR Switchable Azobenzenes with Chalcogen and Halogen Substituents: Periodic Trends, Tuneable Thermal Half-Lives and Chalcogen Bonding. *Chem Sci* **2022**, *13* (39), 11551–11559. https://doi.org/10.1039/D2SC04601F.
